# Global, regional, and national estimates and trends in stillbirths from 2000 to 2019: a systematic assessment

**DOI:** 10.1016/S0140-6736(21)01112-0

**Published:** 2021-08-28

**Authors:** Lucia Hug, Danzhen You, Hannah Blencowe, Anu Mishra, Zhengfan Wang, Miranda J Fix, Jon Wakefield, Allisyn C Moran, Victor Gaigbe-Togbe, Emi Suzuki, Dianna M Blau, Simon Cousens, Andreea Creanga, Trevor Croft, Kenneth Hill, K S Joseph, Salome Maswime, Elizabeth M McClure, Robert Pattinson, Jon Pedersen, Lucy K Smith, Jennifer Zeitlin, Leontine Alkema, UN Inter-agency Group for Child Mortality Estimation and its Core Stillbirth Estimation Group

**Affiliations:** a Division of Data, Analytics, Planning and Monitoring, UNICEF, New York, NY, USA; b Maternal, Adolescent, Reproductive & Child Health Centre, London School of Hygiene & Tropical Medicine, London, UK; c Department of Biostatistics and Epidemiology, University of Massachusetts Amherst, Amherst, MA, USA; d University of Washington, Seattle, WA, USA; e Department of Maternal, Newborn, Child and Adolescent Health, WHO, Geneva, Switzerland; f UN Population Division, New York, NY, USA; g Development Data Group, World Bank, Washington, DC, USA; h Centers for Disease Control and Prevention, Atlanta, GA, USA; i Johns Hopkins University, Baltimore, MD, USA; j The Demographic and Health Surveys Program, ICF, Rockville, MD, USA; k Stanton-Hill Research, Moultonborough, NH, USA; l University of British Columbia, Vancouver, BC, Canada; m Children's and Women's Hospital and Health Centre of British Columbia, Vancouver, BC, Canada; n University of Cape Town, Cape Town, South Africa; o RTI International, Research Triangle Park, NC, USA; p SAMRC/UP Maternal and Infant Health Care Strategies Unit, Department of Obstetrics and Gynaecology, University of Pretoria, Pretoria, South Africa; q Mikro, Oslo, Norway; r Department of Health Sciences, University of Leicester, Leicester, UK; s Inserm UMR 1153, Obstetrical, Perinatal and Pediatric Epidemiology Research Team, Center for Epidemiology and Statistics Sorbonne Paris Cité, DHU Risks in Pregnancy, Paris Descartes University, Paris, France

## Abstract

**Background:**

Stillbirths are a major public health issue and a sensitive marker of the quality of care around pregnancy and birth. The UN Global Strategy for Women's, Children's and Adolescents’ Health (2016–30) and the Every Newborn Action Plan (led by UNICEF and WHO) call for an end to preventable stillbirths. A first step to prevent stillbirths is obtaining standardised measurement of stillbirth rates across countries. We estimated stillbirth rates and their trends for 195 countries from 2000 to 2019 and assessed progress over time.

**Methods:**

For a systematic assessment, we created a dataset of 2833 country-year datapoints from 171 countries relevant to stillbirth rates, including data from registration and health information systems, household-based surveys, and population-based studies. After data quality assessment and exclusions, we used 1531 datapoints to estimate country-specific stillbirth rates for 195 countries from 2000 to 2019 using a Bayesian hierarchical temporal sparse regression model, according to a definition of stillbirth of at least 28 weeks’ gestational age. Our model combined covariates with a temporal smoothing process such that estimates were informed by data for country-periods with high quality data, while being based on covariates for country-periods with little or no data on stillbirth rates. Bias and additional uncertainty associated with observations based on alternative stillbirth definitions and source types, and observations that were subject to non-sampling errors, were included in the model. We compared the estimated stillbirth rates and trends to previously reported mortality estimates in children younger than 5 years.

**Findings:**

Globally in 2019, an estimated 2·0 million babies (90% uncertainty interval [UI] 1·9–2·2) were stillborn at 28 weeks or more of gestation, with a global stillbirth rate of 13·9 stillbirths (90% UI 13·5–15·4) per 1000 total births. Stillbirth rates in 2019 varied widely across regions, from 22·8 stillbirths (19·8–27·7) per 1000 total births in west and central Africa to 2·9 (2·7–3·0) in western Europe. After west and central Africa, eastern and southern Africa and south Asia had the second and third highest stillbirth rates in 2019. The global annual rate of reduction in stillbirth rate was estimated at 2·3% (90% UI 1·7–2·7) from 2000 to 2019, which was lower than the 2·9% (2·5–3·2) annual rate of reduction in neonatal mortality rate (for neonates aged <28 days) and the 4·3% (3·8–4·7) annual rate of reduction in mortality rate among children aged 1–59 months during the same period. Based on the lower bound of the 90% UIs, 114 countries had an estimated decrease in stillbirth rate since 2000, with four countries having a decrease of at least 50·0%, 28 having a decrease of 25·0–49·9%, 50 having a decrease of 10·0–24·9%, and 32 having a decrease of less than 10·0%. For the remaining 81 countries, we found no decrease in stillbirth rate since 2000. Of these countries, 34 were in sub-Saharan Africa, 16 were in east Asia and the Pacific, and 15 were in Latin America and the Caribbean.

**Interpretation:**

Progress in reducing the rate of stillbirths has been slow compared with decreases in the mortality rate of children younger than 5 years. Accelerated improvements are most needed in the regions and countries with high stillbirth rates, particularly in sub-Saharan Africa. Future prevention of stillbirths needs increased efforts to raise public awareness, improve data collection, assess progress, and understand public health priorities locally, all of which require investment.

**Funding:**

Bill & Melinda Gates Foundation and the UK Foreign, Commonwealth and Development Office.

## Introduction

Rate of stillbirth is regarded by the global health community as an important marker of a health system's quality of care during pregnancy and childbirth,^1^ but global monitoring of trends in stillbirth rate has been infrequent. Countries and the global health community have given notably less attention to this public health issue than to maternal and child mortality.^2^ Stillbirths are missing as a specific target in the Sustainable Development Goals agenda,^3^ despite being included in the Every Newborn Action Plan (ENAP) led by UNICEF and WHO^4^ and the UN Global Strategy for Women's, Children's and Adolescents’ Health 2016–30.^5^

**Research in context**

**Evidence before this study**

Before the release of estimates from the UN Inter-agency Group for Child Mortality Estimation, to our knowledge, the only global estimates of stillbirths were published by WHO (latest estimates in 2016) and the Global Burden of Disease Study (GBD; latest estimates in 2017). The previous estimates from WHO for developed countries with high quality data were obtained from stillbirth rate data directly, and smoothed with Loess regression. Estimates for all other countries were obtained from a regression model with country-specific intercepts and global regression coefficients. Stillbirth estimates from GBD 2017 were based on a space-time Gaussian process regression model for estimating the stillbirth rate to neonatal mortality rate ratio as a function of educational attainment of women of reproductive age, a non-linear function of the neonatal death rate, location random effects, and random effects for specific data source types nested within each location. The GBD approach used the definitional adjustments developed by WHO.

**Added value of this study**

In this study, we expanded the global stillbirth database and used a new approach to estimate stillbirth rates for all countries during the period 2000–19. We extended and updated the database with more than 2800 country-year datapoints from 171 countries, building on those provided by WHO in 2016. The new estimation model used a Bayesian hierarchical temporal sparse regression model. The model produced estimates that track high quality data while generating covariate-driven trend estimates for countries with limited or no data. The covariate selection extended previous work by introducing sparsity-inducing priors for estimating regression coefficients. The model also introduced new statistical approaches to address various data quality issues. First, we analysed observed stillbirth rate to neonatal mortality rate ratios for the population of interest and excluded ratios that suggested under-reporting of stillbirths as compared with neonatal deaths. Second, we introduced an estimation approach that incorporated observations with alternative definitions of stillbirth (eg, based on ≥22 weeks’ gestational age) into the model with mean adjustment and additional uncertainty associated with such observations. In the model fitting, we accounted for bias and varying sources of random error for data from different sources. Stillbirth rate estimates were obtained for 195 countries from 2000 to 2019. We also estimated stillbirth-associated indicators, such as rates of change, numbers of stillbirths, aggregated regional outcomes, and uncertainty intervals, and assessed time trends in stillbirths to monitor progress in prevention. The estimation method improved on previous methods by allowing for data-driven changes in stillbirth rate with time, taking biases and measurement errors into account and improving the method for covariate selection.

**Implications of all the available evidence**

We estimated that 2·0 million babies (1·9–2·2) were stillborn in 2019, with most stillbirths occurring in sub-Saharan Africa and south Asia. Progress in reducing stillbirth rates has been slower than progress in improving child survival. The number of stillbirths has increased in sub-Saharan Africa, with modest decreases in stillbirth rates negated by increases in the number of births. Closing gaps in the data and improving data quality in the regions with the highest stillbirth rates will be a necessary first step crucial to reducing the number of stillbirths. High quality stillbirth data are sparse in sub-Saharan Africa and south Asia; these regions accounted for only 17% of the stillbirth data used in the model, while three-quarters of the estimated stillbirths in 2019 occurred in these regions. Although the availability of stillbirth data has increased in recent years, further efforts are needed to improve data collection, data quality, and comparability. Improvements in stillbirth data quality have to occur concomitantly with improvements in women's health and in the provision of antenatal and intrapartum care services for women.

In many administrative registration systems of low-income and lower-middle-income countries, such as civil and vital registration systems or medical birth or death registries (if available), stillbirths are often not recorded, making it difficult to produce reliable and timely stillbirth data and statistics. Data on stillbirth rates in these countries, if available, typically come from health management information systems (HMIS), household surveys, and population-based studies.

The UN Inter-agency Group for Child Mortality Estimation (UN IGME), together with its Technical Advisory Group and Core Stillbirth Estimation Group, developed a set of annual stillbirth estimates for the years 2000–19 on the basis of administrative data, household surveys, and population-based study data.^6^ Building on the data and methods previously used by WHO and the London School of Hygiene and Tropical Medicine (London, UK),^7^, ^8^ we improved on previous estimations by expanding their database, estimating definitional adjustments, incorporating an assessment of data quality, and developing a robust estimation model. The initial method that used a regression model for countries with little data resulted in covariate-driven estimates and preselected covariates. With the new robust estimation model, we made use of the advantages of a Bayesian framework combining covariates with temporal smoothing to allow estimates to be data driven for countries where data on stillbirths are available. In this framework we also applied an improved covariate selection process.

In this paper, we estimated stillbirth rates and their trends for 195 countries from 2000 to 2019 on the basis of the work of the UN IGME. We present key findings on the burden of stillbirths and progress in preventing stillbirths at the global, regional, and national levels.

## Methods

### Design

For this systematic assessment, we present a summary of the source data and methods; a more detailed description of the methods is available in Wang et al.^9^

We created a database with 2833 country-year datapoints from 171 countries starting in the year 2000 up to the year 2019, updating and further developing the database used in 2016 by WHO.^7^ We extracted stillbirth rates from nationwide administrative registration systems such as vital registration systems, medical birth or death registries and HMIS, nationally representative household surveys with pregnancy histories or reproductive calendars, and population-based studies. Subnational population-based study data were sought for all countries without high coverage of routine administrative data from registration systems (appendix pp 3–5).

Definitional adjustment of stillbirth data was required, given that stillbirths were reported inconsistently in countries according to different combinations of definitional criteria, including gestational age, birthweight, or, occasionally, length at birth, and with varying thresholds (panel 1). In some instances, no clear criteria or thresholds were provided. These differences make it difficult to compare stillbirth rates and trends across countries and to calculate the global burden, as highlighted previously.^7^, ^13^, ^14^, ^15^ We estimated stillbirth rate using a 28 weeks’ gestation or more definition of stillbirth (panel 1). If information for the 28 weeks’ gestation definition was not available, adjustments and additional uncertainty associated with alternative definitions were accounted for in the model fitting.^9^ For each definitional conversion, we estimated the mean and variance associated with the ratio of the expected stillbirth rate based on an alternative definition, to the expected stillbirth rate based on the 28 weeks’ gestation or more definition. For low-income and middle-income countries (LMICs), high quality data from studies^16^, ^17^ were used to calculate adjustments and variance; for high-income countries (HICs), national administrative data were used. The World Bank Group income classification of countries from the year 2020 was used.

Panel 1 **Stillbirth definitions**

The term stillbirth generally applies to a baby born with no signs of life after a given viability threshold, with viability typically assessed on the basis of gestational age, birthweight, or length at birth. A stillbirth is defined as the birth of a baby following fetal death before labour (antepartum stillbirth) or during labour or birth (intrapartum stillbirth). Although most stillbirths occur within hours or days of fetal death, occasionally, such as in the case of twins, this can be delayed by months. For international comparisons of stillbirths, the International Classification of Diseases (ICD) definition (ICD 10th and 11th revisions)^10^, ^11^ of late fetal deaths is used. ICD defines late fetal death as the in-utero death of a baby (ie, born with no signs of life at birth) with a birthweight of 1000 g or more; or if birthweight is not available, at a gestational age of 28 weeks or more, or (if gestational age is not available), a body length of 35 cm or more at birth. Early fetal death is defined as the in-utero death of a baby (ie, born with no signs of life at birth) with a birthweight of 500–999 g; or, if birthweight is not available, at a gestational age at birth of 22–27 weeks, or a body length of 25–34 cm at birth. Since gestational age and birthweight thresholds do not perfectly correspond, the UN Inter-agency Group for Child Mortality Estimation (UN IGME) and the Core Stillbirth Estimation Group (CSEG) recommend the use of gestational age rather than birthweight to define a stillbirth. Gestational age is a better predictor of maturity and hence viability; of note, gestational age is the most commonly available criterion across data sources globally. Gestational age is typically measured from the first day of the last normal menstrual period,^10^ although in circumstances in which early ultrasound dating scans are available, gestational age should be based on the best obstetric estimate to avoid recall errors and differences in the length of menstrual cycles.^12^ Recommendations from the UN IGME and the CSEG also include omitting the birth length criterion and making a clearer distinction between stillbirth and fetal death. These recommendations are under review for inclusion in an updated edition of ICD-11.

Consistent with these recommendations, in this paper we defined a stillbirth as the birth of a baby with no signs of life at or after 28 weeks of gestation. When possible, data with a 28 weeks or more gestation definition were extracted. When data were collected according to a different definition (eg, based on birthweight or an alternative gestational age definition), stillbirth rates were adjusted in the modelling to allow for consistent international comparisons. For the estimates presented in this paper, stillbirth rate was defined as the number of stillbirths at 28 weeks’ gestation or more per 1000 total births (ie, livebirths plus stillbirths).

### Data quality and data exclusion

We assessed the quality of the various types and sources of stillbirth data by evaluating completeness and consistency. Data were excluded if the definition of stillbirth used or the method of data collection was not specified, more than 50% of reported stillbirths had unknown gestational age or birthweight, or coverage of livebirths in administrative registration data systems was estimated to be lower than 80% (or 75% for HMIS). Registration data with incomplete coverage of child deaths (<95%) were also excluded on the basis of WHO completeness assessments that used the same threshold.^18^ Additionally, data were excluded on the basis of external information that suggested some stillbirth rate observations were unreliable, for example due to poor quality of the data source, known data quality issues, undercapture of stillbirths, or inconsistency in reported numbers.

As part of the assessment of data quality, the plausibility of the ratio of stillbirth rate (measured according to the 28 weeks’ gestation or more definition) to neonatal mortality rate (for babies aged <28 days) from the same data source was determined. In the case of HMIS data, for which data on neonatal mortality rate might be less reliable than data on stillbirths as neonatal deaths are more likely to occur outside the health facility, or if the HMIS or other data source did not contain neonatal mortality rate, the UN IGME neonatal mortality rate estimates^19^ were used to calculate the ratio of stillbirth rate to neonatal mortality rate for assessment purposes. The UN IGME neonatal mortality rates were estimated within a Bayesian hierarchal framework at the country level and aggregated to region and global levels. The observed stillbirth rate to neonatal mortality rate ratios were compared to the distribution of ratios obtained from high quality LMIC study data.^16^, ^17^ We excluded observations with extremely low ratios using methods detailed in Wang et al.^9^ In summary, if stillbirths were under-reported relative to neonatal deaths for a country-year datapoint, the associated observed ratio of stillbirth rate to neonatal mortality rate would be lower than the true ratio. To quantify whether an observed ratio from our global dataset was extremely low, we calculated the probability of obtaining a ratio that is smaller than the observed ratio (taking account of the uncertainty associated with the observed ratio) using the distribution of ratios obtained from the high quality data. If this probability was less than 0·05, the observation was excluded from the database. This approach was applied to all observations in the database with 28 weeks’ gestation or more definitions and adjusted definitions (appendix pp 5–6).

Due to data quality concerns, 1302 (46·0%) of 2833 datapoints on stillbirths were excluded from the model (regional distribution shown in the appendix [p 4]). Among 195 countries for which we generated stillbirth estimates, 24 countries had no stillbirth data at all and 38 countries had no good quality stillbirth data, after excluding data according to our criteria (appendix pp 37–40).

### Estimation of stillbirth rates

We estimated stillbirth rates using a Bayesian hierarchical temporal sparse regression model for all country-years (appendix p 6). In the model, stillbirth rate was estimated assuming that the logarithm of the observed stillbirth rate plus adjustments and random measurement error equals the logarithm of the true stillbirth rate. Adjustments included those related to application of definition conversions and source type bias. Source type bias was equal to zero for all observations except for those from surveys, which were assumed to have a negative bias associated with them as surveys have been shown to underestimate stillbirths.^20^ Random measurement error referred to the sum of the stochastic or sampling error, the random definitional adjustment, and a random error related to source type. Each error was expected to be zero on average but included a variance term that reflected how much uncertainty was associated with the error. The stochastic or sampling error was due to not observing the complete population or survey sampling design. The random definitional adjustment error was non-zero for alternative definitions of stillbirth (ie, not the ≥28 weeks’ gestation definition) and followed from the analysis of the definitional adjustment ratios. The source type error referred to variances specific to source type, which accounted for random errors that might occur in the data collection process, and potential non-representativeness of observations. The distinct data source types considered in the model were administrative registration data (including vital registration systems and birth and death registries), HMIS, household surveys, and population-based studies.

The estimated stillbirth rate (on the logarithmic scale) for each country for the years 2000–19 was given by the sum of a regression function with a country-specific intercept and a country-specific temporal smoothing process. Resulting estimates were a weighted combination of information from adjusted country data and covariates associated with stillbirth rate, and accounted for the varying uncertainty associated with the adjusted observations. If data were precise when accounting for biases, uncertainty, and non-sampling errors, the stillbirth rate point estimates followed the adjusted country data. In cases of no data or imprecise data, the estimates were based on covariates. The uncertainty associated with the stillbirth rate estimates depended on data availability and precision for the respective country-period; uncertainty decreased as data availability and precision increased. Uncertainty in stillbirth rate estimates increased when extrapolating to periods without data.

The candidate covariates were based on a conceptual framework published in 2016 by Blencowe and colleagues.^7^ The framework included distal determinants such as socioeconomic factors, inter-related and overlapping demographic and biomedical factors (eg, adolescent fertility rate, maternal age, and malaria prevalence), perinatal outcome markers associated with stillbirth, and access to health care. The covariate data from household surveys, such as coverage of antenatal care visits and proportion of caesarean deliveries, were smoothed with a time-series trend to reduce small fluctuations in measured covariates. In the model fitting, regression coefficients for covariates with low predictive power were shrunk towards zero with sparsity-inducing priors^9^ as part of the Bayesian hierarchical temporal sparse regression model. The final covariates used in the model were neonatal mortality rate, low birthweight rate (on a logarithmic scale), coverage of four or more antenatal care visits, caesarean section rate, mean years of schooling of females, and gross national income per capita (on a logarithmic scale). A more detailed description of the model is available in Wang et al^9^ and covariate coefficients are available in the appendix (page 10).

We used a Hamiltonian Monte Carlo algorithm implemented with the use of Stan^21^ and R package RStan^22^ to generate samples from the posterior distributions of stillbirth rate.

### Computation and construction of estimates

Given the inherent uncertainty in stillbirth rate estimates, 90% uncertainty intervals (UIs) are used by the UN IGME instead of the more conventional 95% intervals. Although reporting intervals that are based on higher uncertainty (ie, 95% instead of 90%) would reduce the chance of not including the true value in the interval, the disadvantage of choosing higher uncertainty is that intervals lose their utility in presenting meaningful summaries of a range of likely outcomes when the indicator of interest is highly uncertain. The resulting UIs are not necessarily symmetrical around the point estimates, as stillbirth rates were estimated on the logarithmic scale, but reflect the uncertainty range associated with the stillbirth rates. The UIs for the number of stillbirths generated by the UN IGME do not account for uncertainty associated with other inputs required for calculation, such as the number of livebirths, because uncertainty assessments of these inputs are not yet available. The number of stillbirths was calculated from the number of livebirths estimated by the UN Population Division,^23^ according to the formula: number of stillbirths=livebirths × [stillbirth rate/(1 – stillbirth rate)]. The codes used in the model are available on request. We generated stillbirth rate estimates for 195 countries from 2000 to 2019. We produced regional aggregates from the country estimates and countries within these regions were defined according to UNICEF's regional classifications (appendix pp 7–8) and the World Bank 2020 income classification. We computed the percentage change and the annual rate of reduction with 90% UIs in the stillbirth rate and number of stillbirths for selected periods (2000–19, 2000–09, and 2010–19). The annual rate of reduction was defined as log(rate in t2/rate in t1)/(t1–t2), where t1 and t2 refer to different years (t1<t2). We compare the estimated progress in stillbirth rate with the UN IGME estimates for mortality among children younger than 5 years (neonatal mortality and child mortality at 1–59 months)^19^ and the WHO, UNICEF, UN Population Fund, World Bank Group, and UN Population Division estimates of maternal mortality ratio (number of maternal deaths per 100 000 livebirths).^24^ We also present the ratio of stillbirth rate to UN IGME neonatal mortality rate estimates (with 90% UIs).

### Role of the funding source

The funders of the study had no role in study design, data collection, data analysis, data interpretation, or writing of the report.

## Results

**Figure 1:**
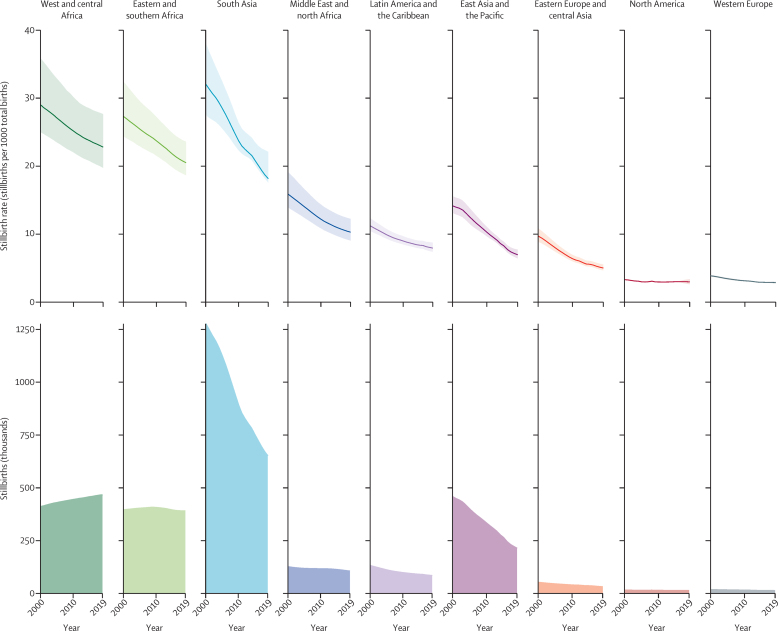
Stillbirth rates and numbers of stillbirths by region, 2000–19 Shading around the lines shows 90% uncertainty intervals.

**Figure 2:**
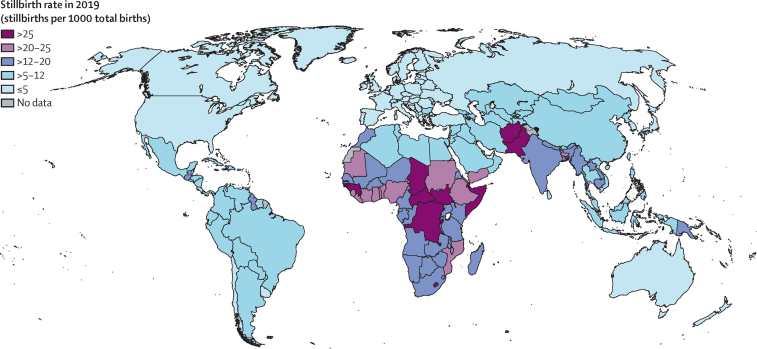
Stillbirth rates by country, 2019

Globally in 2019, an estimated 2·0 million babies (90% UI 1·9–2·2) were stillborn at 28 weeks or more of gestation, with a global stillbirth rate of 13·9 stillbirths (90% UI 13·5–15·4) per 1000 total births (table). Across regions in 2019, the highest stillbirth rate was estimated for west and central Africa at 22·8 stillbirths (19·8–27·7) per 1000 total births, followed by eastern and southern Africa at 20·5 (18·7–23·6) and south Asia at 18·2 (17·6–22·1; table, figure 1). The stillbirth rate in west and central Africa was almost 8 times higher than that in western Europe (2·9 [2·7–3·0]) and North America (3·0 [2·6–3·4]). Slightly more than three-quarters of global stillbirths (approximately 1·5 million) in 2019 occurred in the three regions with the highest stillbirth rates, with 33·1% (90% UI 31·5–37·8) occurring in south Asia, 23·7% (20·2–26·7) in west and central Africa, and 19·8% (17·4–21·7) in eastern and southern Africa. At the country level, the highest point estimates of stillbirth rate (>20 stillbirths per 1000 total births) were concentrated in countries in sub-Saharan Africa (23 of 49) and south Asia (three of eight; figure 2). In 2019, an estimated 83·6% (82·8–85·3) of all stillbirths, or 1·6 million (1·6–1·9), occurred in low-income and lower-middle income countries, while HICs accounted for only 1·9% (1·7–2·0) of the global burden of stillbirths (table). In 128 countries the stillbirth rate point estimate was 12 or fewer stillbirths per 1000 total births in 2019. Among those countries, 55 had point estimates of five or fewer stillbirths per 1000 total births, most of which (44 countries) were in Europe, central Asia, and North America (table, appendix pp 10–17, 43–237).

**Table:** Stillbirth rates and number of stillbirths globally and by region, 2000, 2010, and 2019

		**Stillbirth rate (stillbirths per 1000 total births)**				**Number of stillbirths (thousands)**				
		2000	2010	2019	Percentage decrease 2000 to 2019	2000	2010	2019	Percentage decrease 2000 to 2019	Share of total stillbirths worldwide in 2019
**Global**		**21·4 (20·0 to 23·7)**	**16·8 (16·2 to 18·0)**	**13·9 (13·5 to 15·4)**	**35·1% (27·2 to 39·6)**	**2880 (2688–3202)**	**2357 (2272–2538)**	**1966 (1919–2189)**	**31·7% (23·3 to 36·5)**	**100·0% (100·0–100·0)**
By region*										
Sub-Saharan Africa		28·2 (25·7 to 32·7)	24·5 (22·5 to 27·8)	21·7 (19·9 to 24·8)	23·0% (15·4 to 30·6)	805 (731 to 938)	850 (778 to 968)	856 (782 to 980)	−6·4% (−17·1 to 4·5)	43·6% (39·5 to 46·1)
	Eastern and southern Africa	27·3 (24·4 to 32·4)	23·7 (21·6 to 27·3)	20·5 (18·7 to 23·6)	24·9% (16·0 to 33·8)	395 (351 to 471)	406 (368 to 468)	390 (355 to 450)	1·2% (−10·9 to 13·2)	19·8% (17·4 to 21·7)
	West and central Africa	29·0 (25·0 to 35·9)	25·2 (22·1 to 30·1)	22·8 (19·8 to 27·7)	21·4% (9·0 to 32·9)	410 (352 to 510)	444 (387 to 534)	466 (403 to 568)	−13·7% (−32·2 to 3·4)	23·7% (20·2 to 26·7)
Middle East and north Africa		15·9 (14·0 to 19·2)	12·2 (10·9 to 14·4)	10·3 (9·1 to 12·3)	35·3% (25·8 to 43·9)	125 (110 to 151)	116 (103 to 136)	105 (92 to 125)	16·4% (4·1 to 27·7)	5·3% (4·5 to 6·1)
South Asia		32·1 (27·6 to 38·1)	23·7 (22·3 to 26·4)	18·2 (17·6 to 22·1)	43·4% (26·3 to 50·1)	1276 (1092 to 1526)	893 (839 to 995)	651 (630 to 796)	49·0% (33·2 to 55·3)	33·1% (31·5 to 37·8)
East Asia and the Pacific		14·2 (13·1 to 15·6)	10·3 (9·7 to 11·1)	7·0 (6·4 to 7·7)	50·8% (45·1 to 55·9)	458 (424 to 504)	333 (313 to 358)	213 (196 to 236)	53·5% (48·0 to 58·3)	10·8% (9·5 to 11·6)
Latin America and the Caribbean		11·2 (10·5 to 12·4)	9·0 (8·5 to 9·7)	7·9 (7·4 to 8·8)	29·3% (22·1 to 36·1)	131 (122 to 144)	98 (92 to 105)	83 (78 to 92)	36·3% (29·8 to 42·5)	4·2% (3·7 to 4·6)
North America		3·3 (3·2 to 3·4)	3·0 (2·9 to 3·0)	3·0 (2·6-3·4)	9·8% (−2·6 to 20·6)	14 (14 to 15)	13 (13 to 14)	13 (11 to 15)	9·3% (−3·3 to 20·2)	0·7% (0·5 to 0·7)
Europe and central Asia		6·9 (6·5 to 7·5)	4·9 (4·7 to 5·2)	4·1 (3·9 to 4·4)	41·2% (36·7 to 45·5)	70 (66 to 77)	55 (53 to 59)	44 (42 to 48)	37·1% (32·2 to 41·8)	2·3% (2·0 to 2·4)
	Eastern Europe and central Asia	9·7 (8·9 to 10·9)	6·4 (6·0 to 7·0)	5·0 (4·7 to 5·5)	48·4% (43·0 to 53·4)	52 (47 to 58)	39 (37 to 42)	30 (28 to 34)	41·1% (34·8 to 46·8)	1·5% (1·3 to 1·7)
	Western Europe	3·9 (3·7 to 4·0)	3·1 (3·1 to 3·2)	2·9 (2·7 to 3·0)	25·7% (21·1 to 29·9)	19 (18 to 20)	16 (16 to 17)	14 (13 to 15)	26·4% (21·9 to 30·5)	0·7% (0·6 to 0·7)
**By income group**†										
Low income		29·5 (26·6 to 34·7)	25·7 (23·6 to 29·2)	22·7 (20·9 to 25·6)	23·1% (15·1 to 31·7)	516 (464 to 610)	534 (489 to 610)	532 (490 to 603)	−3·1% (−14·2 to 8·7)	27·1% (24·1 to 29·0)
Lower-middle income		28·0 (25·2 to 32·3)	21·2 (20·1 to 23·4)	17·1 (16·5 to 20·0)	38·9% (26·5 to 45·0)	1759 (1577 to 2037)	1370 (1296 to 1514)	1111 (1073 to 1303)	36·8% (23·7 to 43·3)	56·5% (54·8 to 60·4)
Upper-middle income		13·4 (12·6 to 14·4)	9·7 (9·3 to 10·3)	7·0 (6·6 to 7·6)	47·6% (42·7 to 51·9)	554 (522 to 599)	409 (391 to 433)	285 (269 to 309)	48·6% (43·8 to 52·9)	14·5% (12·9 to 15·3)
High income		3·9 (3·8 to 4·2)	3·3 (3·2 to 3·4)	3·0 (2·8 to 3·2)	24·4% (19·4 to 28·9)	51 (49 to 54)	43 (42 to 46)	38 (36 to 40)	25·9% (21·0 to 30·4)	1·9% (1·7 to 2·0)

Numbers in parentheses are 90% uncertainty intervals.

* UNICEF regional classifications (appendix pp 7–8).

† World Bank Group 2020 classification.

Since the year 2000, the global stillbirth rate decreased by 35·1% (90% UI 27·2 to 39·6), from 21·4 stillbirths (20·0–23·7) per 1000 total births in 2000 to 13·9 (13·5–15·4) in 2019 (table, figure 1). The greatest decrease in stillbirth rate was estimated in east Asia and the Pacific, with a 50·8% (45·1 to 55·9) decrease in rate, followed by eastern Europe and central Asia (48·4% [43·0 to 53·4]) and south Asia (43·4% [26·3 to 50·1]). Excluding North America, the least progress was estimated in west and central Africa, which had a 21·4% (9·0 to 32·9) decrease in stillbirth rate, followed by eastern and southern Africa (24·9% [16·0 to 33·8]). North America showed consistently low stillbirth rates from 2000 to 2019, leading to an estimated percentage decrease of 9·8% (–2·6 to 20·6). Among the 195 countries analysed, based on point estimates, 115 countries reduced their stillbirth rate by at least 25·0% from 2000 to 2019 and, among these, 14 more than halved their stillbirth rate. Among the other 80 countries with a reduction of less than 25·0% in stillbirth rate since 2000, the largest group (n=32) were located in sub-Saharan Africa (appendix pp 18–27). Based on the lower bound of the 90% UIs, we estimated that 114 countries had a decrease in stillbirth rate since 2000, with four countries having a decrease of at least 50·0%, 28 having a decrease of 25·0–49·9%, 50 having a decrease of 10·0–24·9%, and 32 having a decrease of less than 10·0%. For the remaining 81 countries, we found no decrease in stillbirth rate since 2000 after taking the lower bound of uncertainty into account. Of these countries, 34 were in sub-Saharan Africa, 16 were in east Asia and the Pacific, 15 were in Latin America and the Caribbean, and 16 were located across the remaining regions (appendix pp 18–27).

**Figure 3:**
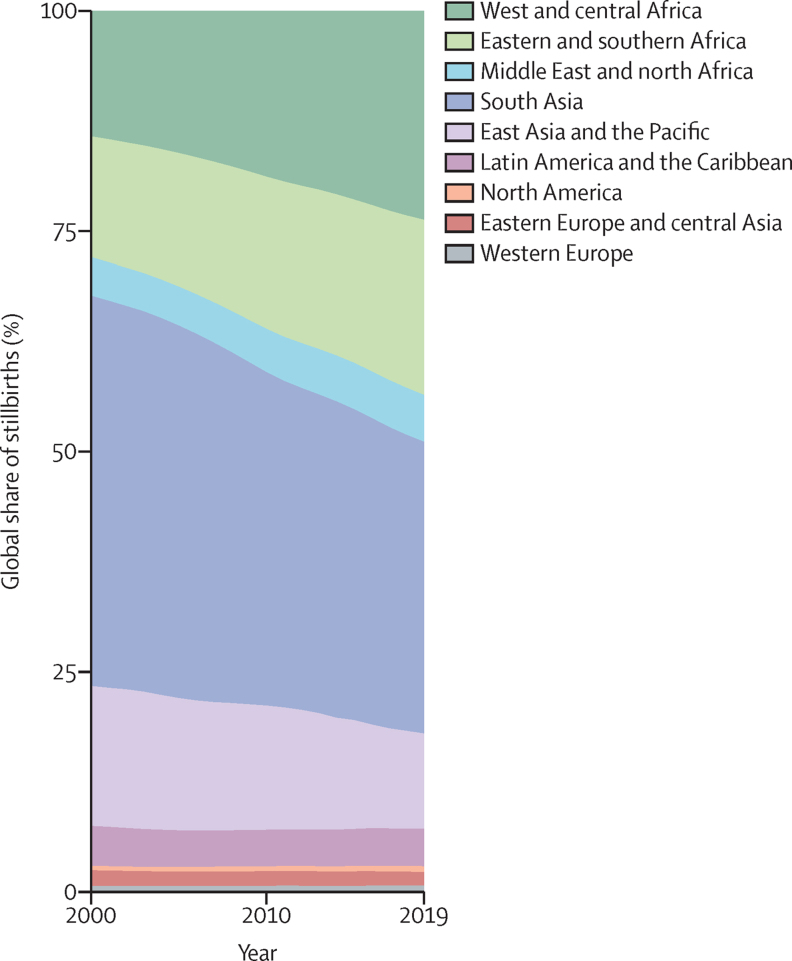
Global share of stillbirths by region, 2000–19

In the 20 years since 2000, an estimated 48·2 million stillbirths (90% UI 46·8–52·0) occurred. Over this period, the global number of stillbirths decreased by 31·7% (90% UI 23·3 to 36·5), with an absolute decrease of 0·9 million (0·6–1·2; table)**.** The estimated global number of livebirths increased by 6·1% over the same 20 years.^23^ West and central Africa showed a small percentage increase of 13·7% (–3·4 to 32·2) in the number of stillbirths and eastern and southern Africa showed almost no change (with a percentage decrease of 1·2% [–10·9 to 13·2]), although the rate of stillbirth decreased in both regions. In the same period, the number of livebirths increased substantially in these two regions of sub-Saharan Africa, by 45·6% in west and central Africa and 32·6% in eastern and southern Africa. Number of stillbirths decreased in south Asia by 49·0% (33·2 to 55·3), although number of livebirths also decreased by 8·6%.^23^ Other regions with decreases in livebirths from 2000 to 2019 were Latin America and the Caribbean (9·6%), east Asia and the Pacific (4·7%), and western Europe (0·8%). The number of stillbirths decreased in other regions, and the proportionate distribution of global stillbirths shifted during 2000–19 towards the regions in sub-Saharan Africa, with the combined share of stillbirths in west and central Africa and eastern and southern Africa increasing from 28·0% (25·1–31·7) in 2000 to 43·6% (39·5–46·1) in 2019 (figure 3). This share for south Asia decreased from 44·3% (39·7 to 48·5) to 33·1% (31·5 to 37·8). At the country level, more than a third of all stillbirths in 2019 occurred in three countries: India (17·3% [16·4–22·4]), Pakistan (9·7% [7·8–11·1]), and Nigeria (8·7% [5·7–12·3]; appendix pp 18–27).

**Figure 4:**
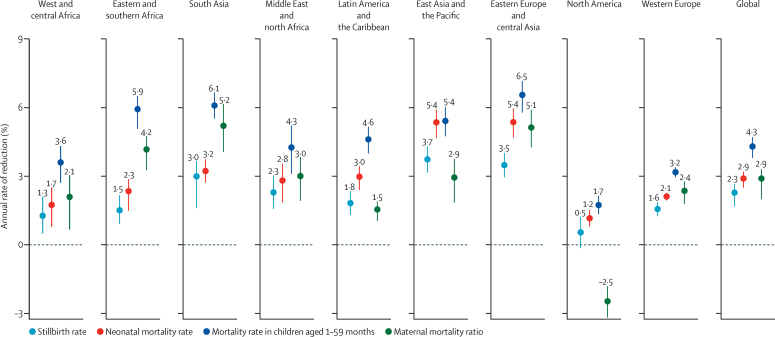
Annual rates of reduction in mortality outcomes globally and by region, 2000–19* Horizontal bars are UIs. Maternal mortality ratio is shown with 80% UIs. All other indicators use 90% UIs. UI=uncertainty interval. *The annual rate of reduction for maternal mortality ratio refers to the years 2000–17.

Progress in reducing the rate of stillbirth was slow compared with decreases in mortality rate among children younger than 5 years^19^ and maternal mortality ratio.^24^ The global annual rate of reduction in stillbirth rate was estimated at 2·3% (90% UI 1·7–2·7) from 2000 to 2019, which was lower than the 2·9% (2·5–3·2) estimated for neonatal mortality rate and 4·3% (3·8–4·7) estimated for mortality rate among children aged 1–59 months during the same period.^19^ Additionally, maternal mortality ratio showed an annual rate of reduction of 2·9% (80% UI 2·0–3·3) from 2000 to 2017^24^ (figure 4). Across all regions, progress in reducing stillbirth rate was slow compared with progress in reducing neonatal mortality rate. The difference was more pronounced when comparing with mortality rate in children aged 1–59 months, for which the annual rate of reduction was at least 1·4 times greater than the annual rate of reduction in stillbirth rate across all regions. In eastern and southern Africa, the annual rate of reduction in mortality rate among children aged 1–59 months was almost 4 times higher, at 5·9% (5·1–6·5) versus 1·5% (0·9–2·2) for stillbirth rate (figure 4). Based on point estimates, only 14 countries reduced stillbirth rate by 50% or more during 2000–19, compared with 117 countries that at least halved mortality rate among children aged 1–59 months, and 49 countries that at least halved neonatal mortality rate. Based on the lower bound of the 90% UIs, only four countries reduced stillbirth rate by at least half, compared with 59 countries that at least halved mortality rate among children aged 1–59 months, and 19 countries that at least halved neonatal mortality rate (appendix pp 18–27).

**Figure 5:**
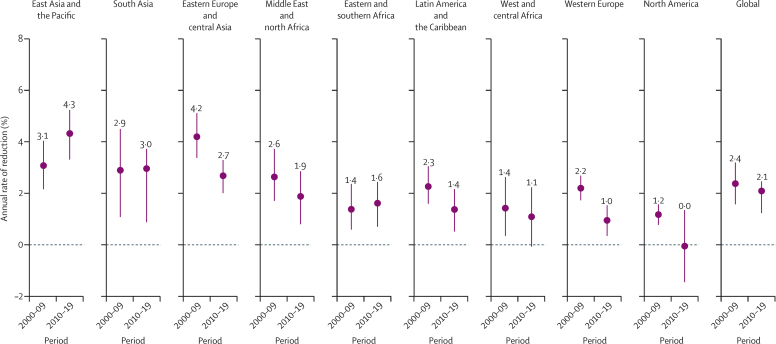
Annual rates of reduction in stillbirth rate globally and by region, 2000–09 and 2010–19 Horizontal bars are 90% uncertainty intervals.

Progress in reducing stillbirth rate did not accelerate in the period from 2010 to 2019: the annual rate of reduction in the global stillbirth rate was 2·4% (1·6–3·2) from 2000 to 2009 and 2·1% (1·2–2·5) from 2010 to 2019 (figure 5). However, in east Asia and the Pacific, the annual rate of reduction increased from 3·1% (2·2–4·1) in the first decade to 4·3% (3·3–5·3) in the second decade. Two other regions had an increase in the annual rate of reduction: eastern and southern Africa, with an annual rate of reduction of 1·4% (0·6–2·4) in 2000–09 versus 1·6% (0·7–2·5) in 2010–19, and south Asia, with an annual rate of reduction of 2·9% (1·1–4·5) in the first decade versus 3·0% (0·9–3·7) in the second decade. All other regions had lower point estimates for the annual rate of reduction during 2010–19 versus 2000–09.

**Figure 6:**
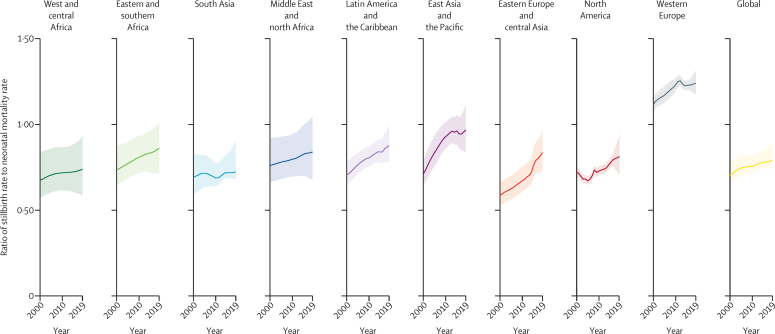
Ratio of stillbirth rate to neonatal mortality rate globally and by region, 2000–19 Shading shows 90% uncertainty intervals.

Globally, the estimated ratio of stillbirth rate to neonatal mortality rate was 0·79 (90% UI 0·74–0·89) in 2019, increasing from 0·70 (0·66–0·78) in 2000 (figure 6). Across regions in 2019, this ratio was greatest in western Europe (1·24 [1·17–1·31]), followed by east Asia and the Pacific (0·97 [0·84–1·12]); the ratio was lowest in west and central Africa (0·74 [0·59–0·93]) and south Asia (0·72 [0·68–0·91]). The ratio of stillbirth rate to neonatal mortality rate increased from the year 2000 for all regions. The smallest change was in south Asia, and the largest increases were in east Asia and the Pacific and eastern Europe and central Asia. At the country level, the stillbirth rate to neonatal mortality rate ratio ranged from 0·36 (0·21–0·64) to 3·17 (2·60–3·75) in 2019, with a pattern of higher ratios in countries with lower stillbirth rates (appendix pp 18–27, 41–42). In many of the high-burden countries with the largest number of stillbirths, the ratios were lower than in countries with lower numbers of stillbirths (appendix p 41).

## Discussion

Reliable estimates are essential to inform programming, research, and policy, and with our model we have improved the collection of comparable input data and the modelling approach to produce reliable estimates for stillbirths for 195 countries. The robust stillbirth estimates from our modelling can be used to guide programming, research, and policy, with an ultimate aim to end preventable stillbirths, and to highlight data needs around stillbirths.

The loss of a baby late in pregnancy remains too common. Although the burden is immeasurable for women, families, and society, stillbirths remain a largely unseen and unaddressed problem. In the 20 years since 2000, an estimated 48·2 million stillbirths occurred globally, with an estimated 2 million women and families experiencing a stillbirth in 2019. The slower progress in preventing stillbirths, compared with reductions in neonatal mortality rate and mortality rate in children aged 1–59 months, highlights the insufficient effort and investments in ending preventable stillbirths. Furthermore, our estimates did not show acceleration in preventing stillbirths in the past 10 years globally. Urgent action is needed to accelerate progress and meet the ENAP target of reducing stillbirth rate to 12 or fewer stillbirths per 1000 total births in every country by 2030.^4^ National aggregate rates are not an accurate reflection of geographical heterogeneity and sociodemographic inequalities in rates, and, therefore, countries already meeting the ENAP target should work to reduce equity gaps. If current trends continue, a total of 19·5 million babies will be stillborn in 2020–30, a great tragedy for too many babies, women, and families.^6^

West and central Africa, eastern and southern Africa, and south Asia are the regions with the highest numbers and burden of stillbirth. In 2019, the risk of a stillbirth in west and central Africa, eastern and southern Africa, and south Asia was around 8 times, 7 times, and 6 times higher, respectively, than in western Europe. Despite modest decreases in stillbirth rate in the two regions of sub-Saharan Africa, the number of stillbirths has levelled off because small decreases in stillbirth rate were negated by the increase in the number of births. Concerted efforts are needed to accelerate progress in sub-Saharan Africa and south Asia. With current trends, 56 countries worldwide will not meet the ENAP target by 2030, 48 of which are located in these high-burden regions. 34 countries are projected to meet the target only after 2050, 28 of which are in sub-Saharan Africa.^6^

Ending preventable stillbirths does not necessarily require new or innovative interventions. Globally, an estimated 42·3% (95% UI 41·3–46·1) of all stillbirths are intrapartum and almost all of these can be prevented with timely, quality care during childbirth, including ongoing intrapartum monitoring and timely intervention in case of complications (panel 2).^6^ Many antepartum stillbirths are also preventable, as long as evidence-based interventions that improve the health of mothers and their babies along the continuum of care, including during antenatal care, are available and can be accessed. Health system strengthening is as important as clinical interventions in stillbirth prevention to ensure that women and babies at risk can access timely, high quality care, including emergency obstetric care and caesarean section if required. Perinatal audits are a potential useful tool to improve the suboptimal care that contributes to many stillbirths, but these audits need to be implemented as part of an intervention package that includes training and adequate resources, supervision, and mentorship.^26^, ^27^

Panel 2 **Timing of stillbirth**

Globally, an estimated 832 000 stillborn babies (95% uncertainty interval [UI] 811 000–990 000) died during labour (intrapartum) in 2019, accounting for an estimated 42·3% (95% UI 41·3–46·1) of all stillbirths.^6^ About half of all stillbirths in the two regions of sub-Saharan Africa (47·7% in eastern and southern Asia and 50·7% in west and central Africa) and in south Asia (49·5%) were intrapartum, compared with about 6·4% in western Europe and North America.^6^ Overall, an estimated 745 000 babies (712 00–897 000) died during labour in 2019 in sub-Saharan Africa and south Asia, accounting for 89·5% of all intrapartum stillbirths worldwide.^6^

Intrapartum stillbirth is a sensitive marker of the timeliness and quality of intrapartum care. Action is urgently needed in the regions of sub-Saharan Africa and south Asia to provide interventions, particularly during labour, that could save lives. Gaps in the availability and quality of data have posed challenges to understanding the true burden of intrapartum stillbirths, contributing to insufficient action to reduce such mortality. Although information on intrapartum stillbirths is recorded in birth registers in most health facilities, in many countries appearance of the skin (fresh rather than macerated) is used as a surrogate marker for intrapartum stillbirth.^25^ However, this is an unreliable measure. Furthermore, these data are infrequently collated at a national level.

Although the majority of causes of stillbirths can now be prevented with available interventions, further research is needed to improve the prevention, detection, and management of fetal growth restriction, an important factor in many antepartum stillbirths.^28^ Currently, the detection of fetal growth restriction is poor, especially in LMICs,^29^, ^30^, ^31^ and, when detected, the potential gains with current approaches (ie, monitoring fetuses with low fetal weight or biometric parameters) are fairly modest and might result in obstetric intervention and iatrogenic prematurity with its associated risks. One new approach is screening of low-risk pregnancies with continuous wave Doppler ultrasound, which can improve detection of fetal growth restriction (unpublished data) and reduce stillbirth rates.^32^

This paper provides a comprehensive analysis of global stillbirth trends based on the latest data for 195 countries. We build on the previous methods of WHO, making greater use of the available input country-level data and improving the covariate selection process in a Bayesian framework for a better prediction. However, our estimates have several limitations. First, the scarcity of data available limits our ability to make precise estimates. High quality stillbirth data are in particularly short supply in the sub-Saharan Africa and south Asia regions, which accounted for only 17% of the stillbirth data used in the model but were responsible for three-quarters of the estimated stillbirths in 2019. Countries in west and central Africa had, on average, 2·0 datapoints included for the past 20 years, while countries in western Europe had 16·4 datapoints available. Limited data availability results in considerable uncertainty in the estimates. The stillbirth rate estimates are more uncertain than other child survival indicators and, although from point estimates, 115 countries reduced their stillbirth rate by at least a quarter since 2000, only 32 countries achieved that reduction with 95% probability. In the absence of empirical country-year observations, the estimates were covariate driven. Covariates were selected on the basis of both a conceptual framework and predictive power, but the selection was limited by data availability. Data measuring the quality of care of health services were not available, and the coverage of interventions such as the frequency of antenatal visits, was used as a proxy.

Second, stillbirth data are often not standardised and definitional adjustments were necessary, increasing the uncertainty in the estimates. For HICs, corresponding data from administrative sources in these settings were used to estimate the definitional adjustments and, for LMICs, high quality study data were used. The definitional adjustments did not take the magnitude of the stillbirth rate into account and this might have affected estimated adjustments because of missed variations. Even with this approach, 9·6% of the stillbirth data in the database could not be used because a clear definition was lacking or the available definition was not widely used elsewhere and no adjustment factor could be calculated. Consistent with International Classification of Diseases recommendations, we only estimated late gestation stillbirths at 28 weeks or more. This definition underestimates the true burden of all stillbirths because it excludes stillbirths occurring at earlier gestational ages. Studies in HICs indicate that about a third of stillbirths occur at 22–27 weeks of gestation,^13^ which was reflected in our definitional adjustments, whereby in high-income settings the stillbirth rate at 22 weeks or more of gestation was 1·5 times the stillbirth rate for stillbirths at 28 weeks or more of gestation. In low-income and middle-income settings the ratio of the 22-week stillbirth rate to the 28-week stillbirth rate was 1·2 (appendix p 9).^9^

Third, our assessment of data quality and source type biases has limitations as the available literature and our understanding of biases in stillbirth data remain poor. We aimed to exclude data of very low quality with a new statistical approach, and estimated source type biases and variance in definitions and data source types. However, further in-depth studies analysing potential reporting biases for stillbirths, early neonatal deaths, and livebirths in surveys, surveillance data, and vital registration data are needed to improve the understanding of these biases and inform future modelling work. Reported stillbirths across all data sources might be influenced by misclassification errors particularly with regards to early neonatal deaths. A few studies analysing survey and surveillance data have shown that the misclassification can occur in both directions, but further research is needed to improve understanding of these biases and develop further data quality assessment criteria.^33^, ^34^, ^35^, ^36^

Lastly, due to limited data availability and poor data quality, we were not always able to distinguish true regional and country effects from data quality issues in empirical observations and covariates. In the absence of data in many LMICs, estimates were driven by covariates and might not fully represent trends within each country. The covariates, or predictors, used in these models also have data limitations. For example, variables on health services, such as antenatal care visits or caesarean section (and variables these relate to, such as skilled birth attendance), while representing coverage of interventions, do not account for all variations in the quality of care that affect stillbirth rates. The estimated ratios of stillbirth rate to neonatal mortality rate in several countries with limited data were lower than ratios derived from high quality data in other countries.^16^, ^17^ Previous studies have reported stillbirth rate to neonatal mortality rate ratios of greater than 1 across varying mortality contexts, with ratios tending to increase with decreasing stillbirth and neonatal mortality rates.^8^, ^37^, ^38^, ^39^

Globally, our estimates are between the previous estimates published by WHO in 2016^7^ and the Global Burden of Disease Study (GBD) in 2017.^40^ WHO estimated 18·4 stillbirths (95% UI 16·6–21·0) per 1000 total births and 2·6 million stillbirths (95% UI 2·4–3·0) worldwide in 2015. Our global stillbirth rate estimate for 2015 was 15·0 stillbirths (90% UI 14·6–16·3) per 1000 total births, with an estimated 2·1 million (90% UI 2·1–2·3) stillbirths. The GBD estimated a global stillbirth rate of 13·1 stillbirths (95% UI 12·5–13·9) per 1000 total births and 1·7 million stillbirths (95% UI 1·6–1·8) in 2016; our stillbirth rate estimate for the same year was 14·7 stillbirths (14·3–16·0) per 1000 total births and 2·1 million stillbirths (2·0–2·3). At the country level, our stillbirth estimates were similar to the modelled stillbirth estimates produced by WHO in 2016^7^ for most countries; they were slightly lower at the global level. Between this study and the WHO analysis, the correlation coefficient of the two sets of country-specific point estimates for 186 countries, with estimates for the years 2000 and 2015, was 0·94. Differences between the two sets of estimates were the result of differences in input data, processing of input data, and modelling approaches. The correlation coefficient of our country-specific point estimates with the GBD point estimates for the year 2016 was 0·87 for 187 countries with estimates available in both sets.

Persistent gaps in stillbirth data, especially in countries and regions with the highest rates and numbers of stillbirths, based on our estimates, can mask the huge burden of stillbirths (panel 3). This invisibility can result in reduced attention, despite impacts on families and particularly women's mental health and wellbeing. Although there were limitations and uncertainty in the estimates, our findings indicate a large burden of stillbirths, with around 2 million stillbirths occurring annually, and slow progress in reducing the rate of stillbirths in the past two decades especially in high-burden countries. The real burden, including stillbirths from 22 weeks’ gestation, is even higher considering that we only considered late stillbirths. Acceleration is needed in increasing the coverage of interventions via high quality antenatal and intrapartum care to achieve the ENAP target of ending preventable stillbirths, and closing equity gaps. The ongoing COVID-19 pandemic poses increasing new challenges to health-care provision and access to service, requiring additional efforts to reduce stillbirths and accelerate progress. Improved data collection systems and timely and quality data will also help to understand the effect of COVID-19 on stillbirths across the globe.

Panel 3 **Data availability and data quality**

A crucial first step to preventing stillbirths is understanding the burden by accurately measuring stillbirth rates. This measurement is challenging due to poor availability of quality stillbirth data. In this analysis, for 62 countries, accounting for 29% of all stillbirths in 2019, no high quality empirical stillbirth data were available and the stillbirth estimates of the UN Inter-agency Group for Child Mortality Estimation were based on a covariate-based model.

To accelerate progress in reducing stillbirths, a focus on reducing data gaps in stillbirth rate data is needed, especially for sub-Saharan Africa and south Asia. Use of comparable definitions for stillbirths across all countries, strengthening the quality of stillbirth data collection in health management information systems (HMIS), linking vital registration systems with HMIS,^41^ and including pregnancy histories in all household surveys will all be important for closing these gaps.

Across all regions, detailed data are needed to better understand when and why stillbirths happen, and to enable improved targeting of interventions to prevent stillbirths. Gaps in data on the timing of stillbirths during pregnancy are large, with high quality information available for only 38 countries.^6^ Comparable data on causes of stillbirth in different settings are scarce. Across all country-income groups, regardless of the classification system used, the most frequently reported cause is commonly “unexplained”.^42^, ^43^, ^44^ In low-income countries, infection and complications during labour and birth remain important causes of stillbirth, while in middle-income and high-income countries stillbirths are more commonly attributed to placental complications; however, data quality for determining causes of stillbirth is generally poor.^42^, ^43^ Improved recording of the timing of stillbirth and causes with a harmonised classification system is urgently needed. In 2016, WHO published the International Classification of Diseases-Perinatal Mortality (ICD-PM) for the application of the ICD 10th revision to deaths during the perinatal period, which is an important step for facilitating comparable reporting of the causes of stillbirths and early neonatal deaths across different settings.^45^ ICD-PM is now being used in a number of settings.^46^, ^47^, ^48^, ^49^

## Data sharing

All input data from administrative registration systems, HMIS, household surveys, and population-based studies are available in the public domain with final estimates. The Bayesian hierarchical temporal sparse regression model codes are available on request to the corresponding author.

## Declaration of interests

We declare no competing interests.
