# Supplementary material for: Global, regional, and national estimates and trends in stillbirths from 2000 to 2019: a systematic assessment
Source: Lancet. 2021 Aug 28;398(10302):772–85. doi: 10.1016/S0140-6736(21)01112-0 (PMC8417352; doi:10.1016/S0140-6736(21)01112-0)
Supplement: Supplementary appendix [file mmc1.pdf]

THE LANCET

Supplementary appendix

This appendix formed part of the original submission and has been peer reviewed. We post it as supplied by the authors.

Supplement to: Hug L, You D, Blencowe H, et al. Global, regional, and national estimates and trends in stillbirths from 2000 to 2019: a systematic assessment. *Lancet* 2021; **398:** 772–85.

Web appendix

Title: Global, regional, and national levels and trends in stillbirths from 2000 to 2019: a systematic assessment.

Authors: Lucia Hug, Danzhen You, Hannah Blencowe, Anu Mishra, Zhengfan Wang, Miranda Fix, Jon Wakefield, Allisyn Moran, Victor Gaigbe-Togbe, Emi Suzuki, Dianna M. Blau, Simon Cousens, Andreea Creanga, Trevor Croft, Kenneth Hill, K. S. Joseph, Salome Maswime, Elizabeth McClure, Robert Pattinson, Jon Pedersen, Lucy K. Smith, Jennifer Zeitlin and Leontine Alkema on behalf of the United Nations Inter-agency Group for Child Mortality Estimation and its Core Stillbirth Estimation Group.

Contents

1. Abbreviations 2
2. Methods 3
2.1. Overview 3
2.3 Assumptions for definitional adjustment of stillbirth data 6
2.4 Stillbirth estimation model 6
2.5 Calculation of stillbirths 6
3. Regional classification 7
4. Supplementary Tables and Figures Table A1: Data availability by region and inclusion 9
Table A2: Data availability and inclusion by data source type 9
Table A3: Definitional adjustment factors 9
Table A4: Source type biases estimates and assumptions 9
Table A5: Covariates used in stillbirth estimation model and corresponding estimated coefficients 10
Table A6: Rates and number of stillbirths, by country or territory 10
Table A7: Share of stillbirths, percentage decrease, annual rate of change in stillbirth rate, neonatal mortality rate and mortality rate among children aged 1–59 months, and ratio of stillbirth to neonatal mortality rate, by country or territory 18
Table A8: Series in SBR database by country or territory 27
Table A9: Data availability and inclusion by country and data source type 37
Figure A4: Ratio of stillbirth rate and neonatal mortality rate and stillbirth rate in countries, 2019 41
Figure A5: Ratio of stillbirth rate and neonatal mortality rate and stillbirth rate in countries, 2000-2019 42
Figure A6: Stillbirth rate estimates with underlying data, by country or territory 43
References 42

1. Abbreviations

DHS	Demographic and Health Survey
HMIS	Health Management Information Systems
MICS	Multiple Indicator Cluster Survey
NMR	Neonatal Mortality Tate
PH	Pregnancy History
RC	Reproductive Calendar
SBR	Stillbirth Rate
UN IGME	United Nations Inter-agency Group for Child Mortality Estimation
VR	Vital Registration

2. Methods

2.1. Overview

The UN IGME's approach to estimate stillbirth rates (SBR) includes the following steps:

1. Compile all available stillbirth data at a country level, derived from administrative sources, household surveys or population-based studies.

2. Evaluate data in accordance with the data quality criteria and produce adjustment or recalculation by applying standardized definitions.

3. Estimate global and country-specific trends of stillbirth rates using a smoothing time series model, supplemented with covariates associated with stillbirth rates. This process averages empirical data on stillbirths derived from the different sources for a given country. In the case of countries with sparse or no data, the identified covariates associated with stillbirth will inform the trend in stillbirth rate.

To increase the transparency of the estimation methodology and make stillbirth data available to users worldwide, UN IGME makes all data sources and stillbirth estimates available on its web portal at www.childmortality.org. 2.2 Data sources

Figure A1: Total stillbirth data and final data used in model by source type

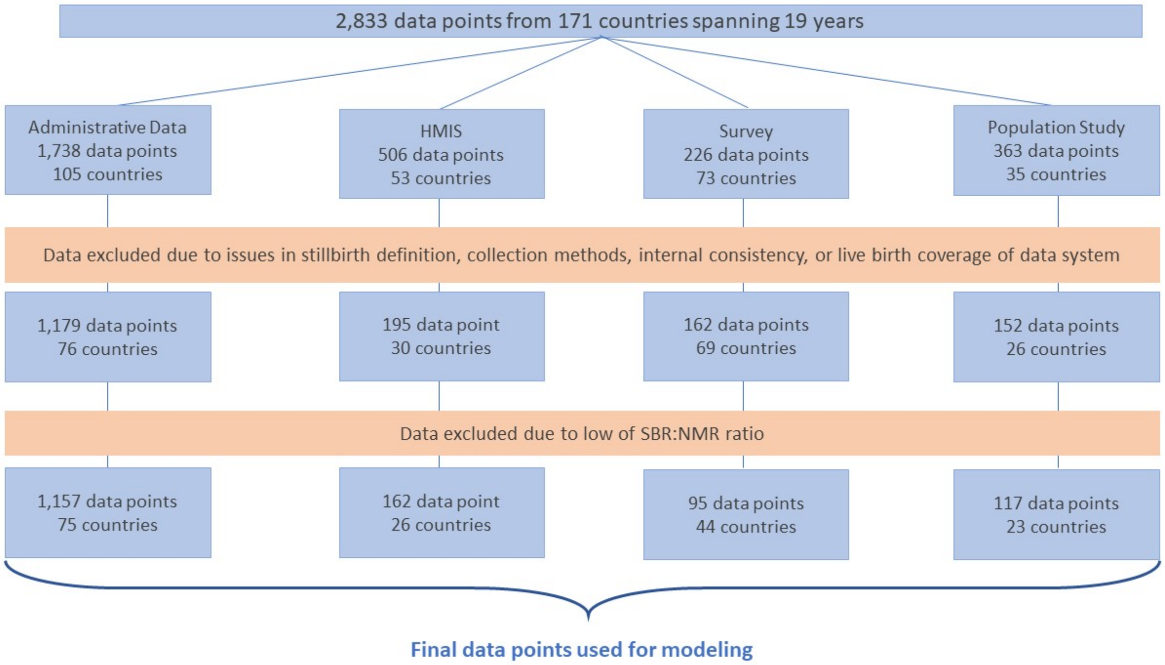

Estimates of stillbirth rates for a country are derived from various sources, such as administrative data (e.g. vital registration systems, birth or death registries, or health management information systems),

household surveys, or from population-based studies obtained from a review of academic literature. Additionally, data on factors associated with stillbirth rate are collected, to be used as covariates in the stillbirth estimation model. Table A5 lists the covariates used in the stillbirth estimation model and data sources.

Figure A2: Data availability by source type and inclusion by region

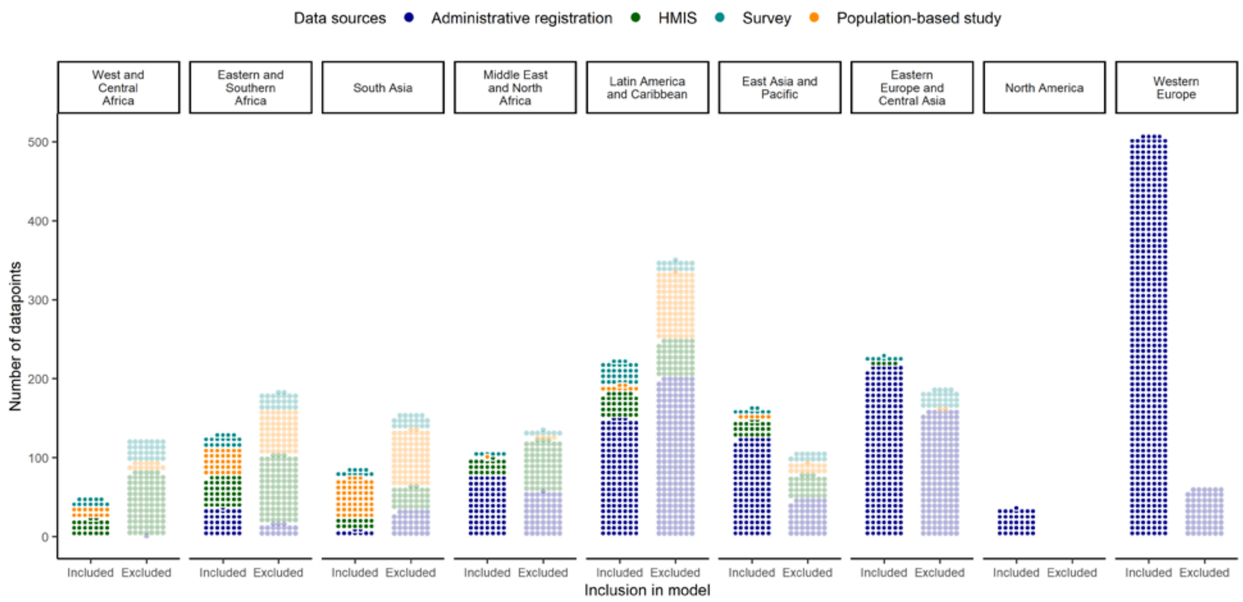

Figure A3: Included data availability by source type and country

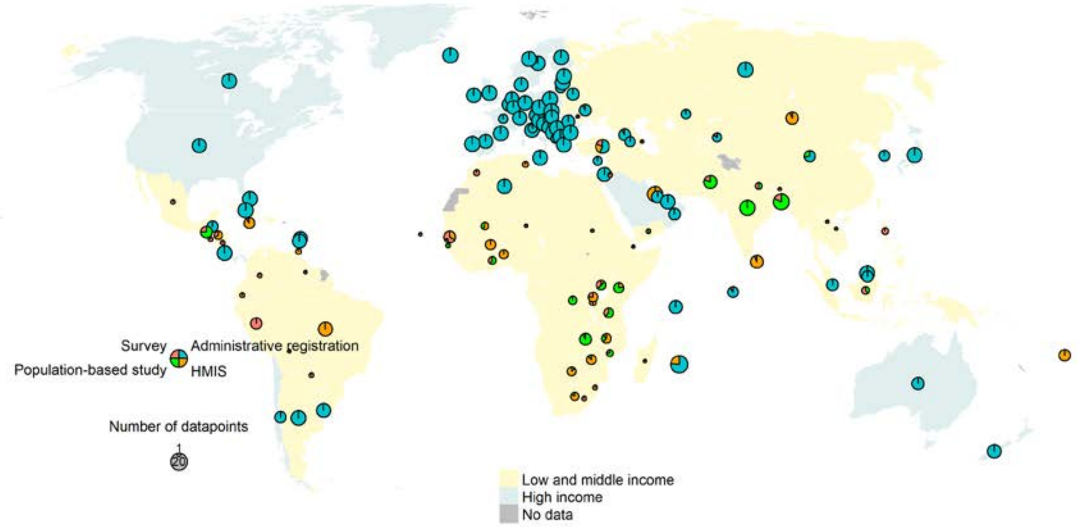

Note: This map does not reflect a position by UN IGME agencies or those of the institutions to which the authors are affiliated on the legal status of any country or territory or the delimitation of any frontiers.

The majority of administrative data comes from registration systems and health data systems including health management information systems (HMIS). Often data from registration systems record stillbirths

and live births using detailed gestational age and/or birthweight. HMIS data are collected in health facilities and in many countries, the District Health Information System-2 (DHIS2) is the commonest HMIS data platform.[1] Few HMIS systems currently report detailed gestational age and/or birthweight data on stillbirths.

Information on stillbirths in household surveys -such as such as the United States Agency for International Development (USAID)-supported Demographic and Health Surveys, Reproductive Health Survey (RHS) and the UNICEF-supported Multiple Indicator Cluster Surveys- are usually collected in two different ways: with a full pregnancy history; or with a reproductive calendar.[2] In the pregnancy history (PH), women of reproductive age are asked about all pregnancies in their lifetime. For each pregnancy they are asked to provide information on the duration of the pregnancy, the outcome of the pregnancy (e.g., miscarriage, stillbirth or livebirth) and the date of birth or end of pregnancy. In some surveys with PH modules the women were only asked whether they had a stillbirth and the date of the stillbirth. In these cases, a seven-month duration of pregnancy was assumed. In some survey-specific cases, a stillbirth was defined by the questionnaire as a fetal death occurring at the fifth or sixth month or later. PH data allow the calculation of stillbirth rates for specific time periods in the past. PH data allow to calculate stillbirth rates for specific time periods. For PH data, the stillbirth estimates were calculated for 5-year calendar year periods instead of the 5-year periods (as measured by up to 4 years prior to survey administration) preceding the survey. We calculated stillbirth rates for 5 intervals (e.g. 25 years), before the survey date. The most recent 5-year calendar period was included in the estimation model.

In the reproductive calendar (RC), women are asked about the duration and month of pregnancy end for pregnancies that did not end in a live birth in the last 60 months. RCs are usually administered alongside a full birth history. In reproductive calendars the stillbirth rate is the number of pregnancies that are terminated in the seventh month or later of pregnancy divided by the number of pregnancies that reached at least the seventh month. The RC data allow the calculation of stillbirth rates for the 5-year period preceding the survey. However, stillbirth estimates from the RC were not included in the model if estimates from the PH in the same survey were available.

Population-based study data were sought for all countries without high coverage of routine administrative data. The literature review undertaken for the previous stillbirth estimates[3] was updated through to 29 January 2019. In addition, further reanalyzed population-based stillbirth data were obtained from a WHO data call to maternal-newborn health experts.

Data were excluded if: they lacked a clear source of definition or clear information on data collection systems; a high proportion of reported stillbirths had unknown gestational age or birthweight; data were internally inconsistent; or coverage of live births in administrative data systems was estimated below 80 per cent. Vital registration data with incomplete coverage of child deaths were also excluded. Consistency across data sources was further assessed by comparing stillbirth estimates to similar data sources within the same country and expected global and regional patterns in mortality.

As part of the assessment of data quality, the plausibility of the ratio of stillbirth rates (measured as per 28 weeks of gestation or more definition) to neonatal mortality rates was assessed, by comparing these ratios to the distribution of ratios obtained from high-quality LMIC study data. High-quality LMIC study data is defined as population-based prospectively collected data with recruitment prior to 28 weeks of gestation, and follow-up to at least 28 days of age of live births.

In assessing the SBR:NMR ratio in the input database, the NMR from the data source was used where available. Where data sources had missing NMR data, the estimated NMR by UN IGME was used. For observations from HMIS and population studies on stillbirths, the ratio of observed SBR to the UN IGME NMR was calculated and the same exclusion approach applied so that observations with extremely low SBR compared to national level NMR were excluded. In summary, the mean and variance of the setting-specific SBR:NMR ratios is estimated, assuming that each observed SBR:NMR ratio is the sum of a setting-specific SBR:NMR ratio and random stochastic error. If stillbirths were under-reported relative to neonatal deaths for a specific observation, its associated observed ratio of SBR to NMR would be lower than the true ratio. To quantify whether an observed ratio is 'extremely' low, the probability of observing a ratio that is smaller than the observed ratio was calculated (taking account of the uncertainty associated with the observed ratio) using the distribution of ratios obtained from the high-quality data. If this probability was less than 0.05, the observation was excluded from the database. This approach was applied to all observations in the database with 28 weeks of gestation or more definitions and adjusted 28-week definitions.

2.3 Assumptions for definitional adjustment of stillbirth data

Data limitations necessitated some assumptions regarding definitional adjustments. For survey data, a seven-month duration of pregnancy is assumed to be equal to a 28-weeks or more definition. Further, in LMICs it is assumed that the SBR observed using a stillbirth definition of a birthweight of 1,000 grams or more is equal to the SBR observed using the 28 weeks of gestation or more definition, and similarly that the SBR observed with a birthweight of 500 grams or more definition equals the SBR observed with a 22 week of gestational age or more definition.

2.4 Stillbirth estimation model

Estimation and projection of stillbirth rates is undertaken using a statistical model for all country-years. In the model, the SBR is estimated assuming that the

Observed log (SBR) = log (true SBR) + bias + measurement error

where the true SBR in a country for years 2000 to 2020 = country-intercept + SBR predicted by covariates + country-specific temporal smoothing process. The bias refers to the definitional adjustment bias and source type bias. The measurement error refers to the stochastic sampling error, source type error, and additional error incurred from definitional adjustments. The model produces estimates of the SBR for years 2000 to 2020 with uncertainty.

Full details on the estimation model and statistical methods are described in Wang et al [4].

2.5 Calculation of stillbirths

The number of stillbirths in each country is calculated using the following formula: Number of stillbirths=livebirths * SBR/(1-SBR). The annual estimate of the number of live births in each country from the World Population Prospects: the 2019 revision[5] are used along with the UN IGME SBR estimates to calculate the estimated numbers of stillbirths.

3. Regional classification

The Regional classifications refer to the UNICEF's regional classification. The regions of Western Europe and Eastern Europe and Central Asia are a sub-regions of Europe and Central Asia and Eastern and Southern Africa and Western and Central Africa are the sub-regions of Sub-Saharan Africa. For further details please refer to http://data.unicef.org/regionalclassifications/.

In addition, the World Bank Group income classification from the year 2020 was used. For further details please refer to <https://datahelpdesk.worldbank.org/knowledgebase/articles/906519> accessed August 3, 2020.

East Asia and Pacific

Australia; Brunei Darussalam; Cambodia; China; Cook Islands; Democratic People's Republic of Korea; Fiji; Indonesia; Japan; Kiribati; Lao People's Democratic Republic; Malaysia; Marshall Islands; Micronesia (Federated States of); Mongolia; Myanmar; Nauru; New Zealand; Niue; Palau; Papua New Guinea; Philippines; Republic of Korea; Samoa; Singapore; Solomon Islands; Thailand; Timor-Leste; Tonga; Tuvalu; Vanuatu; Viet Nam

Europe and Central Asia

Eastern Europe and Central Asia; Western Europe

Eastern Europe and Central Asia

Albania; Armenia; Azerbaijan; Belarus; Bosnia and Herzegovina; Bulgaria; Croatia; Georgia; Kazakhstan; Kyrgyzstan; Montenegro; Republic of Moldova; Northern Macedonia; Romania; Russian Federation; Serbia; Tajikistan; Turkey; Turkmenistan; Ukraine; Uzbekistan

Western Europe

Andorra; Austria; Belgium; Cyprus; Czechia; Denmark; Estonia; Finland; France; Germany; Greece; Hungary; Iceland; Ireland; Italy; Latvia; Lithuania; Luxembourg; Malta; Monaco; Netherlands; Norway; Poland; Portugal; San Marino; Slovakia; Slovenia; Spain; Sweden; Switzerland; United Kingdom

Latin America and Caribbean

Antigua and Barbuda; Argentina; Bahamas; Barbados; Belize; Bolivia (Plurinational State of); Brazil; Chile; Colombia; Costa Rica; Cuba; Dominica; Dominican Republic; Ecuador; El Salvador; Grenada; Guatemala; Guyana; Haiti; Honduras; Jamaica; Mexico; Nicaragua; Panama; Paraguay; Peru; Saint Kitts and Nevis; Saint Lucia; Saint Vincent and the Grenadines; Suriname; Trinidad and Tobago; Uruguay; Venezuela (Bolivarian Republic of)

Middle East and North Africa

Algeria; Bahrain; Egypt; Iran (Islamic Republic of); Iraq; Israel; Jordan; Kuwait; Lebanon; Libya; Morocco; Oman; Qatar; Saudi Arabia; State of Palestine; Syrian Arab Republic; Tunisia; United Arab Emirates; Yemen

North America

Canada; United States of America

South Asia

Afghanistan; Bangladesh; Bhutan; India; Maldives; Nepal; Pakistan; Sri Lanka

Sub-Saharan Africa

Eastern and Southern Africa; West and Central Africa

Eastern and Southern Africa

Angola; Botswana; Burundi; Comoros; Djibouti; Eritrea; Eswatini; Ethiopia; Kenya; Lesotho; Madagascar; Malawi; Mauritius; Mozambique; Namibia; Rwanda; Seychelles; Somalia; South Africa; South Sudan; Sudan; Uganda; United Republic of Tanzania; Zambia; Zimbabwe

West and Central Africa

Benin; Burkina Faso; Cabo Verde; Cameroon; Central African Republic; Chad; Congo; Côte d'Ivoire; Democratic Republic of the Congo; Equatorial Guinea; Gabon; Gambia; Ghana; Guinea; Guinea-Bissau; Liberia; Mali; Mauritania; Niger; Nigeria; Sao Tome and Principe; Senegal; Sierra Leone; Togo

Map A1: UNICEF regional classifications.

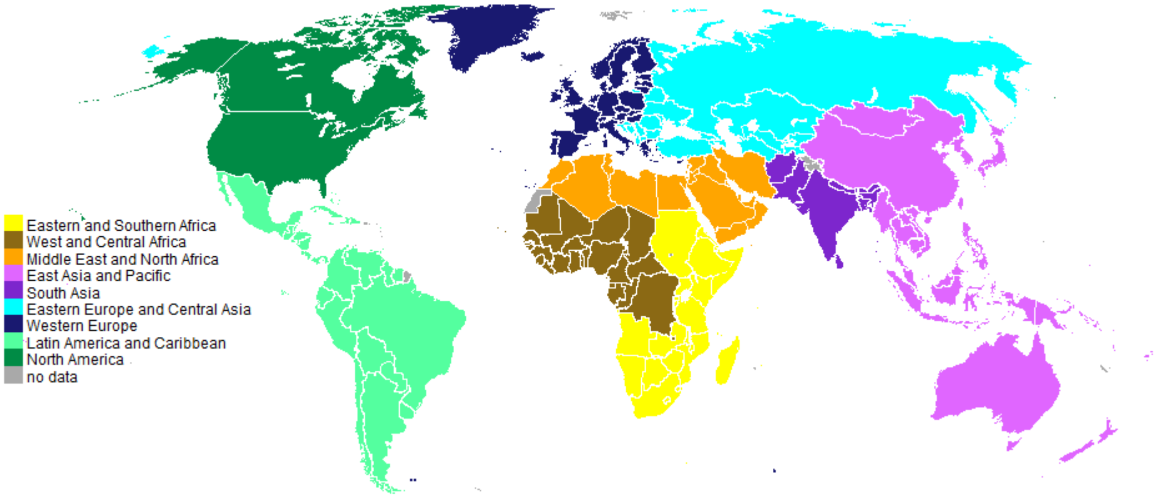

Note: This map does not reflect a position by UN IGME agencies or those of the institutions to which the authors are affiliated on the legal status of any country or territory or the delimitation of any frontiers.

4. Supplementary Tables and Figures

Table A1: Data availability by region and inclusion

Region	Included datapoints	Available datapoints
East Asia and Pacific	154	256
Eastern and Southern Africa	124	281
Eastern Europe and Central Asia	218	399
Latin America and Caribbean	213	463
Middle East and North Africa	106	237
North America	36	36
South Asia	87	188
West and Central Africa	48	165
Western Europe	499	561
**Total**	**1,485**	**2,586**

Table A2: Data availability and inclusion by data source type

Source	Number of country-years available	Number included quality country-years
Administrative registration system	1,738	1,157
Health Management Information System (HMIS)	506	162
Household survey	226	95
Population based studies	363	117

Table A3: Definitional adjustment factors

Definition of stillbirths	LMIC	Bias on log-scale: log(SBR[alternative]) – log(SBR[28])	Standard deviation log-scale
22 or more weeks of gestation	Yes	0.2	0.1
22 weeks or more weeks of gestation	No	0.4	0.2
24 weeks or more weeks of gestation	No	0.2	0.2
Birth weight of 1000 grams or more	No	-0.1	0.1
Birth weight 500 grams or more	No	0.2	0.1

Table A4: Source type biases estimates and assumptions

Source	Bias log-scale, RR	Source type standard deviation	Assumption
Administrative registration systems	0	0.02	0 bias. Estimated standard deviation
Health Management Information System (HMIS)	0	0.05	0 bias. Estimated standard deviation
Population based studies	0	0.24	0 bias. Estimated standard deviation
Household survey	-0.2; 0.85	0.14	N(0,25)T[,0] bias. Estimated standard deviation

Table A5: Covariates used in stillbirth estimation model and corresponding estimated coefficients

Covariate	Data Source	Estimate
GNI per capita based on purchasing power parity (PPP). PPP GNI is gross national income (GNI) converted to international dollars using purchasing power parity rates	World Bank: World Development Indicators database, World Bank	-0.116 (-0.19, -0.04)
Neonatal mortality rate: Probability of dying in the first 28 days of life, expressed per 1,000 live births	UN IGME: Modelled based on data from vital registration, household survey and population census	0.401 (0.32, 0.48)
Low birthweight: Percentage of live births that weighed less than 2,500 grams (less than 5.51 pounds)	UNICEF/WHO Low birthweight (LBW) estimates, 2019 Edition	0.097 (0.04, 0.15)
Antenatal care 4+ visits: Percentage of women (aged 15–49 years) attended at least four times during pregnancy by any provider	WHO/UNICEF: DHS, MICS and other national household surveys	-0.052 (-0.11, 0.01)
Mean years of schooling (female): Average number of years of education received by females aged 25 years and older, converted from educational attainment levels using official durations of each level	UNDP: Estimated by Barro and Lee (2016) using population census, MICS, DHS and other national surveys	-0.062 (-0.12, -0.01)
C-section rate: Percentage of deliveries by C-section	UNICEF: DHS, MICS, RHS and other national household surveys	-0.047 (-0.09, 0)

Note: Numbers in brackets are 95 per cent uncertainty intervals.

Table A6: Rates and number of stillbirths, by country or territory

Note: Numbers in brackets are 90 per cent uncertainty intervals.

Country/territory	Stillbirth rate (stillbirths per 1,000 total births)			Number of stillbirths		
	2000	2010	2019	2000	2010	2019
Afghanistan	36.9	32.6	28.4	39296	39734	35384
	(22.1, 63.4)	(20.6, 53.2)	(16.8, 47.3)	(25167, 63161)	(26424, 60832)	(22732, 55529)
Albania	6.4	4.5	4.1	329	161	137
	(4.6, 8.9)	(4.0, 5.2)	(3.3, 5.0)	(249, 430)	(144, 180)	(114, 164)
Algeria	17.2	12.1	9.5	10605	10796	9674
	(14.7, 20.0)	(10.7, 13.5)	(7.7, 11.6)	(9293, 12091)	(9793, 11884)	(8102, 11477)
Andorra	3.5	2.6	2.1	3	2	1
	(2.5, 5.1)	(1.9, 3.5)	(1.4, 3.0)	(2, 3)	(2, 3)	(1, 2)
Angola	30.0	23.4	19.8	24539	25603	25967
	(18.3, 50.0)	(14.8, 37.0)	(12.2, 32.7)	(16046, 38491)	(17303, 38545)	(17234, 40126)
Antigua and Barbuda	8.8	6.8	5.5	13	10	8
	(5.4, 14.7)	(4.2, 10.8)	(3.2, 9.0)	(9, 20)	(7, 15)	(5, 12)
Argentina	7.8	4.9	5.3	5669	3624	4042
	(7.5, 8.1)	(4.7, 5.0)	(4.9, 5.8)	(5489, 5852)	(3519, 3743)	(3751, 4353)
Armenia	20.7	15.1	12.9	835	681	526
	(14.9, 28.4)	(13.5, 16.9)	(11.1, 14.8)	(632, 1108)	(619, 749)	(466, 594)
Australia	3.2	2.9	2.2	794	868	707
	(3.0, 3.4)	(2.7, 3.0)	(1.9, 2.6)	(754, 836)	(828, 909)	(613, 819)
Austria	2.8	2.5	2.2	226	194	195
	(2.6, 3.1)	(2.3, 2.7)	(1.9, 2.5)	(209, 245)	(182, 206)	(176, 218)
Azerbaijan	19.2	12.6	9.0	2753	2093	1466

	(13.4, 27.6)	(8.9, 18.4)	(5.9, 14.0)	(2031, 3749)	(1540, 2845)	(1020, 2130)
Bahamas	12.5	13.3	11.6	66	71	63
	(10.8, 14.4)	(12.0, 14.6)	(9.9, 13.7)	(59, 75)	(65, 77)	(55, 73)
Bahrain	8.5	6.2	5.9	128	123	131
	(7.0, 10.3)	(5.6, 6.8)	(5.0, 7.0)	(108, 150)	(114, 133)	(112, 152)
Bangladesh	41.4	29.7	24.3	151590	95370	72508
	(34.1, 50.3)	(26.0, 34.0)	(19.9, 29.6)	(128018, 180265)	(85134, 107036)	(60932, 86073)
Barbados	8.4	8.4	7.4	32	28	23
	(5.0, 14.0)	(5.3, 13.1)	(4.5, 12.3)	(21, 49)	(19, 41)	(15, 35)
Belarus	5.0	2.5	2.0	443	273	222
	(3.8, 6.4)	(2.3, 2.6)	(1.8, 2.3)	(359, 545)	(257, 288)	(199, 247)
Belgium	3.4	3.1	2.8	388	393	354
	(3.1, 3.7)	(2.9, 3.3)	(2.4, 3.4)	(363, 413)	(373, 413)	(303, 411)
Belize	11.2	8.7	6.5	83	67	53
	(8.4, 15.1)	(7.6, 10.1)	(5.5, 7.8)	(65, 106)	(59, 75)	(46, 61)
Benin	27.2	24.6	20.3	8156	9141	8795
	(20.2, 37.1)	(22.9, 26.4)	(17.1, 24.3)	(6355, 10623)	(8603, 9699)	(7578, 10257)
Bhutan	16.3	12.2	9.7	265	165	127
	(11.0, 24.3)	(9.4, 15.7)	(7.5, 12.6)	(191, 369)	(131, 205)	(101, 158)
Bolivia (Plurinational State of)	15.5	12.0	8.9	4033	3048	2219
	(10.2, 23.7)	(8.6, 16.5)	(6.1, 12.9)	(2801, 5750)	(2305, 4029)	(1604, 3039)
Bosnia and Herzegovina	4.0	3.2	2.8	164	110	73
	(3.4, 4.7)	(2.9, 3.6)	(2.2, 3.4)	(142, 188)	(101, 120)	(61, 87)
Botswana	11.8	15.5	15.2	574	869	862
	(10.8, 12.9)	(14.4, 16.7)	(12.8, 18.1)	(531, 623)	(813, 929)	(744, 998)
Brazil	10.0	8.5	7.5	35340	25632	21771
	(8.8, 11.2)	(7.8, 9.3)	(6.1, 9.2)	(31945, 39093)	(23843, 27654)	(18361, 25773)
Brunei Darussalam	5.1	4.3	4.6	39	29	29
	(4.2, 6.1)	(3.7, 5.0)	(3.7, 5.7)	(33, 45)	(26, 33)	(24, 35)
Bulgaria	7.6	6.8	5.0	501	503	312
	(7.1, 8.2)	(6.4, 7.2)	(4.6, 5.5)	(472, 533)	(479, 527)	(291, 335)
Burkina Faso	29.7	23.7	19.5	16462	16031	15141
	(21.2, 41.6)	(21.3, 26.4)	(17.7, 21.4)	(12466, 21936)	(14646, 17627)	(13953, 16440)
Burundi	30.4	26.7	26.1	8736	10212	11880
	(21.7, 42.9)	(21.7, 32.5)	(24.2, 28.0)	(6562, 11671)	(8579, 12135)	(11146, 12607)
Cabo Verde	15.8	14.3	10.9	189	160	116
	(9.9, 24.9)	(9.2, 22.4)	(6.8, 17.8)	(128, 283)	(111, 233)	(78, 176)
Cambodia	25.5	16.5	12.4	8905	6146	4573
	(14.9, 43.7)	(10.3, 26.6)	(7.6, 20.9)	(5651, 14137)	(4095, 9256)	(3008, 7082)
Cameroon	23.9	21.7	19.4	15798	17831	17872
	(14.6, 39.2)	(13.6, 34.7)	(11.5, 31.7)	(10312, 24582)	(12059, 26556)	(11650, 27137)
Canada	3.2	2.9	2.8	1075	1114	1072
	(3.0, 3.4)	(2.8, 3.1)	(2.5, 3.1)	(1025, 1126)	(1071, 1157)	(987, 1164)
Central African Republic	34.0	32.5	29.8	5298	5671	5147
	(20.5, 56.2)	(20.8, 51.2)	(18.5, 48.4)	(3448, 8211)	(3836, 8372)	(3410, 7845)
Chad	34.7	30.1	27.5	15404	17361	18802
	(20.5, 57.8)	(18.7, 48.9)	(16.7, 45.5)	(9868, 24233)	(11722, 26067)	(12297, 28597)
Chile	4.2	3.7	3.1	1062	922	711
	(3.3, 5.3)	(3.5, 3.9)	(2.6, 3.7)	(866, 1302)	(885, 960)	(617, 821)
China	15.0	10.2	5.5	268416	179677	92170
	(13.2, 17.1)	(9.3, 11.1)	(4.9, 6.3)	(240484, 298695)	(165959, 194298)	(82934, 102854)
Colombia	9.6	8.4	7.1	8517	6455	5237

	(7.1, 12.9)	(6.6, 10.8)	(5.2, 9.9)	(6672, 10888)	(5224, 7989)	(4016, 6963)
Comoros	30.3	28.0	24.6	636	702	674
	(18.2, 50.5)	(17.6, 44.5)	(14.9, 39.8)	(412, 1006)	(472, 1065)	(440, 1032)
Congo	20.6	17.1	15.0	2535	2748	2664
	(12.3, 33.5)	(10.6, 27.3)	(9.2, 24.5)	(1650, 3839)	(1844, 4070)	(1746, 4070)
Cook Islands	9.6	6.9	5.2	4	2	1
	(6.9, 13.3)	(5.0, 9.3)	(3.5, 7.5)	(3, 6)	(2, 3)	(1, 2)
Costa Rica	4.9	5.1	4.5	373	367	311
	(4.1, 5.7)	(4.8, 5.4)	(4.0, 5.0)	(327, 425)	(348, 387)	(281, 344)
Côte d'Ivoire	30.2	26.8	23.2	20940	21259	21735
	(18.1, 50.6)	(16.9, 42.7)	(13.8, 38.3)	(13680, 32534)	(14323, 32010)	(14072, 33571)
Croatia	5.2	3.4	3.0	234	144	109
	(4.7, 5.7)	(3.1, 3.6)	(2.6, 3.5)	(215, 256)	(135, 154)	(96, 123)
Cuba	10.9	6.9	6.9	1605	868	784
	(10.4, 11.5)	(6.5, 7.2)	(6.5, 7.4)	(1538, 1675)	(833, 904)	(744, 827)
Cyprus	4.4	3.0	2.5	55	39	31
	(3.1, 6.2)	(2.5, 3.7)	(2.0, 3.2)	(41, 74)	(33, 46)	(26, 38)
Czechia	2.8	2.6	2.6	252	288	291
	(2.6, 3.1)	(2.4, 2.7)	(2.4, 2.9)	(234, 271)	(273, 305)	(271, 312)
Democratic People's Republic of Korea	13.7	10.9	8.5	5729	3785	3042
	(8.4, 22.7)	(6.9, 17.3)	(5.3, 13.8)	(3734, 8814)	(2567, 5608)	(2023, 4571)
Democratic Republic of the Congo	33.8	30.1	27.2	76296	90155	98871
	(22.9, 49.4)	(23.2, 38.6)	(21.7, 34.3)	(54639, 107020)	(72648, 112433)	(80995, 120773)
Denmark	3.1	2.1	2.0	207	131	126
	(2.8, 3.6)	(1.9, 2.3)	(1.6, 2.5)	(187, 229)	(122, 142)	(105, 149)
Djibouti	34.9	32.2	27.9	812	710	586
	(24.7, 49.6)	(27.1, 38.2)	(21.6, 36.5)	(604, 1100)	(614, 826)	(468, 742)
Dominica	11.5	12.6	13.7	14	11	14
	(6.7, 18.8)	(7.9, 19.6)	(8.3, 22.4)	(9, 21)	(8, 17)	(9, 21)
Dominican Republic	14.4	12.6	10.7	3062	2638	2224
	(8.7, 23.6)	(8.0, 20.0)	(6.4, 17.9)	(1984, 4722)	(1762, 3936)	(1426, 3447)
Ecuador	15.7	11.1	8.7	5159	3615	2966
	(11.9, 20.4)	(8.9, 13.7)	(6.2, 12.4)	(4088, 6450)	(3007, 4365)	(2209, 3994)
Egypt	17.5	12.3	9.0	31828	28380	23527
	(10.5, 29.1)	(7.8, 19.8)	(5.5, 15.6)	(20771, 49478)	(19363, 42642)	(15545, 36889)
El Salvador	20.1	12.6	10.1	3001	1551	1189
	(15.8, 25.2)	(9.5, 16.7)	(6.7, 14.9)	(2427, 3691)	(1219, 1963)	(848, 1643)
Equatorial Guinea	18.0	15.8	15.1	460	571	681
	(10.7, 30.4)	(9.7, 25.8)	(9.1, 25.3)	(297, 725)	(378, 869)	(441, 1058)
Eritrea	23.2	20.5	18.3	1835	2380	1945
	(14.3, 38.1)	(13.3, 32.6)	(11.1, 30.2)	(1200, 2831)	(1633, 3525)	(1285, 2936)
Estonia	4.7	3.0	2.2	60	46	29
	(4.1, 5.4)	(2.7, 3.4)	(1.8, 2.6)	(53, 67)	(42, 50)	(26, 34)
Eswatini	15.7	14.3	13.2	513	471	398
	(11.2, 21.9)	(10.6, 19.0)	(8.9, 19.4)	(388, 684)	(366, 601)	(287, 559)
Ethiopia	35.8	31.1	24.6	107622	101121	90323
	(21.7, 59.4)	(19.4, 49.5)	(15.1, 40.5)	(69249, 168719)	(67533, 152356)	(58886, 139027)
Fiji	10.2	10.0	8.6	206	191	161
	(8.0, 13.0)	(9.1, 10.9)	(7.0, 10.5)	(166, 252)	(176, 207)	(135, 192)
Finland	2.7	2.1	2.0	153	124	102
	(2.4, 3.0)	(1.9, 2.2)	(1.7, 2.4)	(140, 168)	(115, 133)	(90, 115)
France	5.0	4.5	4.3	3800	3564	3157

	(4.5, 5.6)	(4.0, 5.0)	(3.7, 5.1)	(3472, 4166)	(3235, 3952)	(2753, 3619)
Gabon	17.6	16.0	13.8	733	885	940
	(10.6, 29.7)	(10.0, 25.8)	(8.4, 22.5)	(473, 1159)	(591, 1316)	(612, 1431)
Gambia	27.1	24.5	21.9	1574	1848	2001
	(17.5, 41.7)	(17.2, 35.1)	(14.6, 33.2)	(1084, 2273)	(1372, 2508)	(1414, 2875)
Georgia	15.2	9.3	5.7	827	532	304
	(11.9, 19.4)	(8.4, 10.2)	(5.3, 6.2)	(677, 1022)	(489, 579)	(282, 326)
Germany	2.9	2.3	2.7	2209	1550	2137
	(2.7, 3.2)	(2.2, 2.4)	(2.3, 3.2)	(2027, 2400)	(1480, 1624)	(1861, 2465)
Ghana	28.7	25.1	21.7	20347	20687	19529
	(22.0, 37.6)	(20.8, 30.5)	(16.4, 28.3)	(16165, 25716)	(17447, 24481)	(15409, 24690)
Greece	4.6	3.3	3.1	491	345	244
	(4.2, 4.9)	(3.1, 3.5)	(2.8, 3.5)	(462, 522)	(328, 363)	(221, 270)
Grenada	10.2	9.9	9.8	20	19	18
	(6.2, 17.1)	(6.2, 15.6)	(6.0, 16.1)	(13, 30)	(13, 27)	(12, 27)
Guatemala	19.9	15.9	12.7	8391	6576	5498
	(15.8, 24.9)	(13.5, 18.7)	(10.2, 15.9)	(6888, 10224)	(5723, 7560)	(4576, 6618)
Guinea	30.9	26.8	25.2	11583	11147	11895
	(19.0, 51.3)	(16.9, 42.8)	(15.5, 40.8)	(7613, 18003)	(7514, 16659)	(7850, 18224)
Guinea-Bissau	44.8	38.2	32.2	2366	2370	2209
	(32.2, 64.4)	(28.3, 51.8)	(22.0, 47.0)	(1750, 3235)	(1819, 3092)	(1602, 3076)
Guyana	18.1	16.1	13.8	356	260	216
	(12.3, 26.3)	(11.4, 22.8)	(9.1, 21.2)	(257, 500)	(194, 352)	(151, 313)
Haiti	23.8	22.6	19.9	6562	6361	5470
	(14.6, 40.1)	(14.4, 35.5)	(12.1, 32.7)	(4284, 10226)	(4312, 9420)	(3595, 8293)
Honduras	13.9	10.1	8.5	3133	2112	1787
	(10.7, 18.1)	(8.9, 11.5)	(7.1, 10.1)	(2507, 3938)	(1892, 2363)	(1533, 2068)
Hungary	4.3	3.5	3.3	414	324	303
	(4.0, 4.6)	(3.3, 3.7)	(2.7, 4.0)	(389, 441)	(308, 342)	(254, 359)
Iceland	2.7	2.1	1.9	11	10	8
	(2.2, 3.4)	(1.7, 2.5)	(1.5, 2.4)	(9, 13)	(8, 11)	(6, 9)
India	29.6	20.2	13.9	852386	535683	340622
	(21.9, 40.2)	(17.3, 23.5)	(11.4, 17.0)	(675197, 1086996)	(487933, 629195)	(328322, 471827)
Indonesia	15.2	11.9	9.5	71240	60411	45857
	(11.8, 19.9)	(9.8, 14.4)	(6.9, 12.8)	(56944, 89594)	(51591, 71494)	(35460, 58987)
Iran (Islamic Republic of)	11.3	8.4	6.8	13282	11414	10367
	(6.6, 19.7)	(5.1, 14.1)	(3.9, 11.5)	(8452, 21123)	(7504, 17541)	(6676, 16275)
Iraq	16.4	14.4	11.7	14028	14767	13270
	(9.6, 27.6)	(9.0, 23.1)	(7.1, 19.4)	(9033, 22104)	(9832, 22195)	(8633, 20352)
Ireland	5.0	3.7	2.8	276	267	172
	(4.5, 5.4)	(3.5, 3.9)	(2.4, 3.3)	(256, 297)	(253, 282)	(149, 196)
Israel	4.1	2.7	2.8	522	430	472
	(3.7, 4.6)	(2.6, 2.9)	(2.3, 3.3)	(479, 572)	(409, 452)	(409, 548)
Italy	2.8	2.4	2.4	1507	1319	1070
	(2.4, 3.3)	(2.2, 2.5)	(2.1, 2.7)	(1315, 1732)	(1260, 1386)	(954, 1202)
Jamaica	18.9	15.6	12.7	1090	791	602
	(15.8, 22.7)	(13.5, 17.9)	(9.2, 17.6)	(930, 1273)	(702, 891)	(460, 784)
Japan	2.5	2.0	1.5	2967	2255	1407
	(2.4, 2.7)	(2.0, 2.1)	(1.4, 1.7)	(2863, 3079)	(2182, 2330)	(1297, 1525)
Jordan	12.2	10.0	8.8	2009	2117	1914
	(9.4, 15.9)	(7.5, 13.2)	(6.0, 13.0)	(1601, 2520)	(1674, 2691)	(1390, 2649)
Kazakhstan	11.2	7.4	5.4	2670	2762	2040
	(8.3, 14.9)	(6.9, 8.1)	(4.3, 6.7)	(2085, 3412)	(2581, 2949)	(1718, 2443)

Kenya	22.5	20.6	19.7	29465	31077	30030
	(15.7, 32.2)	(16.4, 25.5)	(18.3, 21.1)	(21873, 40409)	(25725, 37503)	(28181, 31807)
Kiribati	17.0	15.6	14.3	45	48	47
	(10.3, 28.0)	(9.7, 25.0)	(8.7, 24.1)	(29, 69)	(32, 72)	(31, 73)
Kuwait	7.1	6.4	5.8	316	379	325
	(4.2, 11.6)	(4.0, 10.1)	(3.6, 9.6)	(203, 492)	(253, 570)	(215, 500)
Kyrgyzstan	10.8	9.0	6.8	1151	1327	1051
	(8.0, 14.6)	(8.5, 9.5)	(6.2, 7.5)	(891, 1483)	(1269, 1388)	(965, 1144)
Lao People's Democratic Republic	24.3	20.9	16.6	4247	3586	2791
	(15.0, 39.0)	(13.6, 31.1)	(10.6, 25.4)	(2812, 6371)	(2496, 5049)	(1919, 4065)
Latvia	6.0	4.2	3.2	116	93	65
	(5.3, 6.7)	(3.8, 4.6)	(2.8, 3.8)	(105, 128)	(86, 101)	(57, 75)
Lebanon	10.8	7.6	6.3	915	664	745
	(6.4, 18.2)	(4.7, 12.1)	(3.8, 10.4)	(603, 1421)	(449, 987)	(485, 1147)
Lesotho	35.6	30.4	27.9	2292	1824	1611
	(26.7, 45.9)	(23.4, 38.9)	(19.6, 39.7)	(1796, 2875)	(1450, 2268)	(1184, 2178)
Liberia	29.8	25.4	24.2	3594	3739	4008
	(18.2, 49.3)	(16.1, 40.1)	(15.0, 39.6)	(2314, 5485)	(2537, 5554)	(2647, 6137)
Libya	13.8	10.4	8.8	1631	1417	1094
	(8.3, 23.6)	(6.6, 16.6)	(5.3, 14.6)	(1062, 2557)	(952, 2097)	(717, 1653)
Lithuania	4.5	3.4	2.8	152	109	78
	(3.3, 6.2)	(3.1, 3.8)	(2.4, 3.2)	(116, 197)	(99, 120)	(69, 89)
Luxembourg	3.6	3.5	3.4	20	20	22
	(3.0, 4.4)	(3.0, 4.0)	(2.6, 4.3)	(17, 23)	(18, 23)	(18, 27)
Madagascar	20.2	17.5	16.5	13453	13261	14671
	(14.4, 28.6)	(12.9, 23.7)	(11.1, 24.5)	(10015, 18119)	(10261, 17125)	(10469, 20545)
Malawi	22.2	20.0	16.3	11042	11947	10440
	(17.2, 29.0)	(17.0, 23.2)	(14.7, 18.1)	(8848, 13871)	(10447, 13711)	(9524, 11467)
Malaysia	4.9	4.5	5.5	2514	2184	2921
	(3.8, 6.4)	(4.3, 4.7)	(5.0, 6.0)	(1996, 3168)	(2114, 2258)	(2705, 3156)
Maldives	13.7	7.6	5.8	84	56	41
	(10.5, 18.0)	(6.7, 8.6)	(4.4, 7.5)	(67, 106)	(51, 62)	(33, 51)
Mali	28.2	23.0	19.7	15592	16391	16251
	(19.5, 41.0)	(18.0, 29.7)	(18.4, 21.2)	(11354, 21704)	(13325, 20375)	(15326, 17293)
Malta	3.9	3.6	3.0	17	14	13
	(3.1, 4.8)	(3.1, 4.2)	(2.4, 3.8)	(14, 20)	(13, 16)	(11, 16)
Marshall Islands	12.0	11.7	10.9	23	20	15
	(7.3, 20.6)	(7.3, 19.2)	(6.5, 18.2)	(15, 36)	(13, 30)	(10, 23)
Mauritania	30.0	25.4	22.0	3117	3341	3385
	(18.1, 49.7)	(15.9, 40.8)	(13.3, 35.9)	(2004, 4840)	(2238, 5004)	(2208, 5160)
Mauritius	12.5	9.0	10.3	255	136	133
	(11.5, 13.7)	(8.3, 9.7)	(9.3, 11.4)	(236, 275)	(127, 145)	(121, 146)
Mexico	9.6	7.3	6.8	22850	16826	15136
	(6.7, 13.9)	(5.9, 9.2)	(5.3, 8.8)	(16822, 31362)	(13901, 20456)	(12200, 18839)
Micronesia (Federated States of)	15.1	13.2	11.7	50	34	31
	(9.2, 25.6)	(8.3, 21.5)	(7.2, 19.6)	(32, 77)	(23, 51)	(20, 47)
Monaco	2.2	1.7	1.4	1	1	1
	(1.3, 3.8)	(1.0, 2.9)	(0.8, 2.5)	(1, 1)	(0, 1)	(0, 1)
Mongolia	12.1	6.7	5.2	567	458	395
	(10.7, 13.7)	(6.3, 7.2)	(4.2, 6.6)	(510, 631)	(432, 487)	(325, 478)
Montenegro	5.2	4.4	3.6	44	35	26
	(4.5, 6.0)	(3.9, 5.0)	(3.0, 4.3)	(39, 50)	(31, 38)	(22, 31)
Morocco	21.0	16.8	14.0	13754	11743	9562

	(16.3, 27.4)	(13.4, 20.9)	(10.3, 18.8)	(10993, 17236)	(9655, 14265)	(7375, 12354)
Mozambique	27.8	25.5	21.7	22833	25126	25096
	(21.6, 36.7)	(19.9, 32.8)	(16.8, 27.7)	(18366, 28782)	(20460, 31260)	(20130, 31308)
Myanmar	20.0	16.3	14.1	23402	16639	13493
	(12.1, 33.7)	(10.3, 25.8)	(8.6, 23.8)	(15037, 37446)	(11116, 24999)	(8783, 20935)
Namibia	17.5	15.8	14.7	985	1039	1050
	(10.7, 28.4)	(9.8, 24.7)	(8.8, 24.0)	(646, 1500)	(708, 1525)	(690, 1602)
Nauru	15.0	14.4	13.1	5	5	4
	(8.8, 24.8)	(8.9, 22.9)	(7.7, 21.5)	(3, 8)	(3, 7)	(3, 6)
Nepal	31.1	23.0	17.5	24153	14330	9997
	(24.1, 40.2)	(18.2, 29.1)	(12.9, 23.9)	(19285, 30180)	(11710, 17448)	(7663, 13127)
Netherlands	5.2	3.0	2.3	1025	542	400
	(4.9, 5.5)	(2.8, 3.2)	(1.9, 2.8)	(976, 1075)	(516, 568)	(337, 477)
New Zealand	3.6	3.3	2.7	203	211	160
	(3.2, 4.1)	(3.0, 3.6)	(2.1, 3.4)	(184, 225)	(196, 227)	(133, 195)
Nicaragua	15.4	12.6	10.8	2142	1753	1448
	(11.3, 20.6)	(9.8, 16.3)	(7.5, 15.4)	(1637, 2780)	(1414, 2168)	(1048, 1976)
Niger	27.3	21.9	19.6	17090	18416	21283
	(18.9, 40.3)	(16.8, 29.2)	(13.5, 28.4)	(12476, 23843)	(14689, 23432)	(15558, 29051)
Nigeria	27.5	23.7	22.2	149396	159399	171428
	(16.4, 45.2)	(14.9, 37.0)	(13.4, 35.9)	(96254, 230361)	(107518, 237094)	(112440, 260571)
Niue	11.1	11.1	9.4	0	0	0
	(6.6, 18.2)	(6.8, 17.7)	(5.7, 15.7)	(0, 1)	(0, 0)	(0, 0)
Norway	3.7	2.9	2.4	213	175	146
	(3.3, 4.0)	(2.7, 3.1)	(2.1, 2.8)	(197, 230)	(164, 187)	(129, 163)
Oman	8.0	6.2	5.6	450	429	509
	(6.0, 10.9)	(5.7, 6.8)	(5.1, 6.0)	(352, 582)	(399, 463)	(475, 543)
Pakistan	39.9	36.5	30.6	204880	204648	190483
	(31.1, 51.7)	(31.5, 42.4)	(24.9, 37.8)	(164694, 256014)	(180187, 232700)	(159505, 228681)
Palau	10.6	9.2	7.7	3	2	2
	(6.4, 17.5)	(5.9, 14.7)	(4.7, 12.7)	(2, 5)	(2, 4)	(1, 3)
Panama	11.5	9.5	7.7	827	739	617
	(7.0, 19.2)	(5.9, 15.0)	(4.7, 12.7)	(541, 1272)	(493, 1084)	(407, 949)
Papua New Guinea	19.3	17.6	16.1	3902	3911	3850
	(11.5, 32.1)	(10.9, 27.8)	(9.6, 26.4)	(2502, 6078)	(2629, 5801)	(2489, 5810)
Paraguay	17.1	13.4	10.5	2487	1905	1526
	(12.8, 22.2)	(9.7, 18.1)	(7.1, 15.4)	(1972, 3124)	(1471, 2442)	(1095, 2123)
Peru	13.6	9.0	7.1	8481	5433	4080
	(10.5, 17.8)	(7.8, 10.4)	(5.5, 8.8)	(6862, 10614)	(4796, 6152)	(3324, 4949)
Philippines	14.0	12.3	10.4	32900	29338	22966
	(11.1, 17.9)	(10.0, 15.1)	(7.6, 14.2)	(26854, 40519)	(24704, 34843)	(17614, 29758)
Poland	4.3	3.0	2.3	1627	1171	859
	(4.0, 4.5)	(2.8, 3.1)	(2.2, 2.5)	(1555, 1704)	(1127, 1217)	(817, 904)
Portugal	4.0	2.7	2.5	454	256	197
	(3.7, 4.3)	(2.5, 2.8)	(2.3, 2.7)	(427, 483)	(241, 271)	(182, 213)
Qatar	6.3	6.6	5.4	75	135	144
	(5.6, 7.2)	(6.0, 7.2)	(4.1, 7.3)	(67, 84)	(125, 146)	(114, 185)
Republic of Korea	3.1	2.1	1.7	1758	945	625
	(2.3, 4.1)	(2.0, 2.2)	(1.5, 1.9)	(1371, 2233)	(908, 984)	(572, 684)
Republic of Moldova	11.7	8.1	6.9	567	369	278
	(7.9, 17.2)	(5.4, 12.0)	(4.3, 10.8)	(408, 789)	(266, 515)	(189, 406)
Northern Macedonia	10.8	6.2	4.1	288	143	91
	(9.9, 11.7)	(5.4, 7.0)	(3.5, 4.8)	(266, 309)	(128, 159)	(80, 105)

Romania	6.7	4.1	3.2	1464	888	604
	(5.4, 8.3)	(3.9, 4.3)	(2.9, 3.6)	(1220, 1759)	(852, 925)	(553, 659)
Russian Federation	6.7	4.6	3.8	8938	8190	6805
	(6.5, 6.9)	(4.5, 4.7)	(3.3, 4.3)	(8671, 9214)	(7979, 8410)	(6060, 7631)
Rwanda	28.7	18.9	16.9	8851	6701	6798
	(21.0, 39.0)	(17.1, 21.0)	(13.9, 20.5)	(6752, 11679)	(6159, 7324)	(5729, 8022)
Saint Kitts and Nevis	10.6	8.5	7.4	8	6	5
	(6.3, 17.9)	(5.3, 13.6)	(4.5, 12.4)	(5, 13)	(4, 9)	(3, 8)
Saint Lucia	13.4	12.2	11.2	39	28	24
	(11.4, 15.9)	(10.5, 14.1)	(9.1, 13.7)	(34, 46)	(25, 32)	(21, 29)
Saint Vincent and the Grenadines	10.9	13.5	12.1	24	24	19
	(8.9, 13.2)	(11.8, 15.4)	(9.5, 15.4)	(20, 28)	(21, 27)	(16, 23)
Samoa	10.8	9.7	8.8	58	51	42
	(6.3, 18.1)	(6.1, 15.5)	(5.2, 14.7)	(38, 90)	(35, 76)	(28, 65)
San Marino	3.3	2.1	1.8	1	1	0
	(2.0, 5.2)	(1.4, 3.2)	(1.1, 2.8)	(1, 1)	(0, 1)	(0, 1)
Sao Tome and Principe	16.9	14.7	12.6	98	96	86
	(10.1, 28.6)	(9.1, 23.9)	(7.4, 21.0)	(63, 152)	(65, 145)	(56, 133)
Saudi Arabia	9.3	6.7	5.0	5156	4102	2984
	(5.6, 15.4)	(4.2, 10.8)	(3.0, 8.4)	(3328, 7938)	(2757, 6094)	(1947, 4556)
Senegal	25.3	22.6	19.7	10018	11270	11157
	(19.9, 32.4)	(19.7, 26.0)	(18.4, 21.2)	(8143, 12477)	(9996, 12720)	(10484, 11821)
Serbia	5.3	4.9	4.4	622	458	367
	(4.9, 5.7)	(4.6, 5.2)	(4.0, 5.0)	(584, 665)	(434, 483)	(331, 406)
Seychelles	9.0	9.3	9.5	14	16	15
	(7.2, 11.2)	(7.9, 11.0)	(7.1, 12.7)	(12, 17)	(14, 18)	(12, 19)
Sierra Leone	34.3	27.7	23.7	7393	7073	6249
	(20.6, 56.5)	(17.2, 43.9)	(14.4, 39.1)	(4763, 11559)	(4751, 10672)	(4072, 9611)
Singapore	3.0	2.3	2.0	149	112	99
	(2.6, 3.3)	(2.0, 2.5)	(1.6, 2.5)	(136, 164)	(102, 123)	(84, 119)
Slovakia	4.0	3.3	2.8	214	189	157
	(3.6, 4.4)	(3.1, 3.6)	(2.5, 3.1)	(198, 232)	(177, 201)	(144, 171)
Slovenia	3.6	2.9	2.5	62	61	50
	(3.1, 4.1)	(2.6, 3.2)	(2.1, 3.1)	(56, 70)	(56, 67)	(43, 59)
Solomon Islands	13.1	11.7	10.1	195	214	217
	(7.8, 21.9)	(7.2, 18.8)	(5.9, 16.8)	(126, 304)	(143, 325)	(140, 339)
Somalia	29.9	29.1	26.8	13377	15939	17738
	(18.0, 48.7)	(18.0, 45.9)	(16.1, 43.9)	(8653, 20709)	(10577, 23935)	(11534, 27337)
South Africa	20.8	17.3	16.4	21766	21016	19612
	(16.1, 26.7)	(14.9, 20.0)	(12.7, 21.3)	(17627, 26897)	(18515, 23895)	(15689, 24581)
South Sudan	34.2	29.9	28.8	9408	11060	11515
	(20.8, 56.1)	(18.9, 47.3)	(17.6, 47.2)	(6117, 14678)	(7460, 16652)	(7490, 17600)
Spain	3.3	2.7	2.2	1300	1334	870
	(3.1, 3.4)	(2.6, 2.8)	(1.9, 2.7)	(1242, 1360)	(1287, 1386)	(758, 1002)
Sri Lanka	10.3	7.4	5.8	3607	2711	1943
	(7.8, 13.6)	(6.5, 8.5)	(5.4, 6.3)	(2852, 4561)	(2422, 3037)	(1822, 2069)
State of Palestine	14.8	12.3	10.4	1811	1690	1499
	(8.8, 25.1)	(7.6, 19.2)	(6.3, 16.9)	(1163, 2821)	(1130, 2505)	(992, 2284)
Sudan	29.6	26.0	22.6	33073	32875	31584
	(19.0, 46.3)	(17.7, 37.9)	(14.5, 34.7)	(22509, 48255)	(23755, 44929)	(21614, 45513)
Suriname	14.4	12.9	11.2	164	142	120
	(8.6, 24.5)	(8.1, 20.7)	(6.8, 18.6)	(108, 254)	(96, 209)	(79, 184)
Sweden	3.7	2.9	2.4	343	326	293
	(3.4, 4.1)	(2.7, 3.1)	(2.1, 2.9)	(320, 367)	(309, 345)	(258, 333)
Switzerland	2.7	2.3	2.2	209	180	197
	(2.4, 3.1)	(2.1, 2.5)	(1.8, 2.7)	(188, 231)	(167, 195)	(166, 234)

Syrian Arab Republic	12.7	10.7	10.9	6618	6102	4649
	(7.7, 21.1)	(6.8, 17.0)	(6.7, 17.9)	(4308, 10208)	(4206, 9043)	(3077, 7126)
Tajikistan	13.8	10.9	9.0	2673	2623	2542
	(8.1, 23.0)	(6.8, 16.9)	(5.4, 14.8)	(1706, 4180)	(1762, 3865)	(1651, 3886)
Thailand	10.5	7.5	5.8	9683	5965	4098
	(6.3, 17.5)	(4.7, 11.8)	(3.5, 9.4)	(6232, 15062)	(4051, 8807)	(2723, 6223)
Timor-Leste	20.6	15.4	13.0	751	524	498
	(12.2, 34.4)	(9.4, 24.2)	(7.7, 21.6)	(484, 1178)	(347, 786)	(324, 776)
Togo	28.5	25.0	22.4	5739	6130	6062
	(17.4, 47.4)	(15.6, 40.1)	(13.7, 36.8)	(3775, 9026)	(4120, 9200)	(3989, 9299)
Tonga	8.4	7.8	7.7	24	22	19
	(5.1, 14.1)	(4.8, 12.7)	(4.5, 12.8)	(15, 36)	(15, 33)	(13, 30)
Trinidad and Tobago	11.9	10.4	9.1	225	212	161
	(8.7, 16.3)	(9.3, 11.5)	(6.8, 12.1)	(173, 293)	(194, 231)	(126, 206)
Tunisia	16.7	12.1	10.8	2855	2374	2178
	(11.8, 23.7)	(10.1, 14.4)	(9.0, 12.8)	(2128, 3854)	(2047, 2750)	(1868, 2522)
Turkey	12.1	6.2	4.4	16812	8097	5823
	(9.4, 15.4)	(5.7, 6.7)	(4.3, 4.6)	(13562, 20756)	(7561, 8669)	(5636, 6020)
Turkmenistan	10.1	8.7	8.6	1089	1142	1184
	(6.2, 16.6)	(5.4, 13.7)	(5.2, 13.9)	(708, 1653)	(774, 1677)	(784, 1788)
Tuvalu	14.8	13.6	11.9	4	4	3
	(8.9, 24.3)	(8.6, 22.0)	(7.1, 19.7)	(2, 6)	(3, 6)	(2, 5)
Uganda	23.3	20.8	17.8	27555	30869	29928
	(17.9, 30.7)	(17.7, 24.2)	(16.4, 19.4)	(21952, 34678)	(26878, 35280)	(27874, 32240)
Ukraine	6.8	5.2	4.5	2761	2590	1853
	(5.2, 8.9)	(4.8, 5.6)	(3.8, 5.4)	(2179, 3456)	(2414, 2774)	(1600, 2140)
United Arab Emirates	8.4	6.1	4.9	450	579	496
	(7.6, 9.4)	(5.6, 6.6)	(4.3, 5.7)	(412, 493)	(539, 619)	(441, 559)
United Kingdom	4.4	3.8	3.0	3110	3048	2358
	(3.8, 5.3)	(3.6, 4.0)	(2.6, 3.5)	(2697, 3588)	(2932, 3172)	(2070, 2679)
United Republic of Tanzania	25.3	21.8	18.8	36405	39826	40480
	(20.0, 32.1)	(18.3, 26.2)	(14.4, 24.8)	(29837, 44823)	(34114, 46639)	(32131, 51190)
United States	3.3	3.0	3.0	13163	12147	11844
	(3.2, 3.4)	(2.9, 3.1)	(2.6, 3.5)	(12799, 13548)	(11841, 12460)	(10331, 13555)
Uruguay	7.3	4.9	4.7	399	236	225
	(6.8, 7.9)	(4.5, 5.3)	(4.2, 5.4)	(374, 426)	(221, 252)	(203, 251)
Uzbekistan	11.2	8.9	6.5	6357	5840	4535
	(6.7, 18.3)	(5.7, 13.9)	(4.0, 10.7)	(4121, 9734)	(3942, 8589)	(2988, 6864)
Vanuatu	12.2	11.9	11.1	77	92	98
	(7.1, 20.0)	(7.5, 18.8)	(6.8, 18.2)	(49, 120)	(62, 138)	(65, 151)
Venezuela (Bolivarian Republic of)	9.8	8.8	9.4	5699	5263	4865
	(5.6, 16.7)	(5.5, 14.4)	(5.7, 15.4)	(3641, 8991)	(3544, 8006)	(3212, 7516)
Viet Nam	13.3	9.6	7.8	18734	14783	12479
	(8.7, 20.2)	(6.9, 13.7)	(5.2, 11.9)	(13043, 27224)	(11086, 19820)	(8850, 17746)
Yemen	26.6	22.0	23.7	18890	18041	21184
	(18.1, 38.9)	(17.2, 28.5)	(17.1, 32.9)	(13620, 26397)	(14573, 22554)	(16010, 27950)
Zambia	20.7	17.3	14.8	9952	9930	9597
	(15.1, 28.4)	(14.8, 20.3)	(11.8, 18.4)	(7622, 13076)	(8687, 11347)	(7920, 11652)
Zimbabwe	22.8	23.7	16.0	8787	11335	7113
	(16.4, 31.9)	(20.1, 28.0)	(14.9, 17.2)	(6570, 11748)	(9818, 13060)	(6687, 7548)

Table A7: Share of stillbirths, percentage decrease, annual rate of change in stillbirth rate, neonatal mortality rate and mortality rate among children aged 1–59 months, and ratio of stillbirth to neonatal mortality rate, by country or territory

Note: Numbers in brackets are 90 per cent uncertainty intervals.
Significant percentage decrease based on the lower bound of the 90 per cent uncertainty intervals: ****>=50%, ***>=25%-<50% ,** >=10%-<25%,*>=0%-<10%,†No significant decrease

Country/territory	Share of total stillbirths worldwide, 2019	Percentage decrease 2000 to 2019			Annual rate of reduction 20000 to 2019			Ratio of stillbirth rate to neonatal mortlaity rate, 2019
		stillbirth rate	neonatal mortality rate	mortality rate among children aged 1–59 months	stillbirth rate	neonatal mortality rate	mortality rate among children aged 1–59 months	
Afghanistan†	1.80	23.2	40.8	65.1	1.4	2.8	5.5	0.8
	(1.11, 2.69)	(-9.3, 46.2)	(24.3, 55.0)	(55.1, 73.8)	(-0.5, 3.3)	(1.5, 4.2)	(4.2, 7)	(0.5, 1.3)
Albania**	0.01	36.0	38.3	85.7	2.3	2.5	10.2	0.5
	(0.01, 0.01)	(11.9, 53.6)	(22.7, 49.7)	(81.6, 88.9)	(0.7, 4.0)	(1.4, 3.6)	(8.9, 11.6)	(0.4, 0.7)
Algeria***	0.49	44.9	22.6	62.7	3.1	1.4	5.2	0.6
	(0.39, 0.57)	(32, 55.9)	(13.5, 30.0)	(57.6, 67.1)	(2.0, 4.3)	(0.8, 1.9)	(4.5, 5.8)	(0.5, 0.7)
Andorra**	0.00	41.2	63.5	58.9	2.8	5.3	4.7	1.5
	(0.00, 0.00)	(16.7, 58.4)	(-85.7, 92.4)	(-102.1, 91.6)	(1.0, 4.6)	(-3.3, 13.6)	(-3.7, 13)	(0.4, 6.1)
Angola*	1.32	34.0	45.2	70.0	2.2	3.2	6.3	0.7
	(0.84, 1.94)	(5.9, 53.4)	(-9.7, 76.6)	(42.2, 87.0)	(0.3, 4.0)	(-0.5, 7.6)	(2.9, 10.8)	(0.3, 1.8)
Antigua and Barbuda**	0.00	37.9	62.1	49.5	2.5	5.1	3.6	1.5
	(0.00, 0.00)	(11.9, 57.1)	(43.1, 75.2)	(20.6, 68.0)	(0.7, 4.5)	(3.0, 7.3)	(1.2, 6)	(0.8, 2.4)
Argentina***	0.21	31.8	44.4	63.6	2.0	3.1	5.3	0.9
	(0.18, 0.22)	(26.1, 37)	(40.5, 47.9)	(60.3, 66.7)	(1.6, 2.4)	(2.7, 3.4)	(4.9, 5.8)	(0.8, 1.0)
Armenia**	0.03	37.7	61.1	62.5	2.5	5.0	5.2	2.0
	(0.02, 0.03)	(15.8, 53.8)	(44.0, 74.0)	(45.7, 76.2)	(0.9, 4.1)	(3.1, 7.1)	(3.2, 7.6)	(1.4, 3.0)
Australia**	0.04	31.0	35.4	50.3	1.9	2.3	3.7	1.0
	(0.03, 0.04)	(19.4, 40.9)	(30.5, 39.8)	(45.8, 54.7)	(1.1, 2.8)	(1.9, 2.7)	(3.2, 4.2)	(0.8, 1.2)
Austria**	0.01	22.1	33.2	41.7	1.3	2.1	2.8	1.1
	(0.01, 0.01)	(10.8, 31.9)	(20.3, 44.0)	(29.0, 51.8)	(0.6, 2.0)	(1.2, 3.0)	(1.8, 3.8)	(0.9, 1.3)
Azerbaijan***	0.07	53.2	67.5	77.3	4.0	5.9	7.8	0.8
	(0.05, 0.10)	(33.7, 67.4)	(45.2, 81.5)	(60.5, 87.1)	(2.2, 5.9)	(3.2, 8.9)	(4.9, 10.8)	(0.5, 1.6)
Bahamas†	0.00	7.1	16.5	28.6	0.4	0.9	1.8	1.7
	(0.00, 0.00)	(-11.8, 22.6)	(-13.1, 39.0)	(2.9, 48.8)	(-0.6, 1.4)	(-0.7, 2.6)	(0.2, 3.5)	(1.2, 2.4)
Bahrain**	0.01	30.7	38.1	48.9	1.9	2.5	3.5	2.0
	(0.01, 0.01)	(14, 44.5)	(15.3, 54.5)	(32.9, 61.5)	(0.8, 3.1)	(0.9, 4.1)	(2.1, 5)	(1.4, 2.7)
Bangladesh***	3.69	41.3	55.4	73.9	2.8	4.3	7.1	1.3
	(2.96, 4.22)	(26, 53.6)	(49.4, 60.7)	(69.9, 77.4)	(1.6, 4.0)	(3.6, 4.9)	(6.3, 7.8)	(1.0, 1.6)
Barbados†	0.00	12.0	9.7	26.8	0.7	0.5	1.6	0.9
	(0.00, 0.00)	(-26.4, 38.7)	(-31.3, 38.0)	(-12.5, 54.1)	(-1.2, 2.6)	(-1.4, 2.5)	(-0.6, 4.1)	(0.5, 1.4)
Belarus***	0.01	59.6	79.9	70.1	4.8	8.4	6.4	1.7
	(0.01, 0.01)	(48.8, 68)	(75.2, 83.8)	(64.9, 74.7)	(3.5, 6)	(7.3, 9.6)	(5.5, 7.2)	(1.3, 2.2)

Belgium*	0.02	16.2	32.1	50.9	0.9	2.0	3.7	1.4
	(0.01, 0.02)	(1.6, 29.1)	(17.2, 44.4)	(39.4, 61.4)	(0.1, 1.8)	(1.0, 3.1)	(2.6, 5.0)	(1.1, 1.8)
Belize**	0.00	41.8	32.4	64.1	2.9	2.1	5.4	0.8
	(0.00, 0.00)	(22.6, 56.1)	(17.4, 44.1)	(54.9, 71.7)	(1.3, 4.3)	(1.0, 3.1)	(4.2, 6.6)	(0.6, 1.0)
Benin†	0.45	25.2	22.7	40.4	1.5	1.4	2.7	0.7
	(0.37, 0.51)	(-0.1, 44.7)	(7.8, 35.9)	(30.9, 49.2)	(0, 3.1)	(0.4, 2.3)	(1.9, 3.6)	(0.5, 0.8)
Bhutan**	0.01	40.9	47.7	74.2	2.8	3.4	7.1	0.6
	(0.00, 0.01)	(16.2, 58.1)	(11.9, 69.8)	(56.7, 87.0)	(0.9, 4.6)	(0.7, 6.3)	(4.4, 10.7)	(0.3, 1.0)
Bolivia (Plurinational State of)**	0.11	42.7	49.9	75.8	2.9	3.6	7.5	0.6
	(0.08, 0.15)	(17.9, 59.9)	(30.5, 64.0)	(66.6, 82.9)	(1.0, 4.8)	(1.9, 5.4)	(5.8, 9.3)	(0.4, 1.0)
Bosnia and Herzegovina**	0.00	30.5	38.6	46.0	1.9	2.6	3.2	0.7
	(0.00, 0.00)	(12.3, 44.9)	(25.9, 49.7)	(29.4, 59.1)	(0.7, 3.1)	(1.6, 3.6)	(1.8, 4.7)	(0.5, 0.8)
Botswana†	0.04	-29.2	-209.8	62.3	-1.3	-6.0	5.1	0.8
	(0.04, 0.05)	(-52.1, -9.2)	(-636.2, 0.7)	(13.0, 88.5)	(-2.2, -0.5)	(-10.5, 0)	(0.7, 11.4)	(0.3, 2.5)
Brazil*	1.11	24.9	56.2	63.9	1.5	4.4	5.4	1.0
	(0.88, 1.28)	(8.7, 38.4)	(44.4, 65.4)	(54.3, 72.1)	(0.5, 2.6)	(3.1, 5.6)	(4.1, 6.7)	(0.7, 1.2)
Brunei Darussalam†	0.00	9.4	-21.0	-1.0	0.5	-1.0	-0.1	0.8
	(0.00, 0.00)	(-14, 28.1)	(-46.4, -0.3)	(-22.2, 16.7)	(-0.7, 1.7)	(-2.0, 0.0)	(-1.1, 1.0)	(0.6, 1.0)
Bulgaria***	0.02	34.0	57.5	64.7	2.2	4.5	5.5	1.5
	(0.01, 0.02)	(27.7, 39.8)	(53.5, 61.2)	(61.4, 67.7)	(1.7, 2.7)	(4.0, 5)	(5.0, 5.9)	(1.4, 1.8)
Burkina Faso**	0.77	34.4	36.5	56.1	2.2	2.4	4.3	0.8
	(0.67, 0.82)	(13, 50.8)	(4.1, 59.2)	(38.4, 68.7)	(0.7, 3.7)	(0.2, 4.7)	(2.5, 6.1)	(0.5, 1.2)
Burundi†	0.60	14.2	43.2	70.5	0.8	3.0	6.4	1.2
	(0.53, 0.63)	(-14.3, 35.4)	(10.8, 63.8)	(54.7, 81.1)	(-0.7, 2.3)	(0.6, 5.3)	(4.2, 8.8)	(0.8, 1.9)
Cabo Verde*	0.01	30.6	48.9	71.6	1.9	3.5	6.6	1.2
	(0.00, 0.01)	(1.3, 51)	(33.5, 60.7)	(62.0, 79.2)	(0.1, 3.7)	(2.1, 4.9)	(5.1, 8.3)	(0.8, 2.1)
Cambodia***	0.23	51.2	58.9	83.3	3.8	4.7	9.4	0.9
	(0.15, 0.35)	(28.9, 66.6)	(19.7, 78.8)	(67.6, 91.7)	(1.8, 5.8)	(1.2, 8.2)	(5.9, 13.1)	(0.4, 1.8)
Cameroon†	0.91	19.0	25.9	55.4	1.1	1.6	4.2	0.7
	(0.57, 1.32)	(-14.6, 43.2)	(4.4, 42.7)	(45.5, 64.1)	(-0.7, 3.0)	(0.2, 2.9)	(3.2, 5.4)	(0.5, 1.2)
Canada*	0.05	14.2	10.9	38.3	0.8	0.6	2.5	0.8
	(0.05, 0.06)	(5.6, 21.9)	(3.1, 18.1)	(31.0, 45.5)	(0.3, 1.3)	(0.2, 1.1)	(1.9, 3.2)	(0.7, 0.9)
Central African Republic†	0.26	12.3	19.6	42.2	0.7	1.1	2.9	0.8
	(0.17, 0.38)	(-25.2, 37.9)	(-40.5, 53.8)	(-0.6, 66.9)	(-1.2, 2.5)	(-1.8, 4.1)	(0, 5.8)	(0.4, 1.6)
Chad†	0.96	20.9	25.0	43.4	1.2	1.5	3.0	0.8
	(0.6, 1.39)	(-12.3, 44.7)	(-6.6, 47.6)	(24.2, 58.3)	(-0.6, 3.1)	(-0.3, 3.4)	(1.5, 4.6)	(0.5, 1.4)
Chile*	0.04	25.8	20.2	53.4	1.6	1.2	4.0	0.7
	(0.03, 0.04)	(4.5, 42)	(-7.1, 40.6)	(35.2, 68.4)	(0.2, 2.9)	(-0.4, 2.7)	(2.3, 6.1)	(0.5, 0.9)
China****	4.69	63.2	81.6	74.8	5.3	8.9	7.3	1.4
	(3.99, 5.09)	(56.9, 68.3)	(77.7, 84.9)	(69.2, 79.5)	(4.4, 6)	(7.9, 10)	(6.2, 8.3)	(1.2, 1.8)
Colombia†	0.27	25.7	44.5	45.4	1.6	3.1	3.2	0.9

	(0.2, 0.34)	(-2.9, 45.6)	(19.9, 61.8)	(21.3, 62.9)	(-0.2, 3.2)	(1.2, 5.1)	(1.3, 5.2)	(0.6, 1.6)
Comoros†	0.03	18.9	26.6	45.2	1.1	1.6	3.2	0.8
	(0.02, 0.05)	(-15.1, 42.6)	(-68.1, 68.3)	(-27.2, 76.4)	(-0.7, 2.9)	(-2.7, 6)	(-1.3, 7.6)	(0.3, 2.5)
Congo†	0.14	26.9	37.0	66.2	1.6	2.4	5.7	0.8
	(0.09, 0.2)	(-4.5, 48.5)	(-12.9, 64.3)	(40.3, 80.7)	(-0.2, 3.5)	(-0.6, 5.4)	(2.7, 8.6)	(0.4, 1.6)
Cook Islands**	0.00	46.0	58.8	56.5	3.2	4.7	4.4	1.3
	(0.00, 0.00)	(24.6, 61.4)	(26.0, 76.5)	(7.7, 91.3)	(1.5, 5)	(1.6, 7.6)	(0.4, 10.4)	(0.6, 2.7)
Costa Rica†	0.02	7.7	19.8	54.3	0.4	1.2	4.1	0.7
	(0.01, 0.02)	(-8.9, 21.8)	(14.0, 25.2)	(49.4, 58.7)	(-0.4, 1.3)	(0.8, 1.5)	(3.6, 4.7)	(0.6, 0.8)
Cote d'Ivoire†	1.11	23.0	26.8	53.0	1.4	1.6	4.0	0.7
	(0.69, 1.63)	(-9.2, 45.6)	(4.9, 44.7)	(39.3, 63.6)	(-0.5, 3.2)	(0.3, 3.1)	(2.6, 5.3)	(0.4, 1.2)
Croatia***	0.01	41.9	47.8	32.0	2.9	3.4	2.0	1.0
	(0.00, 0.01)	(32.5, 49.8)	(39.1, 55.6)	(15.3, 45.2)	(2.1, 3.6)	(2.6, 4.3)	(0.9, 3.2)	(0.8, 1.2)
Cuba***	0.04	36.8	49.2	34.5	2.4	3.6	2.2	3.2
	(0.04, 0.04)	(32.3, 41)	(36.7, 58.1)	(18.7, 46.0)	(2.1, 2.8)	(2.4, 4.6)	(1.1, 3.2)	(2.6, 3.7)
Cyprus**	0.00	42.5	63.8	66.6	2.9	5.4	5.8	1.9
	(0.00, 0.00)	(19.7, 59.3)	(43.7, 76.2)	(45.9, 79.4)	(1.2, 4.7)	(3.0, 7.5)	(3.2, 8.3)	(1.2, 2.9)
Czechia†	0.01	6.9	39.7	44.3	0.4	2.7	3.1	1.6
	(0.01, 0.02)	(-2.9, 15.8)	(32.9, 45.9)	(37.8, 50.0)	(-0.2, 0.9)	(2.1, 3.2)	(2.5, 3.7)	(1.4, 2.0)
Democratic People's Republic of Korea**	0.15	38.0	63.9	77.2	2.5	5.4	7.8	0.9
	(0.10, 0.22)	(12.2, 56.4)	(54.3, 72.4)	(70.7, 84.1)	(0.7, 4.4)	(4.1, 6.8)	(6.5, 9.7)	(0.5, 1.7)
Democratic Republic of the Congo†	5.03	19.4	28.8	53.1	1.1	1.8	4.0	1.0
	(3.96, 5.90)	(-12.8, 42.6)	(-14.6, 57.8)	(27.2, 71.0)	(-0.6, 2.9)	(-0.7, 4.5)	(1.7, 6.5)	(0.6, 1.7)
Denmark**	0.01	35.9	14.1	65.3	2.3	0.8	5.6	0.7
	(0.01, 0.01)	(21.9, 47.6)	(0.1, 29.9)	(42.2, 81.5)	(1.3, 3.4)	(0, 1.9)	(2.9, 8.9)	(0.6, 0.9)
Djibouti†	0.03	20.2	30.6	53.5	1.2	1.9	4.0	0.9
	(0.02, 0.04)	(-13.9, 43.5)	(-21.0, 61.6)	(20.0, 76.0)	(-0.7, 3.0)	(-1, 5)	(1.2, 7.5)	(0.5, 1.7)
Dominica†	0.00	-19.4	-118.2	-63.5	-0.9	-4.1	-2.6	0.5
	(0.00, 0.00)	(-72.3, 16.3)	(-162.8, -80.6)	(-199.7, -42.6)	(-2.9, 0.9)	(-5.1, -3.1)	(-5.8, -1.9)	(0.4, 0.9)
Dominican Republic†	0.11	25.7	17.2	50.4	1.6	1.0	3.7	0.6
	(0.07, 0.17)	(-5.9, 48.7)	(-24.3, 44.4)	(19.7, 69.4)	(-0.3, 3.5)	(-1.1, 3.1)	(1.2, 6.2)	(0.4, 1.2)
Ecuador**	0.15	44.5	51.2	52.3	3.1	3.8	3.9	1.2
	(0.11, 0.2)	(22.7, 59.6)	(40.4, 60.1)	(41.6, 61.0)	(1.4, 4.8)	(2.7, 4.8)	(2.8, 5)	(0.9, 1.8)
Egypt**	1.20	48.3	50.1	62.9	3.5	3.7	5.2	0.8
	(0.76, 1.81)	(24.6, 64.2)	(26.9, 65.7)	(45.5, 74.6)	(1.5, 5.4)	(1.6, 5.6)	(3.2, 7.2)	(0.5, 1.6)
El Salvador***	0.06	49.7	55.6	62.9	3.6	4.3	5.2	1.5
	(0.04, 0.08)	(30.4, 64.2)	(25.5, 72.8)	(40.0, 78.3)	(1.9, 5.4)	(1.5, 6.9)	(2.7, 8)	(0.8, 2.8)
Equatorial Guinea†	0.03	16.4	34.7	53.5	0.9	2.2	4.0	0.5
	(0.02, 0.05)	(-17.8, 41.4)	(-20.3, 67.1)	(20.2, 74.2)	(-0.9, 2.8)	(-1, 5.9)	(1.2, 7.1)	(0.2, 1.2)
Eritrea†	0.10	21.3	32.8	61.8	1.3	2.1	5.1	1.0

	(0.06, 0.14)	(-11.7, 45.1)	(-15.8, 61.1)	(34.4, 78.1)	(-0.6, 3.2)	(-0.8, 5)	(2.2, 8)	(0.5, 2.0)
Estonia***	0.00	53.5	79.9	76.5	4.0	8.5	7.6	2.0
	(0.00, 0.00)	(44.1, 61.2)	(74.8, 84.0)	(70.7, 81.0)	(3.1, 5)	(7.2, 9.7)	(6.5, 8.7)	(1.7, 2.8)
Eswatini†	0.02	15.6	14.5	65.0	0.9	0.8	5.5	0.7
	(0.01, 0.03)	(-18.6, 39.7)	(-39.0, 48.2)	(45.4, 77.8)	(-0.9, 2.7)	(-1.7, 3.5)	(3.2, 7.9)	(0.4, 1.3)
Ethiopia*	4.60	31.2	42.7	75.4	2.0	2.9	7.4	0.9
	(2.92, 6.66)	(2.3, 52.2)	(27.6, 54.5)	(69.2, 80.8)	(0.1, 3.9)	(1.7, 4.1)	(6.2, 8.7)	(0.6, 1.4)
Fiji†	0.01	15.9	-19.1	-9.5	0.9	-0.9	-0.5	0.8
	(0.01, 0.01)	(-10.5, 35.7)	(-45.7, 3.3)	(-29.4, 8.2)	(-0.5, 2.3)	(-2, 0.2)	(-1.4, 0.4)	(0.6, 1.0)
Finland**	0.01	23.9	43.6	45.3	1.4	3.0	3.2	1.5
	(0.00, 0.01)	(11.7, 34.6)	(35.1, 50.7)	(35.8, 53.6)	(0.7, 2.2)	(2.3, 3.7)	(2.3, 4.0)	(1.1, 1.6)
France†	0.16	13.2	3.4	31.3	0.7	0.2	2.0	1.6
	(0.13, 0.18)	(-2.2, 26.5)	(-5.3, 11.1)	(24.3, 38.1)	(-0.1, 1.6)	(-0.3, 0.6)	(1.5, 2.5)	(1.3, 1.9)
Gabon†	0.05	21.4	28.9	60.3	1.3	1.8	4.9	0.7
	(0.03, 0.07)	(-11.1, 45.1)	(-19.7, 58.2)	(34.5, 77.0)	(-0.6, 3.2)	(-0.9, 4.6)	(2.2, 7.7)	(0.4, 1.3)
Gambia†	0.10	19.4	27.9	67.5	1.1	1.7	5.9	0.8
	(0.07, 0.14)	(-15.1, 43.3)	(3.5, 44.8)	(58.7, 75.1)	(-0.7, 3.0)	(0.2, 3.1)	(4.7, 7.3)	(0.5, 1.2)
Georgia****	0.02	62.3	78.2	67.4	5.1	8.0	5.9	1.2
	(0.01, 0.02)	(53.4, 69.8)	(72.8, 82.6)	(58.6, 74.6)	(4.0, 6.3)	(6.8, 9.2)	(4.6, 7.2)	(1.0, 1.4)
Germany†	0.11	7.9	17.9	40.7	0.4	1.0	2.8	1.2
	(0.09, 0.12)	(-9.1, 21.4)	(12.2, 23.3)	(35.8, 45.7)	(-0.5, 1.3)	(0.7, 1.4)	(2.3, 3.2)	(1.0, 1.4)
Ghana†	0.99	24.5	36.0	64.1	1.5	2.3	5.4	0.9
	(0.75, 1.21)	(-2.2, 43.9)	(18.3, 49.8)	(54.6, 71.9)	(-0.1, 3.0)	(1.1, 3.6)	(4.2, 6.7)	(0.7, 1.3)
Greece**	0.01	32.0	42.5	40.1	2.0	2.9	2.7	1.4
	(0.01, 0.01)	(23.5, 39.7)	(33.3, 50.8)	(29.0, 50.0)	(1.4, 2.7)	(2.1, 3.7)	(1.8, 3.6)	(1.2, 1.8)
Grenada†	0.00	3.8	-37.0	25.6	0.2	-1.7	1.6	0.9
	(0.00, 0.00)	(-36.3, 34.1)	(-79.6, -4.0)	(-1.5, 46.5)	(-1.6, 2.2)	(-3.1, -0.2)	(-0.1, 3.3)	(0.5, 1.4)
Guatemala**	0.28	36.0	42.5	60.2	2.3	2.9	4.8	1.0
	(0.22, 0.33)	(16.8, 50.5)	(24.6, 56.8)	(47.7, 70.2)	(1.0, 3.7)	(1.5, 4.4)	(3.4, 6.4)	(0.8, 1.5)
Guinea†	0.61	18.5	33.3	43.3	1.1	2.1	3.0	0.8
	(0.38, 0.88)	(-14.9, 43.2)	(11.2, 50.3)	(30.4, 53.9)	(-0.7, 3.0)	(0.6, 3.7)	(1.9, 4.1)	(0.5, 1.3)
Guinea-Bissau*	0.11	28.2	35.9	64.1	1.7	2.3	5.4	0.9
	(0.08, 0.15)	(0.5, 48.4)	(-3.4, 61.9)	(44.1, 78.5)	(0, 3.5)	(-0.2, 5.1)	(3.1, 8.1)	(0.5, 1.7)
Guyana†	0.01	23.7	30.4	46.1	1.4	1.9	3.3	0.7
	(0.01, 0.02)	(-8.7, 46.4)	(-15.8, 58.2)	(5.3, 70.2)	(-0.4, 3.3)	(-0.8, 4.6)	(0.3, 6.4)	(0.4, 1.4)
Haiti†	0.28	16.6	14.9	49.5	1.0	0.9	3.6	0.8
	(0.17, 0.41)	(-17.5, 41.2)	(-24.3, 41.7)	(29.3, 63.5)	(-0.8, 2.8)	(-1.1, 2.8)	(1.8, 5.3)	(0.4, 1.4)
Honduras**	0.09	38.7	47.2	61.4	2.6	3.4	5.0	0.9
	(0.07, 0.10)	(20.4, 53.4)	(17.4, 66.2)	(40.2, 76.6)	(1.2, 4.0)	(1.0, 5.7)	(2.7, 7.6)	(0.6, 1.5)
Hungary*	0.02	23.2	65.4	62.1	1.4	5.6	5.1	1.7
	(0.01, 0.02)	(8.1, 36.2)	(59.5, 70.3)	(55.4, 67.9)	(0.4, 2.4)	(4.8, 6.4)	(4.2, 6)	(1.3, 2.1)
Iceland*	0.00	29.4	51.5	50.7	1.8	3.8	3.7	1.9
	(0.00, 0.00)	(7.6, 46.2)	(30.5, 66.2)	(29.5, 66.1)	(0.4, 3.3)	(1.9, 5.7)	(1.8, 5.7)	(1.2, 2.5)
India***	17.33	53.0	51.9	73.7	4.0	3.8	7.0	0.6

	(16.4, 22.36)	(28.2, 59.9)	(45.2, 58.0)	(69.7, 77.3)	(1.7, 4.8)	(3.2, 4.6)	(6.3, 7.8)	(0.5, 0.7)
Indonesia**	2.33	37.8	45.5	61.4	2.5	3.2	5.0	0.8
	(1.73, 2.87)	(15.6, 54.8)	(33.0, 55.1)	(52.5, 68.4)	(0.9, 4.2)	(2.1, 4.2)	(3.9, 6.1)	(0.6, 1.0)
Iran (Islamic Republic of)**	0.53	40.2	54.3	65.9	2.7	4.1	5.7	0.8
	(0.33, 0.79)	(15.1, 58.2)	(16.5, 74.9)	(35.9, 86.0)	(0.9, 4.6)	(0.9, 7.3)	(2.3, 10.3)	(0.4, 1.8)
Iraq†	0.68	28.8	35.1	48.6	1.8	2.3	3.5	0.8
	(0.42, 1.00)	(-2.1, 50.6)	(14.5, 51.5)	(30.6, 62.6)	(-0.1, 3.7)	(0.8, 3.8)	(1.9, 5.2)	(0.5, 1.4)
Ireland***	0.01	42.9	47.6	62.6	2.9	3.4	5.2	1.3
	(0.01, 0.01)	(33.2, 51.4)	(37.0, 56.8)	(53.4, 70.7)	(2.1, 3.8)	(2.4, 4.4)	(4.0, 6.5)	(1.1, 1.7)
Israel**	0.02	33.0	46.1	47.4	2.1	3.2	3.4	1.4
	(0.02, 0.03)	(20.4, 43.5)	(41.5, 50.3)	(42.7, 51.7)	(1.2, 3.0)	(2.8, 3.7)	(2.9, 3.8)	(1.1, 1.5)
Italy†	0.05	15.1	44.5	42.4	0.9	3.1	2.9	1.2
	(0.05, 0.06)	(-1.9, 29.3)	(38.3, 50.1)	(34.1, 49.8)	(-0.1, 1.8)	(2.5, 3.7)	(2.2, 3.6)	(1.1, 1.4)
Jamaica*	0.03	32.9	41.2	24.5	2.1	2.8	1.5	1.3
	(0.02, 0.04)	(9, 50.5)	(-6.3, 67.4)	(-62.0, 70.1)	(0.5, 3.7)	(-0.3, 5.9)	(-2.5, 6.3)	(0.7, 2.5)
Japan***	0.07	40.2	52.3	40.8	2.7	3.9	2.8	1.8
	(0.06, 0.08)	(34.7, 45.4)	(48.4, 55.7)	(37.2, 44.3)	(2.2, 3.2)	(3.5, 4.3)	(2.4, 3.1)	(1.6, 2.0)
Jordan†	0.10	27.5	43.0	41.0	1.7	3.0	2.8	1.0
	(0.07, 0.13)	(-0.4, 48.4)	(20.9, 59.4)	(14.6, 59.7)	(0, 3.5)	(1.2, 4.7)	(0.8, 4.8)	(0.6, 1.5)
Kazakhstan***	0.10	51.7	79.5	70.8	3.8	8.3	6.5	1.2
	(0.08, 0.12)	(34.2, 64.3)	(76.0, 82.4)	(65.7, 74.8)	(2.2, 5.4)	(7.5, 9.1)	(5.6, 7.3)	(1.0, 1.5)
Kenya†	1.53	12.1	26.3	68.6	0.7	1.6	6.1	0.9
	(1.34, 1.60)	(-17.8, 36)	(-4.1, 47.6)	(56.0, 77.5)	(-0.9, 2.4)	(-0.2, 3.4)	(4.3, 7.9)	(0.7, 1.3)
Kiribati†	0.00	15.7	23.0	32.4	0.9	1.4	2.1	0.6
	(0.00, 0.00)	(-20.7, 40.5)	(-44.3, 64.5)	(-22.9, 64.4)	(-1, 2.7)	(-1.9, 5.5)	(-1.1, 5.4)	(0.3, 1.6)
Kuwait†	0.02	17.6	31.0	41.8	1.0	2.0	2.8	1.3
	(0.01, 0.02)	(-17.7, 41.6)	(20.4, 40.0)	(31.8, 50.7)	(-0.9, 2.8)	(1.2, 2.7)	(2.0, 3.7)	(0.8, 1.8)
Kyrgyzstan**	0.05	36.7	40.1	79.6	2.4	2.7	8.4	0.6
	(0.05, 0.06)	(17.6, 51.4)	(22.1, 53.6)	(70.8, 86.3)	(1.0, 3.8)	(1.3, 4.0)	(6.5, 10.5)	(0.5, 1.0)
Lao People's Democratic Republic*	0.14	32.0	42.2	66.1	2.0	2.9	5.7	0.8
	(0.09, 0.2)	(3.6, 52.8)	(21.8, 58.9)	(54.3, 75.7)	(0.2, 3.9)	(1.3, 4.7)	(4.1, 7.4)	(0.5, 1.3)
Latvia***	0.00	45.8	74.1	75.3	3.2	7.1	7.4	1.7
	(0.00, 0.00)	(35.9, 53.9)	(65.4, 80.4)	(67.2, 81.7)	(2.3, 4.1)	(5.6, 8.6)	(5.9, 9)	(1.3, 2.3)
Lebanon**	0.04	41.4	64.5	62.4	2.8	5.4	5.2	1.5
	(0.02, 0.06)	(17.3, 58.9)	(29.6, 84.1)	(26.2, 84.6)	(1.0, 4.7)	(1.8, 9.7)	(1.6, 9.8)	(0.6, 3.9)
Lesotho†	0.08	21.7	-15.9	37.2	1.3	-0.8	2.4	0.7
	(0.06, 0.11)	(-8.2, 43)	(-74.3, 23.6)	(5.8, 58.6)	(-0.4, 3.0)	(-2.9, 1.4)	(0.3, 4.6)	(0.4, 1.0)
Liberia†	0.20	18.8	29.1	63.8	1.1	1.8	5.4	0.7
	(0.13, 0.3)	(-17, 43.3)	(1.8, 47.8)	(52.5, 72.6)	(-0.8, 3.0)	(0.1, 3.4)	(3.9, 6.8)	(0.4, 1.0)
Libya**	0.06	36.8	59.2	58.9	2.4	4.7	4.7	1.4
	(0.03, 0.08)	(10.8, 56.1)	(32.7, 76.2)	(31.1, 77.2)	(0.6, 4.3)	(2.1, 7.6)	(2.0, 7.8)	(0.6, 2.5)
Lithuania**	0.00	39.4	55.1	73.9	2.6	4.2	7.1	1.4

	(0.00, 0.00)	(18.1, 54.8)	(45.7, 63.0)	(68.5, 78.7)	(1.1, 4.2)	(3.2, 5.2)	(6.1, 8.1)	(1.1, 1.7)
Luxembourg†	0.00	8.1	38.5	44.1	0.4	2.6	3.1	2.3
	(0.00, 0.00)	(-19.6, 29.2)	(13.4, 56.4)	(20.6, 61.1)	(-0.9, 1.8)	(0.8, 4.4)	(1.2, 5)	(1.5, 3.2)
Madagascar†	0.75	18.6	34.9	60.1	1.1	2.3	4.8	0.8
	(0.51, 1.01)	(-15, 42.5)	(14.8, 50.4)	(48.6, 68.8)	(-0.7, 2.9)	(0.8, 3.7)	(3.5, 6.1)	(0.5, 1.2)
Malawi*	0.53	26.6	48.9	84.0	1.6	3.5	9.6	0.8
	(0.45, 0.57)	(7.1, 42.3)	(21.8, 67.7)	(76.1, 89.9)	(0.4, 2.9)	(1.3, 5.9)	(7.5, 12.1)	(0.5, 1.3)
Malaysia†	0.15	-11.1	6.3	25.3	-0.6	0.3	1.5	1.2
	(0.13, 0.16)	(-41.3, 12.6)	(-9.9, 20.2)	(11.5, 36.4)	(-1.8, 0.7)	(-0.5, 1.2)	(0.6, 2.4)	(1.0, 1.3)
Maldives***	0.00	57.6	77.4	84.5	4.5	7.8	9.8	1.2
	(0.00, 0.00)	(41.8, 69.5)	(71.3, 82.0)	(79.4, 88.7)	(2.8, 6.3)	(6.6, 9)	(8.3, 11.5)	(0.9, 1.5)
Mali*	0.83	30.2	36.6	55.5	1.9	2.4	4.3	0.6
	(0.73, 0.87)	(4.5, 49.5)	(16.5, 52.4)	(43.5, 65.0)	(0.2, 3.6)	(0.9, 3.9)	(3.0, 5.5)	(0.5, 0.8)
Malta†	0.00	21.6	7.2	9.4	1.3	0.4	0.5	0.6
	(0.00, 0.00)	(-1.5, 39.5)	(-21.5, 28.7)	(-22.0, 33.9)	(-0.1, 2.6)	(-1, 1.8)	(-1, 2.2)	(0.5, 0.9)
Marshall Islands†	0.00	9.8	16.3	27.3	0.5	0.9	1.7	0.7
	(0.00, 0.00)	(-28.1, 37.8)	(-51.5, 53.6)	(-25.8, 60.8)	(-1.3, 2.5)	(-2.2, 4.0)	(-1.2, 4.9)	(0.4, 1.4)
Mauritania†	0.17	26.8	25.4	42.1	1.6	1.5	2.9	0.7
	(0.11, 0.25)	(-4.7, 49.1)	(-46.6, 62.7)	(-14.9, 71.5)	(-0.2, 3.6)	(-2, 5.2)	(-0.7, 6.6)	(0.3, 1.6)
Mauritius*	0.01	17.8	17.8	8.9	1.0	1.0	0.5	1.0
	(0.01, 0.01)	(7.7, 27.1)	(6.1, 27.7)	(-6.8, 22.4)	(0.4, 1.7)	(0.3, 1.7)	(-0.3, 1.3)	(0.9, 1.2)
Mexico*	0.77	29.0	39.7	60.2	1.8	2.7	4.9	0.8
	(0.59, 0.92)	(0.9, 50.1)	(29.7, 48.1)	(53.2, 66.3)	(0, 3.7)	(1.9, 3.5)	(4.0, 5.7)	(0.6, 1.0)
Micronesia (Federated States of)†	0.00	22.7	33.8	53.2	1.4	2.2	4.0	0.7
	(0.00, 0.00)	(-9, 45.6)	(-47.4, 70.3)	(-6.5, 80.0)	(-0.5, 3.2)	(-2, 6.4)	(-0.3, 8.5)	(0.3, 2.2)
Monaco*	0.00	35.6	40.8	38.8	2.3	2.8	2.6	0.8
	(0.00, 0.00)	(8.4, 55.1)	(7.4, 62.4)	(3.0, 63.9)	(0.5, 4.2)	(0.4, 5.2)	(0.2, 5.4)	(0.6, 2.4)
Mongolia***	0.02	56.8	65.4	82.4	4.4	5.6	9.2	0.6
	(0.02, 0.02)	(46.2, 65.3)	(59.5, 70.2)	(80.1, 84.6)	(3.3, 5.6)	(4.8, 6.4)	(8.5, 9.8)	(0.5, 0.8)
Montenegro**	0.00	31.2	85.1	81.5	2.0	10.0	8.9	2.7
	(0.00, 0.00)	(15.3, 44)	(80.3, 89.0)	(74.5, 86.7)	(0.9, 3.1)	(8.6, 11.6)	(7.2, 10.6)	(1.9, 3.8)
Morocco**	0.49	33.5	49.3	66.0	2.1	3.6	5.7	1.0
	(0.36, 0.60)	(10.6, 51)	(31.0, 63.0)	(52.3, 75.9)	(0.6, 3.8)	(2.0, 5.2)	(3.9, 7.5)	(0.7, 1.5)
Mozambique†	1.28	22.0	38.1	63.7	1.3	2.5	5.3	0.8
	(0.98, 1.54)	(-2.4, 40.9)	(6.8, 57.6)	(45.4, 75.0)	(-0.1, 2.8)	(0.4, 4.5)	(3.2, 7.3)	(0.5, 1.2)
Myanmar†	0.69	29.3	39.8	57.7	1.8	2.7	4.5	0.6
	(0.43, 1.02)	(-0.1, 51.1)	(11.0, 60.9)	(37.8, 72.5)	(0, 3.8)	(0.6, 4.9)	(2.5, 6.8)	(0.4, 1.3)
Namibia†	0.05	15.9	15.4	56.1	0.9	0.9	4.3	0.8
	(0.03, 0.08)	(-19.5, 40.4)	(-59.1, 53.4)	(17.2, 76.0)	(-0.9, 2.7)	(-2.4, 4.0)	(1.0, 7.5)	(0.3, 1.4)
Nauru†	0.00	12.3	18.5	35.9	0.7	1.1	2.3	0.7
	(0.00, 0.00)	(-23.9, 38.3)	(-55.3, 59.7)	(-31.1, 75.1)	(-1.1, 2.5)	(-2.3, 4.8)	(-1.4, 7.3)	(0.3, 1.5)
Nepal**	0.51	43.8	51.0	73.5	3.0	3.8	7.0	0.9
	(0.37, 0.64)	(23.3, 58.6)	(34.7, 63.0)	(64.0, 80.3)	(1.4, 4.6)	(2.2, 5.2)	(5.4, 8.6)	(0.6, 1.3)
Netherlands***	0.02	55.3	32.4	38.6	4.2	2.1	2.6	0.9

	(0.02, 0.02)	(46.4, 62.6)	(28.1, 36.5)	(33.2, 43.6)	(3.3, 5.2)	(1.7, 2.4)	(2.1, 3.0)	(0.7, 1.0)
New Zealand*	0.01	26.3	24.8	45.9	1.6	1.5	3.2	1.0
	(0.01, 0.01)	(7.9, 40.6)	(8.3, 40.1)	(32.5, 57.8)	(0.4, 2.7)	(0.5, 2.7)	(2.1, 4.5)	(0.8, 1.5)
Nicaragua*	0.07	29.6	37.9	71.0	1.8	2.5	6.5	1.1
	(0.05, 0.1)	(0.8, 50.1)	(21.7, 49.6)	(63.4, 76.7)	(0, 3.7)	(1.3, 3.6)	(5.3, 7.7)	(0.6, 1.5)
Niger†	1.08	28.4	43.7	69.7	1.8	3.0	6.3	0.8
	(0.76, 1.42)	(-2.1, 50.6)	(8.5, 65.5)	(51.8, 81.3)	(-0.1, 3.7)	(0.5, 5.6)	(3.8, 8.8)	(0.4, 1.4)
Nigeria†	8.72	19.0	22.6	41.2	1.1	1.3	2.8	0.6
	(5.68, 12.25)	(-15.8, 43.1)	(-3.4, 41.3)	(22.5, 54.4)	(-0.8, 3.0)	(-0.2, 2.8)	(1.3, 4.1)	(0.4, 1.0)
Niue†	0.00	15.5	3.4	0.5	0.9	0.2	0.0	0.7
	(0.00, 0.00)	(-21.1, 40.3)	(-111.3, 55.2)	(-116.9, 56.4)	(-1, 2.7)	(-3.9, 4.2)	(-4.1, 4.4)	(0.3, 2.2)
Norway**	0.01	33.5	47.7	52.2	2.1	3.4	3.9	1.7
	(0.01, 0.01)	(23.7, 42.4)	(40.4, 54.3)	(44.6, 58.9)	(1.4, 2.9)	(2.7, 4.1)	(3.1, 4.7)	(1.5, 2.1)
Oman**	0.03	30.7	30.6	30.6	1.9	1.9	1.9	1.1
	(0.02, 0.03)	(10.7, 46.9)	(9.5, 48.5)	(12.0, 47.6)	(0.6, 3.3)	(0.5, 3.5)	(0.7, 3.4)	(0.7, 1.5)
Pakistan†	9.69	23.2	27.6	49.4	1.4	1.7	3.6	0.7
	(7.83, 11.1)	(-0.8, 41.2)	(13.8, 39.4)	(38.6, 58.5)	(0, 2.8)	(0.8, 2.6)	(2.6, 4.6)	(0.6, 1.0)
Palau†	0.00	26.9	40.9	38.7	1.7	2.8	2.6	0.8
	(0.00, 0.00)	(-4.5, 49)	(-17.4, 70.6)	(-19.9, 71.5)	(-0.2, 3.5)	(-0.8, 6.4)	(-1, 6.6)	(0.4, 2.1)
Panama*	0.03	32.9	43.6	41.9	2.1	3.0	2.9	0.9
	(0.02, 0.05)	(4.4, 53.2)	(-4.5, 69.8)	(-7.0, 70.9)	(0.2, 4.0)	(-0.2, 6.3)	(-0.4, 6.5)	(0.4, 2.1)
Papua New Guinea†	0.20	16.6	28.6	43.2	1.0	1.8	3.0	0.7
	(0.12, 0.29)	(-19.4, 41.7)	(-0.4, 50.9)	(19.5, 61.0)	(-0.9, 2.8)	(0, 3.7)	(1.1, 4.9)	(0.4, 1.3)
Paraguay**	0.08	38.2	40.2	45.2	2.5	2.7	3.2	1.0
	(0.05, 0.10)	(13, 55.4)	(-15.7, 68.0)	(-6.1, 71.5)	(0.7, 4.2)	(-0.8, 6)	(-0.3, 6.6)	(0.5, 2.2)
Peru***	0.21	48.2	60.1	69.9	3.5	4.8	6.3	1.1
	(0.16, 0.24)	(32.1, 61.3)	(47.9, 69.0)	(61.1, 76.6)	(2.0, 5)	(3.4, 6.2)	(5.0, 7.6)	(0.9, 1.7)
Philippines*	1.17	25.7	18.6	34.7	1.6	1.1	2.2	0.8
	(0.86, 1.46)	(0.2, 45)	(-10.5, 39.8)	(11.2, 52.2)	(0, 3.1)	(-0.5, 2.7)	(0.6, 3.9)	(0.5, 1.2)
Poland***	0.04	45.5	52.8	52.9	3.2	4.0	4.0	0.9
	(0.04, 0.05)	(41.6, 49.1)	(50.4, 55.2)	(49.9, 55.7)	(2.8, 3.6)	(3.7, 4.2)	(3.6, 4.3)	(0.8, 0.9)
Portugal***	0.01	38.0	41.3	55.4	2.5	2.8	4.2	1.3
	(0.01, 0.01)	(31.6, 43.9)	(34.7, 47.5)	(50.1, 60.2)	(2.0, 3.0)	(2.2, 3.4)	(3.7, 4.8)	(1.1, 1.5)
Qatar†	0.01	14.7	48.7	47.0	0.8	3.5	3.3	1.6
	(0.01, 0.01)	(-11.7, 34.4)	(37.9, 57.2)	(36.4, 56.8)	(-0.6, 2.2)	(2.5, 4.5)	(2.4, 4.4)	(1.2, 2.1)
Republic of Korea***	0.03	44.8	54.8	60.0	3.1	4.2	4.8	1.1
	(0.03, 0.03)	(27.6, 57.3)	(49.4, 59.5)	(55.7, 64.1)	(1.7, 4.5)	(3.6, 4.8)	(4.3, 5.4)	(0.9, 1.3)
Republic of Moldova**	0.01	41.3	48.6	65.7	2.8	3.5	5.6	0.6
	(0.01, 0.02)	(18.4, 58.4)	(23.9, 65.0)	(43.7, 79.8)	(1.1, 4.6)	(1.4, 5.5)	(3.0, 8.4)	(0.4, 1.2)
Republic of North Macedonia****	0.00	62.0	57.1	67.2	5.1	4.4	5.9	1.0
	(0.00, 0.01)	(55.4, 67.4)	(49.6, 63.7)	(59.8, 73.5)	(4.3, 5.9)	(3.6, 5.3)	(4.8, 7)	(1.0, 1.5)

Romania***	0.03	52.0	67.0	67.9	3.9	5.8	6.0	0.9
	(0.03, 0.03)	(41.4, 60.8)	(58.2, 73.7)	(59.9, 74.3)	(2.8, 4.9)	(4.6, 7)	(4.8, 7.1)	(0.8, 1.1)
Russian Federation***	0.35	43.9	70.8	69.7	3.0	6.5	6.3	1.4
	(0.29, 0.38)	(36.8, 50.3)	(66.9, 74.4)	(66.0, 72.8)	(2.4, 3.7)	(5.8, 7.2)	(5.7, 6.9)	(1.3, 1.9)
Rwanda**	0.35	40.9	61.1	87.0	2.8	5.0	10.7	1.1
	(0.28, 0.40)	(19.1, 57)	(30.9, 78.3)	(76.6, 92.7)	(1.1, 4.4)	(1.9, 8.1)	(7.6, 13.8)	(0.6, 1.9)
Saint Kitts and Nevis†	0.00	29.7	38.0	29.7	1.9	2.5	1.9	0.7
	(0.00, 0.00)	(-1.8, 51)	(7.4, 58.1)	(-12.5, 57.4)	(-0.1, 3.8)	(0.4, 4.6)	(-0.6, 4.5)	(0.4, 1.1)
Saint Lucia†	0.00	16.5	-9.4	-40.1	1.0	-0.5	-1.8	0.9
	(0.00, 0.00)	(-4.8, 33.1)	(-34.4, 11.8)	(-79.6, -7.7)	(-0.2, 2.1)	(-1.6, 0.7)	(-3.1, -0.4)	(0.7, 1.1)
Saint Vincent and the Grenadines†	0.00	-11.1	30.9	39.1	-0.6	1.9	2.6	1.3
	(0.00, 0.00)	(-44, 14)	(8.3, 48.3)	(15.1, 56.8)	(-1.9, 0.8)	(0.5, 3.5)	(0.9, 4.4)	(0.9, 1.8)
Samoa†	0.00	18.8	28.3	26.9	1.1	1.7	1.6	1.1
	(0.00, 0.00)	(-16, 43.4)	(-13.4, 55.1)	(-16.9, 56.1)	(-0.8, 3.0)	(-0.7, 4.2)	(-0.8, 4.3)	(0.5, 2.1)
San Marino**	0.00	45.8	74.3	70.1	3.2	7.1	6.3	2.3
	(0.00, 0.00)	(21.6, 62.2)	(42.2, 91.2)	(33.0, 87.7)	(1.3, 5.1)	(2.9, 12.8)	(2.1, 11)	(1.0, 7)
Sao Tome and Principe†	0.00	25.3	37.7	74.5	1.5	2.5	7.2	0.9
	(0.00, 0.01)	(-4.7, 47.5)	(-20.2, 66.2)	(55.9, 86.9)	(-0.2, 3.4)	(-1, 5.7)	(4.3, 10.7)	(0.4, 1.7)
Saudi Arabia**	0.15	45.9	69.9	69.8	3.2	6.3	6.3	1.4
	(0.09, 0.22)	(21.9, 61.6)	(55.7, 80.1)	(53.7, 80.9)	(1.3, 5)	(4.3, 8.5)	(4.1, 8.7)	(0.7, 2.5)
Senegal*	0.57	22.1	42.0	74.8	1.3	2.9	7.3	0.9
	(0.5, 0.59)	(3.6, 37.5)	(23.1, 56.0)	(67.1, 81.0)	(0.2, 2.5)	(1.4, 4.3)	(5.8, 8.7)	(0.7, 1.2)
Serbia*	0.02	15.7	58.7	57.5	0.9	4.7	4.5	1.4
	(0.02, 0.02)	(4.6, 25.2)	(48.0, 67.5)	(45.2, 67.2)	(0.2, 1.5)	(3.4, 5.9)	(3.2, 5.9)	(1.1, 1.8)
Seychelles†	0.00	-5.6	1.0	-11.4	-0.3	0.1	-0.6	1.1
	(0.00, 0.00)	(-43, 21.6)	(-40.5, 31.2)	(-67.0, 27.1)	(-1.9, 1.3)	(-1.8, 2.0)	(-2.7, 1.7)	(0.7, 1.7)
Sierra Leone*	0.32	30.8	36.6	57.1	1.9	2.4	4.5	0.8
	(0.2, 0.47)	(1.3, 51.6)	(17.5, 52.9)	(47.3, 65.1)	(0.1, 3.8)	(1.0, 4.0)	(3.4, 5.5)	(0.5, 1.2)
Singapore**	0.01	33.1	43.8	27.4	2.1	3.0	1.7	2.2
	(0.00, 0.01)	(18, 45.1)	(31.4, 54.2)	(14.8, 38.3)	(1.0, 3.2)	(2.0, 4.1)	(0.8, 2.5)	(1.9, 3.1)
Slovakia**	0.01	29.9	42.7	39.4	1.9	2.9	2.6	1.0
	(0.01, 0.01)	(21.5, 37.6)	(37.1, 47.7)	(33.3, 45.0)	(1.3, 2.5)	(2.4, 3.4)	(2.1, 3.1)	(0.8, 1.1)
Slovenia**	0.00	28.9	64.3	59.2	1.8	5.4	4.7	2.2
	(0.00, 0.00)	(13.1, 41.9)	(56.4, 70.7)	(48.1, 67.3)	(0.7, 2.9)	(4.4, 6.5)	(3.5, 5.9)	(1.7, 2.8)
Solomon Islands†	0.01	23.1	36.3	35.0	1.4	2.4	2.3	1.2
	(0.01, 0.02)	(-8.7, 45.8)	(-3.1, 63.2)	(-1.3, 60.3)	(-0.4, 3.2)	(-0.2, 5.3)	(-0.1, 4.9)	(0.6, 2.3)
Somalia†	0.90	10.4	16.2	37.5	0.6	0.9	2.5	0.7
	(0.57, 1.33)	(-25.9, 36.9)	(-70.7, 60.9)	(-19.3, 67.5)	(-1.2, 2.4)	(-2.8, 4.9)	(-0.9, 5.9)	(0.3, 1.8)
South Africa†	1.00	21.1	23.1	59.2	1.2	1.4	4.7	1.4
	(0.76, 1.21)	(-6.1, 41)	(-2.3, 43.7)	(51.5, 66.0)	(-0.3, 2.8)	(-0.1, 3.0)	(3.8, 5.7)	(1.0, 1.8)
South Sudan†	0.59	15.9	31.0	54.8	0.9	2.0	4.2	0.7

	(0.37, 0.86)	(-17.7, 40.1)	(-66.5, 76.7)	(1.1, 85.2)	(-0.9, 2.7)	(-2.7, 7.7)	(0.1, 10.1)	(0.3, 2.2)
Spain**	0.04	31.2	36.2	50.2	2.0	2.4	3.7	1.2
	(0.04, 0.05)	(20.3, 40.5)	(26.7, 44.9)	(41.2, 58.7)	(1.2, 2.7)	(1.6, 3.1)	(2.8, 4.7)	(1.0, 1.5)
Sri Lanka***	0.10	43.2	55.4	59.5	3.0	4.2	4.8	1.4
	(0.09, 0.10)	(27.7, 55.6)	(44.3, 64.2)	(48.7, 69.0)	(1.7, 4.3)	(3.1, 5.4)	(3.5, 6.2)	(1.1, 1.7)
State of Palestine*	0.08	30.2	35.3	35.4	1.9	2.3	2.3	1.0
	(0.05, 0.11)	(0.6, 50.5)	(4.2, 55.9)	(2.3, 56.8)	(0, 3.7)	(0.2, 4.3)	(0.1, 4.4)	(0.6, 1.8)
Sudan†	1.61	23.6	26.2	53.6	1.4	1.6	4.0	0.8
	(1.06, 2.23)	(-7.8, 46.1)	(-3.5, 47.4)	(36.6, 67.4)	(-0.4, 3.3)	(-0.2, 3.4)	(2.4, 5.9)	(0.5, 1.4)
Suriname†	0.01	22.4	34.9	51.6	1.3	2.3	3.8	1.0
	(0.00, 0.01)	(-9.8, 45.6)	(-11.1, 60.6)	(11.8, 75.6)	(-0.5, 3.2)	(-0.6, 4.9)	(0.7, 7.4)	(0.5, 1.7)
Sweden**	0.01	34.4	40.6	32.9	2.2	2.7	2.1	1.8
	(0.01, 0.02)	(24, 43.1)	(34.1, 46.4)	(24.7, 40.2)	(1.4, 3.0)	(2.2, 3.3)	(1.5, 2.7)	(1.5, 2.1)
Switzerland*	0.01	19.0	20.3	42.4	1.1	1.2	2.9	0.8
	(0.01, 0.01)	(0.6, 33.5)	(8.7, 30.8)	(30.6, 52.7)	(0, 2.1)	(0.5, 1.9)	(1.9, 3.9)	(0.7, 1.0)
Syrian Arab Republic†	0.24	14.4	11.7	1.1	0.8	0.7	0.1	1.0
	(0.15, 0.35)	(-21.8, 39.2)	(-28.0, 51.4)	(-41.3, 46.9)	(-1, 2.6)	(-1.3, 3.8)	(-1.8, 3.3)	(0.6, 1.9)
Tajikistan*	0.13	34.7	47.3	66.4	2.2	3.4	5.7	0.6
	(0.08, 0.19)	(7.3, 54.5)	(10.5, 70.8)	(43.4, 79.7)	(0.4, 4.1)	(0.6, 6.5)	(3.0, 8.4)	(0.3, 1.3)
Thailand**	0.21	45.2	58.6	59.7	3.2	4.6	4.8	1.1
	(0.13, 0.3)	(22.1, 61.5)	(44.1, 68.7)	(41.6, 71.1)	(1.3, 5)	(3.1, 6.1)	(2.8, 6.5)	(0.6, 1.9)
Timor-Leste*	0.03	37.1	46.8	66.0	2.4	3.3	5.7	0.7
	(0.02, 0.04)	(9, 56.1)	(3.5, 72.4)	(40.7, 81.4)	(0.5, 4.3)	(0.2, 6.8)	(2.8, 8.9)	(0.3, 1.5)
Togo†	0.31	21.4	31.4	49.1	1.3	2.0	3.6	0.9
	(0.19, 0.45)	(-10.2, 45.3)	(11.3, 47.8)	(36.3, 60.1)	(-0.5, 3.2)	(0.6, 3.4)	(2.4, 4.8)	(0.5, 1.4)
Tonga†	0.00	8.5	5.9	9.3	0.5	0.3	0.5	1.0
	(0.00, 0.00)	(-30.5, 34.9)	(-48.5, 40.4)	(-34.9, 42.0)	(-1.4, 2.3)	(-2.1, 2.7)	(-1.6, 2.9)	(0.5, 1.7)
Trinidad and Tobago†	0.01	23.5	38.7	36.3	1.4	2.6	2.4	0.8
	(0.01, 0.01)	(-8.1, 46.3)	(-26.3, 70.6)	(-33.9, 72.0)	(-0.4, 3.3)	(-1.2, 6.5)	(-1.5, 6.7)	(0.3, 1.9)
Tunisia**	0.11	35.5	36.6	55.8	2.3	2.4	4.3	0.9
	(0.09, 0.13)	(10.7, 53.8)	(22.9, 47.3)	(42.0, 66.4)	(0.6, 4.1)	(1.4, 3.4)	(2.9, 5.7)	(0.8, 1.1)
Turkey****	0.30	63.4	71.7	76.4	5.3	6.6	7.6	0.8
	(0.26, 0.31)	(54.7, 70.2)	(66.0, 76.3)	(71.8, 80.2)	(4.2, 6.4)	(5.7, 7.6)	(6.7, 8.5)	(0.7, 1.0)
Turkmenistan†	0.06	15.1	20.5	54.4	0.9	1.2	4.1	0.4
	(0.04, 0.09)	(-20.1, 39.7)	(-16.2, 46.9)	(31.6, 69.6)	(-1, 2.7)	(-0.8, 3.3)	(2.0, 6.3)	(0.2, 0.6)
Tuvalu†	0.00	19.5	33.7	54.0	1.1	2.2	4.1	0.7
	(0.00, 0.00)	(-13.8, 43.3)	(-29.2, 65.2)	(4.3, 78.2)	(-0.7, 3.0)	(-1.3, 5.6)	(0.2, 8)	(0.3, 1.6)
Uganda*	1.52	23.5	37.7	77.6	1.4	2.5	7.9	0.9
	(1.33, 1.61)	(3.8, 39.3)	(13.7, 55.3)	(69.9, 83.6)	(0.2, 2.6)	(0.8, 4.2)	(6.3, 9.5)	(0.6, 1.3)
Ukraine**	0.09	33.5	55.1	53.3	2.1	4.2	4.0	0.9
	(0.08, 0.11)	(12.3, 49.1)	(42.7, 67.5)	(32.2, 69.3)	(0.7, 3.6)	(2.9, 5.9)	(2.0, 6.2)	(0.7, 1.3)
United Arab Emirates***	0.03	41.5	32.8	33.8	2.8	2.1	2.2	1.2

	(0.02, 0.03)	(32.2, 49.8)	(15.4, 48.5)	(11.0, 48.4)	(2.0, 3.6)	(0.9, 3.5)	(0.6, 3.5)	(1.0, 1.5)
United Kingdom**	0.12	31.6	26.8	45.7	2.0	1.6	3.2	1.1
	(0.1, 0.13)	(16.9, 43.6)	(22.0, 31.3)	(41.1, 49.9)	(1.0, 3.0)	(1.3, 2.0)	(2.8, 3.6)	(0.9, 1.2)
United Republic of Tanzania*	2.06	25.6	39.5	68.9	1.6	2.6	6.2	0.9
	(1.57, 2.50)	(0.8, 44)	(16.0, 55.6)	(57.2, 77.1)	(0, 3.0)	(0.9, 4.3)	(4.5, 7.8)	(0.6, 1.4)
United States of America†	0.60	9.3	20.3	27.2	0.5	1.2	1.7	0.8
	(0.5, 0.67)	(-4.2, 21.2)	(14.2, 26.0)	(21.1, 32.7)	(-0.2, 1.3)	(0.8, 1.6)	(1.2, 2.1)	(0.7, 0.9)
Uruguay***	0.01	35.8	50.0	66.8	2.3	3.6	5.8	1.1
	(0.01, 0.01)	(27.3, 43.2)	(44.4, 55.1)	(62.3, 70.7)	(1.7, 3.0)	(3.1, 4.2)	(5.1, 6.5)	(1.0, 1.3)
Uzbekistan**	0.23	41.3	64.7	78.3	2.8	5.5	8.0	0.7
	(0.15, 0.34)	(15.9, 58.9)	(51.6, 76.1)	(68.6, 86.3)	(0.9, 4.7)	(3.8, 7.5)	(6.1, 10.5)	(0.4, 1.3)
Vanuatu†	0.00	9.3	9.2	8.0	0.5	0.5	0.4	1.0
	(0.00, 0.01)	(-31.1, 35.7)	(-75.2, 53.1)	(-64.2, 51.7)	(-1.4, 2.3)	(-3, 4.0)	(-2.6, 3.8)	(0.5, 2.2)
Venezuela (Bolivarian Republic of)†	0.25	4.5	-31.9	8.4	0.2	-1.5	0.5	0.6
	(0.16, 0.37)	(-38.2, 33.8)	(-67.2, -0.9)	(-32.5, 42.3)	(-1.7, 2.2)	(-2.7, 0)	(-1.5, 2.9)	(0.4, 1.2)
Viet Nam**	0.63	41.1	32.1	34.1	2.8	2.0	2.2	0.7
	(0.43, 0.86)	(15.5, 58.9)	(-1.3, 53.5)	(1.9, 56.9)	(0.9, 4.7)	(-0.1, 4.0)	(0.1, 4.4)	(0.5, 1.3)
Yemen†	1.08	10.7	27.6	45.9	0.6	1.7	3.2	0.9
	(0.78, 1.37)	(-26.7, 37.1)	(-17.9, 57.3)	(10.0, 68.5)	(-1.2, 2.4)	(-0.9, 4.5)	(0.6, 6.1)	(0.5, 1.6)
Zambia*	0.49	28.6	31.3	67.9	1.8	2.0	6.0	0.6
	(0.39, 0.57)	(1.8, 48.2)	(10.6, 47.6)	(59.1, 75.1)	(0.1, 3.5)	(0.6, 3.4)	(4.7, 7.3)	(0.5, 0.9)
Zimbabwe*	0.36	29.7	2.2	56.6	1.9	0.1	4.4	0.6
	(0.32, 0.38)	(6.2, 47.2)	(-34.5, 29.6)	(40.7, 68.6)	(0.3, 3.4)	(-1.6, 1.8)	(2.7, 6.1)	(0.5, 0.9)

Table A8: Series in SBR database by country or territory

Note: The series name refers to a specific data collection source and estimation method. There are several sources including vital registration data (including sample vital registration data) and sample surveys – where: vital registration series are based on birth and death registration data and survey series are based on data births and stillbirths from pregnancy histories or reproductive calendars.

Country or territory	Data series	Inclusion
Afghanistan	Health Survey 2018	0
	Afghanistan Mortality Survey 2010	0
	HMIS-DHIS2 data submitted to WHO/UN IGME version 2014	0
	Demographic and Health Survey 2015	0
Albania	Demographic and Health Survey 2008-09	0
	HMIS-DHIS2 data submitted to WHO/UN IGME version 2014	1
	Vital Registration data submitted to WHO/UN IGME version 2018	1
	Demographic and Health Survey 2017-18	0
Algeria	Vital Registration data submitted to WHO/UN IGME version 2020	1
Andorra	Birth or Death Registry	1
Angola	Inquérito de Indicadores Múltiplos e de Saúde 2015-16	0
Argentina	Vital Registration data submitted to WHO/UN IGME version 2020	1
Armenia	Vital Registration data submitted to WHO/UN IGME version 2020	1
	Demographic and Health Survey 2005	0

	Demographic and Health Survey 2010	0
	Demographic and Health Survey 2015-16	0
Australia	Vital Registration data submitted to WHO/UN IGME version 2019	1
Austria	Vital Registration data submitted to WHO/UN IGME version 2020	1
Azerbaijan	Vital Registration data submitted to WHO/UN IGME version 2014	0
	Demographic and Health Survey 2006	1
Bahamas	Vital Registration data submitted to WHO/UN IGME version 2019	1
Bahrain	HMIS-DHIS2 data submitted to WHO/UN IGME version 2014	1
Bangladesh	Demographic and Health Survey 2004	1
	Cherry 2008	1
	West 2011	1
	Demographic and Health Survey 2007	1
	Baqui 2011	1
	Khanam 2017	0
	Prost 2013	1
	Ellis 2011	0
	Azad 2010	1
	Nahar 2013	0
	Shah 2014	1
	Sikder 2014	0
	Demographic and Health Survey 2011	1
	West 2014	0
	HMIS-DHIS2 data submitted to WHO/UN IGME version 2014	0
	Halim 2018	1
	Owais 2013	0
	Nelson 2018	1
	Hanifi 2012, 2013, 2014, 2015, 2016, 2017	1
	Demographic and Health Survey 2014	1
	AMANHI 2018	1
	Vital Registration data submitted to WHO/UN IGME version 2019	0
	Child Health and Mortality Prevention Surveillance Network (CHAMPS) Program	1
Belarus	Vital Registration data submitted to WHO/UN IGME version 2019	1
Belgium	Vital Registration data submitted to WHO/UN IGME version 2019	1
Belize	Vital Registration data submitted to WHO/UN IGME version 2014	1
Benin	Enquête démographique et de santé 2006	0
	HMIS-DHIS2 data submitted to WHO/UN IGME version 2014	1
	Demographic and Health Survey 2011-12	0
	Demographic and Health Survey 2017-18	0
Bhutan	HMIS-DHIS2 data submitted to WHO/UN IGME version 2019	1
Bolivia (Plurinational State of)	Encuesta Nacional de Demografía y Salud 2003	0
	Encuesta Nacional de Demografía y Salud 2008	0
	Demographic and Health Survey 2016	1
Bosnia and Herzegovina	Vital Registration data submitted to WHO/UN IGME version 2015	1
	Skokic 2006	0
Botswana	HMIS-DHIS2 data submitted to WHO/UN IGME version 2020 data obtained by active search version 2020	1
	Vital Registration data submitted to WHO/UN IGME version 2020 data obtained by active search version 2020	1
Brazil	Vital Registration data submitted to WHO/UN IGME version 2019	0
	HMIS-DHIS2 data submitted to WHO/UN IGME version 2020	1
	Vieira 2016	0
	Barros 2005	0
	Nascimento 2017	0
	Maria 2017	0
	Andrews 2017	0
	Silva 2015	0
Brunei Darussalam	Vital Registration data submitted to WHO/UN IGME version 2019	1

	Birth or Death Registry	1
Bulgaria	Vital Registration data submitted to WHO/UN IGME version 2019	1
Burkina Faso	Enquête démographique et de santé 2003	0
	HMIS-DHIS2 data submitted to WHO/UN IGME version 2014	1
	Roberfroid 2008	0
	Enquête démographique et de santé et à Indicateurs Multiples 2010	0
Burundi	Enquête démographique et de santé 2010	1
	Enquête démographique et de santé 2016-17	1
	HMIS-DHIS2 data submitted to WHO/UN IGME version 2020	1
Cape Verde	II Inquérito Demográfico e de Saúde Reproductiva 2005	1
Cambodia	Demographic and Health Survey 2010	0
	Demographic and Health Survey 2014	0
Cameroon	Demographic and Health Survey 2018	0
Canada	Vital Registration data submitted to WHO/UN IGME version 2019	1
	Vital Registration data submitted to WHO/UN IGME version 2018	0
Chad	HMIS-DHIS2 data submitted to WHO/UN IGME version 2014	0
Chile	Vital Registration data submitted to WHO/UN IGME version 2014	1
China	Vital Registration data submitted to WHO/UN IGME version 2014	1
	Zheng 2015	1
	Zeng 2008	1
	Xu 2012	0
	Liu 2013	0
	Sun 2013	0
	Qu 2019	0
	Wang 2016	0
	Yang 2018	0
	Zhu 2016	0
	Zhong	0
	Ge 2016	0
	Wang 2018	0
	Zang 2019	1
Colombia	Vital Registration data submitted to WHO/UN IGME version 2020	0
	Encuesta Nacional de Demografía y Salud 2005	1
	Encuesta Nacional de Demografía y Salud 2010	0
	Encuesta Nacional de Demografía y Salud 2015	1
Comoros	Deuxième Enquête Démographique, de Santé 2012	0
Congo	HMIS-DHIS2 data submitted to WHO/UN IGME version 2020	0
Cook Islands	Vital Registration data submitted to WHO/UN IGME version 2015	1
Costa Rica	Vital Registration data submitted to WHO/UN IGME version 2019	1
Cote d'Ivoire	Waiswa	0
Croatia	Vital Registration data submitted to WHO/UN IGME version 2014	1
	Birth or Death Registry	1
Cuba	Vital Registration data submitted to WHO/UN IGME version 2020	1
Cyprus	Birth or Death Registry	0
	Vital Registration data submitted to WHO/UN IGME version NA data submitted to WHO/UN IGME version 2019	1
Czechia	Vital Registration data submitted to WHO/UN IGME version 2020	1
Democratic Republic of the Congo	Global Network Re-analysed	1
	McClure 2007	0
	McClure 2011	0
	Ntambue 2013	0
	AMANHI 2018	1
Denmark	Vital Registration data submitted to WHO/UN IGME version 2014	1
Djibouti	HMIS-DHIS2 data submitted to WHO/UN IGME version 2014	1
Dominican Republic	Encuesta Demográfica y de Salud 2002	0
	HMIS-DHIS2 data submitted to WHO/UN IGME version 2020	0
	McClure 2018	0

Ecuador	Vital Registration data submitted to WHO/UN IGME version 2019	0
	Encuesta Nacional de Salud y Nutrición 2012	1
	Encuesta Demográfica y de Salud Materna e Infantil 2004	1
	Encuesta Nacional de Salud y Nutrición 2018	0
Egypt	HMIS-DHIS2 data submitted to WHO/UN IGME version 2020	0
	Demographic and Health Survey 2003	0
	Demographic and Health Survey 2005	0
	Demographic and Health Survey 2008	0
	Demographic and Health Survey 2014	0
	Demographic and Health Survey 2015	0
El Salvador	Encuesta Nacional de Salud Familiar 2002-03	1
	Encuesta Nacional de Salud Familiar 2008	1
	HMIS-DHIS2 data submitted to WHO/UN IGME version 2020 data obtained by active search version 2020	0
Equatorial Guinea	Demographic and Health Survey 2011	0
Estonia	Birth or Death Registry	1
Eswatini	Multiple Indicator Cluster Survey 2010	1
	Demographic and Health Survey 2006-07	1
Ethiopia	Demographic and Health Survey 2005	0
	Yaya 2014	0
	Demographic and Health Survey 2011	0
	Assefa 2012	0
	Andargie 2013	0
	Waiswa	0
	Yirgu 2016	0
	Demographic and Health Survey 2016	0
	HMIS-DHIS2 data submitted to WHO/UN IGME version 2020	0
	Atnafu 2016	0
	Child Health and Mortality Prevention Surveillance Network (CHAMPS) Program	0
Fiji	HMIS-DHIS2 data submitted to WHO/UN IGME version 2015	1
Finland	Birth or Death Registry	1
France	Vital Registration data submitted to WHO/UN IGME version 2014	1
	Birth or Death Registry	1
Gambia	Demographic and Health Survey 2013	0
	HMIS-DHIS2 data submitted to WHO/UN IGME version 2020	0
	Waiswa	1
Georgia	Birth or Death Registry	1
	Reproductive Health Survey 2005	1
	Vital Registration data submitted to WHO/UN IGME version 2018	0
Germany	Birth or Death Registry	1
	Vital Registration data submitted to WHO/UN IGME version NA data submitted to WHO/UN IGME version 2019	1
Ghana	Demographic and Health Survey 2003	0
	Edmond 2008	0
	Kirkwood 2010	1
	Maternal Health Survey 2007	1
	Maternal Health Survey 2017	1
	Ha 2012	0
	Demographic and Health Survey 2008	0
	HMIS-DHIS2 data submitted to WHO/UN IGME version 2018 data obtained by active search version 2018	0
	Waiswa	1
	Demographic and Health Survey 2014	0
	AMANHI 2018	1
Greece	Vital Registration data submitted to WHO/UN IGME version 2019	1
Grenada	Vital Registration data submitted to WHO/UN IGME version 2019	0
Guatemala	Vital Registration data submitted to WHO/UN IGME version 2019	0

	Encuesta Nacional de Salud Materno Infantil 2002	1
	Encuesta Nacional de Salud Materno Infantil 2008-09	1
	McClure 2011	0
	Global Network Re-analysed	1
	Saleem 2018	0
	Saleem 2014	0
	Encuesta Nacional de Salud Materno Infantil 2014-15	1
	McClure 2018	0
Guinea	Enquête démographique et de santé 2005	0
	HMIS-DHIS2 data submitted to WHO/UN IGME version 2020 data obtained by active search version 2020	0
	Guinea 2018 Demographic and Health Survey 2018	0
Guinea-Bissau	Kaestrel 2005	1
	Waiswa	1
Guyana	Demographic and Health Survey 2009	1
Honduras	Encuesta Nacional de Demografía y Salud 2005-06	1
	HMIS-DHIS2 data submitted to WHO/UN IGME version 2020	1
	Encuesta Nacional de Demografía y Salud 2011-12	1
Hungary	Vital Registration data submitted to WHO/UN IGME version 2015	1
Iceland	Birth or Death Registry	1
India	Baqui 2006	1
	National Family Health Survey 2005-06	0
	Gupta 2006	1
	McClure 2007	0
	Prost 2013	1
	More 2012	0
	McClure 2011	0
	Bhandari 2012	0
	Kodkany 2015	0
	Vital Registration data submitted to WHO/UN IGME version 2020	0
	HMIS-DHIS2 data submitted to WHO/UN IGME version 2014	0
	Goudar 2013	0
	Tripathy 2016	0
	Global Network Re-analysed	1
	McClure 2015	0
	Spector 2012	0
	Saleem 2014	0
	Waiswa	0
	Altijani 2018	0
	Dandona 2017	1
	Reddy 2017	0
	National Family Health Survey 2015-16	0
	AMANHI 2018	1
	McClure 2018	0
	Dhaded 2018	0
Indonesia	Demographic and Health Survey 2002-03	0
	Sunawang 2009	1
	Shankar 2008	1
	Demographic and Health Survey 2007	1
	Burke 2011	0
	Demographic and Health Survey (Special) 2012	1
	HMIS-DHIS2 data submitted to WHO/UN IGME version 2020	0
	Demographic and Health Survey 2017	1
Iran (Islamic Republic of)	Alizadeh 2015	0
	Hadavi 2011	0
Iraq	Vital Registration data submitted to WHO/UN IGME version 2019	0
Ireland	Birth or Death Registry	1

	Vital Registration data submitted to WHO/UN IGME version NA data submitted to WHO/UN IGME version 2019	0
Israel	Vital Registration data submitted to WHO/UN IGME version 2014	1
Italy	Vital Registration data submitted to WHO/UN IGME version 2015	1
Jamaica	Vital Registration data submitted to WHO/UN IGME version 2020	0
	HMIS-DHIS2 data submitted to WHO/UN IGME version 2015	1
	Reproductive and Health Survey 2008-09	1
Japan	Vital Registration data submitted to WHO/UN IGME version 2020	1
Jordan	Population and Family Health Survey 2002	1
	Population and Family Health Survey 2007	0
	Population and Family Health Survey 2009	1
	Population and Family Health Survey 2012	0
	Population and Family Health Survey 2017-18	0
Kazakhstan	Vital Registration data submitted to WHO/UN IGME version 2014	1
Kenya	Demographic and Health Survey 2003	0
	Demographic and Health Survey 2008-09	0
	McClure 2011	0
	HMIS-DHIS2 data submitted to WHO/UN IGME version 2020	1
	Global Network Re-analysed	1
	McClure 2015	0
	Saleem 2014	0
	Waiswa	0
	Demographic and Health Survey 2014	0
	Creanga 2016	0
	AMANHI 2018	0
	McClure 2018	0
	Child Health and Mortality Prevention Surveillance Network (CHAMPS) Program	0
Kuwait	Vital Registration data submitted to WHO/UN IGME version 2020	0
Kyrgyzstan	Vital Registration data submitted to WHO/UN IGME version 2020	1
	Demographic and Health Survey 2012	1
	Birth or Death Registry	1
Lao People's Democratic Republic	The Lao Social Indicator Survey LSIS II 2017	1
Latvia	Birth or Death Registry	1
Lebanon	Birth or Death Registry	0
Lesotho	Demographic and Health Survey 2004	1
	Demographic and Health Survey 2009	0
	Demographic and Health Survey 2014	1
Liberia	Demographic and Health Survey 2013	0
	HMIS-DHIS2 data submitted to WHO/UN IGME version 2019	0
	Moseson 2014	0
Lithuania	Vital Registration data submitted to WHO/UN IGME version 2019	1
Luxembourg	Birth or Death Registry	1
Madagascar	HMIS-DHIS2 data submitted to WHO/UN IGME version 2020	0
	Enquête démographique et de santé 2003-04	0
	Enquête démographique et de santé 2008-09	1
Malawi	Demographic and Health Survey 2004	1
	Prost 2013	0
	Demographic and Health Survey 2010	0
	Colbourn 2013	1
	HMIS-DHIS2 data submitted to WHO/UN IGME version 2019	1
	Waiswa	1
	Ellard 2016	0
	Demographic and Health Survey 2015-16	0
Malaysia	Vital Registration data submitted to WHO/UN IGME version 2020	1
Maldives	Vital Registration data submitted to WHO/UN IGME version 2014	1
	Demographic and Health Survey 2009	1

	Demographic and Health Survey 2016-17	0
Mali	Enquête démographique et de santé 2006	0
	HMIS-DHIS2 data submitted to WHO/UN IGME version 2020	1
	Enquête sur la prévalence de l'Anémie et de la Parasitémie palustre chez les enfants 2010	0
	Enquête Démographique et de Santé 2012-13	0
	Demographic and Health Survey 2018	0
	Child Health and Mortality Prevention Surveillance Network (CHAMPS) Program	1
Malta	Vital Registration data submitted to WHO/UN IGME version 2020	1
Mauritius	Vital Registration data submitted to WHO/UN IGME version 2019	1
	HMIS-DHIS2 data submitted to WHO/UN IGME version 2018	1
Mexico	Vital Registration data submitted to WHO/UN IGME version 2019	0
	Encuesta Nacional de la Dinámica Demográfica 2018	1
	Encuesta Nacional de la Dinámica Demográfica 2014	1
Republic of Moldova	Vital Registration data submitted to WHO/UN IGME version 2014	0
	Birth or Death Registry	0
Monaco	Vital Registration data submitted to WHO/UN IGME version 2015	0
Mongolia	HMIS-DHIS2 data submitted to WHO/UN IGME version 2018	1
	Multiple Indicator Cluster Survey 2013-14	1
Montenegro	Vital Registration data submitted to WHO/UN IGME version 2019	1
Morocco	HMIS-DHIS2 data submitted to WHO/UN IGME version 2014	0
	Enquête sur la population et la santé familiale 2003-04	1
	Enquête sur la population et la santé familiale 2011	1
	Enquête sur la population et la santé familiale 2018	1
Mozambique	Inquérito Demográfico e de Saúde 2003	1
	Demographic and Health Survey 2011	0
	Sacoor 2018	1
	Arnaldo 2018	0
	HMIS-DHIS2 data submitted to WHO/UN IGME version 2019	0
	Child Health and Mortality Prevention Surveillance Network (CHAMPS) Program	1
Myanmar	Vital Registration data submitted to WHO/UN IGME version 2020	0
	Demographic and Health Survey 2015-16	0
Namibia	Demographic and Health Survey 2006-07	0
	Demographic and Health Survey 2013	0
Nauru	Birth or Death Registry	0
Nepal	Prost 2013	0
	Osrin 2005	0
	Manandhar 2004	1
	Demographic and Health Survey 2006	1
	Demographic and Health Survey 2011	0
	Lee 2011	1
	Demographic and Health Survey 2016	1
	Steinhoff 2017	0
	Kozuki 2017	0
Netherlands	Birth or Death Registry	1
	Vital Registration data submitted to WHO/UN IGME version 2019	0
New Zealand	Vital Registration data submitted to WHO/UN IGME version 2014	1
Nicaragua	Encuesta Nicaragüense de Demografía y Salud 2006	1
	Demographic and Health Survey 2011-12	1
Niger	HMIS-DHIS2 data submitted to WHO/UN IGME version 2014	0
	Enquête démographique et de santé et à indicateurs multiples 2006	0
	Zagre 2007	0
	Enquête démographique et de santé et à indicateurs multiples 2012	1
Nigeria	Demographic and Health Survey 2008	0
	Demographic and Health Survey 2013	0
	Demographic and Health Survey 2018	0
Norway	Vital Registration data submitted to WHO/UN IGME version 2020	1
Oman	Birth or Death Registry	0

	Vital Registration data submitted to WHO/UN IGME version 2020	1
Pakistan	Memon 2015	0
	Bhutta 2008	0
	Demographic and Health Survey 2006-07	1
	Jehan 2007	0
	Demographic and Health Survey 2012-13	1
	Bhutta 2009	1
	McClure 2007	0
	McClure 2011	0
	Bhutta 2011	0
	Global Network Re-analysed	1
	Saleem 2018	0
	Saleem 2014	0
	HMIS-DHIS2 data submitted to WHO/UN IGME version 2020 data obtained by active search version 2020	0
	AMANHI 2018	1
	McClure 2018	0
	Demographic and Health Survey 2017-18	1
Panama	Vital Registration data submitted to WHO/UN IGME version 2015	0
Papua New Guinea	HMIS-DHIS2 data submitted to WHO/UN IGME version 2020	0
Paraguay	Vital Registration data submitted to WHO/UN IGME version 2020	0
	Encuesta Nacional de Demografía y Salud Sexual y Reproductiva 2004	1
	Encuesta Nacional de Demografía y Salud Sexual y Reproductiva 2008	1
Peru	Demographic and Health Survey 2004-08	1
	Encuesta Demográfica y de Salud Familiar 2007-08	1
	Gonzales 2007	0
	Encuesta Demográfica y de Salud Familiar 2009	1
	Encuesta Demográfica y de Salud Familiar 2010	1
	Encuesta Demográfica y de Salud Familiar 2011	1
	Encuesta Demográfica y de Salud Familiar 2012	1
	Encuesta Demográfica y de Salud Familiar 2013	1
	Encuesta Demográfica y de Salud Familiar 2014	1
	Demographic and Health Survey (Continuous) 2017	1
	Demographic and Health Survey (Continuous) 2018	1
	Encuesta Demográfica y de Salud Familiar 2019	1
Philippines	Vital Registration data submitted to WHO/UN IGME version 2014	0
	National Demographic and Health Survey 2003	1
	National Demographic and Health Survey 2013	1
	National Demographic and Health Survey 2008	1
	Demographic and Health Survey 2017	1
Poland	Birth or Death Registry	1
	Vital Registration data submitted to WHO/UN IGME version 2019	1
Portugal	Vital Registration data submitted to WHO/UN IGME version 2019	1
Qatar	Vital Registration data submitted to WHO/UN IGME version 2015	1
Republic of Korea	Vital Registration data submitted to WHO/UN IGME version 2020	1
	Demographic and Health Survey 2005	1
Romania	Vital Registration data submitted to WHO/UN IGME version 2020	1
Russian Federation	Vital Registration data submitted to WHO/UN IGME version 2020	1
Rwanda	Enquête démographique et de santé 2005	0
	HMIS-DHIS2 data submitted to WHO/UN IGME version 2014	1
	Demographic and Health Survey 2010	1
	Demographic and Health Survey 2014-15	1
Saint Lucia	Vital Registration data submitted to WHO/UN IGME version 2020	1
Saint Vincent and the Grenadines	Vital Registration data submitted to WHO/UN IGME version 2020	1
San Marino	Vital Registration data submitted to WHO/UN IGME version 2015	1
Sao Tome and Principe	Demographic and Health Survey 2008-09	0

Saudi Arabia	HMIS-DHIS2 data submitted to WHO/UN IGME version 2014	0
	Khashoggi 2005	0
Senegal	Enquête démographique et de santé 2005	1
	HMIS-DHIS2 data submitted to WHO/UN IGME version 2014	1
	Enquête démographique et de santé à indicateurs multiples 2010-11	1
	Enquête Démographique et de Santé Continue 2012-13	1
	Enquête Démographique et de Santé Continue 2014	1
	Enquête Démographique et de Santé Continue 2015	1
	Enquête Démographique et de Santé Continue 2016	1
	Enquête Démographique et de Santé Continue 2017	1
	Demographic Health Survey 2018	1
Serbia	Vital Registration data submitted to WHO/UN IGME version 2019	1
Seychelles	Vital Registration data submitted to WHO/UN IGME version 2014	1
Sierra Leone	Demographic and Health Survey 2008	0
	Demographic and Health Survey 2013	0
	Demographic and Health Survey 2019	0
Singapore	Vital Registration data submitted to WHO/UN IGME version 2020	1
Slovakia	Vital Registration data submitted to WHO/UN IGME version 2019	1
Slovenia	Birth or Death Registry	1
	Vital Registration data submitted to WHO/UN IGME version NA data submitted to WHO/UN IGME version 2019	0
South Africa	Vital Registration data submitted to WHO/UN IGME version 2014	0
	HMIS-DHIS2 data submitted to WHO/UN IGME version 2014	1
	Waiswa	1
	Lavin 2018	0
	Demographic and Health Survey 2016	0
Spain	Vital Registration data submitted to WHO/UN IGME version 2015	1
Sri Lanka	Vital Registration data submitted to WHO/UN IGME version 2014	0
	Demographic and Health Survey 2006-07	1
	HMIS-DHIS2 data submitted to WHO/UN IGME version 2019	1
State of Palestine	HMIS-DHIS2 data submitted to WHO/UN IGME version 2014	0
Sudan	HMIS-DHIS2 data submitted to WHO/UN IGME version 2014	0
	Ali 2014	1
Suriname	Vital Registration data submitted to WHO/UN IGME version 2014	0
	HMIS-DHIS2 data submitted to WHO/UN IGME version 2014	0
Sweden	Birth or Death Registry	1
	Vital Registration data submitted to WHO/UN IGME version NA data submitted to WHO/UN IGME version 2019	0
Switzerland	Vital Registration data submitted to WHO/UN IGME version 2018	1
Tajikistan	Demographic and Health Survey 2017	0
	Demographic and Health Survey 2012	0
	Vital Registration data submitted to WHO/UN IGME version 2020	0
United Republic of Tanzania	Kilonzo 2001	1
	HMIS-DHIS2 data submitted to WHO/UN IGME version 2020 data obtained by active search version 2020	0
	Waiswa	1
	Mosha 2014	1
	AMANHI 2018	1
Northern Macedonia	Vital Registration data submitted to WHO/UN IGME version 2014	1
Thailand	Mo-suwan 2009	0
Timor-Leste	Demographic and Health Survey 2009-10	0
	Demographic and Health Survey 2016	0
	HMIS-DHIS2 data submitted to WHO/UN IGME version 2020	0
Togo	HMIS-DHIS2 data submitted to WHO/UN IGME version 2014	0
Trinidad and Tobago	Vital Registration data submitted to WHO/UN IGME version 2019	0
	HMIS-DHIS2 data submitted to WHO/UN IGME version 2018 data obtained by active search version 2018	1
Tunisia	Vital Registration data submitted to WHO/UN IGME version 2015	0

	HMIS-DHIS2 data submitted to WHO/UN IGME version 2020	1
Turkey	Demographic and Health Survey 2003	1
	Demographic and Health Survey 2008	1
	Arslan 2013	0
	Vital Registration data submitted to WHO/UN IGME version 2019	1
	HMIS-DHIS2 data submitted to WHO/UN IGME version 2018	1
	Demographic and Health Survey 2013	1
Turkmenistan	Vital Registration data submitted to WHO/UN IGME version 2014	0
United Arab Emirates	Vital Registration data submitted to WHO/UN IGME version 2020	1
Uganda	Kujala 2017	1
	Asiki 2015	1
	Demographic and Health Survey 2006	1
	Ndyomugyeni 2011	1
	Nankabirwa 2011	1
	Demographic and Health Survey 2011	1
	HMIS-DHIS2 data submitted to WHO/UN IGME version 2020	1
	Kananura 2017	0
	Uganda Demographic and Health Survey 2016	1
Ukraine	Vital Registration data submitted to WHO/UN IGME version 2020	1
	Demographic and Health Survey 2007	1
	Multiple Indicator Cluster Survey 2012	0
United Kingdom	Vital Registration data submitted to WHO/UN IGME version 2019	1
	Demographic and Health Survey 2004-05	1
	Demographic and Health Survey 2010	1
	Demographic and Health Survey 2015-16	1
United States of America	Vital Registration data submitted to WHO/UN IGME version 2014	1
Uruguay	Vital Registration data submitted to WHO/UN IGME version 2020	1
Uzbekistan	Vital Registration data submitted to WHO/UN IGME version 2014	0
Venezuela (Bolivarian Republic of)	Vital Registration data submitted to WHO/UN IGME version 2015	0
Viet Nam	Demographic and Health Survey 2002	0
	Graner 2009	0
	Persson 2013	1
Yemen	National Health and Demographic Survey 2013	1
	Al-Shahethi 2018	1
Zambia	Demographic and Health Survey 2007	0
	McClure 2007	0
	Stringer 2011	0
	McClure 2011	0
	Gill 2011	1
	Turnbull 2011	1
	Chi 2011	1
	Stringer 2015	0
	Global Network Re-analysed	1
	McClure 2015	0
	Saleem 2014	0
	Demographic and Health Survey 2013-14	1
	AMANHI 2018	1
	McClure 2018	0
	Demographic and Health Survey 2018	0
Zimbabwe	Demographic and Health Survey 2005-06	0
	HMIS-DHIS2 data submitted to WHO/UN IGME version 2020	1
	Demographic and Health Survey 2010-11	0
	Tachiweyika 2011	1
	Demographic and Health Survey 2015	0

Table A9: Data availability and inclusion by country and data source type

		Total Datapoints		Administrative datapoints		HMIS datapoints		Household survey datapoints		Population based study datapoints	
Country Name	Included quality data category	All	Include d quality data in model	All	Include d quality data in model	Al l	Include d quality data in model	Al l	Include d quality data in model	Al l	Include d quality data in model
Albania	No Data	11	0	0	0	4	0	7	0	0	0
Algeria	>=5 to 10	10	8	7	7	1	1	2	0	0	0
Andorra	>=10	19	18	19	18	0	0	0	0	0	0
Angola	>=10	19	19	19	19	0	0	0	0	0	0
Antigua and Barbuda	No Data	1	0	0	0	0	0	1	0	0	0
Argentina	No Data	0	0	0	0	0	0	0	0	0	0
Armenia	>=10	19	19	19	19	0	0	0	0	0	0
Australia	>=5 to 10	28	9	19	9	0	0	9	0	0	0
Austria	>=10	21	13	21	13	0	0	0	0	0	0
Azerbaijan	>=10	21	19	21	19	0	0	0	0	0	0
Bahamas	<5	18	1	16	0	0	0	2	1	0	0
Bahrain	>=10	19	19	19	19	0	0	0	0	0	0
Bangladesh	>=10	20	20	0	0	20	20	0	0	0	0
Barbados	>=10	40	22	2	0	9	0	4	4	25	18
Belarus	No Data	0	0	0	0	0	0	0	0	0	0
Belgium	>=10	19	13	19	13	0	0	0	0	0	0
Belize	>=10	19	17	19	17	0	0	0	0	0	0
Benin	>=10	12	11	12	11	0	0	0	0	0	0
Bhutan	>=5 to 10	11	7	0	0	8	7	3	0	0	0
Bolivia (Plurinational State of)	<5	1	1	0	0	1	1	0	0	0	0
Bosnia and Herzegovina	<5	3	1	0	0	0	0	3	1	0	0
Botswana	>=10	15	14	14	14	0	0	0	0	1	0
Brazil	>=5 to 10	13	7	7	1	6	6	0	0	0	0
Brunei Darussalam	>=10	12 8	17	18	0	36	17	0	0	74	0
Bulgaria	>=10	19	19	19	19	0	0	0	0	0	0
Burkina Faso	>=10	20	20	20	20	0	0	0	0	0	0
Burundi	>=5 to 10	13	9	0	0	10	9	2	0	1	0
Cambodia	<5	5	4	0	0	3	2	2	2	0	0
Cameroon	No Data	2	0	0	0	0	0	2	0	0	0
Canada	No Data	1	0	0	0	0	0	1	0	0	0
Cabo Verde	>=10	19	19	19	19	0	0	0	0	0	0
Central African Republic	<5	2	1	1	0	0	0	1	1	0	0
Chad	No Data	0	0	0	0	0	0	0	0	0	0
Chile	No Data	1	0	0	0	1	0	0	0	0	0
China	>=10	17	11	17	11	0	0	0	0	0	0
Colombia	>=10	23	11	8	8	0	0	0	0	15	3
Comoros	<5	23	2	20	0	0	0	3	2	0	0
Congo	No Data	1	0	0	0	0	0	1	0	0	0
Democratic Republic of the Congo	No Data	4	0	0	0	4	0	0	0	0	0
Cook Islands	>=5 to 10	10	6	0	0	0	0	0	0	10	6
Costa Rica	>=10	17	17	17	17	0	0	0	0	0	0
Cote d'Ivoire	>=10	19	19	19	19	0	0	0	0	0	0

Croatia	No Data	1	0	0	0	0	0	0	0	1	0
Cuba	>=10	20	18	20	18	0	0	0	0	0	0
Cyprus	>=10	20	20	20	20	0	0	0	0	0	0
Czechia	>=5 to 10	13	7	13	7	0	0	0	0	0	0
Denmark	>=10	20	20	20	20	0	0	0	0	0	0
Djibouti	>=10	18	17	18	17	0	0	0	0	0	0
Dominica	<5	2	1	0	0	2	1	0	0	0	0
Dominican Republic	No Data	0	0	0	0	0	0	0	0	0	0
Ecuador	No Data	8	0	0	0	7	0	1	0	0	0
Egypt	<5	24	2	18	0	0	0	6	2	0	0
El Salvador	No Data	23	0	0	0	18	0	5	0	0	0
Equatorial Guinea	<5	17	2	0	0	14	0	3	2	0	0
Eritrea	No Data	1	0	0	0	0	0	1	0	0	0
Estonia	No Data	0	0	0	0	0	0	0	0	0	0
Ethiopia	>=10	20	20	20	20	0	0	0	0	0	0
Micronesia (Federated States of)	No Data	21	0	0	0	6	0	3	0	12	0
Fiji	No Data	0	0	0	0	0	0	0	0	0	0
Finland	>=10	11	11	0	0	11	11	0	0	0	0
France	>=10	19	19	19	19	0	0	0	0	0	0
Gabon	>=5 to 10	16	7	16	7	0	0	0	0	0	0
Gambia	No Data	0	0	0	0	0	0	0	0	0	0
Georgia	<5	11	1	0	0	9	0	1	0	1	1
Germany	>=10	28	12	27	11	0	0	1	1	0	0
Ghana	>=10	21	18	21	18	0	0	0	0	0	0
Greece	>=5 to 10	17	5	0	0	5	0	7	2	5	3
Grenada	>=10	19	19	19	19	0	0	0	0	0	0
Guatemala	No Data	3	0	3	0	0	0	0	0	0	0
Guinea	>=10	40	12	18	0	0	0	3	3	19	9
Guinea-Bissau	No Data	12	0	0	0	10	0	2	0	0	0
Guyana	<5	2	2	0	0	0	0	0	0	2	2
Haiti	<5	1	1	0	0	0	0	1	1	0	0
Honduras	No Data	0	0	0	0	0	0	0	0	0	0
Hungary	>=5 to 10	13	6	0	0	11	4	2	2	0	0
Iceland	>=10	17	16	17	16	0	0	0	0	0	0
India	>=10	20	20	20	20	0	0	0	0	0	0
Indonesia	>=10	89	20	10	0	11	0	2	0	66	20
Iran (Islamic Republic of)	>=5 to 10	13	5	0	0	6	0	4	3	3	2
Iraq	No Data	3	0	0	0	0	0	0	0	3	0
Ireland	No Data	18	0	18	0	0	0	0	0	0	0
Israel	>=10	20	18	20	18	0	0	0	0	0	0
Italy	>=10	17	17	17	17	0	0	0	0	0	0
Jamaica	>=10	18	18	18	18	0	0	0	0	0	0
Japan	>=10	29	10	18	0	10	9	1	1	0	0
Jordan	>=10	19	19	19	19	0	0	0	0	0	0
Kazakhstan	<5	5	2	0	0	0	0	5	2	0	0
Kenya	>=5 to 10	16	8	16	8	0	0	0	0	0	0
Kiribati	>=5 to 10	36	9	0	0	11	2	3	0	22	7
Democratic People's Republic of Korea	No Data	0	0	0	0	0	0	0	0	0	0
Republic of Korea	No Data	0	0	0	0	0	0	0	0	0	0
Kuwait	>=10	10	10	10	10	0	0	0	0	0	0
Kyrgyzstan	No Data	19	0	19	0	0	0	0	0	0	0
Lao People's Democratic Republic	>=5 to 10	26	7	23	6	0	0	3	1	0	0
Latvia	<5	1	1	0	0	0	0	1	1	0	0
Lebanon	>=10	19	19	19	19	0	0	0	0	0	0

Lesotho	No Data	7	0	7	0	0	0	0	0	0	0
Liberia	<5	3	2	0	0	0	0	3	2	0	0
Libya	No Data	10	0	0	0	8	0	1	0	1	0
Lithuania	No Data	0	0	0	0	0	0	0	0	0	0
Luxembourg	>=5 to 10	19	9	19	9	0	0	0	0	0	0
Northern Macedonia	>=10	17	16	17	16	0	0	0	0	0	0
Madagascar	>=10	19	19	19	19	0	0	0	0	0	0
Malawi	<5	21	1	0	0	19	0	2	1	0	0
Malaysia	>=5 to 10	17	8	0	0	8	5	3	1	6	2
Maldives	>=10	19	11	19	11	0	0	0	0	0	0
Mali	>=10	17	10	15	9	0	0	2	1	0	0
Malta	>=5 to 10	21	5	0	0	13	3	4	0	4	2
Marshall Islands	>=10	19	19	19	19	0	0	0	0	0	0
Mauritania	No Data	0	0	0	0	0	0	0	0	0	0
Mauritius	No Data	0	0	0	0	0	0	0	0	0	0
Mexico	>=10	26	26	20	20	6	6	0	0	0	0
Republic of Moldova	<5	26	2	19	0	0	0	7	2	0	0
Monaco	<5	21	1	19	0	0	0	2	1	0	0
Mongolia	No Data	3	0	3	0	0	0	0	0	0	0
Montenegro	>=10	16	13	0	0	15	12	1	1	0	0
Morocco	>=10	19	18	19	18	0	0	0	0	0	0
Mozambique	<5	20	3	0	0	17	0	3	3	0	0
Myanmar	<5	8	4	0	0	2	0	2	1	4	3
Namibia	No Data	6	0	5	0	0	0	1	0	0	0
Nauru	No Data	2	0	0	0	0	0	2	0	0	0
Nepal	No Data	5	0	5	0	0	0	0	0	0	0
Netherlands	<5	15	4	0	0	0	0	9	2	6	2
New Zealand	>=10	32	16	32	16	0	0	0	0	0	0
Nicaragua	>=10	16	16	16	16	0	0	0	0	0	0
Niger	<5	3	2	0	0	0	0	3	2	0	0
Nigeria	<5	15	1	0	0	12	0	2	1	1	0
Niue	No Data	3	0	0	0	0	0	3	0	0	0
Norway	No Data	0	0	0	0	0	0	0	0	0	0
Oman	>=10	19	19	19	19	0	0	0	0	0	0
Pakistan	>=10	13	12	13	12	0	0	0	0	0	0
Palau	>=10	38	15	0	0	6	0	5	3	27	12
Panama	No Data	0	0	0	0	0	0	0	0	0	0
Papua New Guinea	No Data	19	0	19	0	0	0	0	0	0	0
Paraguay	No Data	10	0	0	0	10	0	0	0	0	0
Peru	<5	20	2	18	0	0	0	2	2	0	0
Philippines	>=10	12	11	0	0	0	0	11	11	1	0
Poland	<5	25	4	15	0	0	0	10	4	0	0
Portugal	>=10	22	20	22	20	0	0	0	0	0	0
Qatar	>=10	20	20	20	20	0	0	0	0	0	0
Romania	>=10	12	12	12	12	0	0	0	0	0	0
Russian Federation	>=10	19	14	19	14	0	0	0	0	0	0
Rwanda	>=10	22	20	22	20	0	0	0	0	0	0
Saint Kitts and Nevis	>=5 to 10	12	8	0	0	9	6	3	2	0	0
Saint Lucia	No Data	0	0	0	0	0	0	0	0	0	0
Samoa	>=10	20	17	20	17	0	0	0	0	0	0
San Marino	No Data	0	0	0	0	0	0	0	0	0	0
Sao Tome and Principe	>=5 to 10	7	7	7	7	0	0	0	0	0	0
Saudi Arabia	No Data	1	0	0	0	0	0	1	0	0	0
Senegal	No Data	16	0	0	0	15	0	0	0	1	0
Serbia	>=10	24	12	0	0	16	4	8	8	0	0
Seychelles	>=10	19	19	19	19	0	0	0	0	0	0
Sierra Leone	>=10	15	15	15	15	0	0	0	0	0	0
Singapore	No Data	3	0	0	0	0	0	3	0	0	0

Slovakia	>=10	19	12	19	12	0	0	0	0	0	0
Slovenia	>=10	21	20	21	20	0	0	0	0	0	0
Solomon Islands	>=10	19	18	19	18	0	0	0	0	0	0
Somalia	No Data	0	0	0	0	0	0	0	0	0	0
South Africa	No Data	0	0	0	0	0	0	0	0	0	0
South Sudan	>=5 to 10	32	6	11	0	16	5	1	0	4	1
Spain	No Data	0	0	0	0	0	0	0	0	0	0
Sri Lanka	>=10	18	17	18	17	0	0	0	0	0	0
Saint Vincent and the Grenadines	>=10	30	14	16	0	13	13	1	1	0	0
State of Palestine	>=10	17	17	17	17	0	0	0	0	0	0
Sudan	No Data	14	0	0	0	14	0	0	0	0	0
Suriname	<5	11	1	0	0	10	0	0	0	1	1
Eswatini	No Data	9	0	9	0	0	0	0	0	0	0
Sweden	<5	6	2	0	0	3	0	3	2	0	0
Switzerland	>=10	19	18	19	18	0	0	0	0	0	0
Syrian Arab Republic	>=10	17	17	17	17	0	0	0	0	0	0
Tajikistan	No Data	0	0	0	0	0	0	0	0	0	0
United Republic of Tanzania	No Data	26	0	19	0	0	0	7	0	0	0
Thailand	>=5 to 10	13	8	0	0	5	0	3	3	5	5
Timor-Leste	No Data	1	0	0	0	0	0	0	0	1	0
Togo	No Data	14	0	0	0	12	0	2	0	0	0
Tonga	No Data	9	0	0	0	9	0	0	0	0	0
Trinidad and Tobago	No Data	0	0	0	0	0	0	0	0	0	0
Tunisia	<5	20	3	17	0	3	3	0	0	0	0
Turkey	<5	16	3	11	0	5	3	0	0	0	0
Turkmenistan	>=10	17	16	9	9	4	4	3	3	1	0
Tuvalu	No Data	18	0	18	0	0	0	0	0	0	0
Uganda	No Data	0	0	0	0	0	0	0	0	0	0
Ukraine	>=5 to 10	24	8	0	0	8	1	3	3	13	4
United Arab Emirates	>=10	24	12	20	11	0	0	4	1	0	0
United Kingdom	>=10	18	18	18	18	0	0	0	0	0	0
United States of America	>=10	19	19	19	19	0	0	0	0	0	0
Uruguay	>=10	17	17	17	17	0	0	0	0	0	0
Uzbekistan	>=10	19	17	19	17	0	0	0	0	0	0
Vanuatu	No Data	12	0	12	0	0	0	0	0	0	0
Venezuela (Bolivarian Republic of)	No Data	0	0	0	0	0	0	0	0	0	0
Viet Nam	No Data	13	0	13	0	0	0	0	0	0	0
Yemen	<5	3	1	0	0	0	0	1	0	2	1
Zambia	<5	2	2	0	0	0	0	1	1	1	1
Zimbabwe	>=10	26	12	0	0	0	0	3	1	23	11

Figure A4: Ratio of stillbirth rate and neonatal mortality rate and stillbirth rate in countries, 2019

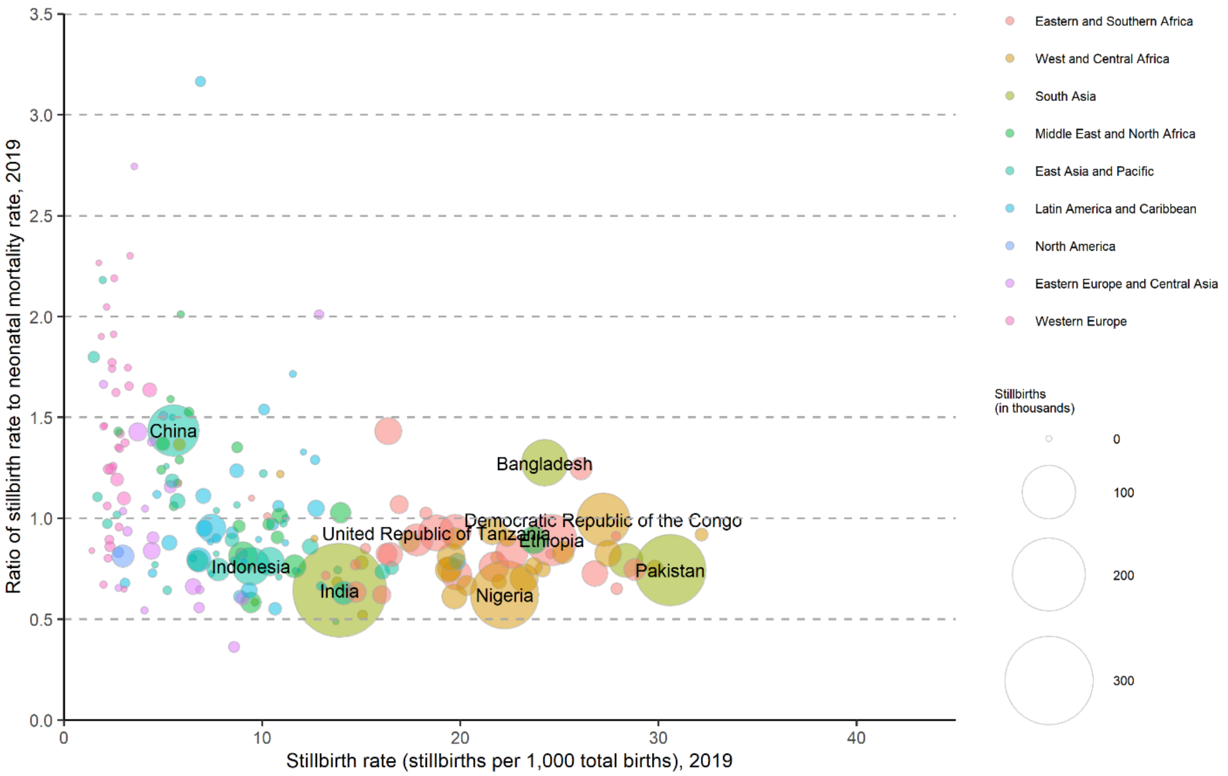

Note: Each bubble represents a country, the size of the bubbles indicates the number of stillbirths.

Figure A5: Ratio of stillbirth rate and neonatal mortality rate and stillbirth rate in countries, 2000-2019

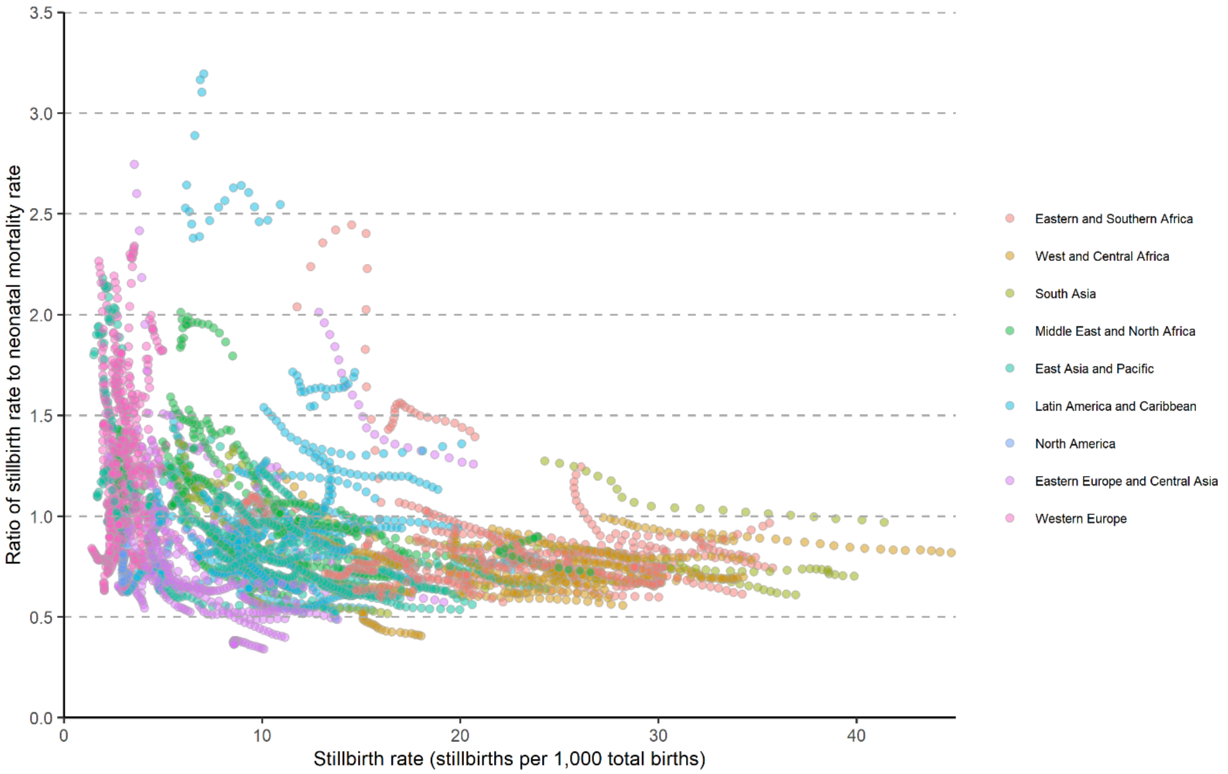

Figure A6: Stillbirth rate estimates with underlying data, by country or territory (pp 47-241)

References

[1] <www.dhis2.org>, accessed 22 September 2020.

[2] Bradley S E K, Winfrey W, Croft T N (2015). Contraceptive Use and Perinatal Mortality in the DHS: An Assessment of the Quality and Consistency of Calendars and Histories. DHS Methodological Reports No. 17. Rockville, Maryland, USA: ICF International.

[3] Blencowe H, et al., 'National, regional, and worldwide estimates of stillbirth rates in 2015, with trends from 2000: a systematic analysis', Lancet Global Health 20164 (2): 98–108.

[4] Wang, Z et al. Estimating the Stillbirth Rate for 195 Countries Using A Bayesian Sparse Regression Model with Temporal Smoothing. https://arxiv.org/abs/2010.03551.(2020) arXiv preprint arXiv:2010.03551 (2020).

[5] United Nations Department of Economic and Social Affairs Population Division, World Population Prospects: The 2019 revision, UN, New York, 2019; available here.

Afghanistan

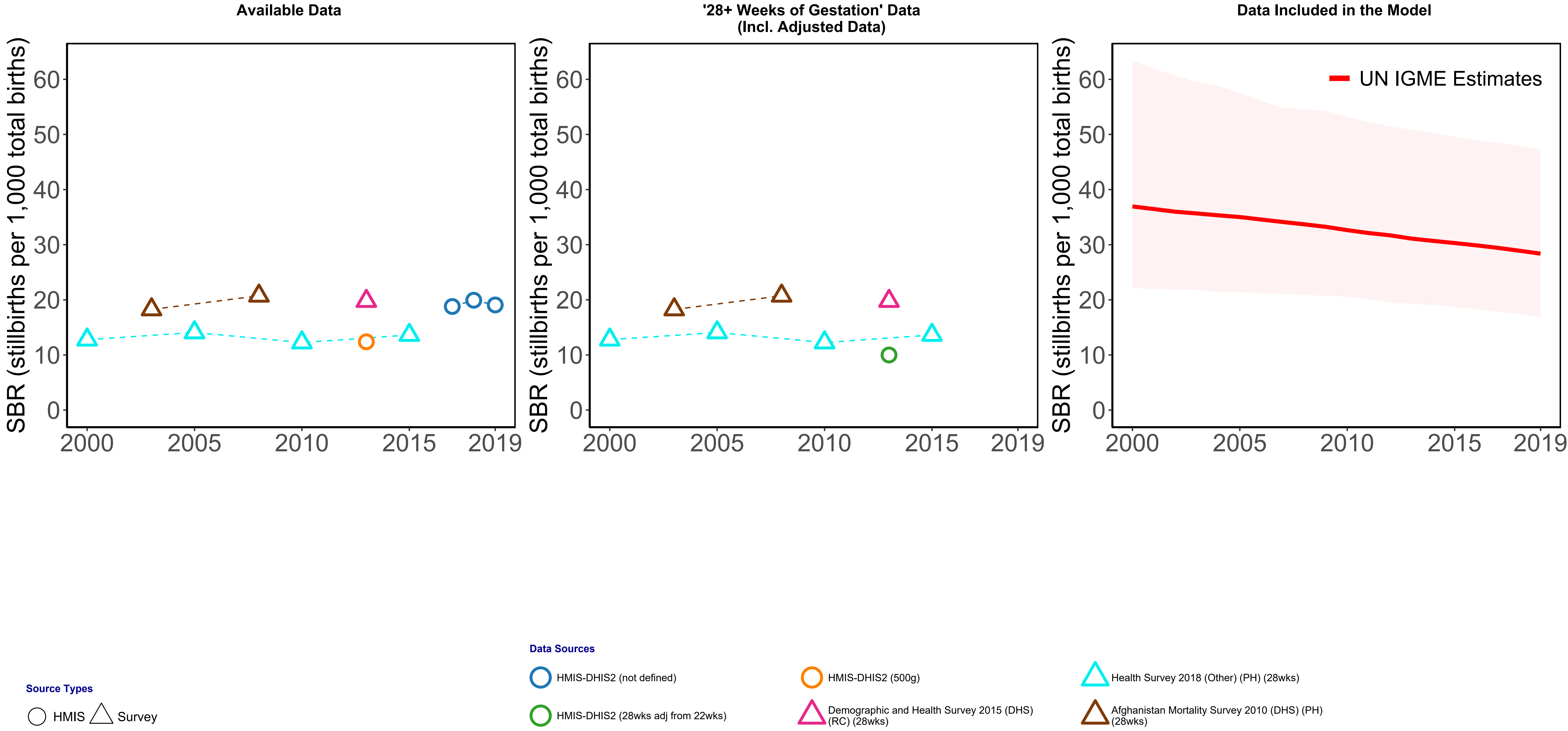

Angola

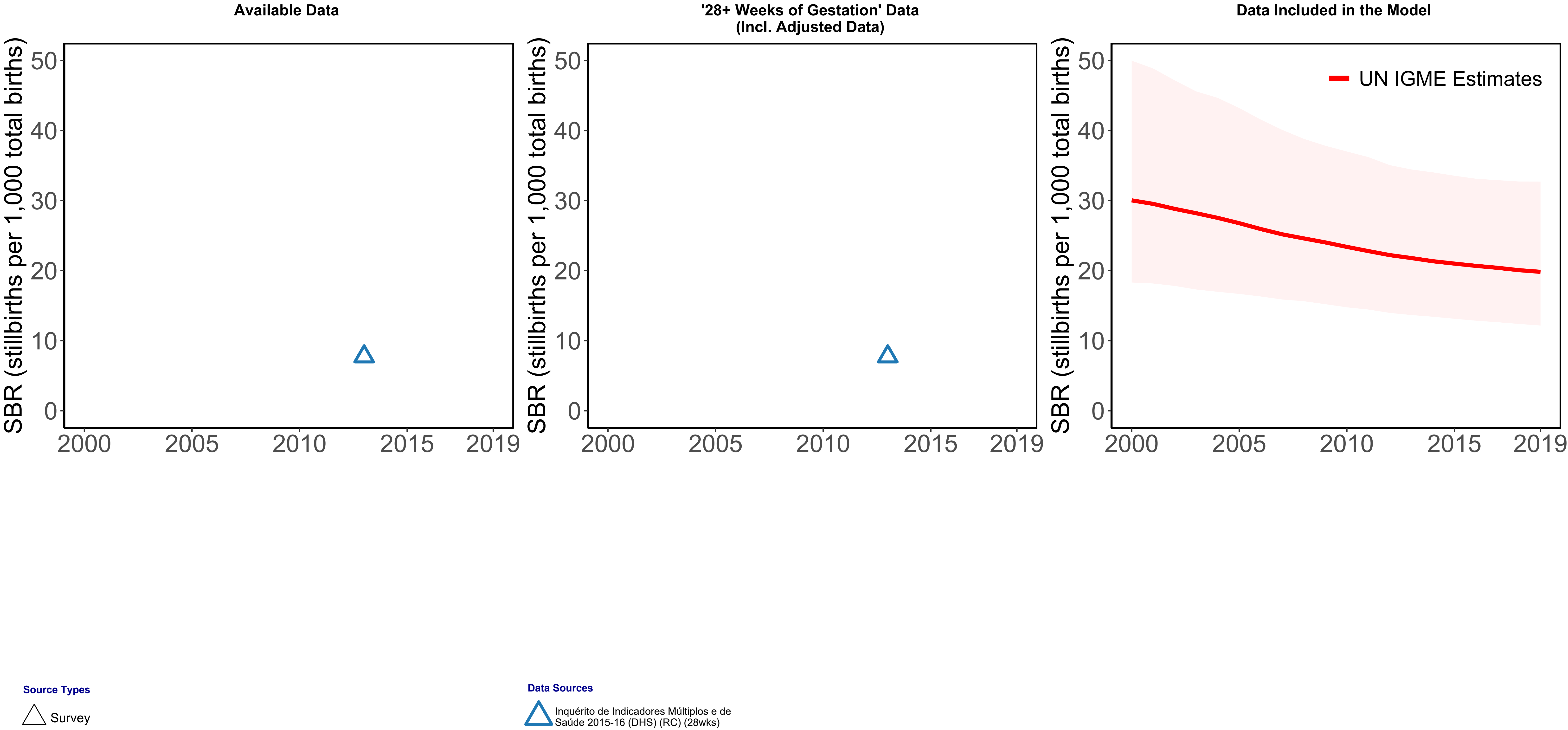

Available Data
'28+ Weeks of Gestation' Data
(Incl. Adjusted Data)
Data Included in the Model
SBR (stillbirths per 1,000 total births)
0
10
20
30
40
50
2000
2005
2010
2015
2019
UN IGME Estimates
Source Types
Survey
Data Sources
Inquérito de Indicadores Múltiplos e de Saúde 2015-16 (DHS) (RC) (28wks)

Albania

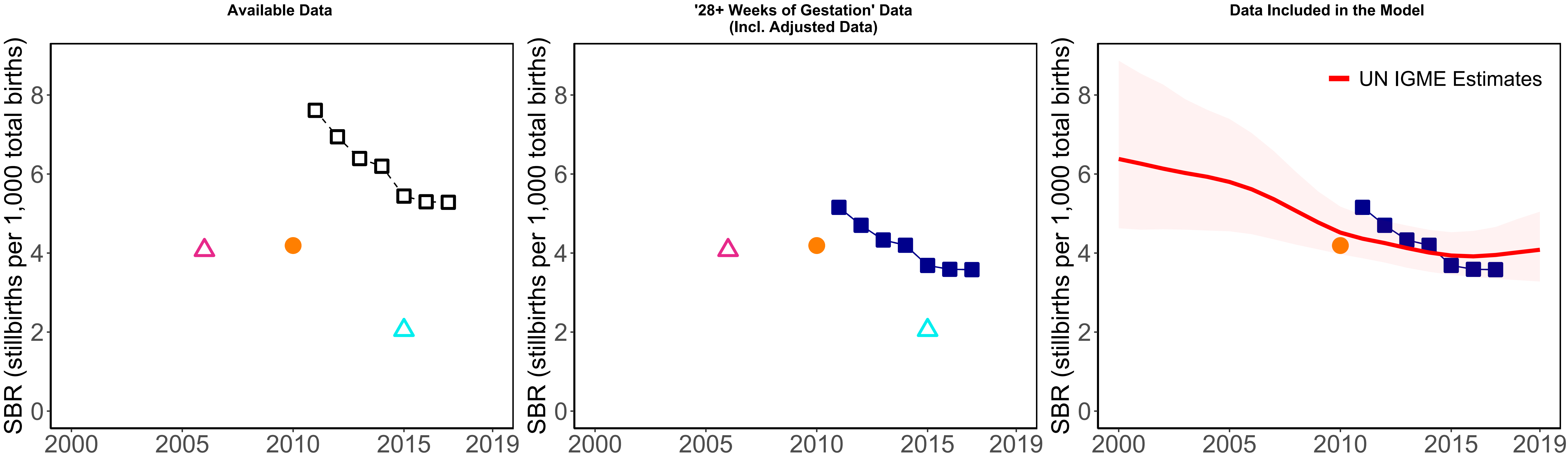

Source Types

Administrative HMIS Survey

Data Sources

Vital Registration (22wks)

HMIS-DHIS2 (28wks)

Demographic and Health Survey 2008-09 (DHS) (RC) (28wks)

Vital Registration (28wks adj from 22wks)

Demographic and Health Survey 2017-18 (DHS) (RC) (28wks)

Andorra

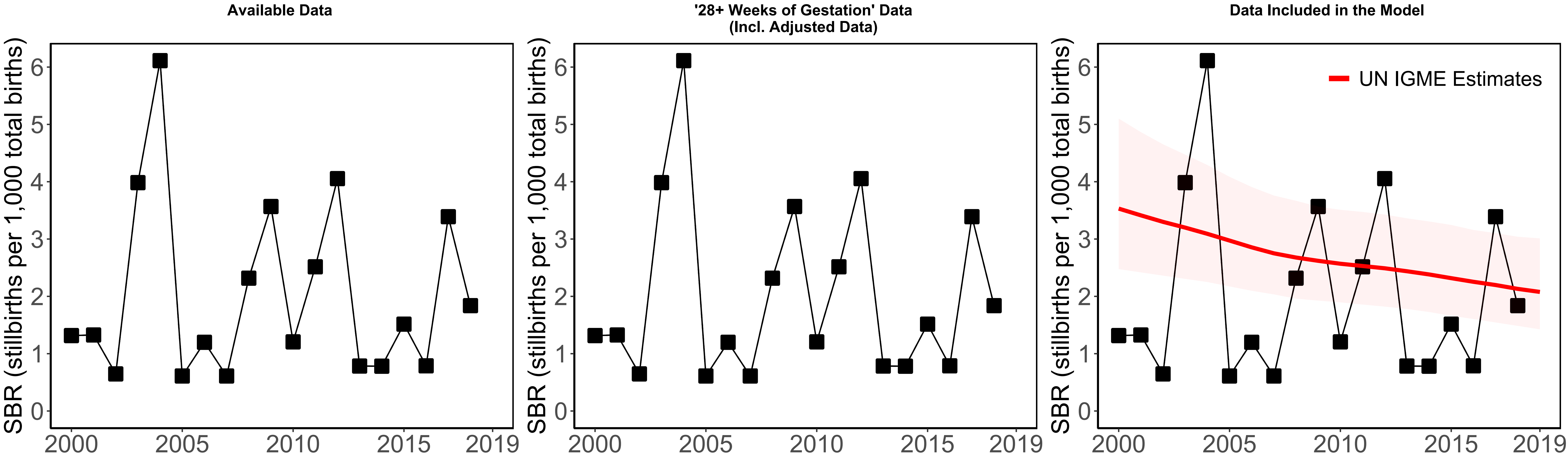

Source Types

Administrative

Data Sources

Birth or Death Registry (28wks)

United Arab Emirates

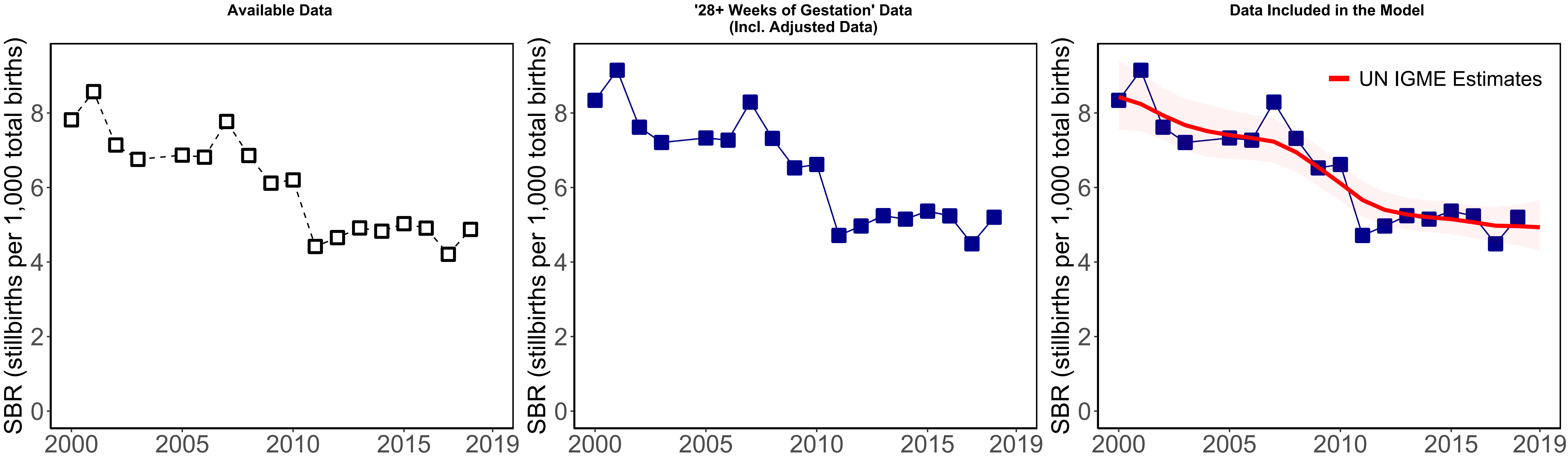

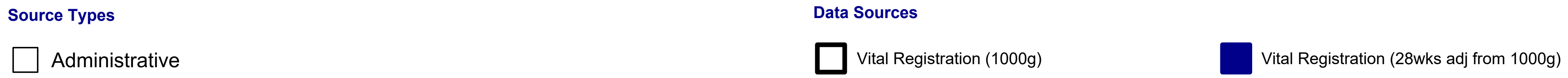

Argentina

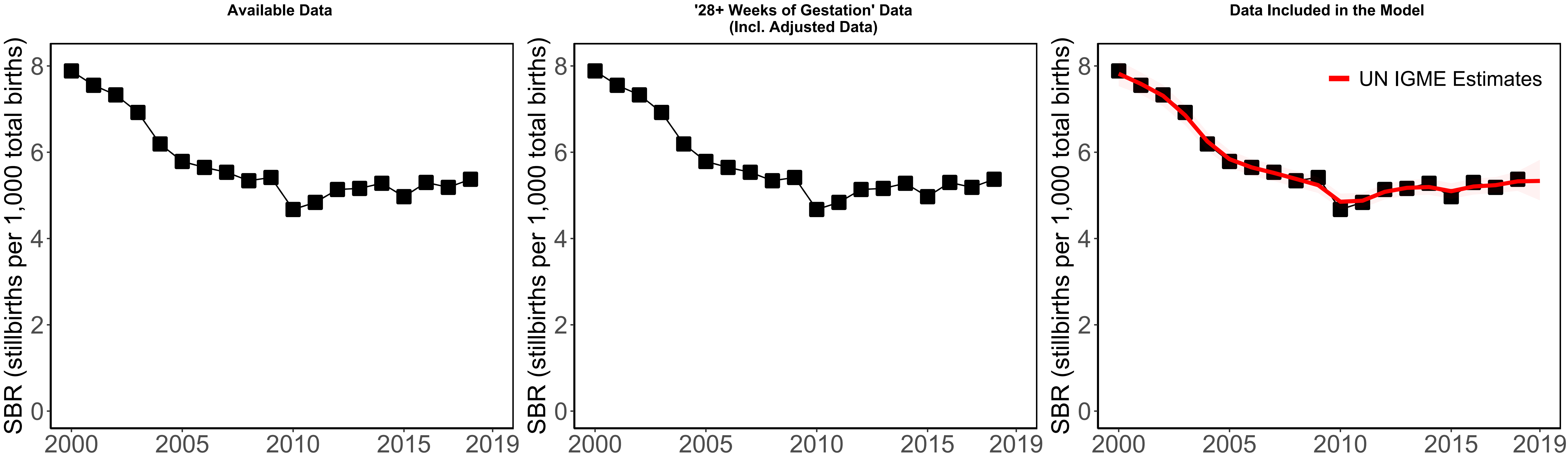

Source Types

Administrative

Data Sources

Vital Registration (28wks)

Armenia

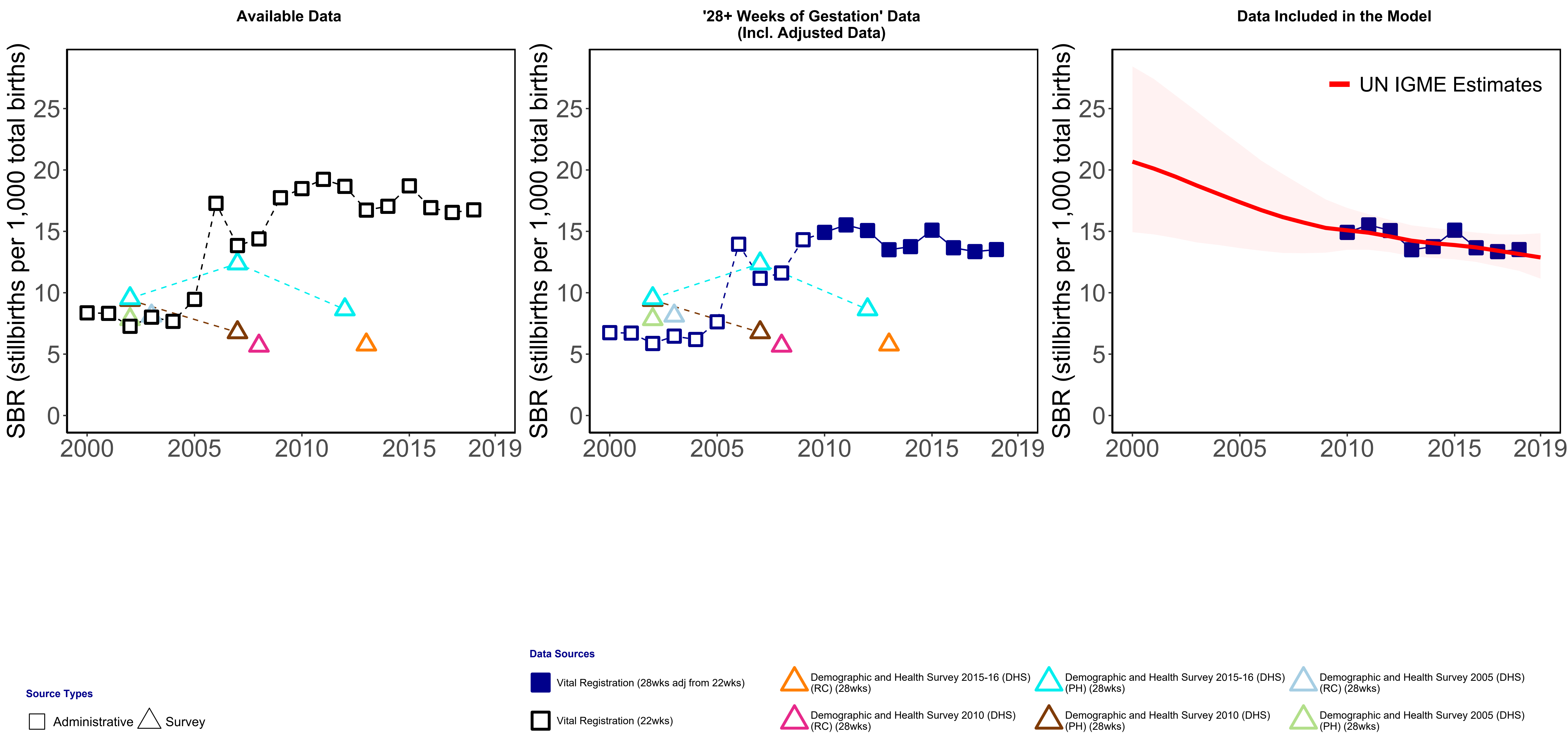
Available Data
'28+ Weeks of Gestation' Data
(Incl. Adjusted Data)
Data Included in the Model
SBR (stillbirths per 1,000 total births)
UN IGME Estimates
0
5
10
15
20
25
2000
2005
2010
2015
2019
Source Types
Administrative
Survey
Data Sources
Vital Registration (28wks adj from 22wks)
Vital Registration (22wks)
Demographic and Health Survey 2015-16 (DHS) (RC) (28wks)
Demographic and Health Survey 2010 (DHS) (RC) (28wks)
Demographic and Health Survey 2015-16 (DHS) (PH) (28wks)
Demographic and Health Survey 2010 (DHS) (PH) (28wks)
Demographic and Health Survey 2005 (DHS) (RC) (28wks)
Demographic and Health Survey 2005 (DHS) (PH) (28wks)

Antigua and Barbuda

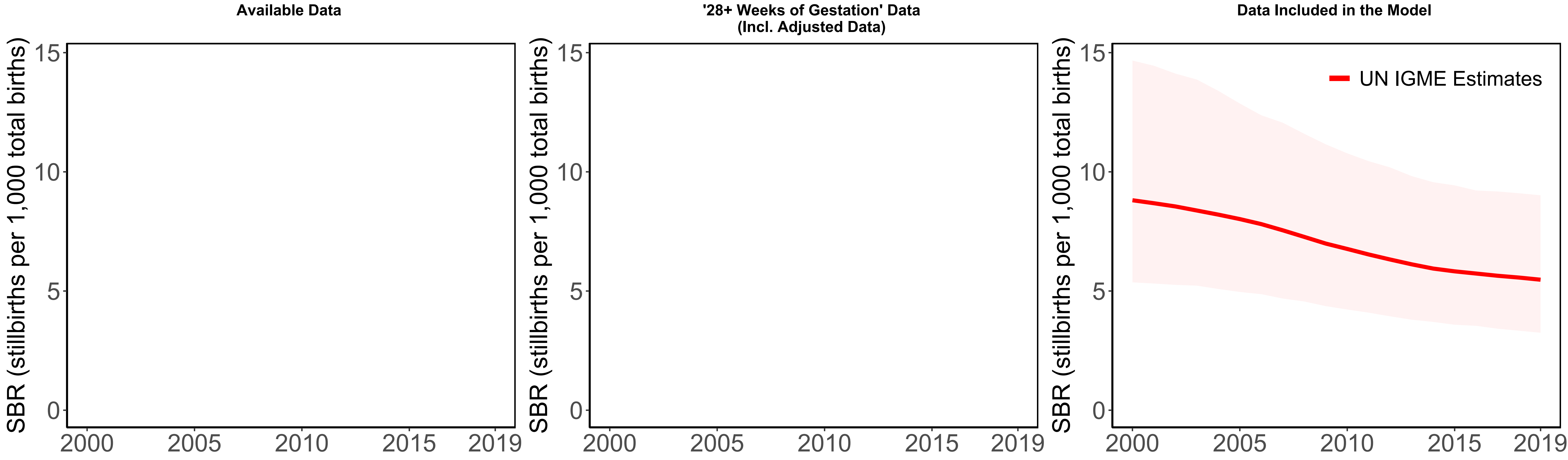

Australia

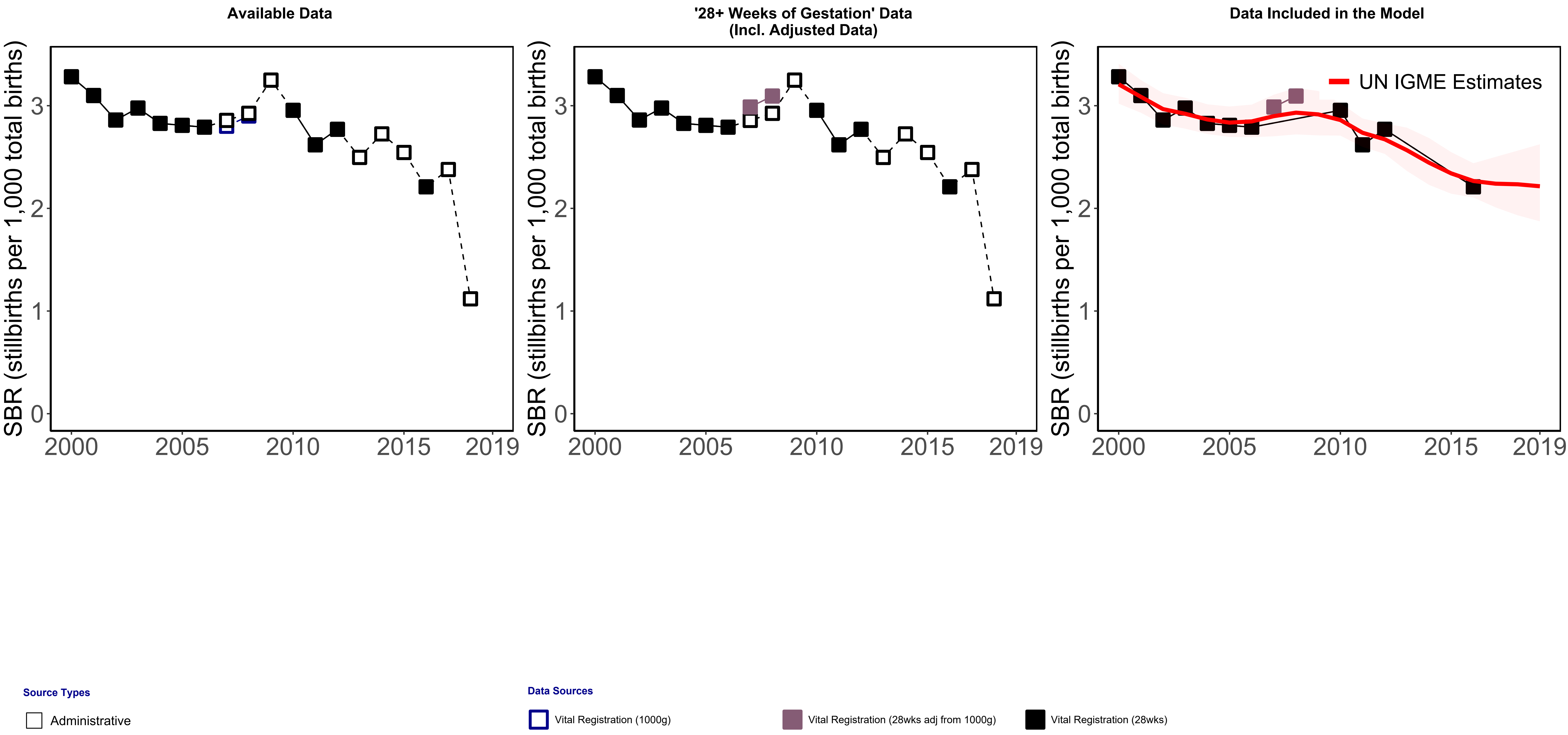

Available Data
'28+ Weeks of Gestation' Data
(Incl. Adjusted Data)
Data Included in the Model
SBR (stillbirths per 1,000 total births)
0
1
2
3
2000
2005
2010
2015
2019
UN IGME Estimates
Source Types
Administrative
Data Sources
Vital Registration (1000g)
Vital Registration (28wks adj from 1000g)
Vital Registration (28wks)

Austria

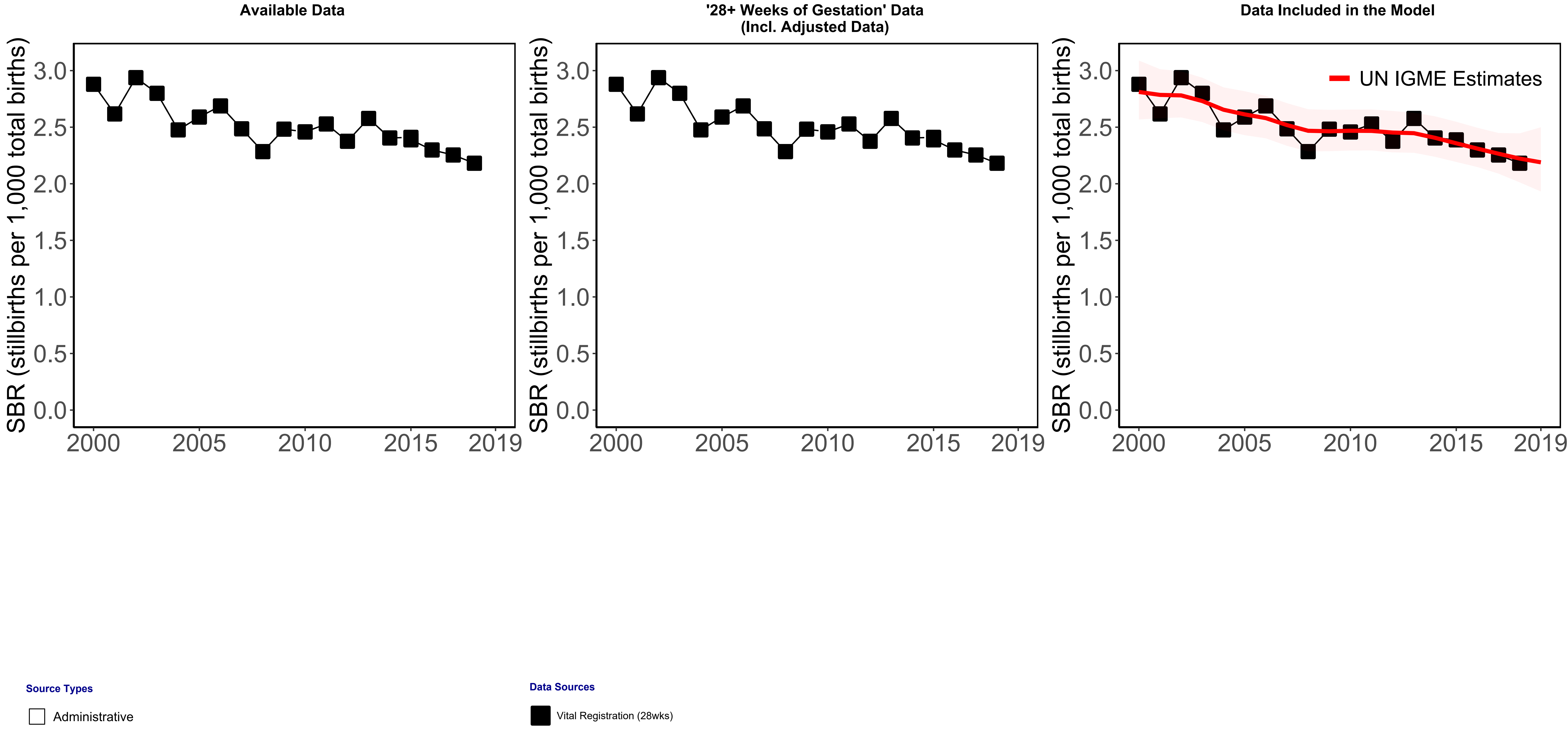

Available Data
'28+ Weeks of Gestation' Data
(Incl. Adjusted Data)
Data Included in the Model
SBR (stillbirths per 1,000 total births)
3.0
2.5
2.0
1.5
1.0
0.5
0.0
2000
2005
2010
2015
2019
UN IGME Estimates
Source Types
Administrative
Data Sources
Vital Registration (28wks)

Azerbaijan

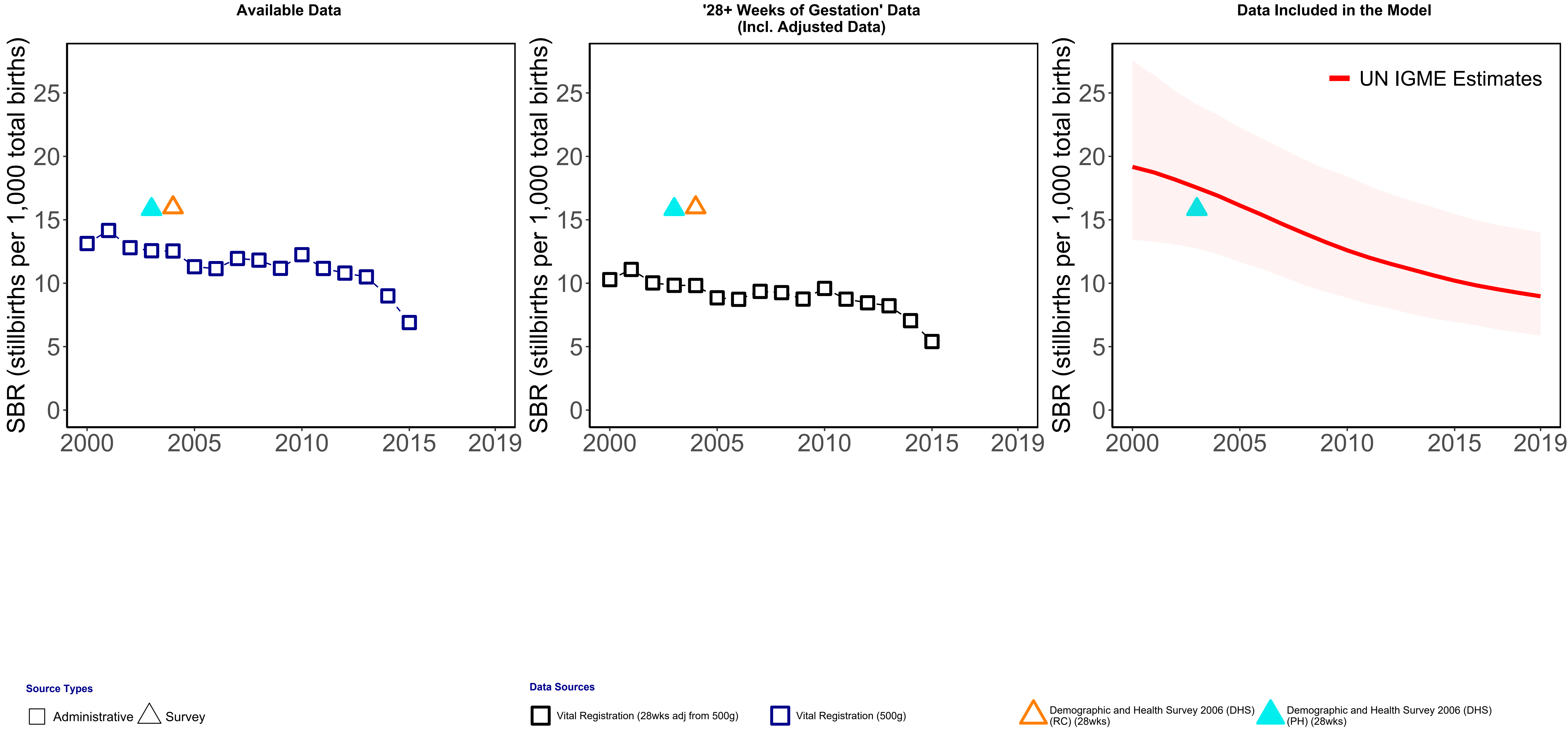

Available Data
'28+ Weeks of Gestation' Data
(Incl. Adjusted Data)
Data Included in the Model
UN IGME Estimates
SBR (stillbirths per 1,000 total births)
0
5
10
15
20
25
2000
2005
2010
2015
2019
Source Types
Administrative
Survey
Data Sources
Vital Registration (28wks adj from 500g)
Vital Registration (500g)
Demographic and Health Survey 2006 (DHS) (RC) (28wks)
Demographic and Health Survey 2006 (DHS) (PH) (28wks)

Burundi

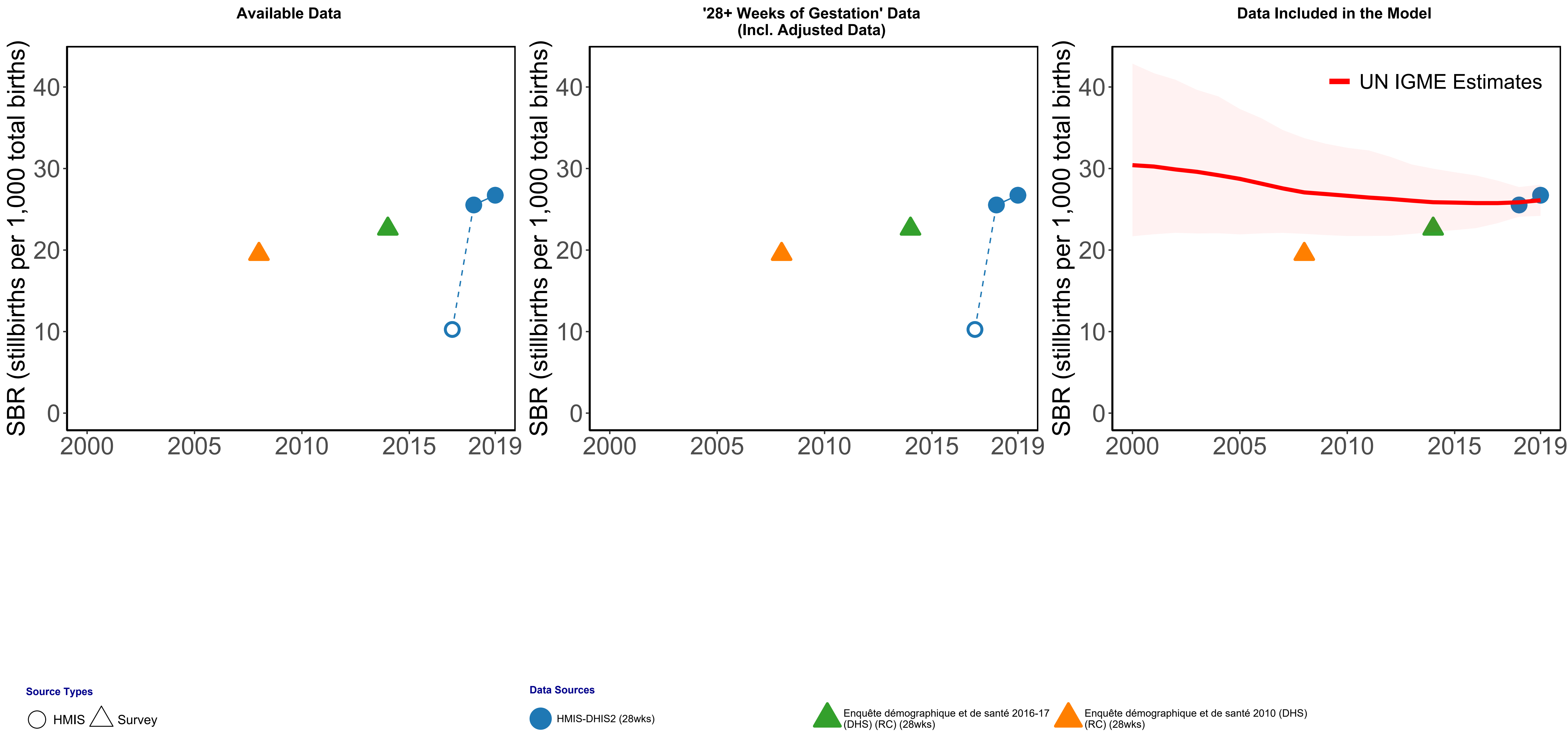
Available Data
'28+ Weeks of Gestation' Data
(Incl. Adjusted Data)
Data Included in the Model
SBR (stillbirths per 1,000 total births)
UN IGME Estimates
0
10
20
30
40
2000
2005
2010
2015
2019
Source Types
HMIS
Survey
Data Sources
HMIS-DHIS2 (28wks)
Enquête démographique et de santé 2016-17 (DHS) (RC) (28wks)
Enquête démographique et de santé 2010 (DHS) (RC) (28wks)

Belgium

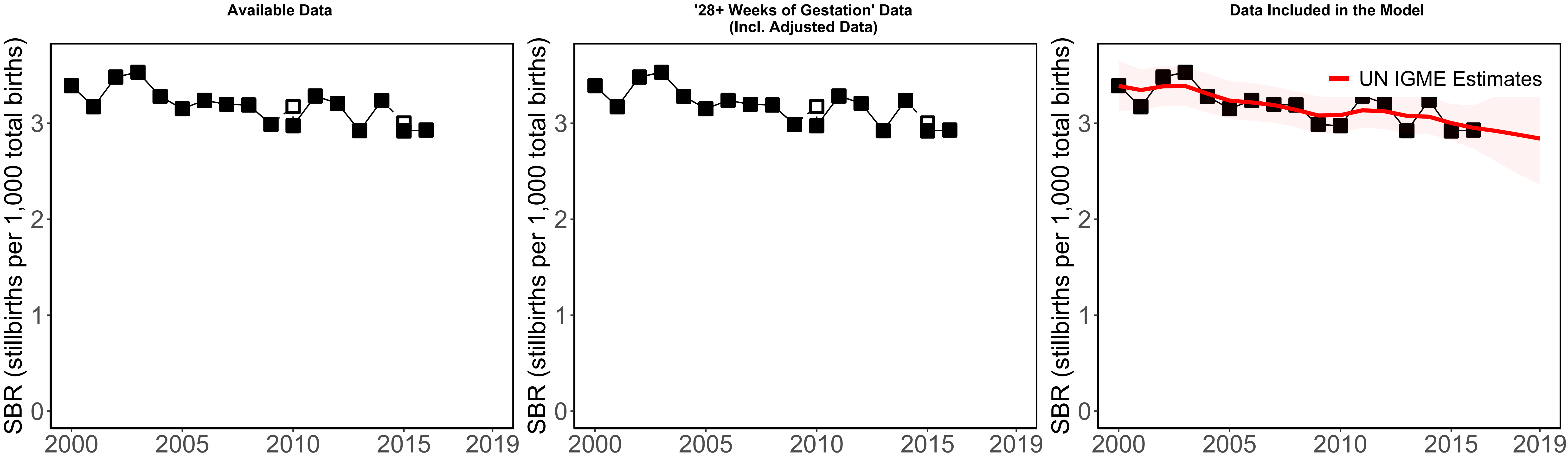

Source Types

Administrative

Data Sources

Vital Registration (28wks)

Benin

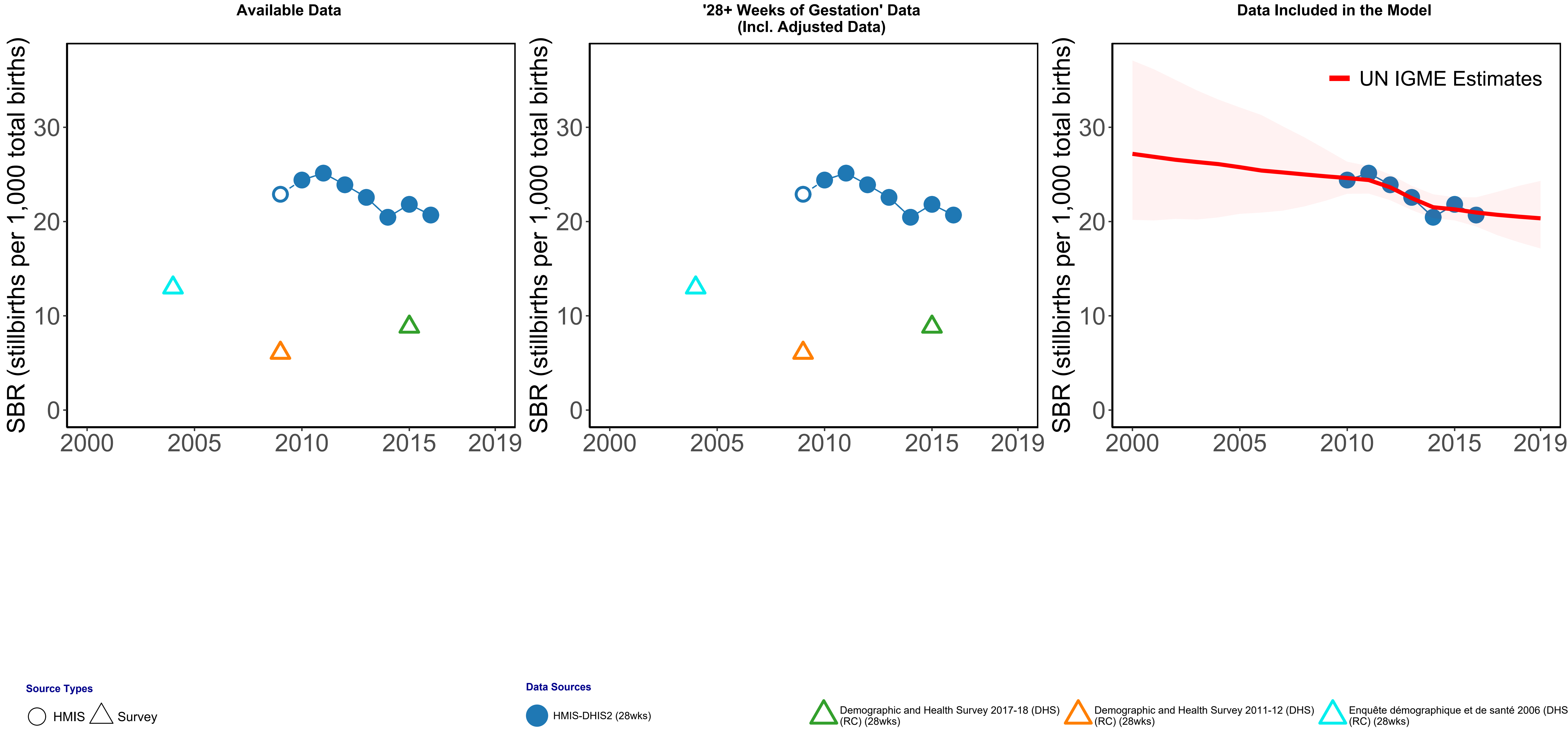

Available Data
'28+ Weeks of Gestation' Data
(Incl. Adjusted Data)
Data Included in the Model
SBR (stillbirths per 1,000 total births)
0
10
20
30
2000
2005
2010
2015
2019
UN IGME Estimates
Source Types
HMIS
Survey
Data Sources
HMIS-DHIS2 (28wks)
Demographic and Health Survey 2017-18 (DHS) (RC) (28wks)
Demographic and Health Survey 2011-12 (DHS) (RC) (28wks)
Enquête démographique et de santé 2006 (DHS (RC) (28wks)

Burkina Faso

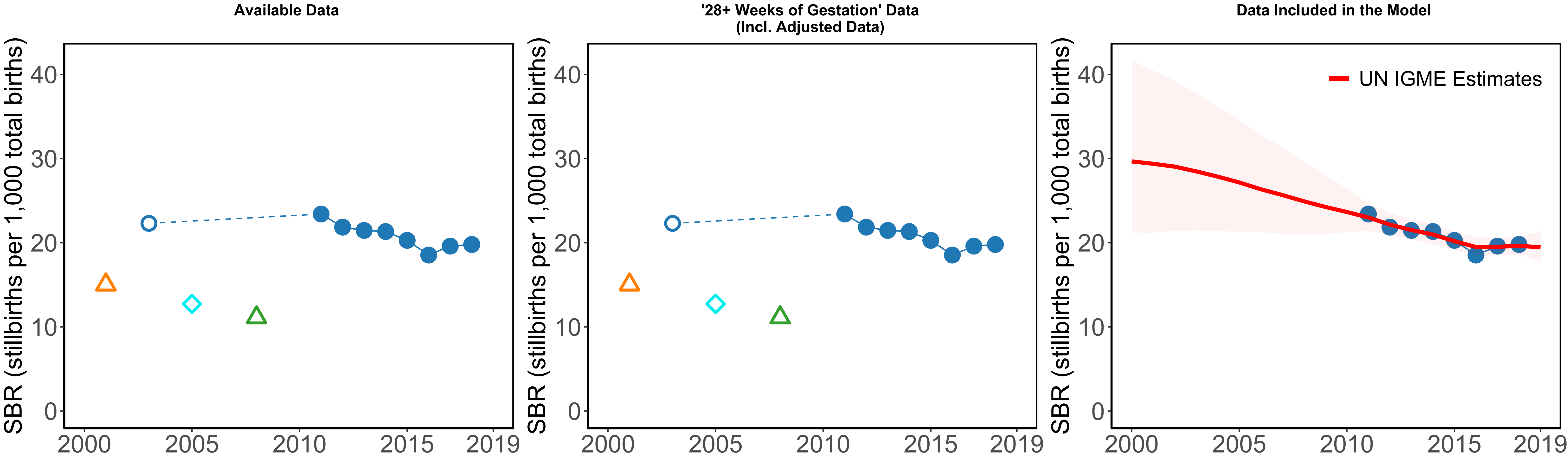

Source Types
HMIS Survey Population study

Data Sources
HMIS-DHIS2 (28wks)
Enquête démographique et de santé et à Indicateurs Multiples 2010 (DHS) (RC) (28wks)
Enquête démographique et de santé 2003 (DHS) (RC) (28wks)
Roberfroid 2008 (28wks)

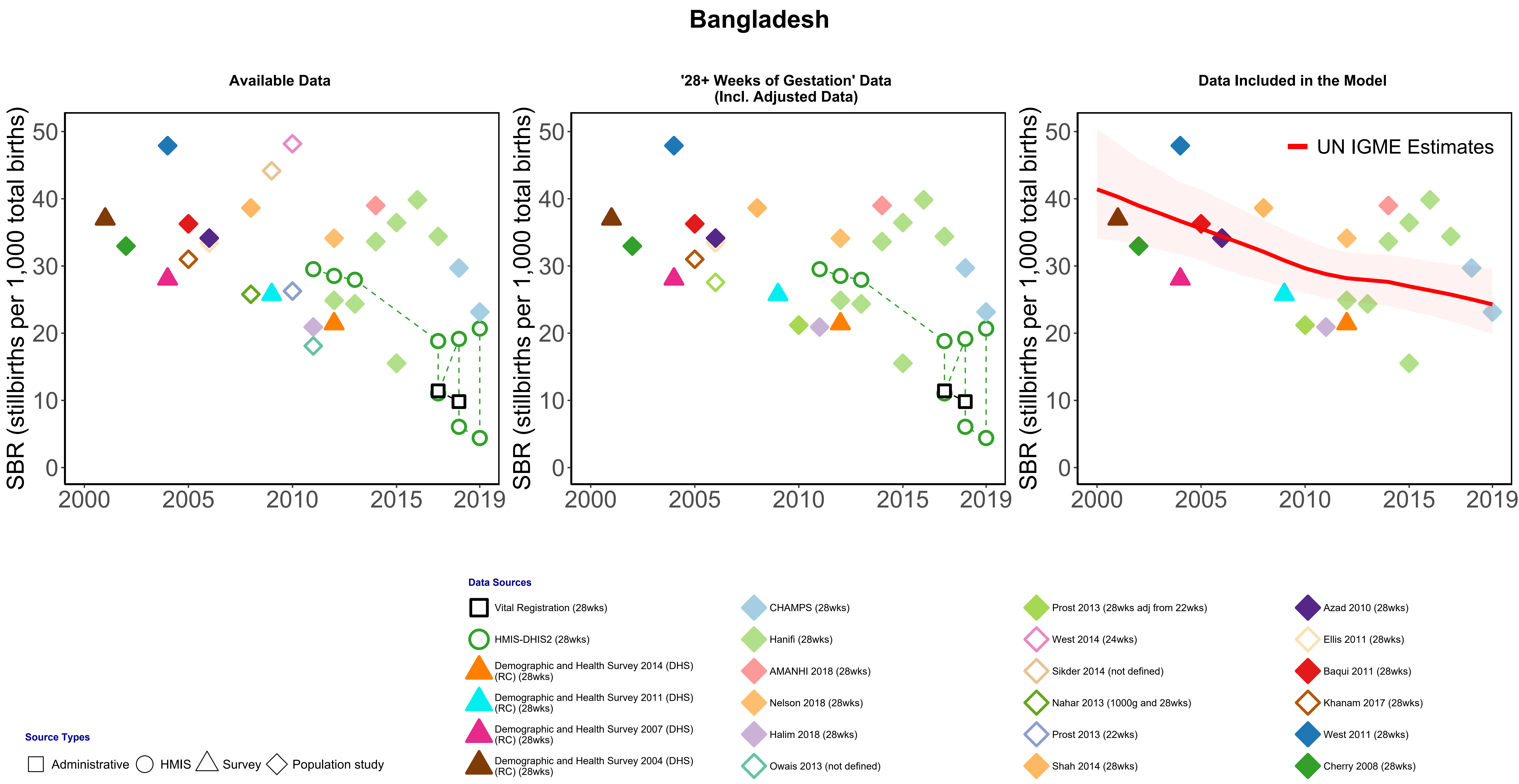
Bangladesh
Available Data
'28+ Weeks of Gestation' Data
(Incl. Adjusted Data)
Data Included in the Model
SBR (stillbirths per 1,000 total births)
0
10
20
30
40
50
2000
2005
2010
2015
2019
UN IGME Estimates
Data Sources
Vital Registration (28wks)
HMIS-DHIS2 (28wks)
Demographic and Health Survey 2014 (DHS) (RC) (28wks)
Demographic and Health Survey 2011 (DHS) (RC) (28wks)
Demographic and Health Survey 2007 (DHS) (RC) (28wks)
Demographic and Health Survey 2004 (DHS) (RC) (28wks)
CHAMPS (28wks)
Hanifi (28wks)
AMANHI 2018 (28wks)
Nelson 2018 (28wks)
Halim 2018 (28wks)
Owais 2013 (not defined)
Prost 2013 (28wks adj from 22wks)
West 2014 (24wks)
Sikder 2014 (not defined)
Nahar 2013 (1000g and 28wks)
Prost 2013 (22wks)
Shah 2014 (28wks)
Azad 2010 (28wks)
Ellis 2011 (28wks)
Baqui 2011 (28wks)
Khanam 2017 (28wks)
West 2011 (28wks)
Cherry 2008 (28wks)
Source Types
Administrative
HMIS
Survey
Population study

Bulgaria

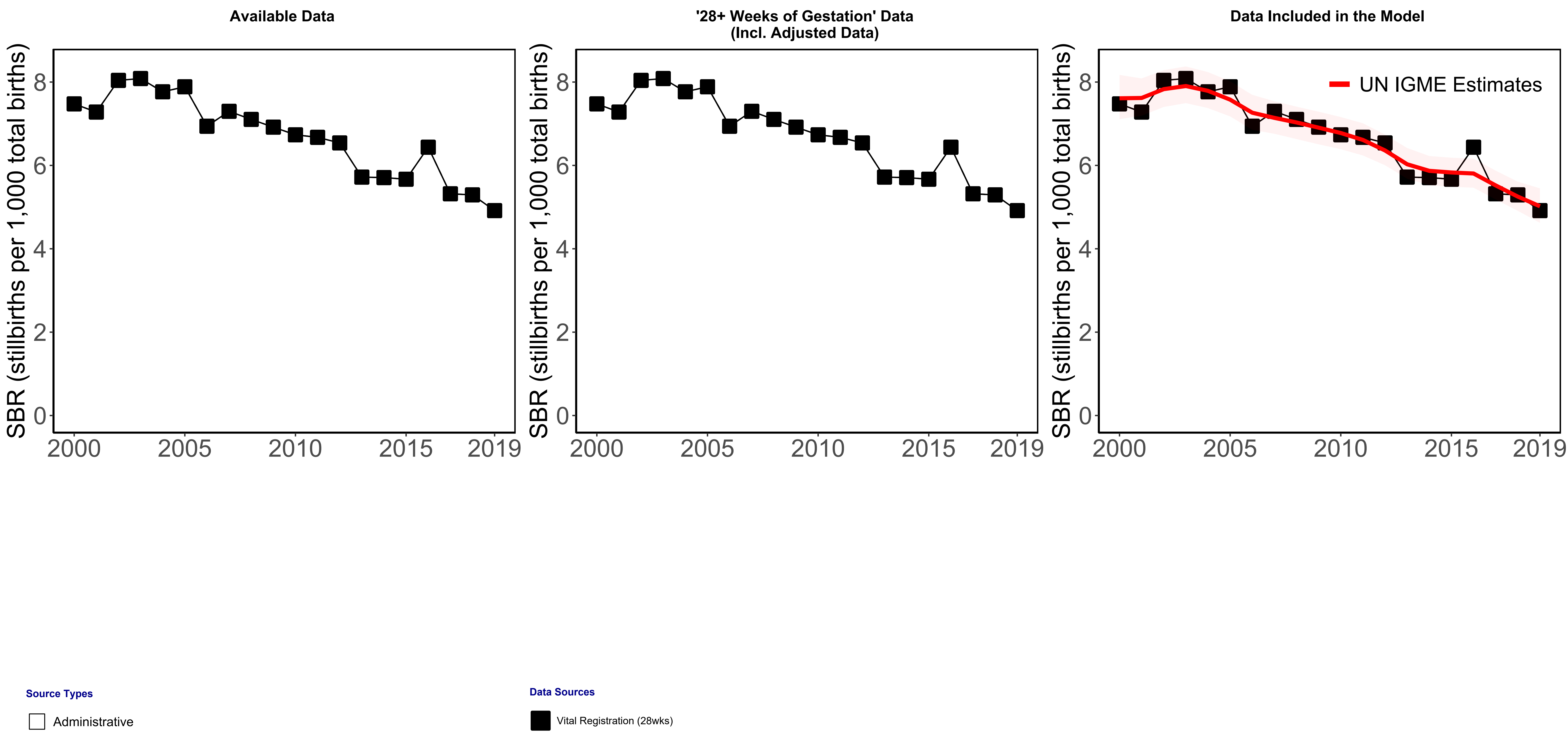

Available Data
'28+ Weeks of Gestation' Data
(Incl. Adjusted Data)
Data Included in the Model
SBR (stillbirths per 1,000 total births)
UN IGME Estimates
0
2
4
6
8
2000
2005
2010
2015
2019
Source Types
Administrative
Data Sources
Vital Registration (28wks)

Bahrain

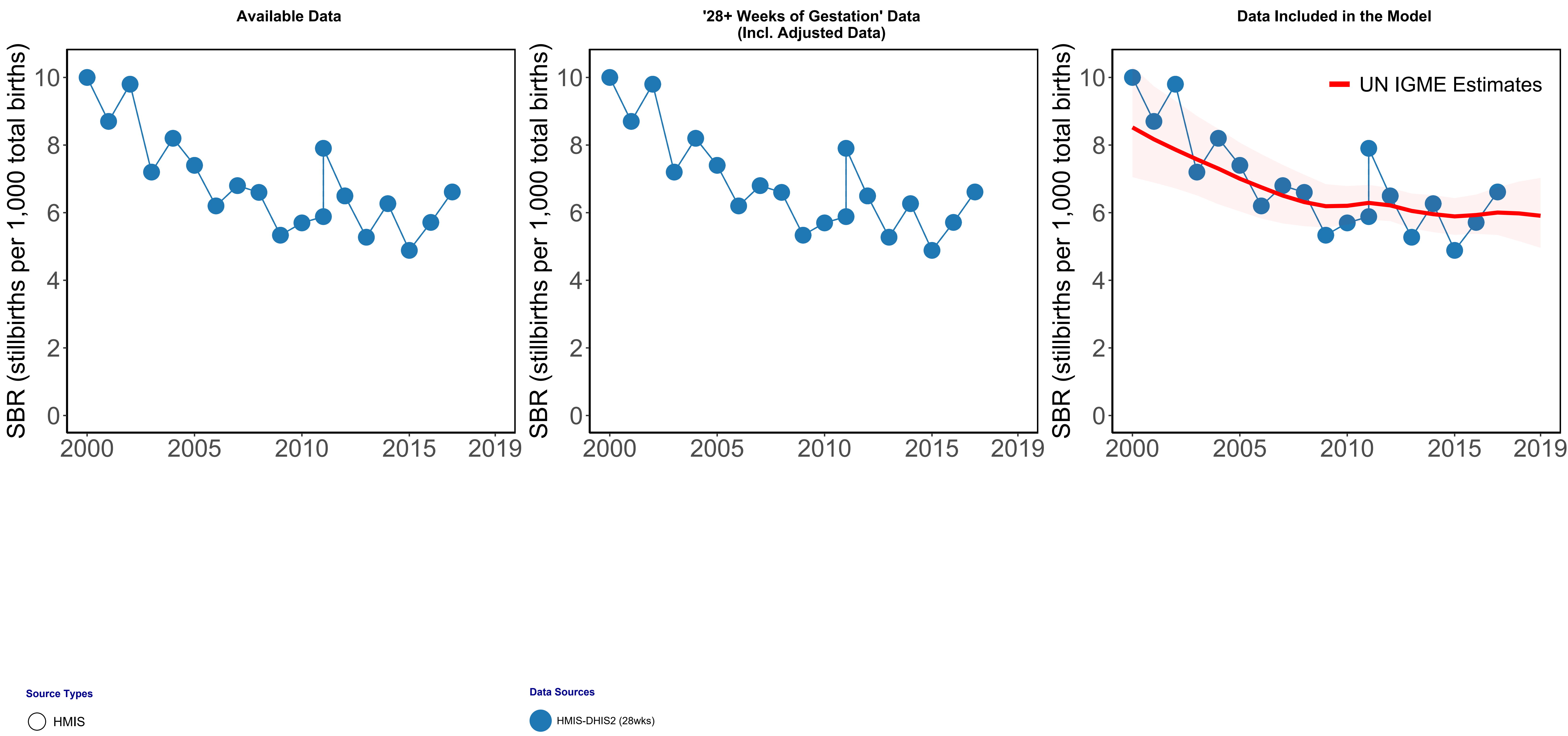

Available Data
'28+ Weeks of Gestation' Data
(Incl. Adjusted Data)
Data Included in the Model
SBR (stillbirths per 1,000 total births)
0
2
4
6
8
10
2000
2005
2010
2015
2019
UN IGME Estimates
Source Types
HMIS
Data Sources
HMIS-DHIS2 (28wks)

Bahamas

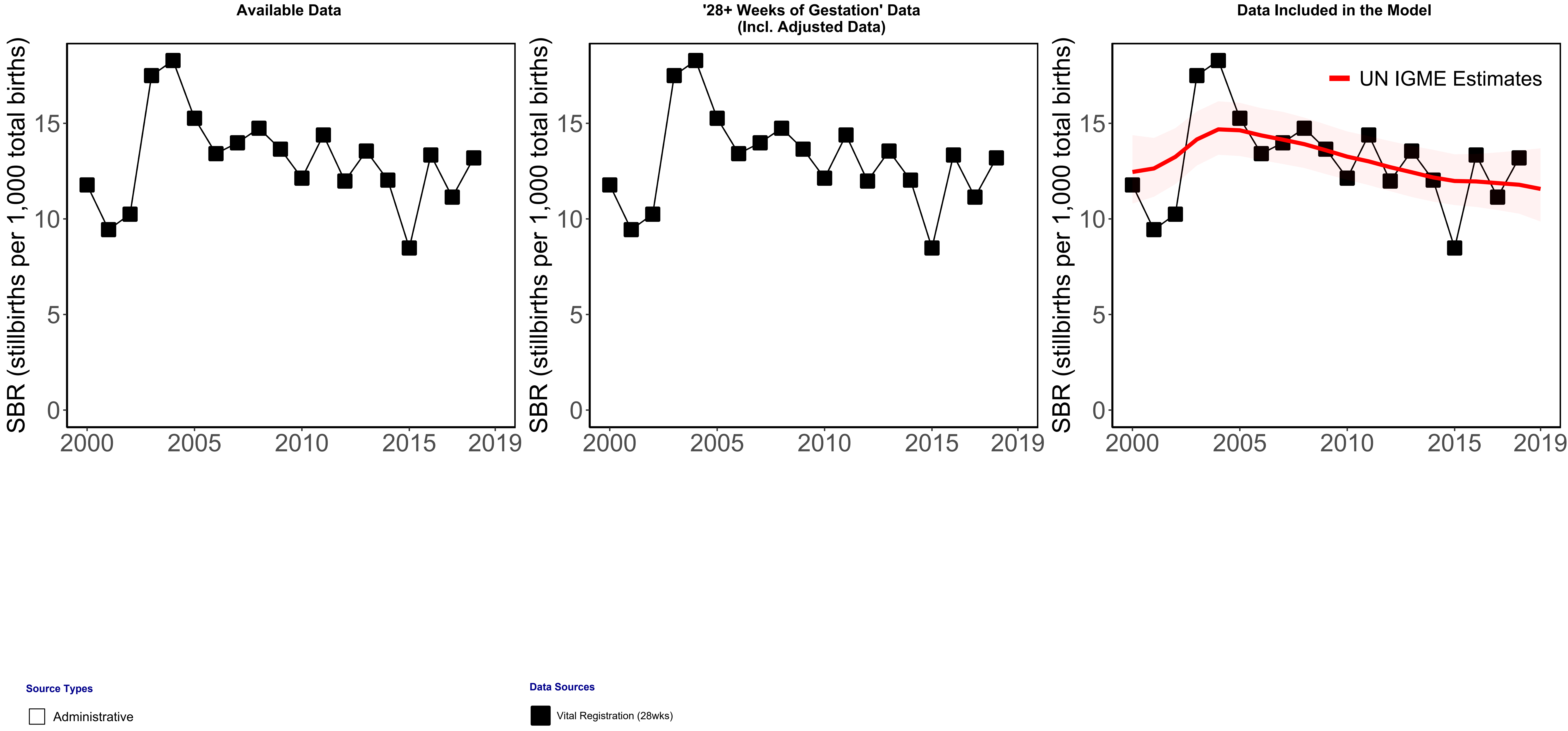

Available Data
'28+ Weeks of Gestation' Data
(Incl. Adjusted Data)
Data Included in the Model
UN IGME Estimates
SBR (stillbirths per 1,000 total births)
0
5
10
15
2000
2005
2010
2015
2019
Source Types
Administrative
Data Sources
Vital Registration (28wks)

Bosnia and Herzegovina

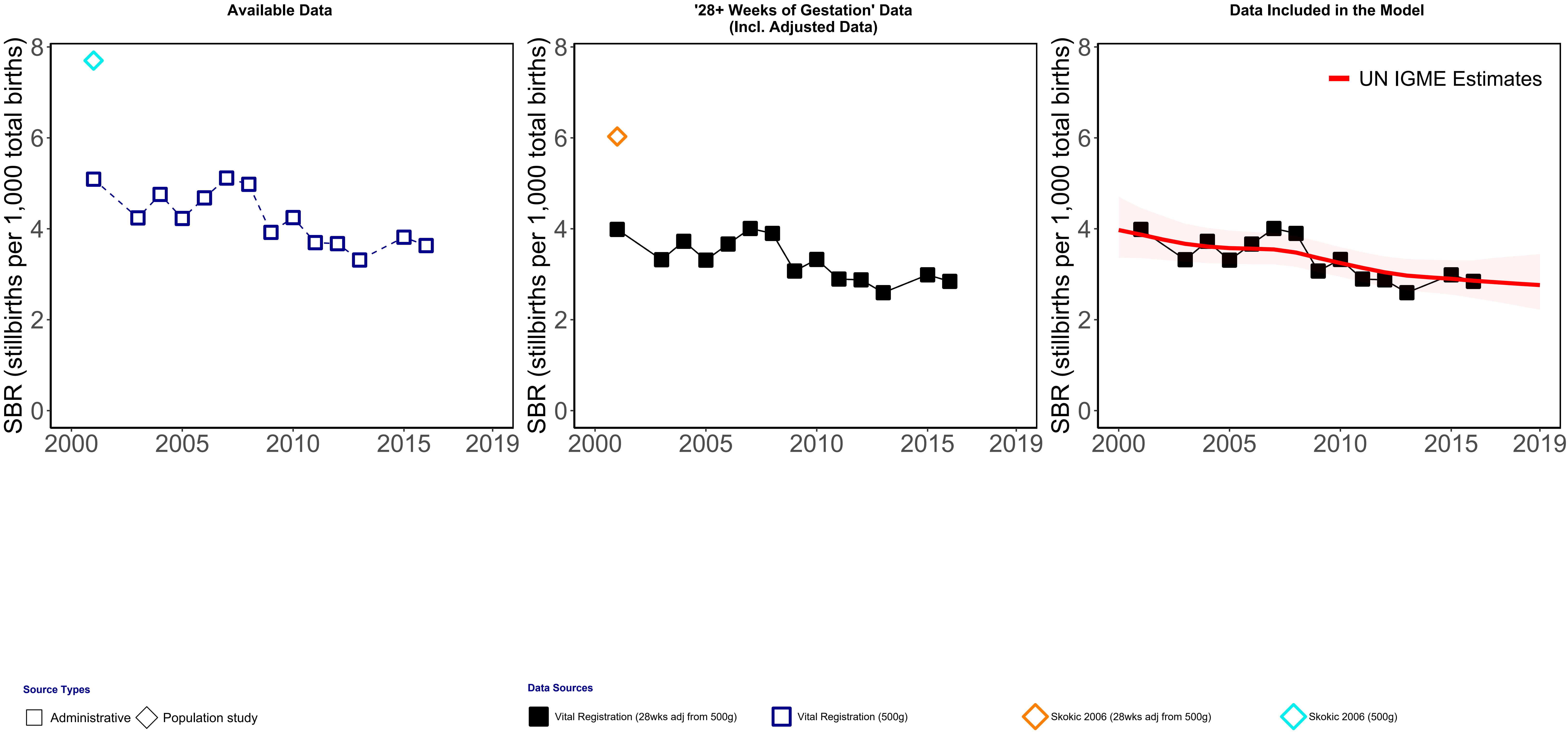

Belarus

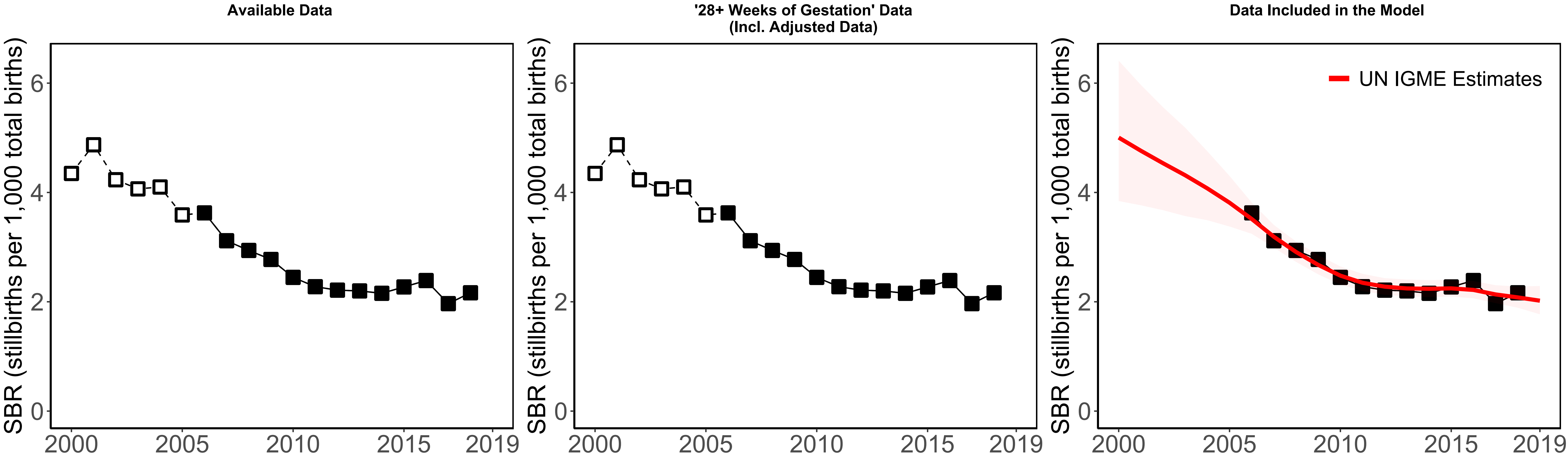

Source Types

Administrative

Data Sources

Vital Registration (28wks)

Belize

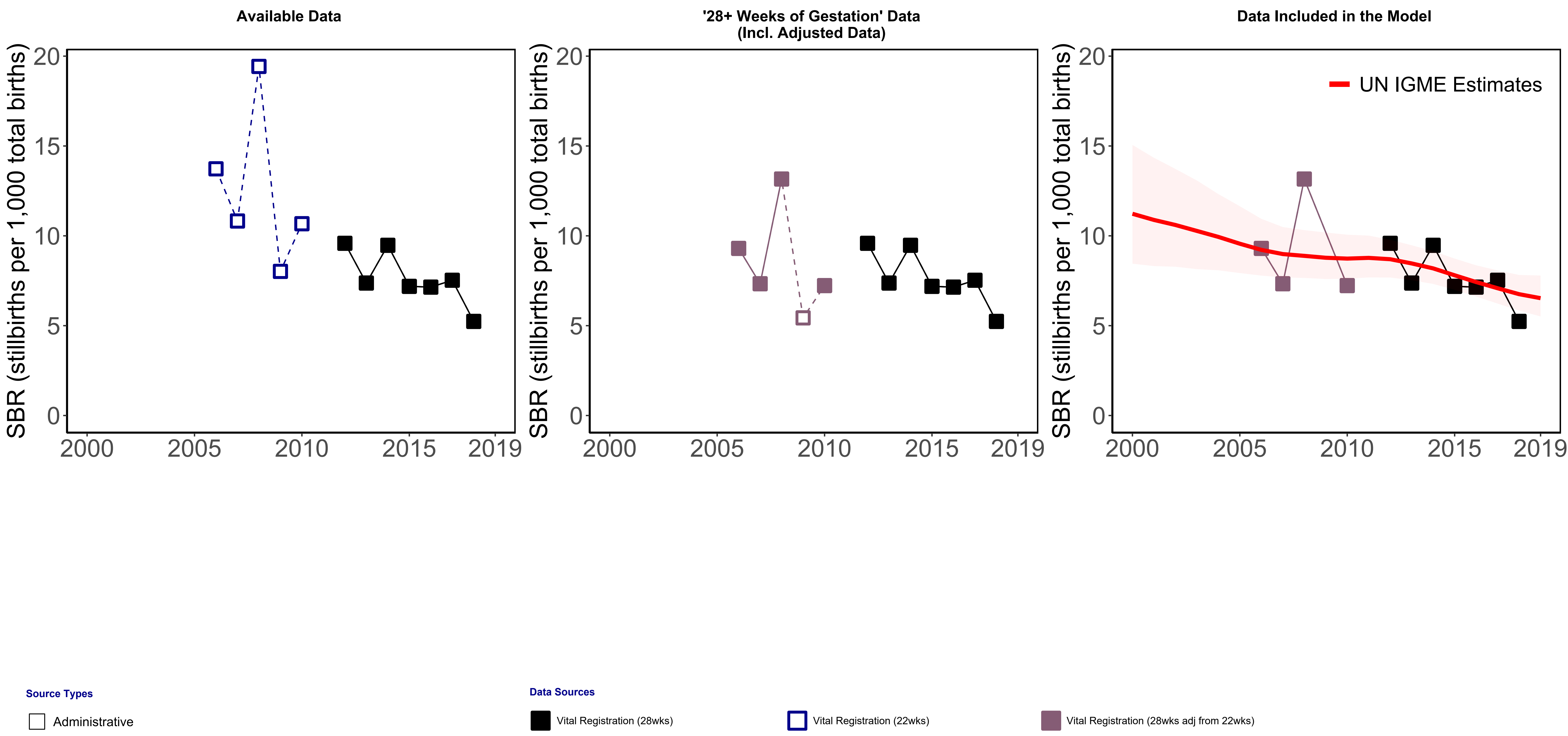

Available Data
'28+ Weeks of Gestation' Data
(Incl. Adjusted Data)
Data Included in the Model
SBR (stillbirths per 1,000 total births)
0
5
10
15
20
2000
2005
2010
2015
2019
UN IGME Estimates
Source Types
Administrative
Data Sources
Vital Registration (28wks)
Vital Registration (22wks)
Vital Registration (28wks adj from 22wks)

Bolivia

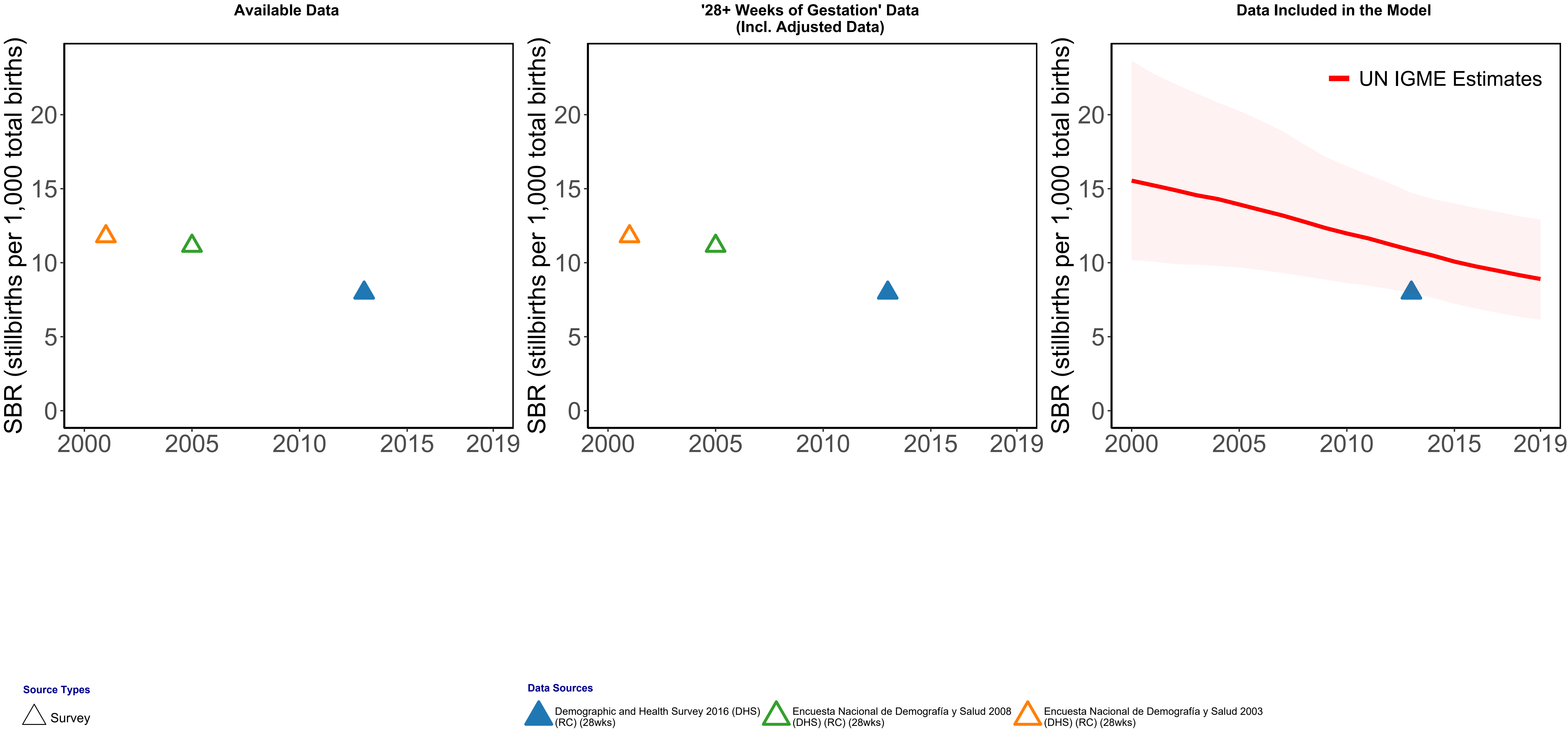

Available Data
'28+ Weeks of Gestation' Data
(Incl. Adjusted Data)
Data Included in the Model
SBR (stillbirths per 1,000 total births)
0
5
10
15
20
2000
2005
2010
2015
2019
UN IGME Estimates
Source Types
Survey
Data Sources
Demographic and Health Survey 2016 (DHS) (RC) (28wks)
Encuesta Nacional de Demografía y Salud 2008 (DHS) (RC) (28wks)
Encuesta Nacional de Demografía y Salud 2003 (DHS) (RC) (28wks)

Brazil

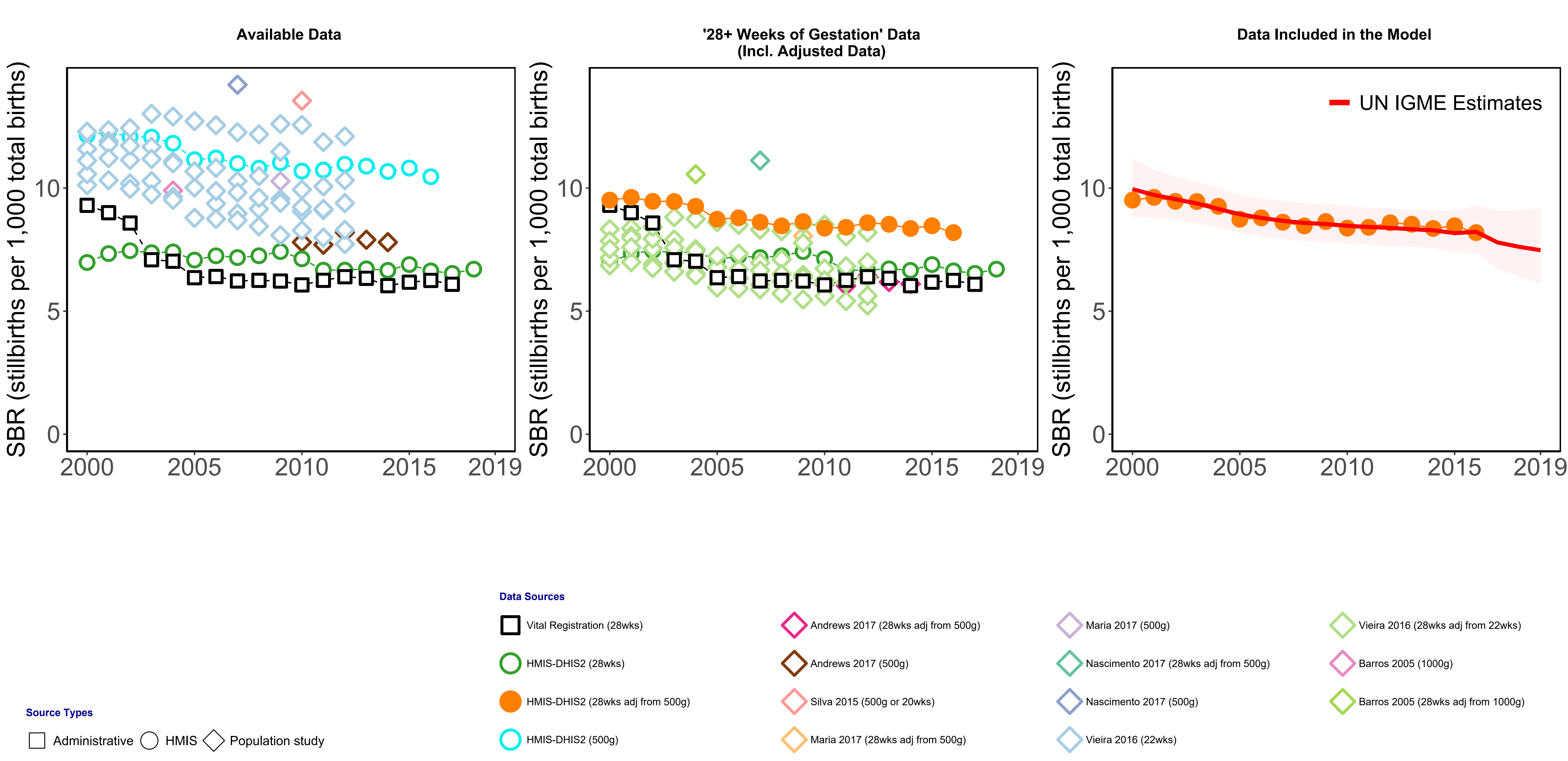
Available Data
'28+ Weeks of Gestation' Data
(Incl. Adjusted Data)
Data Included in the Model
SBR (stillbirths per 1,000 total births)
0
5
10
2000
2005
2010
2015
2019
UN IGME Estimates
Data Sources
Vital Registration (28wks)
HMIS-DHIS2 (28wks)
HMIS-DHIS2 (28wks adj from 500g)
HMIS-DHIS2 (500g)
Andrews 2017 (28wks adj from 500g)
Andrews 2017 (500g)
Silva 2015 (500g or 20wks)
Maria 2017 (28wks adj from 500g)
Maria 2017 (500g)
Nascimento 2017 (28wks adj from 500g)
Nascimento 2017 (500g)
Vieira 2016 (22wks)
Vieira 2016 (28wks adj from 22wks)
Barros 2005 (1000g)
Barros 2005 (28wks adj from 1000g)
Source Types
Administrative
HMIS
Population study

Barbados

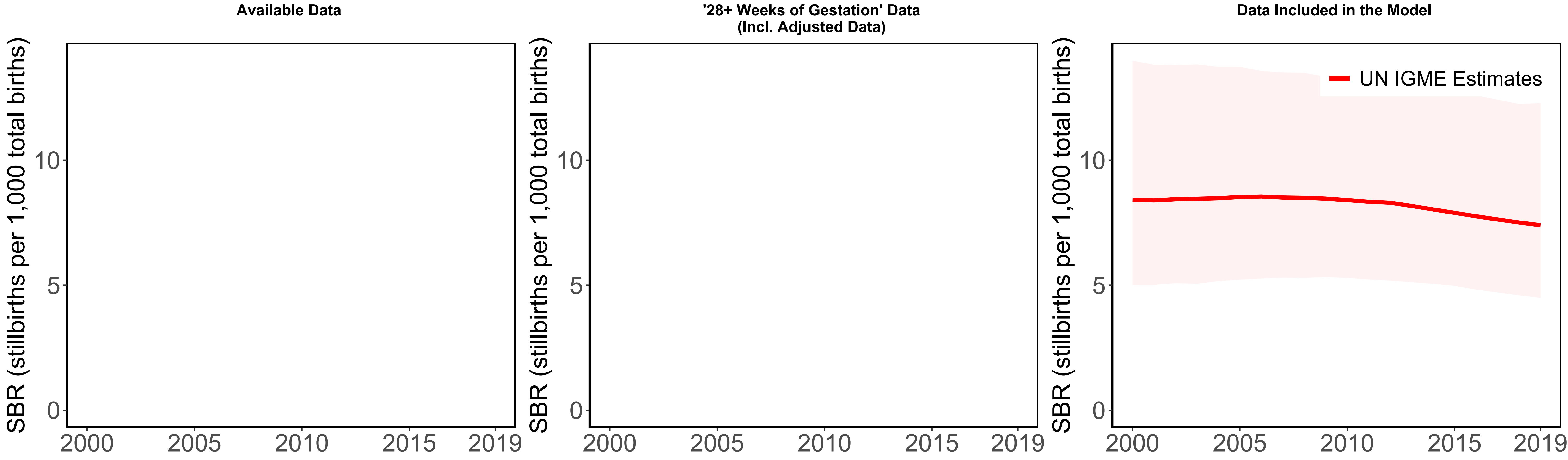

Available Data
'28+ Weeks of Gestation' Data
(Incl. Adjusted Data)
Data Included in the Model
SBR (stillbirths per 1,000 total births)
UN IGME Estimates
0
5
10
2000
2005
2010
2015
2019

Brunei Darussalam

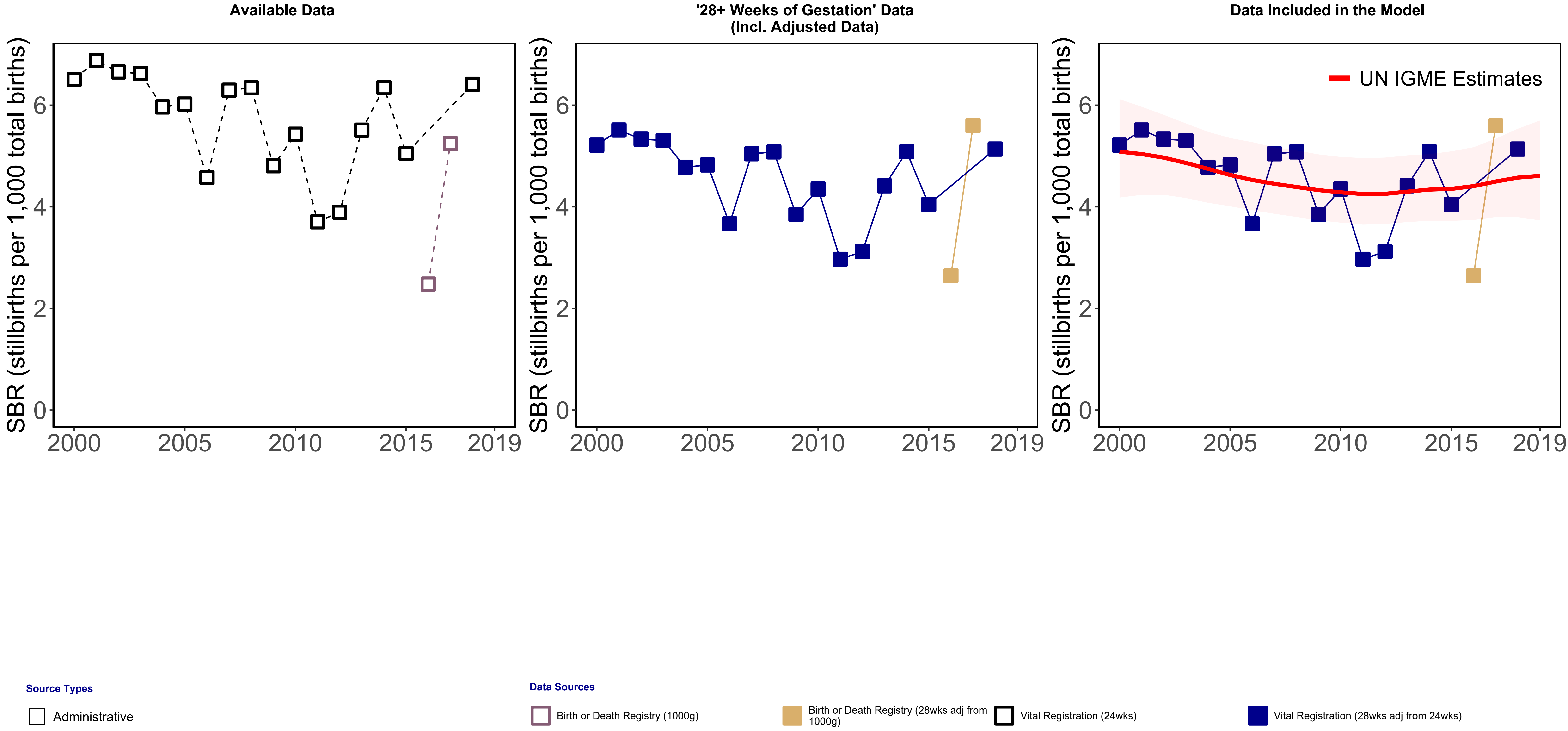

Bhutan

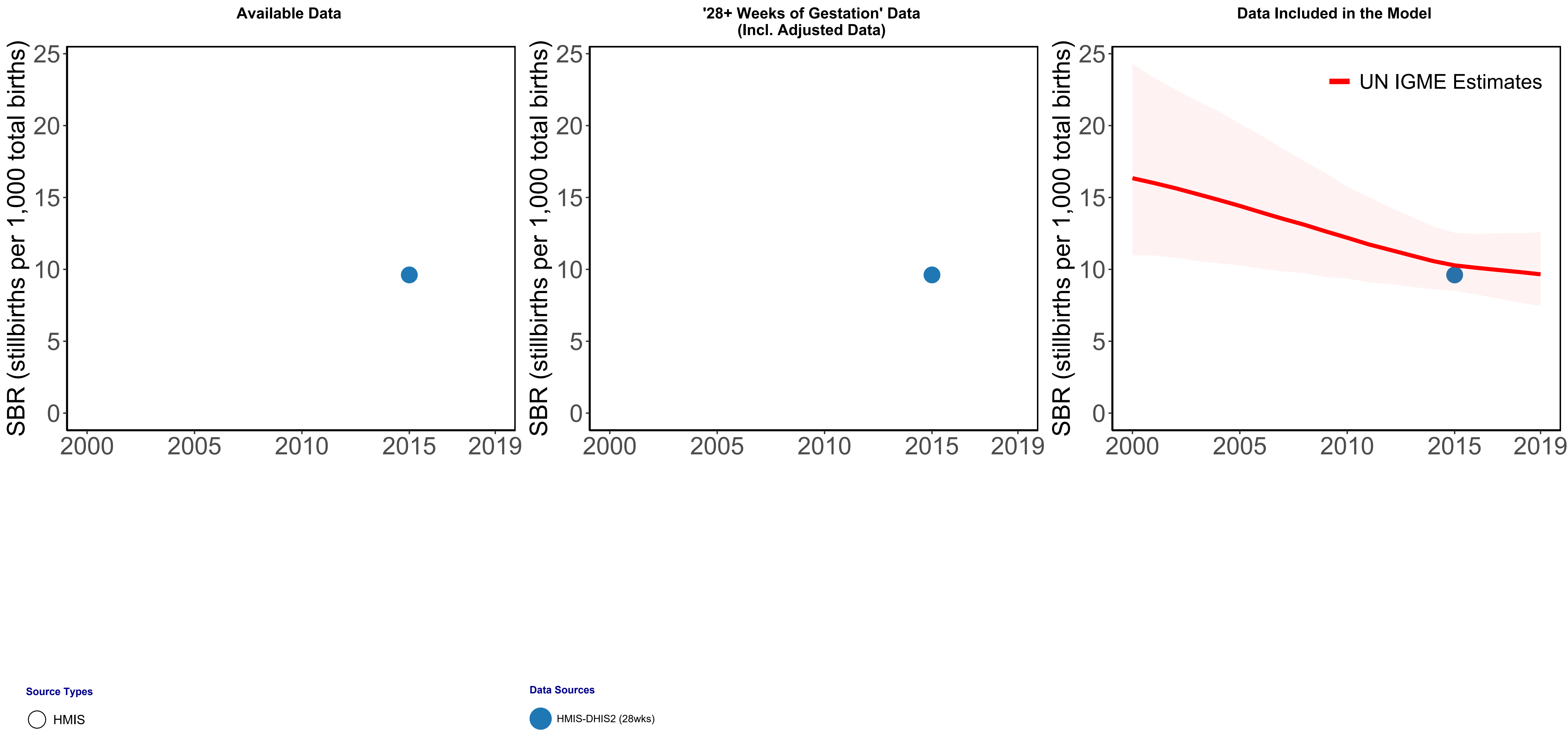
Available Data
'28+ Weeks of Gestation' Data
(Incl. Adjusted Data)
Data Included in the Model
SBR (stillbirths per 1,000 total births)
0
5
10
15
20
25
2000
2005
2010
2015
2019
UN IGME Estimates
Source Types
HMIS
Data Sources
HMIS-DHIS2 (28wks)

Botswana

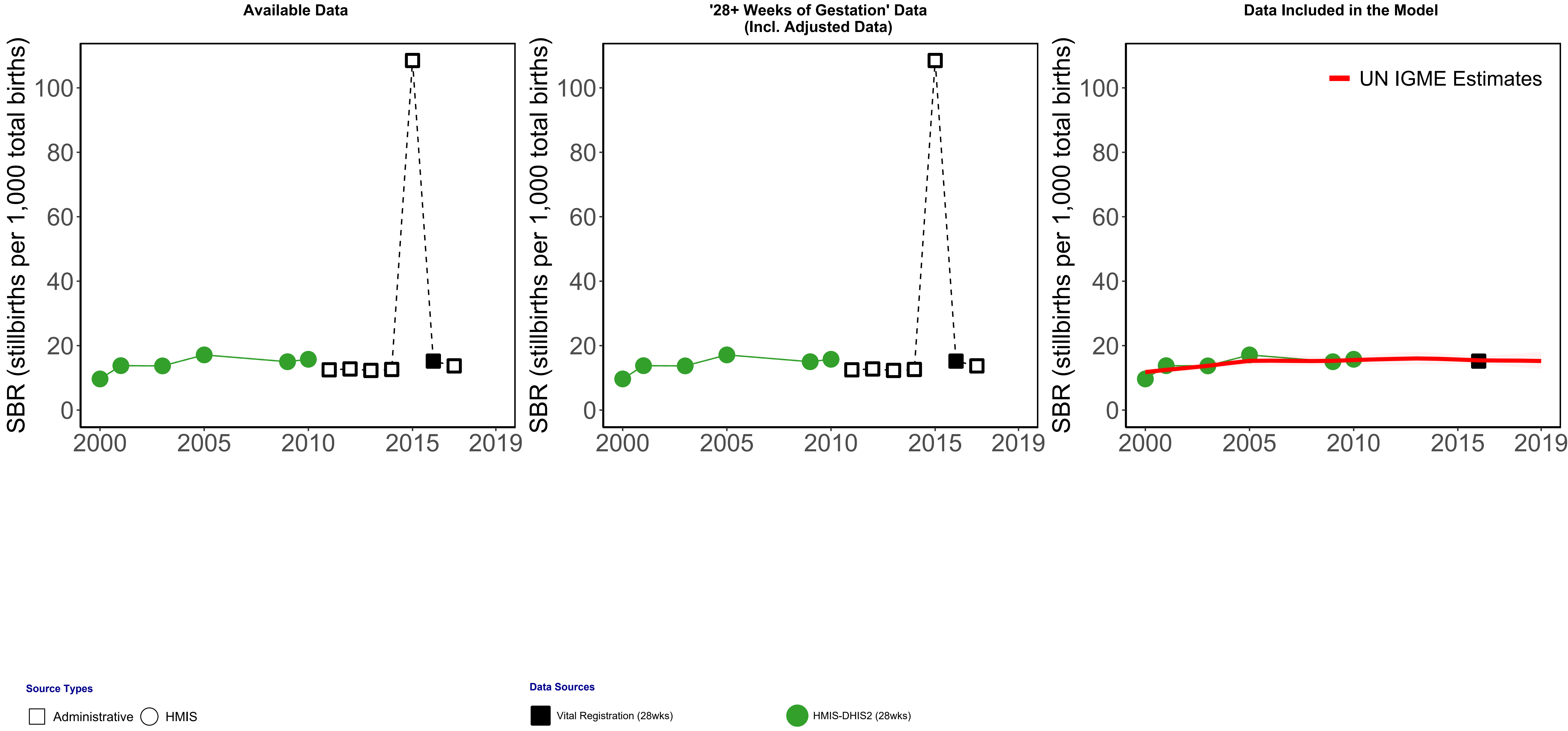
Available Data
'28+ Weeks of Gestation' Data
(Incl. Adjusted Data)
Data Included in the Model
UN IGME Estimates
SBR (stillbirths per 1,000 total births)
0
20
40
60
80
100
2000
2005
2010
2015
2019
Source Types
Administrative
HMIS
Data Sources
Vital Registration (28wks)
HMIS-DHIS2 (28wks)

Central African Republic

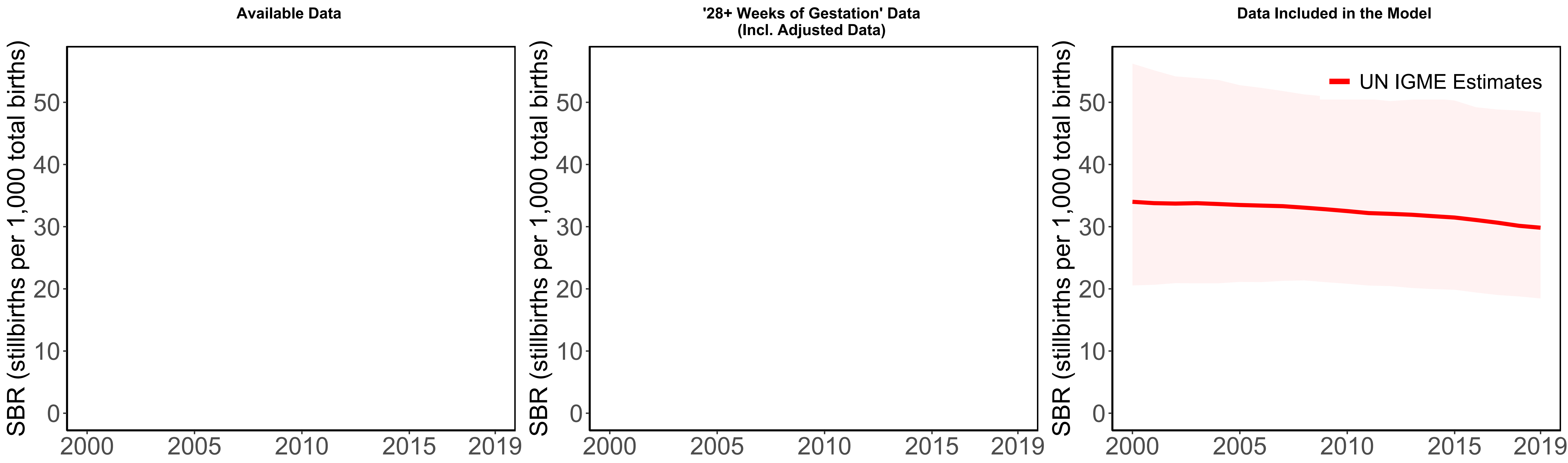

Canada

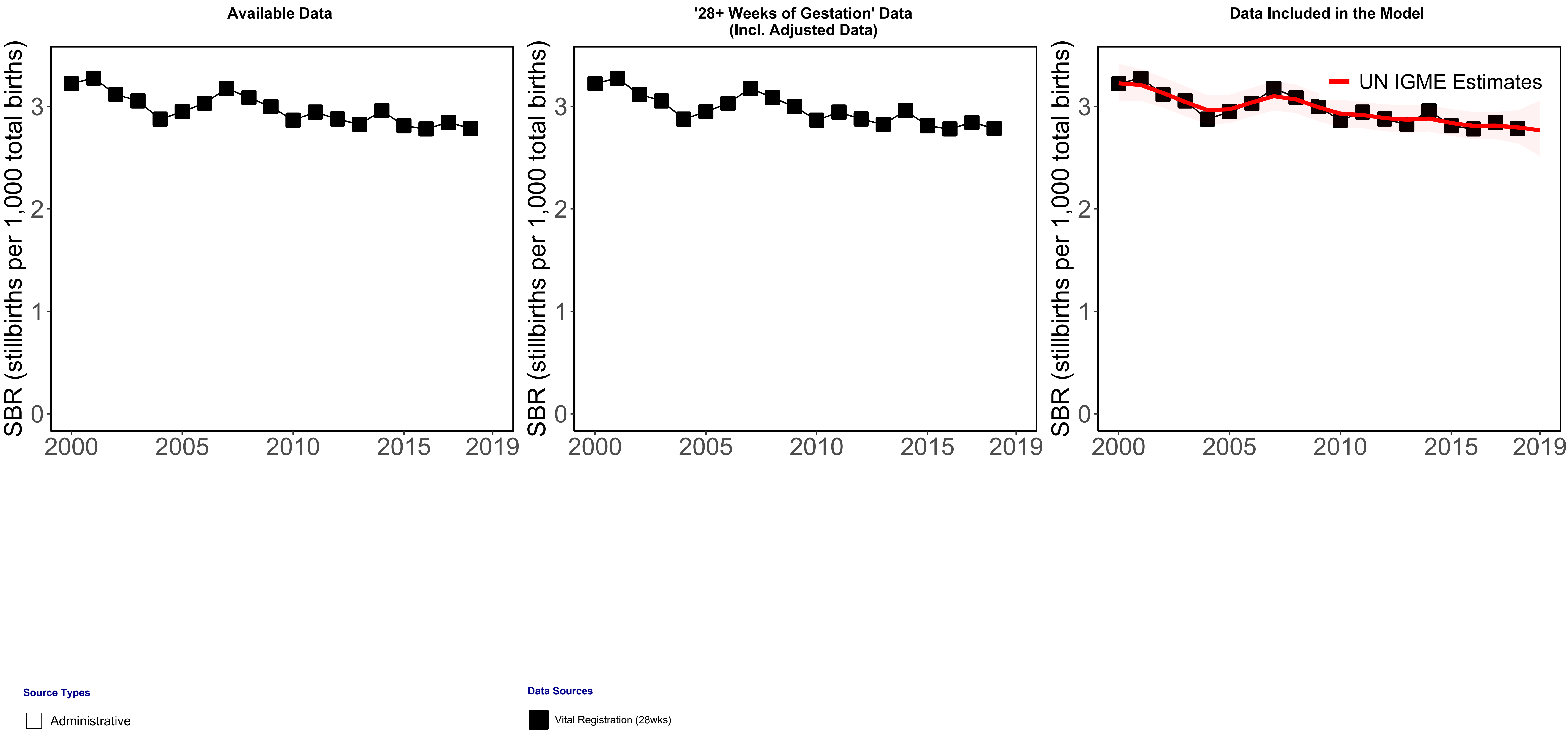
Available Data
'28+ Weeks of Gestation' Data
(Incl. Adjusted Data)
Data Included in the Model
SBR (stillbirths per 1,000 total births)
0
1
2
3
2000
2005
2010
2015
2019
UN IGME Estimates
Source Types
Administrative
Data Sources
Vital Registration (28wks)

Switzerland

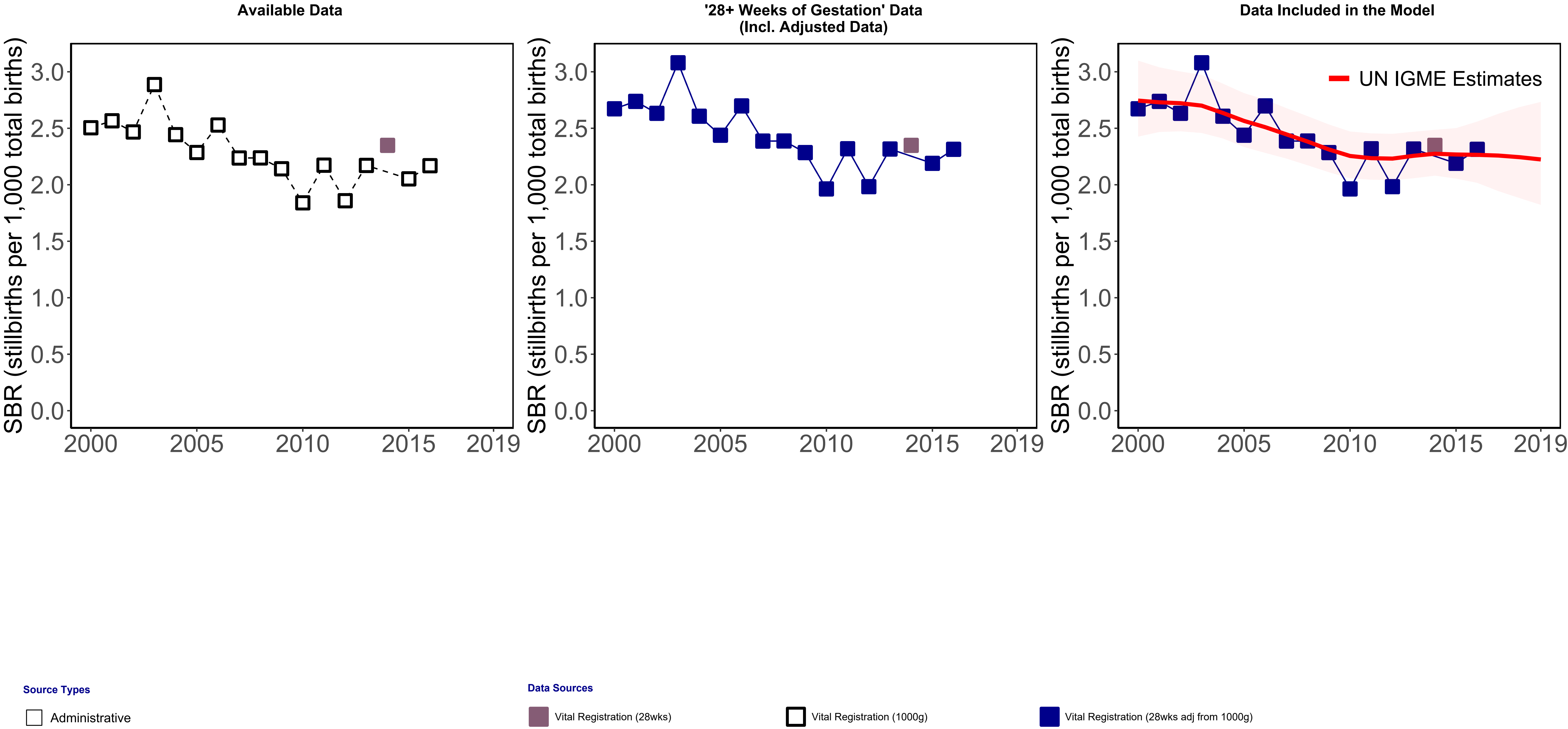

Chile

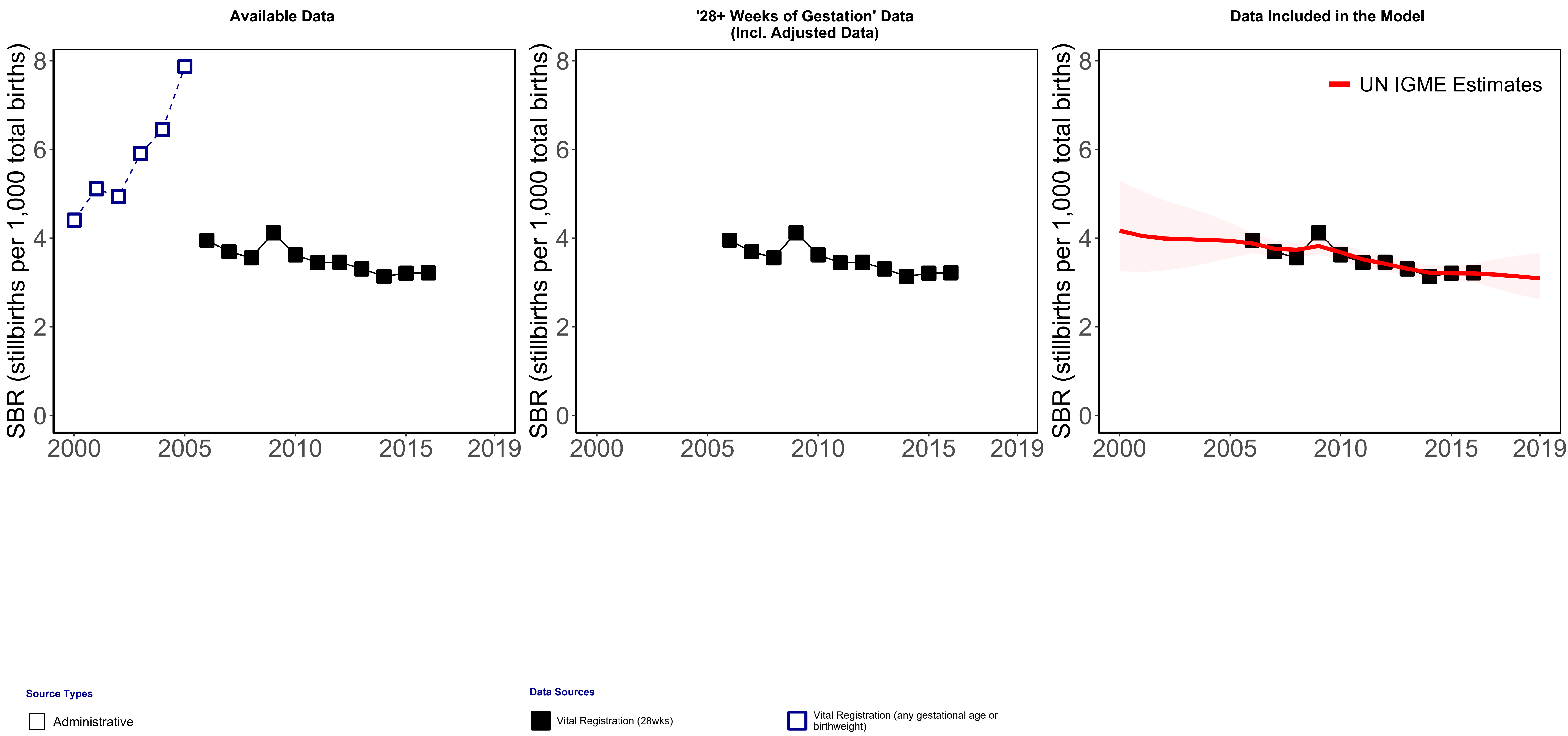

Available Data
'28+ Weeks of Gestation' Data
(Incl. Adjusted Data)
Data Included in the Model
SBR (stillbirths per 1,000 total births)
0
2
4
6
8
2000
2005
2010
2015
2019
UN IGME Estimates
Source Types
Administrative
Data Sources
Vital Registration (28wks)
Vital Registration (any gestational age or birthweight)

China

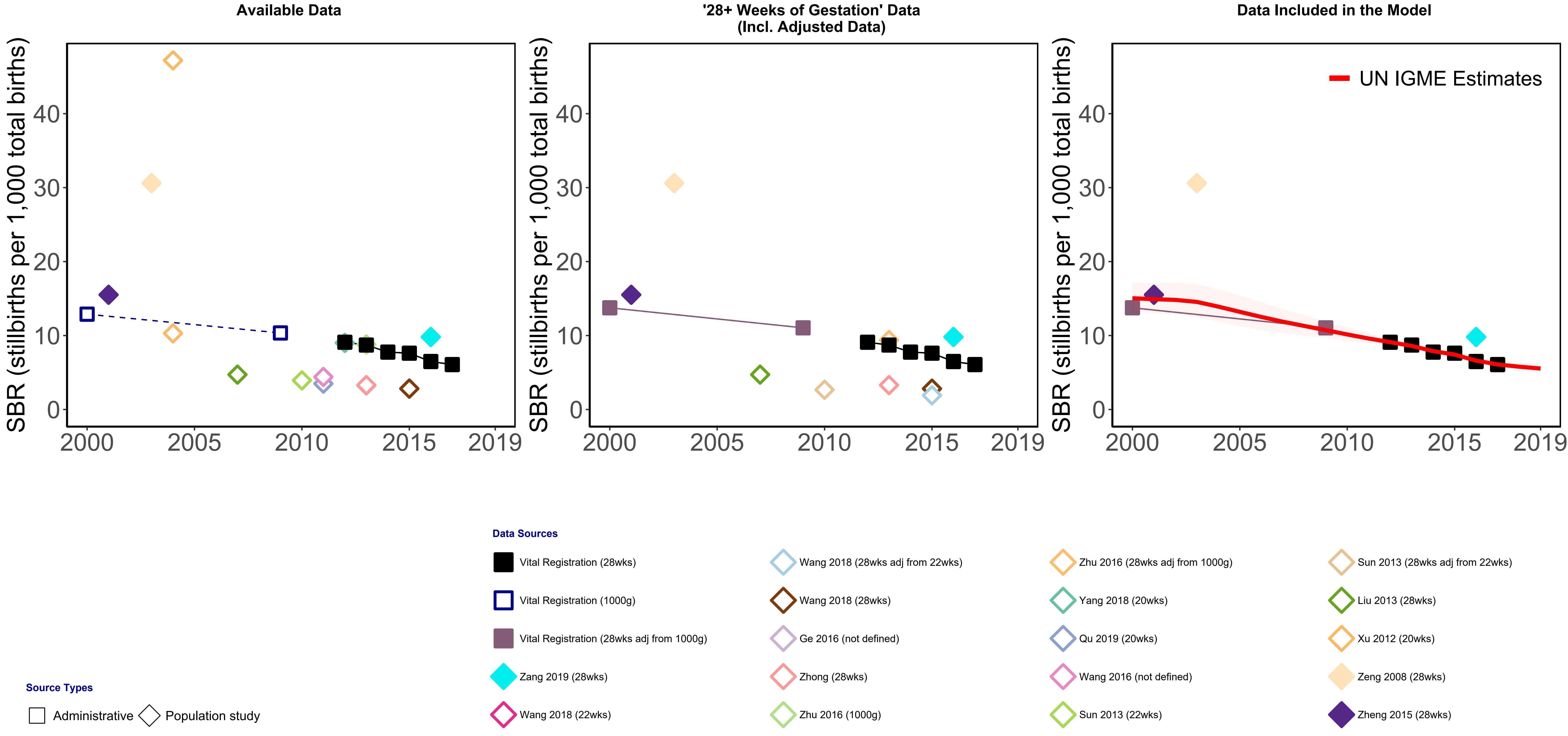
Available Data
'28+ Weeks of Gestation' Data
(Incl. Adjusted Data)
Data Included in the Model
SBR (stillbirths per 1,000 total births)
UN IGME Estimates
Data Sources
Vital Registration (28wks)
Vital Registration (1000g)
Vital Registration (28wks adj from 1000g)
Zang 2019 (28wks)
Wang 2018 (22wks)
Wang 2018 (28wks adj from 22wks)
Wang 2018 (28wks)
Ge 2016 (not defined)
Zhong (28wks)
Zhu 2016 (1000g)
Zhu 2016 (28wks adj from 1000g)
Yang 2018 (20wks)
Qu 2019 (20wks)
Wang 2016 (not defined)
Sun 2013 (22wks)
Sun 2013 (28wks adj from 22wks)
Liu 2013 (28wks)
Xu 2012 (20wks)
Zeng 2008 (28wks)
Zheng 2015 (28wks)
Source Types
Administrative
Population study

Cote d'Ivoire

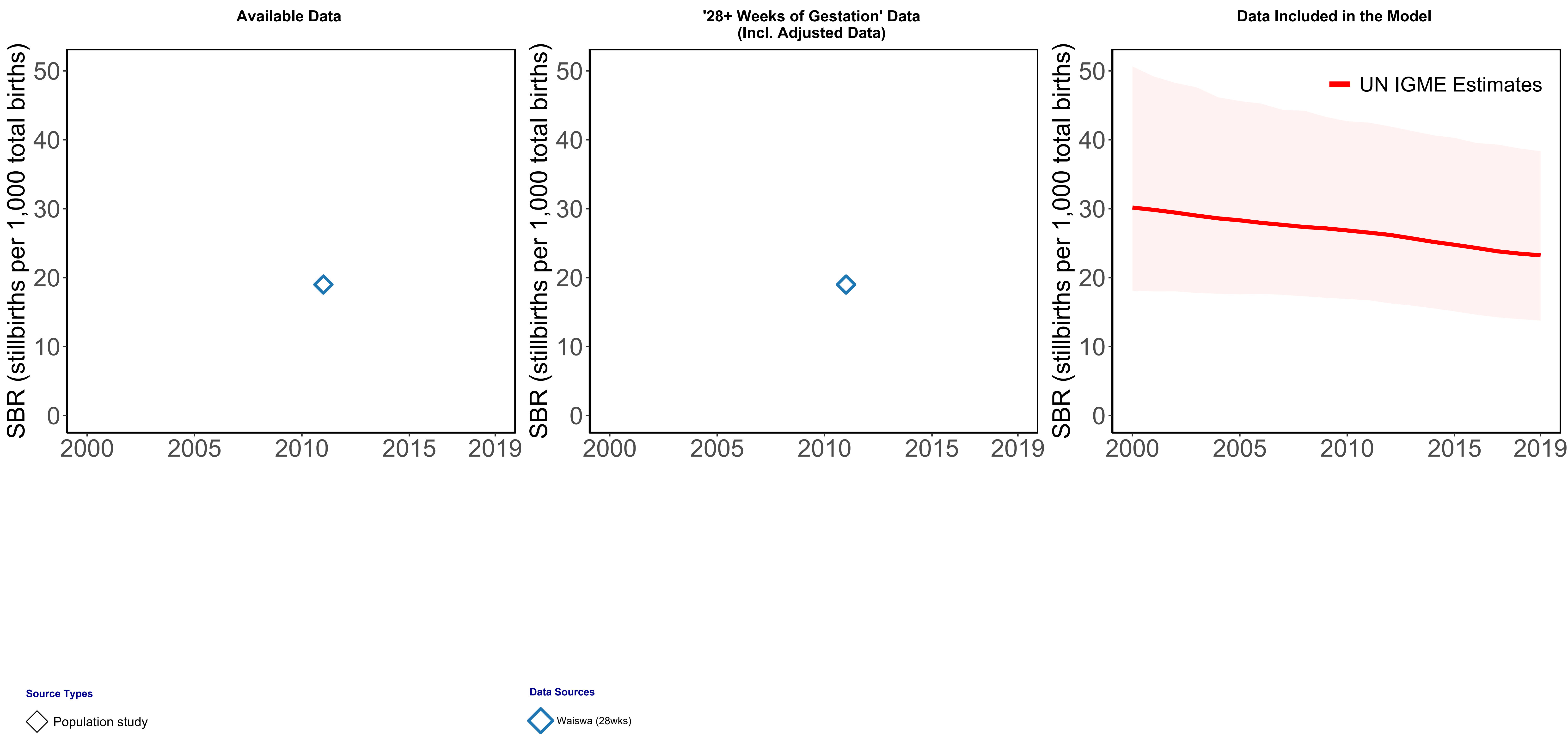

Cameroon

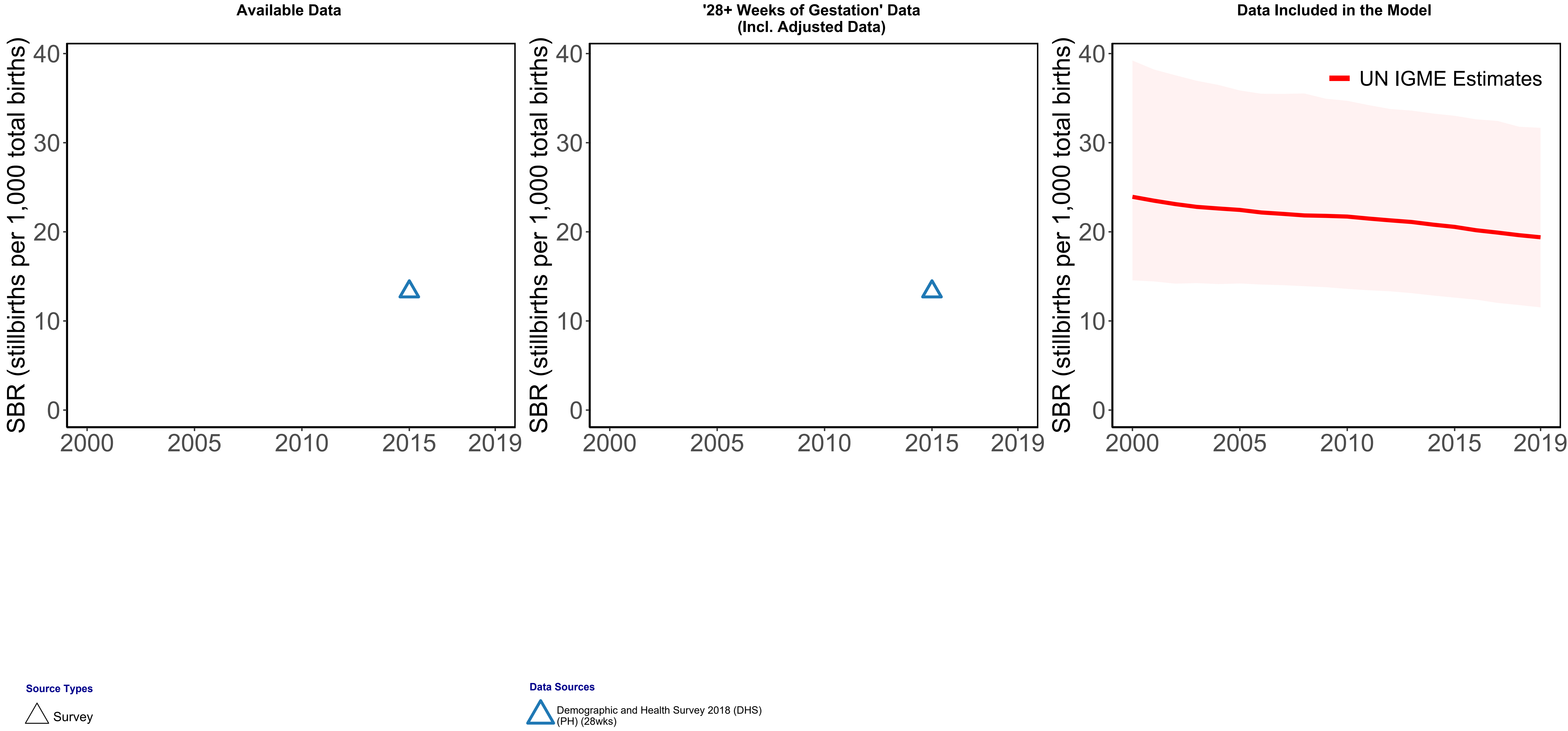
Available Data
'28+ Weeks of Gestation' Data
(Incl. Adjusted Data)
Data Included in the Model
SBR (stillbirths per 1,000 total births)
40
30
20
10
0
2000
2005
2010
2015
2019
UN IGME Estimates
Source Types
Survey
Data Sources
Demographic and Health Survey 2018 (DHS)
(PH) (28wks)

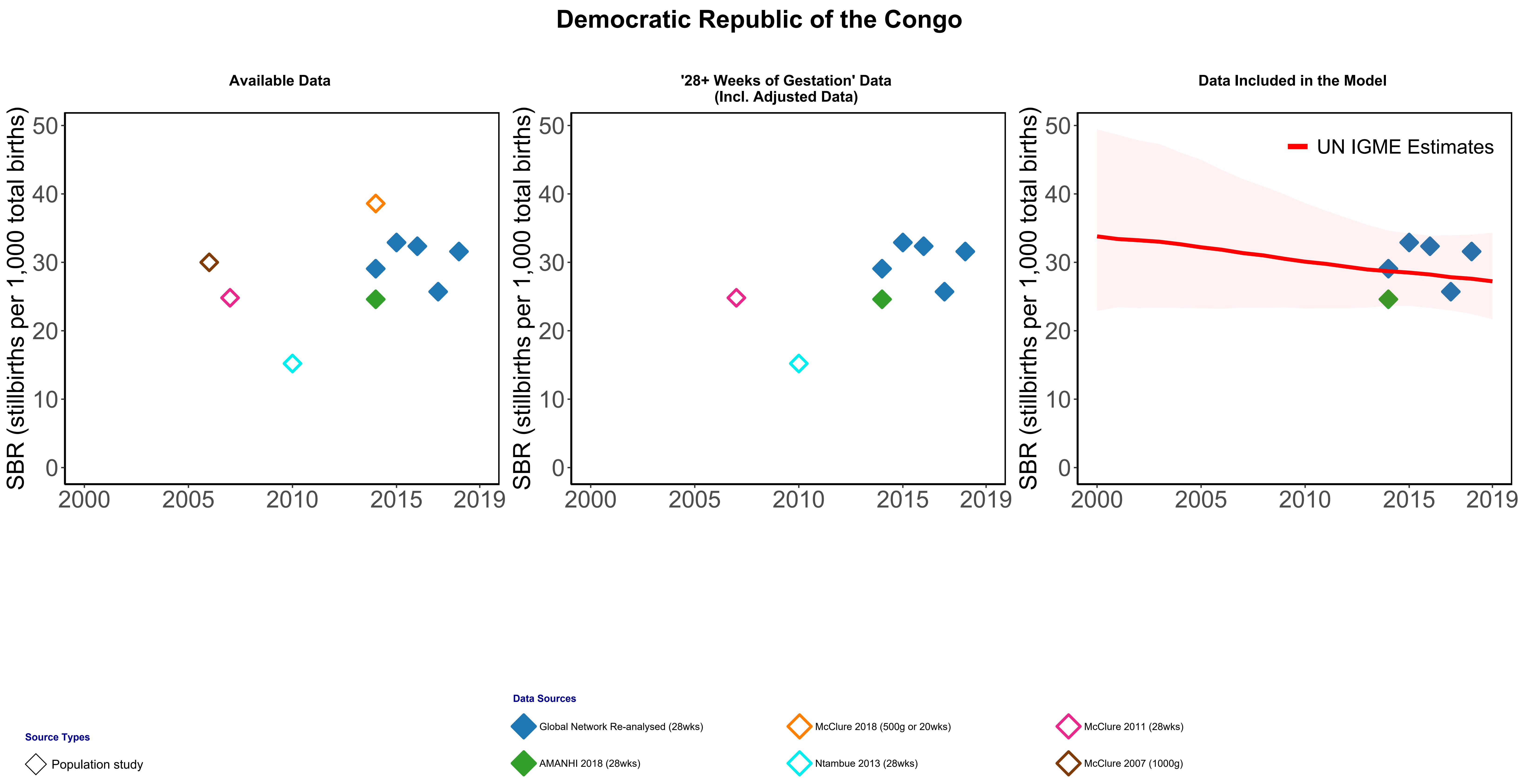

Democratic Republic of the Congo
Available Data
'28+ Weeks of Gestation' Data
(Incl. Adjusted Data)
Data Included in the Model
SBR (stillbirths per 1,000 total births)
0
10
20
30
40
50
2000
2005
2010
2015
2019
UN IGME Estimates
Source Types
Population study
Data Sources
Global Network Re-analysed (28wks)
AMANHI 2018 (28wks)
McClure 2018 (500g or 20wks)
Ntambue 2013 (28wks)
McClure 2011 (28wks)
McClure 2007 (1000g)

Congo

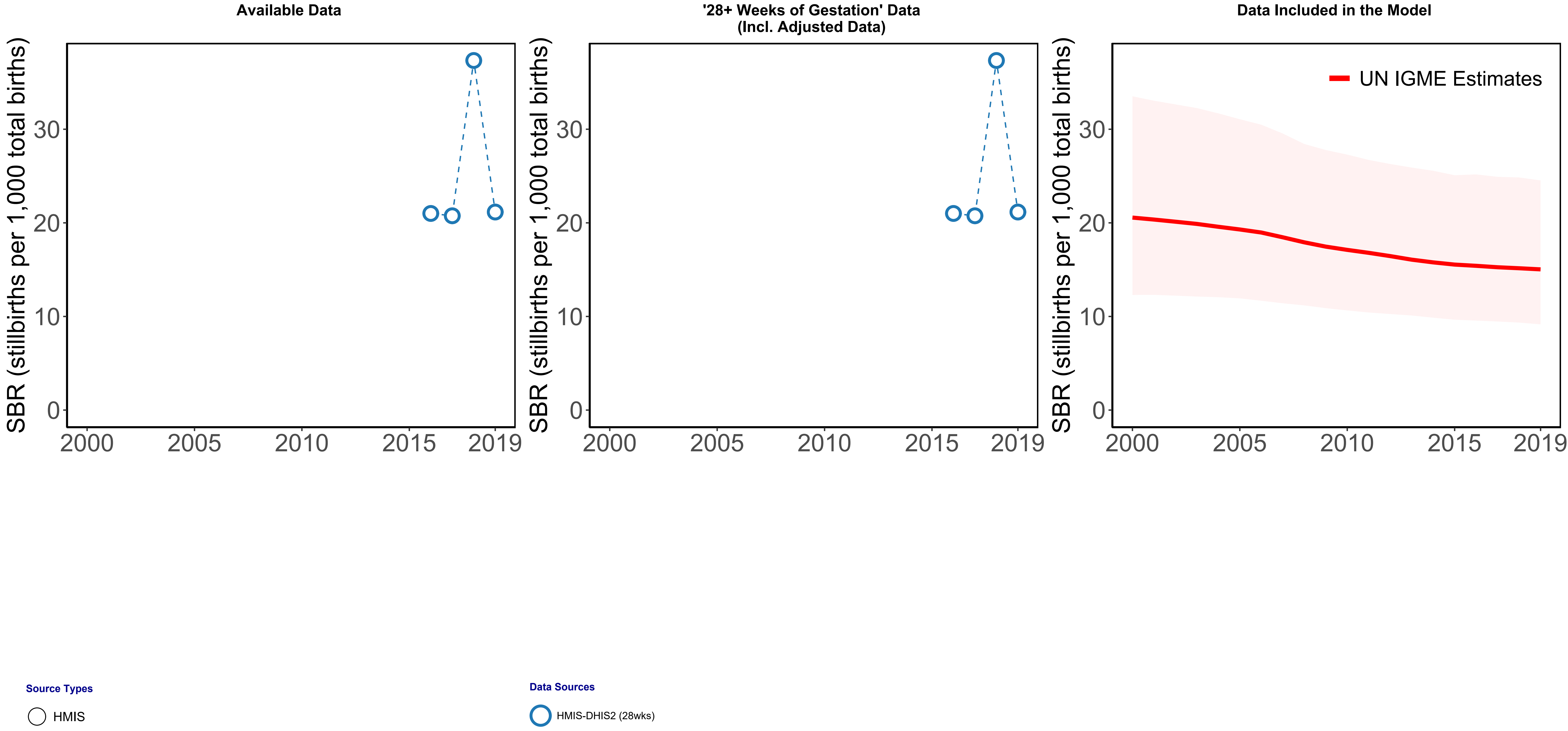
Available Data
'28+ Weeks of Gestation' Data
(Incl. Adjusted Data)
Data Included in the Model
SBR (stillbirths per 1,000 total births)
0
10
20
30
2000
2005
2010
2015
2019
UN IGME Estimates
Source Types
HMIS
Data Sources
HMIS-DHIS2 (28wks)

Cook Islands

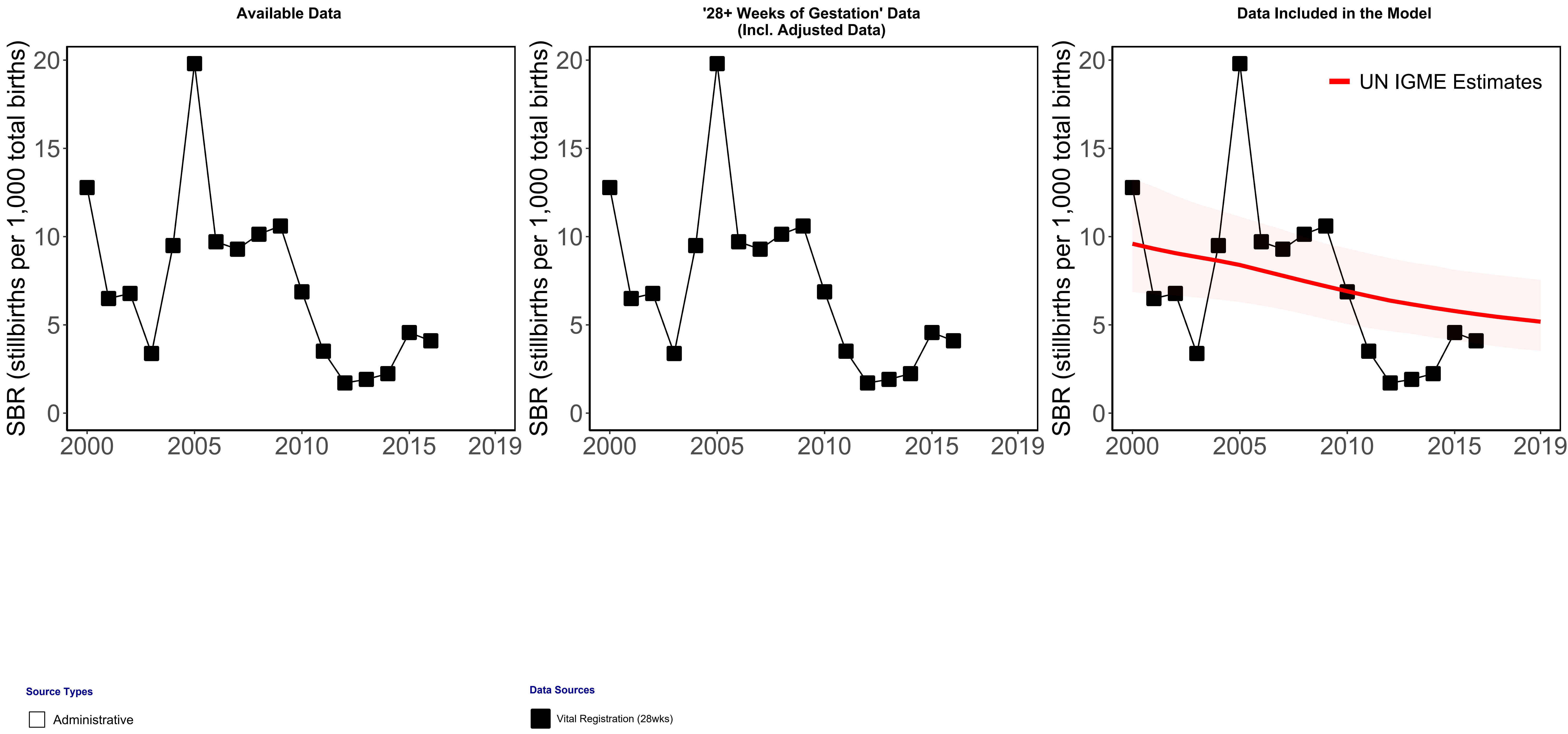

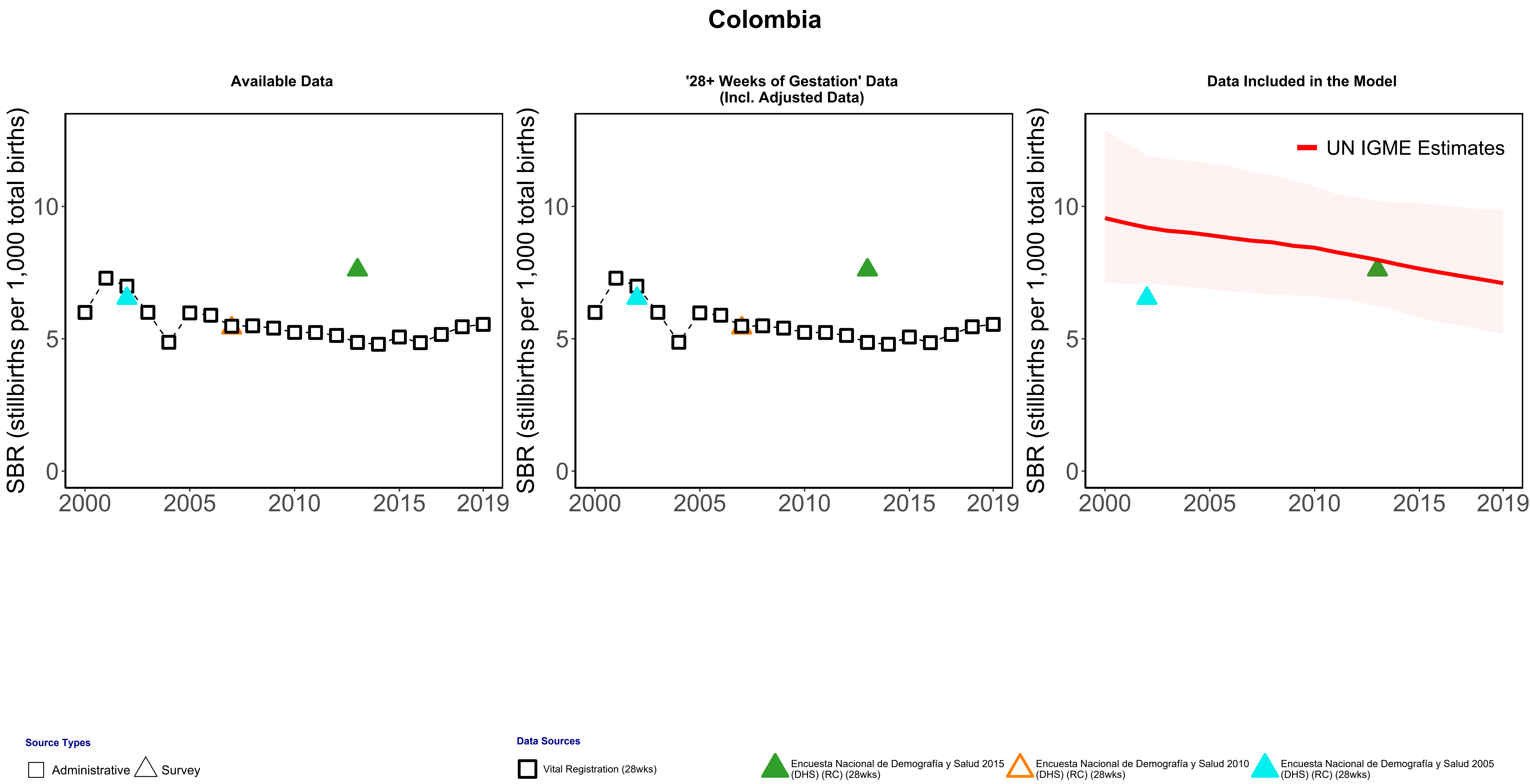
Colombia
Available Data
'28+ Weeks of Gestation' Data
(Incl. Adjusted Data)
Data Included in the Model
SBR (stillbirths per 1,000 total births)
UN IGME Estimates
0
5
10
2000
2005
2010
2015
2019
Source Types
Administrative
Survey
Data Sources
Vital Registration (28wks)
Encuesta Nacional de Demografía y Salud 2015 (DHS) (RC) (28wks)
Encuesta Nacional de Demografía y Salud 2010 (DHS) (RC) (28wks)
Encuesta Nacional de Demografía y Salud 2005 (DHS) (RC) (28wks)

Comoros

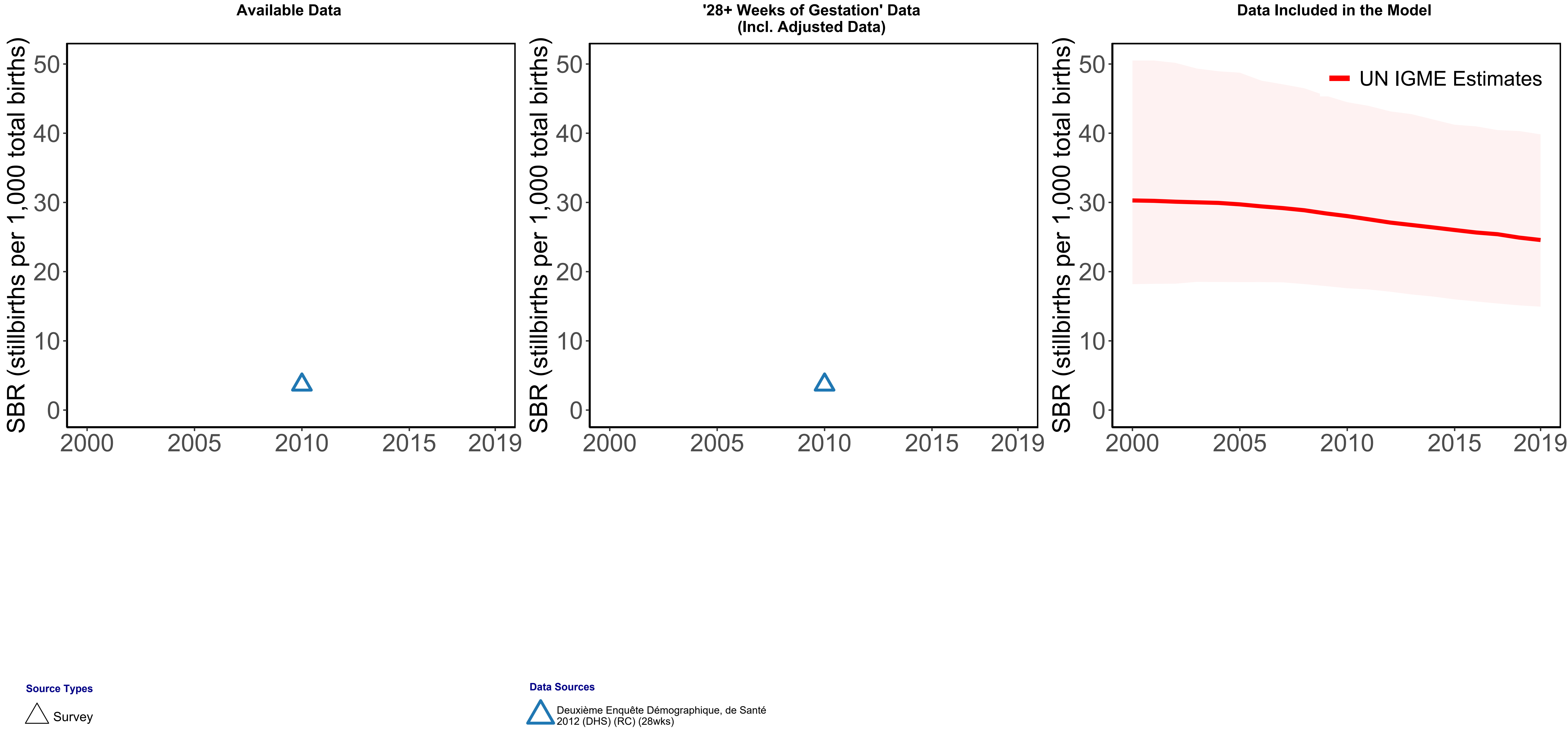
Available Data
'28+ Weeks of Gestation' Data
(Incl. Adjusted Data)
Data Included in the Model
SBR (stillbirths per 1,000 total births)
0
10
20
30
40
50
2000
2005
2010
2015
2019
UN IGME Estimates
Source Types
Survey
Data Sources
Deuxième Enquête Démographique, de Santé
2012 (DHS) (RC) (28wks)

Cape Verde

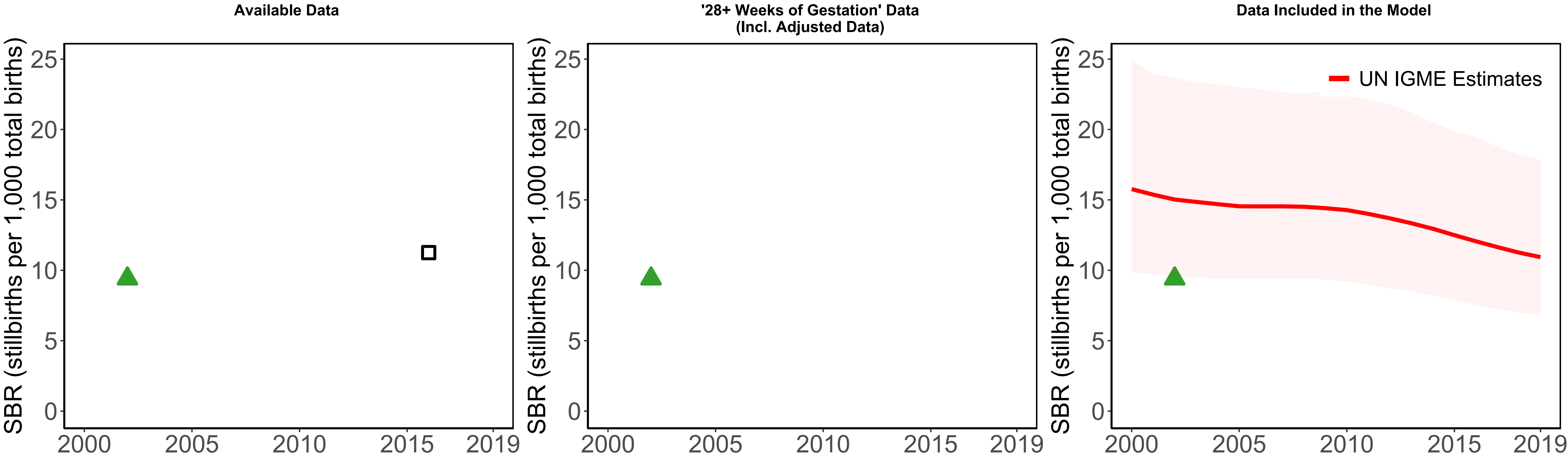

Source Types

Administrative, Survey

Data Sources

Vital Registration (any gestational age or birthweight)

II Inquérito Demográfico e de Saúde Reproductiva 2005 (DHS) (RC) (28wks)

Costa Rica

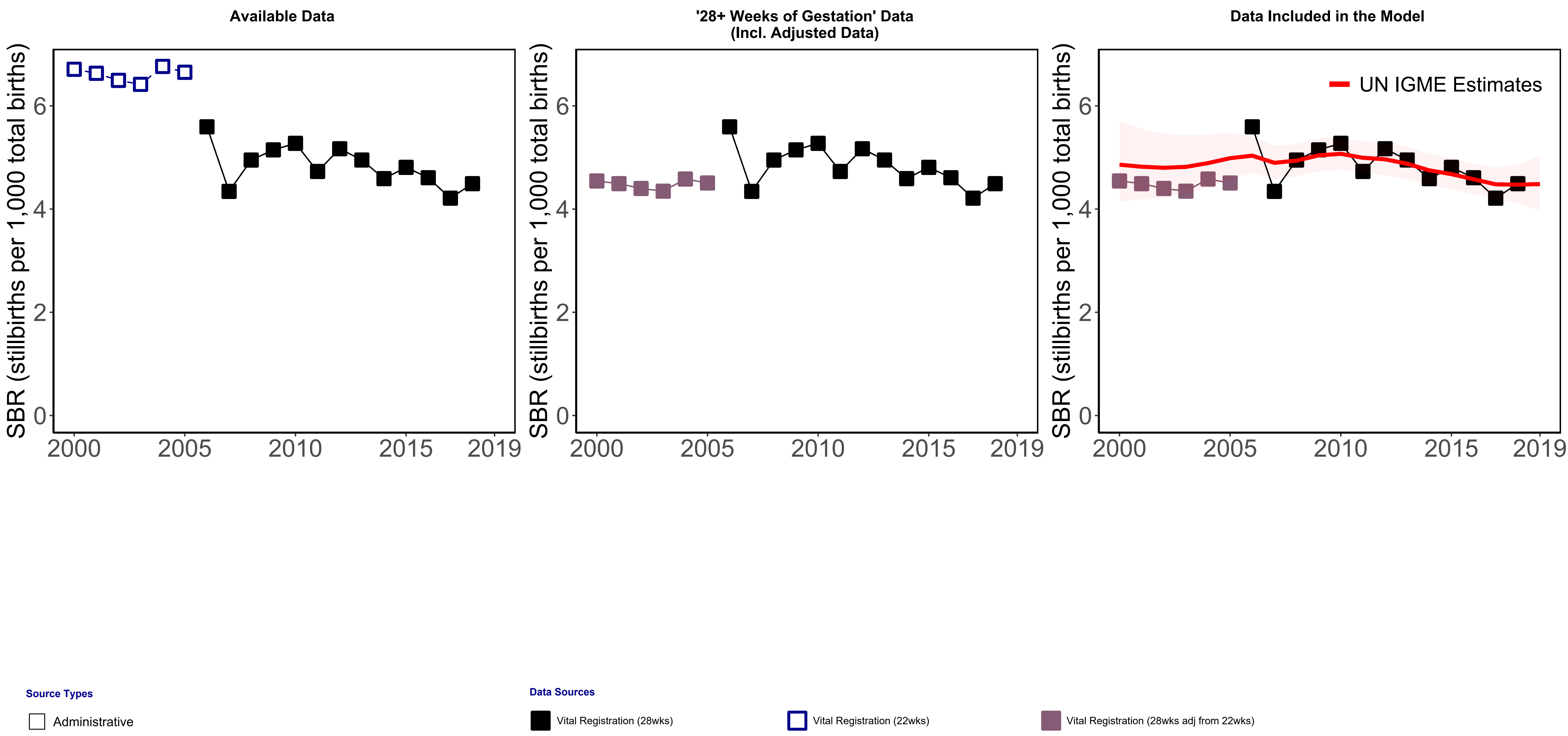
Available Data
'28+ Weeks of Gestation' Data
(Incl. Adjusted Data)
Data Included in the Model
UN IGME Estimates
SBR (stillbirths per 1,000 total births)
0
2
4
6
2000
2005
2010
2015
2019
Source Types
Administrative
Data Sources
Vital Registration (28wks)
Vital Registration (22wks)
Vital Registration (28wks adj from 22wks)

Cuba

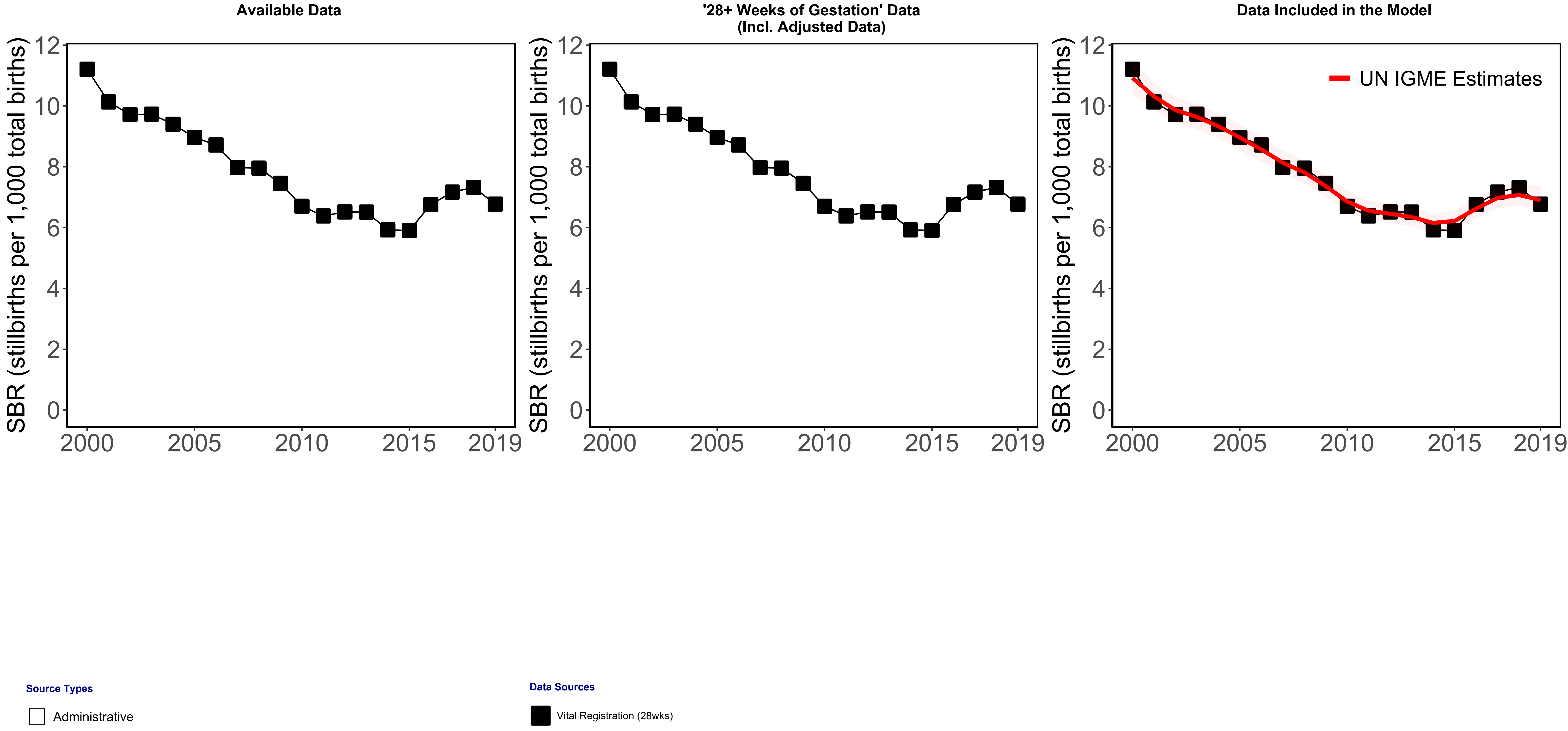
Available Data
'28+ Weeks of Gestation' Data
(Incl. Adjusted Data)
Data Included in the Model
SBR (stillbirths per 1,000 total births)
UN IGME Estimates
0
2
4
6
8
10
12
2000
2005
2010
2015
2019
Source Types
Administrative
Data Sources
Vital Registration (28wks)

Cyprus

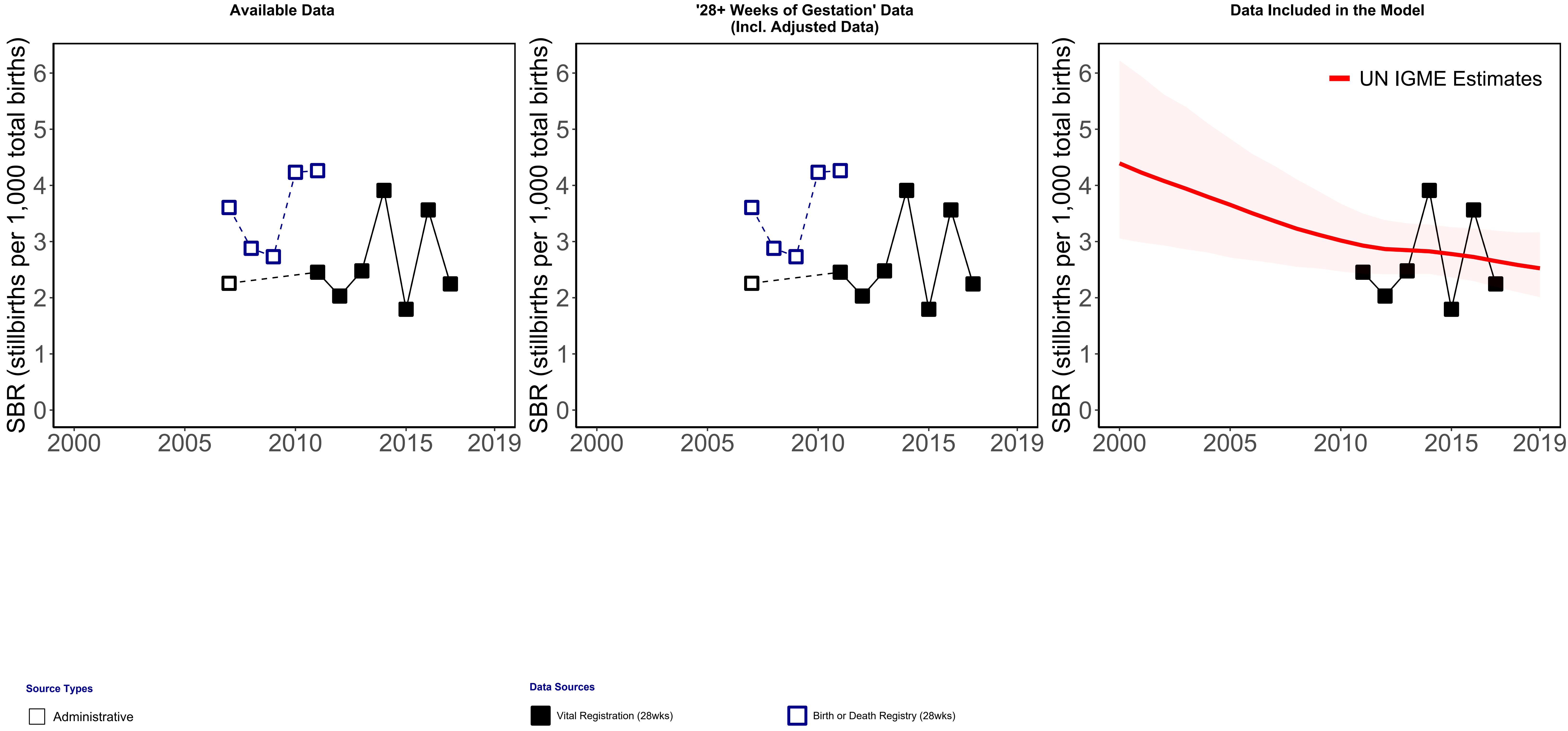
Available Data
'28+ Weeks of Gestation' Data (Incl. Adjusted Data)
Data Included in the Model
SBR (stillbirths per 1,000 total births)
0
1
2
3
4
5
6
2000
2005
2010
2015
2019
UN IGME Estimates
Source Types
Administrative
Data Sources
Vital Registration (28wks)
Birth or Death Registry (28wks)

Czechia

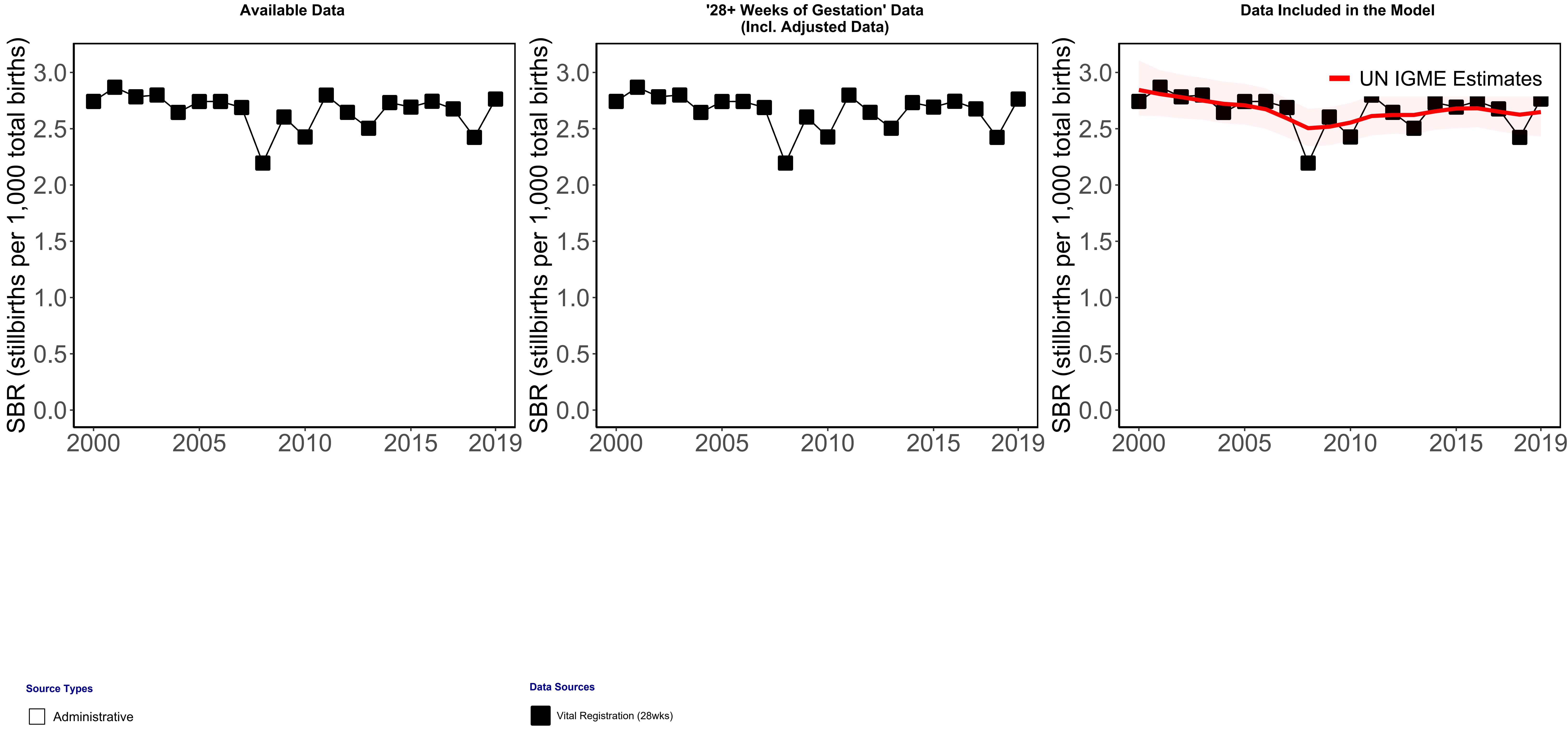
Available Data
'28+ Weeks of Gestation' Data
(Incl. Adjusted Data)
Data Included in the Model
SBR (stillbirths per 1,000 total births)
0.0
0.5
1.0
1.5
2.0
2.5
3.0
2000
2005
2010
2015
2019
UN IGME Estimates
Source Types
Administrative
Data Sources
Vital Registration (28wks)

Germany

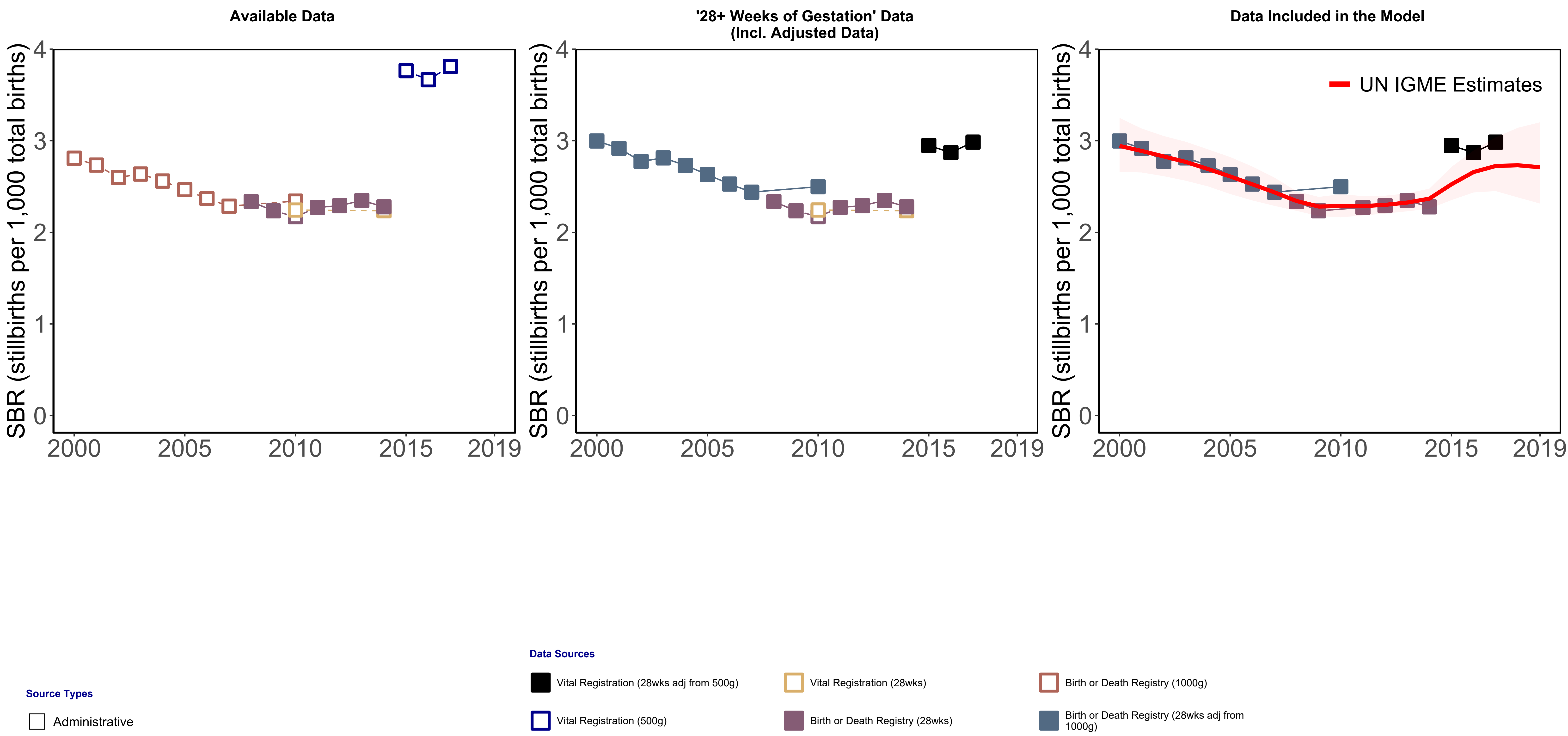

Available Data
'28+ Weeks of Gestation' Data
(Incl. Adjusted Data)
Data Included in the Model
SBR (stillbirths per 1,000 total births)
UN IGME Estimates
Source Types
Administrative
Data Sources
Vital Registration (28wks adj from 500g)
Vital Registration (28wks)
Birth or Death Registry (1000g)
Vital Registration (500g)
Birth or Death Registry (28wks)
Birth or Death Registry (28wks adj from 1000g)

Djibouti

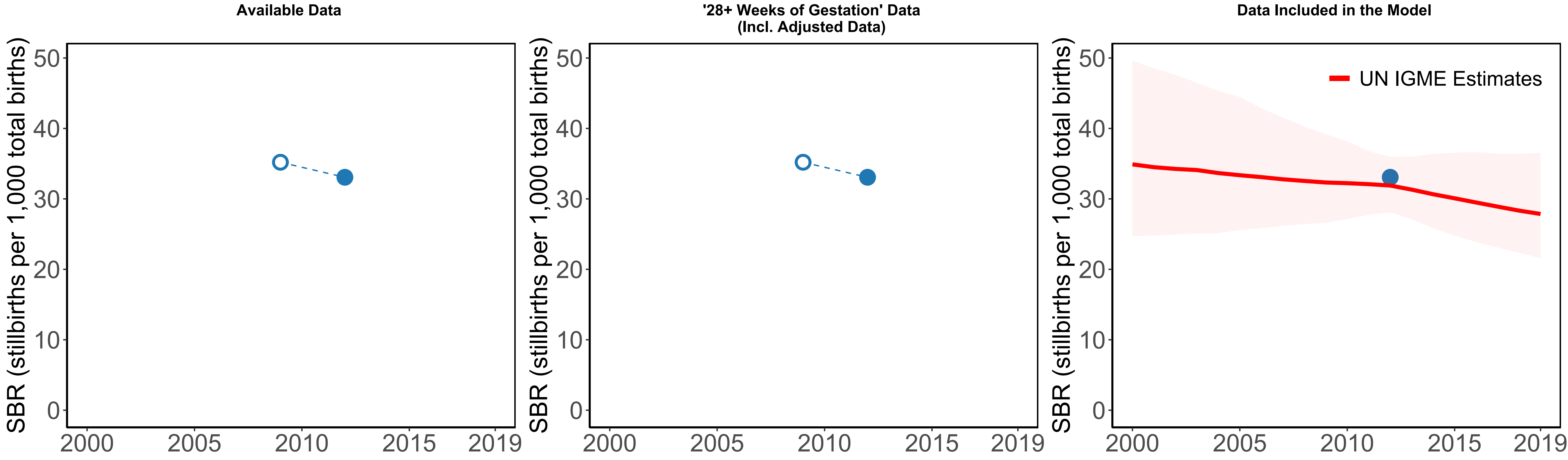

Source Types

HMIS

Data Sources

HMIS-DHIS2 (28wks)

Dominica

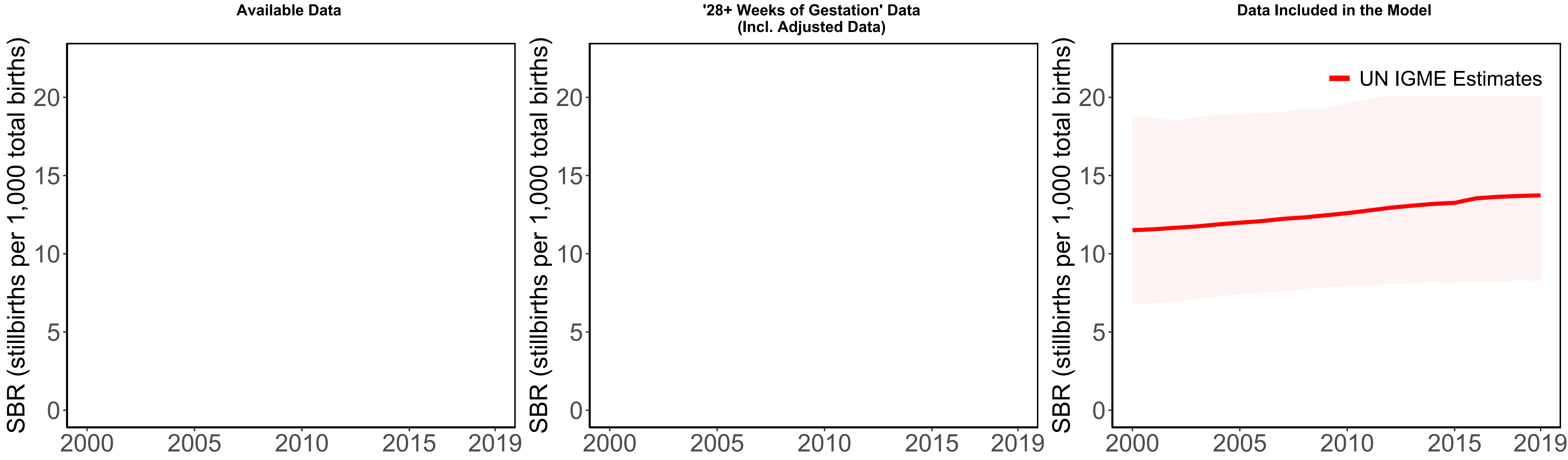

Available Data
'28+ Weeks of Gestation' Data
(Incl. Adjusted Data)
Data Included in the Model
UN IGME Estimates
SBR (stillbirths per 1,000 total births)
0
5
10
15
20
2000
2005
2010
2015
2019

Denmark

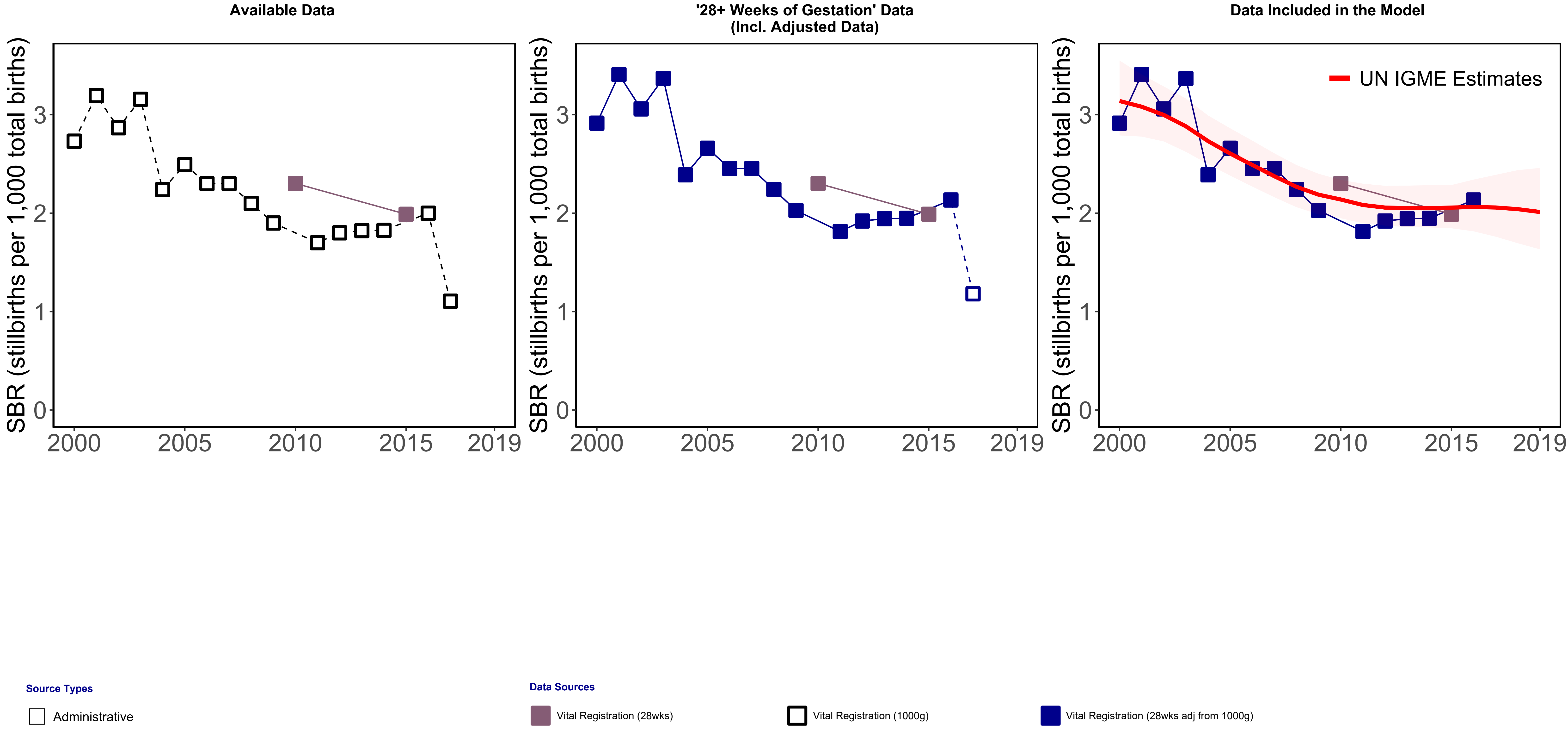

Available Data
'28+ Weeks of Gestation' Data
(Incl. Adjusted Data)
Data Included in the Model
SBR (stillbirths per 1,000 total births)
UN IGME Estimates
0
1
2
3
2000
2005
2010
2015
2019
Source Types
Administrative
Data Sources
Vital Registration (28wks)
Vital Registration (1000g)
Vital Registration (28wks adj from 1000g)

Dominican Republic

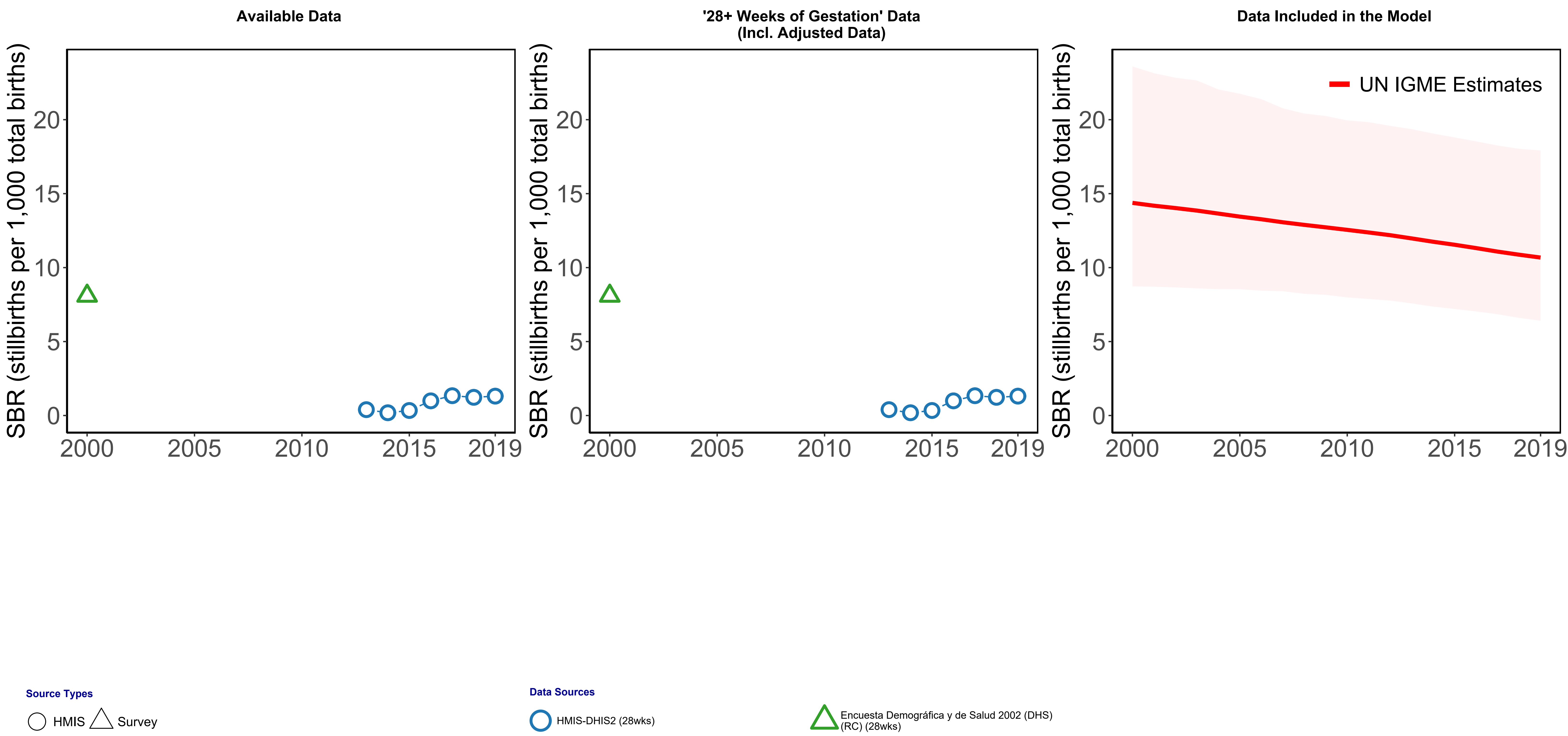

Algeria

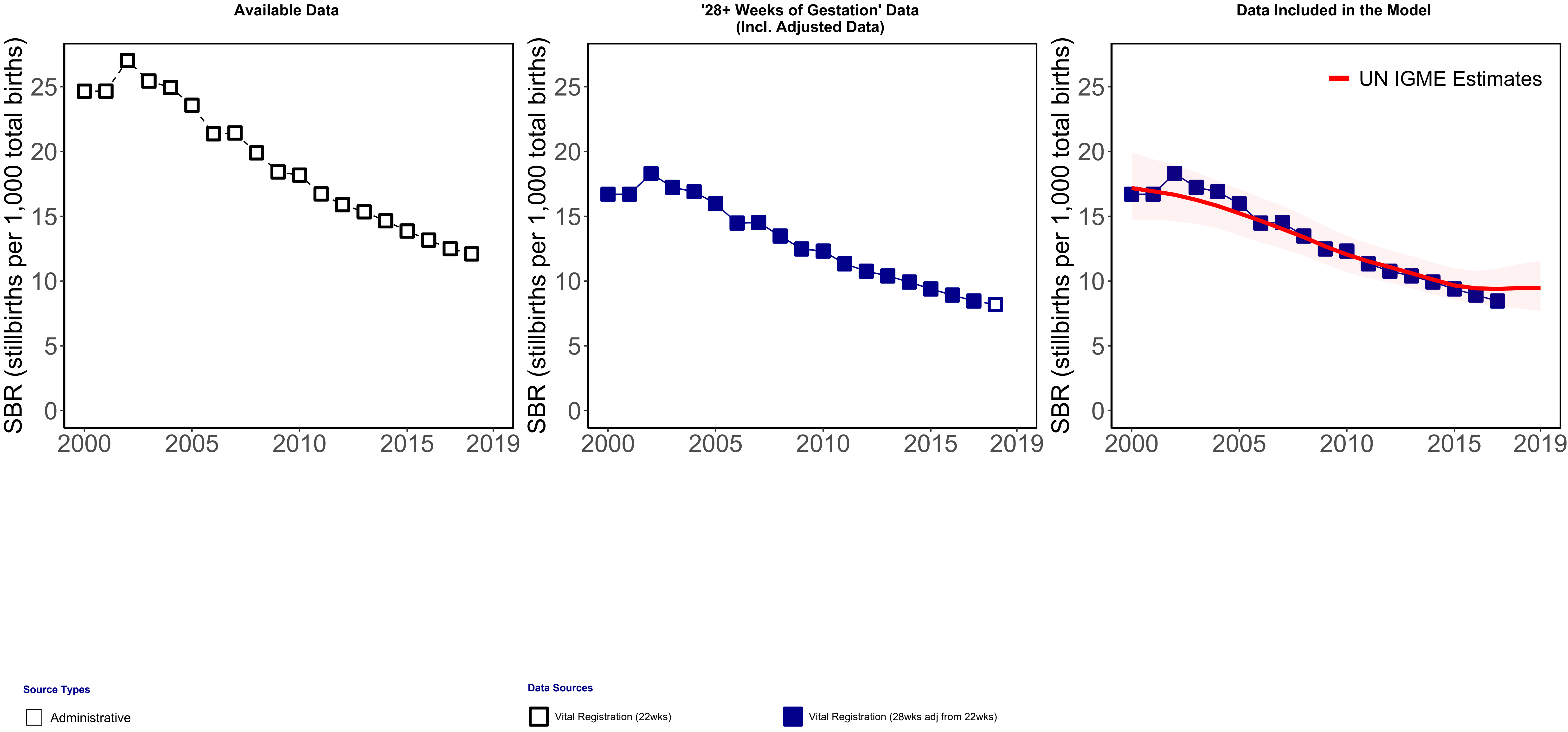

Available Data
'28+ Weeks of Gestation' Data
(Incl. Adjusted Data)
Data Included in the Model
SBR (stillbirths per 1,000 total births)
UN IGME Estimates
0
5
10
15
20
25
2000
2005
2010
2015
2019
Source Types
Administrative
Data Sources
Vital Registration (22wks)
Vital Registration (28wks adj from 22wks)

Ecuador

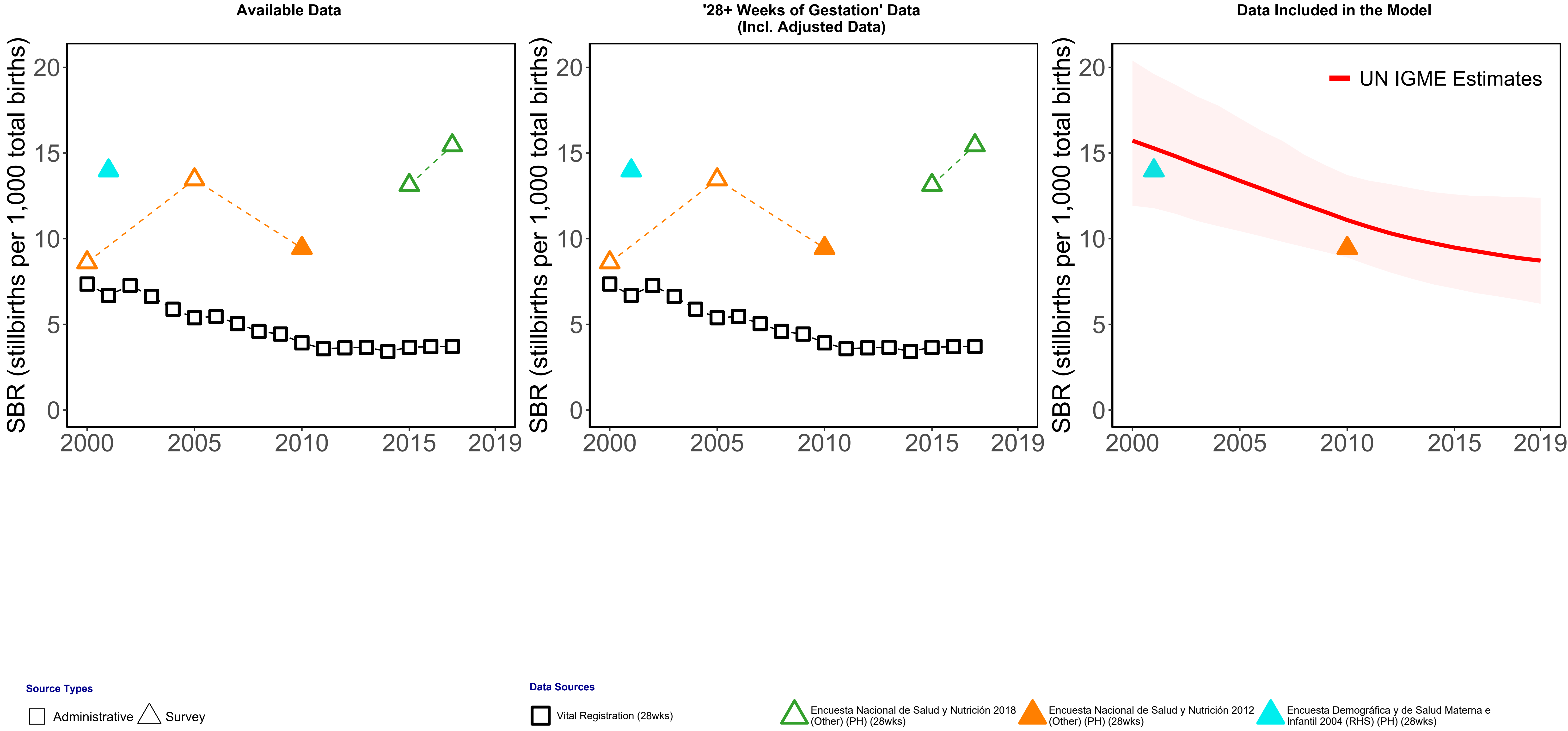
Available Data
'28+ Weeks of Gestation' Data
(Incl. Adjusted Data)
Data Included in the Model
SBR (stillbirths per 1,000 total births)
0
5
10
15
20
2000
2005
2010
2015
2019
UN IGME Estimates
Source Types
Administrative
Survey
Data Sources
Vital Registration (28wks)
Encuesta Nacional de Salud y Nutrición 2018 (Other) (PH) (28wks)
Encuesta Nacional de Salud y Nutrición 2012 (Other) (PH) (28wks)
Encuesta Demográfica y de Salud Materna e Infantil 2004 (RHS) (PH) (28wks)

Egypt

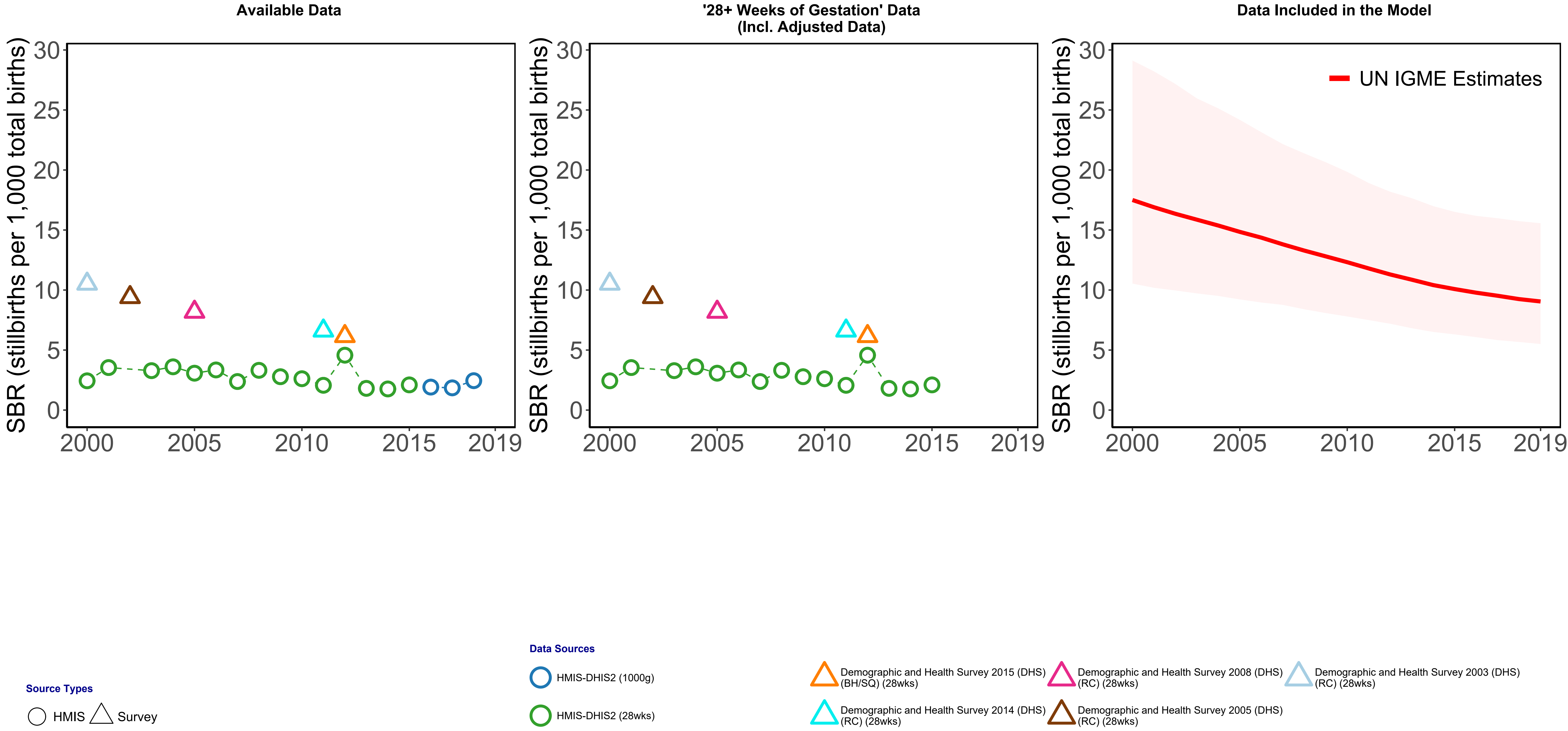

Available Data
'28+ Weeks of Gestation' Data
(Incl. Adjusted Data)
Data Included in the Model
SBR (stillbirths per 1,000 total births)
30
25
20
15
10
5
0
2000
2005
2010
2015
2019
UN IGME Estimates
Data Sources
HMIS-DHIS2 (1000g)
HMIS-DHIS2 (28wks)
Demographic and Health Survey 2015 (DHS) (BH/SQ) (28wks)
Demographic and Health Survey 2014 (DHS) (RC) (28wks)
Demographic and Health Survey 2008 (DHS) (RC) (28wks)
Demographic and Health Survey 2005 (DHS) (RC) (28wks)
Demographic and Health Survey 2003 (DHS) (RC) (28wks)
Source Types
HMIS
Survey

Eritrea

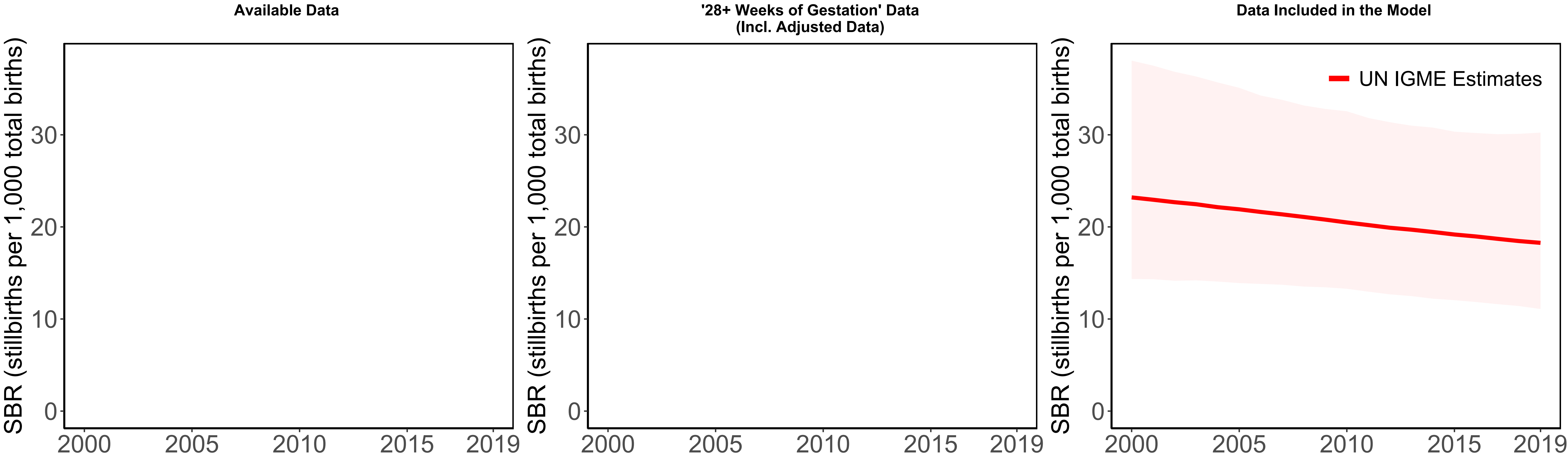

Available Data
'28+ Weeks of Gestation' Data
(Incl. Adjusted Data)
Data Included in the Model
UN IGME Estimates
SBR (stillbirths per 1,000 total births)
0
10
20
30
2000
2005
2010
2015
2019

Spain

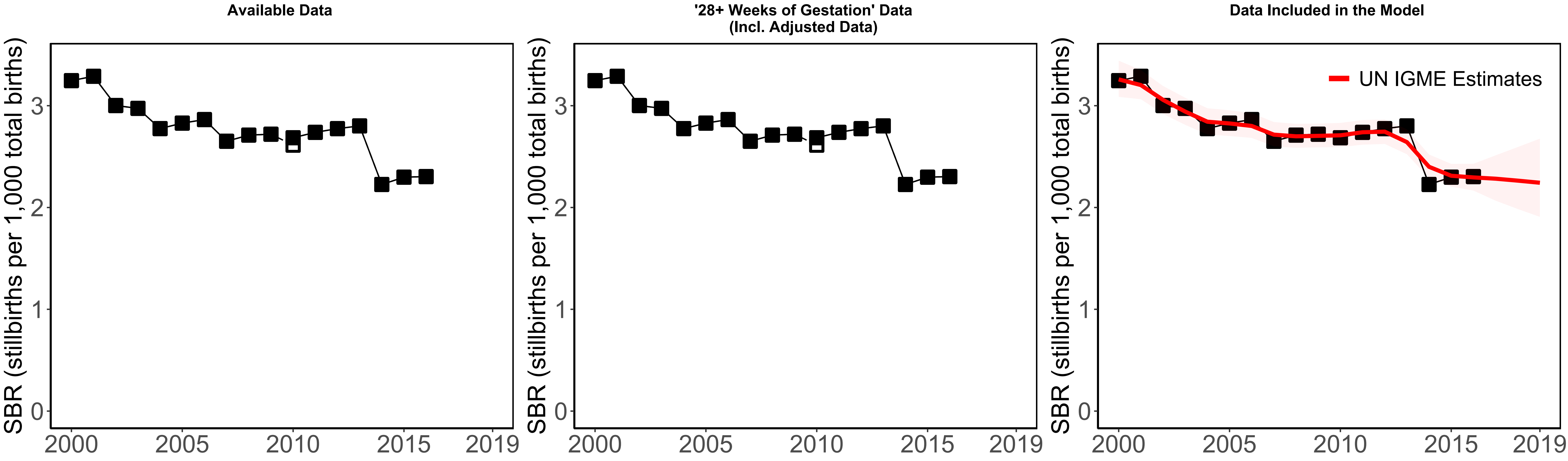

Source Types

Administrative

Data Sources

Vital Registration (28wks)

Estonia

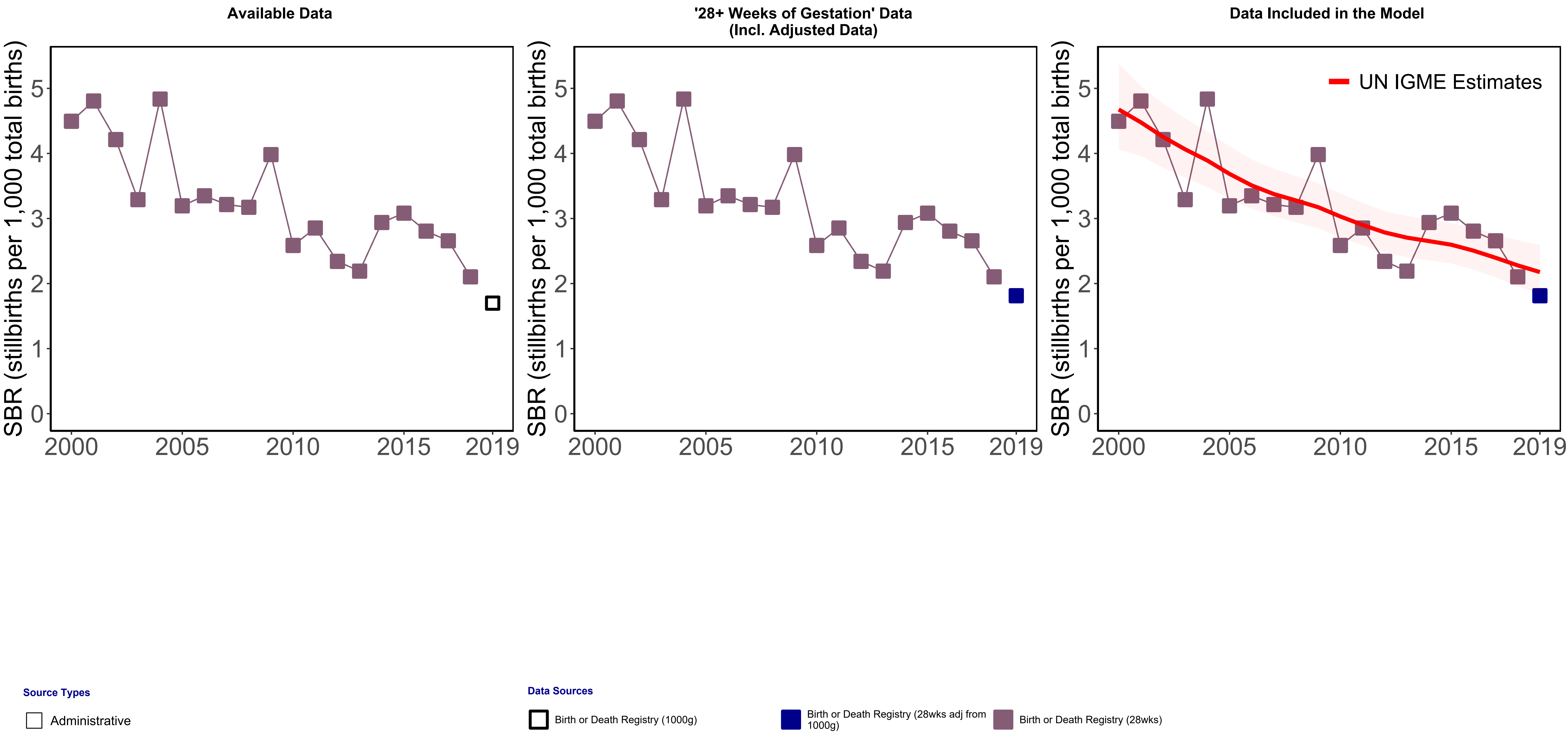
Available Data
'28+ Weeks of Gestation' Data
(Incl. Adjusted Data)
Data Included in the Model
UN IGME Estimates
SBR (stillbirths per 1,000 total births)
0
1
2
3
4
5
2000
2005
2010
2015
2019
Source Types
Administrative
Data Sources
Birth or Death Registry (1000g)
Birth or Death Registry (28wks adj from 1000g)
Birth or Death Registry (28wks)

Ethiopia

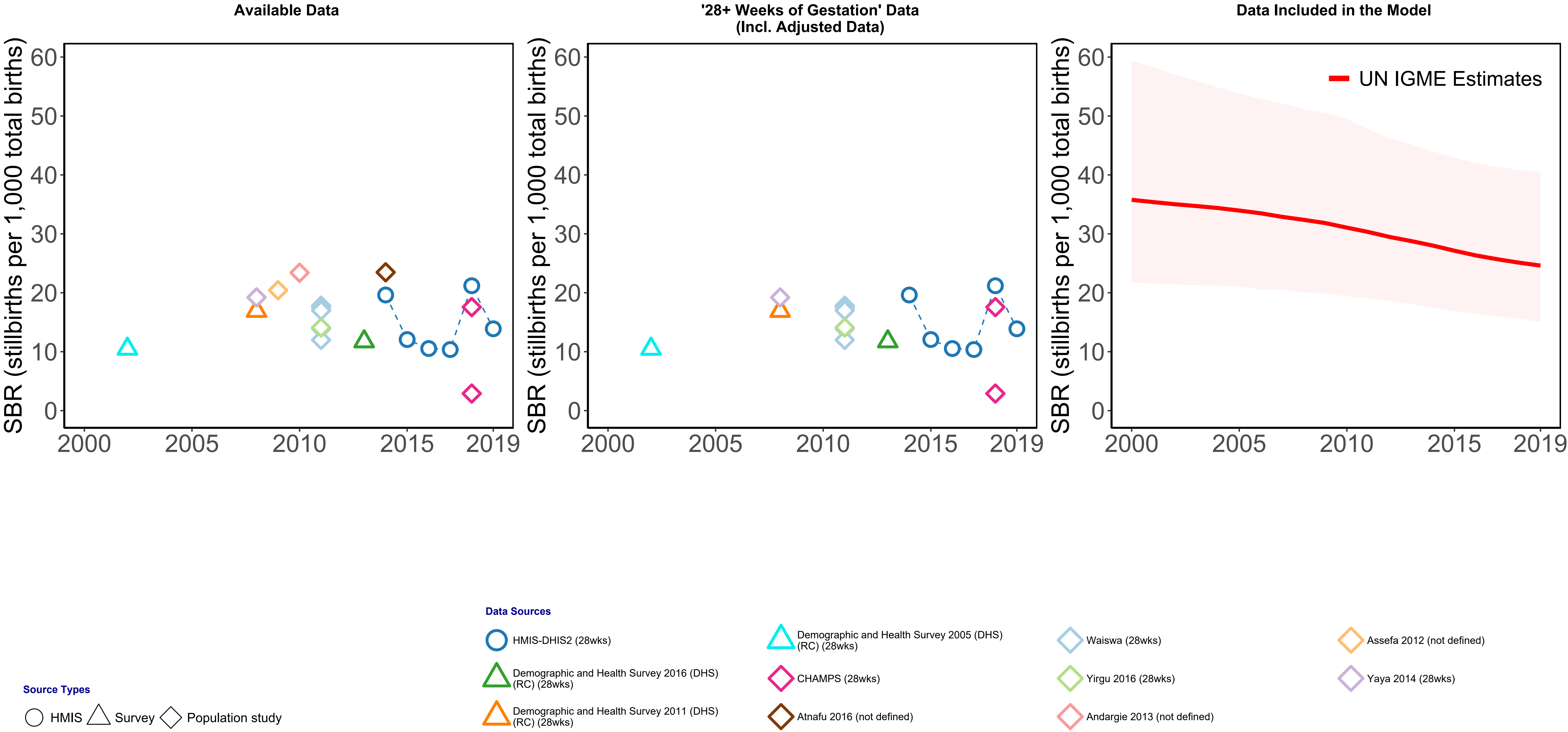

Available Data
'28+ Weeks of Gestation' Data
(Incl. Adjusted Data)
Data Included in the Model
SBR (stillbirths per 1,000 total births)
0
10
20
30
40
50
60
2000
2005
2010
2015
2019
UN IGME Estimates
Data Sources
HMIS-DHIS2 (28wks)
Demographic and Health Survey 2005 (DHS) (RC) (28wks)
Waiswa (28wks)
Assefa 2012 (not defined)
Demographic and Health Survey 2016 (DHS) (RC) (28wks)
CHAMPS (28wks)
Yirgu 2016 (28wks)
Yaya 2014 (28wks)
Demographic and Health Survey 2011 (DHS) (RC) (28wks)
Atnafu 2016 (not defined)
Andargie 2013 (not defined)
Source Types
HMIS
Survey
Population study

Finland

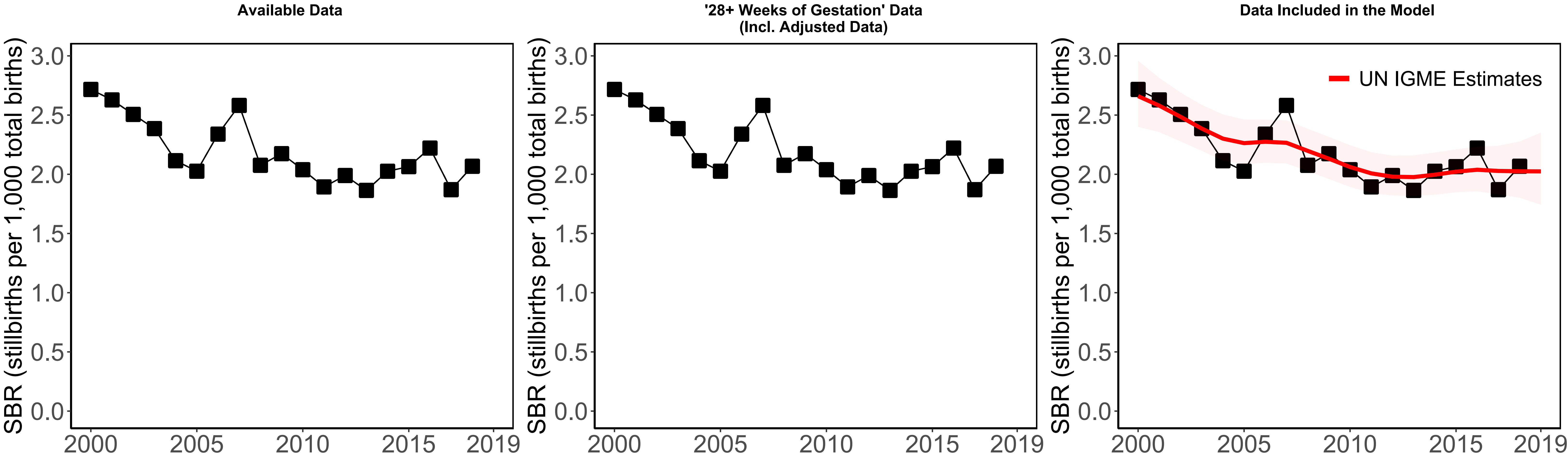

Available Data
'28+ Weeks of Gestation' Data
(Incl. Adjusted Data)
Data Included in the Model
UN IGME Estimates
SBR (stillbirths per 1,000 total births)
3.0
2.5
2.0
1.5
1.0
0.5
0.0
2000
2005
2010
2015
2019

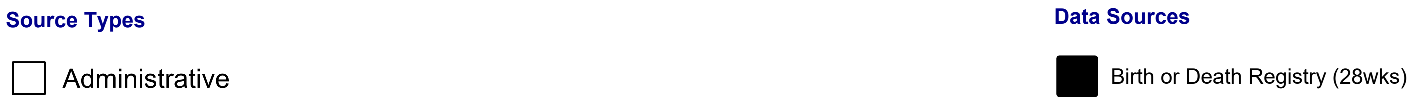

Source Types
Administrative
Data Sources
Birth or Death Registry (28wks)

Fiji

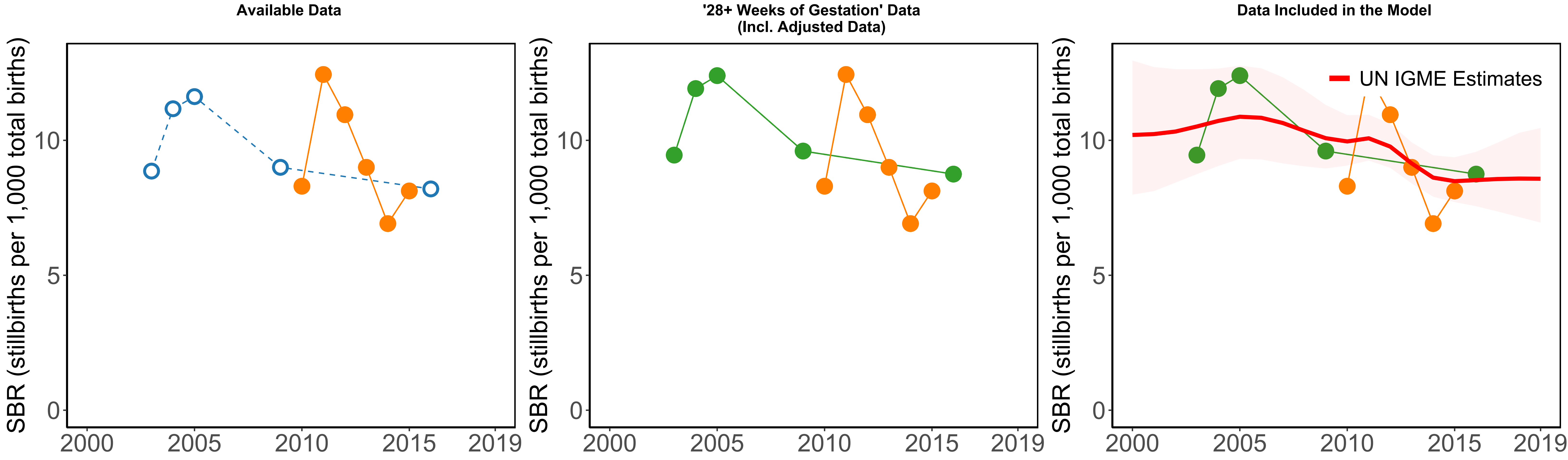

Source Types

HMIS

Data Sources

HMIS-DHIS2 (28wks)

HMIS-DHIS2 (1000g)

HMIS-DHIS2 (28wks adj from 1000g)

France

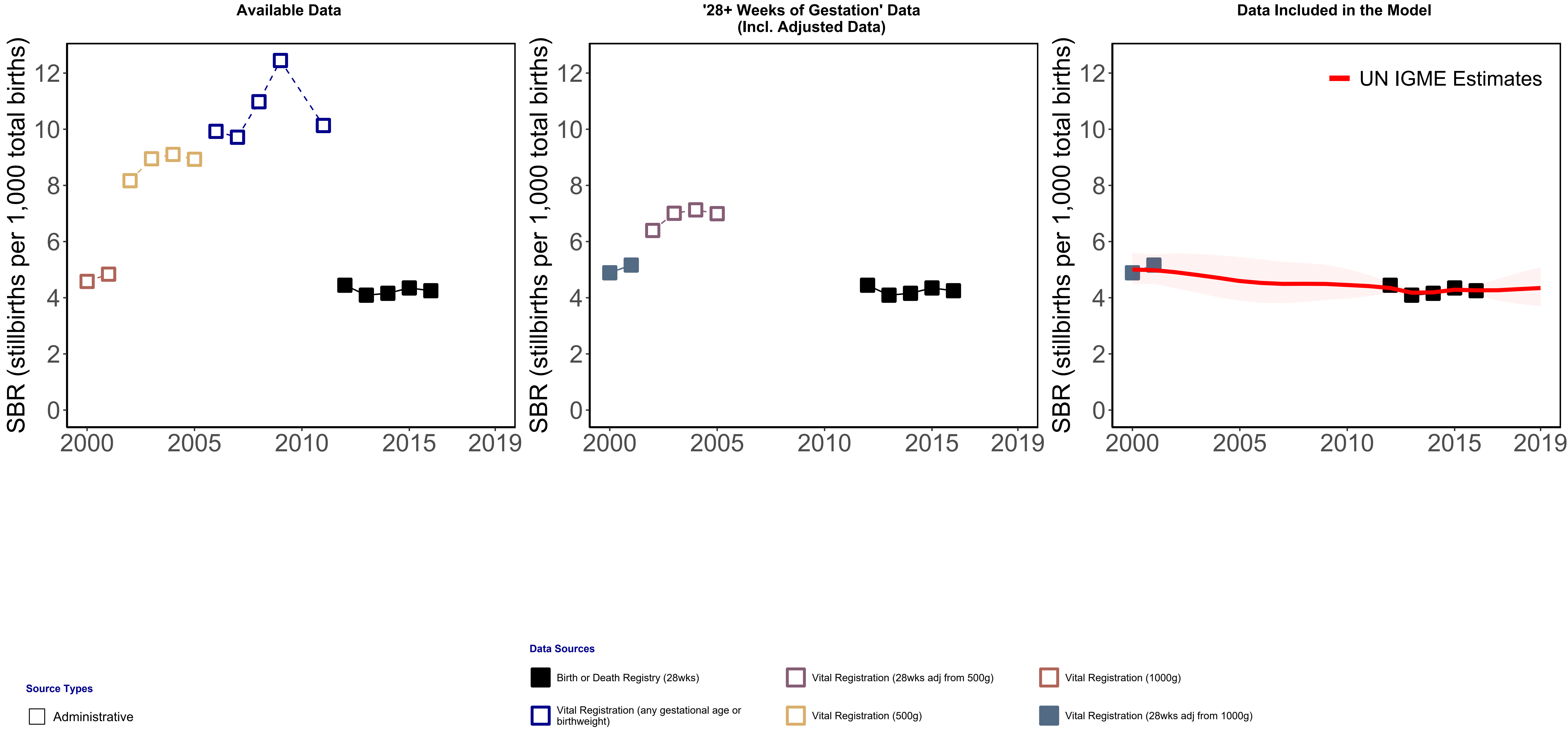
Available Data
'28+ Weeks of Gestation' Data
(Incl. Adjusted Data)
Data Included in the Model
SBR (stillbirths per 1,000 total births)
0
2
4
6
8
10
12
2000
2005
2010
2015
2019
UN IGME Estimates
Source Types
Administrative
Data Sources
Birth or Death Registry (28wks)
Vital Registration (any gestational age or birthweight)
Vital Registration (28wks adj from 500g)
Vital Registration (500g)
Vital Registration (1000g)
Vital Registration (28wks adj from 1000g)

Micronesia (Federated States of)

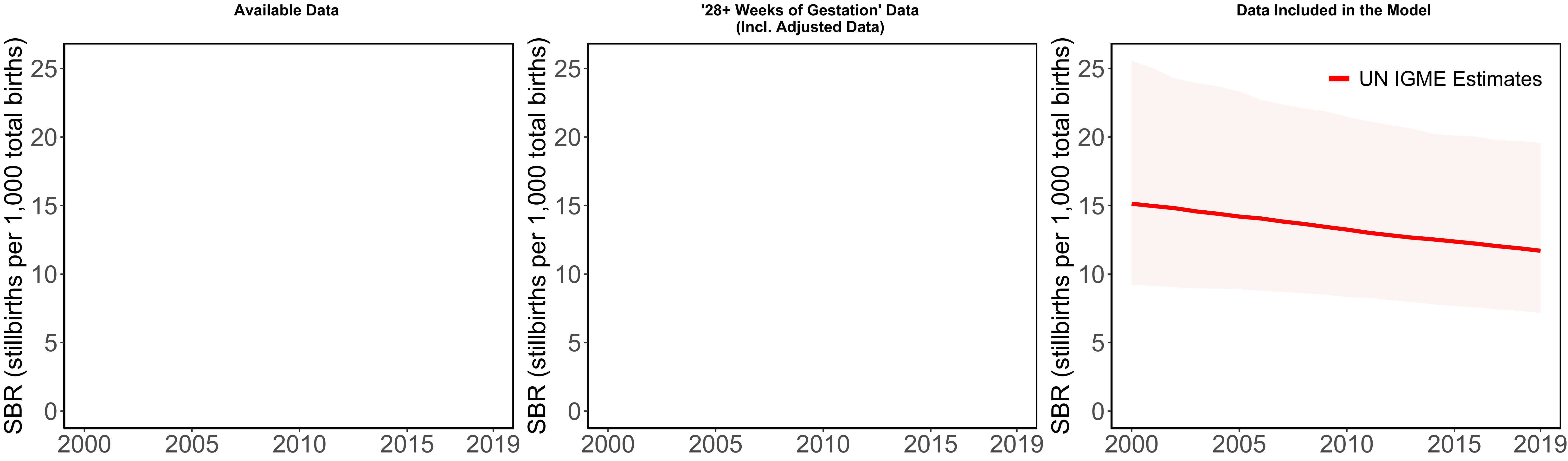

Gabon

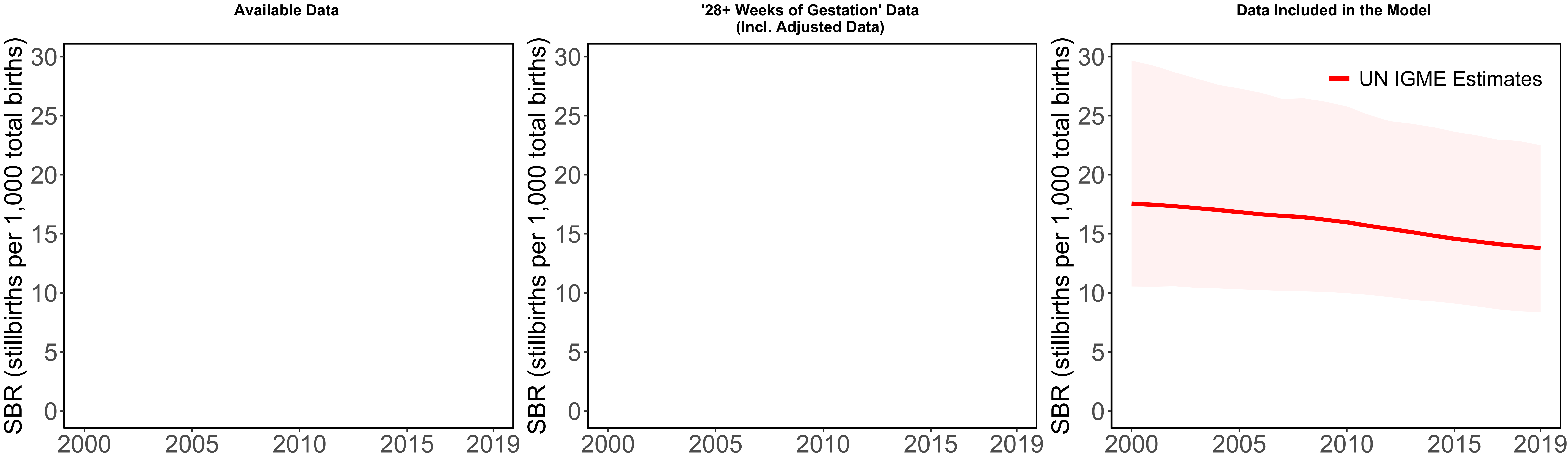

Available Data
'28+ Weeks of Gestation' Data
(Incl. Adjusted Data)
Data Included in the Model
SBR (stillbirths per 1,000 total births)
UN IGME Estimates
0
5
10
15
20
25
30
2000
2005
2010
2015
2019

United Kingdom

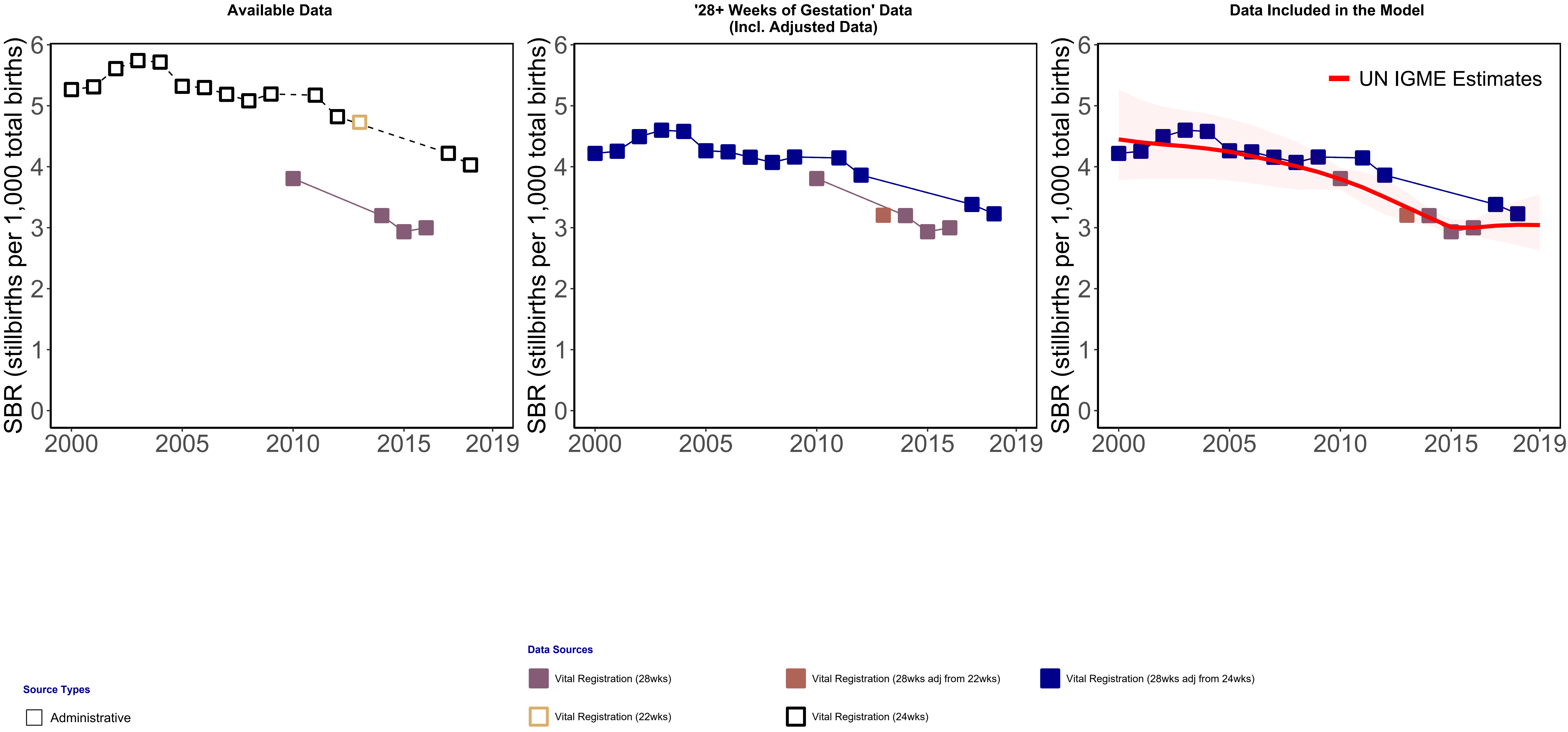

Georgia

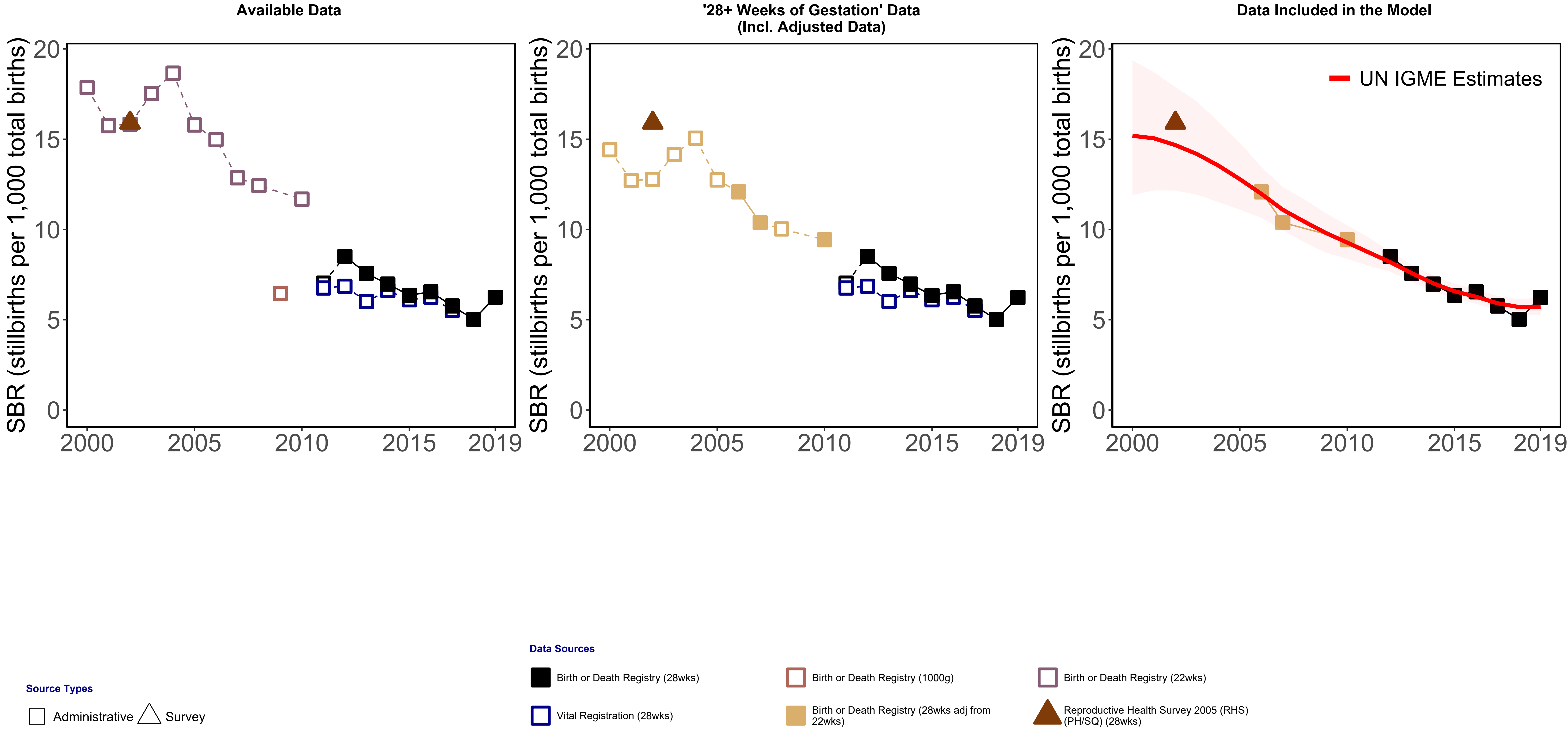

Available Data
'28+ Weeks of Gestation' Data
(Incl. Adjusted Data)
Data Included in the Model
SBR (stillbirths per 1,000 total births)
0
5
10
15
20
2000
2005
2010
2015
2019
UN IGME Estimates
Source Types
Administrative
Survey
Data Sources
Birth or Death Registry (28wks)
Birth or Death Registry (1000g)
Birth or Death Registry (22wks)
Vital Registration (28wks)
Birth or Death Registry (28wks adj from 22wks)
Reproductive Health Survey 2005 (RHS) (PH/SQ) (28wks)

Ghana

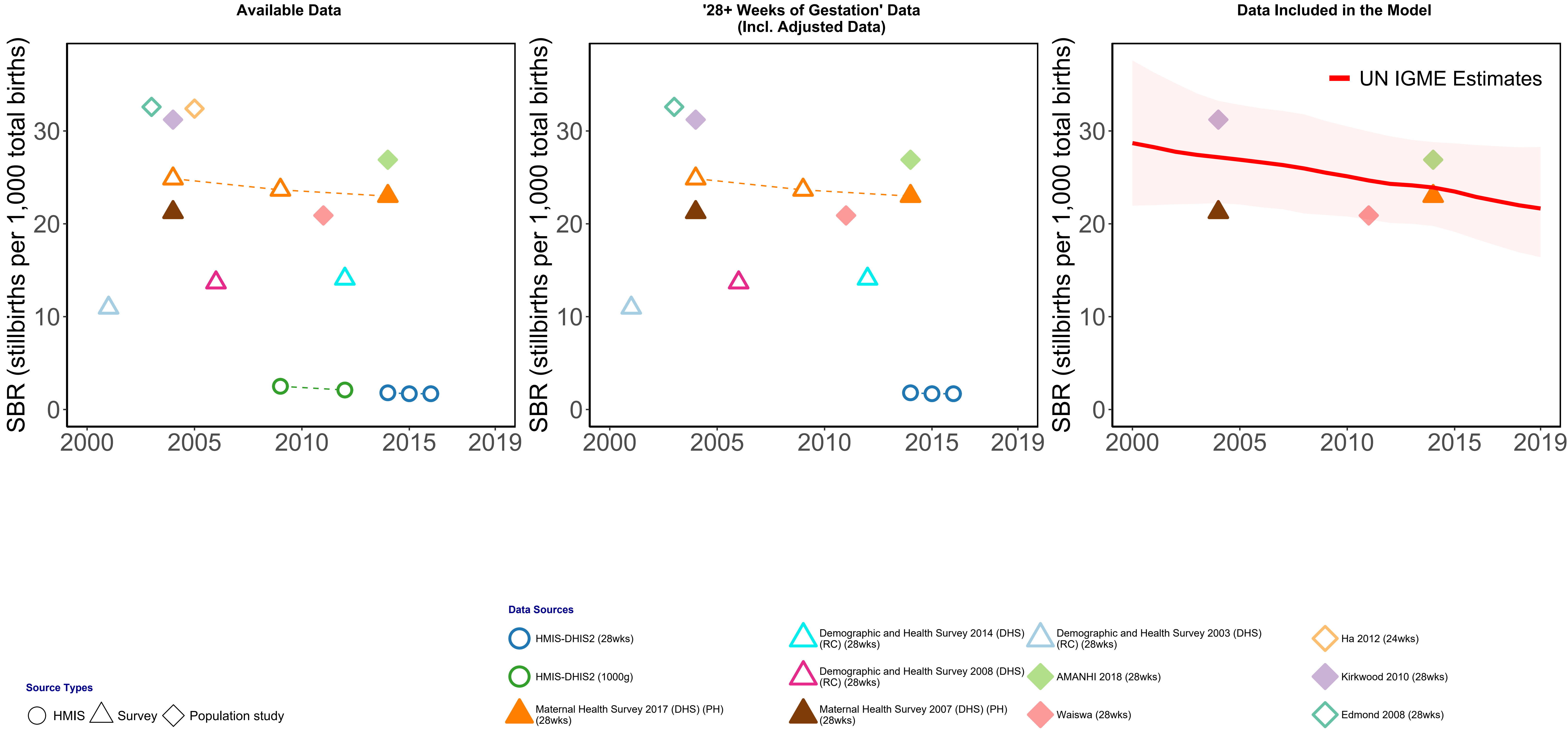

Available Data
'28+ Weeks of Gestation' Data
(Incl. Adjusted Data)
Data Included in the Model
SBR (stillbirths per 1,000 total births)
0
10
20
30
2000
2005
2010
2015
2019
UN IGME Estimates
Data Sources
HMIS-DHIS2 (28wks)
HMIS-DHIS2 (1000g)
Maternal Health Survey 2017 (DHS) (PH) (28wks)
Demographic and Health Survey 2014 (DHS) (RC) (28wks)
Demographic and Health Survey 2008 (DHS) (RC) (28wks)
Maternal Health Survey 2007 (DHS) (PH) (28wks)
Demographic and Health Survey 2003 (DHS) (RC) (28wks)
AMANHI 2018 (28wks)
Waiswa (28wks)
Ha 2012 (24wks)
Kirkwood 2010 (28wks)
Edmond 2008 (28wks)
Source Types
HMIS
Survey
Population study

Guinea

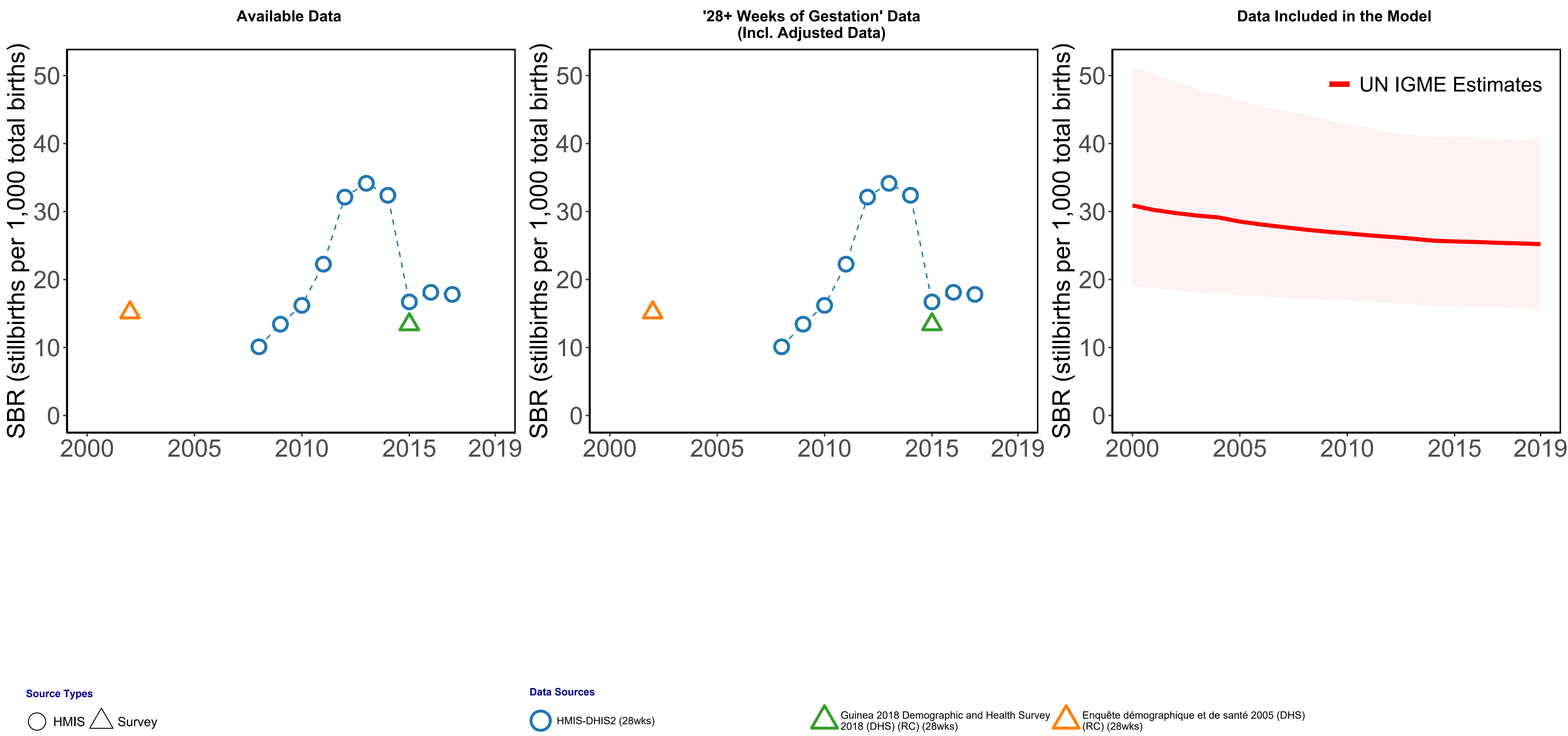

Available Data
'28+ Weeks of Gestation' Data (Incl. Adjusted Data)
Data Included in the Model
SBR (stillbirths per 1,000 total births)
0
10
20
30
40
50
2000
2005
2010
2015
2019
UN IGME Estimates
Source Types
HMIS
Survey
Data Sources
HMIS-DHIS2 (28wks)
Guinea 2018 Demographic and Health Survey 2018 (DHS) (RC) (28wks)
Enquête démographique et de santé 2005 (DHS) (RC) (28wks)

Gambia

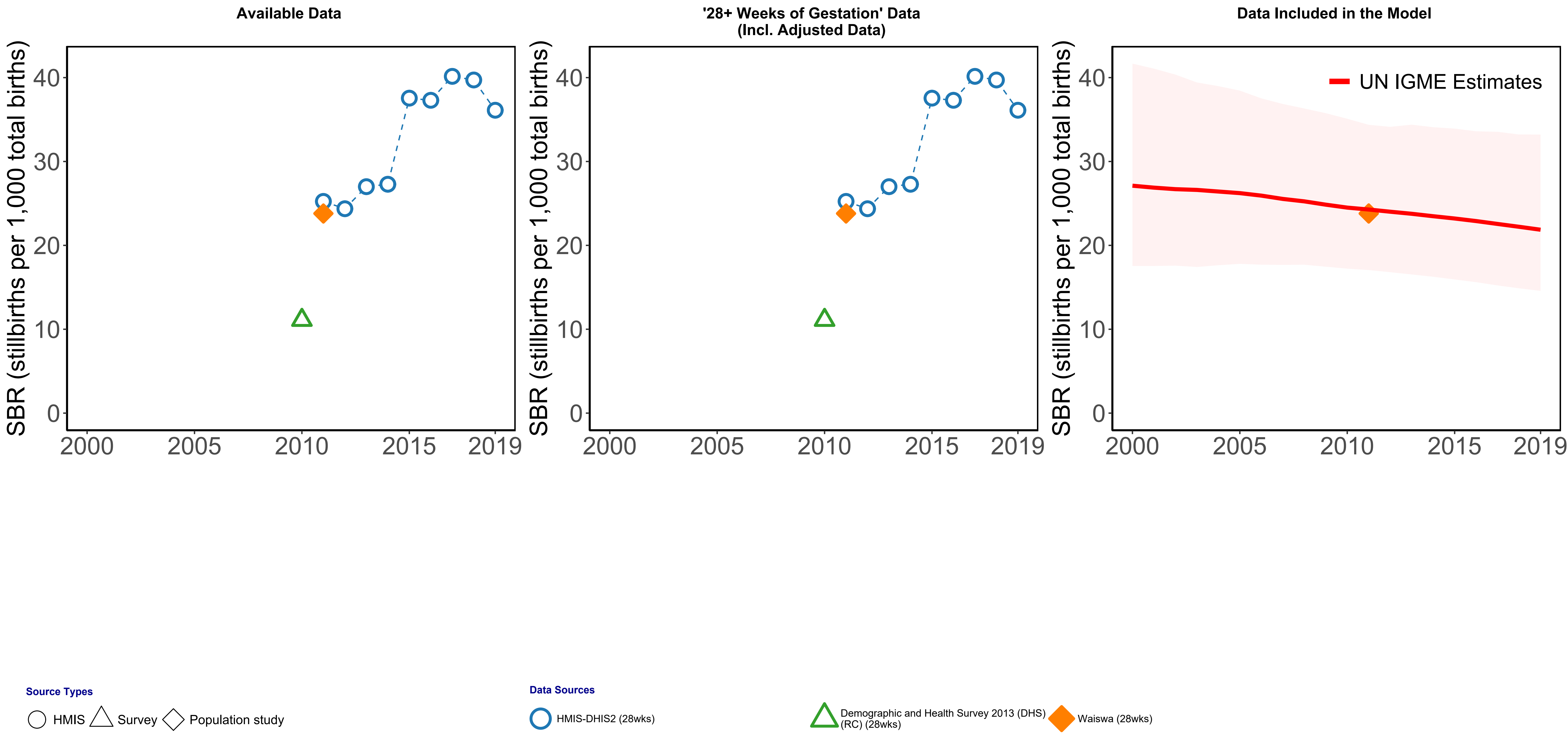

Available Data
'28+ Weeks of Gestation' Data
(Incl. Adjusted Data)
Data Included in the Model
SBR (stillbirths per 1,000 total births)
0
10
20
30
40
2000
2005
2010
2015
2019
UN IGME Estimates
Source Types
HMIS
Survey
Population study
Data Sources
HMIS-DHIS2 (28wks)
Demographic and Health Survey 2013 (DHS) (RC) (28wks)
Waiswa (28wks)

Guinea-Bissau

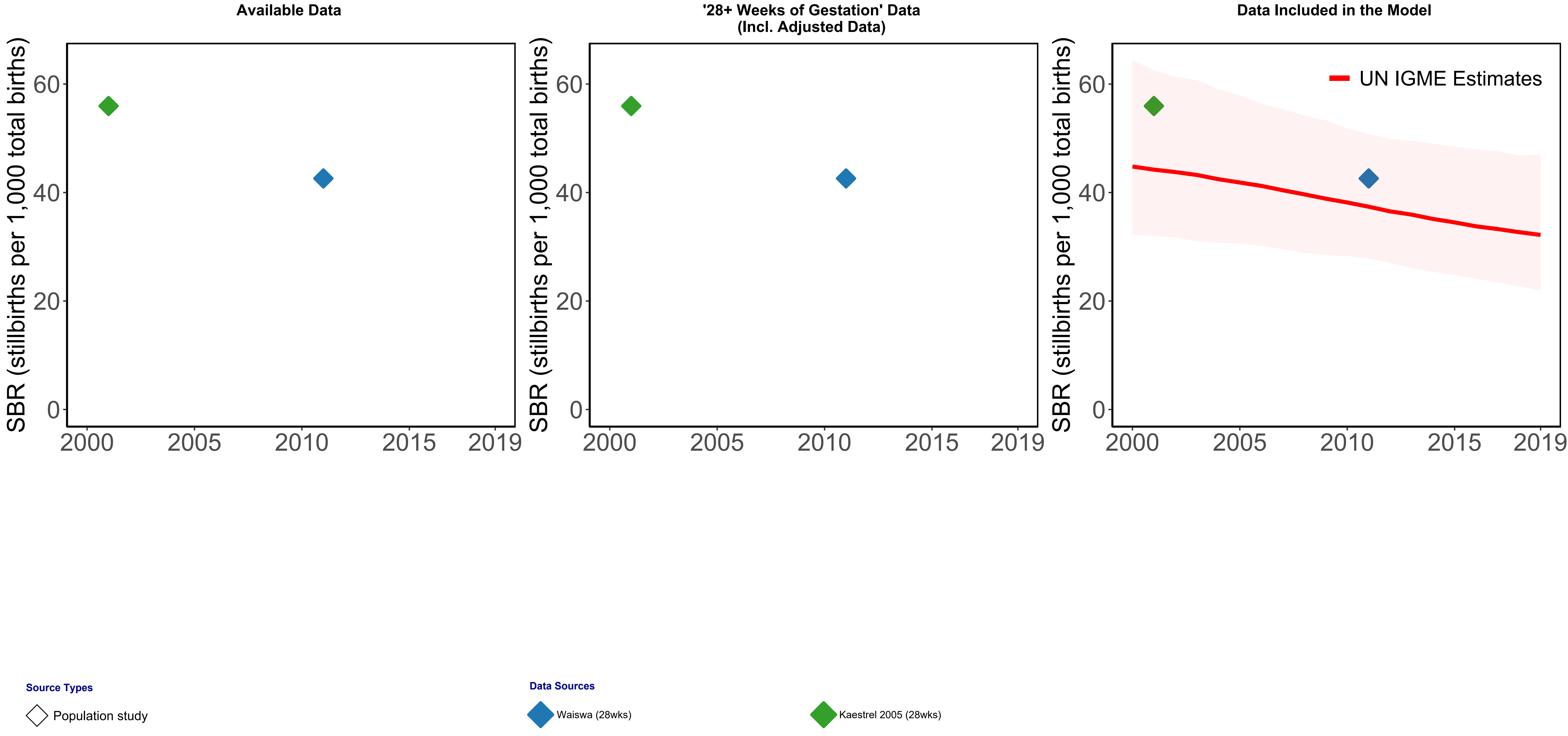

Equatorial Guinea

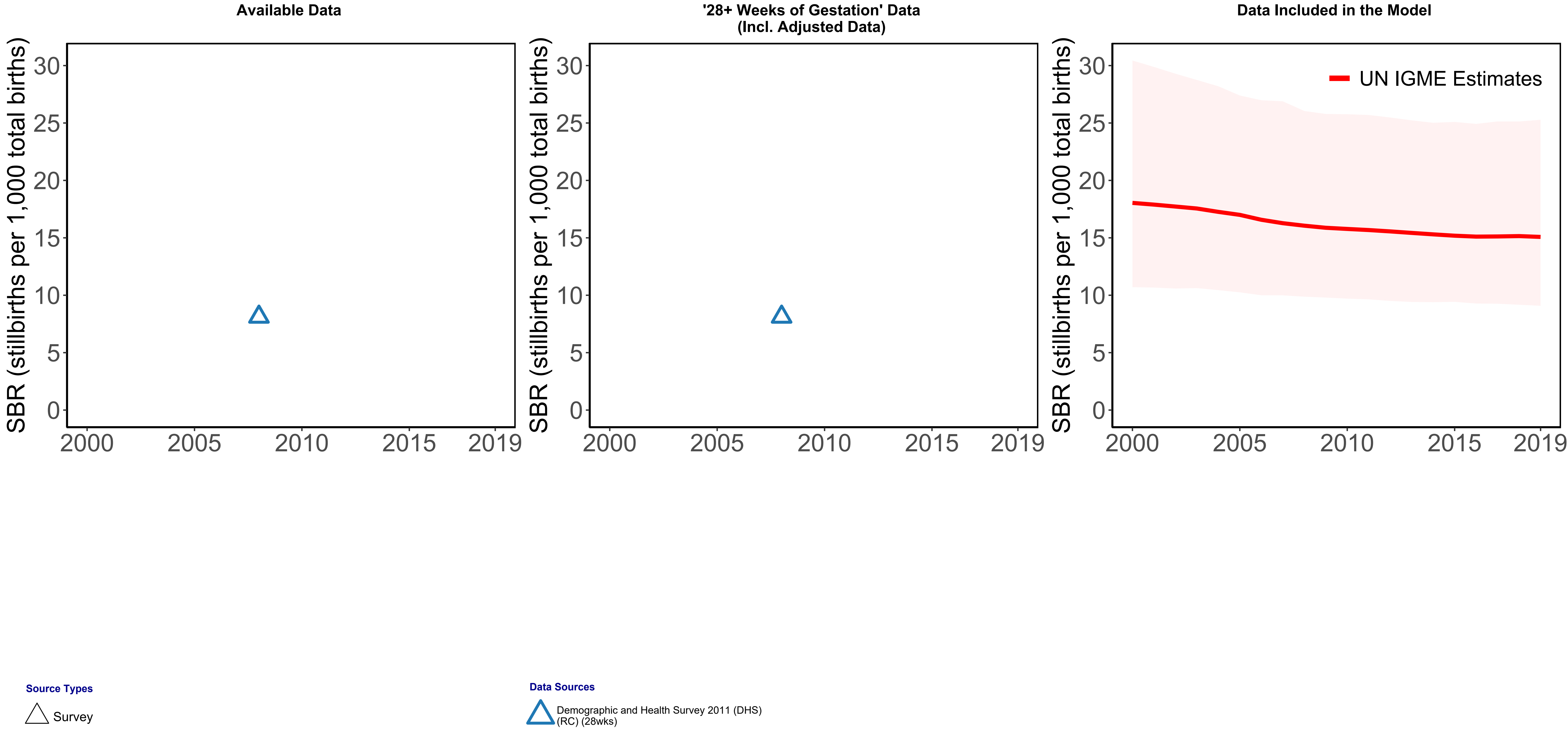

Greece

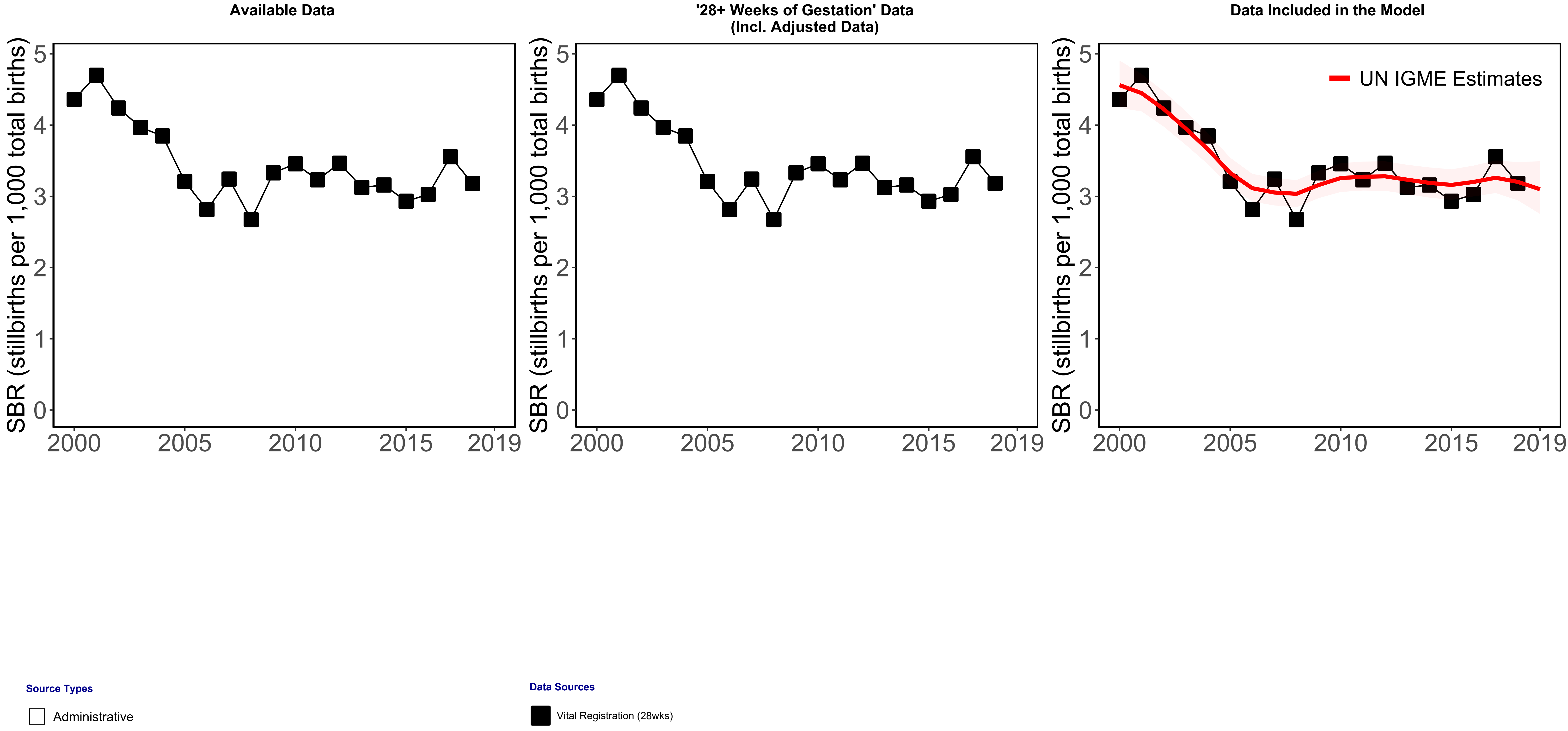

Available Data
'28+ Weeks of Gestation' Data
(Incl. Adjusted Data)
Data Included in the Model
SBR (stillbirths per 1,000 total births)
0
1
2
3
4
5
2000
2005
2010
2015
2019
UN IGME Estimates
Source Types
Administrative
Data Sources
Vital Registration (28wks)

Grenada

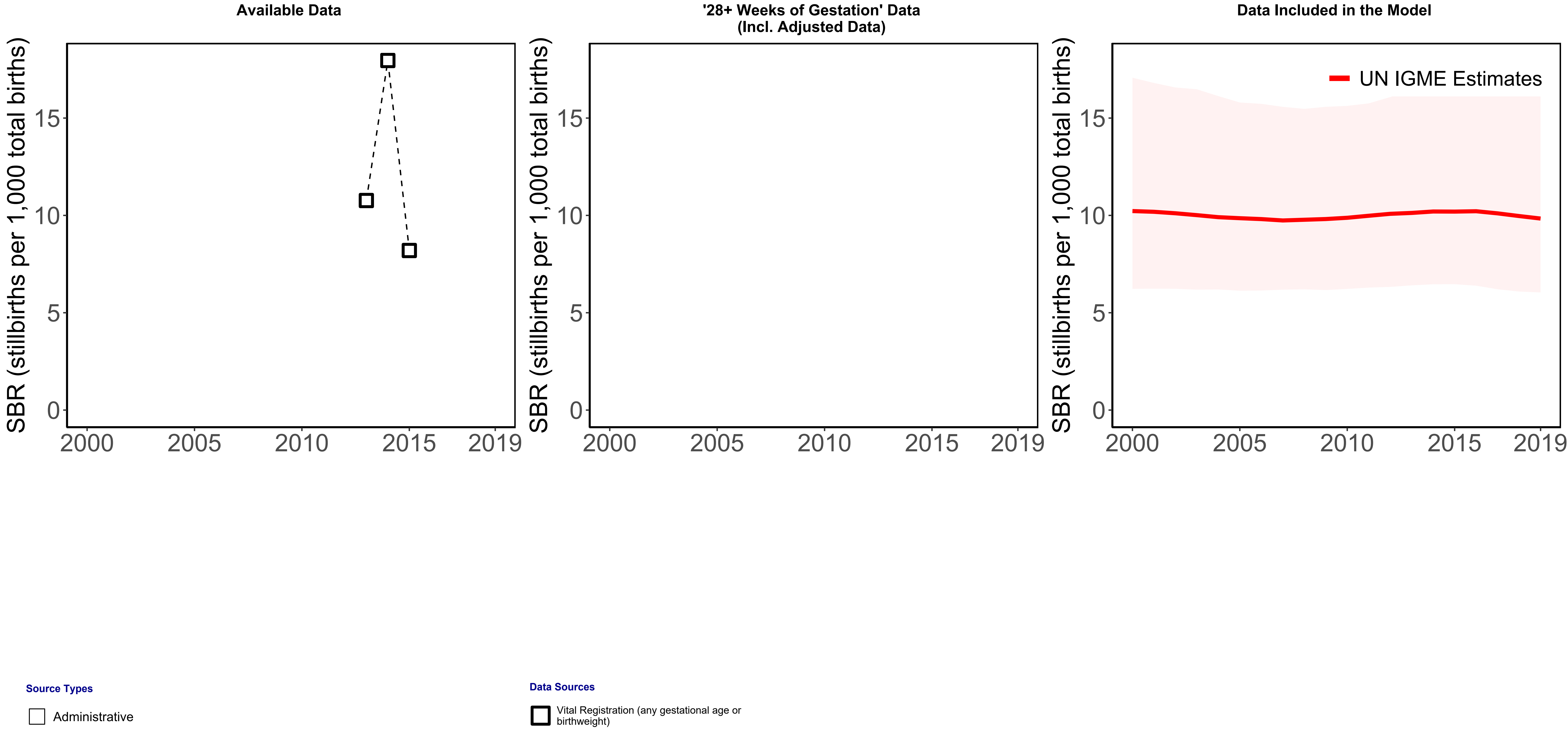

Available Data
'28+ Weeks of Gestation' Data
(Incl. Adjusted Data)
Data Included in the Model
SBR (stillbirths per 1,000 total births)
0
5
10
15
2000
2005
2010
2015
2019
UN IGME Estimates
Source Types
Administrative
Data Sources
Vital Registration (any gestational age or birthweight)

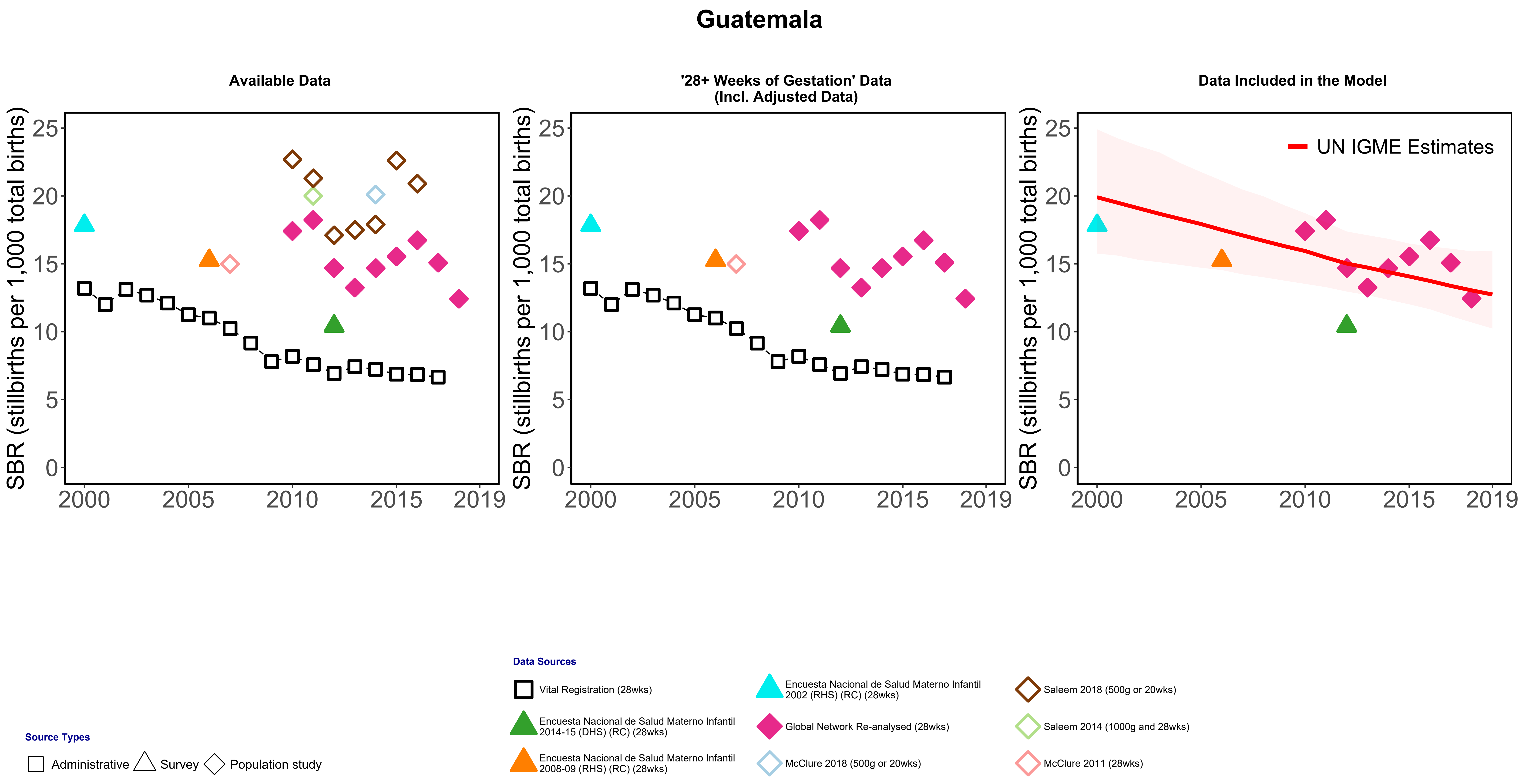
Guatemala
Available Data
'28+ Weeks of Gestation' Data
(Incl. Adjusted Data)
Data Included in the Model
SBR (stillbirths per 1,000 total births)
0
5
10
15
20
25
2000
2005
2010
2015
2019
UN IGME Estimates
Data Sources
Vital Registration (28wks)
Encuesta Nacional de Salud Materno Infantil 2002 (RHS) (RC) (28wks)
Saleem 2018 (500g or 20wks)
Encuesta Nacional de Salud Materno Infantil 2014-15 (DHS) (RC) (28wks)
Global Network Re-analysed (28wks)
Saleem 2014 (1000g and 28wks)
Encuesta Nacional de Salud Materno Infantil 2008-09 (RHS) (RC) (28wks)
McClure 2018 (500g or 20wks)
McClure 2011 (28wks)
Source Types
Administrative
Survey
Population study

Guyana

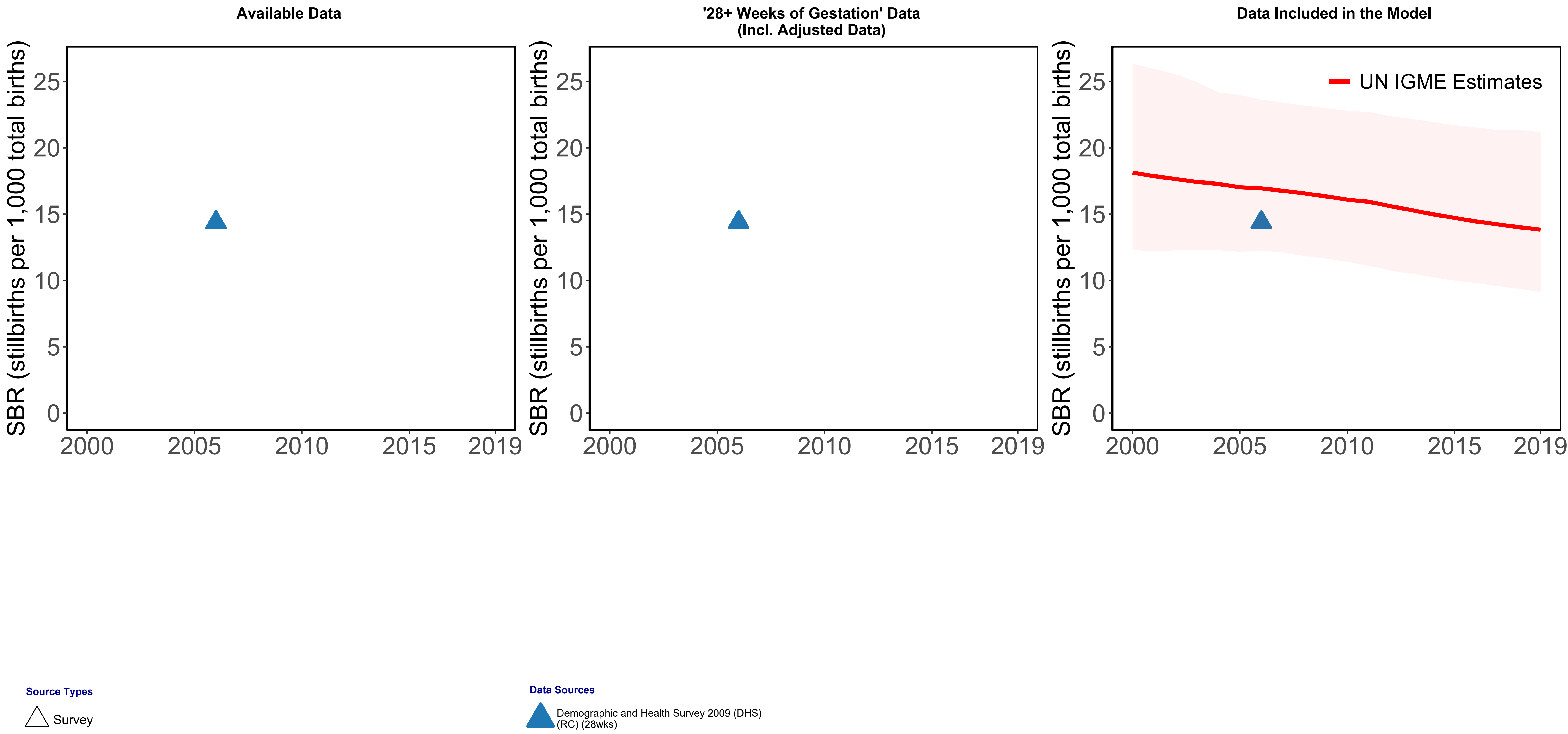

Available Data
'28+ Weeks of Gestation' Data
(Incl. Adjusted Data)
Data Included in the Model
SBR (stillbirths per 1,000 total births)
0
5
10
15
20
25
2000
2005
2010
2015
2019
UN IGME Estimates
Source Types
Survey
Data Sources
Demographic and Health Survey 2009 (DHS) (RC) (28wks)

Honduras

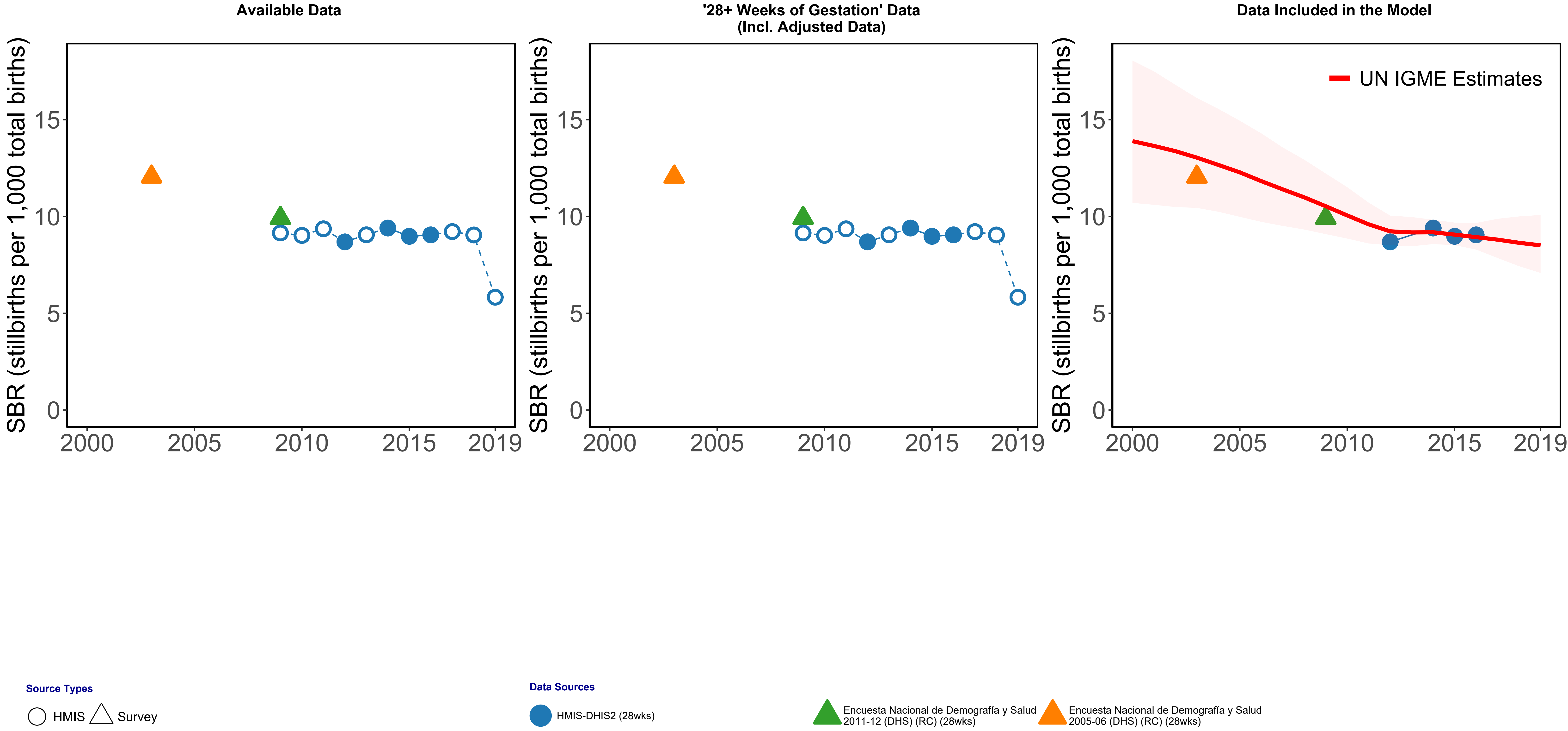
Available Data
'28+ Weeks of Gestation' Data
(Incl. Adjusted Data)
Data Included in the Model
SBR (stillbirths per 1,000 total births)
0
5
10
15
2000
2005
2010
2015
2019
UN IGME Estimates
Source Types
HMIS
Survey
Data Sources
HMIS-DHIS2 (28wks)
Encuesta Nacional de Demografía y Salud 2011-12 (DHS) (RC) (28wks)
Encuesta Nacional de Demografía y Salud 2005-06 (DHS) (RC) (28wks)

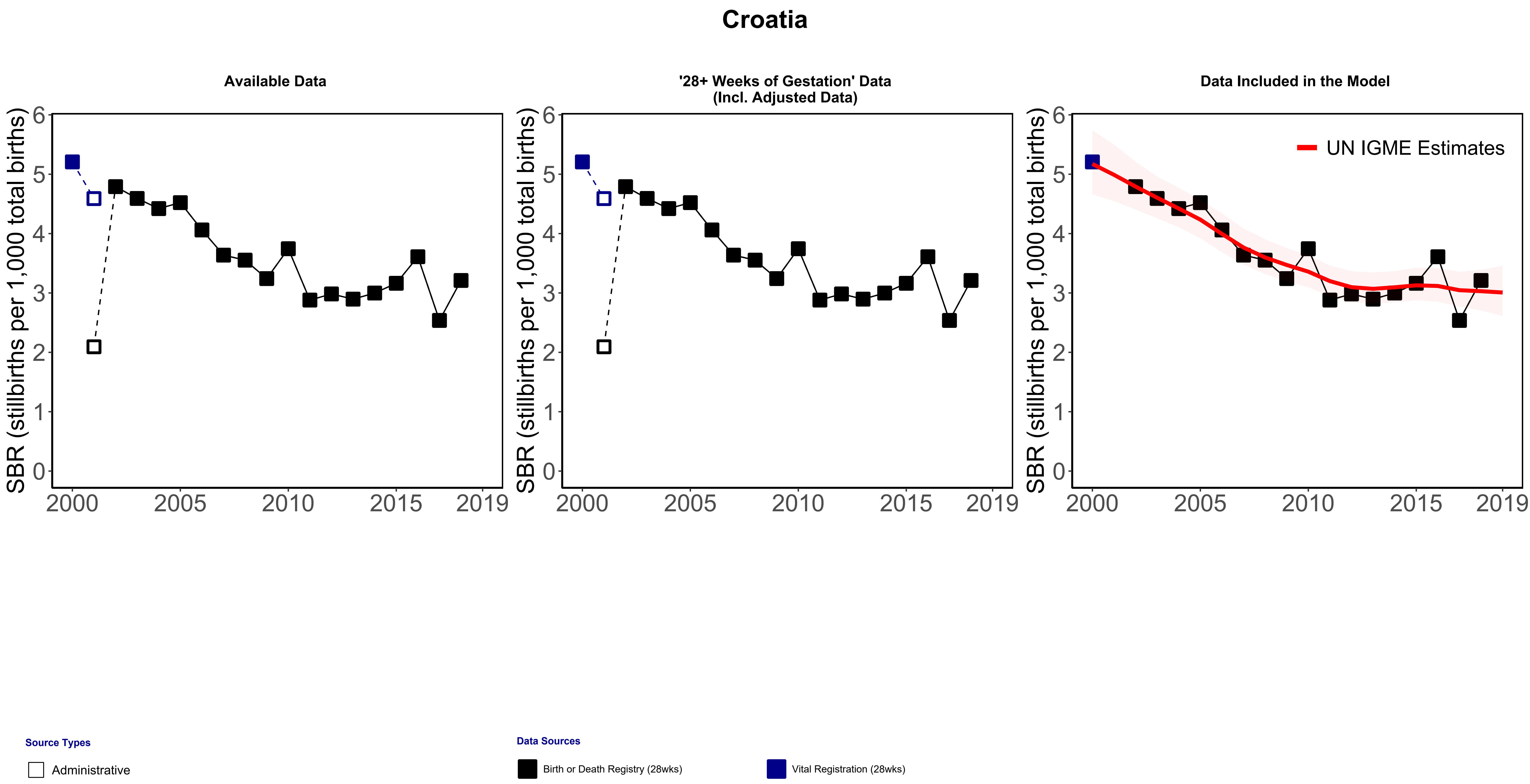

Croatia
Available Data
'28+ Weeks of Gestation' Data
(Incl. Adjusted Data)
Data Included in the Model
SBR (stillbirths per 1,000 total births)
UN IGME Estimates
0
1
2
3
4
5
6
2000
2005
2010
2015
2019
Source Types
Administrative
Data Sources
Birth or Death Registry (28wks)
Vital Registration (28wks)

Haiti

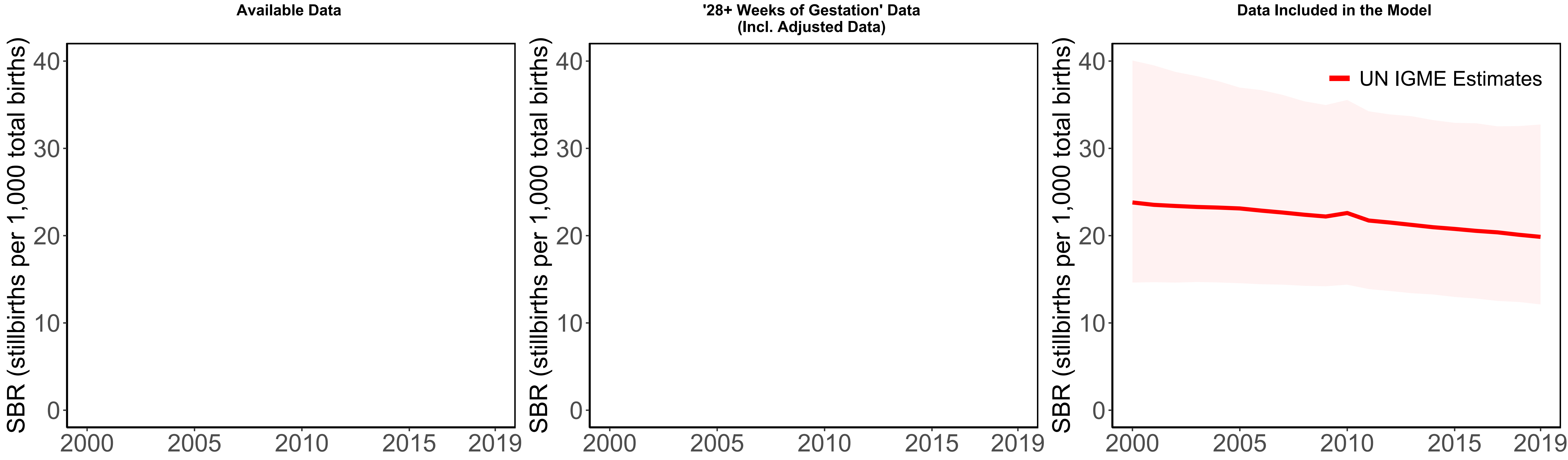
Available Data
'28+ Weeks of Gestation' Data
(Incl. Adjusted Data)
Data Included in the Model
SBR (stillbirths per 1,000 total births)
UN IGME Estimates
0
10
20
30
40
2000
2005
2010
2015
2019

Hungary

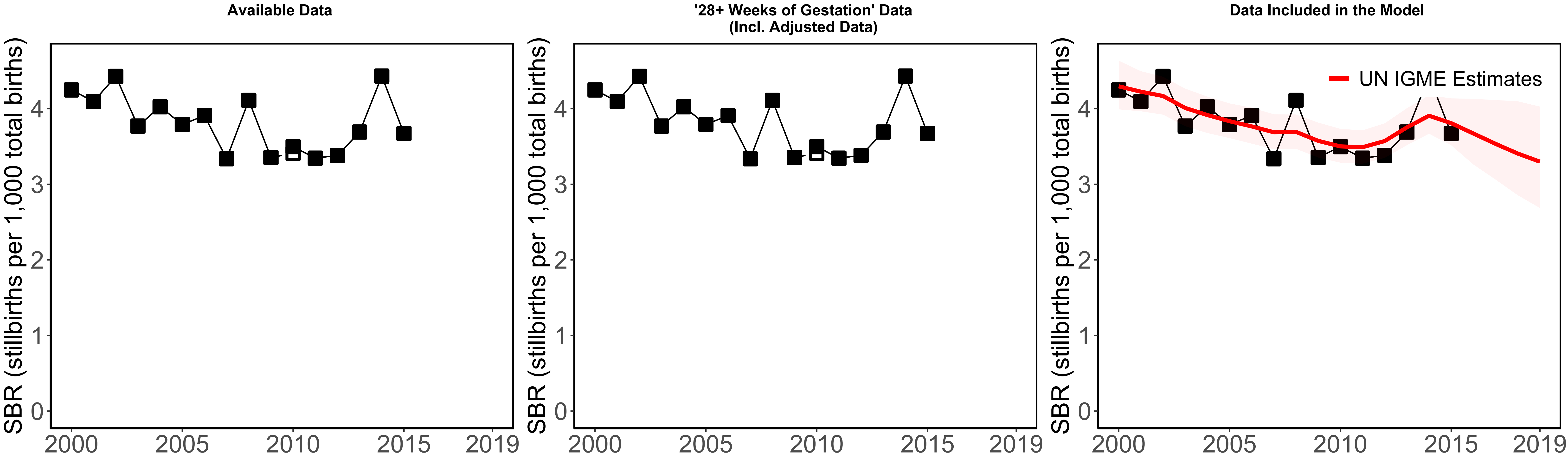

Available Data
'28+ Weeks of Gestation' Data
(Incl. Adjusted Data)
Data Included in the Model
SBR (stillbirths per 1,000 total births)
UN IGME Estimates
0
1
2
3
4
2000
2005
2010
2015
2019

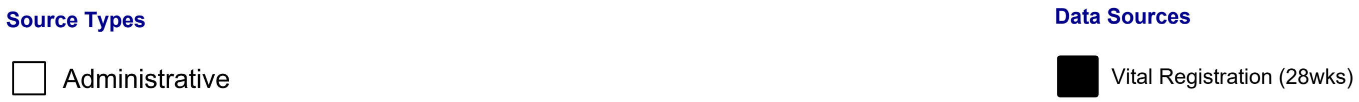

Source Types
Administrative
Data Sources
Vital Registration (28wks)

Indonesia

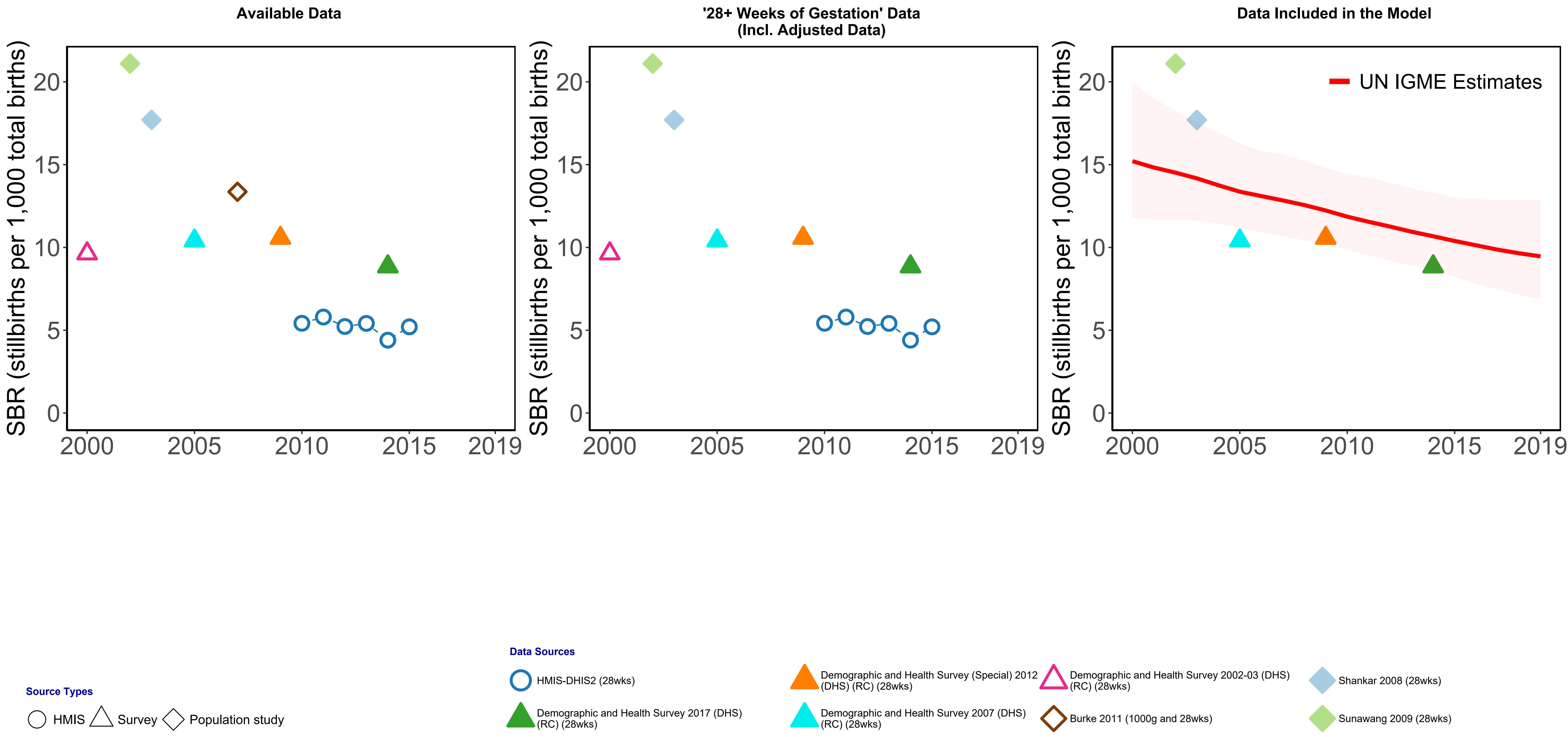
Available Data
'28+ Weeks of Gestation' Data
(Incl. Adjusted Data)
Data Included in the Model
SBR (stillbirths per 1,000 total births)
0
5
10
15
20
2000
2005
2010
2015
2019
UN IGME Estimates
Source Types
HMIS
Survey
Population study
Data Sources
HMIS-DHIS2 (28wks)
Demographic and Health Survey 2017 (DHS) (RC) (28wks)
Demographic and Health Survey (Special) 2012 (DHS) (RC) (28wks)
Demographic and Health Survey 2007 (DHS) (RC) (28wks)
Demographic and Health Survey 2002-03 (DHS) (RC) (28wks)
Burke 2011 (1000g and 28wks)
Shankar 2008 (28wks)
Sunawang 2009 (28wks)

India

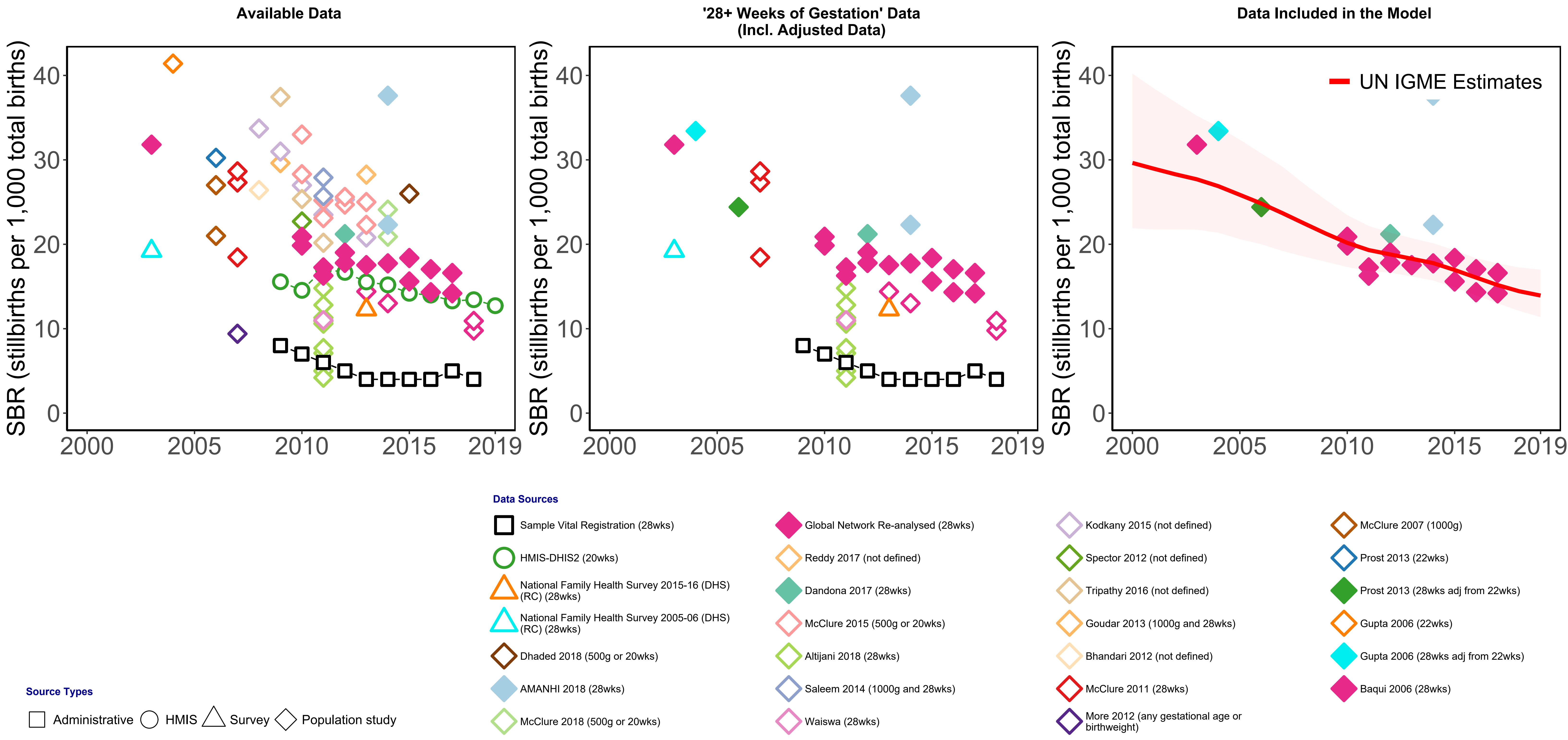

Available Data
'28+ Weeks of Gestation' Data
(Incl. Adjusted Data)
Data Included in the Model
SBR (stillbirths per 1,000 total births)
0
10
20
30
40
2000
2005
2010
2015
2019
UN IGME Estimates
Data Sources
Sample Vital Registration (28wks)
HMIS-DHIS2 (20wks)
National Family Health Survey 2015-16 (DHS) (RC) (28wks)
National Family Health Survey 2005-06 (DHS) (RC) (28wks)
Dhaded 2018 (500g or 20wks)
AMANHI 2018 (28wks)
McClure 2018 (500g or 20wks)
Global Network Re-analysed (28wks)
Reddy 2017 (not defined)
Dandona 2017 (28wks)
McClure 2015 (500g or 20wks)
Altijani 2018 (28wks)
Saleem 2014 (1000g and 28wks)
Waiswa (28wks)
Kodkany 2015 (not defined)
Spector 2012 (not defined)
Tripathy 2016 (not defined)
Goudar 2013 (1000g and 28wks)
Bhandari 2012 (not defined)
McClure 2011 (28wks)
More 2012 (any gestational age or birthweight)
McClure 2007 (1000g)
Prost 2013 (22wks)
Prost 2013 (28wks adj from 22wks)
Gupta 2006 (22wks)
Gupta 2006 (28wks adj from 22wks)
Baqui 2006 (28wks)
Source Types
Administrative
HMIS
Survey
Population study

Ireland

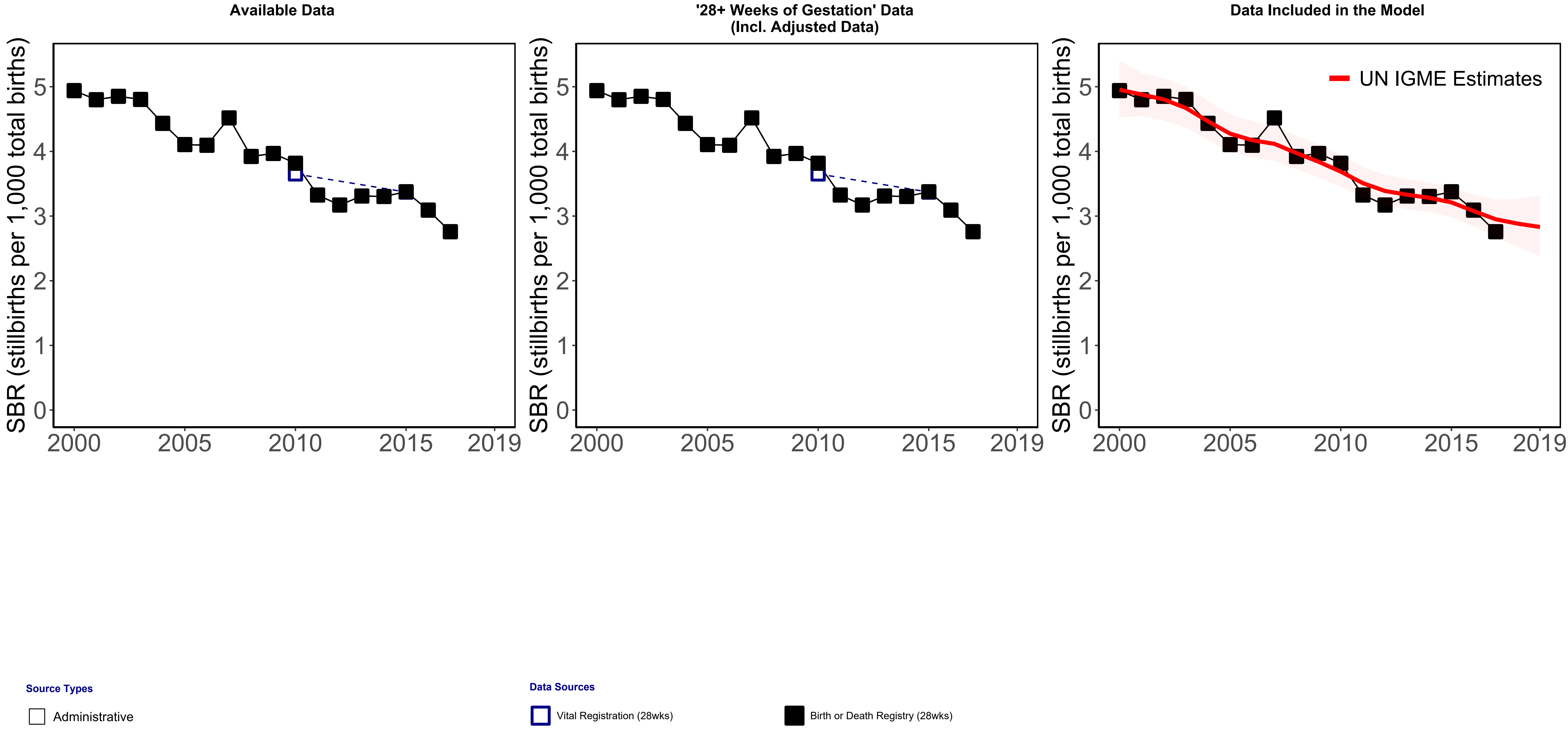

Available Data
'28+ Weeks of Gestation' Data
(Incl. Adjusted Data)
Data Included in the Model
SBR (stillbirths per 1,000 total births)
0
1
2
3
4
5
2000
2005
2010
2015
2019
UN IGME Estimates
Source Types
Administrative
Data Sources
Vital Registration (28wks)
Birth or Death Registry (28wks)

Iran (Islamic Republic of)

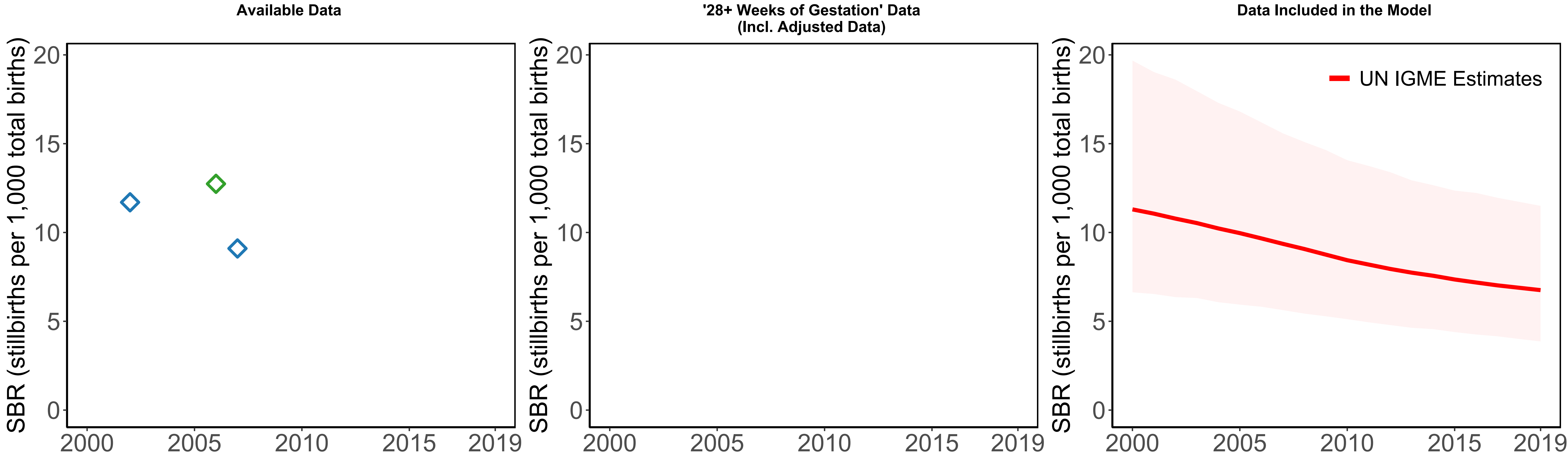

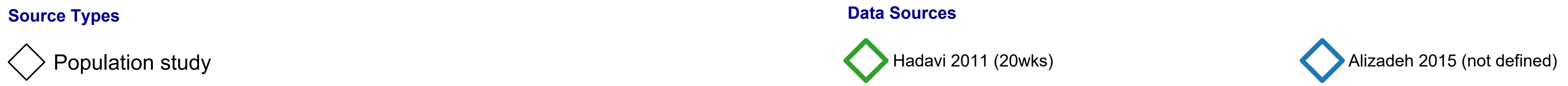

Iraq

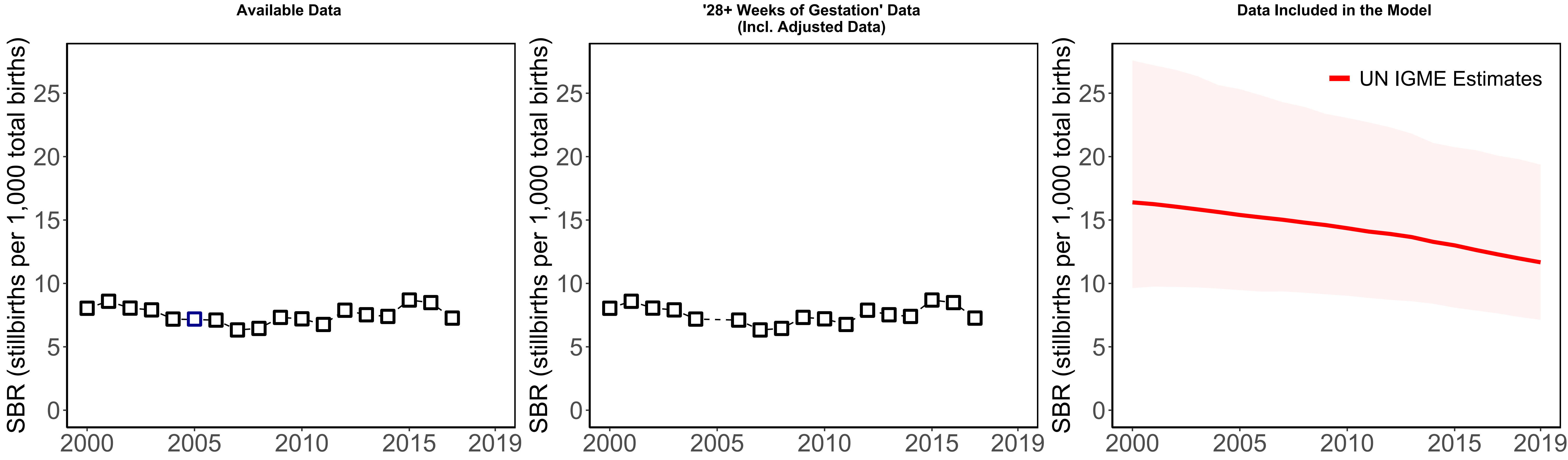

Source Types

Administrative

Data Sources

Vital Registration (28wks)

Vital Registration (not defined)

Iceland

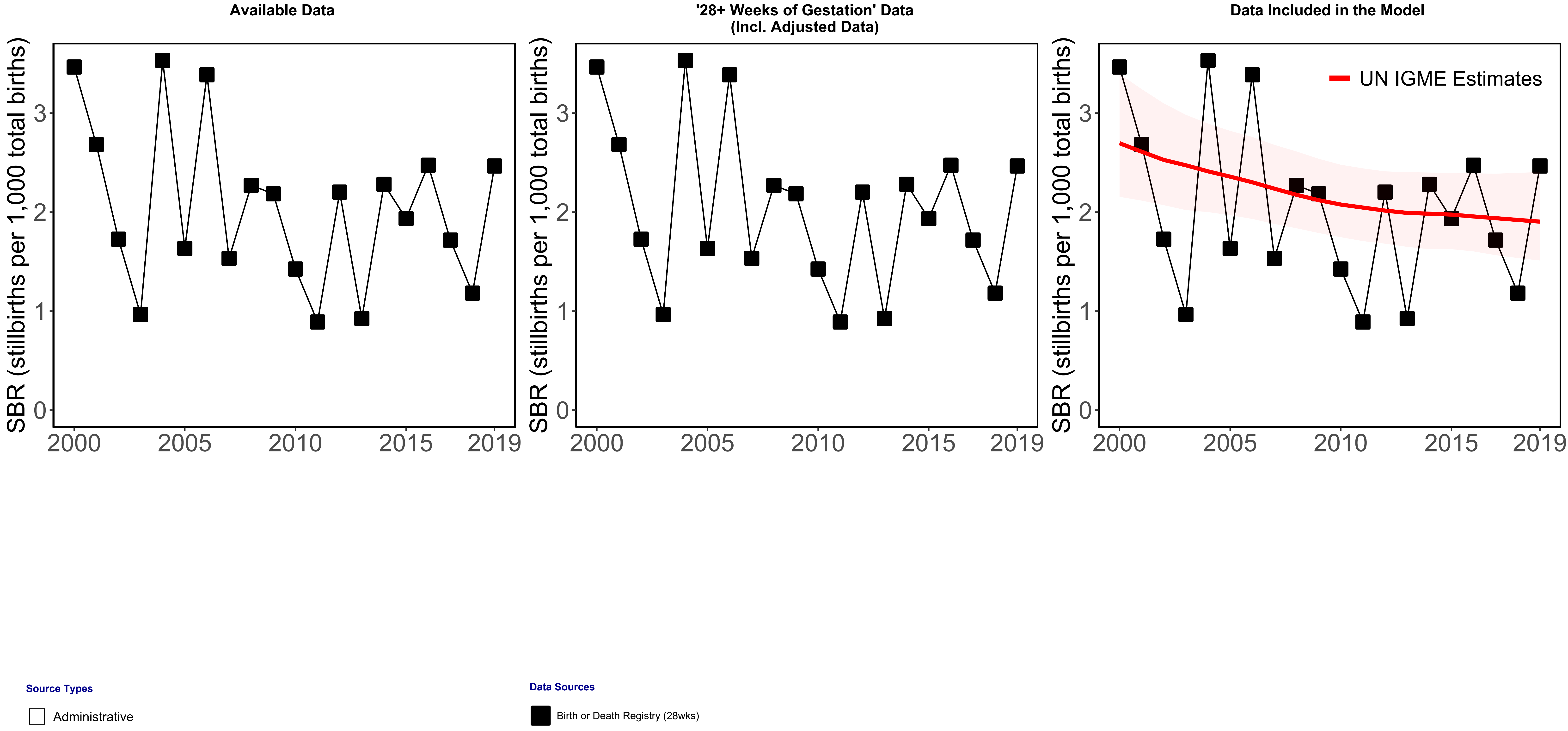

Available Data
'28+ Weeks of Gestation' Data
(Incl. Adjusted Data)
Data Included in the Model
SBR (stillbirths per 1,000 total births)
0
1
2
3
2000
2005
2010
2015
2019
UN IGME Estimates
Source Types
Administrative
Data Sources
Birth or Death Registry (28wks)

Israel

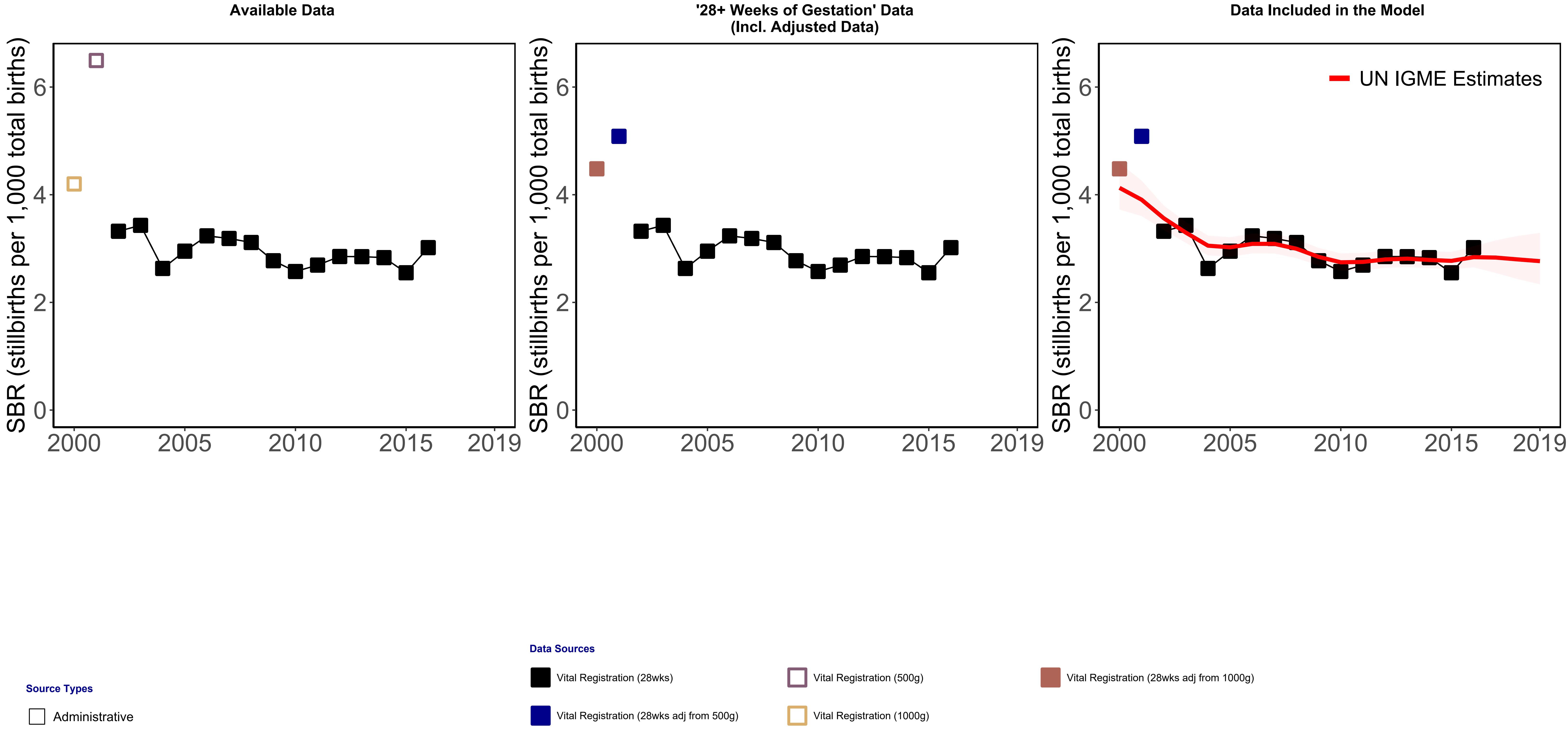

Available Data
'28+ Weeks of Gestation' Data
(Incl. Adjusted Data)
Data Included in the Model
UN IGME Estimates
SBR (stillbirths per 1,000 total births)
0
2
4
6
2000
2005
2010
2015
2019
Data Sources
Vital Registration (28wks)
Vital Registration (500g)
Vital Registration (28wks adj from 1000g)
Vital Registration (28wks adj from 500g)
Vital Registration (1000g)
Source Types
Administrative

Italy

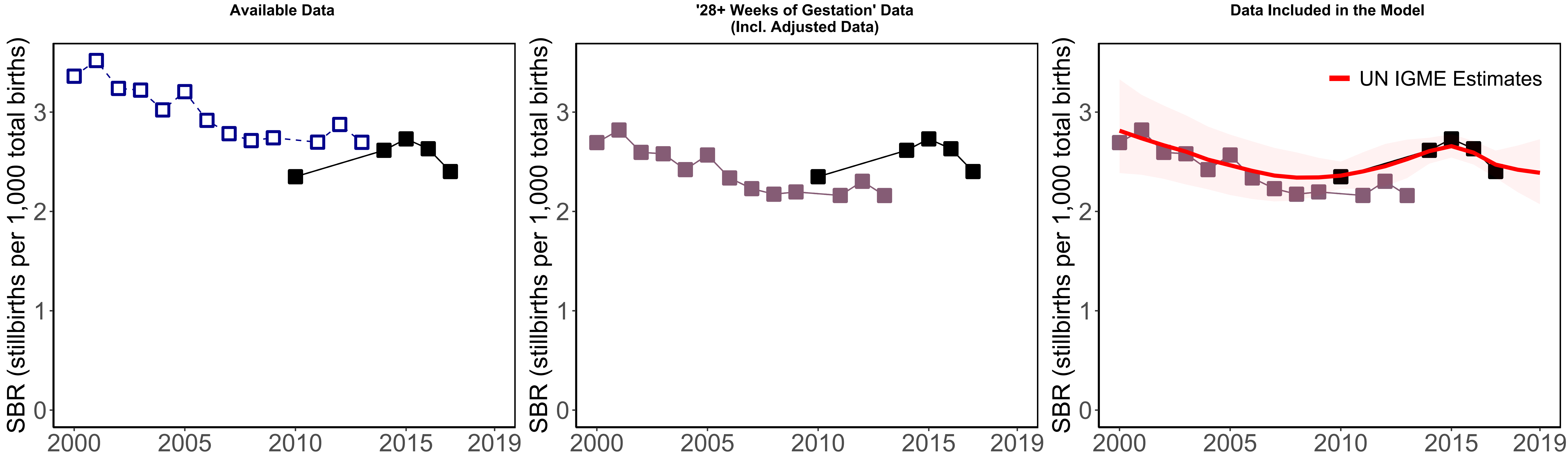

Source Types

Administrative

Data Sources

Vital Registration (28wks)

Vital Registration (24wks)

Vital Registration (28wks adj from 24wks)

Jamaica

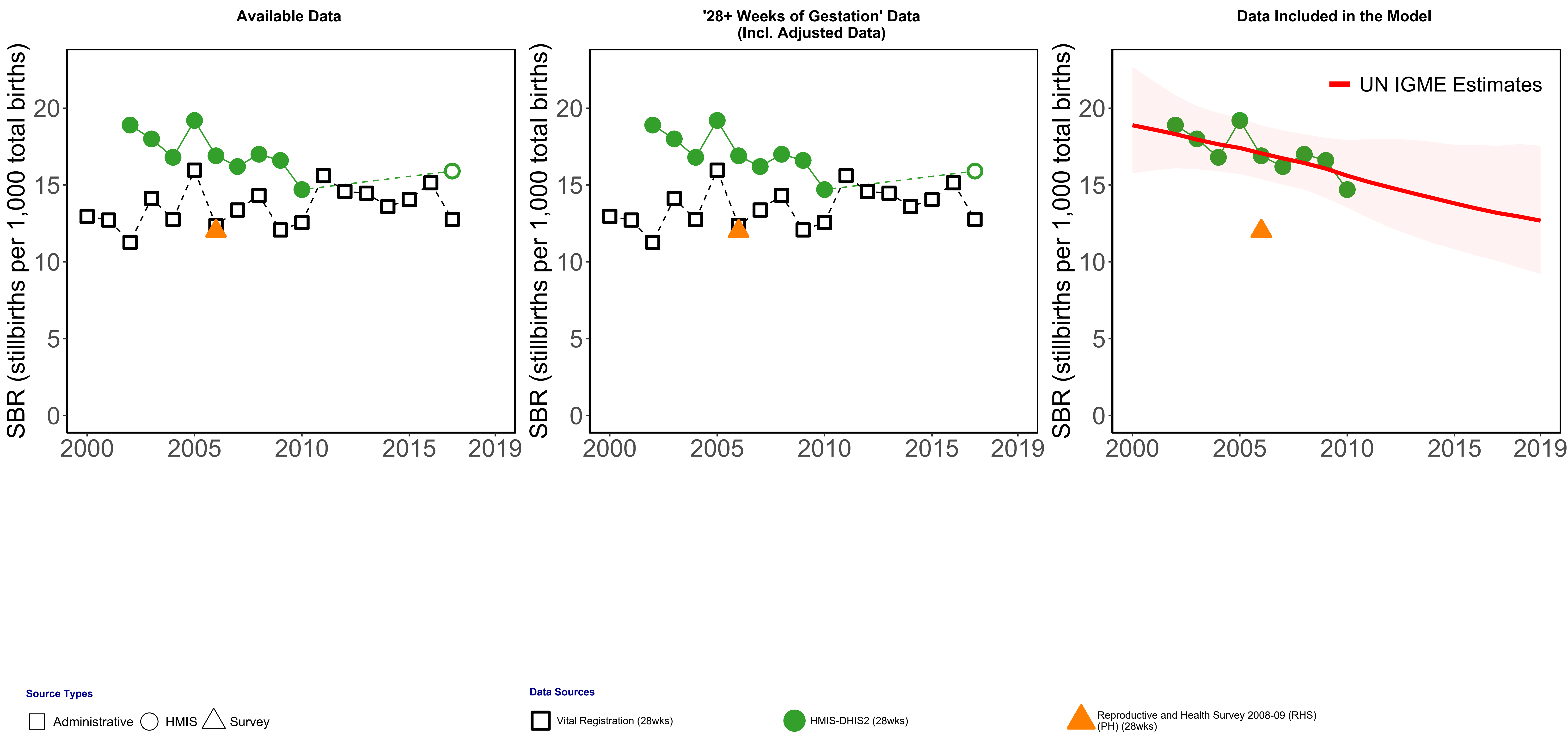
Available Data
'28+ Weeks of Gestation' Data
(Incl. Adjusted Data)
Data Included in the Model
SBR (stillbirths per 1,000 total births)
UN IGME Estimates
0
5
10
15
20
2000
2005
2010
2015
2019
Source Types
Administrative
HMIS
Survey
Data Sources
Vital Registration (28wks)
HMIS-DHIS2 (28wks)
Reproductive and Health Survey 2008-09 (RHS) (PH) (28wks)

Jordan

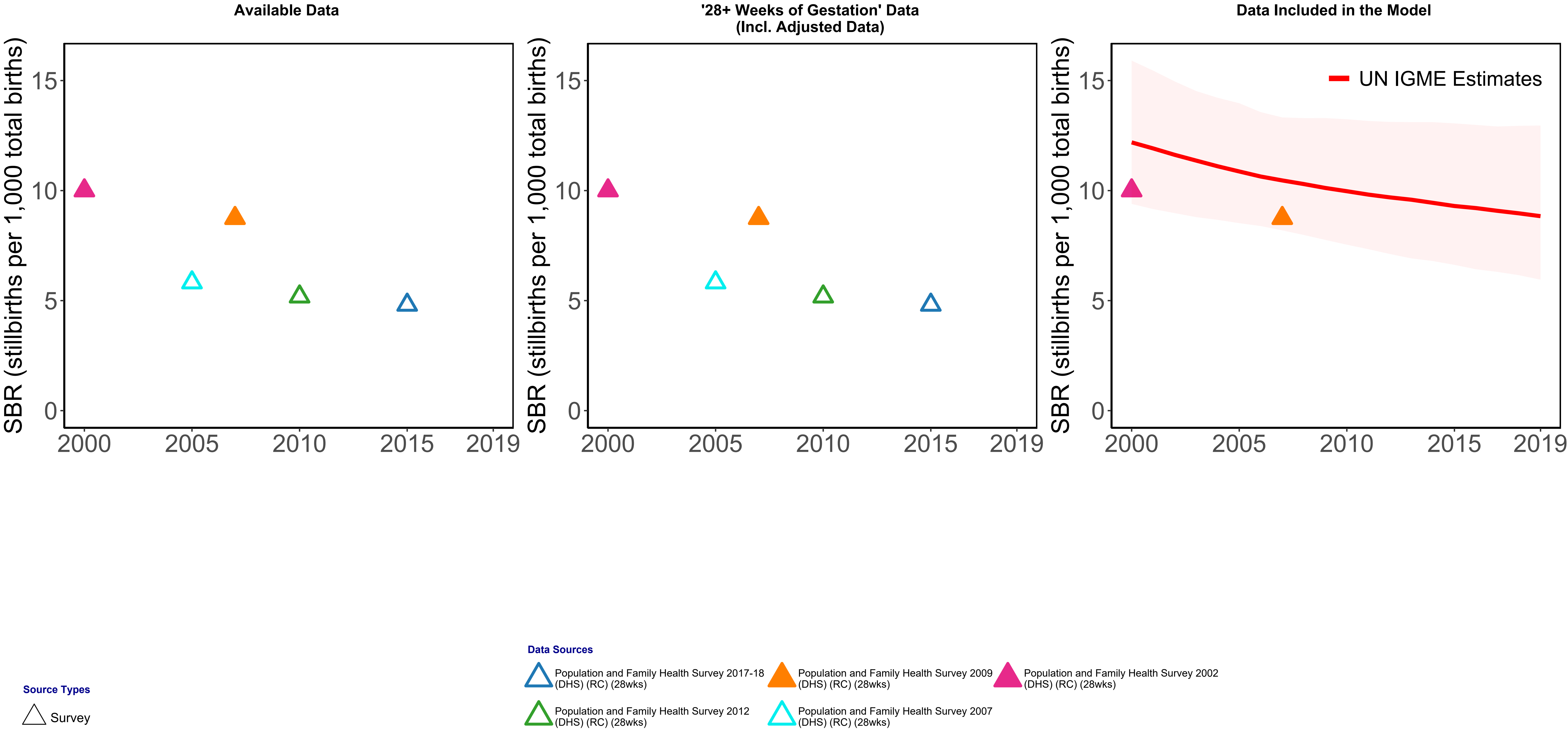
Available Data
'28+ Weeks of Gestation' Data
(Incl. Adjusted Data)
Data Included in the Model
SBR (stillbirths per 1,000 total births)
0
5
10
15
2000
2005
2010
2015
2019
UN IGME Estimates
Data Sources
Population and Family Health Survey 2017-18 (DHS) (RC) (28wks)
Population and Family Health Survey 2009 (DHS) (RC) (28wks)
Population and Family Health Survey 2002 (DHS) (RC) (28wks)
Population and Family Health Survey 2012 (DHS) (RC) (28wks)
Population and Family Health Survey 2007 (DHS) (RC) (28wks)
Source Types
Survey

Japan

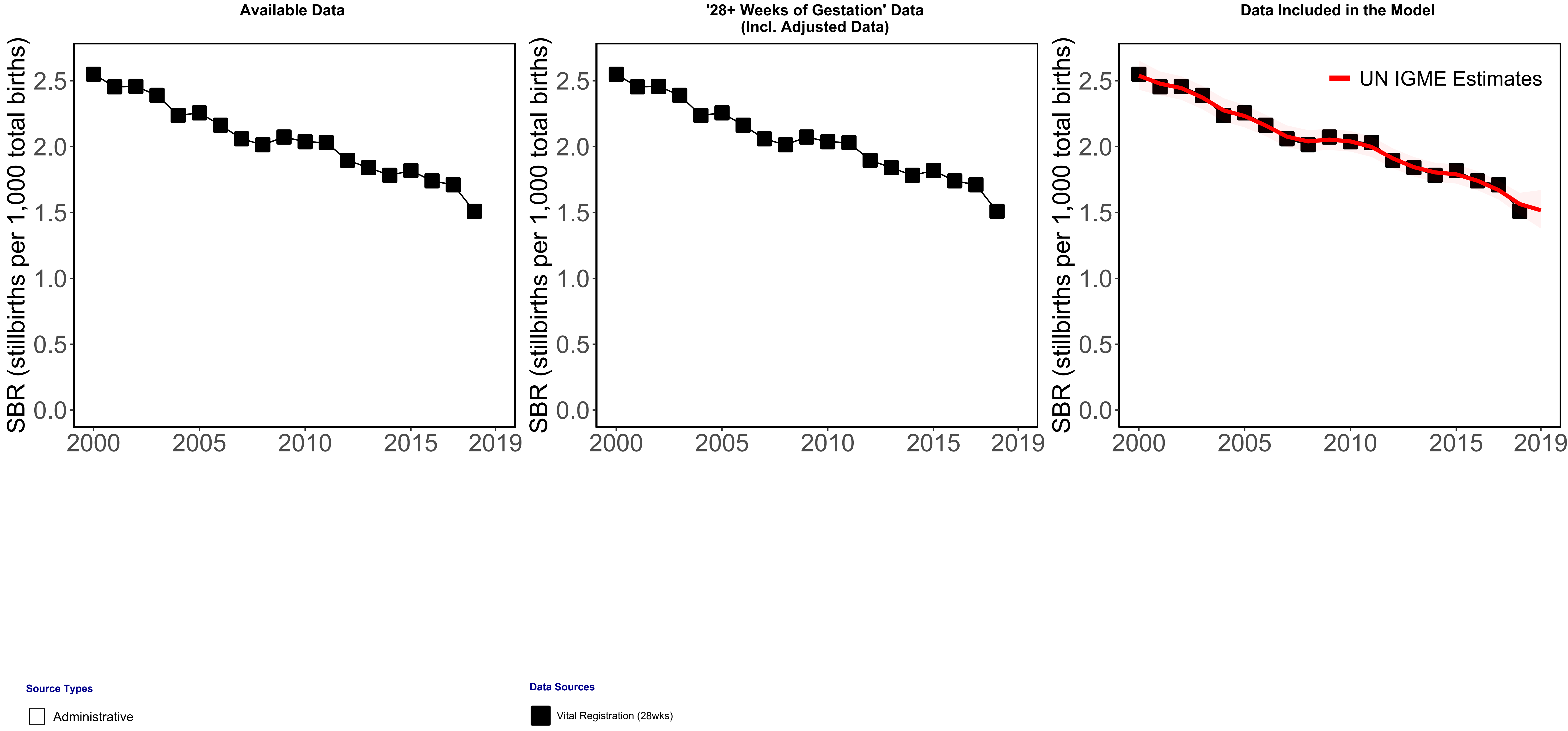

Available Data
'28+ Weeks of Gestation' Data (Incl. Adjusted Data)
Data Included in the Model
SBR (stillbirths per 1,000 total births)
0.0
0.5
1.0
1.5
2.0
2.5
2000
2005
2010
2015
2019
UN IGME Estimates
Source Types
Administrative
Data Sources
Vital Registration (28wks)

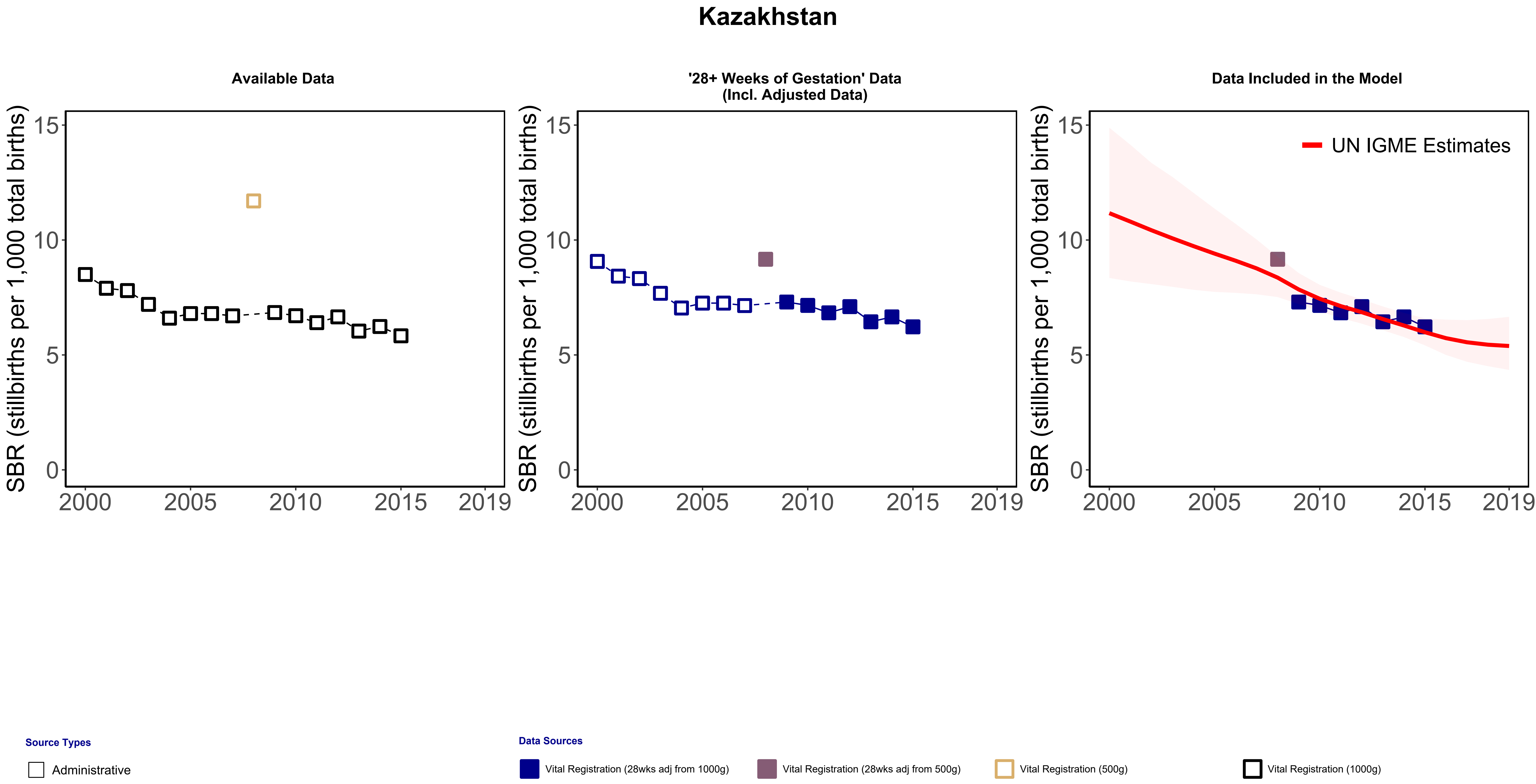

Kazakhstan
Available Data
'28+ Weeks of Gestation' Data
(Incl. Adjusted Data)
Data Included in the Model
SBR (stillbirths per 1,000 total births)
0
5
10
15
2000
2005
2010
2015
2019
UN IGME Estimates
Source Types
Administrative
Data Sources
Vital Registration (28wks adj from 1000g)
Vital Registration (28wks adj from 500g)
Vital Registration (500g)
Vital Registration (1000g)

Kenya

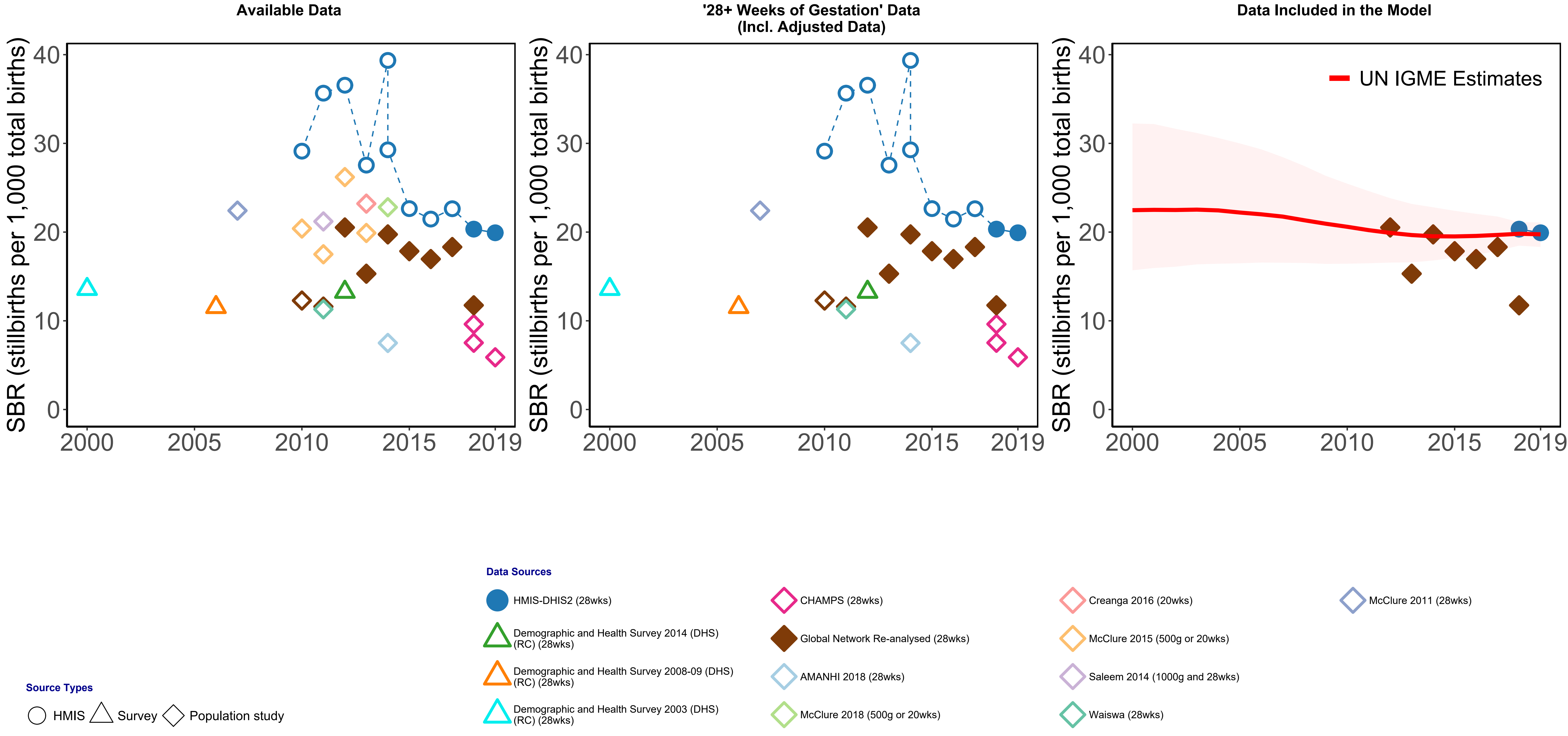
Available Data
'28+ Weeks of Gestation' Data
(Incl. Adjusted Data)
Data Included in the Model
SBR (stillbirths per 1,000 total births)
0
10
20
30
40
2000
2005
2010
2015
2019
UN IGME Estimates
Data Sources
HMIS-DHIS2 (28wks)
Demographic and Health Survey 2014 (DHS) (RC) (28wks)
Demographic and Health Survey 2008-09 (DHS) (RC) (28wks)
Demographic and Health Survey 2003 (DHS) (RC) (28wks)
CHAMPS (28wks)
Global Network Re-analysed (28wks)
AMANHI 2018 (28wks)
McClure 2018 (500g or 20wks)
Creanga 2016 (20wks)
McClure 2015 (500g or 20wks)
Saleem 2014 (1000g and 28wks)
Waiswa (28wks)
McClure 2011 (28wks)
Source Types
HMIS
Survey
Population study

Kyrgyzstan

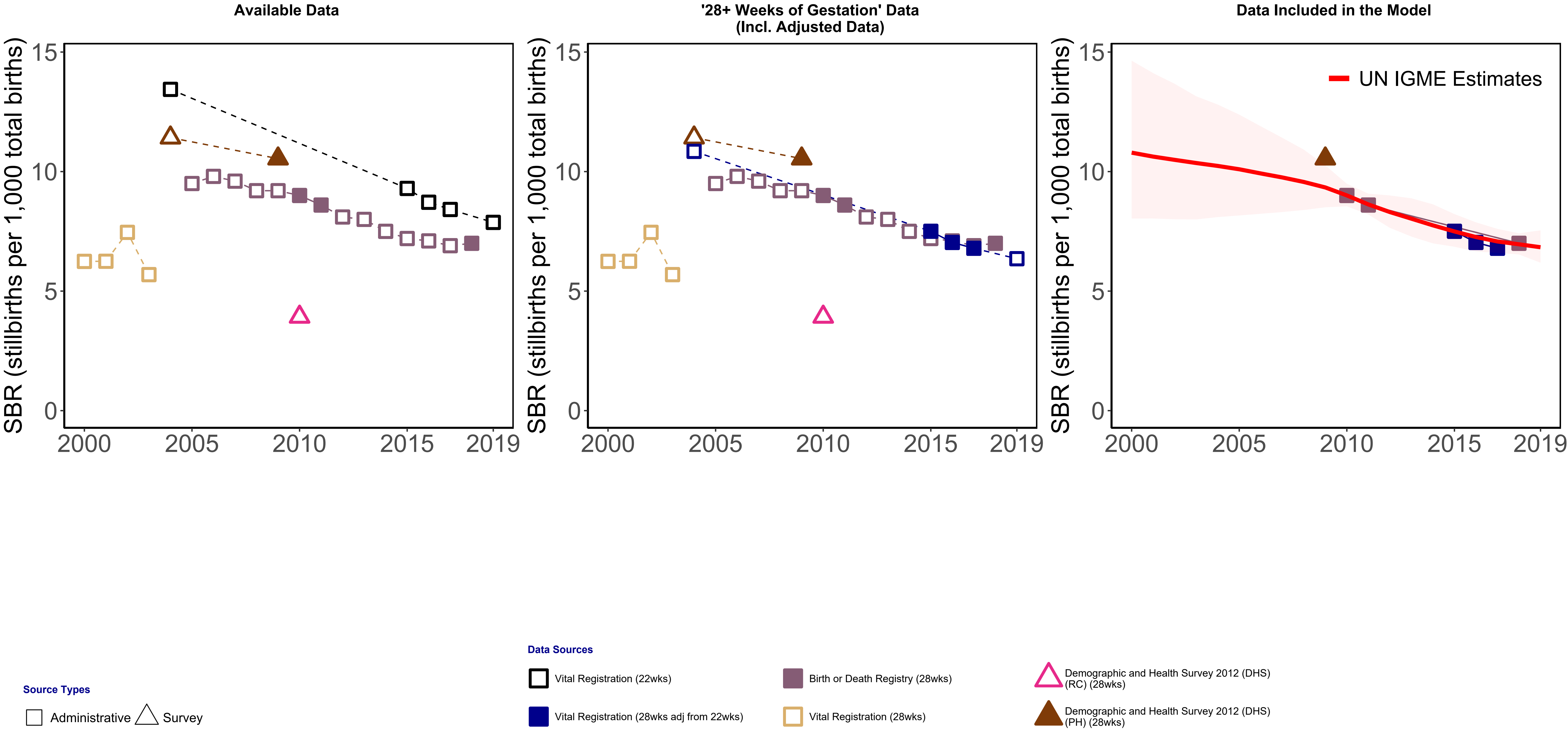
Available Data
'28+ Weeks of Gestation' Data
(Incl. Adjusted Data)
Data Included in the Model
SBR (stillbirths per 1,000 total births)
0
5
10
15
2000
2005
2010
2015
2019
UN IGME Estimates
Source Types
Administrative
Survey
Data Sources
Vital Registration (22wks)
Vital Registration (28wks adj from 22wks)
Birth or Death Registry (28wks)
Vital Registration (28wks)
Demographic and Health Survey 2012 (DHS) (RC) (28wks)
Demographic and Health Survey 2012 (DHS) (PH) (28wks)

Cambodia

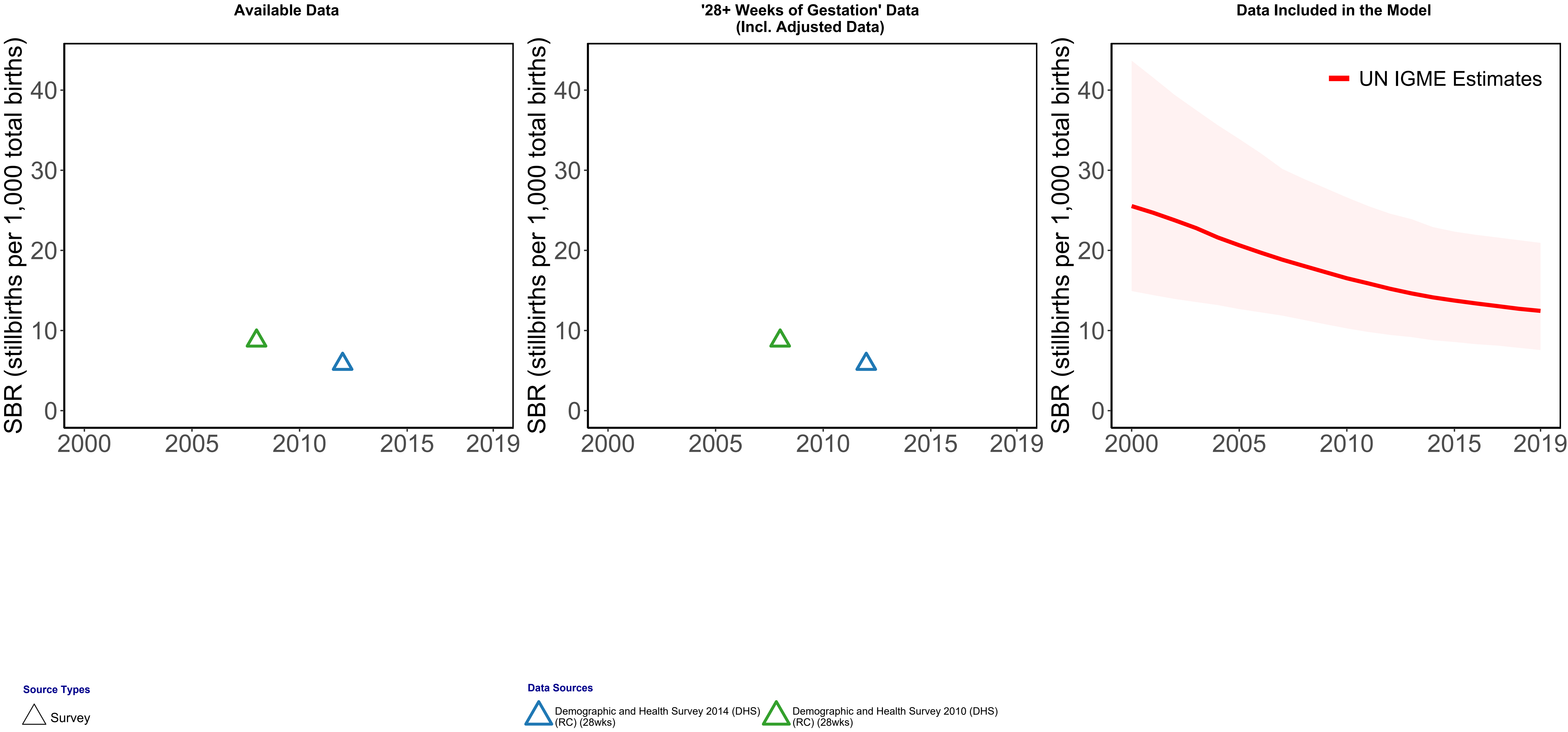
Available Data
'28+ Weeks of Gestation' Data
(Incl. Adjusted Data)
Data Included in the Model
SBR (stillbirths per 1,000 total births)
0
10
20
30
40
2000
2005
2010
2015
2019
UN IGME Estimates
Source Types
Survey
Data Sources
Demographic and Health Survey 2014 (DHS) (RC) (28wks)
Demographic and Health Survey 2010 (DHS) (RC) (28wks)

Kiribati

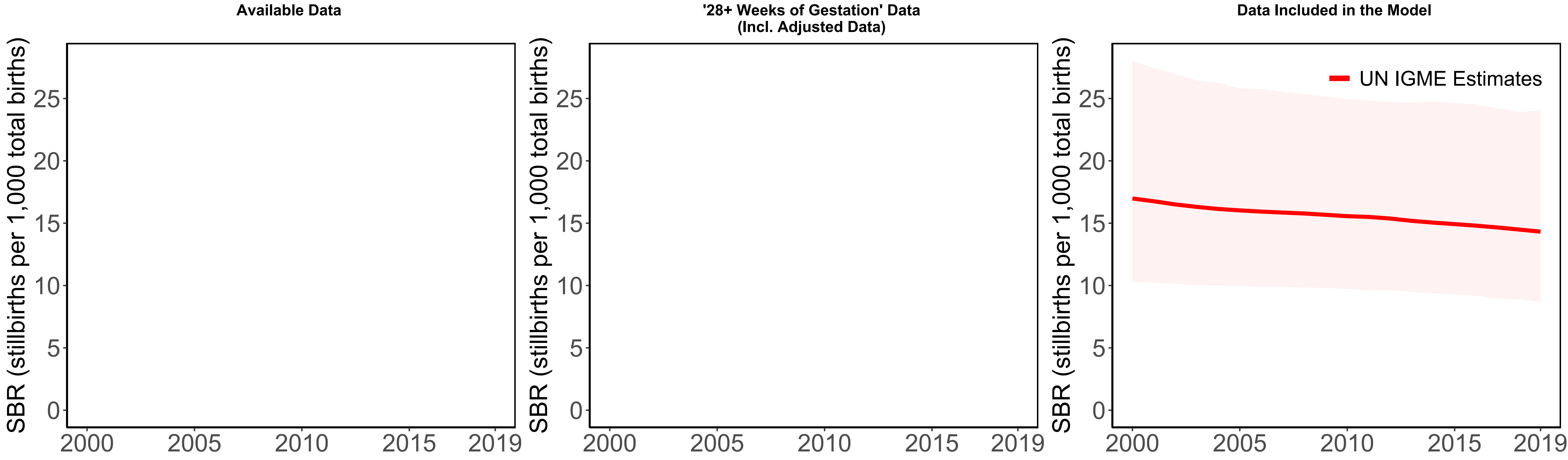

Available Data
'28+ Weeks of Gestation' Data (Incl. Adjusted Data)
Data Included in the Model
UN IGME Estimates
SBR (stillbirths per 1,000 total births)
0
5
10
15
20
25
2000
2005
2010
2015
2019

Saint Kitts and Nevis

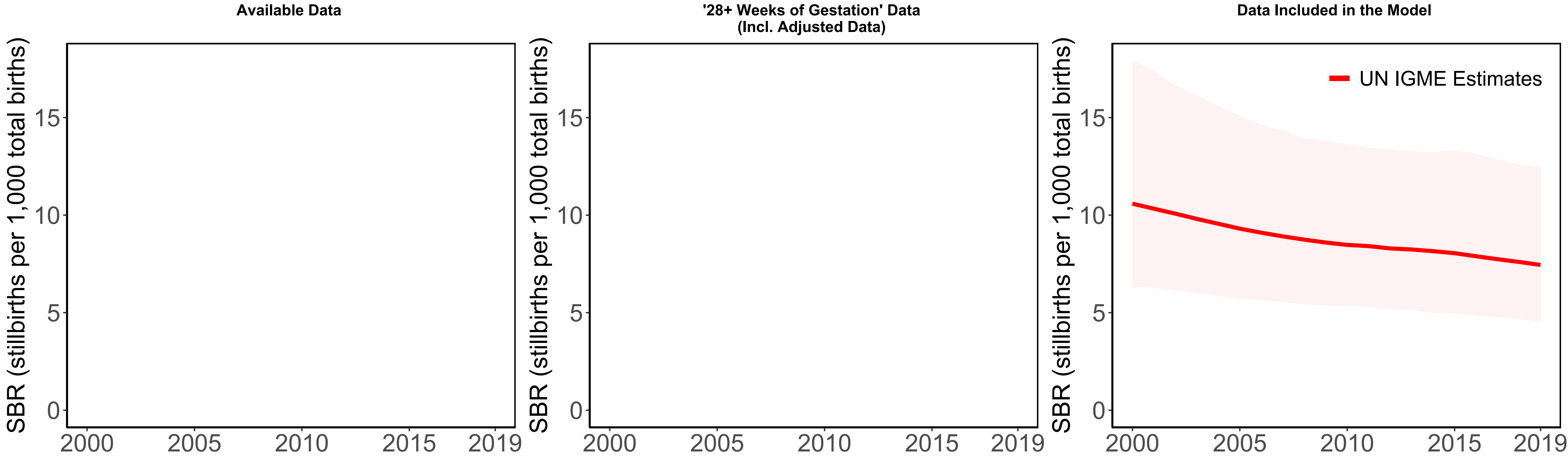

Republic of Korea

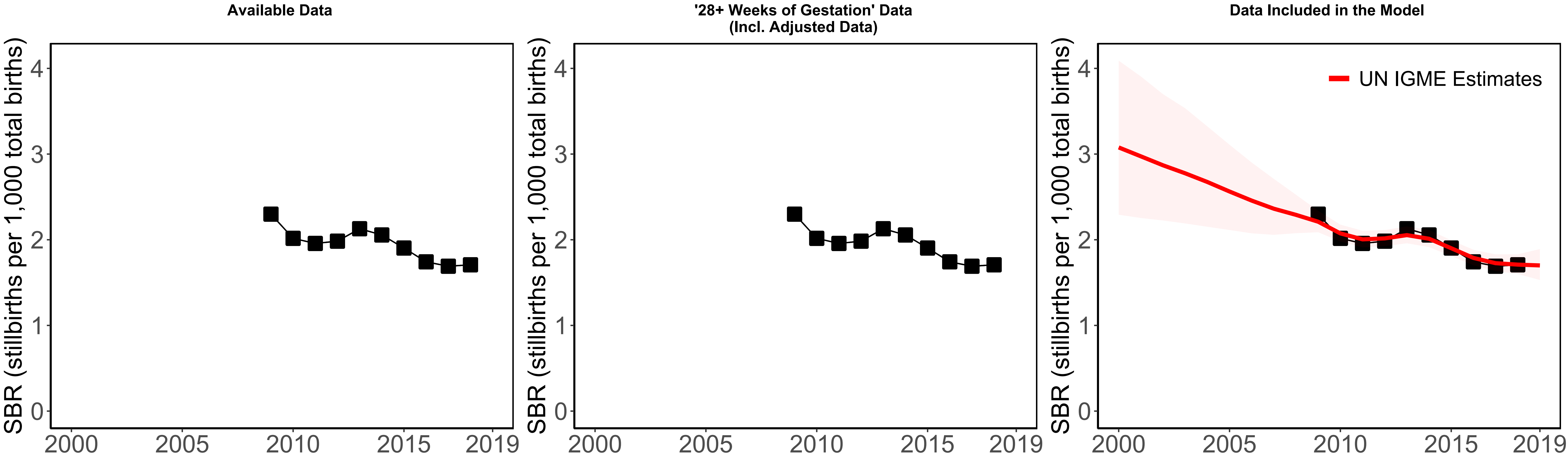

Source Types

Administrative

Data Sources

Vital Registration (28wks)

Kuwait

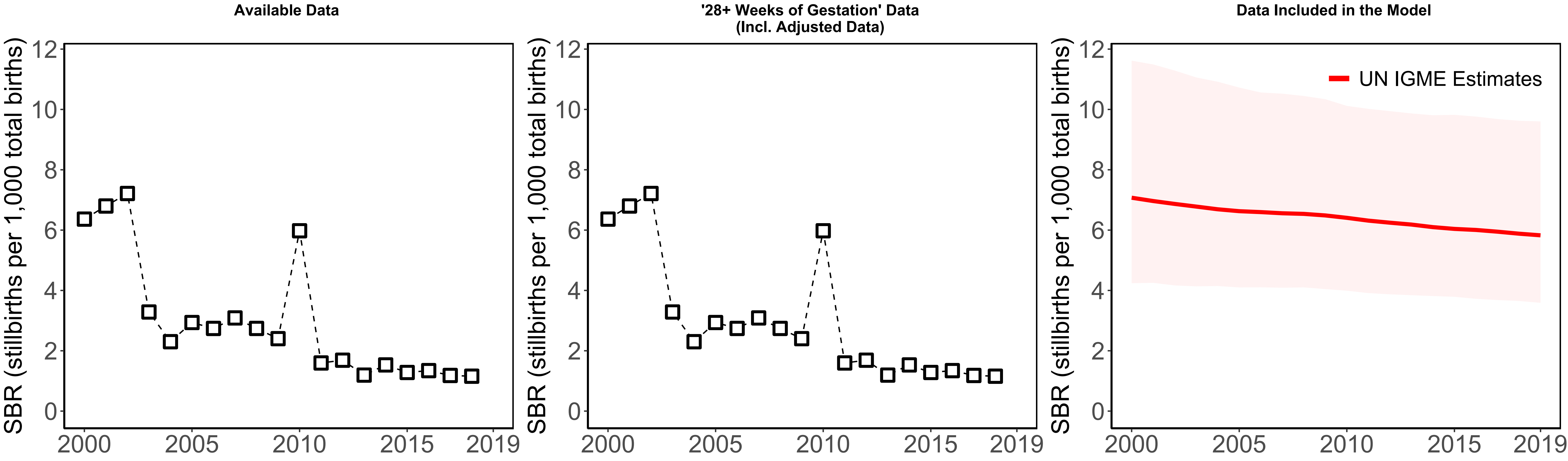

Source Types

Administrative

Data Sources

Vital Registration (28wks)

Lao People's Democratic Republic

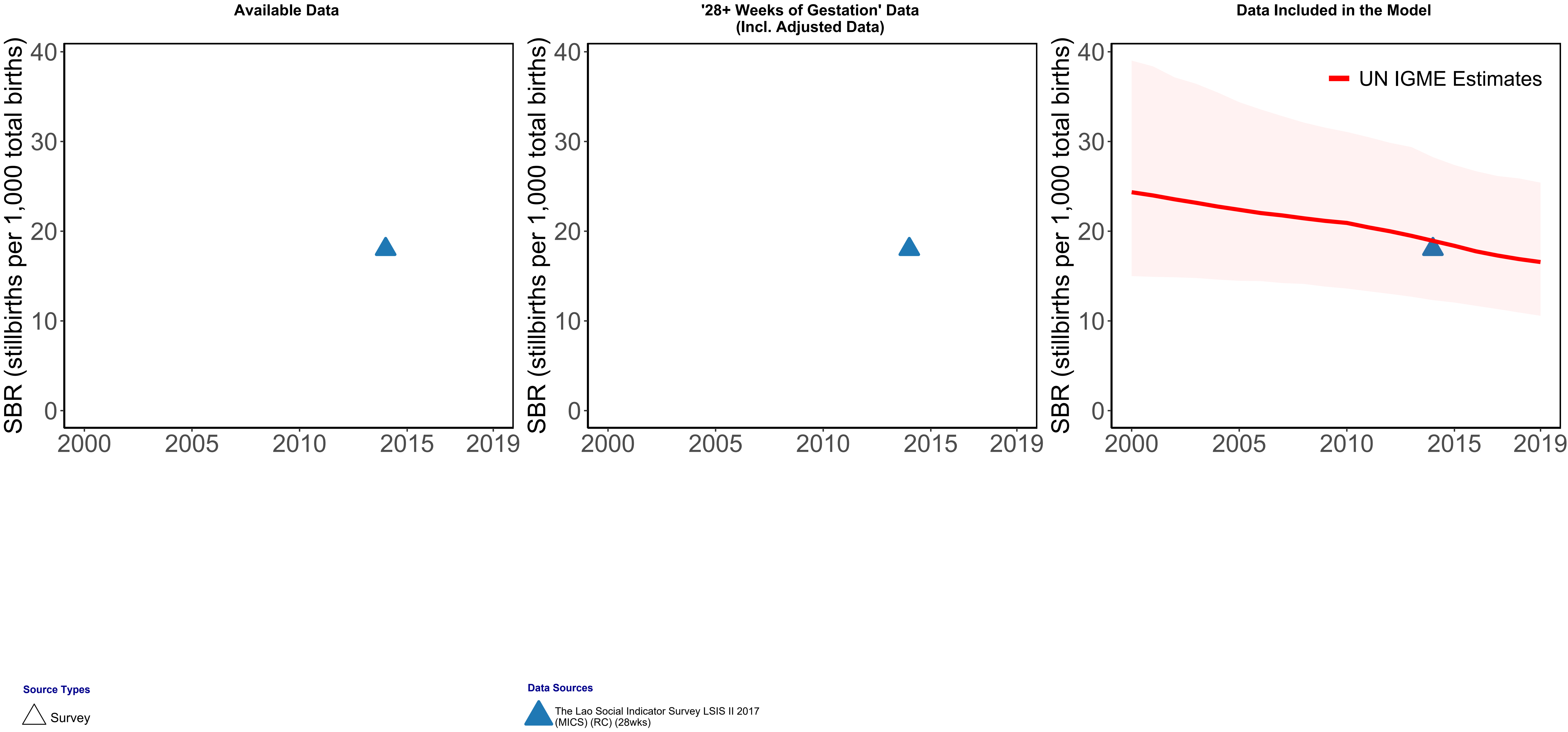

Lebanon

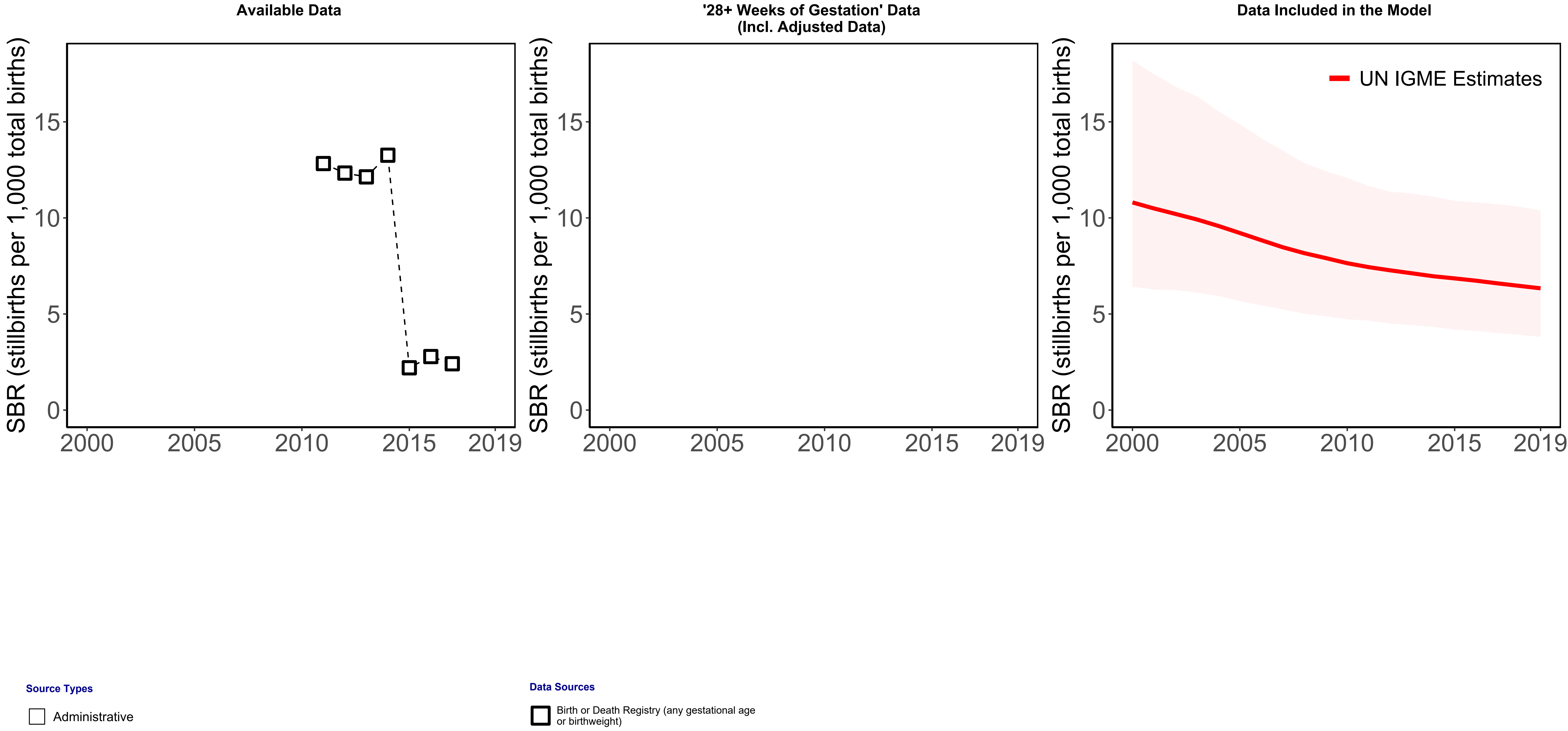

Available Data
'28+ Weeks of Gestation' Data
(Incl. Adjusted Data)
Data Included in the Model
SBR (stillbirths per 1,000 total births)
UN IGME Estimates
0
5
10
15
2000
2005
2010
2015
2019
Source Types
Administrative
Data Sources
Birth or Death Registry (any gestational age
or birthweight)

Liberia

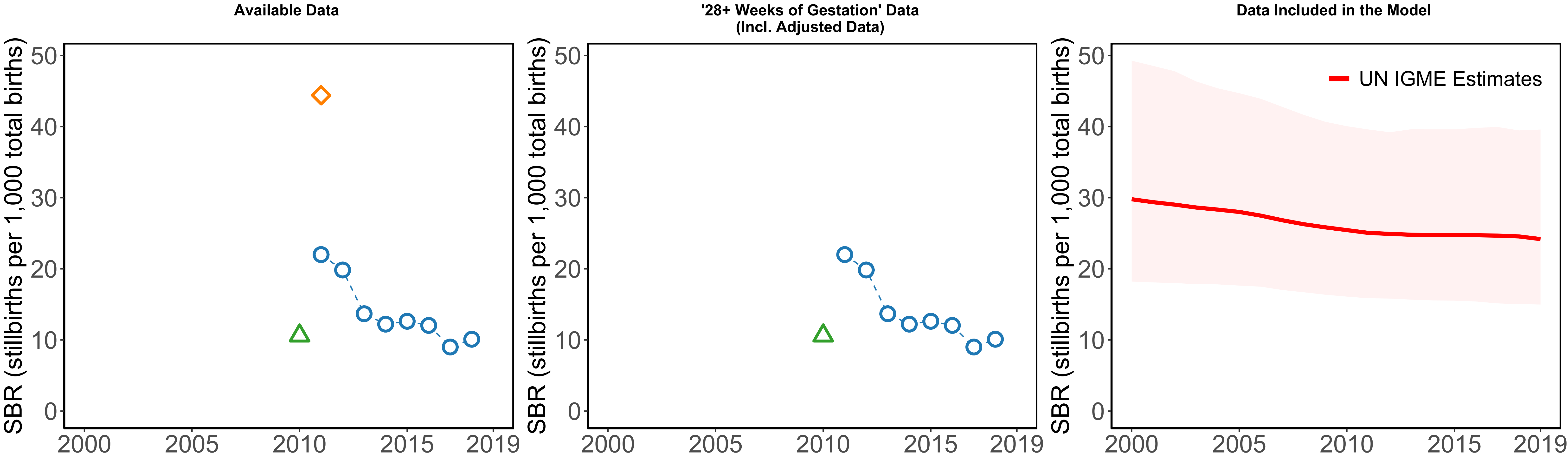

Source Types

HMIS Survey Population study

Data Sources

HMIS-DHIS2 (28wks)

Demographic and Health Survey 2013 (DHS) (RC) (28wks)

Moseson 2014 (1000g and 28wks)

Libya

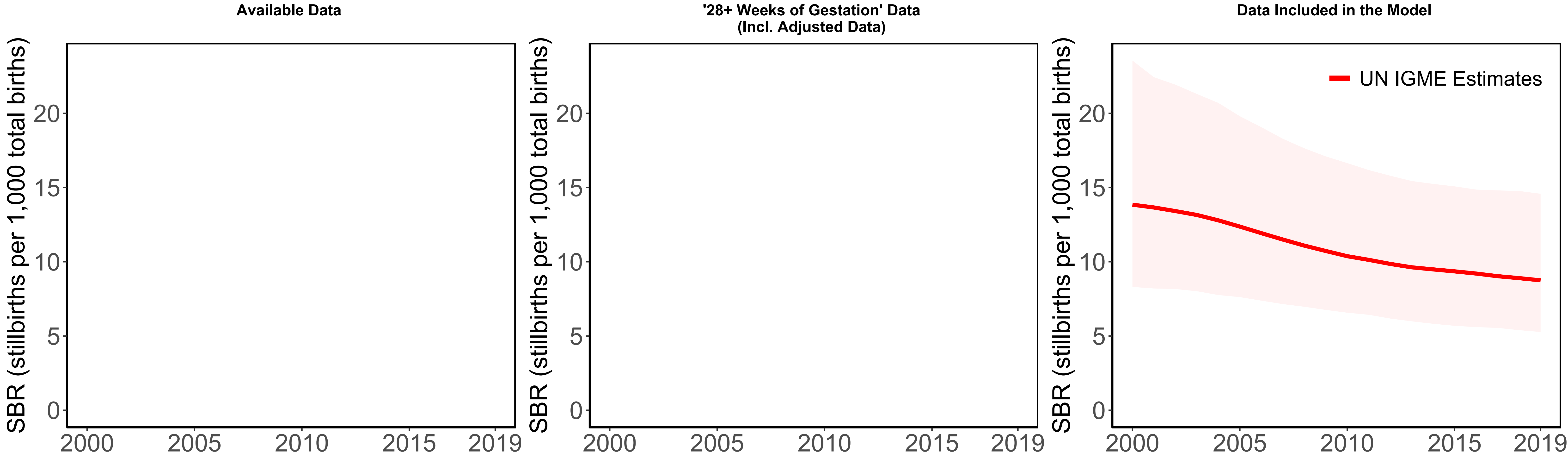
Available Data
'28+ Weeks of Gestation' Data
(Incl. Adjusted Data)
Data Included in the Model
SBR (stillbirths per 1,000 total births)
UN IGME Estimates
0
5
10
15
20
2000
2005
2010
2015
2019

Saint Lucia

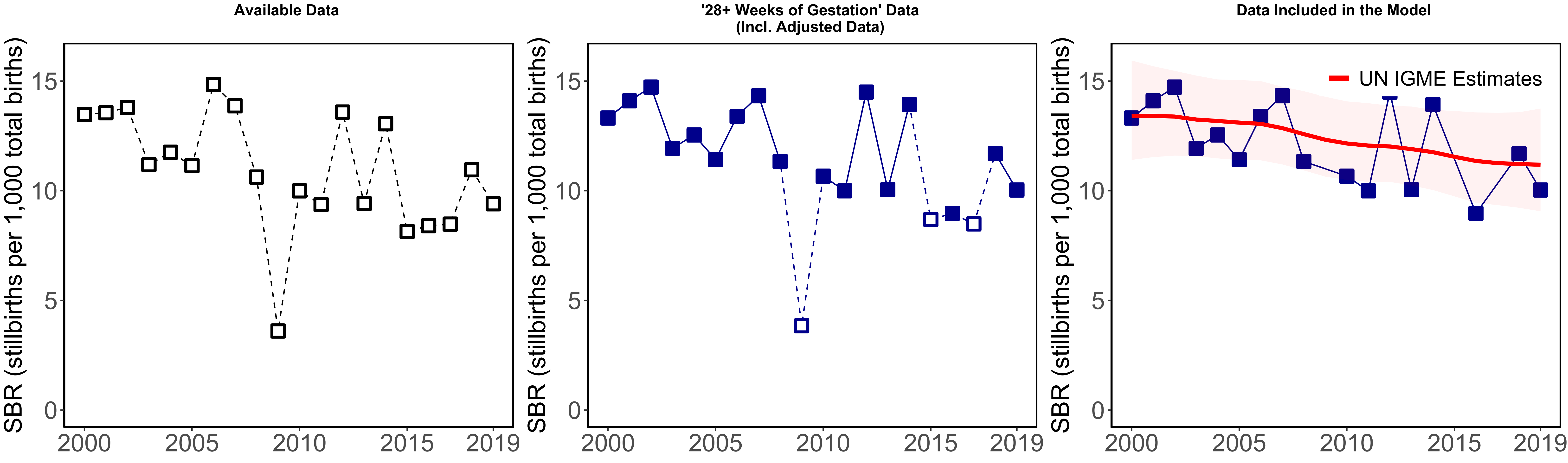
Available Data
'28+ Weeks of Gestation' Data
(Incl. Adjusted Data)
Data Included in the Model
SBR (stillbirths per 1,000 total births)
UN IGME Estimates
0
5
10
15
2000
2005
2010
2015
2019

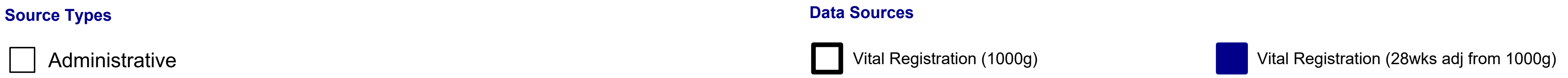
Source Types
Administrative
Data Sources
Vital Registration (1000g)
Vital Registration (28wks adj from 1000g)

Sri Lanka

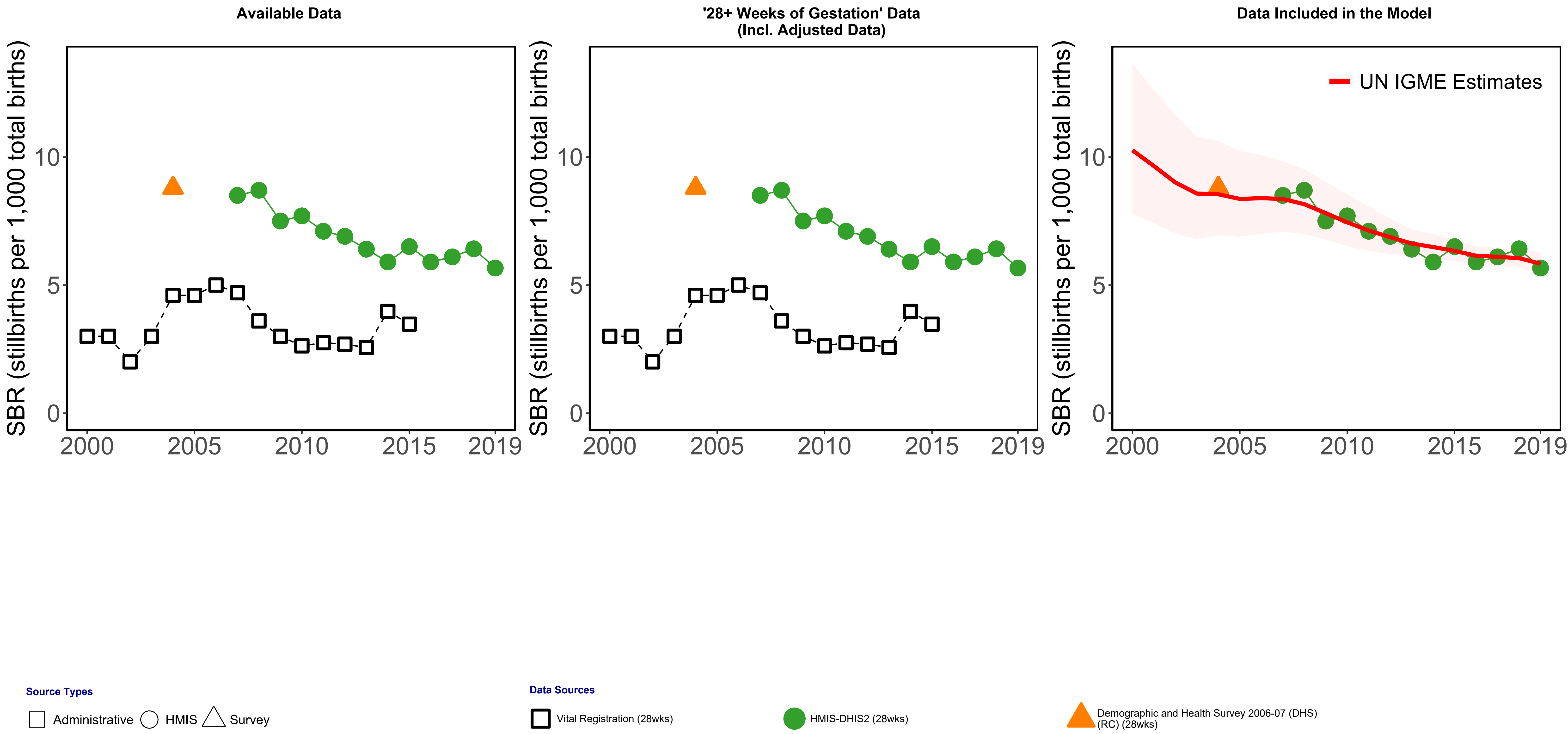

Available Data
'28+ Weeks of Gestation' Data
(Incl. Adjusted Data)
Data Included in the Model
SBR (stillbirths per 1,000 total births)
0
5
10
2000
2005
2010
2015
2019
UN IGME Estimates
Source Types
Administrative
HMIS
Survey
Data Sources
Vital Registration (28wks)
HMIS-DHIS2 (28wks)
Demographic and Health Survey 2006-07 (DHS) (RC) (28wks)

Lesotho

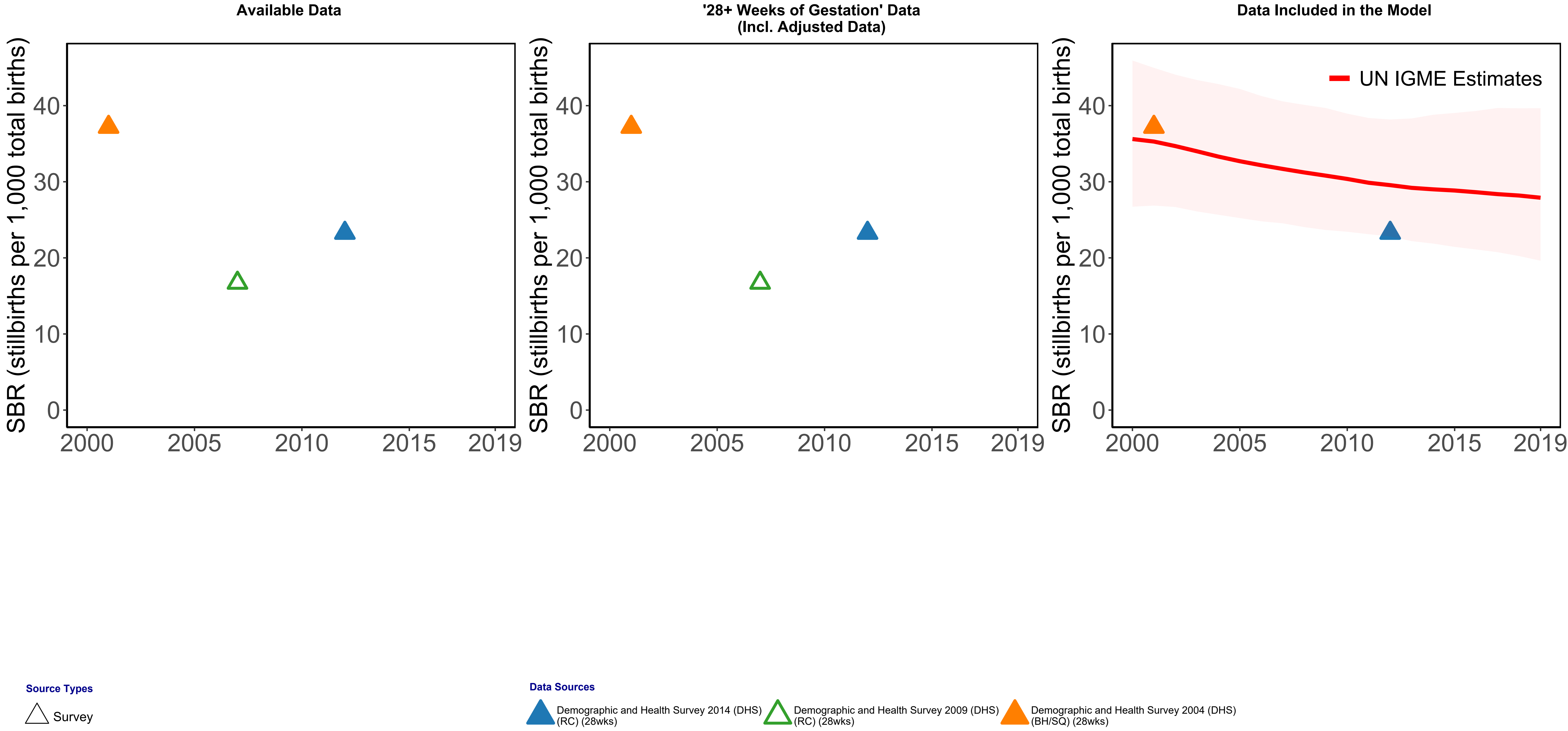

Available Data
'28+ Weeks of Gestation' Data
(Incl. Adjusted Data)
Data Included in the Model
SBR (stillbirths per 1,000 total births)
UN IGME Estimates
0
10
20
30
40
2000
2005
2010
2015
2019
Source Types
Survey
Data Sources
Demographic and Health Survey 2014 (DHS) (RC) (28wks)
Demographic and Health Survey 2009 (DHS) (RC) (28wks)
Demographic and Health Survey 2004 (DHS) (BH/SQ) (28wks)

Lithuania

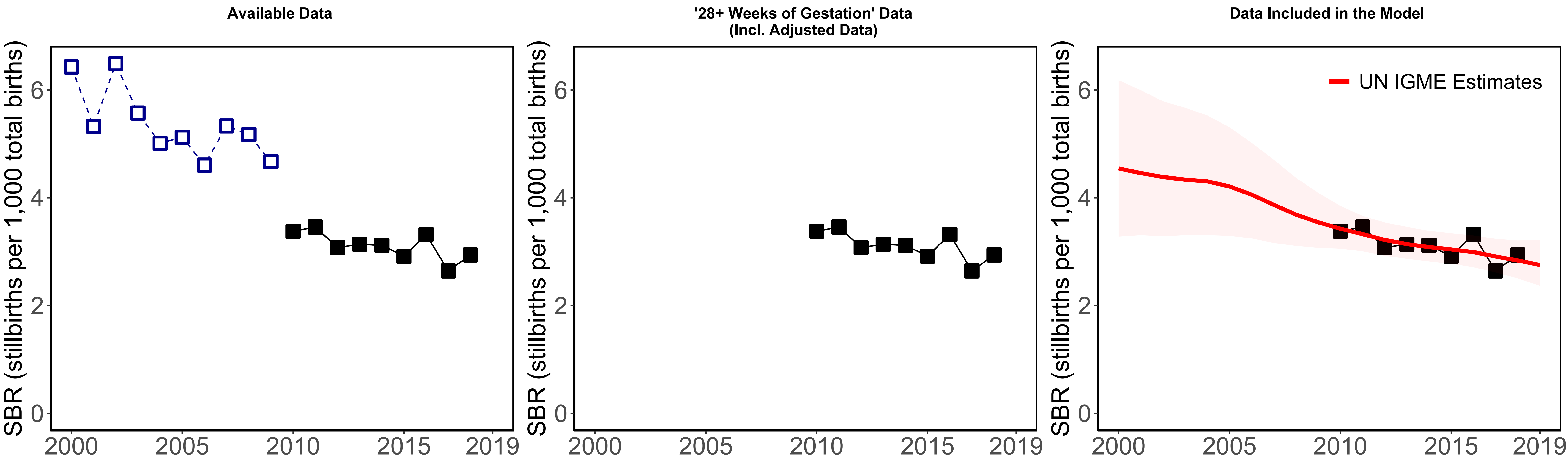

Available Data
'28+ Weeks of Gestation' Data
(Incl. Adjusted Data)
Data Included in the Model
UN IGME Estimates
SBR (stillbirths per 1,000 total births)
0
2
4
6
2000
2005
2010
2015
2019

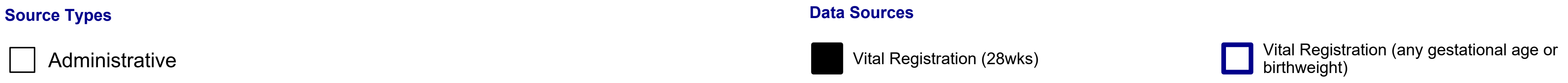

Source Types
Administrative
Data Sources
Vital Registration (28wks)
Vital Registration (any gestational age or birthweight)

Luxembourg

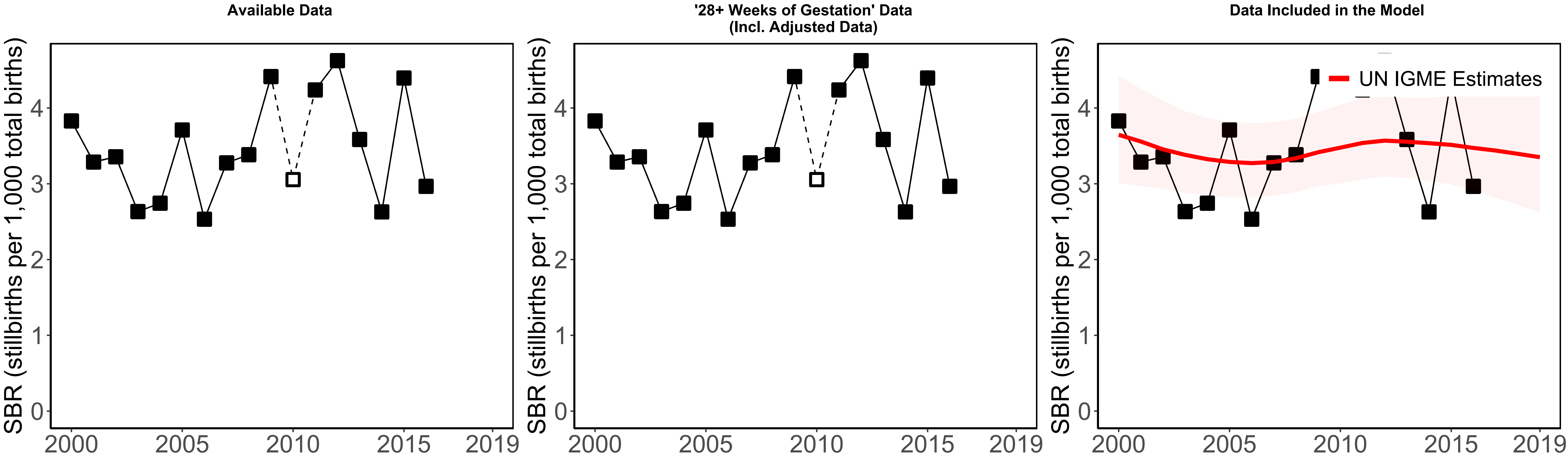

Available Data
'28+ Weeks of Gestation' Data
(Incl. Adjusted Data)
Data Included in the Model
UN IGME Estimates
SBR (stillbirths per 1,000 total births)
0
1
2
3
4
2000
2005
2010
2015
2019

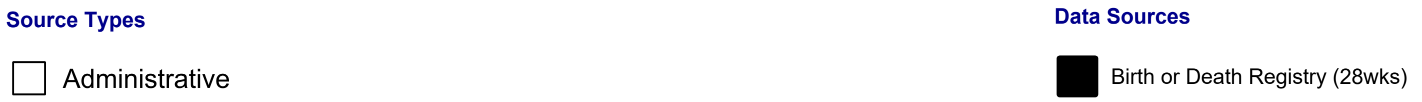

Source Types
Administrative
Data Sources
Birth or Death Registry (28wks)

Latvia

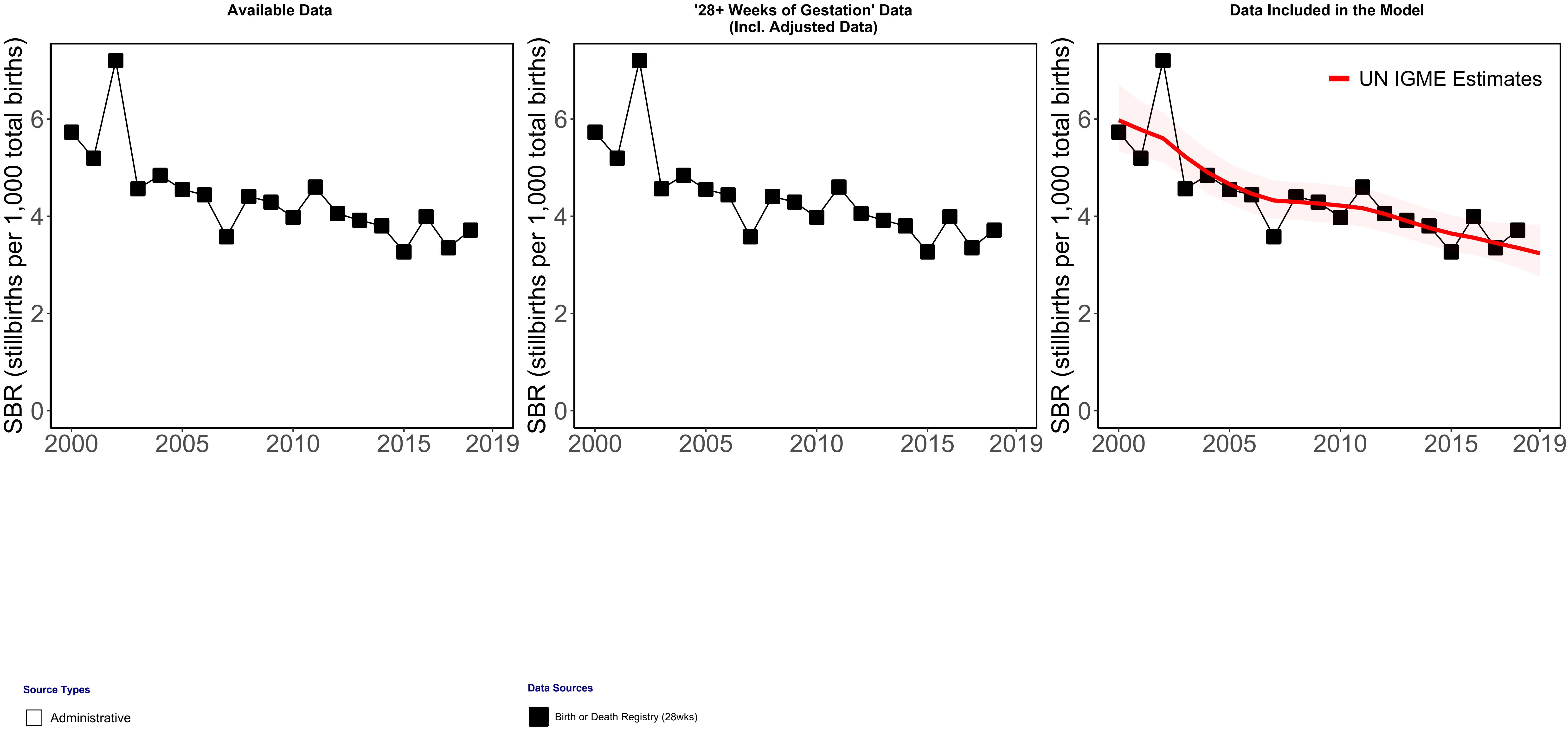
Available Data
'28+ Weeks of Gestation' Data
(Incl. Adjusted Data)
Data Included in the Model
UN IGME Estimates
SBR (stillbirths per 1,000 total births)
0
2
4
6
2000
2005
2010
2015
2019
Source Types
Administrative
Data Sources
Birth or Death Registry (28wks)

Morocco

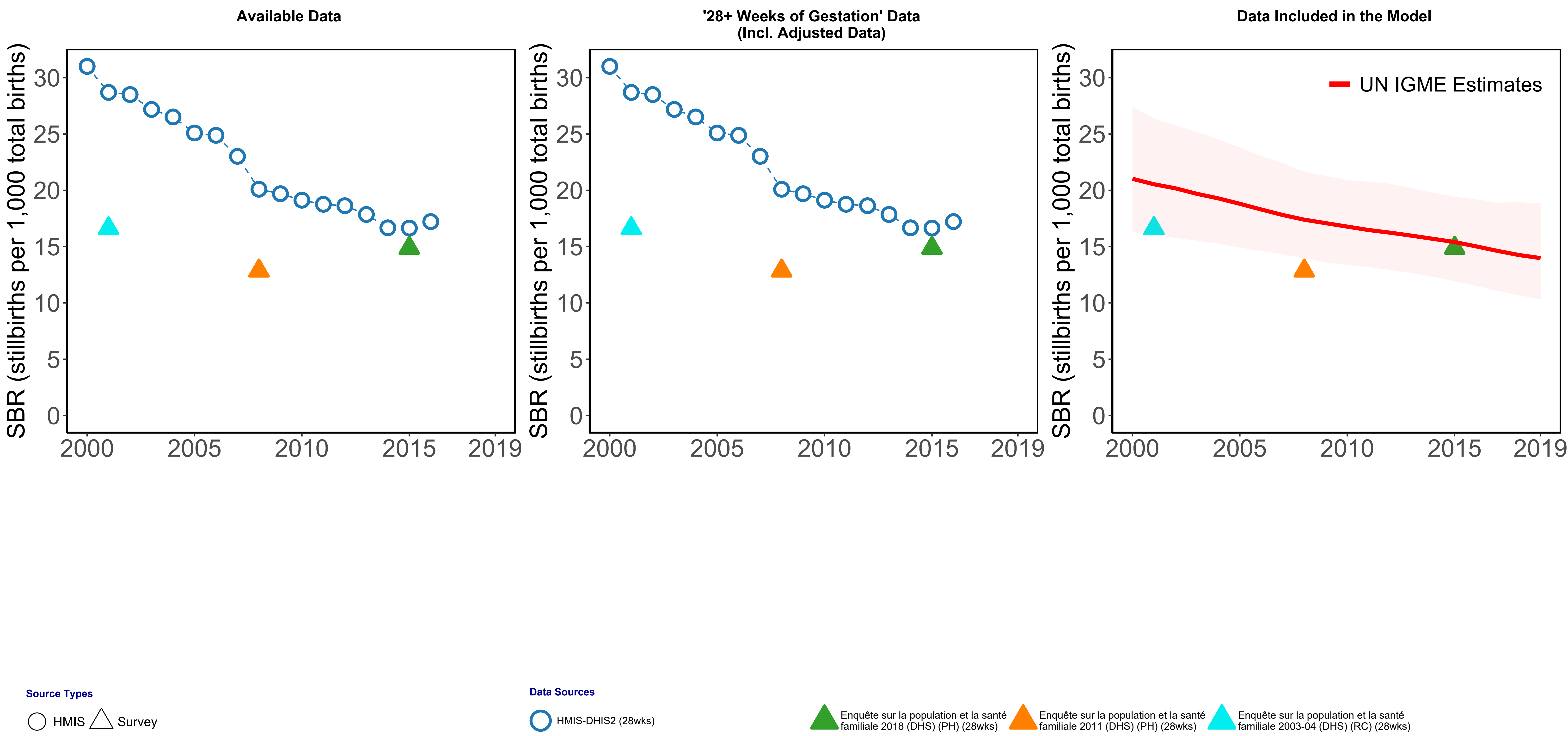

Available Data
'28+ Weeks of Gestation' Data
(Incl. Adjusted Data)
Data Included in the Model
SBR (stillbirths per 1,000 total births)
0
5
10
15
20
25
30
2000
2005
2010
2015
2019
UN IGME Estimates
Source Types
HMIS
Survey
Data Sources
HMIS-DHIS2 (28wks)
Enquête sur la population et la santé familiale 2018 (DHS) (PH) (28wks)
Enquête sur la population et la santé familiale 2011 (DHS) (PH) (28wks)
Enquête sur la population et la santé familiale 2003-04 (DHS) (RC) (28wks)

Monaco

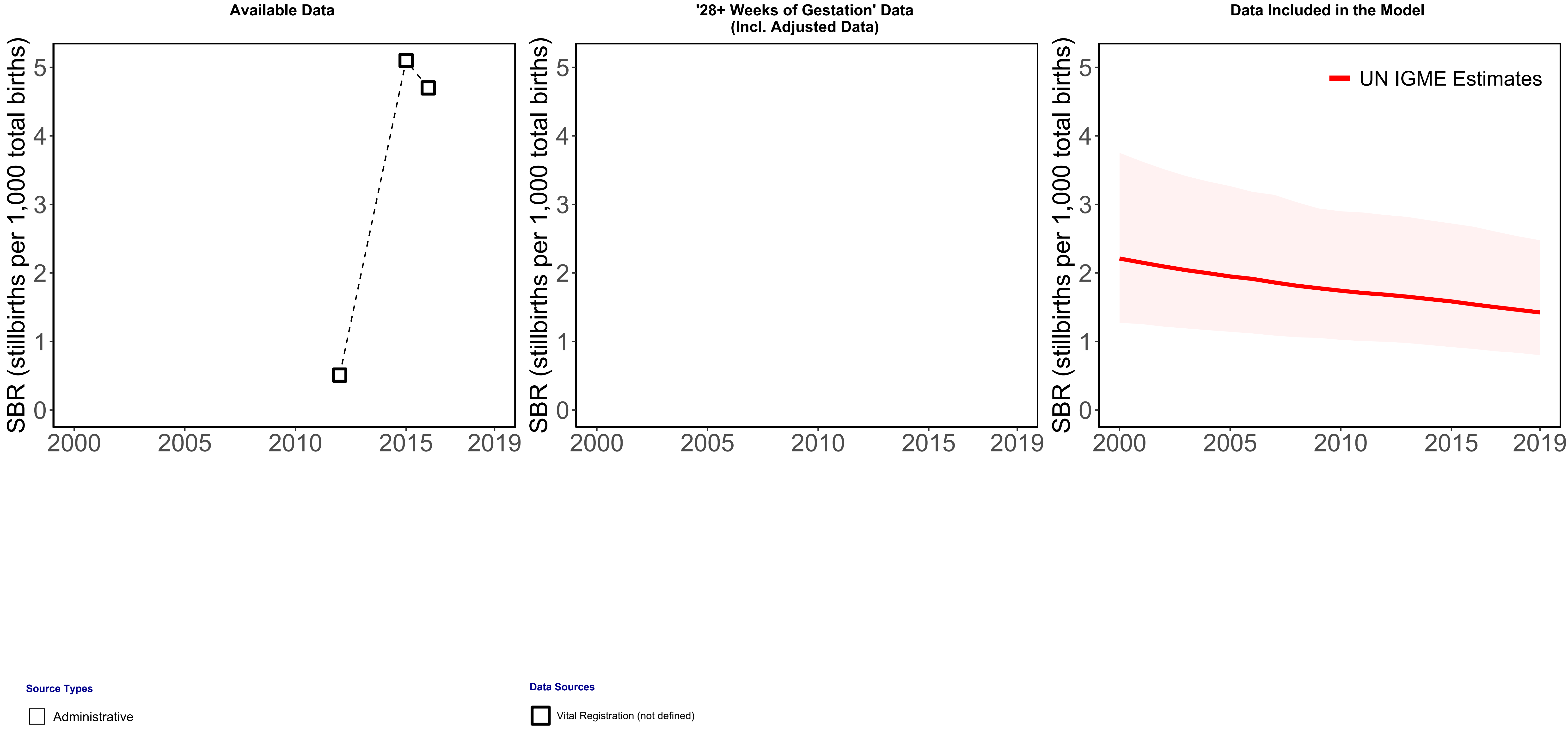
Available Data
'28+ Weeks of Gestation' Data
(Incl. Adjusted Data)
Data Included in the Model
SBR (stillbirths per 1,000 total births)
0
1
2
3
4
5
2000
2005
2010
2015
2019
UN IGME Estimates
Source Types
Administrative
Data Sources
Vital Registration (not defined)

Republic of Moldova

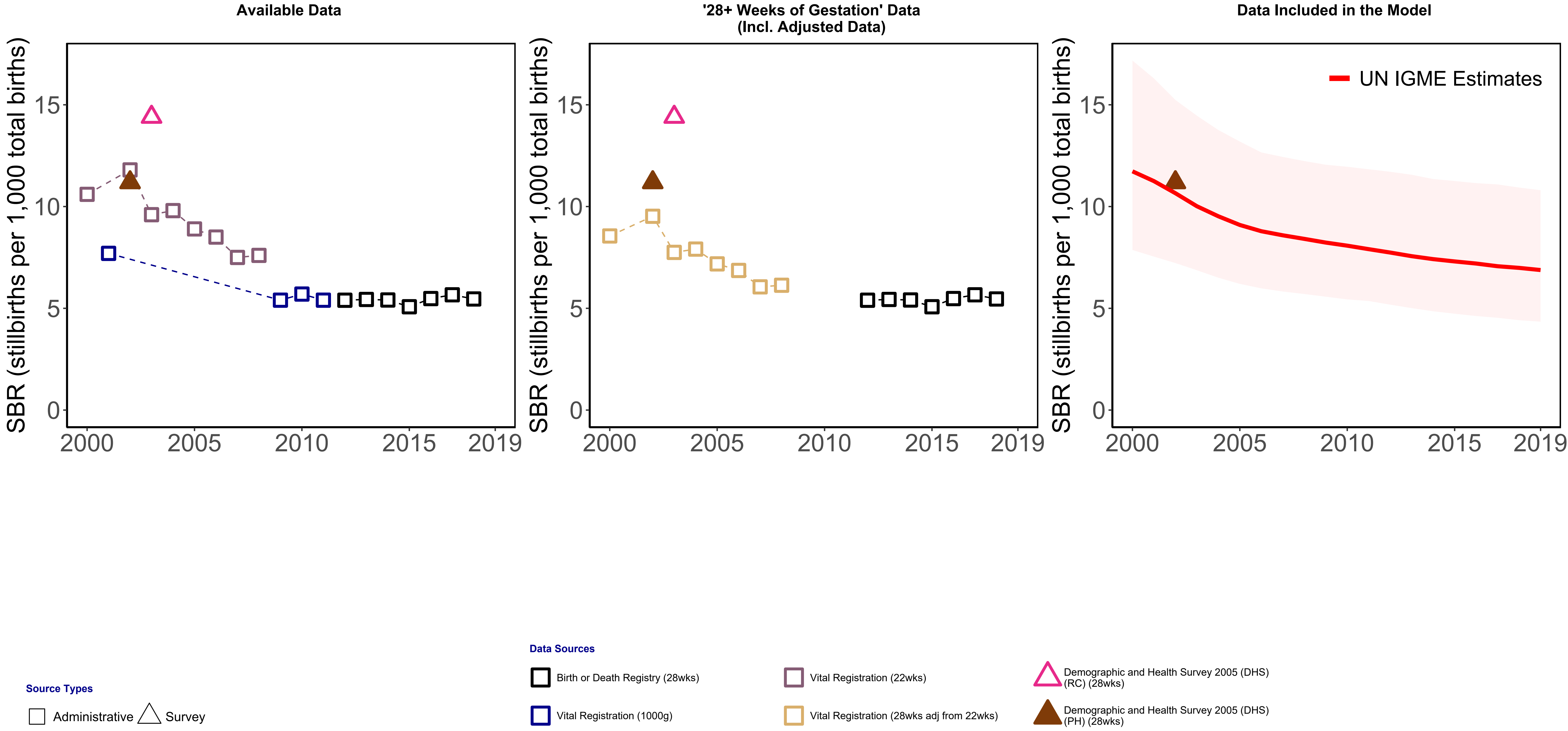

Madagascar

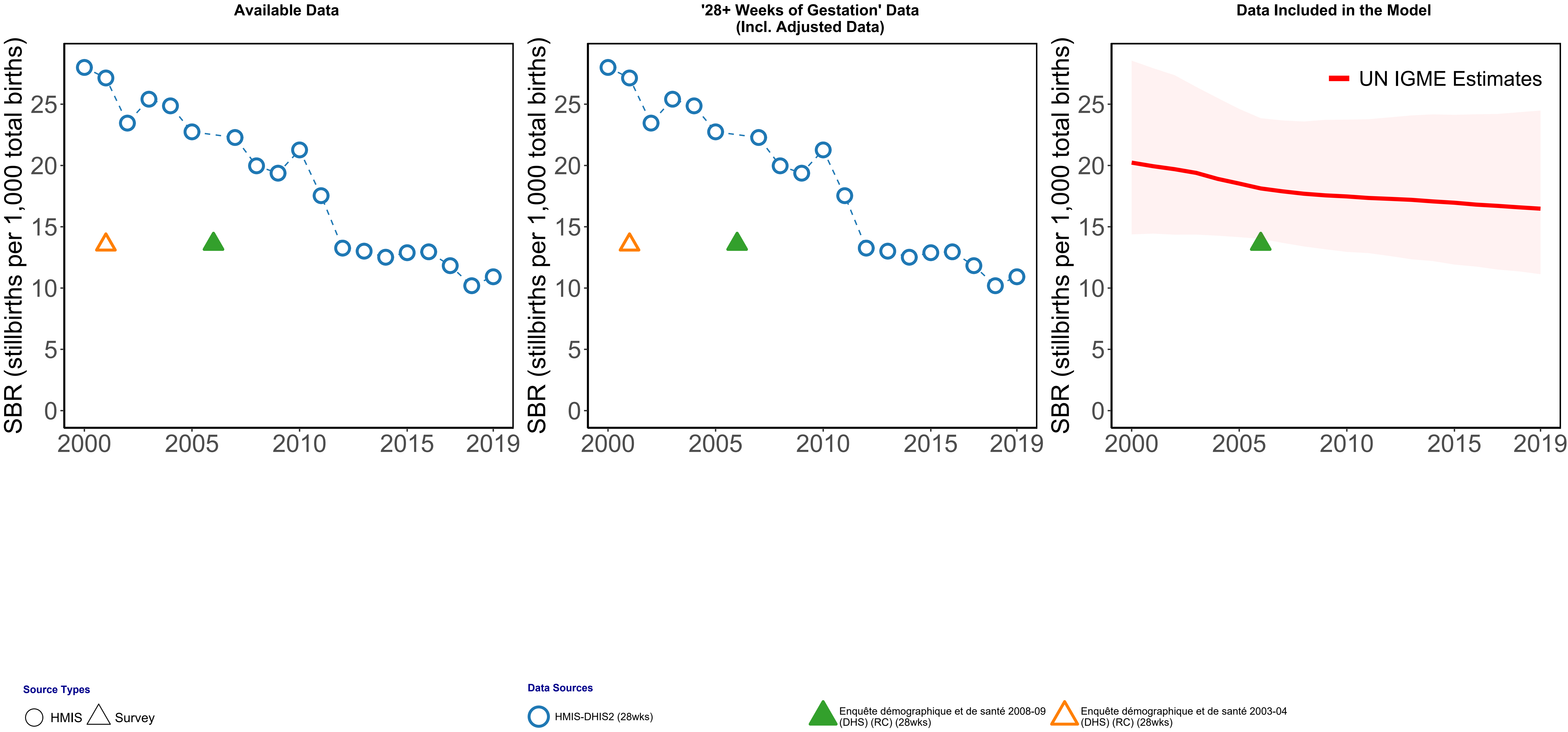

Available Data
'28+ Weeks of Gestation' Data
(Incl. Adjusted Data)
Data Included in the Model
UN IGME Estimates
SBR (stillbirths per 1,000 total births)
0
5
10
15
20
25
2000
2005
2010
2015
2019
Source Types
HMIS
Survey
Data Sources
HMIS-DHIS2 (28wks)
Enquête démographique et de santé 2008-09 (DHS) (RC) (28wks)
Enquête démographique et de santé 2003-04 (DHS) (RC) (28wks)

Maldives

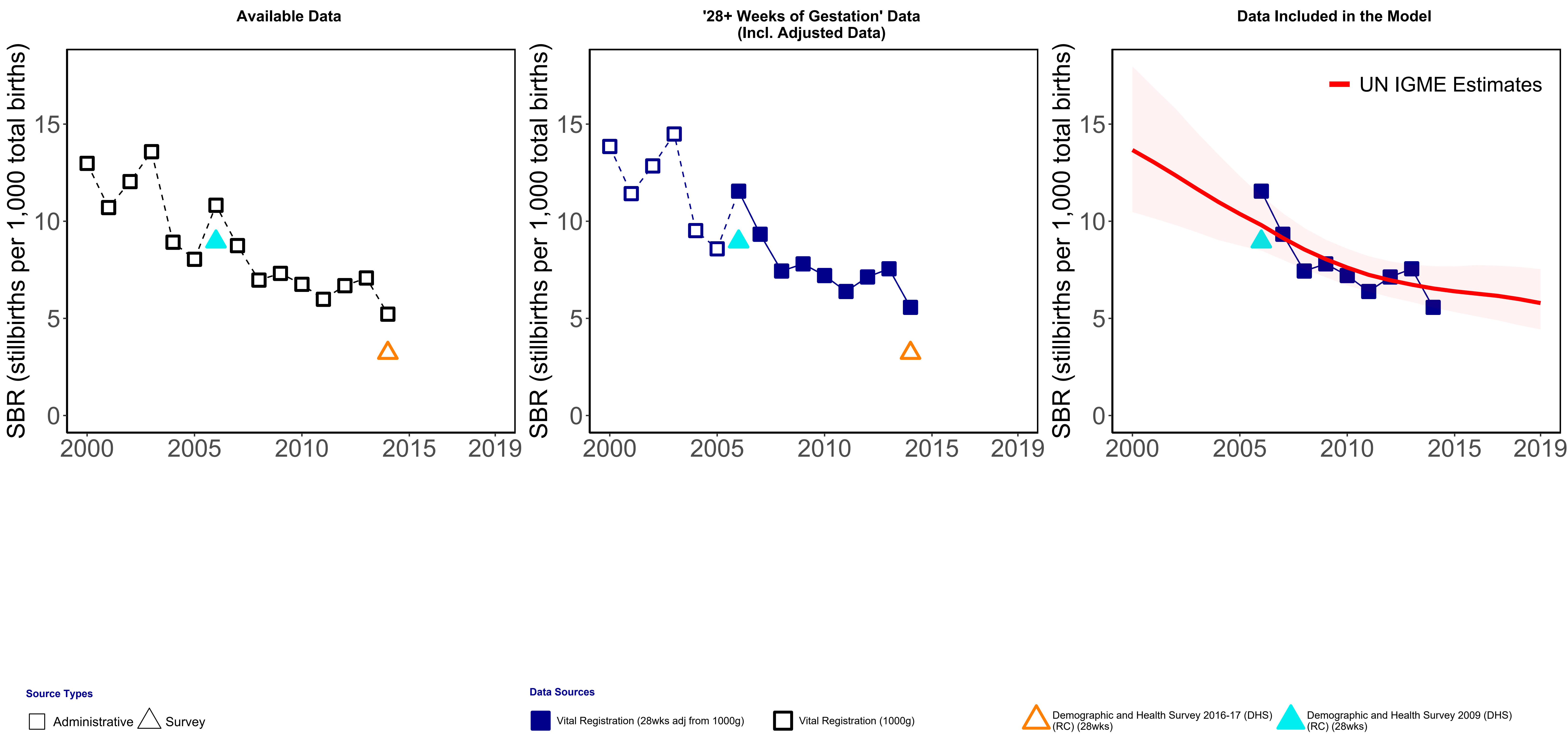

Available Data
'28+ Weeks of Gestation' Data
(Incl. Adjusted Data)
Data Included in the Model
SBR (stillbirths per 1,000 total births)
UN IGME Estimates
0
5
10
15
2000
2005
2010
2015
2019
Source Types
Administrative
Survey
Data Sources
Vital Registration (28wks adj from 1000g)
Vital Registration (1000g)
Demographic and Health Survey 2016-17 (DHS) (RC) (28wks)
Demographic and Health Survey 2009 (DHS) (RC) (28wks)

Mexico

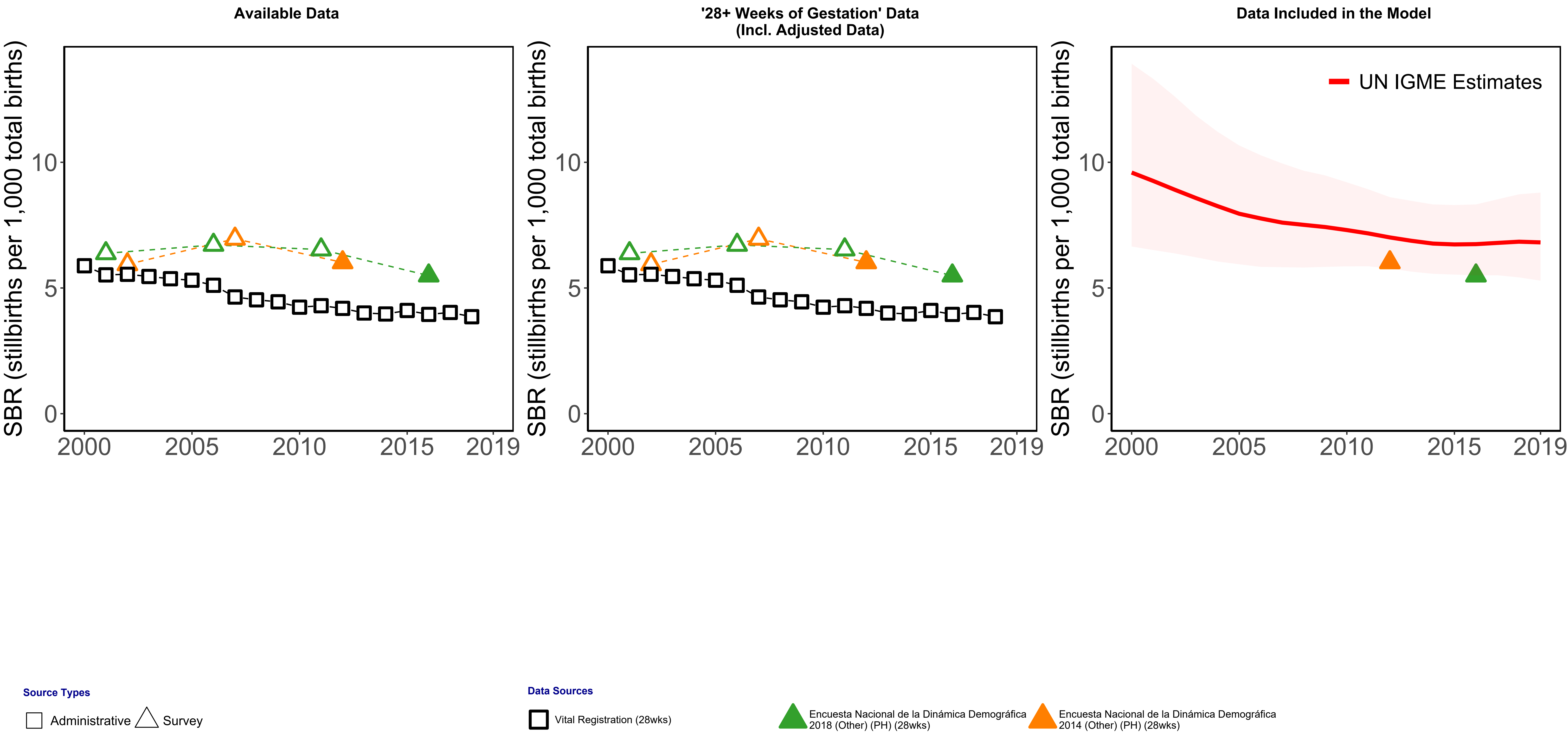

Available Data
'28+ Weeks of Gestation' Data
(Incl. Adjusted Data)
Data Included in the Model
SBR (stillbirths per 1,000 total births)
0
5
10
2000
2005
2010
2015
2019
UN IGME Estimates
Source Types
Administrative
Survey
Data Sources
Vital Registration (28wks)
Encuesta Nacional de la Dinámica Demográfica 2018 (Other) (PH) (28wks)
Encuesta Nacional de la Dinámica Demográfica 2014 (Other) (PH) (28wks)

Marshall Islands

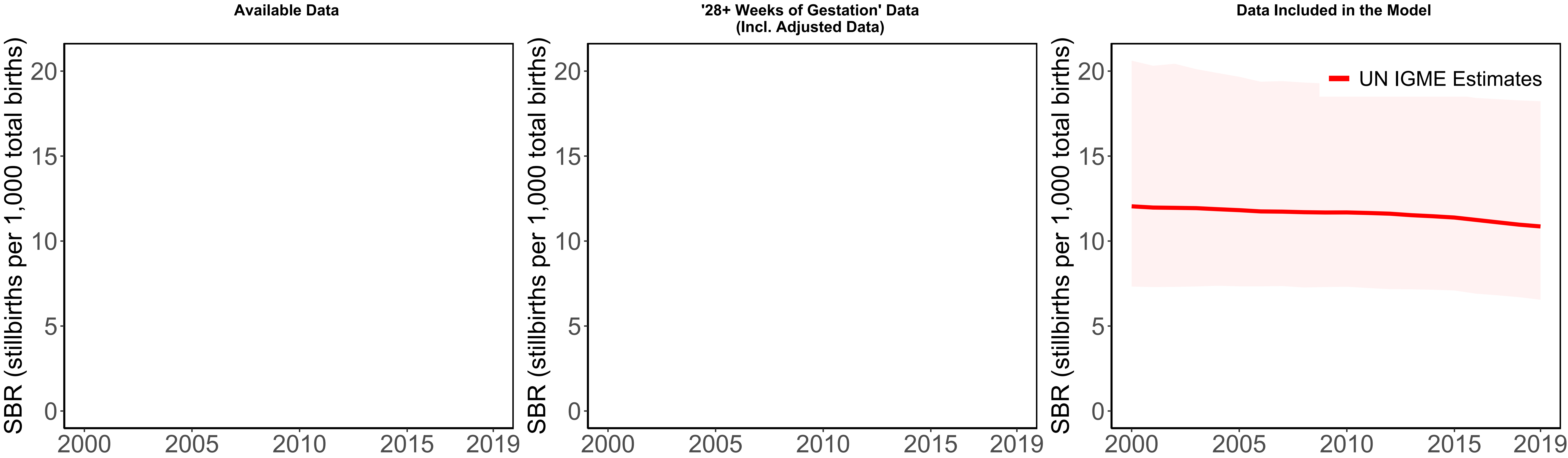

Republic of North Macedonia

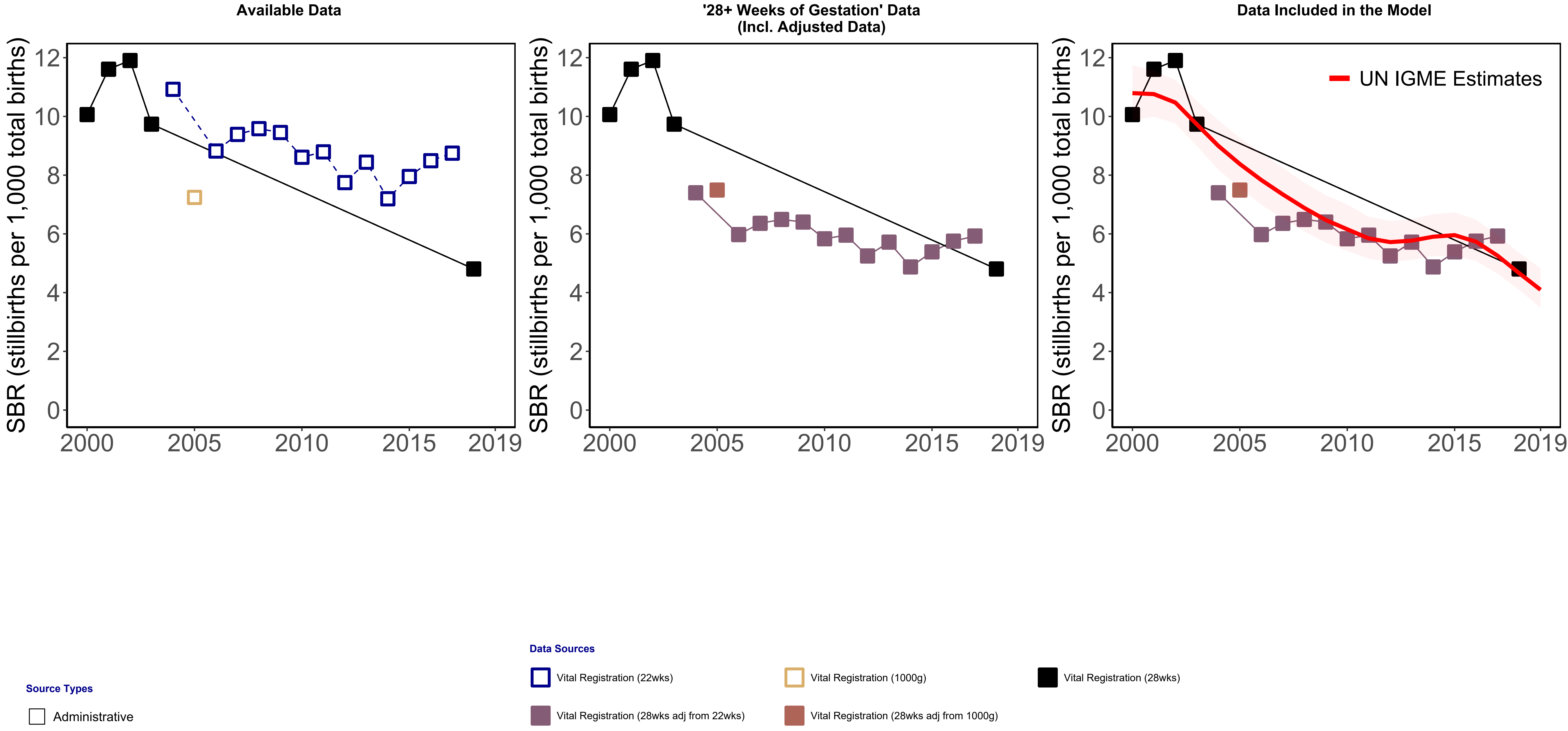

Mali

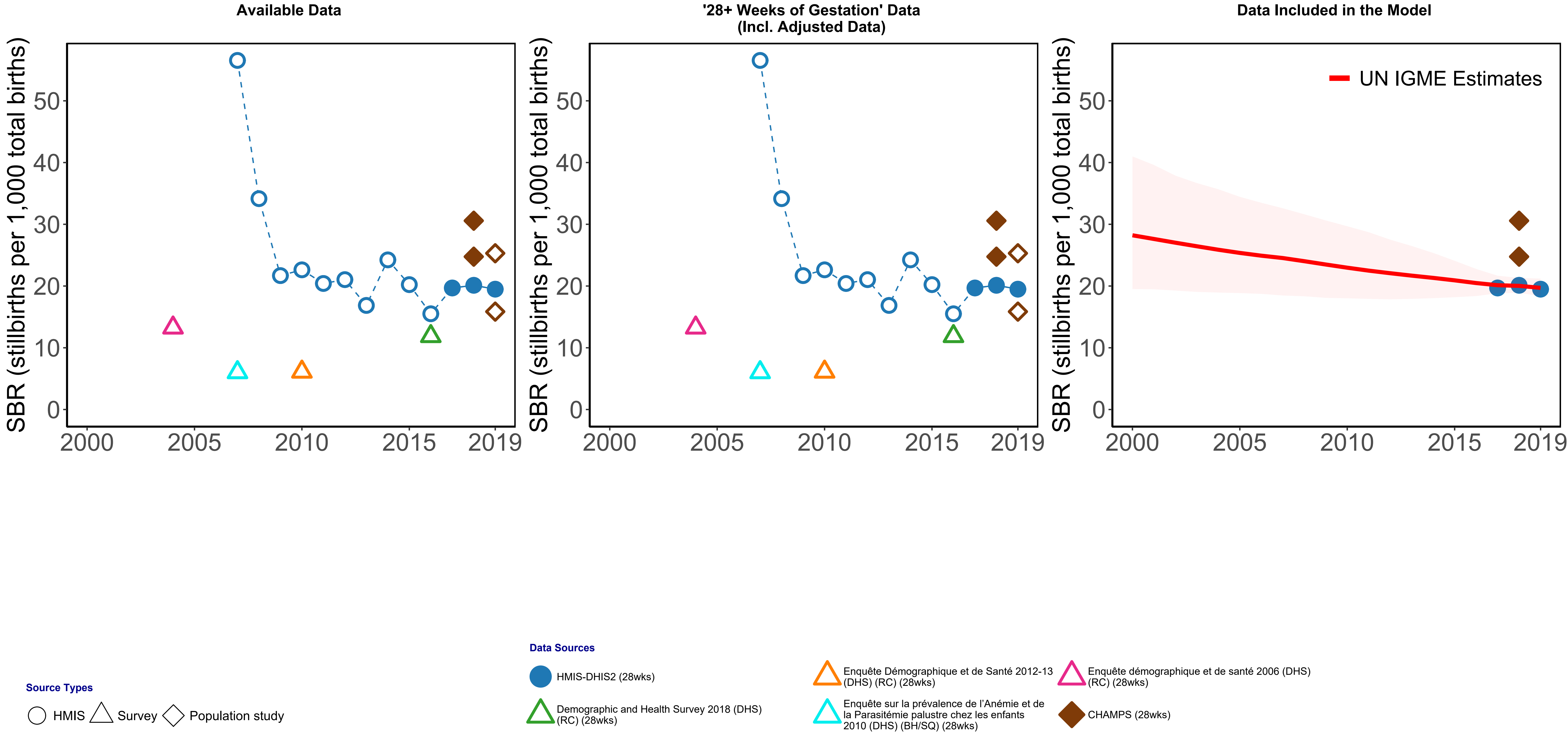
Available Data
'28+ Weeks of Gestation' Data
(Incl. Adjusted Data)
Data Included in the Model
SBR (stillbirths per 1,000 total births)
UN IGME Estimates
0
10
20
30
40
50
2000
2005
2010
2015
2019
Source Types
HMIS
Survey
Population study
Data Sources
HMIS-DHIS2 (28wks)
Demographic and Health Survey 2018 (DHS) (RC) (28wks)
Enquête Démographique et de Santé 2012-13 (DHS) (RC) (28wks)
Enquête sur la prévalence de l'Anémie et de la Parasitémie palustre chez les enfants 2010 (DHS) (BH/SQ) (28wks)
Enquête démographique et de santé 2006 (DHS) (RC) (28wks)
CHAMPS (28wks)

Malta

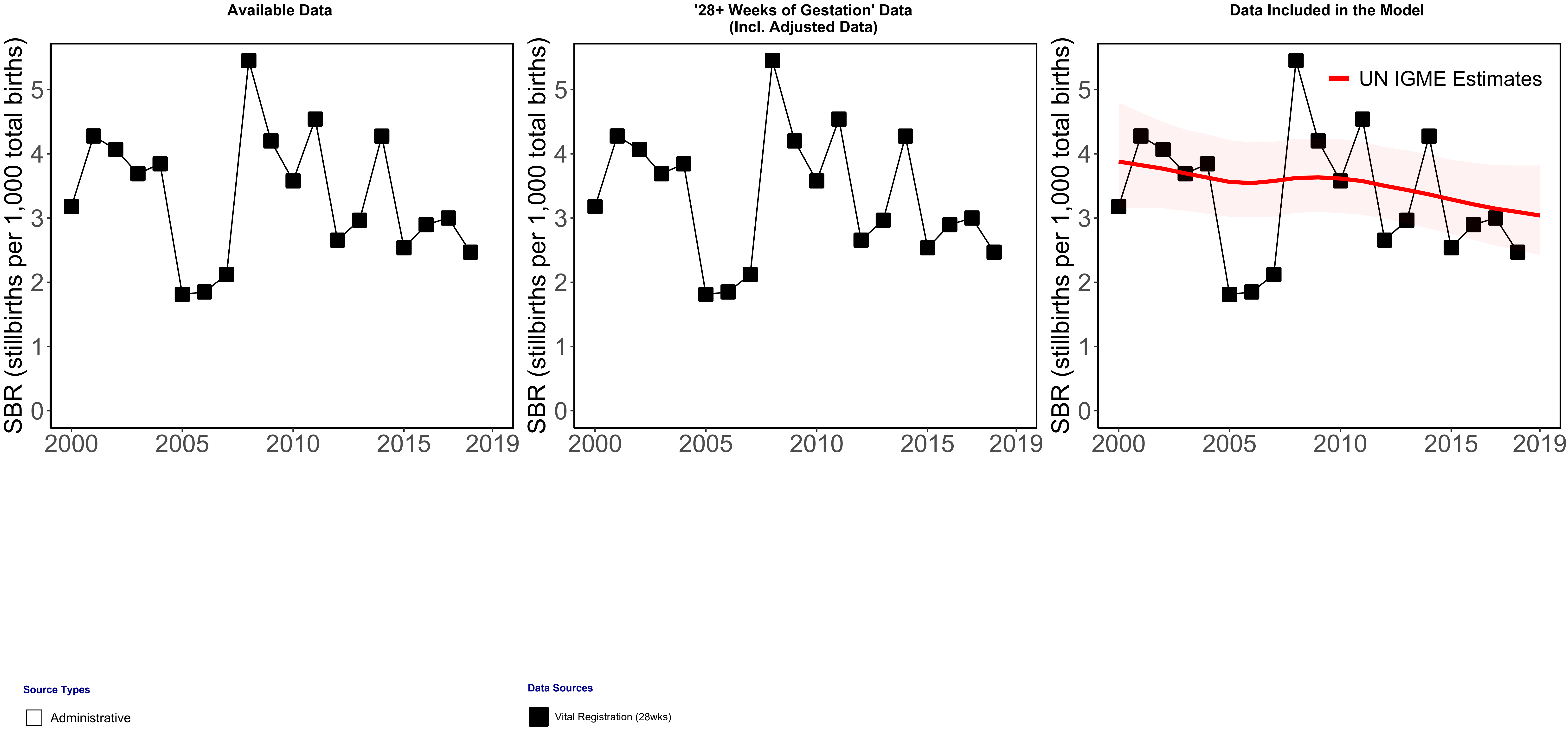
Available Data
'28+ Weeks of Gestation' Data
(Incl. Adjusted Data)
Data Included in the Model
SBR (stillbirths per 1,000 total births)
0
1
2
3
4
5
2000
2005
2010
2015
2019
UN IGME Estimates
Source Types
Administrative
Data Sources
Vital Registration (28wks)

Myanmar

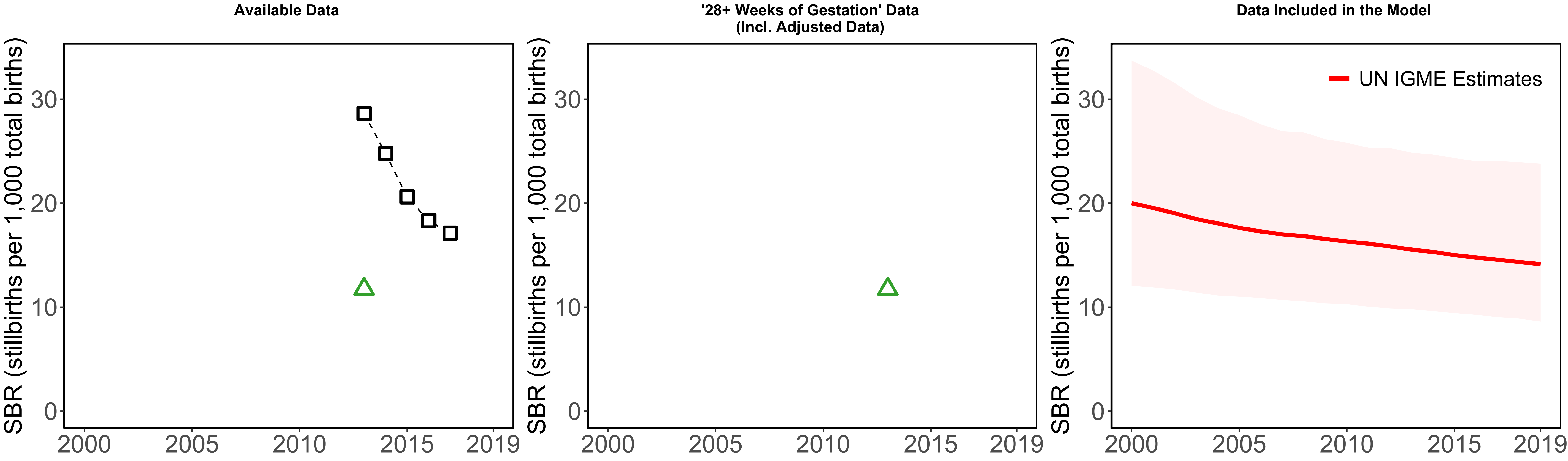

Source Types

Administrative Survey

Data Sources

Vital Registration (not defined)

Demographic and Health Survey 2015-16 (DHS) (RC) (28wks)

Montenegro

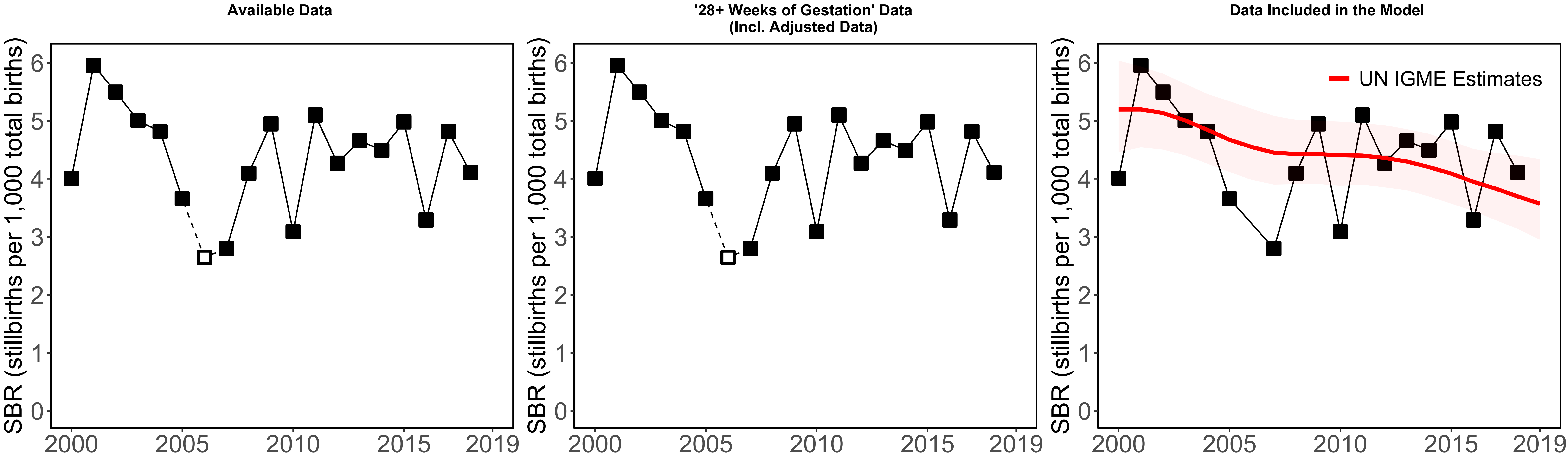
Available Data
'28+ Weeks of Gestation' Data
(Incl. Adjusted Data)
Data Included in the Model
UN IGME Estimates
SBR (stillbirths per 1,000 total births)
0
1
2
3
4
5
6
2000
2005
2010
2015
2019

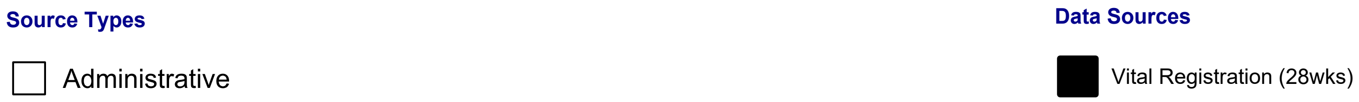
Source Types
Administrative
Data Sources
Vital Registration (28wks)

Mongolia

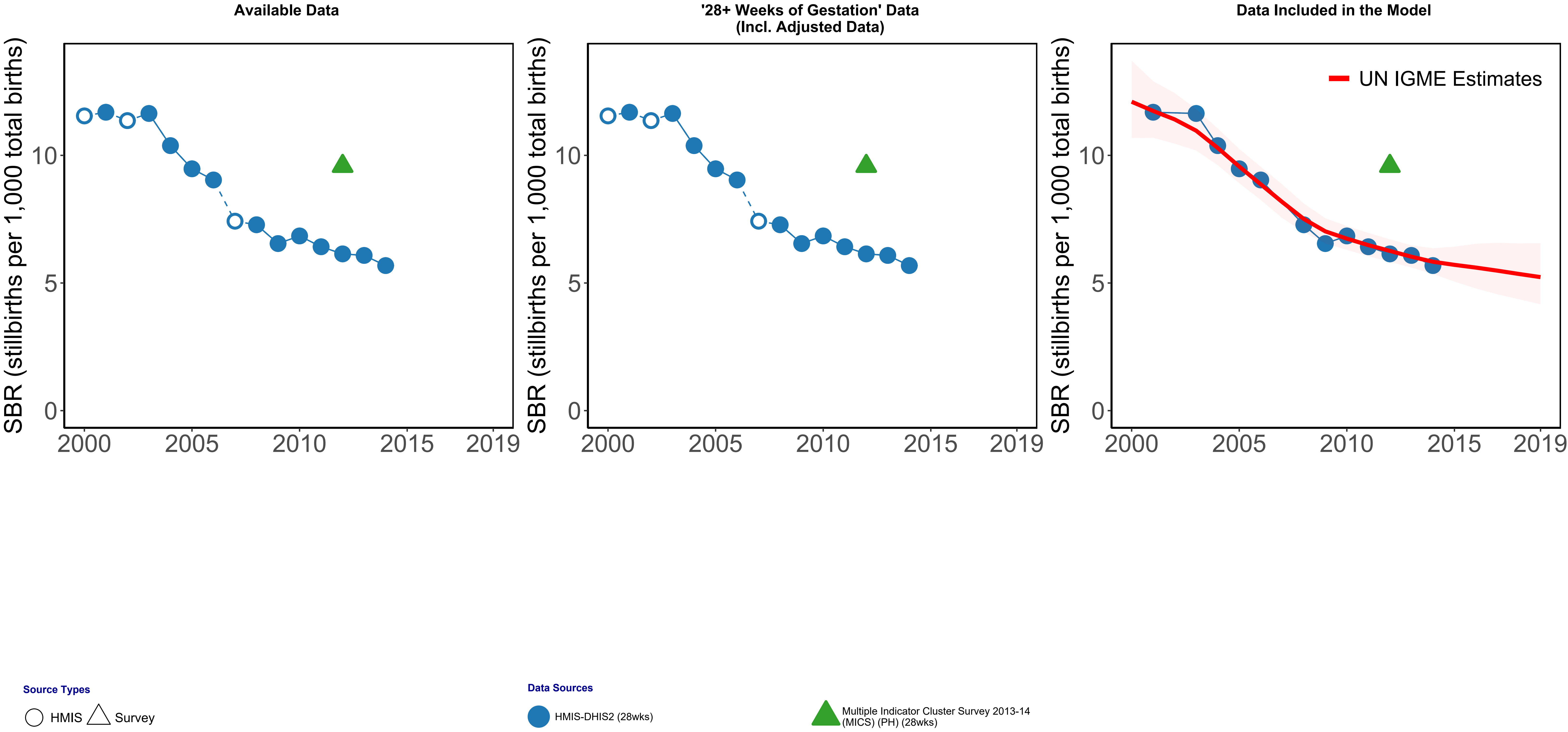

Available Data
'28+ Weeks of Gestation' Data
(Incl. Adjusted Data)
Data Included in the Model
SBR (stillbirths per 1,000 total births)
0
5
10
2000
2005
2010
2015
2019
UN IGME Estimates
Source Types
HMIS
Survey
Data Sources
HMIS-DHIS2 (28wks)
Multiple Indicator Cluster Survey 2013-14
(MICS) (PH) (28wks)

Mozambique

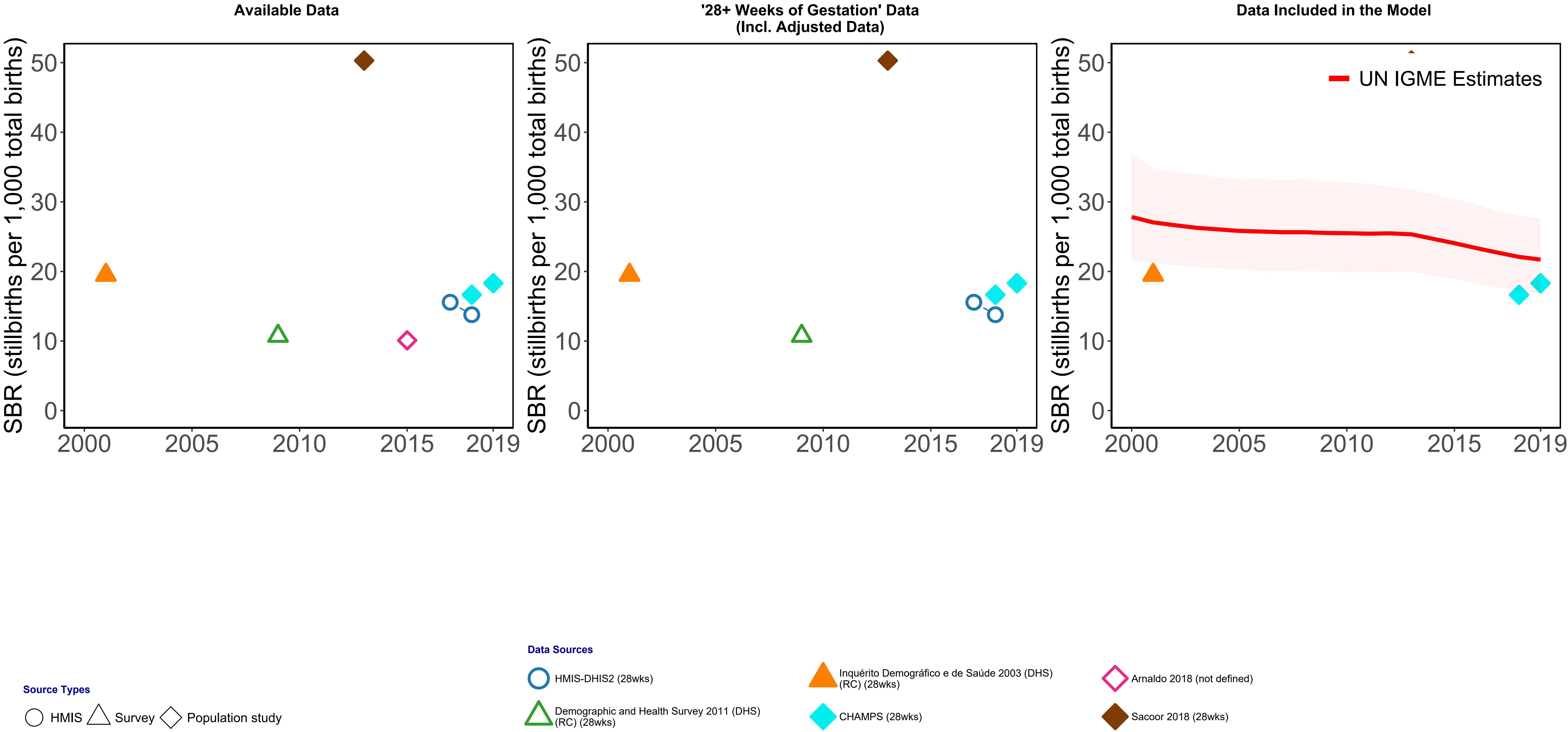
Available Data
'28+ Weeks of Gestation' Data
(Incl. Adjusted Data)
Data Included in the Model
SBR (stillbirths per 1,000 total births)
0
10
20
30
40
50
2000
2005
2010
2015
2019
UN IGME Estimates
Source Types
HMIS
Survey
Population study
Data Sources
HMIS-DHIS2 (28wks)
Demographic and Health Survey 2011 (DHS) (RC) (28wks)
Inquérito Demográfico e de Saúde 2003 (DHS) (RC) (28wks)
CHAMPS (28wks)
Arnaldo 2018 (not defined)
Sacoor 2018 (28wks)

Mauritania

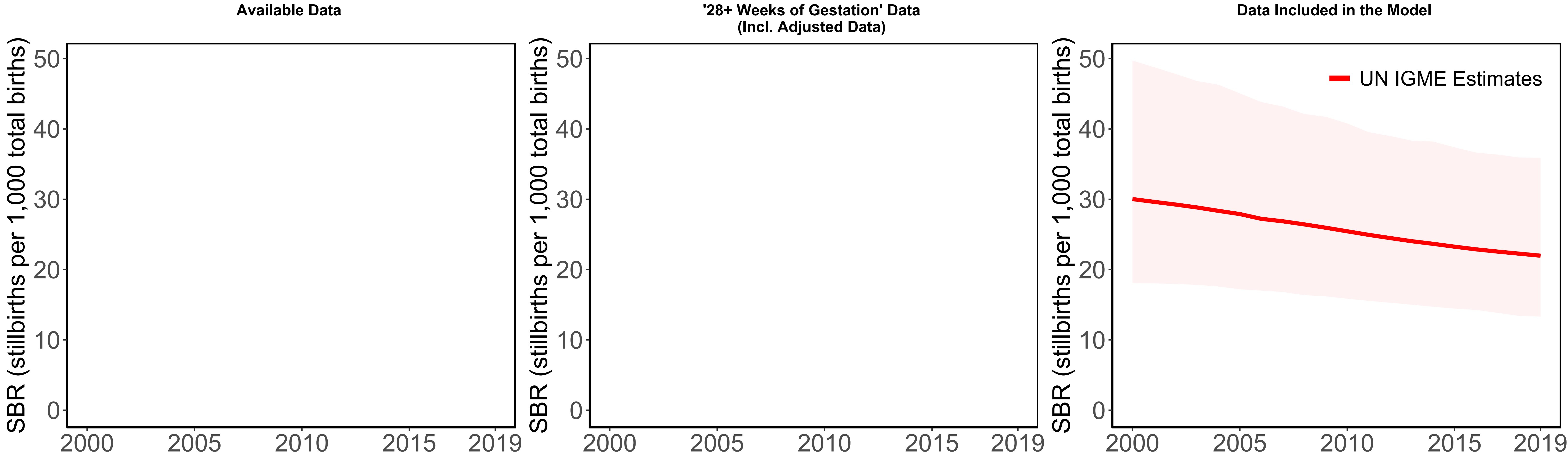

Available Data
'28+ Weeks of Gestation' Data
(Incl. Adjusted Data)
Data Included in the Model
SBR (stillbirths per 1,000 total births)
0
10
20
30
40
50
2000
2005
2010
2015
2019
UN IGME Estimates

Mauritius

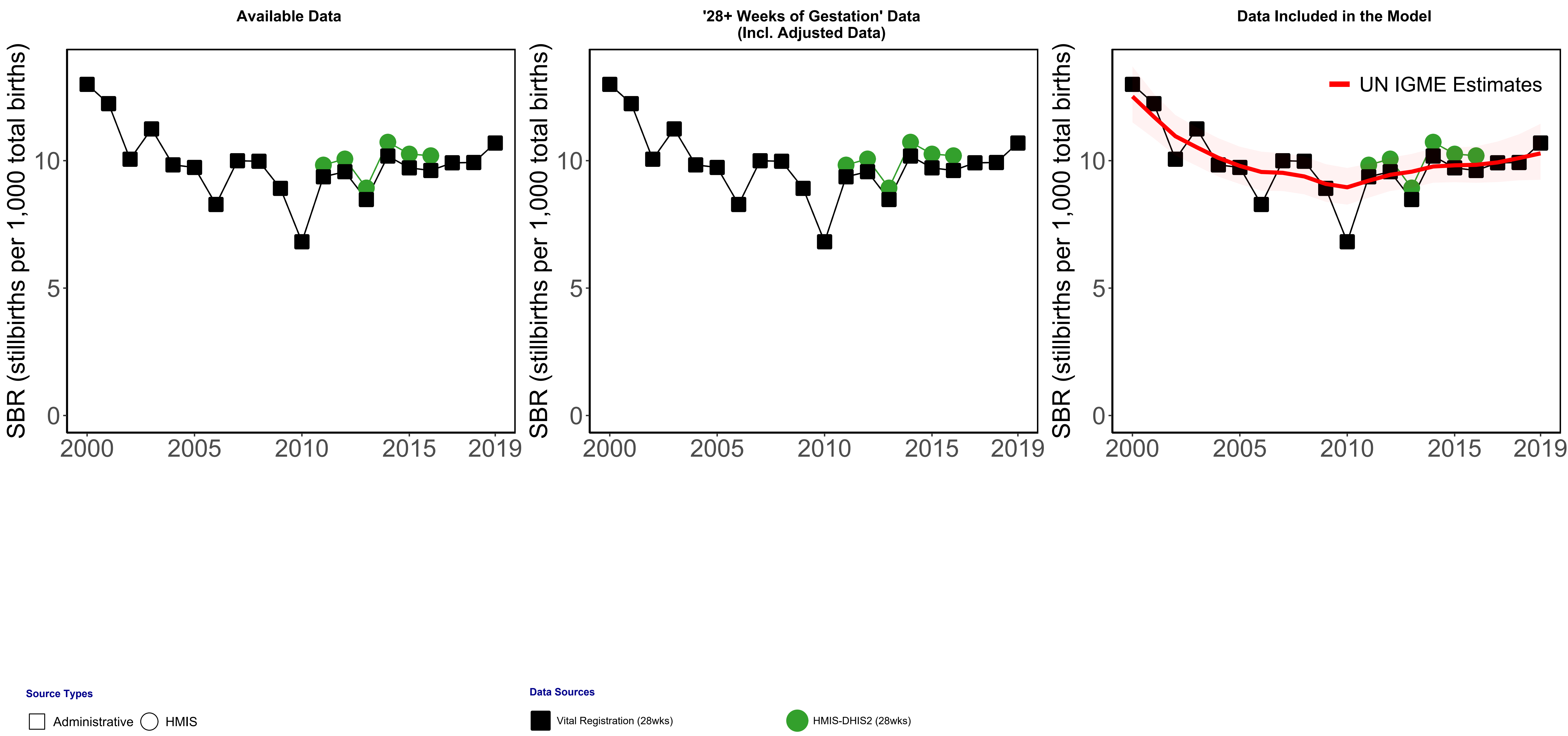

Available Data
'28+ Weeks of Gestation' Data
(Incl. Adjusted Data)
Data Included in the Model
SBR (stillbirths per 1,000 total births)
0
5
10
2000
2005
2010
2015
2019
UN IGME Estimates
Source Types
Administrative
HMIS
Data Sources
Vital Registration (28wks)
HMIS-DHIS2 (28wks)

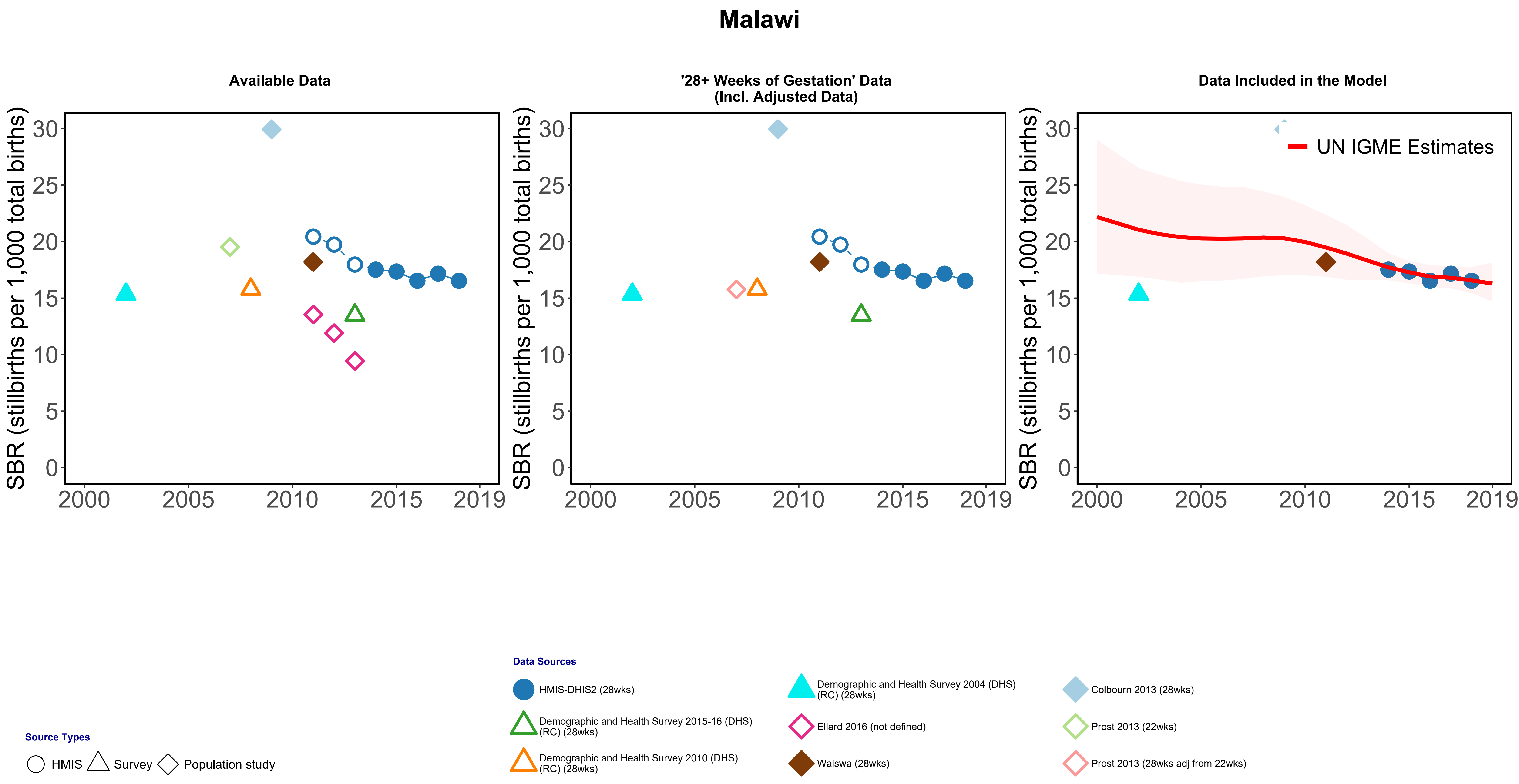
Malawi
Available Data
'28+ Weeks of Gestation' Data
(Incl. Adjusted Data)
Data Included in the Model
SBR (stillbirths per 1,000 total births)
UN IGME Estimates
Source Types
HMIS
Survey
Population study
Data Sources
HMIS-DHIS2 (28wks)
Demographic and Health Survey 2015-16 (DHS) (RC) (28wks)
Demographic and Health Survey 2010 (DHS) (RC) (28wks)
Demographic and Health Survey 2004 (DHS) (RC) (28wks)
Ellard 2016 (not defined)
Waiswa (28wks)
Colbourn 2013 (28wks)
Prost 2013 (22wks)
Prost 2013 (28wks adj from 22wks)

Malaysia

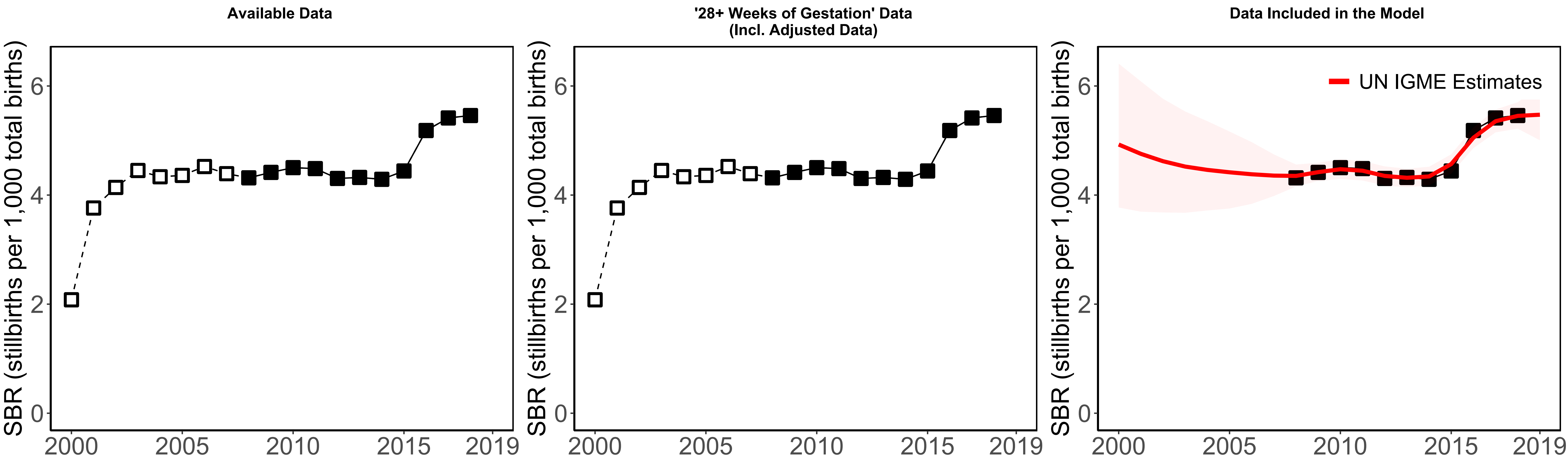

Available Data
'28+ Weeks of Gestation' Data
(Incl. Adjusted Data)
Data Included in the Model
UN IGME Estimates
SBR (stillbirths per 1,000 total births)
0
2
4
6
2000
2005
2010
2015
2019

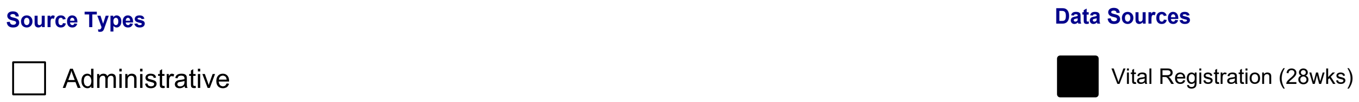

Source Types
Administrative
Data Sources
Vital Registration (28wks)

Namibia

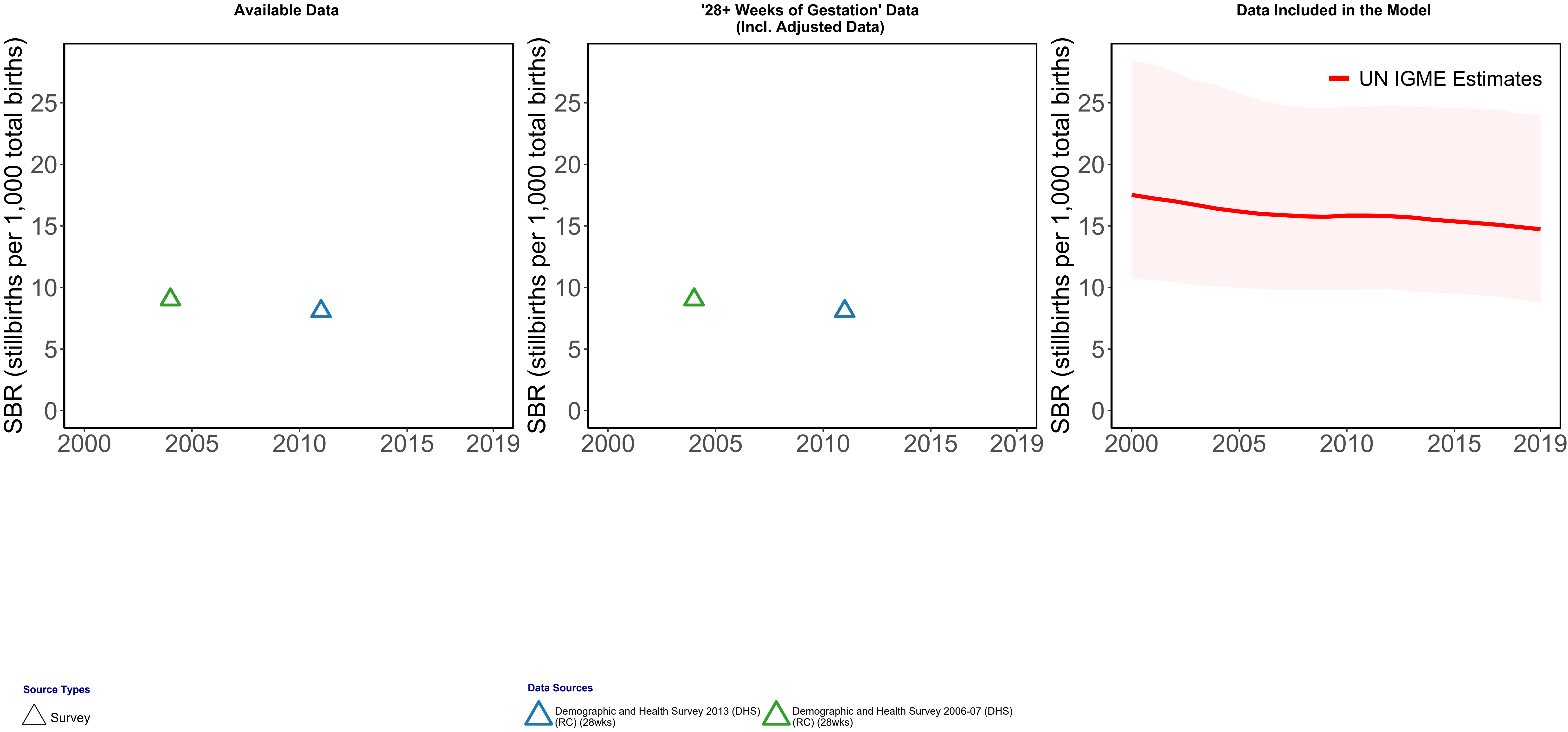

Available Data
'28+ Weeks of Gestation' Data
(Incl. Adjusted Data)
Data Included in the Model
SBR (stillbirths per 1,000 total births)
0
5
10
15
20
25
2000
2005
2010
2015
2019
UN IGME Estimates
Source Types
Survey
Data Sources
Demographic and Health Survey 2013 (DHS) (RC) (28wks)
Demographic and Health Survey 2006-07 (DHS) (RC) (28wks)

Niger

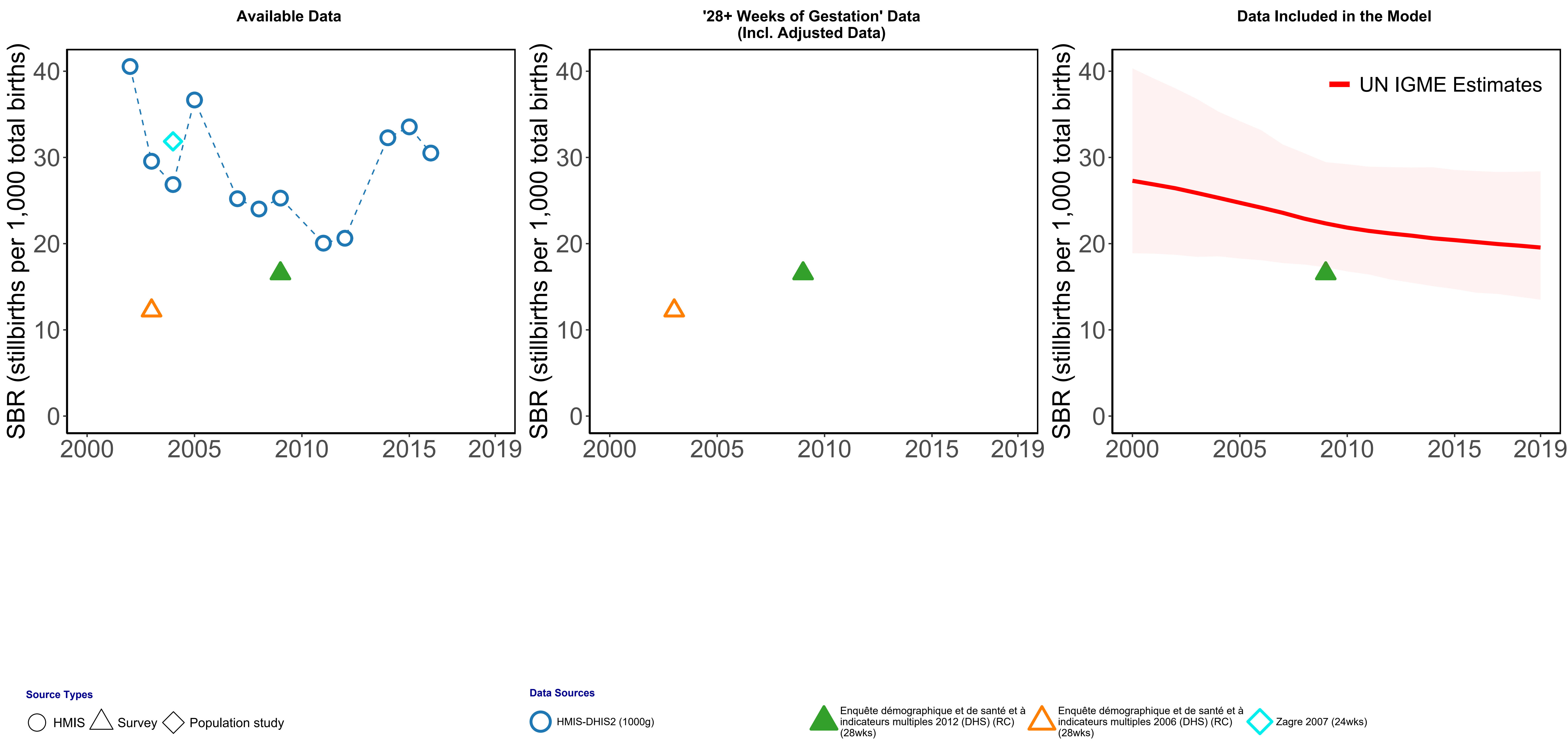
Available Data
'28+ Weeks of Gestation' Data
(Incl. Adjusted Data)
Data Included in the Model
SBR (stillbirths per 1,000 total births)
0
10
20
30
40
2000
2005
2010
2015
2019
UN IGME Estimates
Source Types
HMIS
Survey
Population study
Data Sources
HMIS-DHIS2 (1000g)
Enquête démographique et de santé et à indicateurs multiples 2012 (DHS) (RC) (28wks)
Enquête démographique et de santé et à indicateurs multiples 2006 (DHS) (RC) (28wks)
Zagre 2007 (24wks)

Nigeria

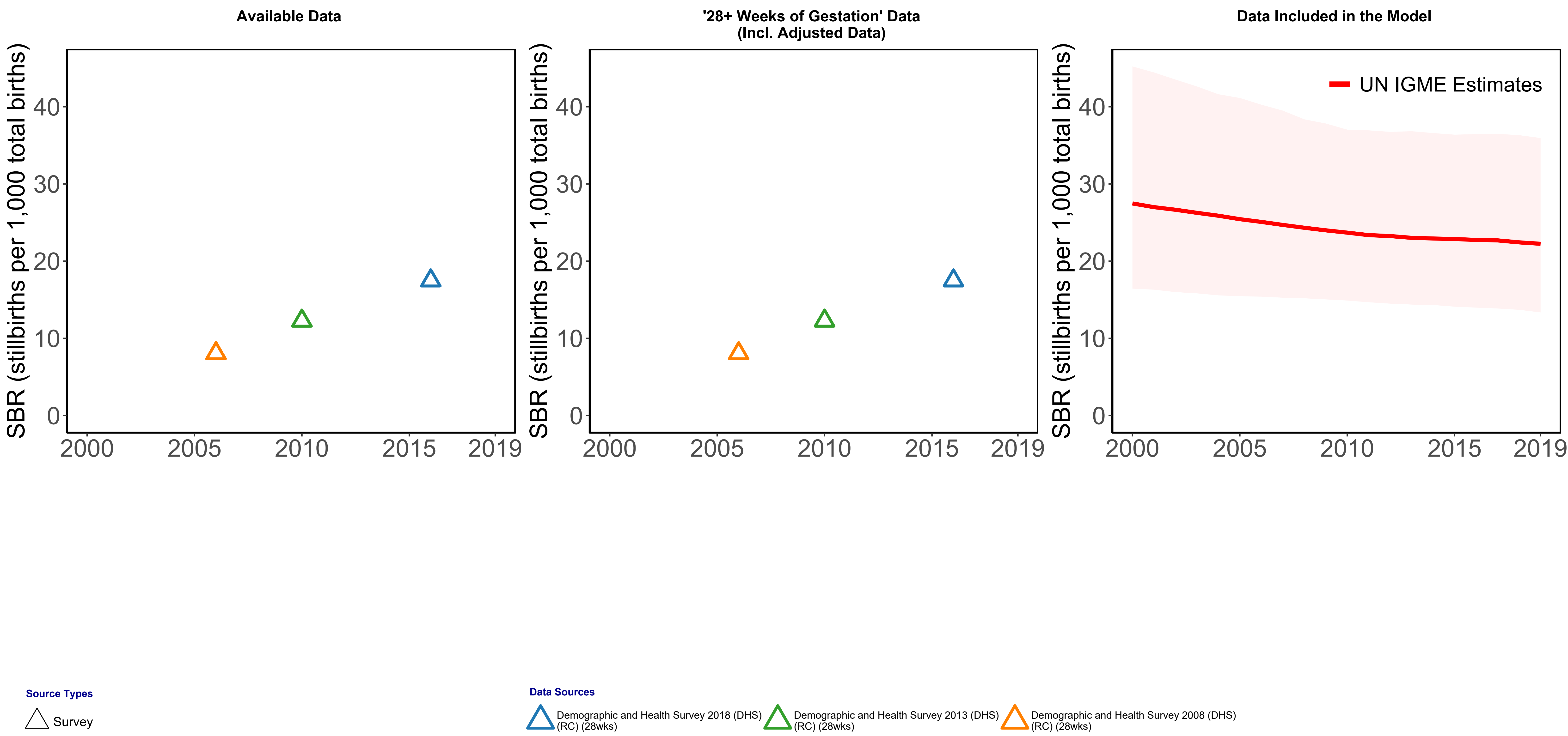

Available Data
'28+ Weeks of Gestation' Data
(Incl. Adjusted Data)
Data Included in the Model
UN IGME Estimates
SBR (stillbirths per 1,000 total births)
0
10
20
30
40
2000
2005
2010
2015
2019
Source Types
Survey
Data Sources
Demographic and Health Survey 2018 (DHS) (RC) (28wks)
Demographic and Health Survey 2013 (DHS) (RC) (28wks)
Demographic and Health Survey 2008 (DHS) (RC) (28wks)

Nicaragua

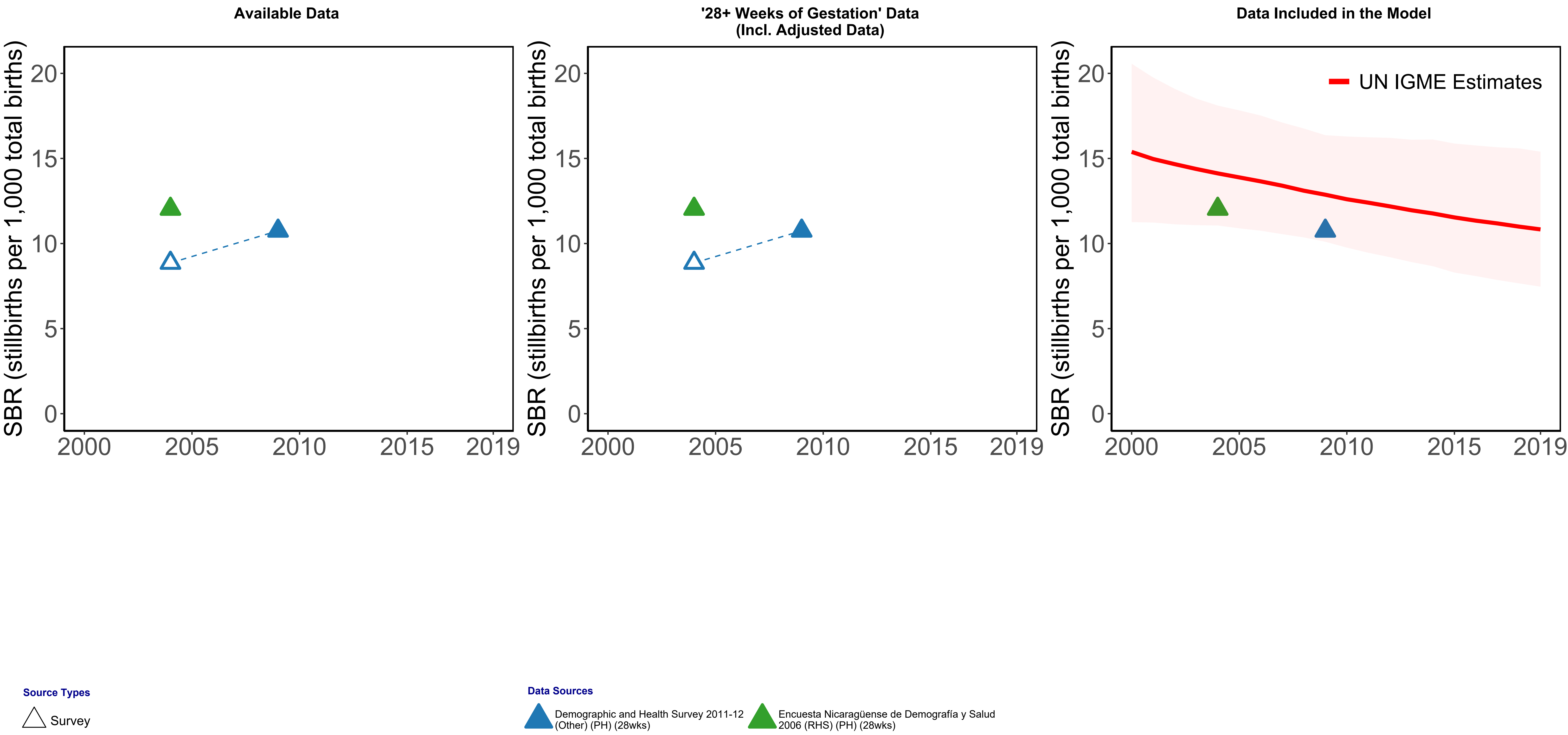

Available Data
'28+ Weeks of Gestation' Data
(Incl. Adjusted Data)
Data Included in the Model
SBR (stillbirths per 1,000 total births)
0
5
10
15
20
2000
2005
2010
2015
2019
UN IGME Estimates
Source Types
Survey
Data Sources
Demographic and Health Survey 2011-12 (Other) (PH) (28wks)
Encuesta Nicaragüense de Demografía y Salud 2006 (RHS) (PH) (28wks)

Niue

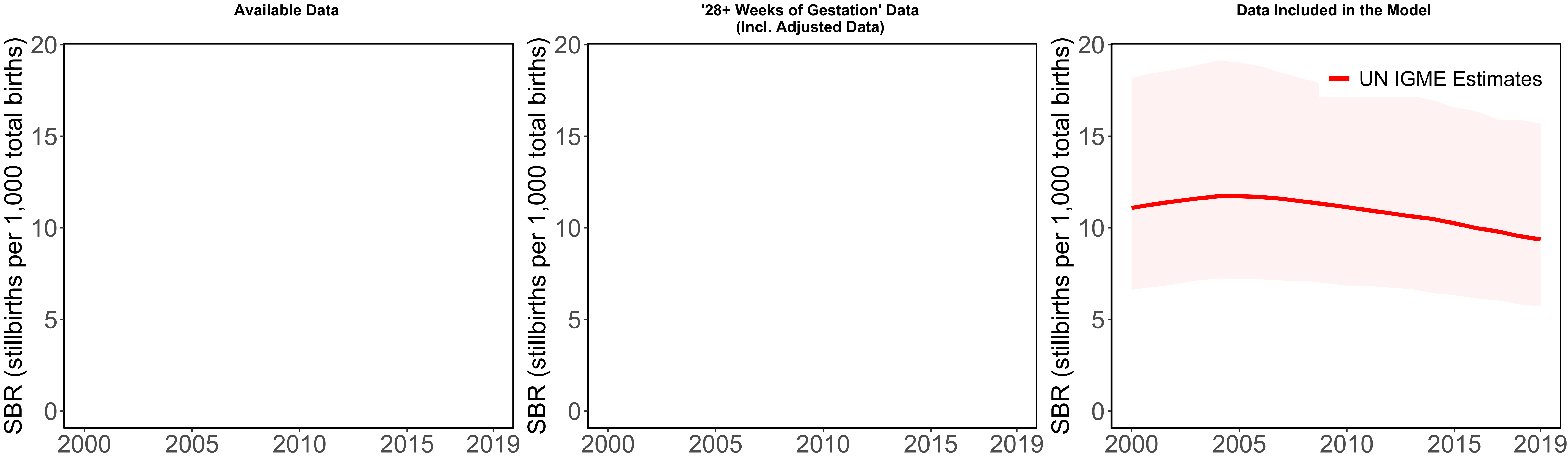

Available Data
'28+ Weeks of Gestation' Data
(Incl. Adjusted Data)
Data Included in the Model
SBR (stillbirths per 1,000 total births)
0
5
10
15
20
2000
2005
2010
2015
2019
UN IGME Estimates

Netherlands

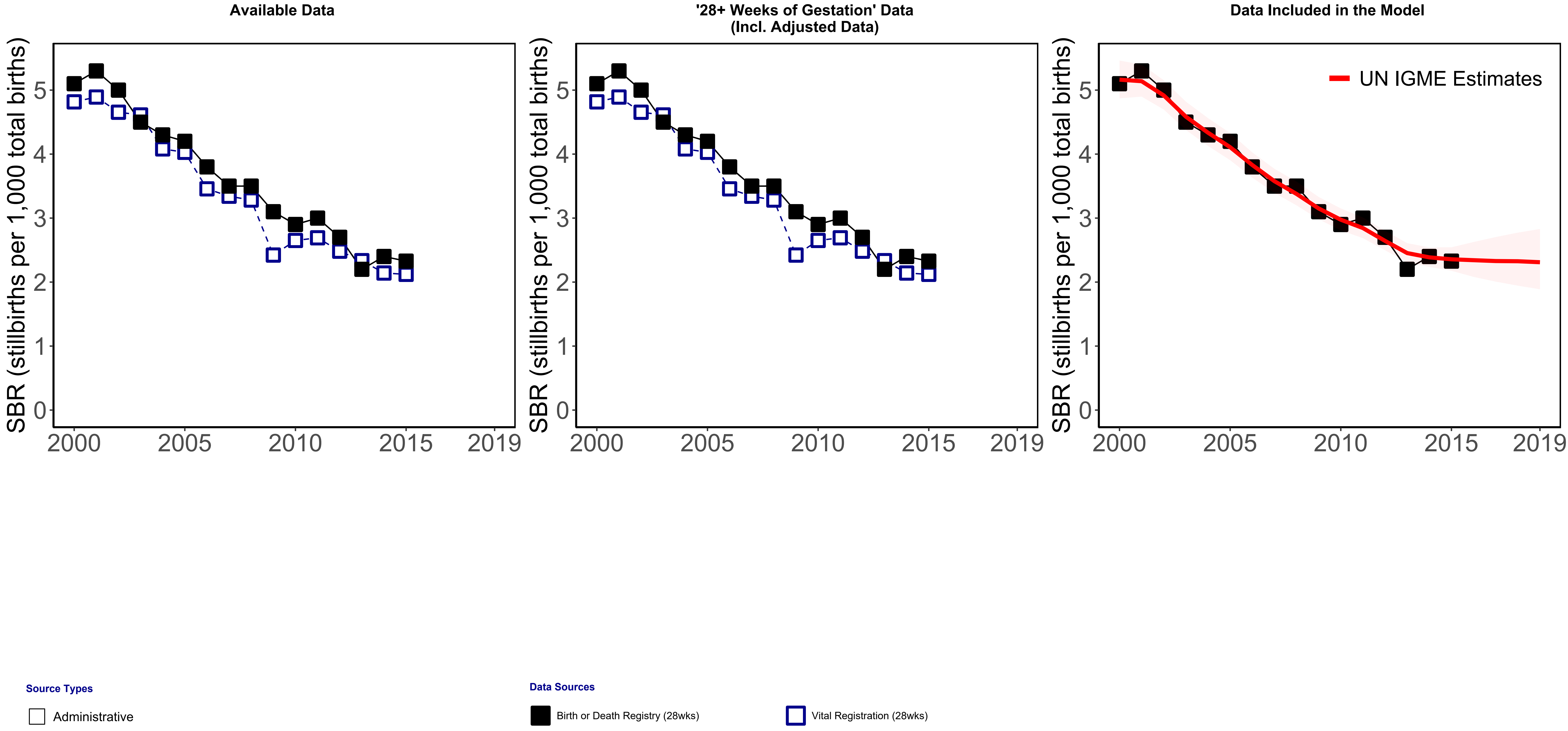

Norway

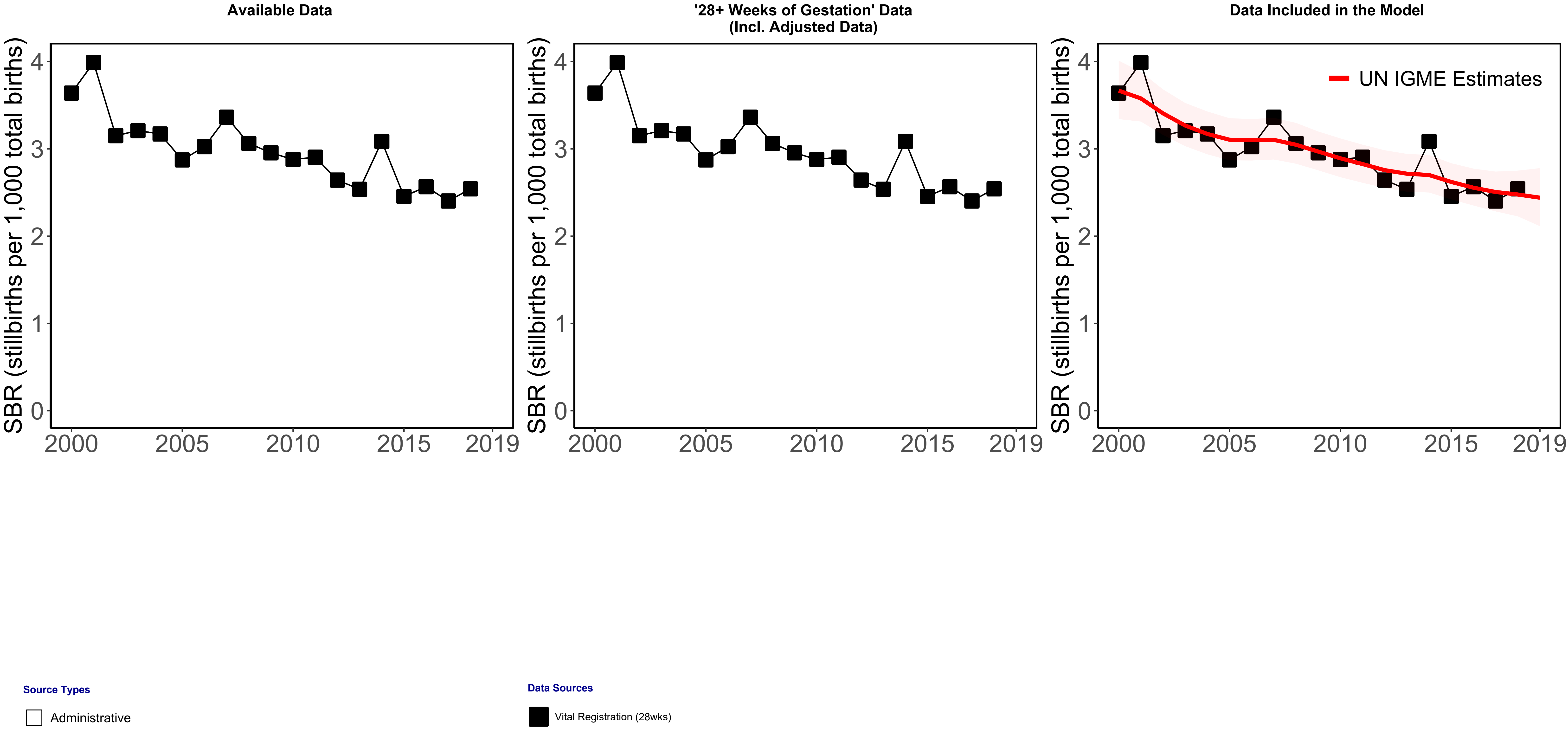

Available Data
'28+ Weeks of Gestation' Data
(Incl. Adjusted Data)
Data Included in the Model
SBR (stillbirths per 1,000 total births)
0
1
2
3
4
2000
2005
2010
2015
2019
UN IGME Estimates
Source Types
Administrative
Data Sources
Vital Registration (28wks)

Nepal

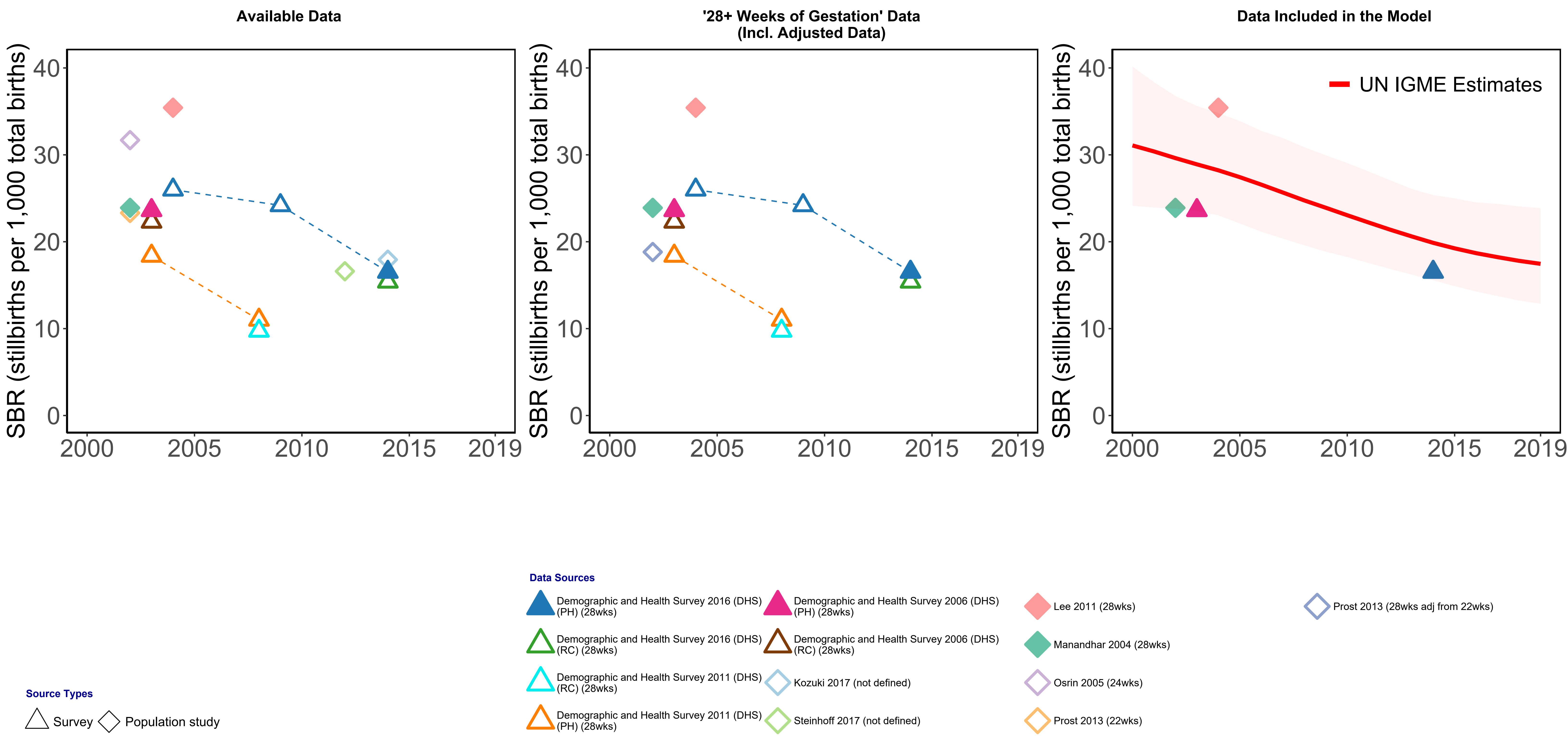
Available Data
'28+ Weeks of Gestation' Data
(Incl. Adjusted Data)
Data Included in the Model
SBR (stillbirths per 1,000 total births)
0
10
20
30
40
2000
2005
2010
2015
2019
UN IGME Estimates
Data Sources
Demographic and Health Survey 2016 (DHS) (PH) (28wks)
Demographic and Health Survey 2016 (DHS) (RC) (28wks)
Demographic and Health Survey 2011 (DHS) (RC) (28wks)
Demographic and Health Survey 2011 (DHS) (PH) (28wks)
Demographic and Health Survey 2006 (DHS) (PH) (28wks)
Demographic and Health Survey 2006 (DHS) (RC) (28wks)
Kozuki 2017 (not defined)
Steinhoff 2017 (not defined)
Lee 2011 (28wks)
Manandhar 2004 (28wks)
Osrin 2005 (24wks)
Prost 2013 (22wks)
Prost 2013 (28wks adj from 22wks)
Source Types
Survey
Population study

Nauru

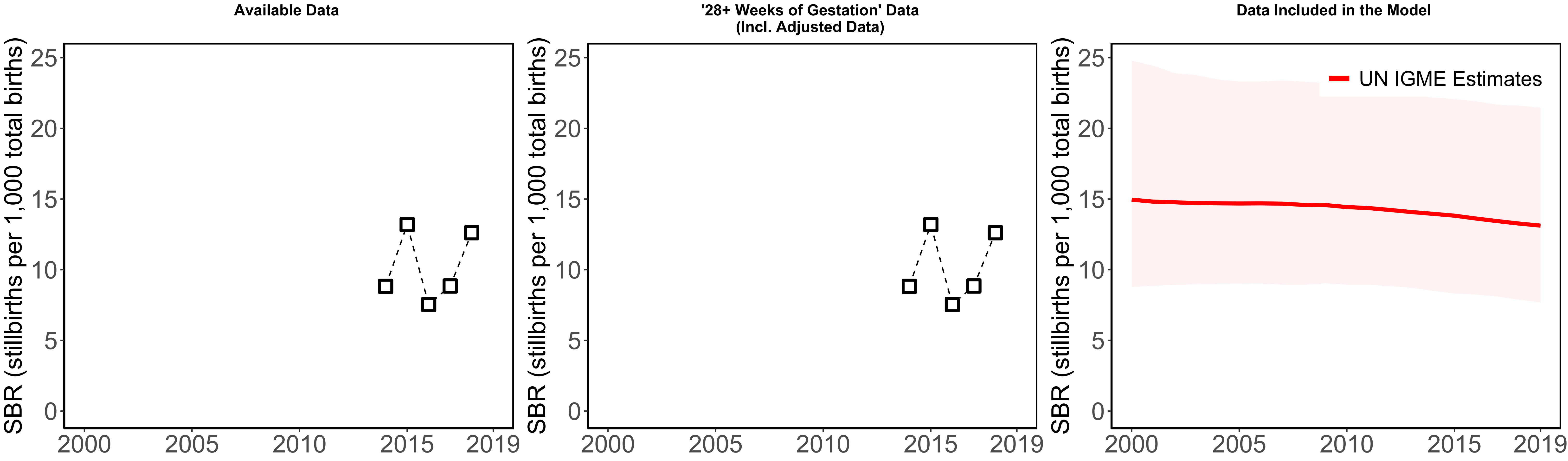

Source Types

Administrative

Data Sources

Birth or Death Registry (28wks)

New Zealand

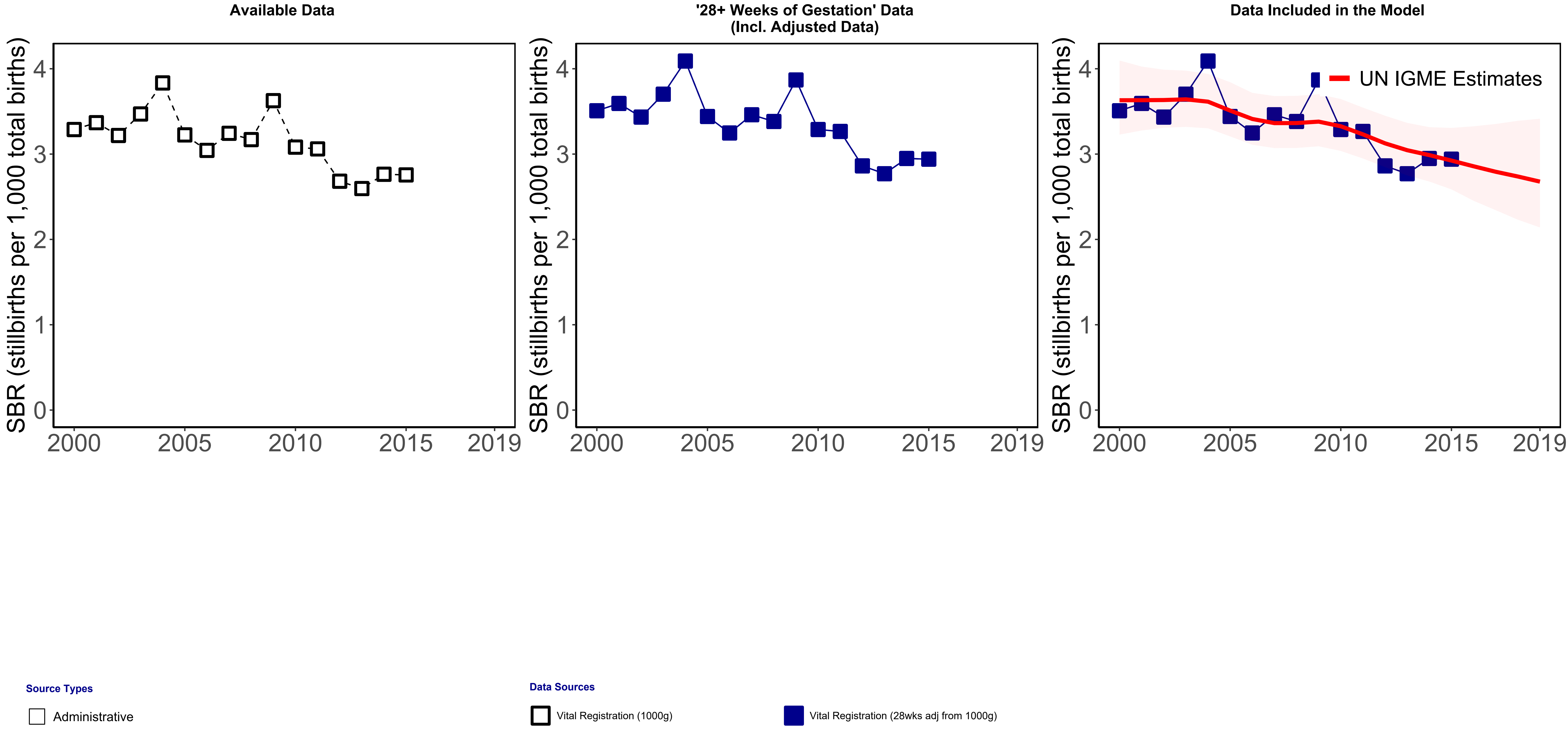
Available Data
'28+ Weeks of Gestation' Data
(Incl. Adjusted Data)
Data Included in the Model
SBR (stillbirths per 1,000 total births)
0
1
2
3
4
2000
2005
2010
2015
2019
UN IGME Estimates
Source Types
Administrative
Data Sources
Vital Registration (1000g)
Vital Registration (28wks adj from 1000g)

Oman

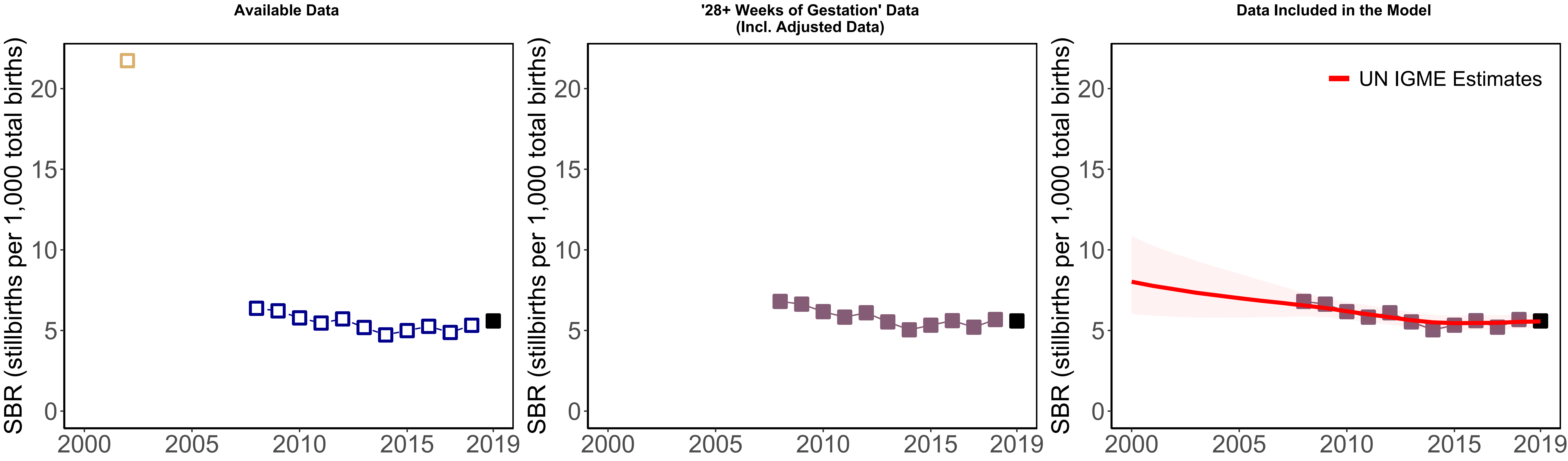

Available Data
'28+ Weeks of Gestation' Data
(Incl. Adjusted Data)
Data Included in the Model
UN IGME Estimates
SBR (stillbirths per 1,000 total births)
0
5
10
15
20
2000
2005
2010
2015
2019

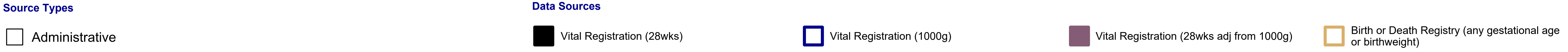

Source Types
Administrative
Data Sources
Vital Registration (28wks)
Vital Registration (1000g)
Vital Registration (28wks adj from 1000g)
Birth or Death Registry (any gestational age or birthweight)

Pakistan

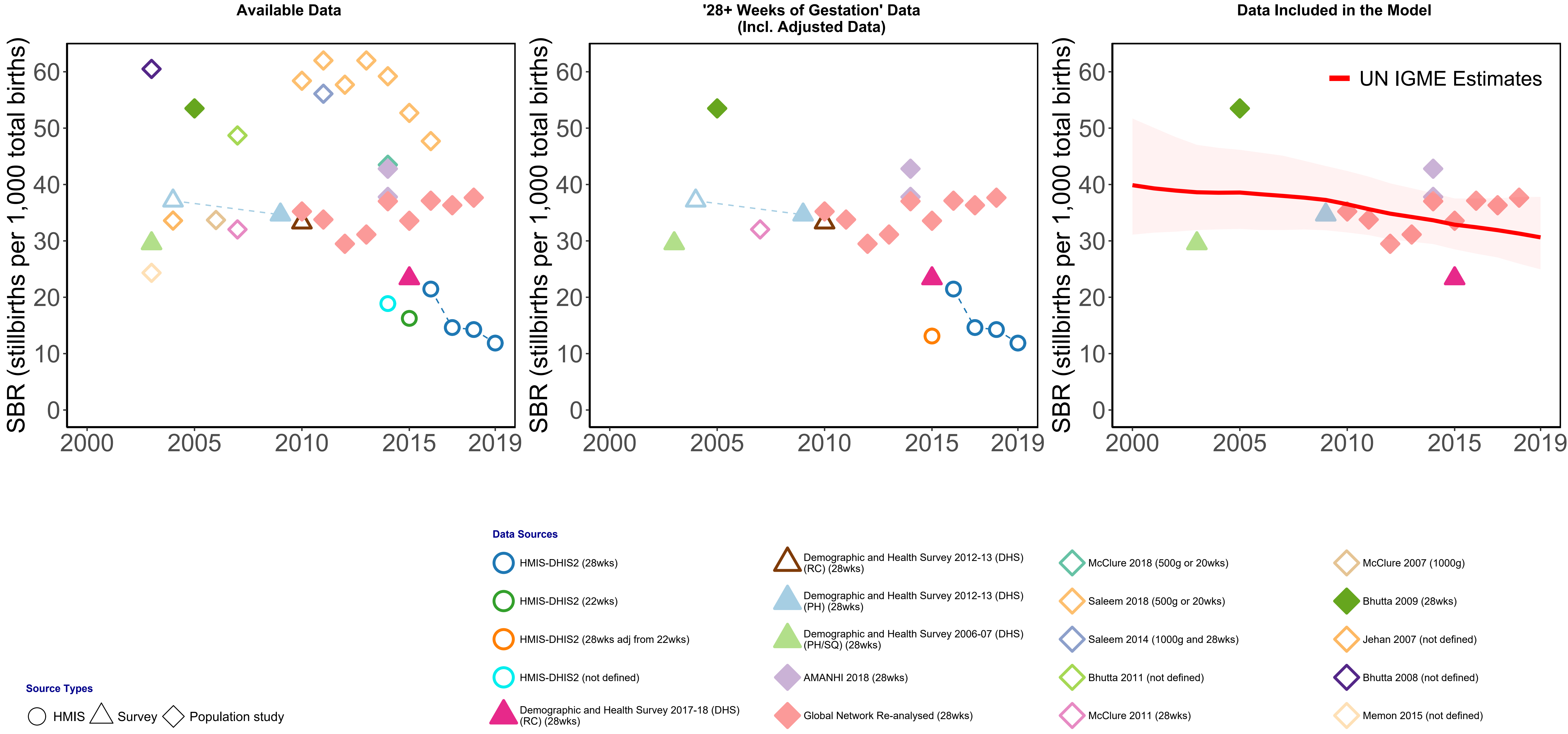
Available Data
'28+ Weeks of Gestation' Data
(Incl. Adjusted Data)
Data Included in the Model
SBR (stillbirths per 1,000 total births)
0
10
20
30
40
50
60
2000
2005
2010
2015
2019
UN IGME Estimates
Data Sources
HMIS-DHIS2 (28wks)
HMIS-DHIS2 (22wks)
HMIS-DHIS2 (28wks adj from 22wks)
HMIS-DHIS2 (not defined)
Demographic and Health Survey 2017-18 (DHS) (RC) (28wks)
Demographic and Health Survey 2012-13 (DHS) (RC) (28wks)
Demographic and Health Survey 2012-13 (DHS) (PH) (28wks)
Demographic and Health Survey 2006-07 (DHS) (PH/SQ) (28wks)
AMANHI 2018 (28wks)
Global Network Re-analysed (28wks)
McClure 2018 (500g or 20wks)
Saleem 2018 (500g or 20wks)
Saleem 2014 (1000g and 28wks)
Bhutta 2011 (not defined)
McClure 2011 (28wks)
McClure 2007 (1000g)
Bhutta 2009 (28wks)
Jehan 2007 (not defined)
Bhutta 2008 (not defined)
Memon 2015 (not defined)
Source Types
HMIS
Survey
Population study

Panama

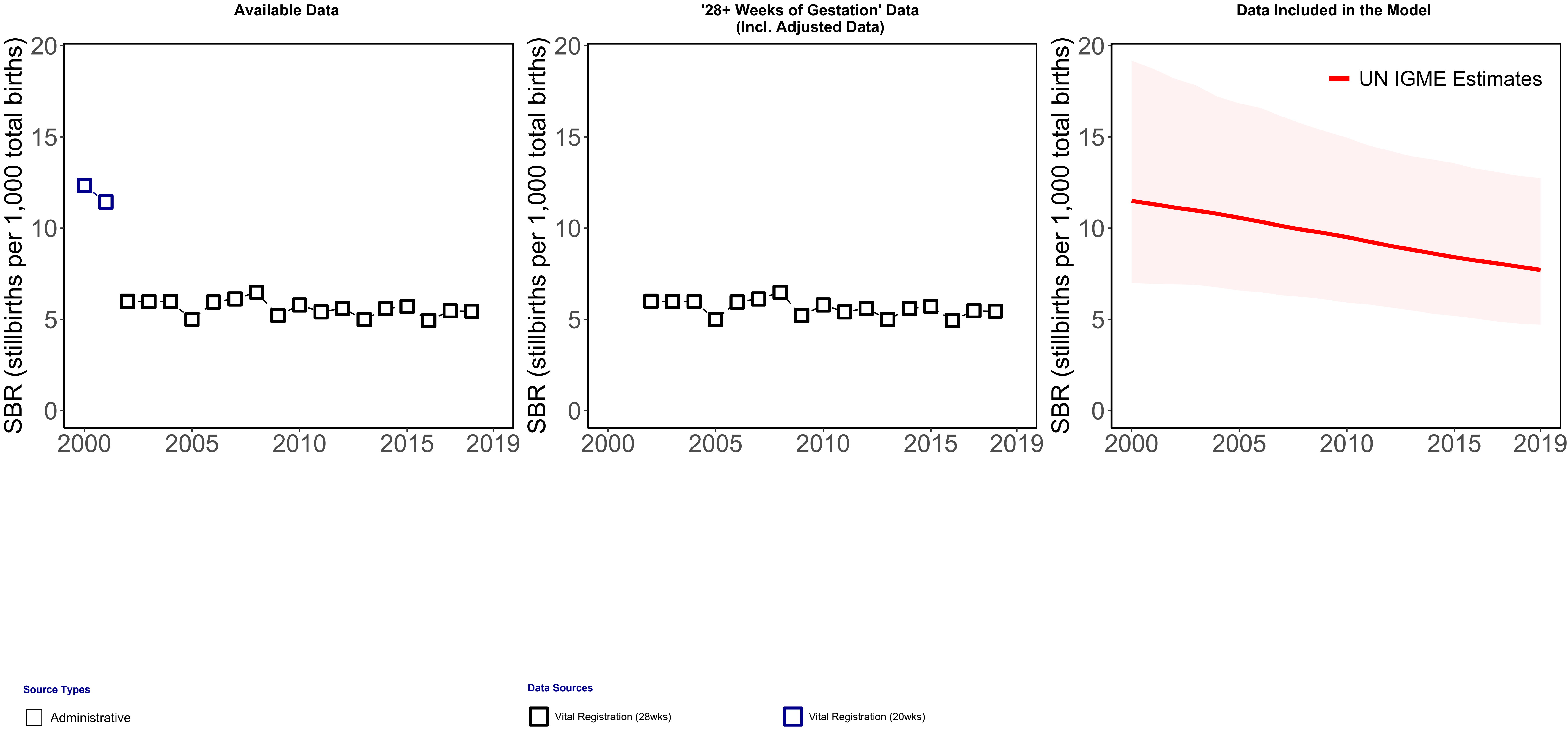
Available Data
'28+ Weeks of Gestation' Data
(Incl. Adjusted Data)
Data Included in the Model
SBR (stillbirths per 1,000 total births)
0
5
10
15
20
2000
2005
2010
2015
2019
UN IGME Estimates
Source Types
Administrative
Data Sources
Vital Registration (28wks)
Vital Registration (20wks)

Peru

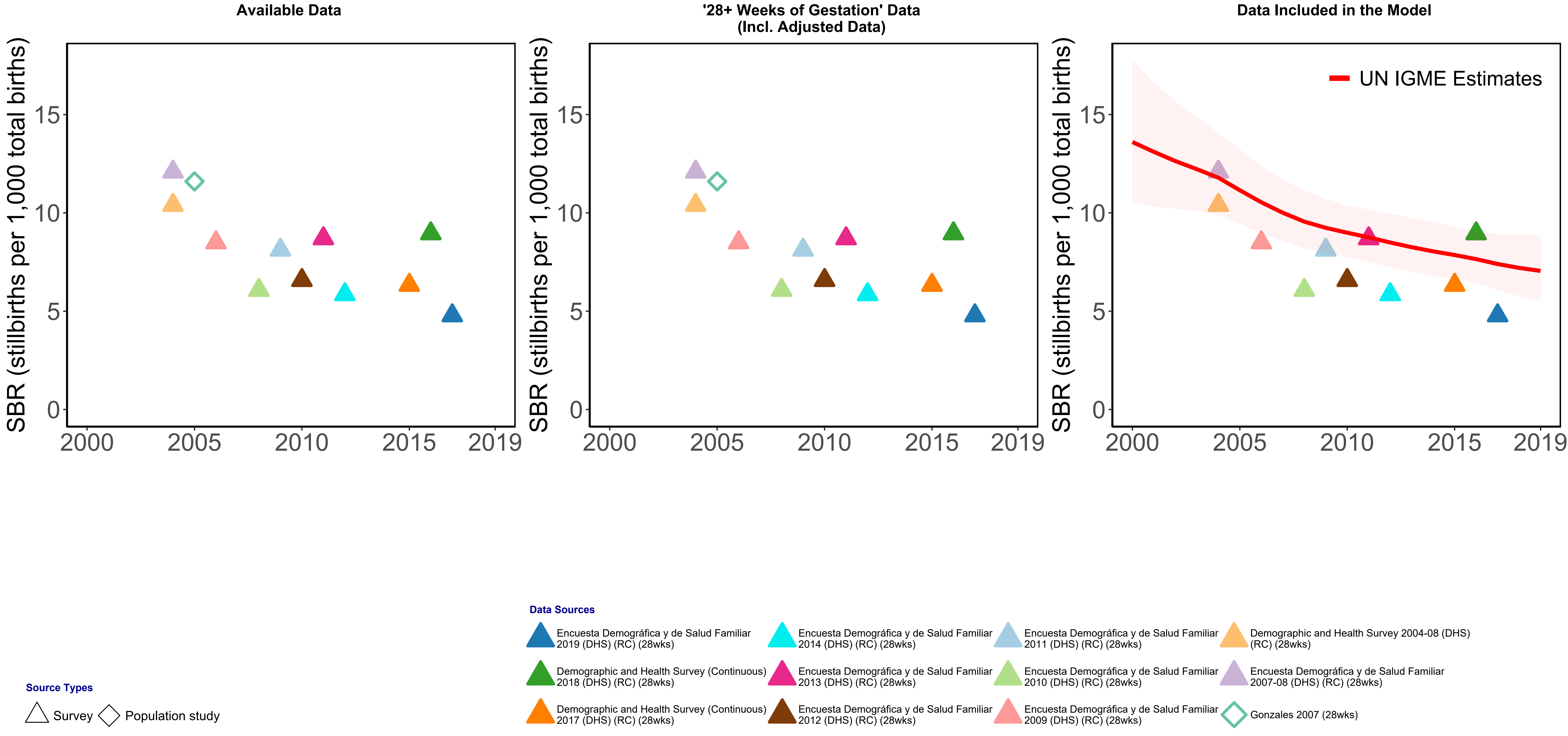
Available Data
'28+ Weeks of Gestation' Data
(Incl. Adjusted Data)
Data Included in the Model
SBR (stillbirths per 1,000 total births)
0
5
10
15
2000
2005
2010
2015
2019
UN IGME Estimates
Source Types
Survey
Population study
Data Sources
Encuesta Demográfica y de Salud Familiar 2019 (DHS) (RC) (28wks)
Encuesta Demográfica y de Salud Familiar 2014 (DHS) (RC) (28wks)
Encuesta Demográfica y de Salud Familiar 2011 (DHS) (RC) (28wks)
Demographic and Health Survey 2004-08 (DHS) (RC) (28wks)
Demographic and Health Survey (Continuous) 2018 (DHS) (RC) (28wks)
Encuesta Demográfica y de Salud Familiar 2013 (DHS) (RC) (28wks)
Encuesta Demográfica y de Salud Familiar 2010 (DHS) (RC) (28wks)
Encuesta Demográfica y de Salud Familiar 2007-08 (DHS) (RC) (28wks)
Demographic and Health Survey (Continuous) 2017 (DHS) (RC) (28wks)
Encuesta Demográfica y de Salud Familiar 2012 (DHS) (RC) (28wks)
Encuesta Demográfica y de Salud Familiar 2009 (DHS) (RC) (28wks)
Gonzales 2007 (28wks)

Philippines

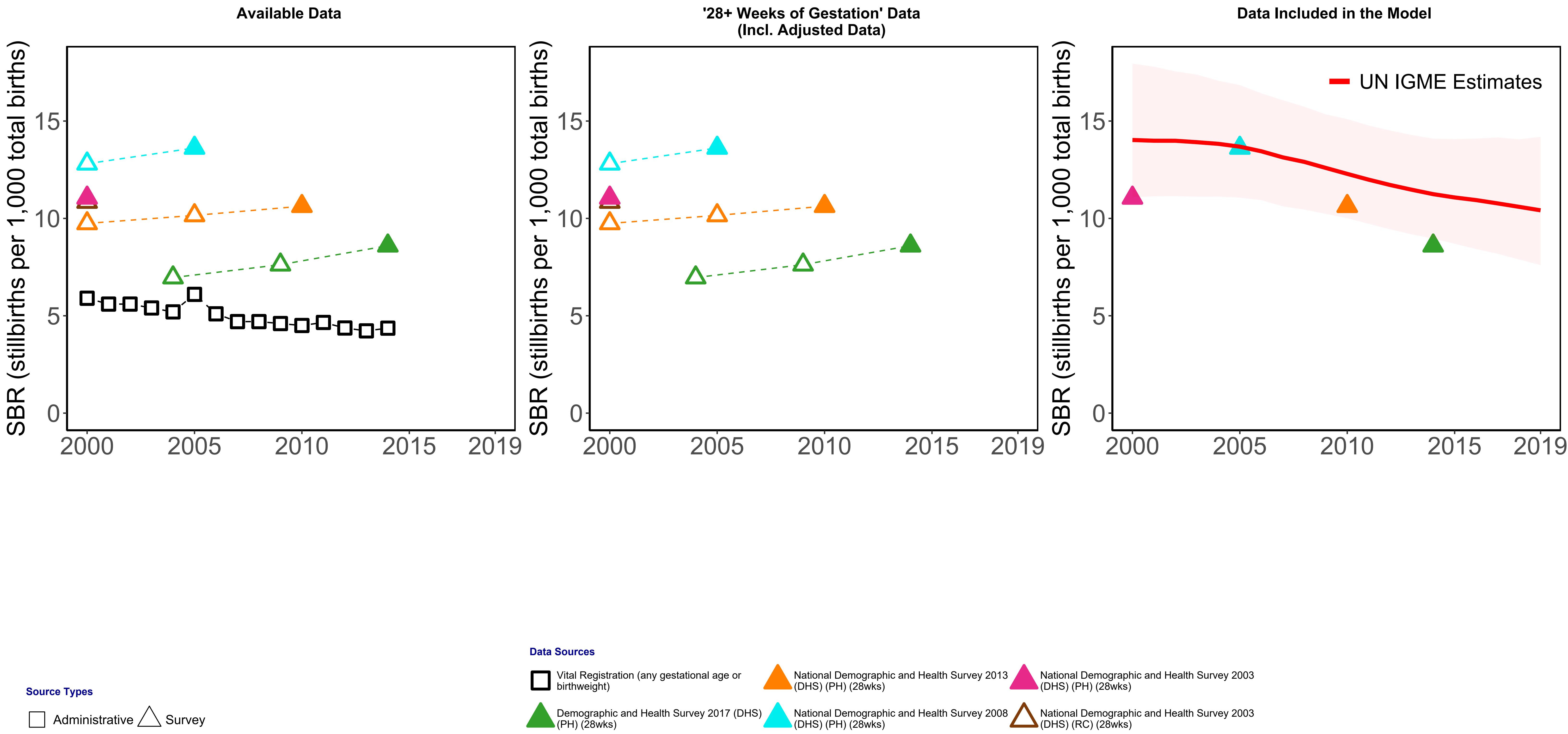

Palau

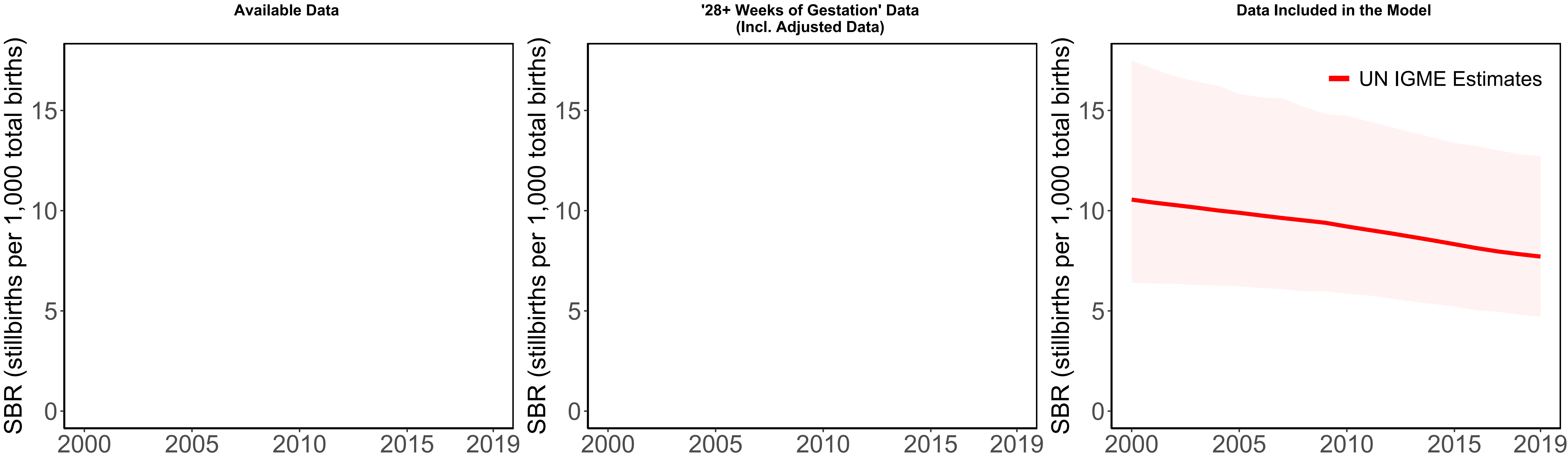

Available Data
'28+ Weeks of Gestation' Data
(Incl. Adjusted Data)
Data Included in the Model
SBR (stillbirths per 1,000 total births)
0
5
10
15
2000
2005
2010
2015
2019
UN IGME Estimates

Papua New Guinea

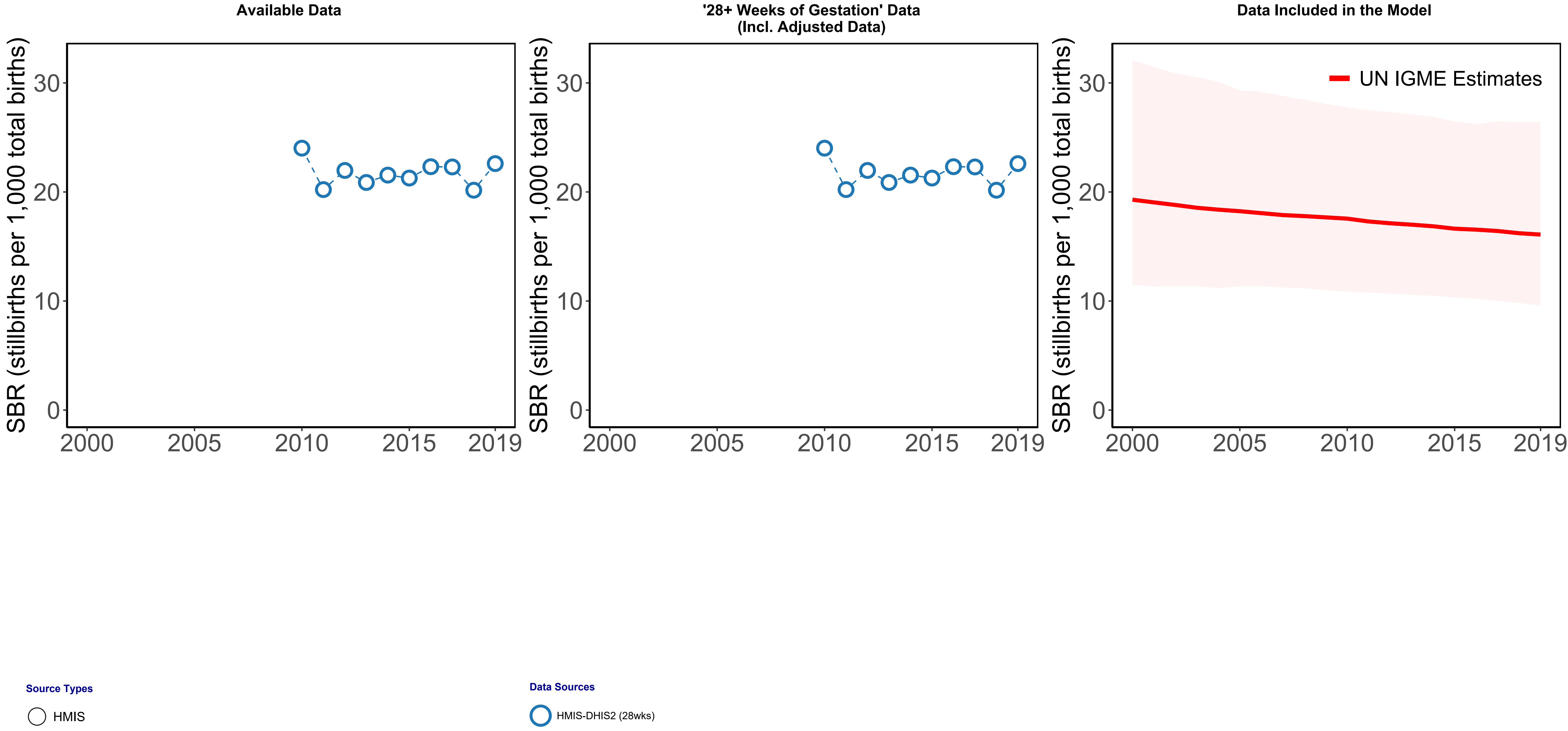

Poland

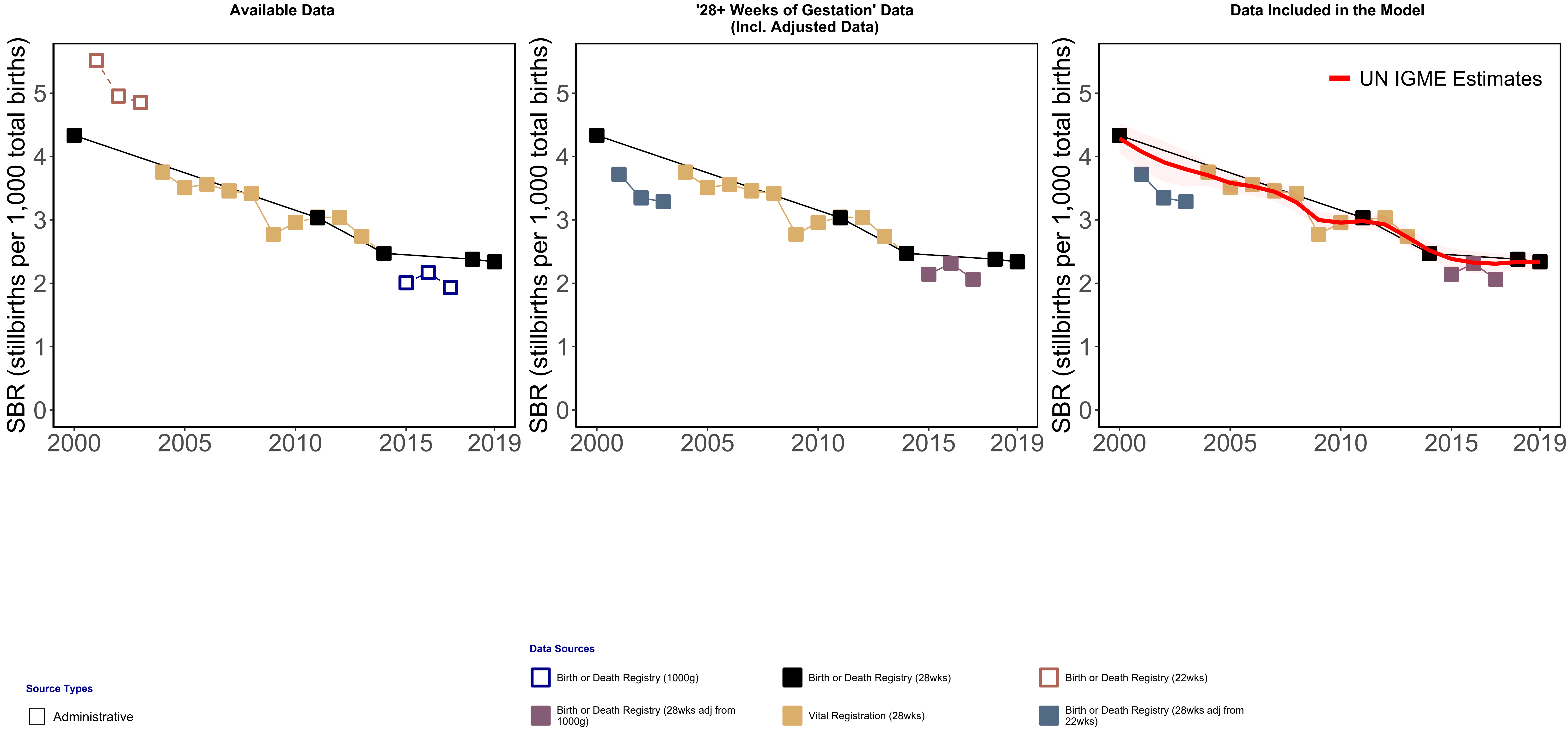
Available Data
'28+ Weeks of Gestation' Data
(Incl. Adjusted Data)
Data Included in the Model
UN IGME Estimates
SBR (stillbirths per 1,000 total births)
0
1
2
3
4
5
2000
2005
2010
2015
2019
Source Types
Administrative
Data Sources
Birth or Death Registry (1000g)
Birth or Death Registry (28wks)
Birth or Death Registry (22wks)
Birth or Death Registry (28wks adj from 1000g)
Vital Registration (28wks)
Birth or Death Registry (28wks adj from 22wks)

People's Republic of Korea

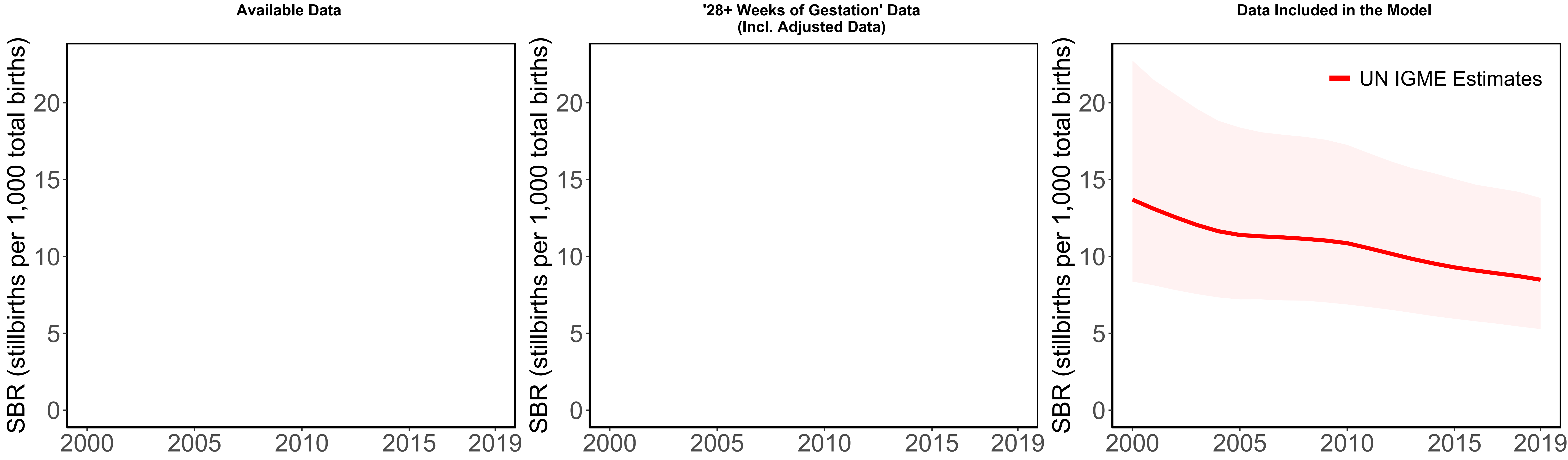

Portugal

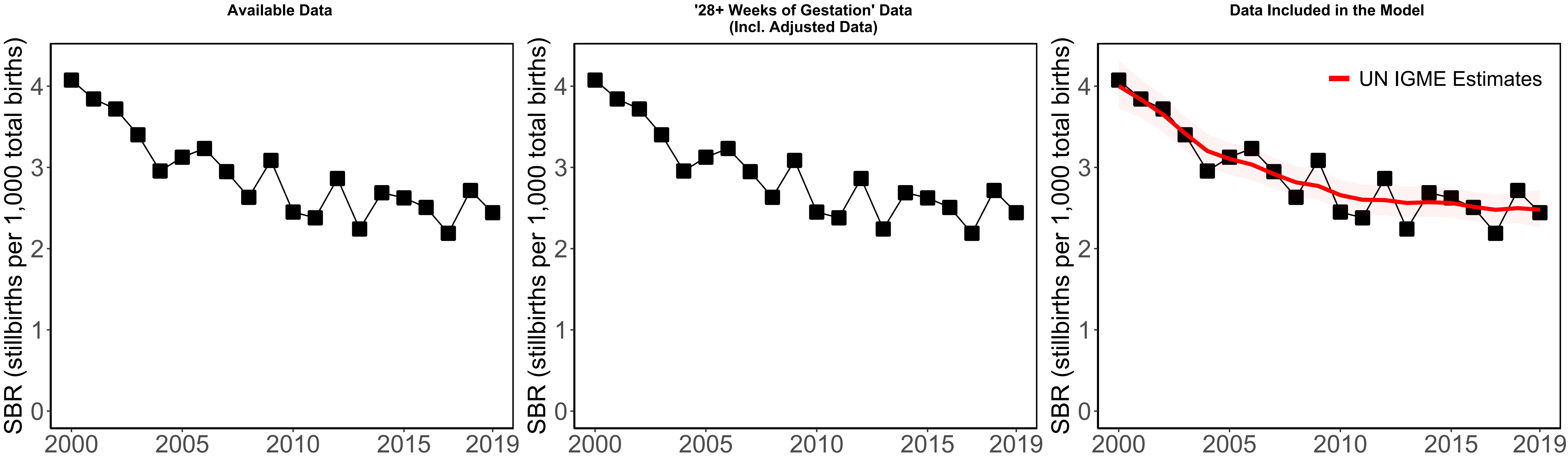

Source Types

Administrative

Data Sources

Vital Registration (28wks)

Paraguay

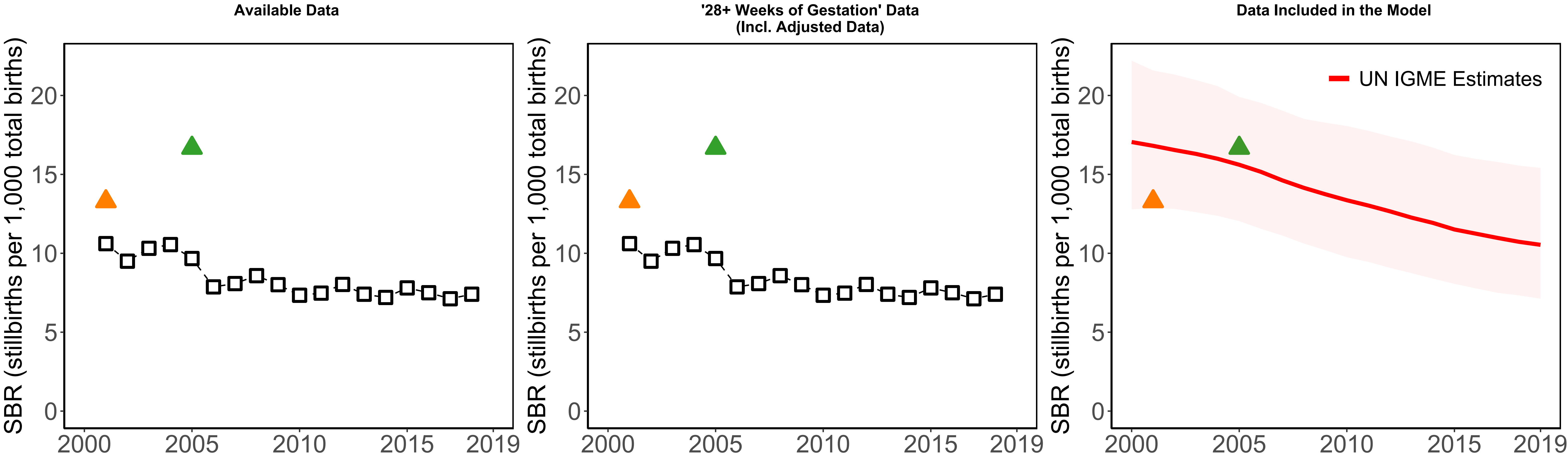

Source Types
Administrative
Survey

Data Sources
Vital Registration (28wks)
Encuesta Nacional de Demografía y Salud Sexual y Reproductiva 2008 (RHS) (PH) (28wks)
Encuesta Nacional de Demografía y Salud Sexual y Reproductiva 2004 (RHS) (PH) (28wks)

State of Palestine

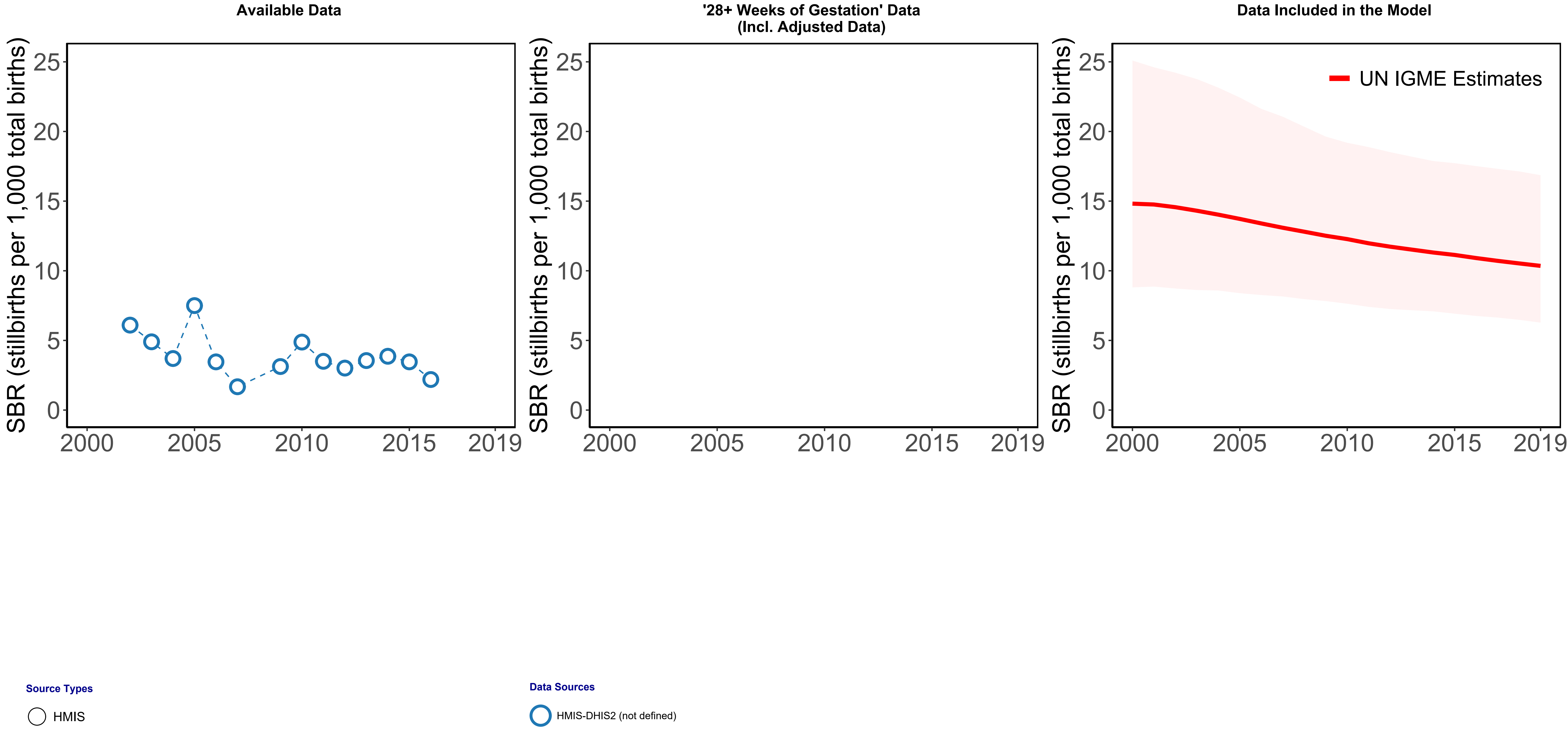

Qatar

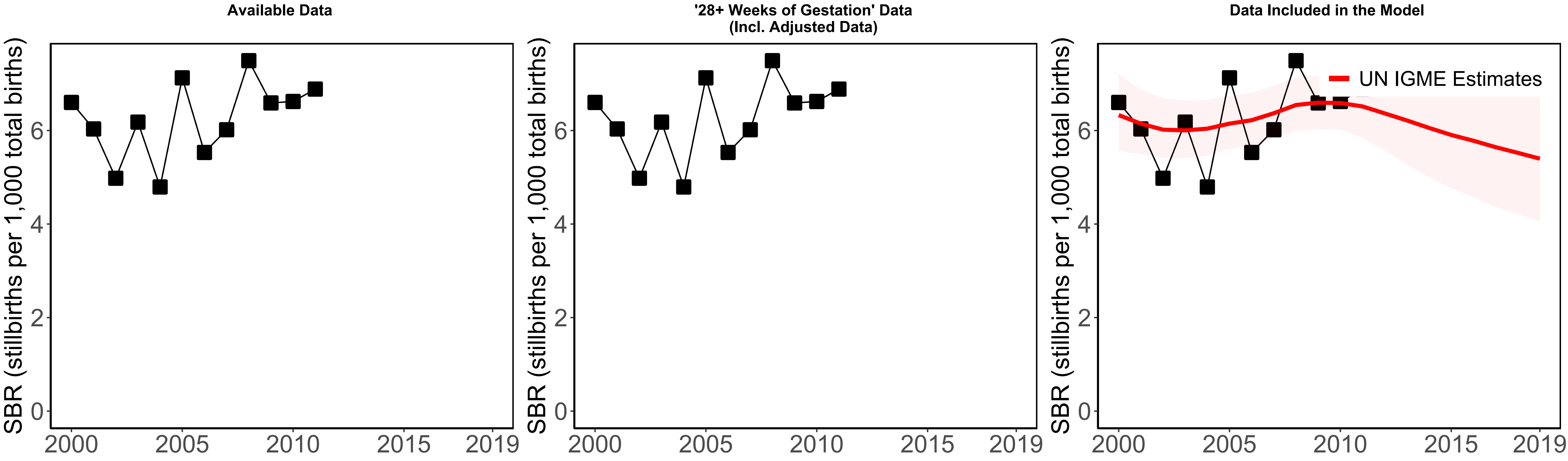

Source Types

Administrative

Data Sources

Vital Registration (28wks)

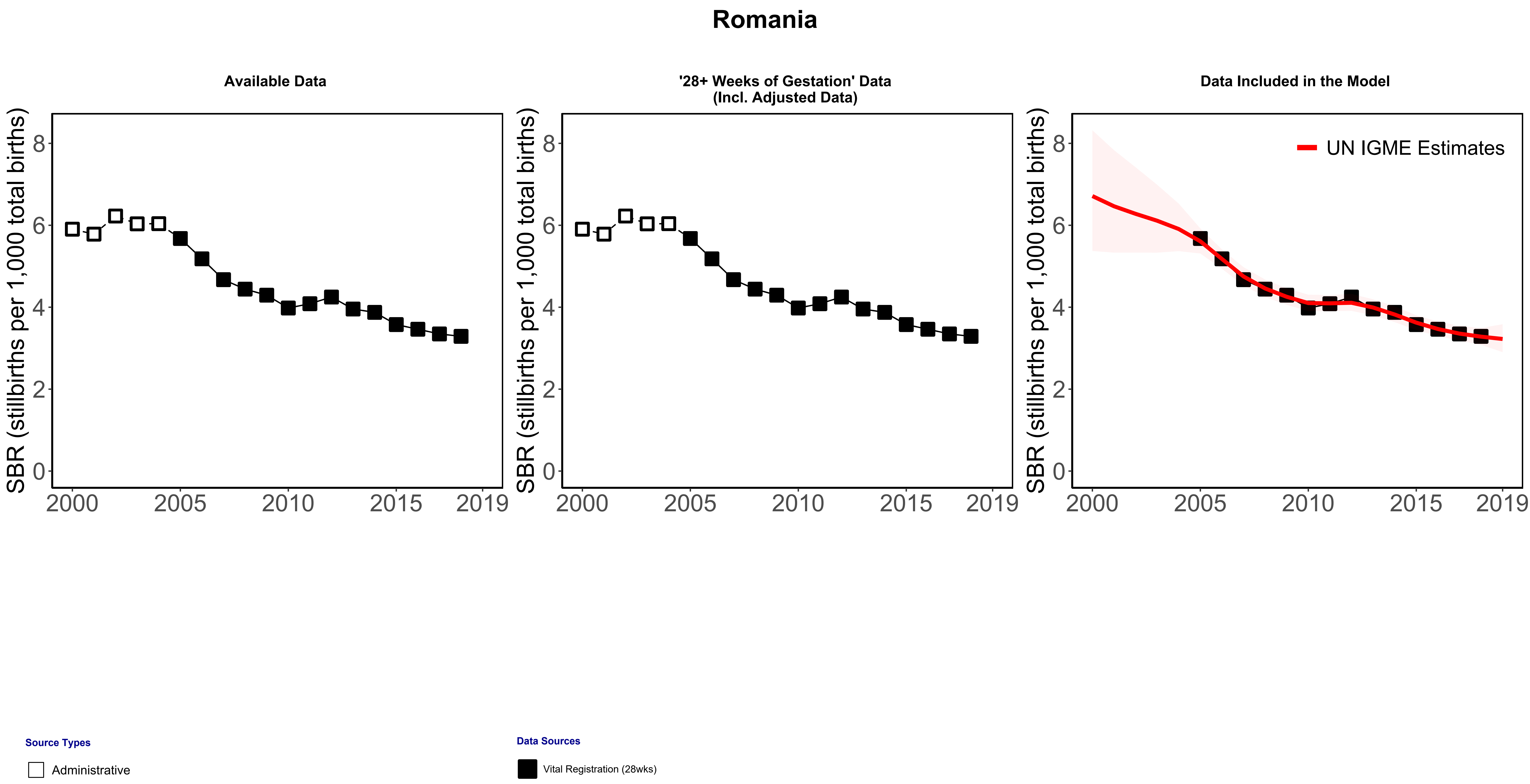
Romania
Available Data
'28+ Weeks of Gestation' Data
(Incl. Adjusted Data)
Data Included in the Model
UN IGME Estimates
SBR (stillbirths per 1,000 total births)
0
2
4
6
8
2000
2005
2010
2015
2019
Source Types
Administrative
Data Sources
Vital Registration (28wks)

Russian Federation

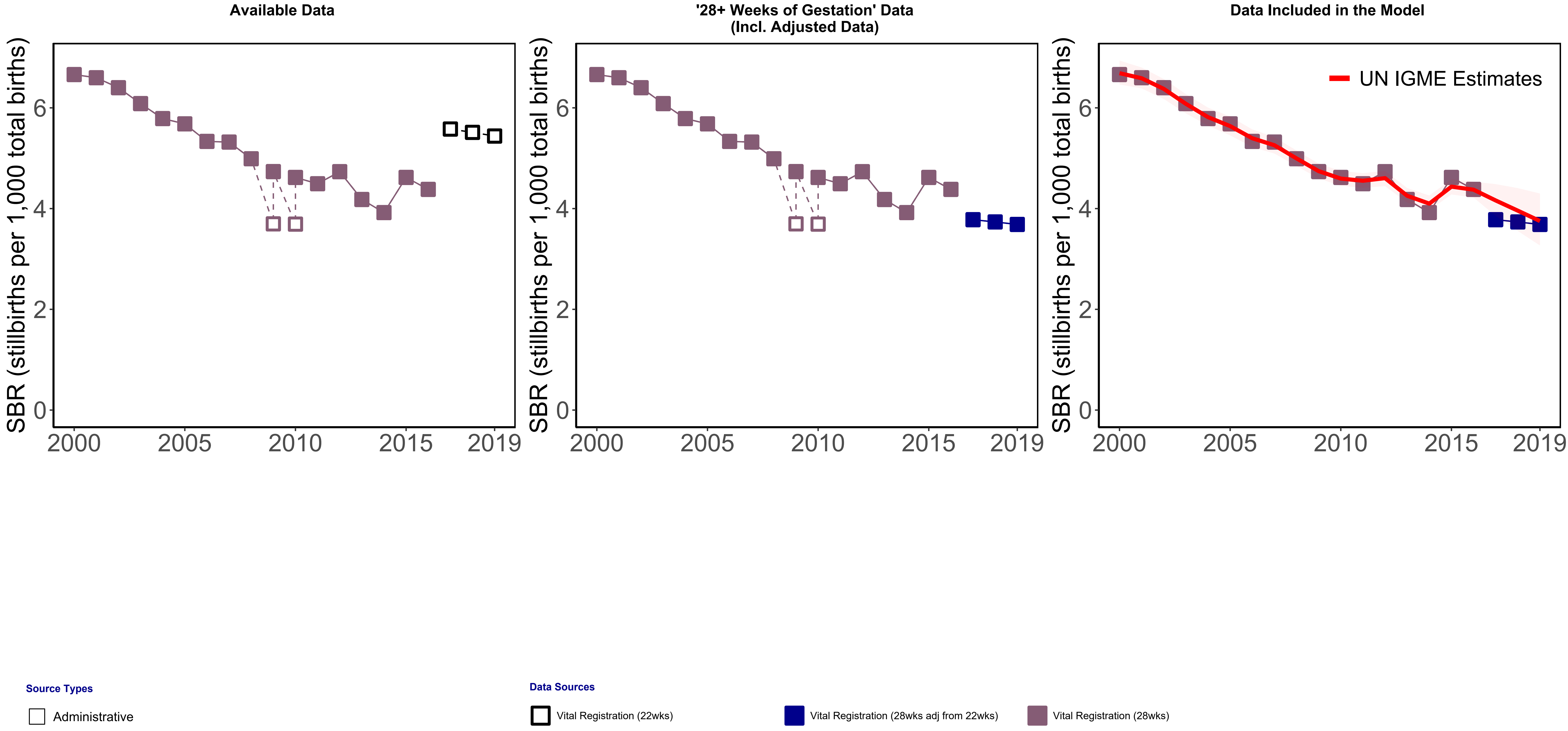

Rwanda

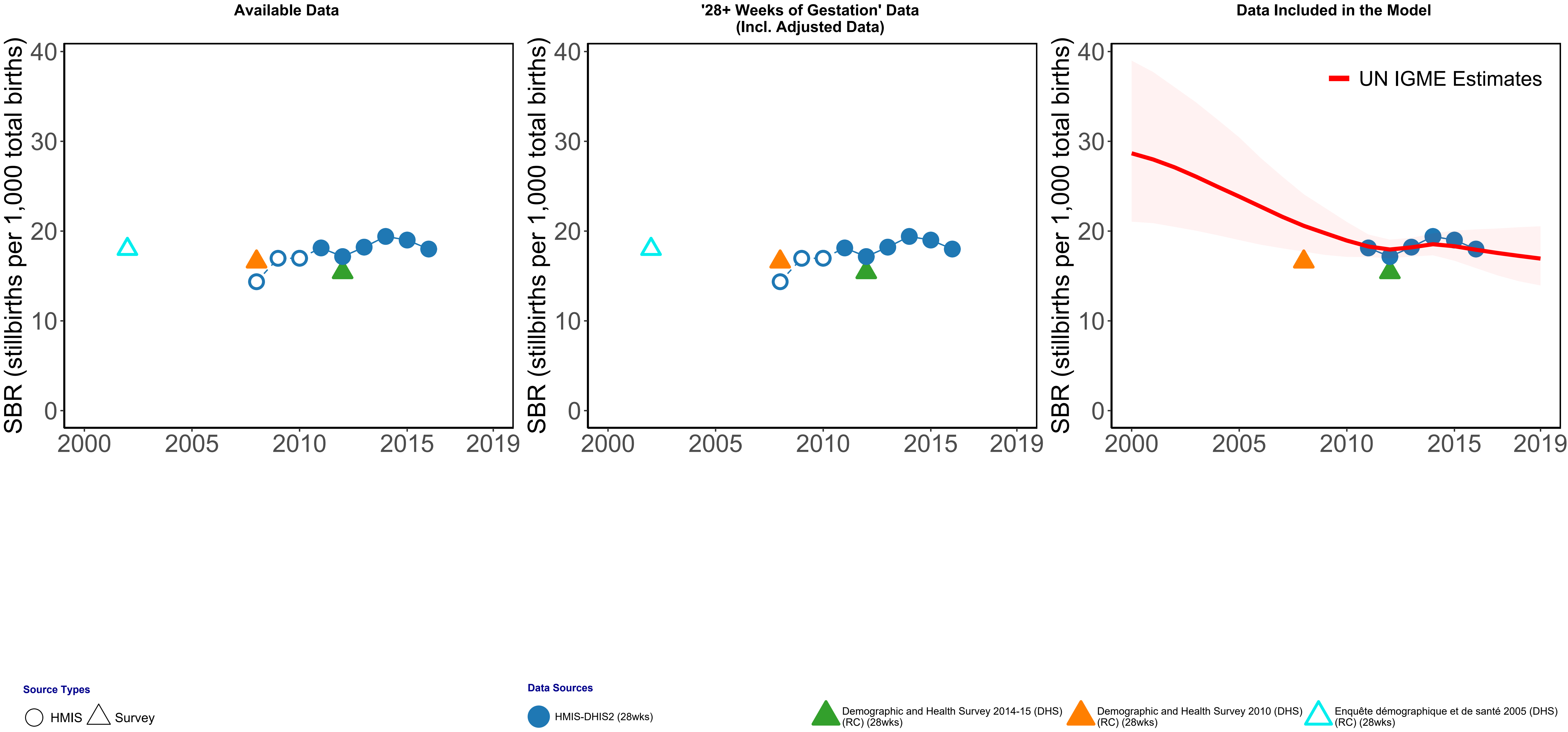
Available Data
'28+ Weeks of Gestation' Data
(Incl. Adjusted Data)
Data Included in the Model
SBR (stillbirths per 1,000 total births)
0
10
20
30
40
2000
2005
2010
2015
2019
UN IGME Estimates
Source Types
HMIS
Survey
Data Sources
HMIS-DHIS2 (28wks)
Demographic and Health Survey 2014-15 (DHS) (RC) (28wks)
Demographic and Health Survey 2010 (DHS) (RC) (28wks)
Enquête démographique et de santé 2005 (DHS) (RC) (28wks)

Saudi Arabia

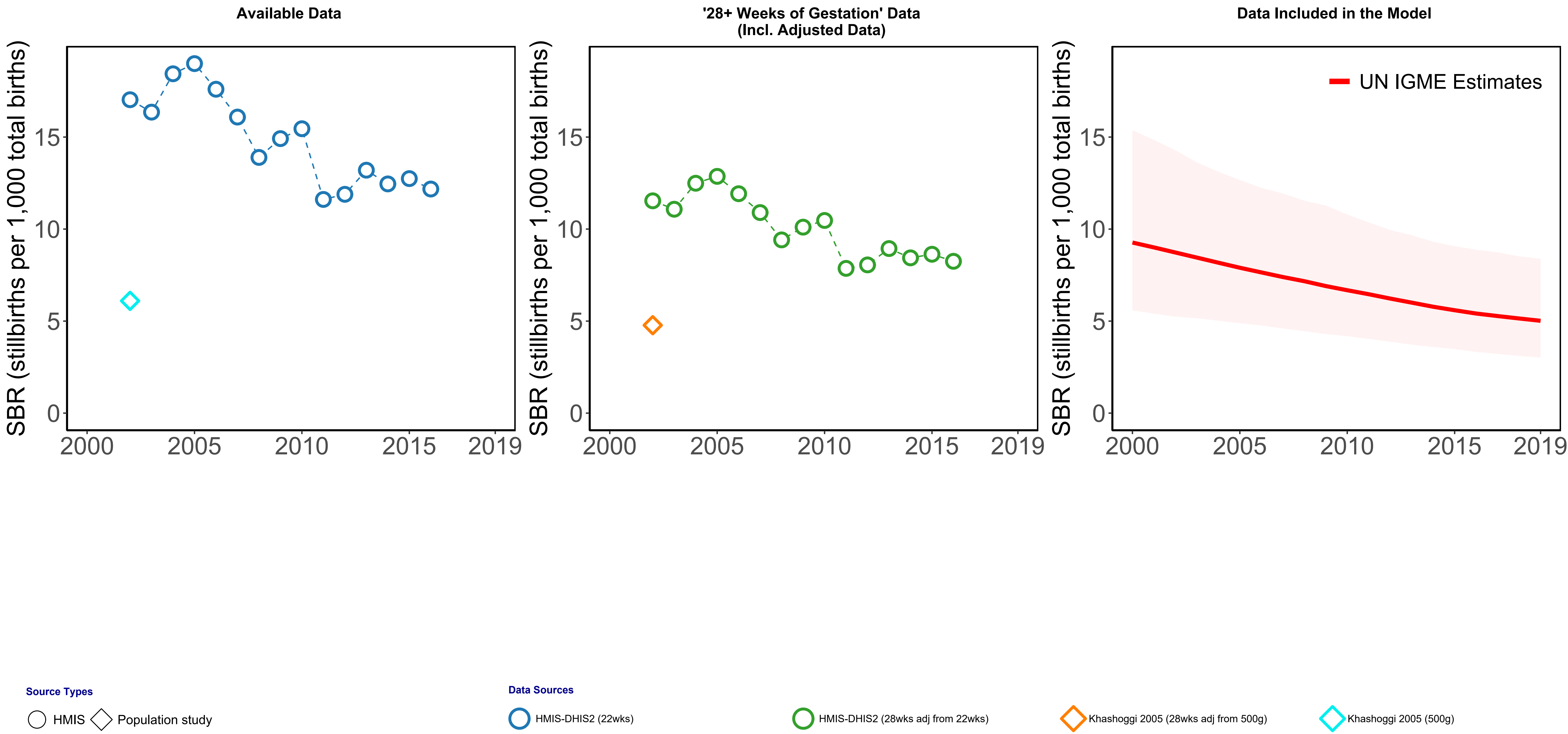

Sudan

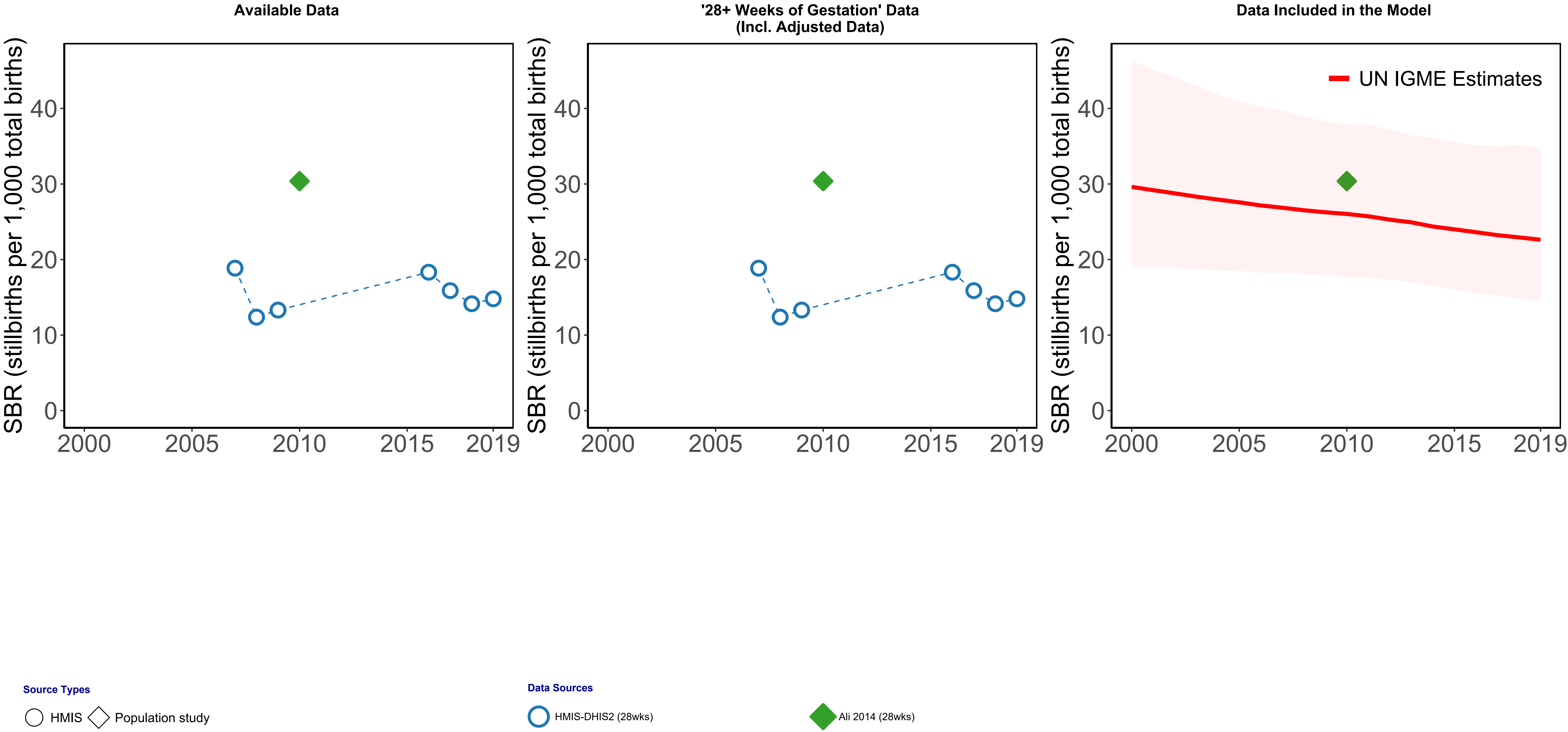
Available Data
'28+ Weeks of Gestation' Data
(Incl. Adjusted Data)
Data Included in the Model
SBR (stillbirths per 1,000 total births)
0
10
20
30
40
2000
2005
2010
2015
2019
UN IGME Estimates
Source Types
HMIS
Population study
Data Sources
HMIS-DHIS2 (28wks)
Ali 2014 (28wks)

Senegal

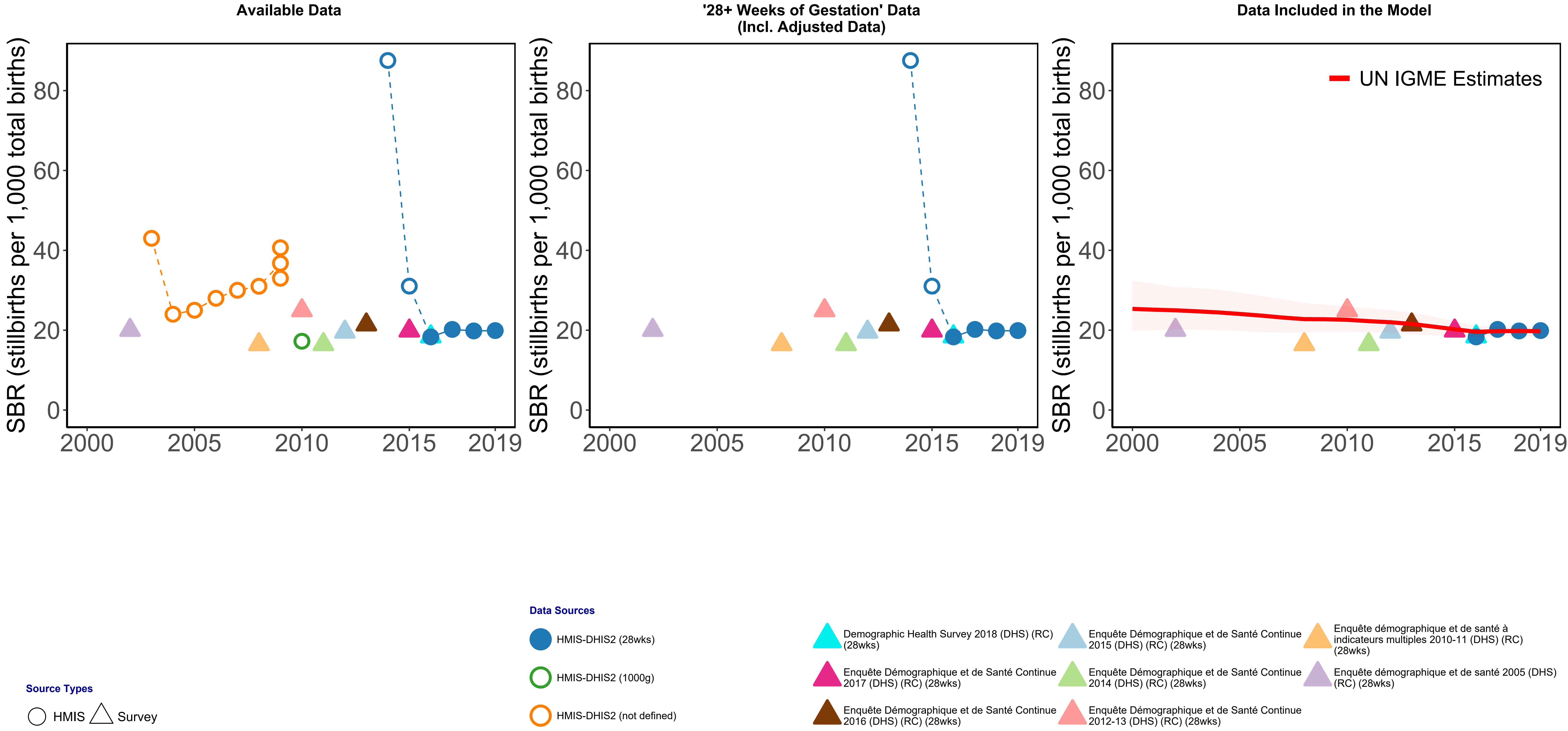

Available Data
'28+ Weeks of Gestation' Data
(Incl. Adjusted Data)
Data Included in the Model
SBR (stillbirths per 1,000 total births)
0
20
40
60
80
2000
2005
2010
2015
2019
UN IGME Estimates
Data Sources
HMIS-DHIS2 (28wks)
HMIS-DHIS2 (1000g)
HMIS-DHIS2 (not defined)
Demographic Health Survey 2018 (DHS) (RC) (28wks)
Enquête Démographique et de Santé Continue 2017 (DHS) (RC) (28wks)
Enquête Démographique et de Santé Continue 2016 (DHS) (RC) (28wks)
Enquête Démographique et de Santé Continue 2015 (DHS) (RC) (28wks)
Enquête Démographique et de Santé Continue 2014 (DHS) (RC) (28wks)
Enquête Démographique et de Santé Continue 2012-13 (DHS) (RC) (28wks)
Enquête démographique et de santé à indicateurs multiples 2010-11 (DHS) (RC) (28wks)
Enquête démographique et de santé 2005 (DHS) (RC) (28wks)
Source Types
HMIS
Survey

Singapore

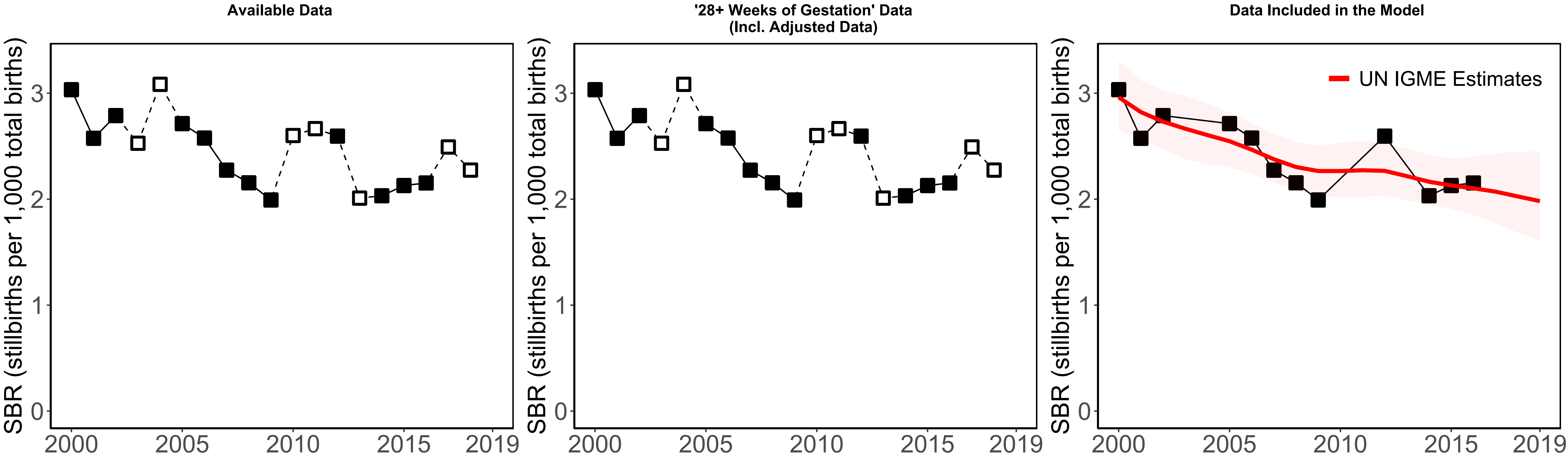

Source Types

Administrative

Data Sources

Vital Registration (28wks)

Solomon Islands

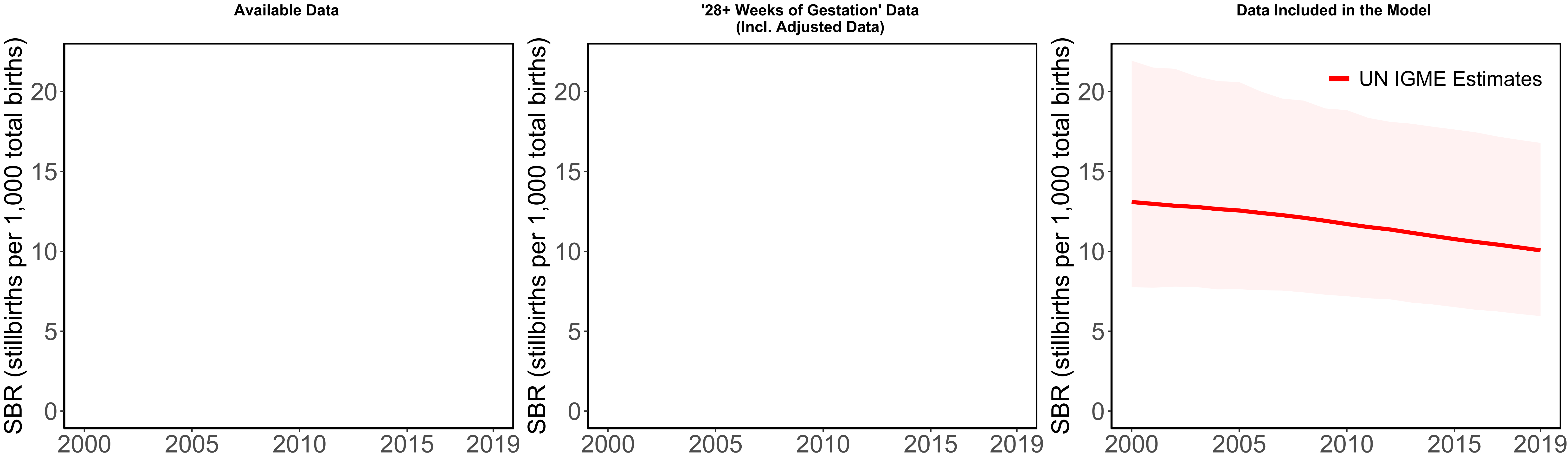

Sierra Leone

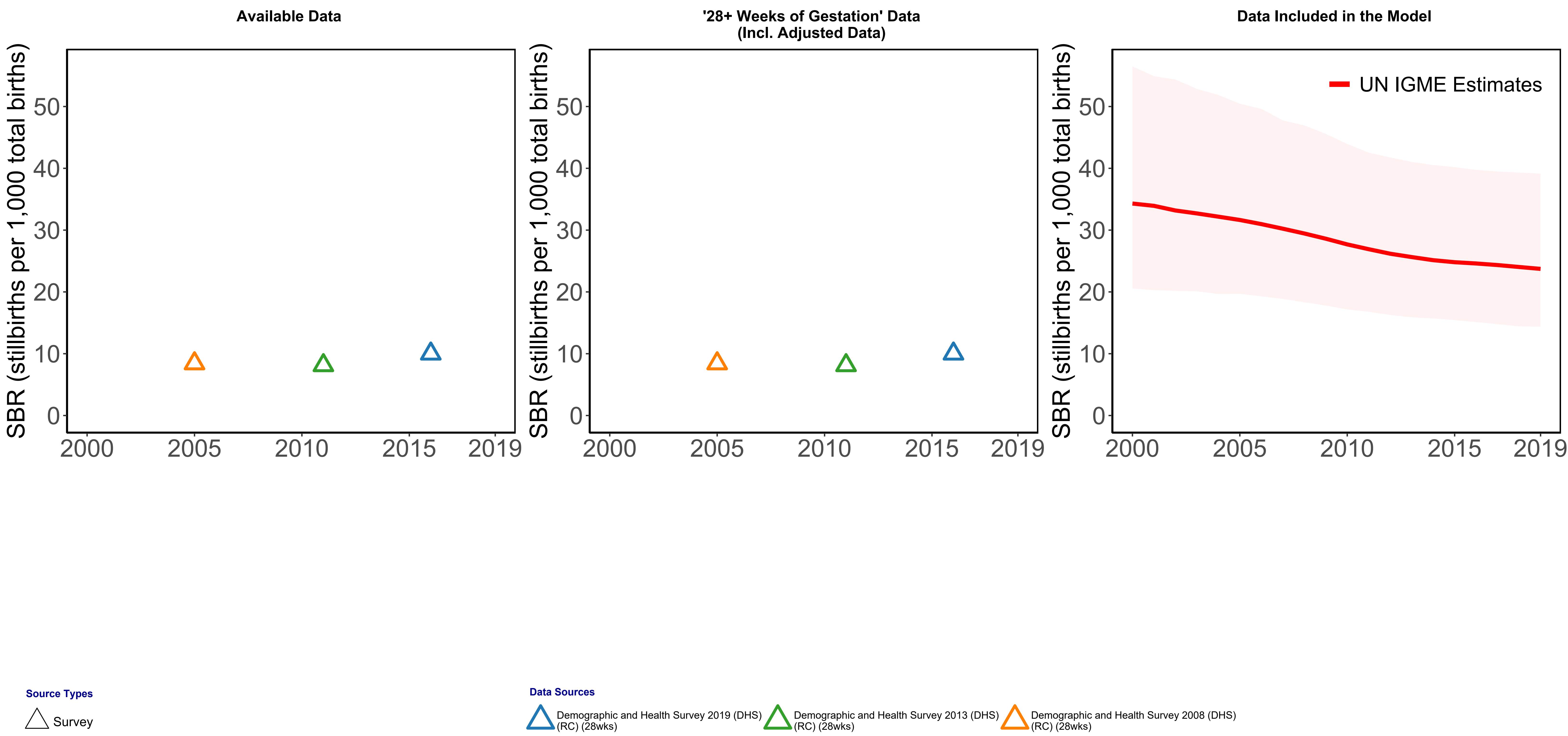

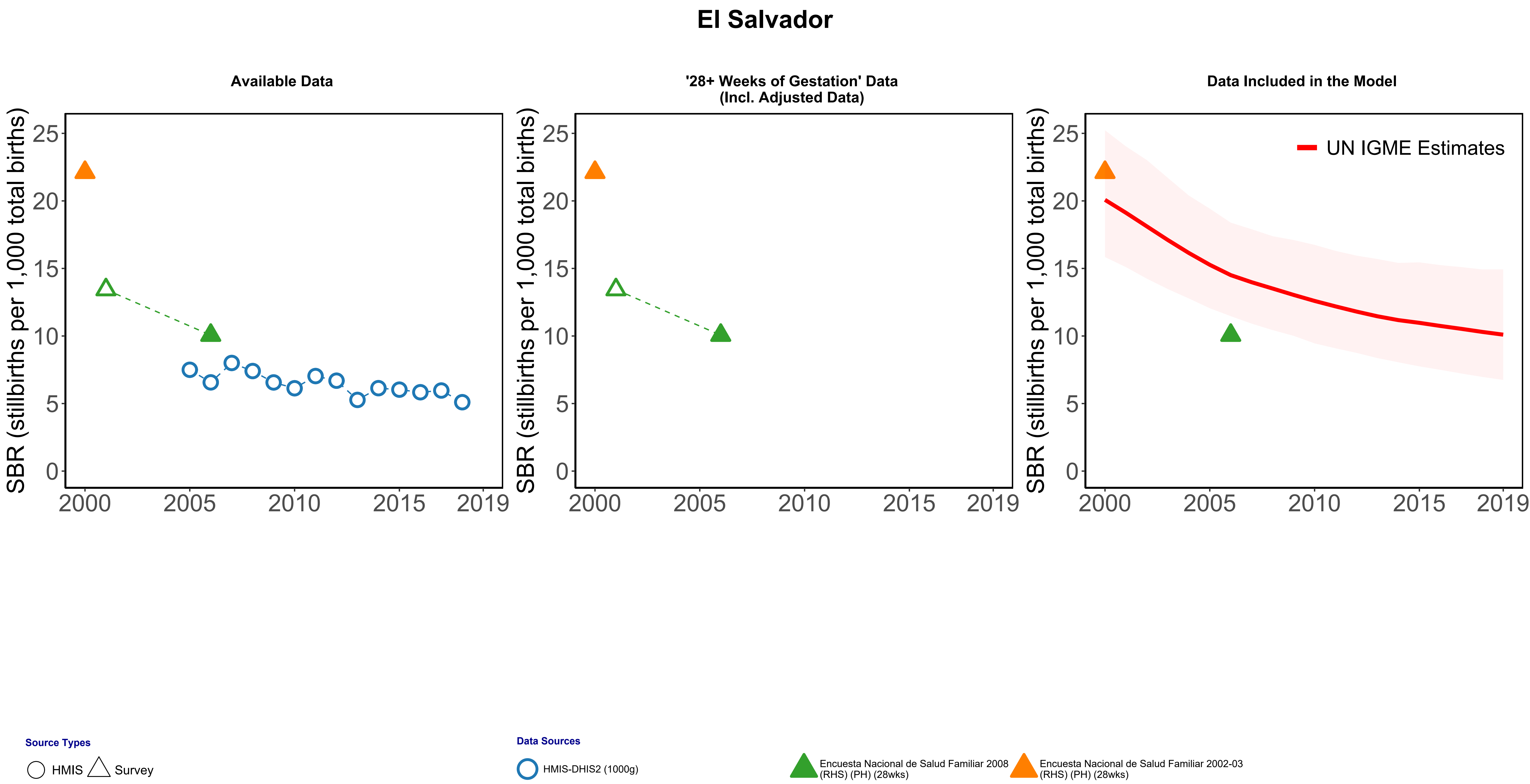
El Salvador
Available Data
'28+ Weeks of Gestation' Data
(Incl. Adjusted Data)
Data Included in the Model
SBR (stillbirths per 1,000 total births)
0
5
10
15
20
25
2000
2005
2010
2015
2019
UN IGME Estimates
Source Types
HMIS
Survey
Data Sources
HMIS-DHIS2 (1000g)
Encuesta Nacional de Salud Familiar 2008 (RHS) (PH) (28wks)
Encuesta Nacional de Salud Familiar 2002-03 (RHS) (PH) (28wks)

San Marino

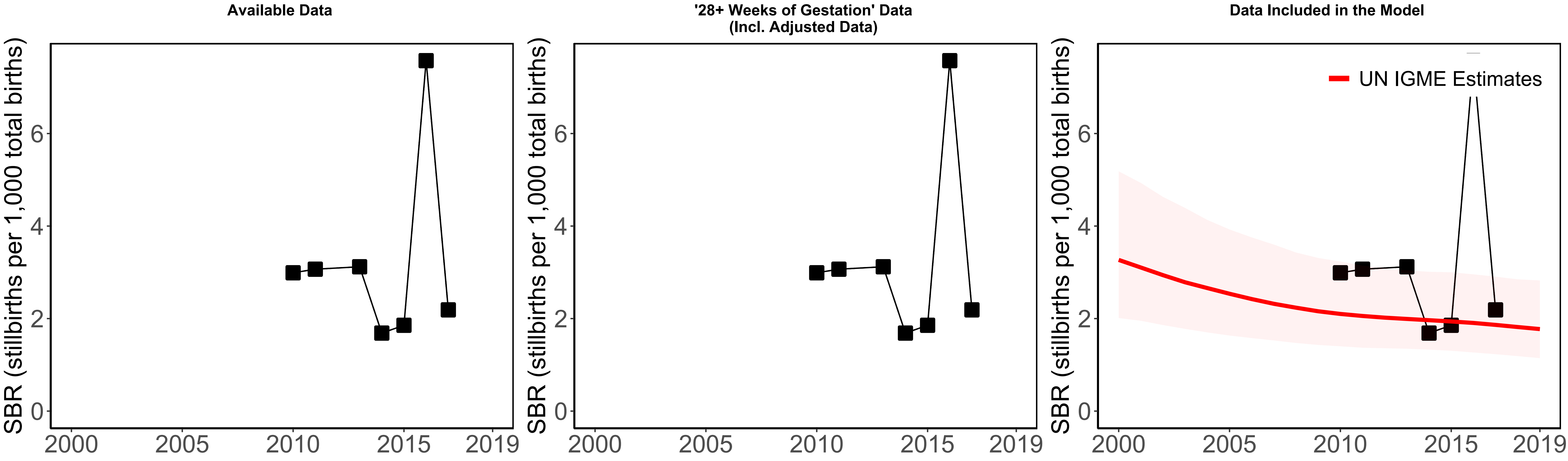

Source Types

Administrative

Data Sources

Vital Registration (28wks)

Somalia

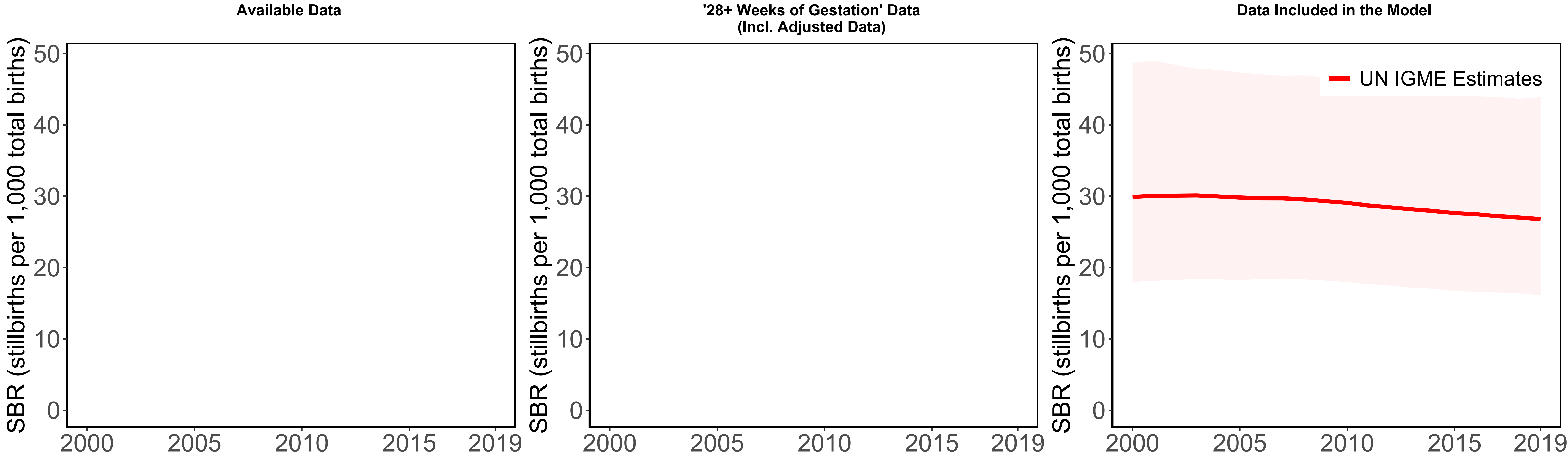

Available Data
'28+ Weeks of Gestation' Data
(Incl. Adjusted Data)
Data Included in the Model
SBR (stillbirths per 1,000 total births)
0
10
20
30
40
50
2000
2005
2010
2015
2019
UN IGME Estimates

Serbia

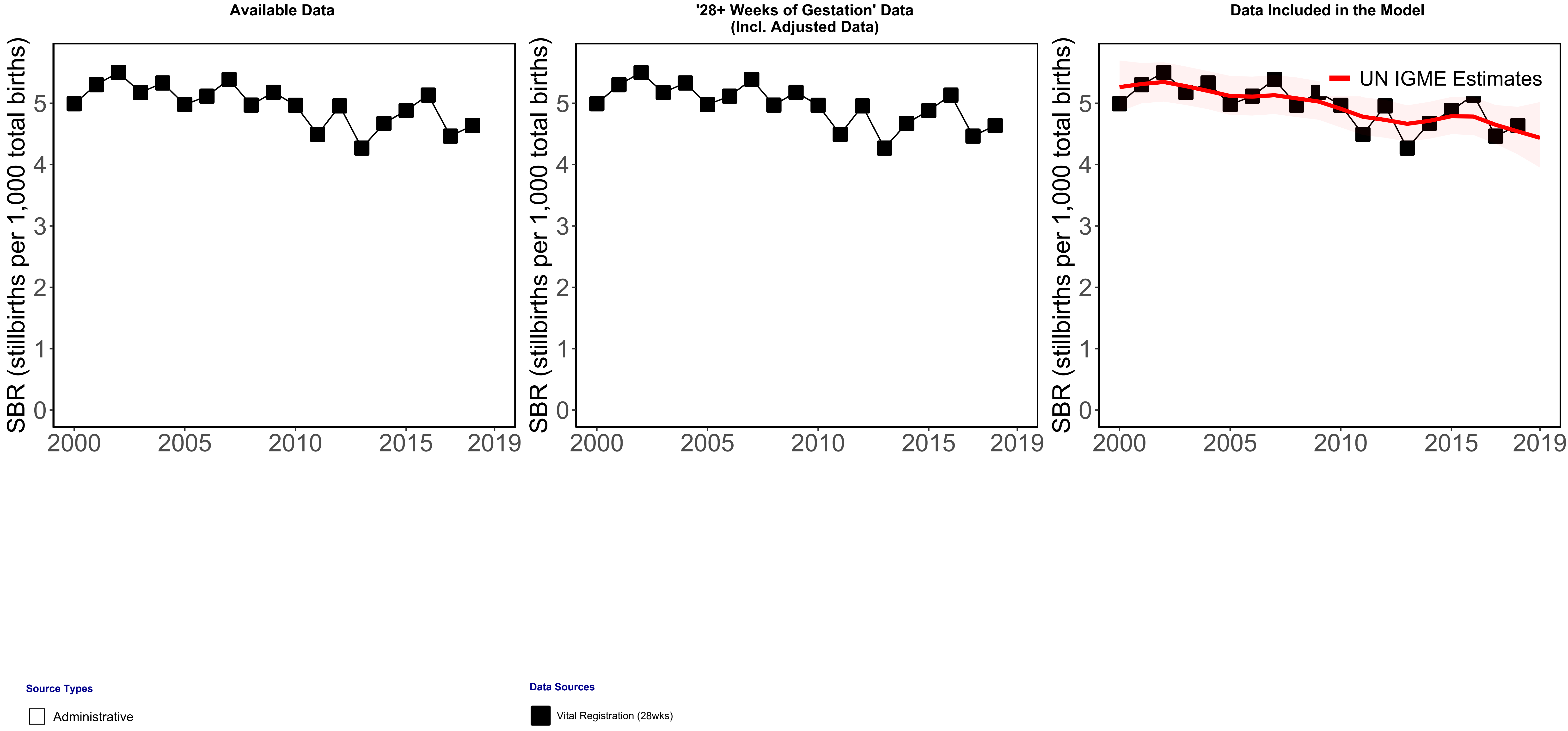

Available Data
'28+ Weeks of Gestation' Data
(Incl. Adjusted Data)
Data Included in the Model
SBR (stillbirths per 1,000 total births)
0
1
2
3
4
5
2000
2005
2010
2015
2019
UN IGME Estimates
Source Types
Administrative
Data Sources
Vital Registration (28wks)

South Sudan

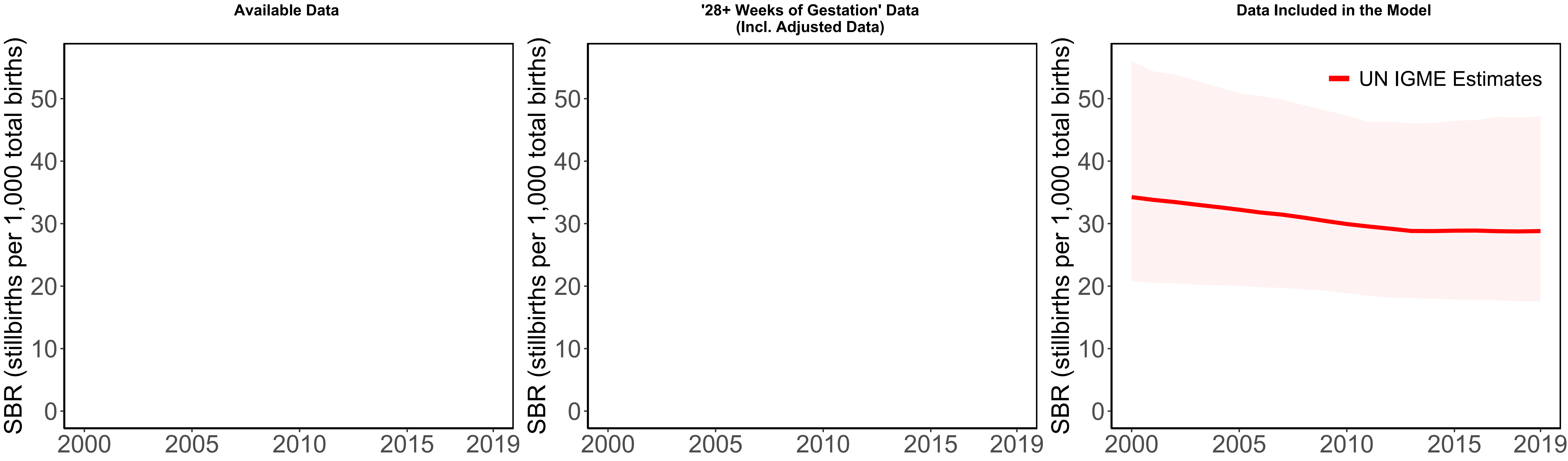

Available Data
'28+ Weeks of Gestation' Data
(Incl. Adjusted Data)
Data Included in the Model
SBR (stillbirths per 1,000 total births)
0
10
20
30
40
50
2000
2005
2010
2015
2019
UN IGME Estimates

Sao Tome and Principe

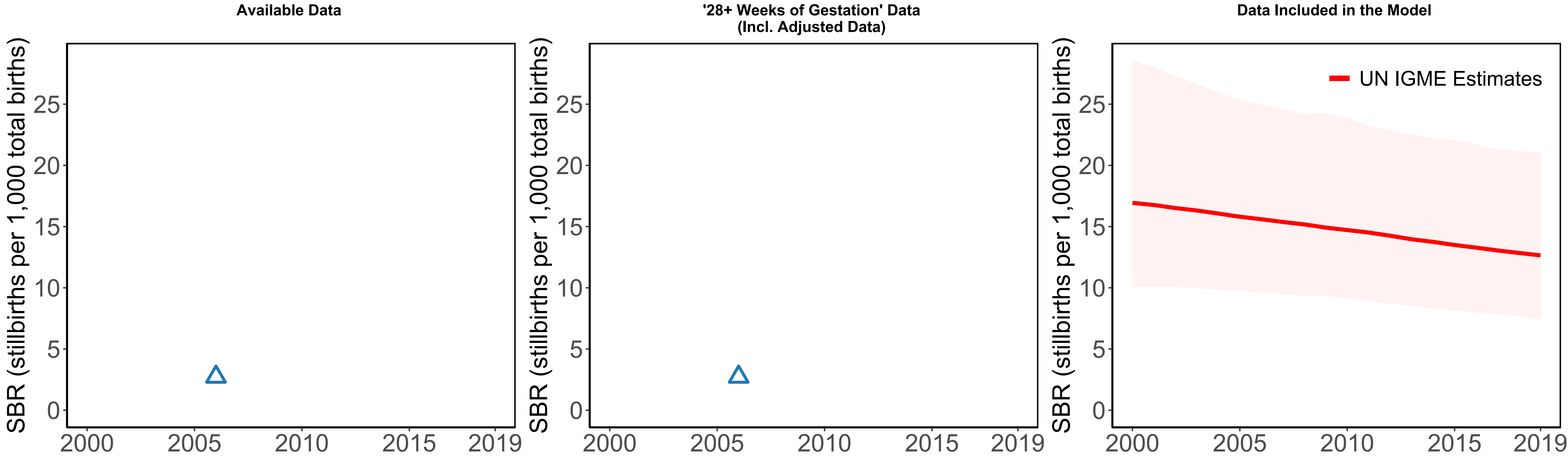

Source Types

Survey

Data Sources

Demographic and Health Survey 2008-09 (DHS) (BH/SQ) (28wks)

Suriname

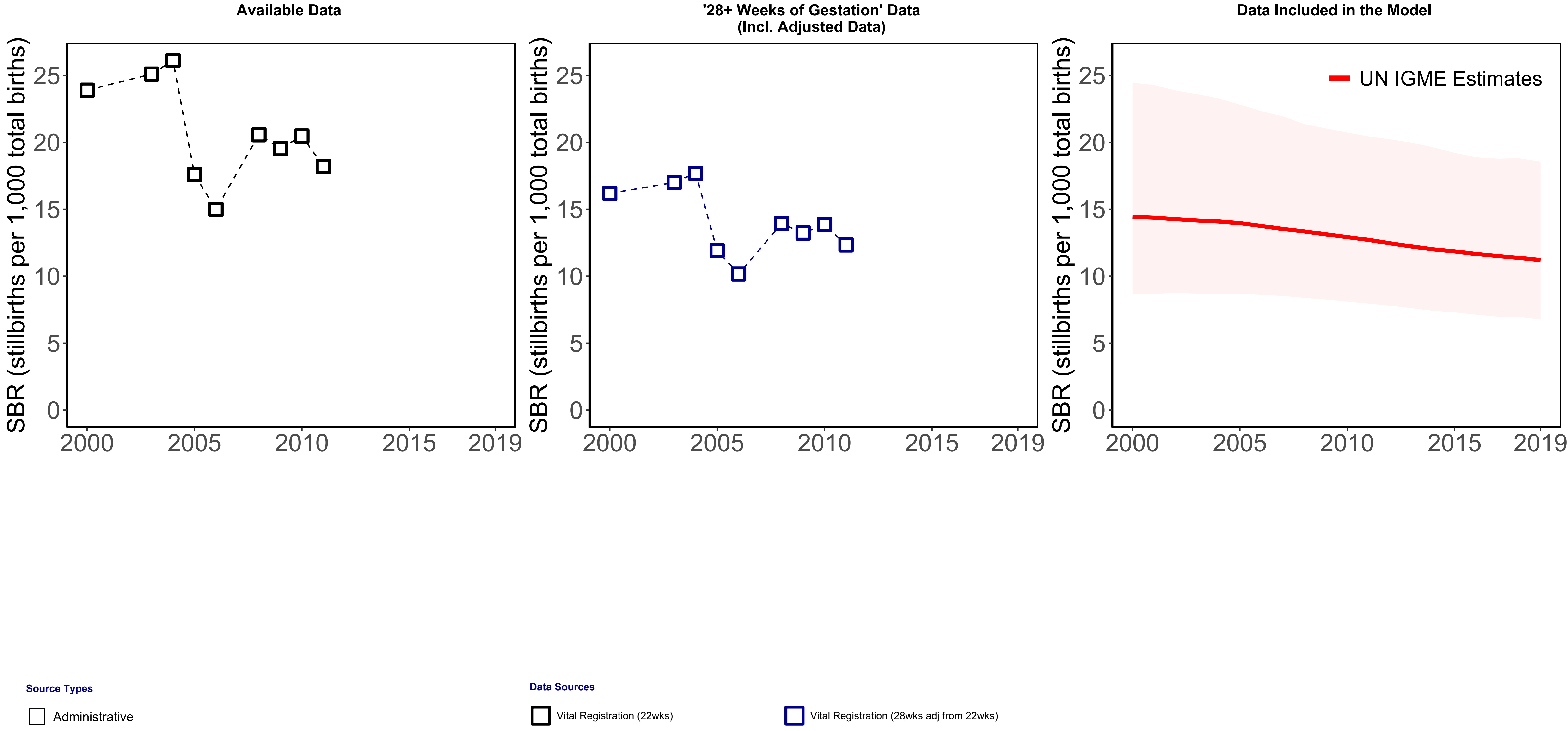
Available Data
'28+ Weeks of Gestation' Data
(Incl. Adjusted Data)
Data Included in the Model
SBR (stillbirths per 1,000 total births)
0
5
10
15
20
25
2000
2005
2010
2015
2019
UN IGME Estimates
Source Types
Administrative
Data Sources
Vital Registration (22wks)
Vital Registration (28wks adj from 22wks)

Slovakia

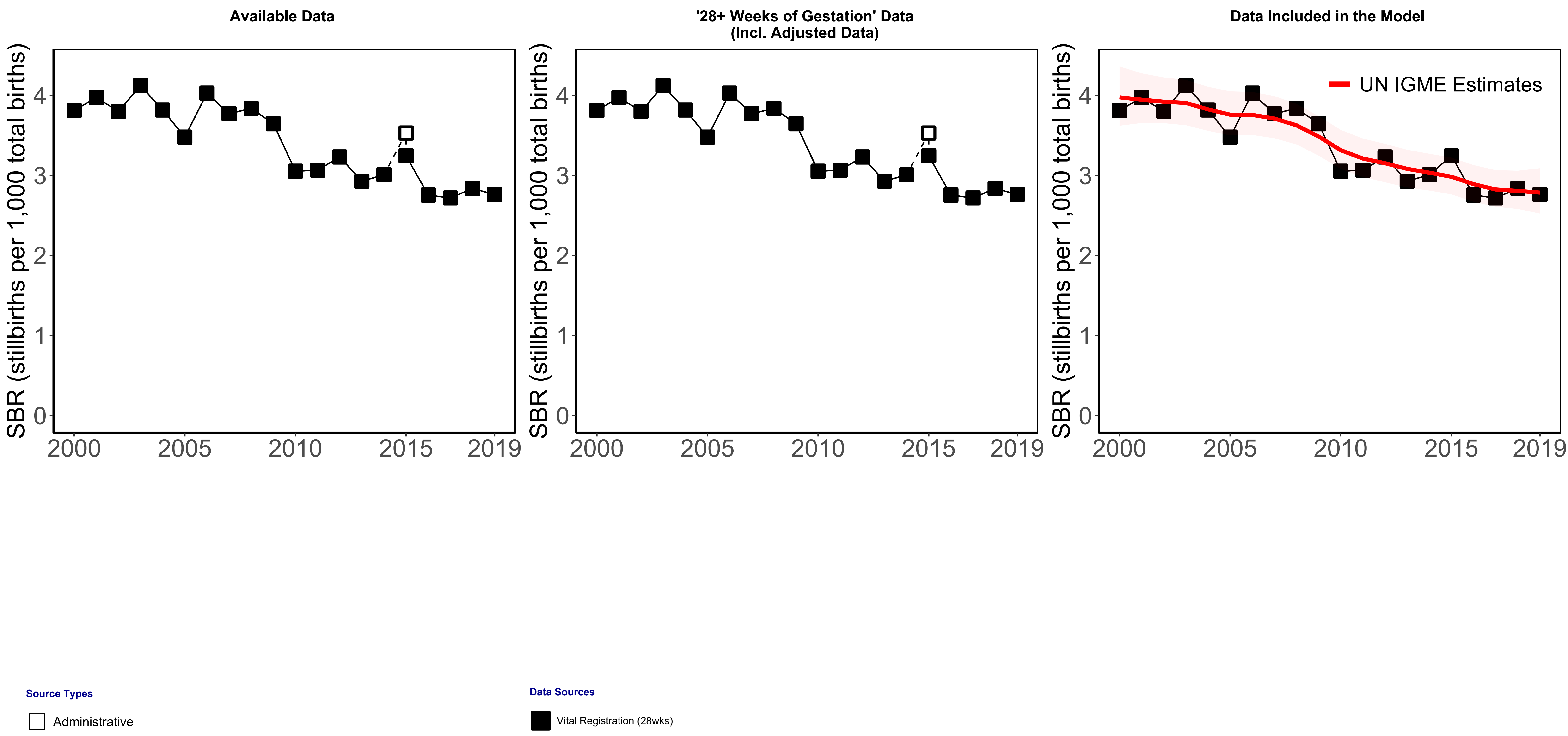
Available Data
'28+ Weeks of Gestation' Data
(Incl. Adjusted Data)
Data Included in the Model
SBR (stillbirths per 1,000 total births)
0
1
2
3
4
2000
2005
2010
2015
2019
UN IGME Estimates
Source Types
Administrative
Data Sources
Vital Registration (28wks)

Slovenia

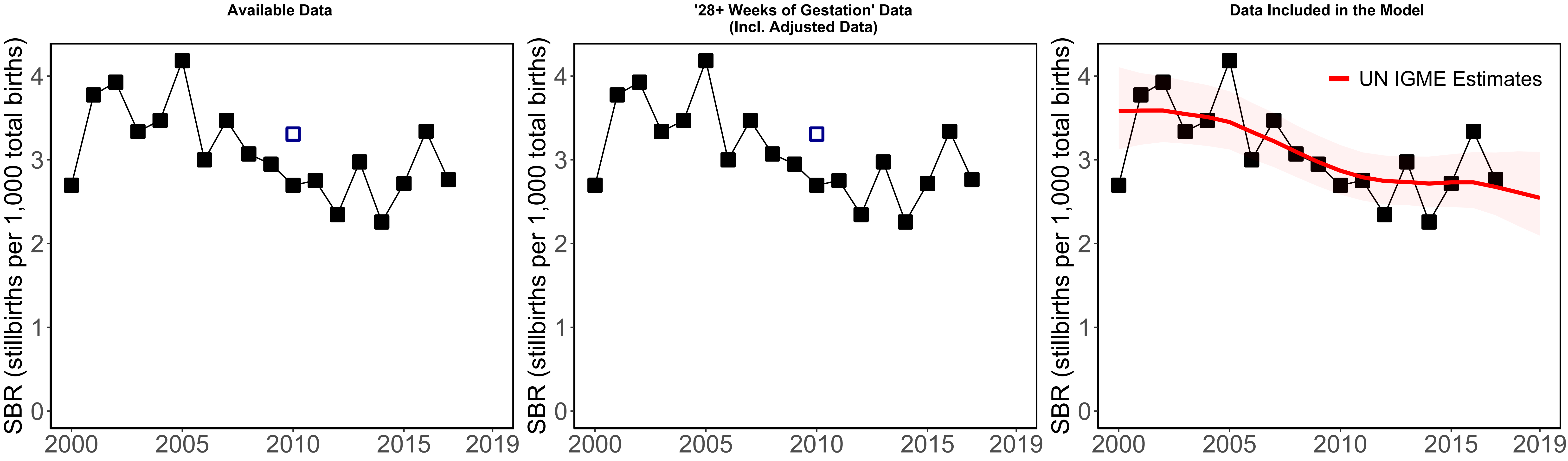

Available Data
'28+ Weeks of Gestation' Data
(Incl. Adjusted Data)
Data Included in the Model
SBR (stillbirths per 1,000 total births)
0
1
2
3
4
2000
2005
2010
2015
2019
UN IGME Estimates

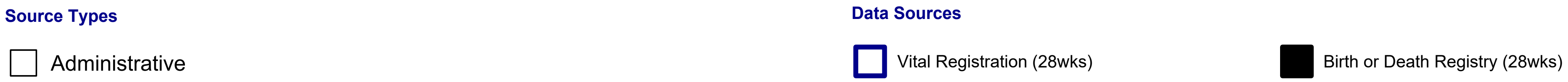

Source Types
Administrative
Data Sources
Vital Registration (28wks)
Birth or Death Registry (28wks)

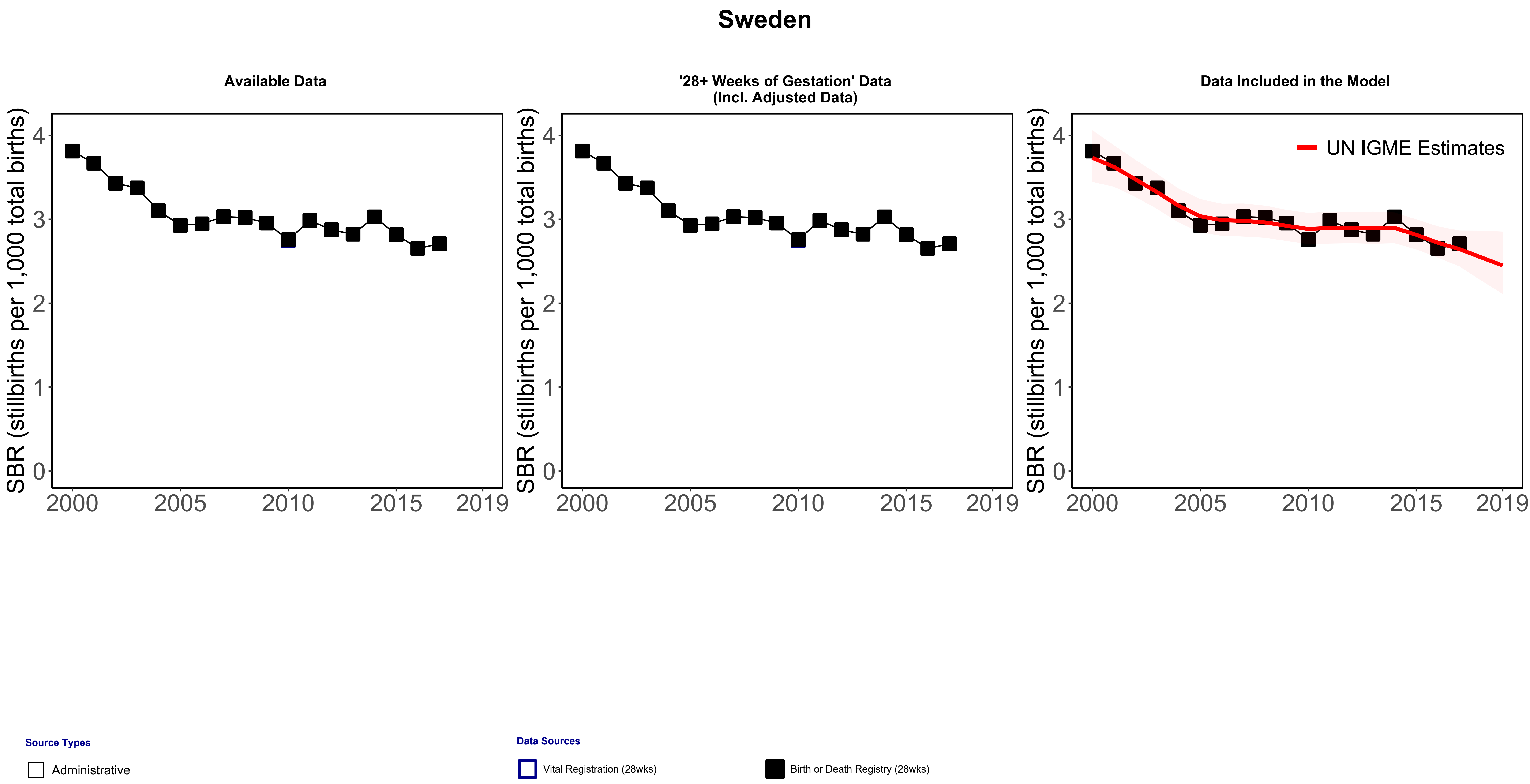
Sweden
Available Data
'28+ Weeks of Gestation' Data (Incl. Adjusted Data)
Data Included in the Model
SBR (stillbirths per 1,000 total births)
0
1
2
3
4
2000
2005
2010
2015
2019
UN IGME Estimates
Source Types
Administrative
Data Sources
Vital Registration (28wks)
Birth or Death Registry (28wks)

Eswatini

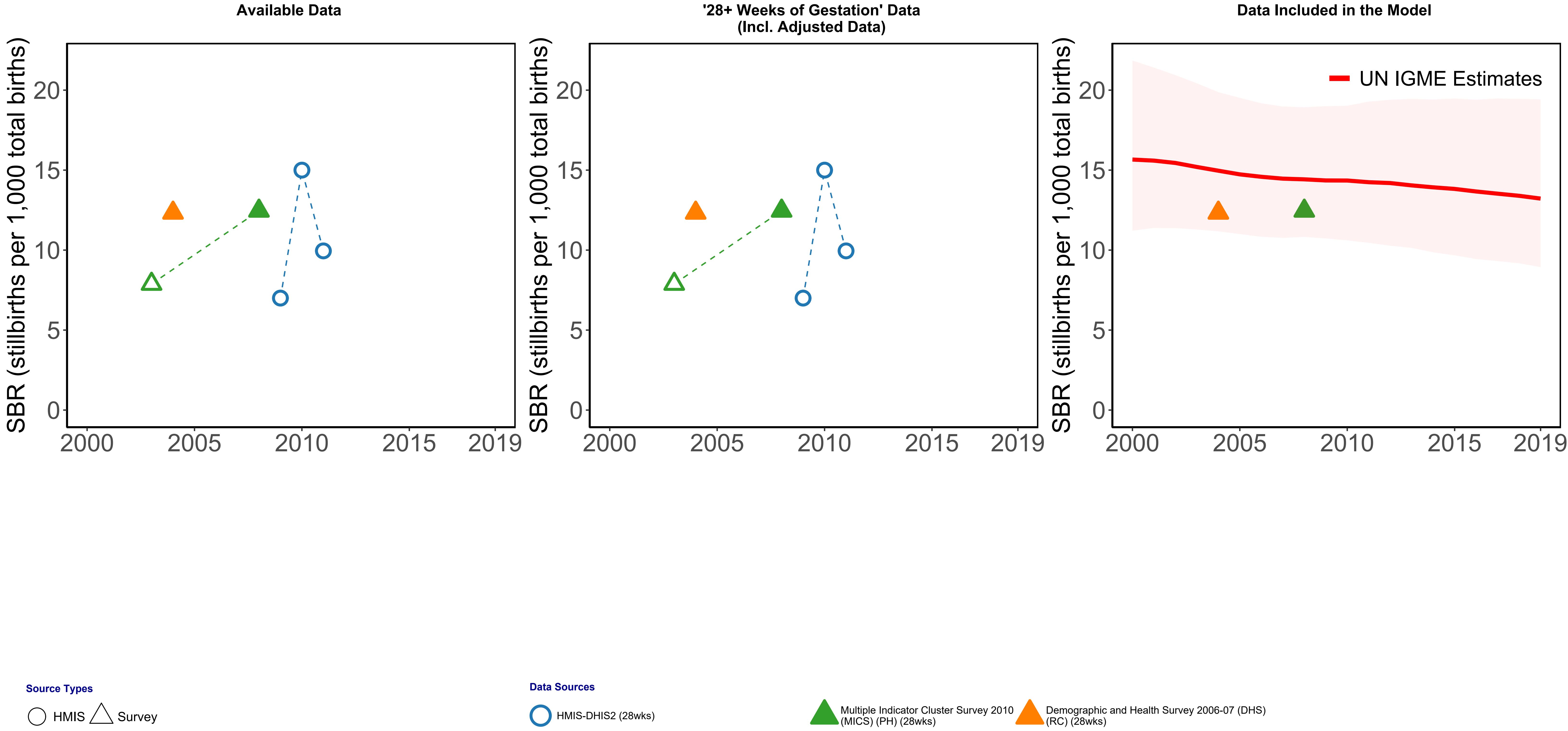
Available Data
'28+ Weeks of Gestation' Data
(Incl. Adjusted Data)
Data Included in the Model
SBR (stillbirths per 1,000 total births)
0
5
10
15
20
2000
2005
2010
2015
2019
UN IGME Estimates
Source Types
HMIS
Survey
Data Sources
HMIS-DHIS2 (28wks)
Multiple Indicator Cluster Survey 2010 (MICS) (PH) (28wks)
Demographic and Health Survey 2006-07 (DHS) (RC) (28wks)

Seychelles

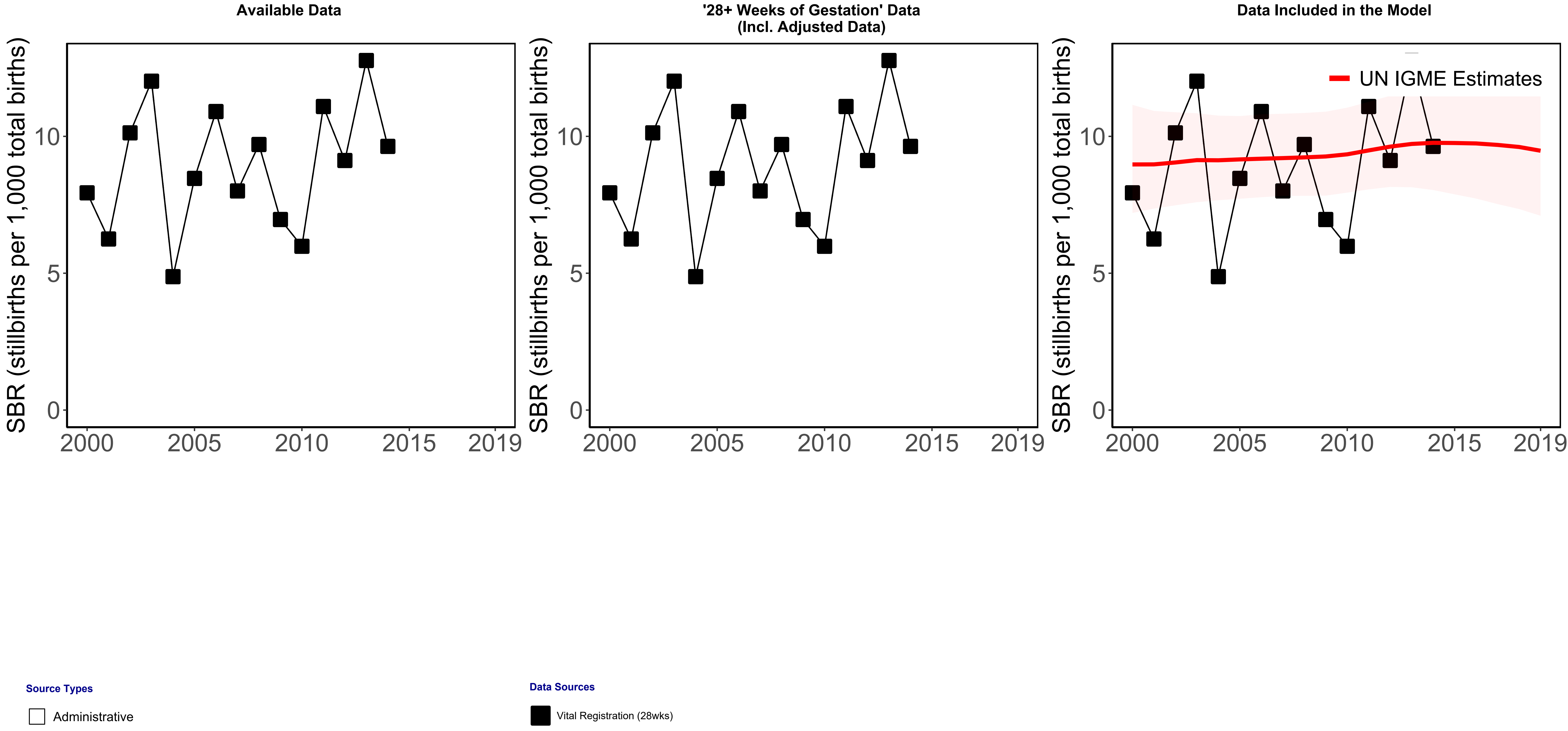

Available Data
'28+ Weeks of Gestation' Data (Incl. Adjusted Data)
Data Included in the Model
UN IGME Estimates
SBR (stillbirths per 1,000 total births)
0
5
10
2000
2005
2010
2015
2019
Source Types
Administrative
Data Sources
Vital Registration (28wks)

Syrian Arab Republic

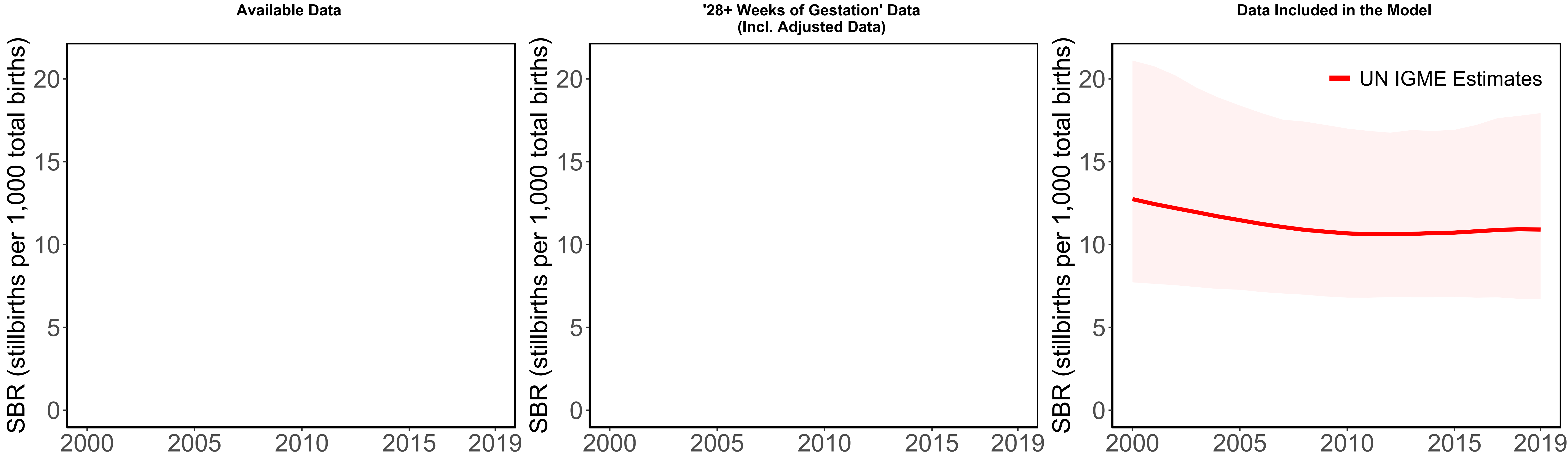

Chad

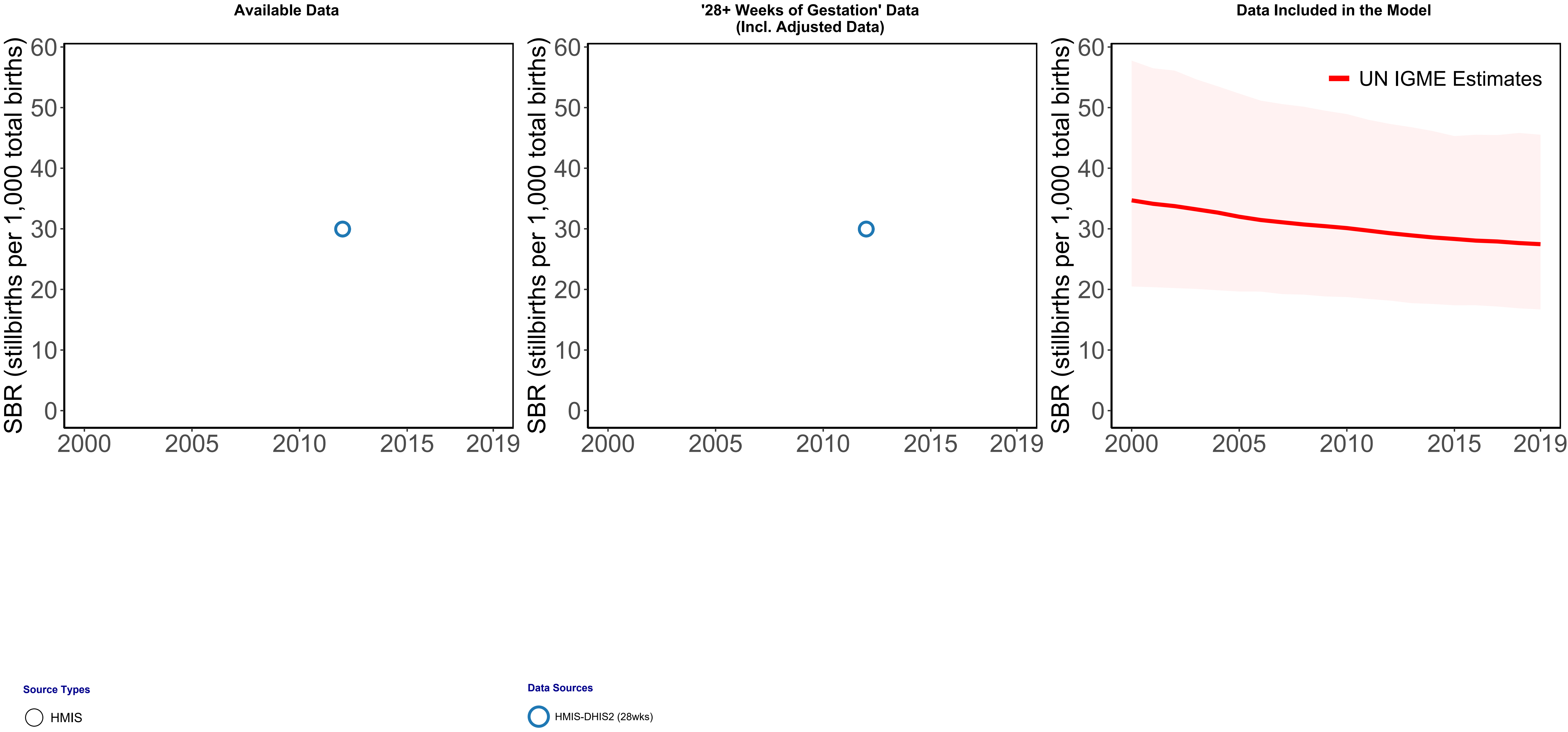

Available Data
'28+ Weeks of Gestation' Data
(Incl. Adjusted Data)
Data Included in the Model
SBR (stillbirths per 1,000 total births)
0
10
20
30
40
50
60
2000
2005
2010
2015
2019
UN IGME Estimates
Source Types
HMIS
Data Sources
HMIS-DHIS2 (28wks)

Togo

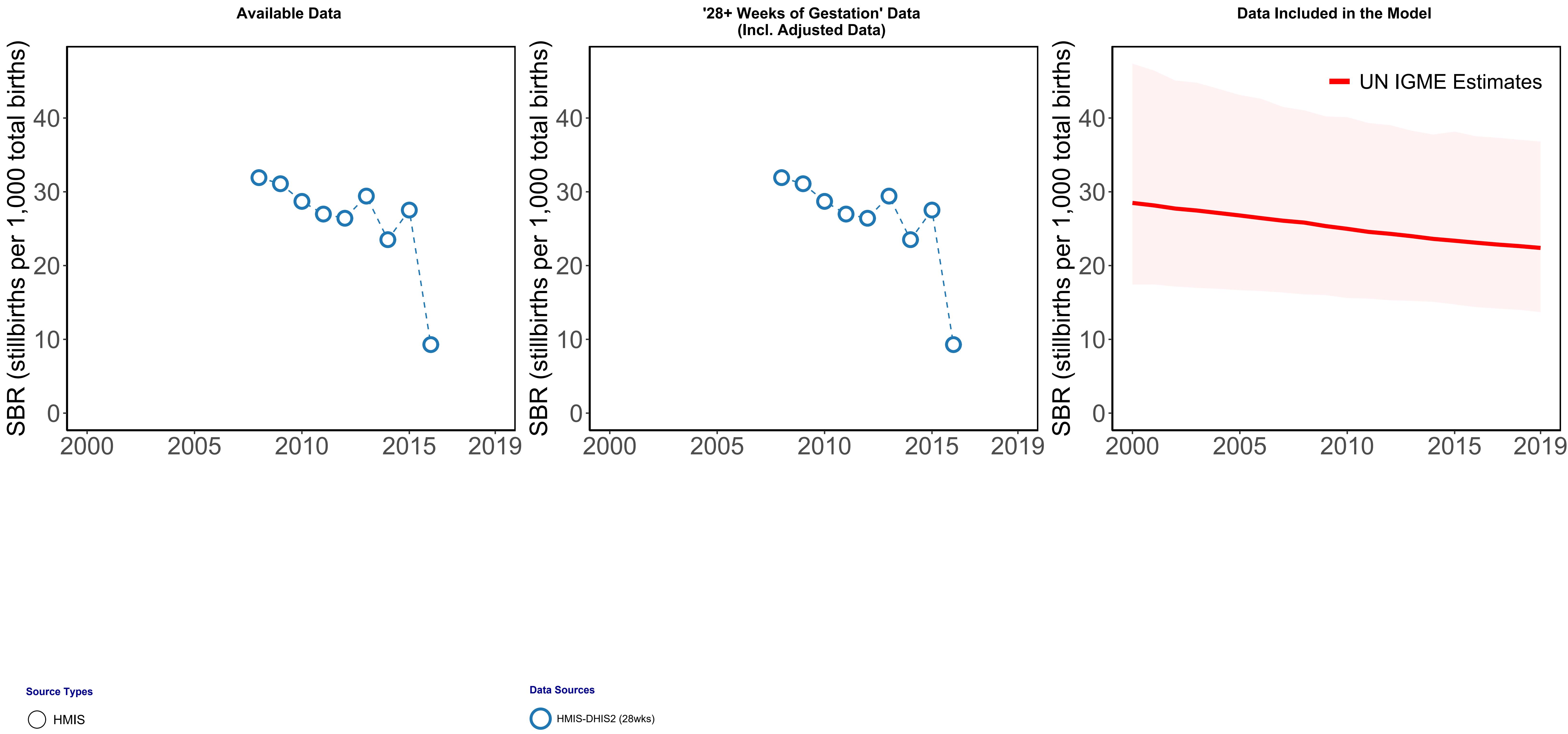
Available Data
'28+ Weeks of Gestation' Data
(Incl. Adjusted Data)
Data Included in the Model
SBR (stillbirths per 1,000 total births)
0
10
20
30
40
2000
2005
2010
2015
2019
UN IGME Estimates
Source Types
HMIS
Data Sources
HMIS-DHIS2 (28wks)

Thailand

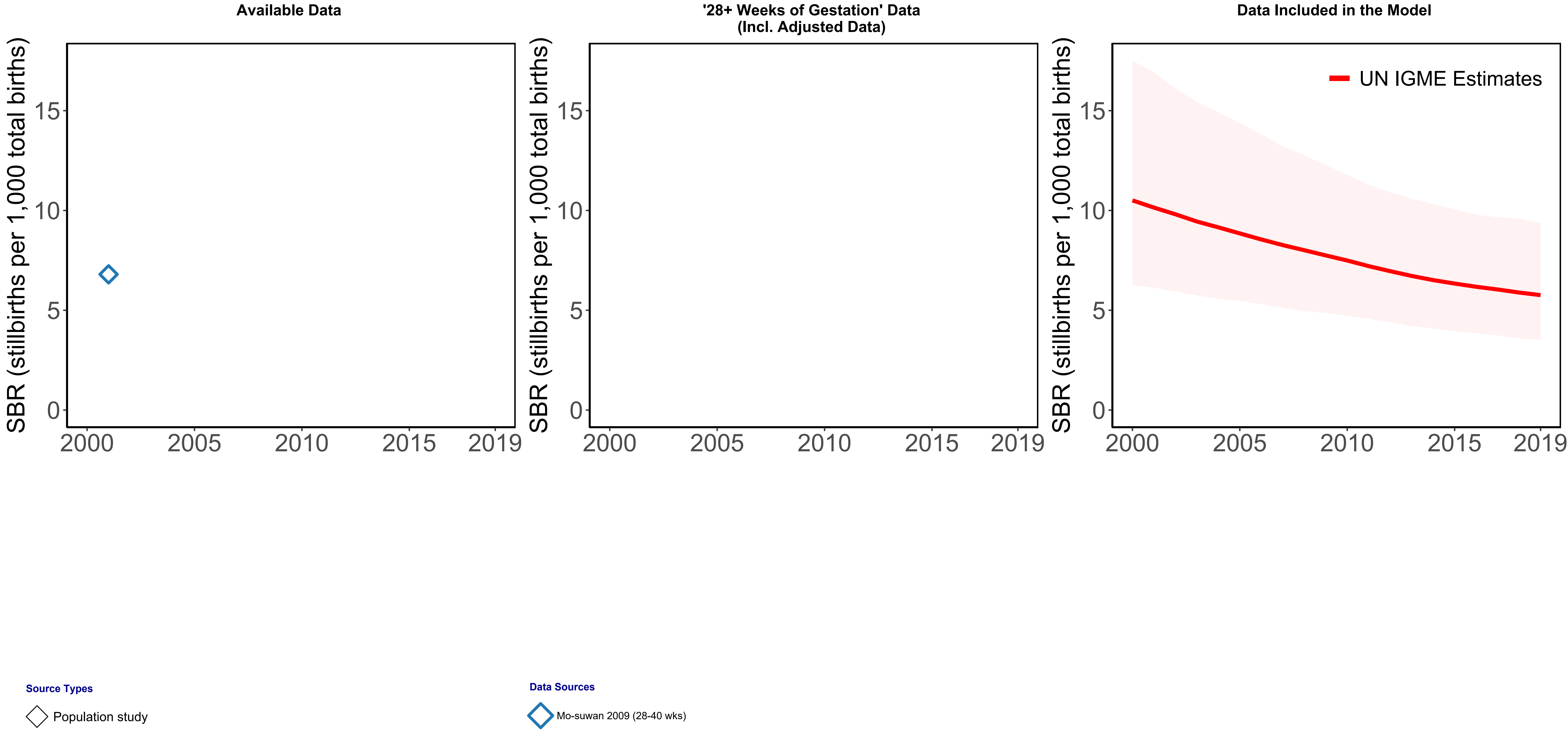
Available Data
'28+ Weeks of Gestation' Data
(Incl. Adjusted Data)
Data Included in the Model
SBR (stillbirths per 1,000 total births)
0
5
10
15
2000
2005
2010
2015
2019
UN IGME Estimates
Source Types
Population study
Data Sources
Mo-suwan 2009 (28-40 wks)

Tajikistan

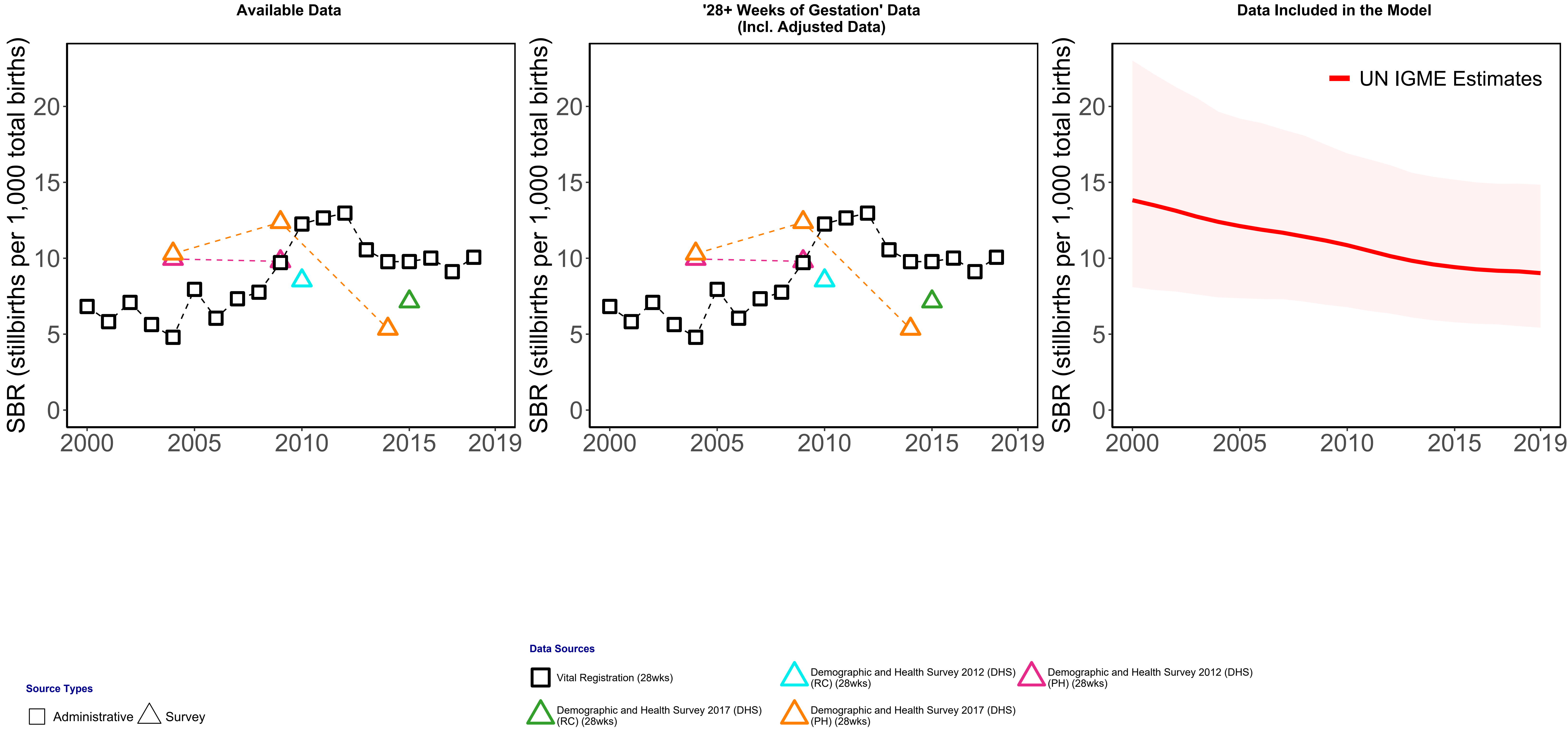

Available Data
'28+ Weeks of Gestation' Data
(Incl. Adjusted Data)
Data Included in the Model
SBR (stillbirths per 1,000 total births)
0
5
10
15
20
2000
2005
2010
2015
2019
UN IGME Estimates
Source Types
Administrative
Survey
Data Sources
Vital Registration (28wks)
Demographic and Health Survey 2012 (DHS) (RC) (28wks)
Demographic and Health Survey 2012 (DHS) (PH) (28wks)
Demographic and Health Survey 2017 (DHS) (RC) (28wks)
Demographic and Health Survey 2017 (DHS) (PH) (28wks)

Turkmenistan

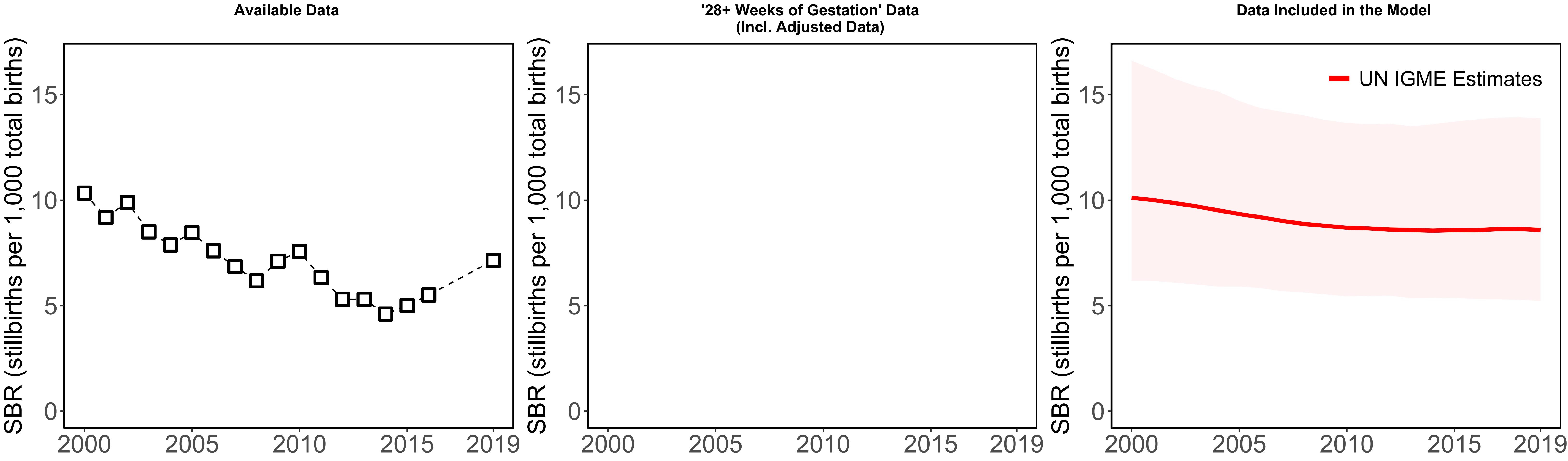

Source Types

Administrative

Data Sources

Vital Registration (not defined)

Timor-Leste

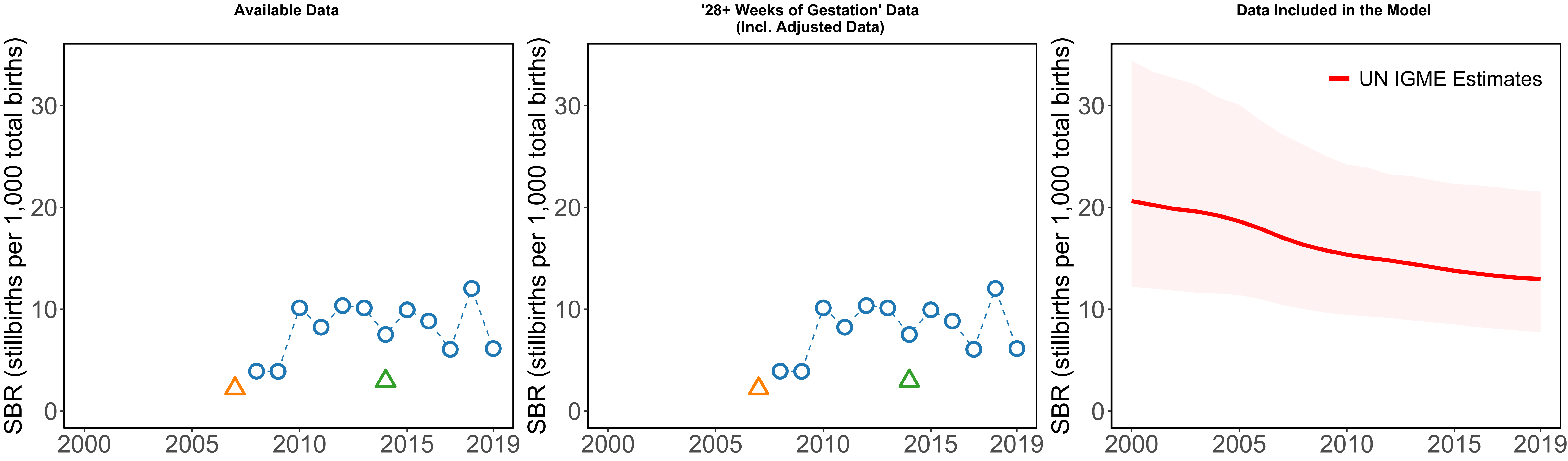

Source Types

HMIS Survey

Data Sources

HMIS-DHIS2 (28wks)

Demographic and Health Survey 2016 (DHS) (RC) (28wks)

Demographic and Health Survey 2009-10 (DHS) (RC) (28wks)

Tonga

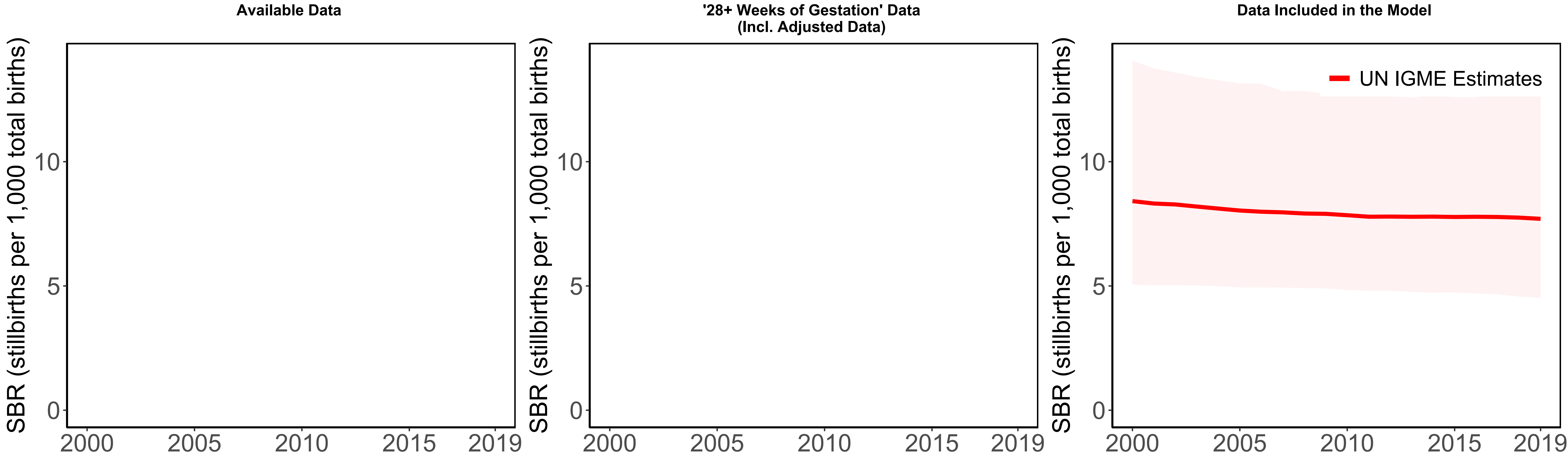

Available Data
'28+ Weeks of Gestation' Data
(Incl. Adjusted Data)
Data Included in the Model
SBR (stillbirths per 1,000 total births)
UN IGME Estimates
0
5
10
2000
2005
2010
2015
2019

Trinidad and Tobago

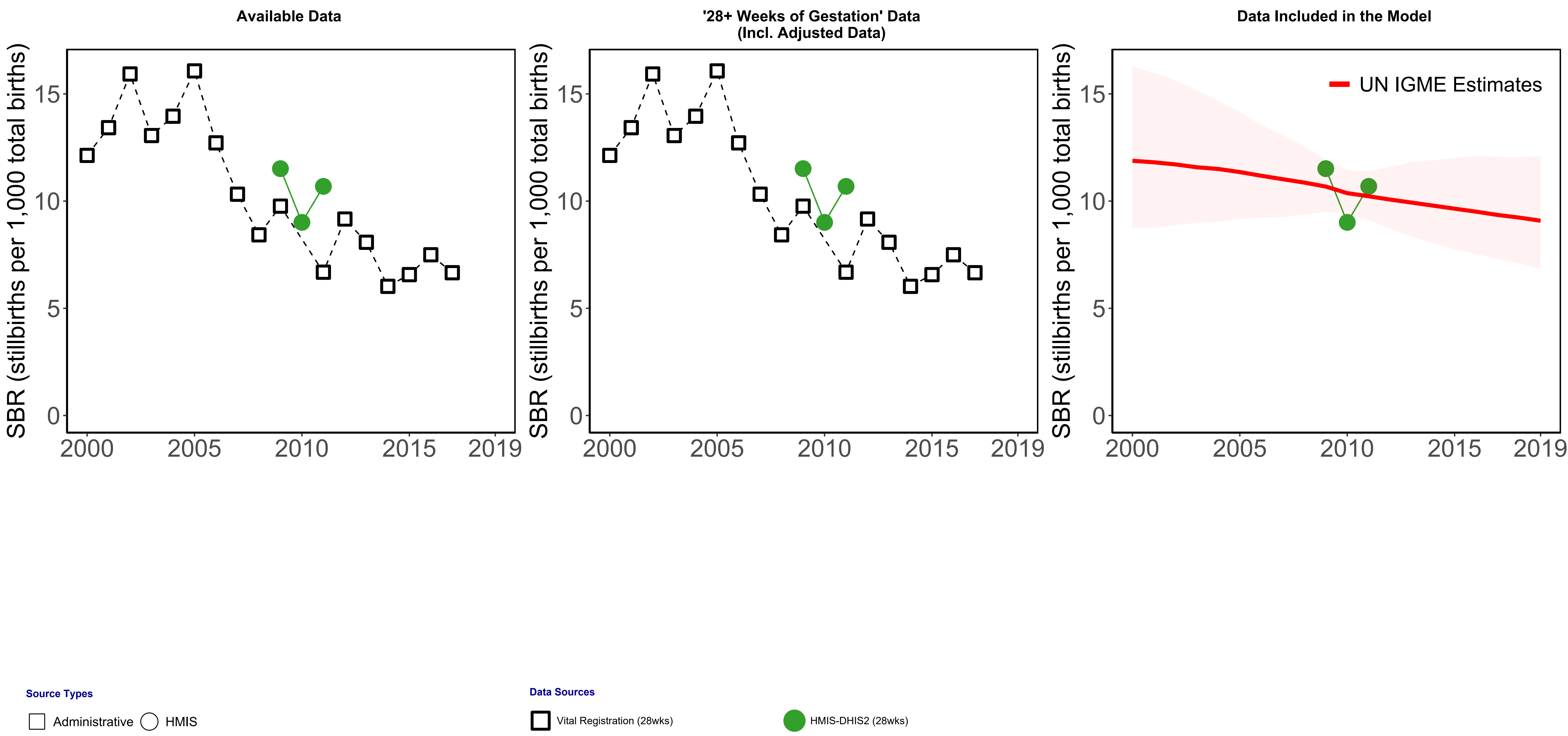

Tunisia

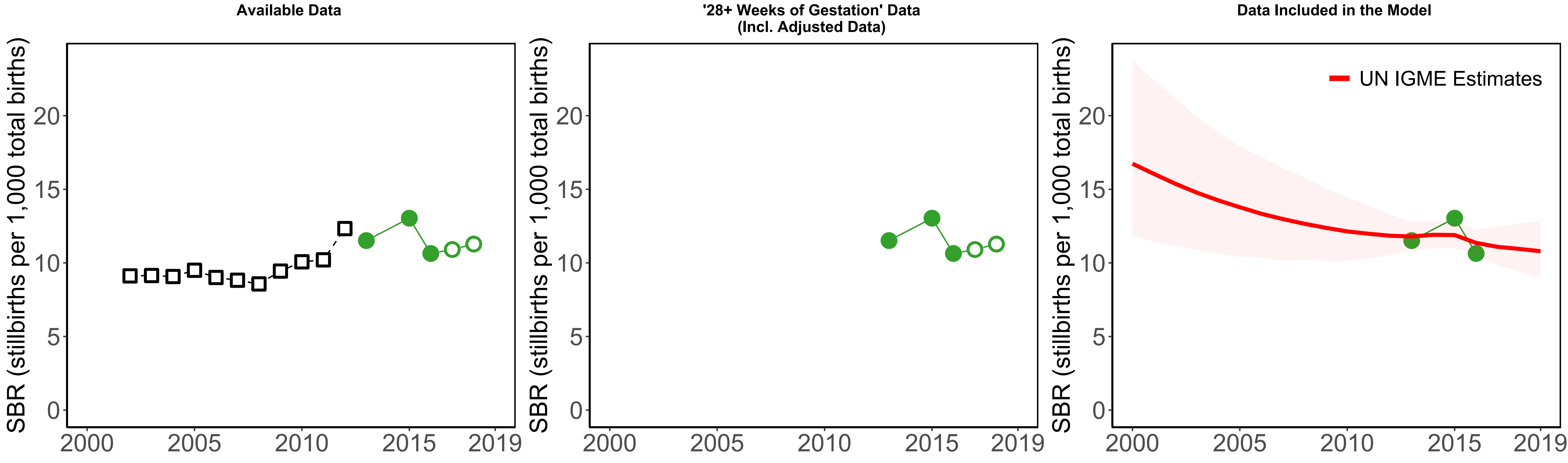

Source Types

Administrative HMIS

Data Sources

Vital Registration (not defined)

HMIS-DHIS2 (28wks)

Turkey

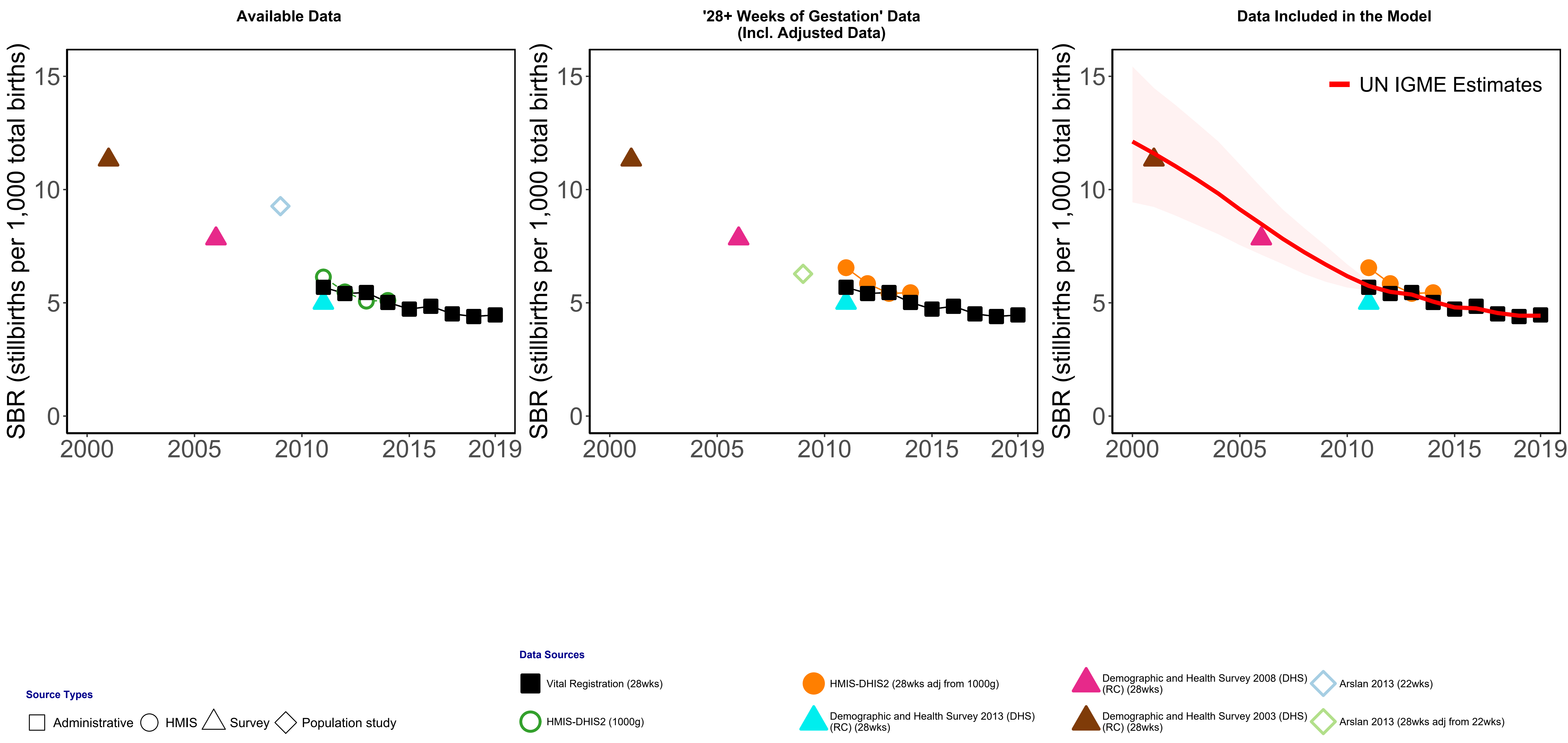
Available Data
'28+ Weeks of Gestation' Data
(Incl. Adjusted Data)
Data Included in the Model
SBR (stillbirths per 1,000 total births)
0
5
10
15
2000
2005
2010
2015
2019
UN IGME Estimates
Source Types
Administrative
HMIS
Survey
Population study
Data Sources
Vital Registration (28wks)
HMIS-DHIS2 (1000g)
HMIS-DHIS2 (28wks adj from 1000g)
Demographic and Health Survey 2013 (DHS) (RC) (28wks)
Demographic and Health Survey 2008 (DHS) (RC) (28wks)
Demographic and Health Survey 2003 (DHS) (RC) (28wks)
Arslan 2013 (22wks)
Arslan 2013 (28wks adj from 22wks)

Tuvalu

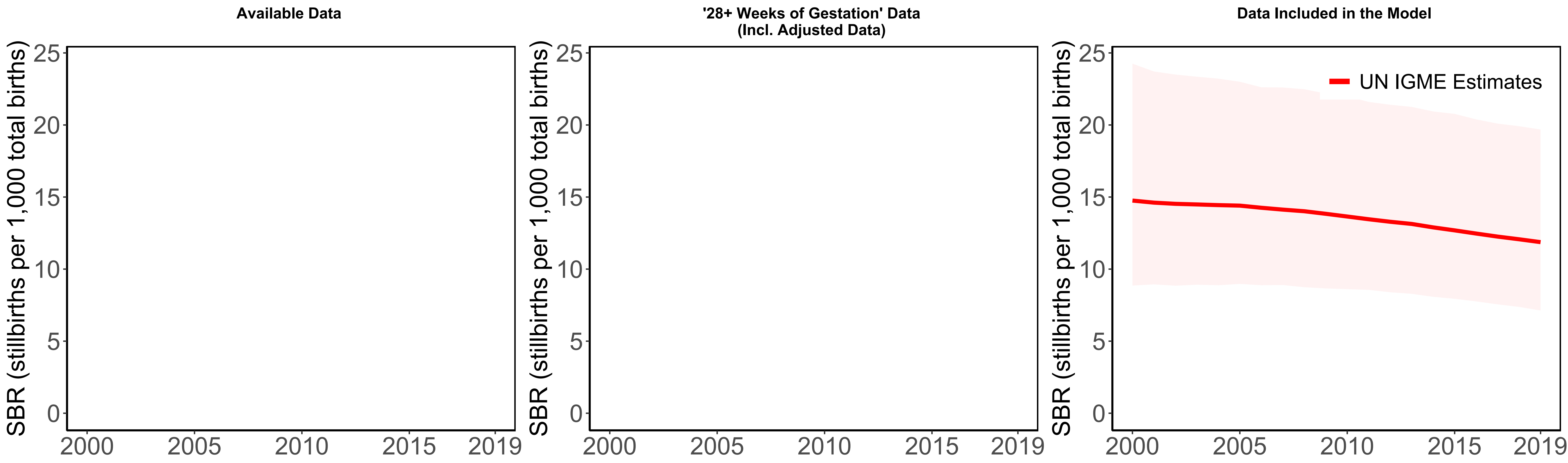
Available Data
'28+ Weeks of Gestation' Data
(Incl. Adjusted Data)
Data Included in the Model
UN IGME Estimates
SBR (stillbirths per 1,000 total births)
0
5
10
15
20
25
2000
2005
2010
2015
2019

United Republic of Tanzania

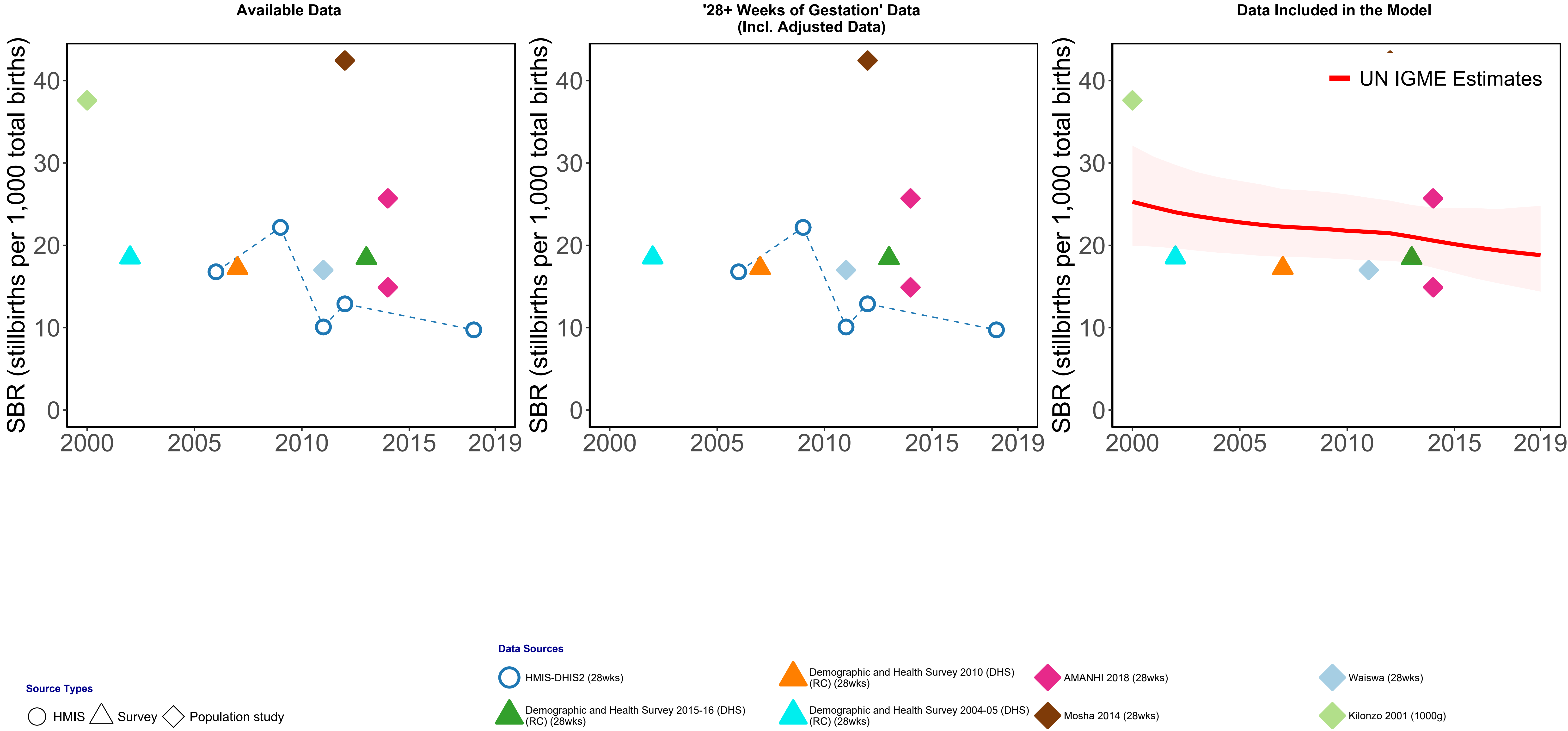

Uganda

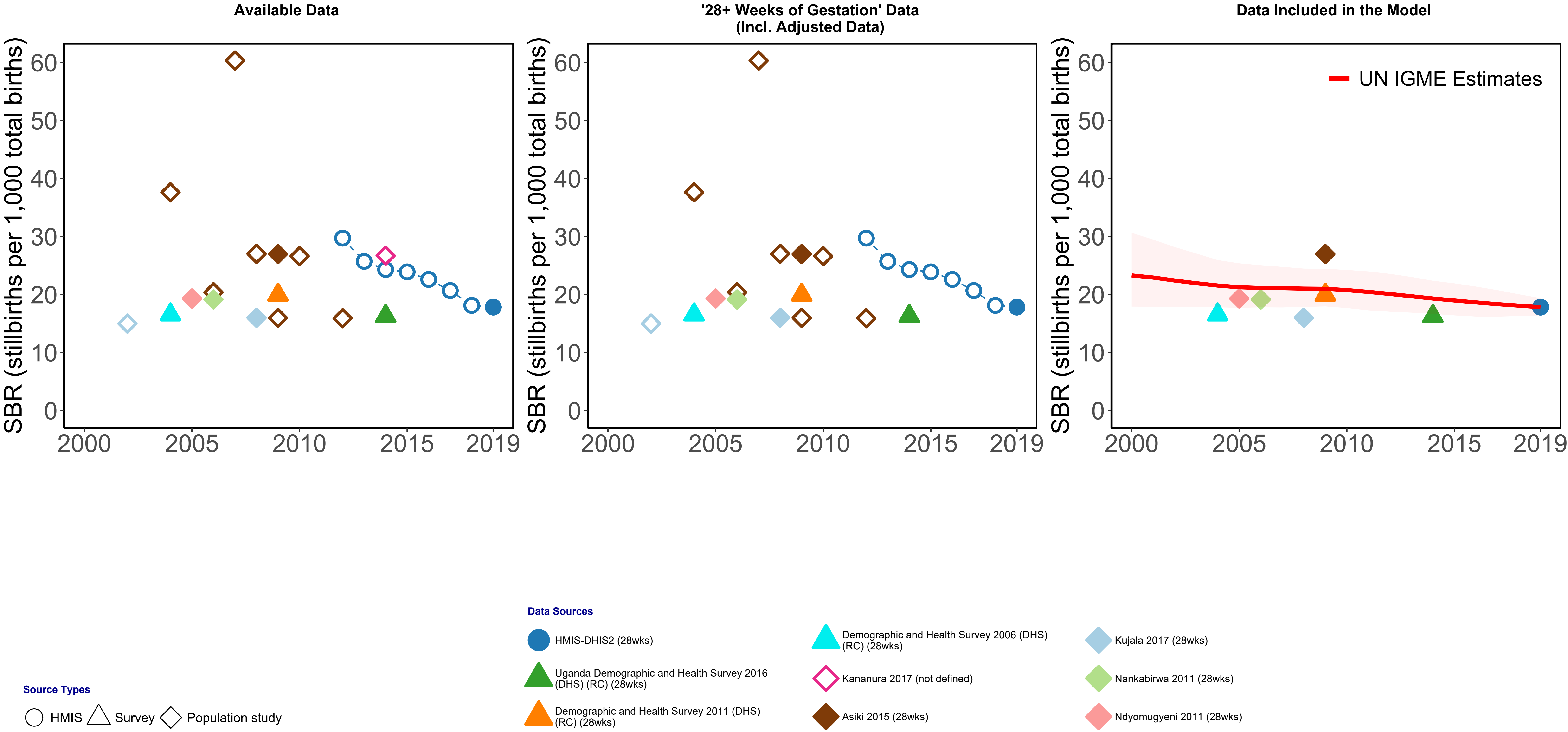

Available Data
'28+ Weeks of Gestation' Data
(Incl. Adjusted Data)
Data Included in the Model
SBR (stillbirths per 1,000 total births)
0
10
20
30
40
50
60
2000
2005
2010
2015
2019
UN IGME Estimates
Source Types
HMIS
Survey
Population study
Data Sources
HMIS-DHIS2 (28wks)
Uganda Demographic and Health Survey 2016 (DHS) (RC) (28wks)
Demographic and Health Survey 2011 (DHS) (RC) (28wks)
Demographic and Health Survey 2006 (DHS) (RC) (28wks)
Kananura 2017 (not defined)
Asiki 2015 (28wks)
Kujala 2017 (28wks)
Nankabirwa 2011 (28wks)
Ndyomugyeni 2011 (28wks)

Ukraine

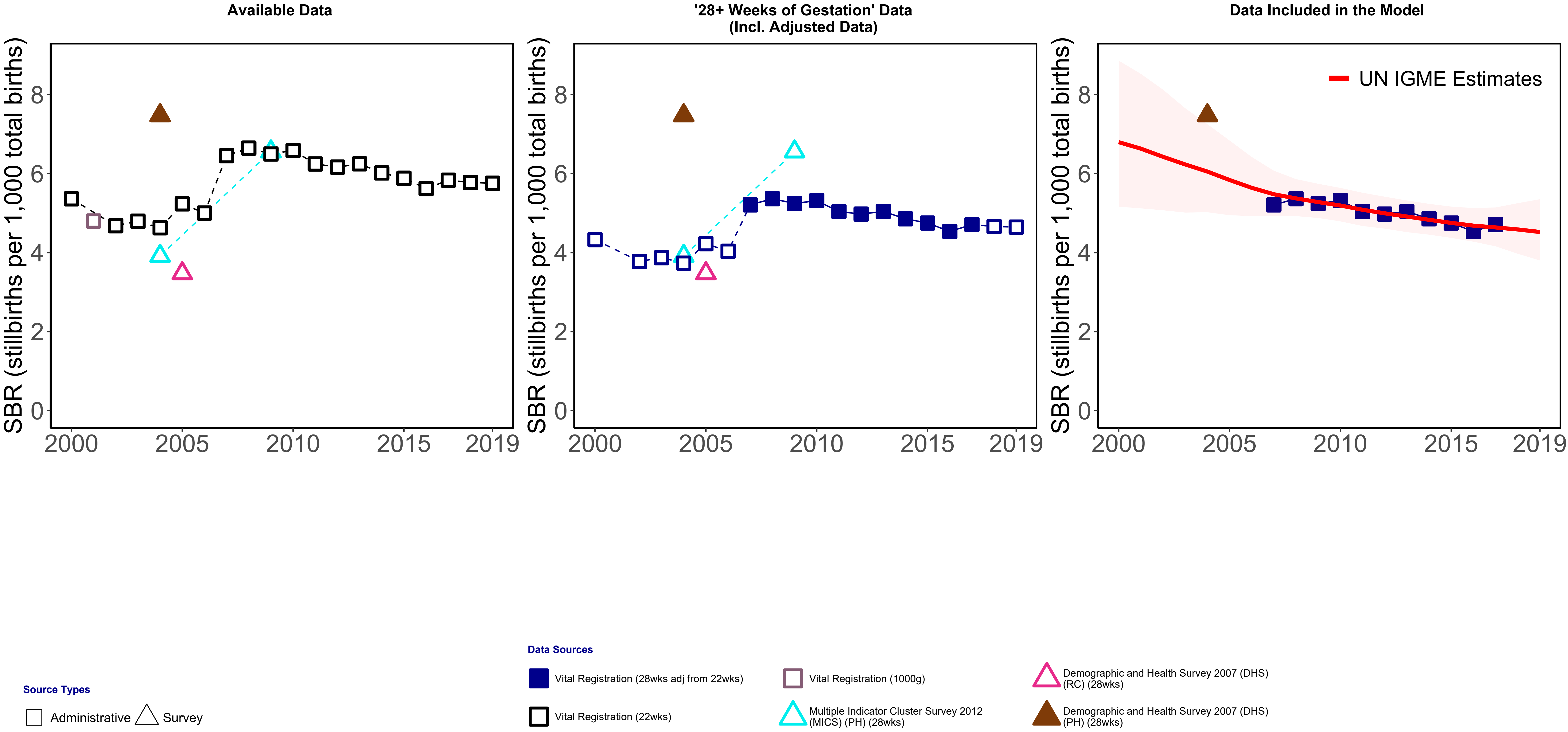

Available Data
'28+ Weeks of Gestation' Data
(Incl. Adjusted Data)
Data Included in the Model
SBR (stillbirths per 1,000 total births)
0
2
4
6
8
2000
2005
2010
2015
2019
UN IGME Estimates
Source Types
Administrative
Survey
Data Sources
Vital Registration (28wks adj from 22wks)
Vital Registration (22wks)
Vital Registration (1000g)
Multiple Indicator Cluster Survey 2012 (MICS) (PH) (28wks)
Demographic and Health Survey 2007 (DHS) (RC) (28wks)
Demographic and Health Survey 2007 (DHS) (PH) (28wks)

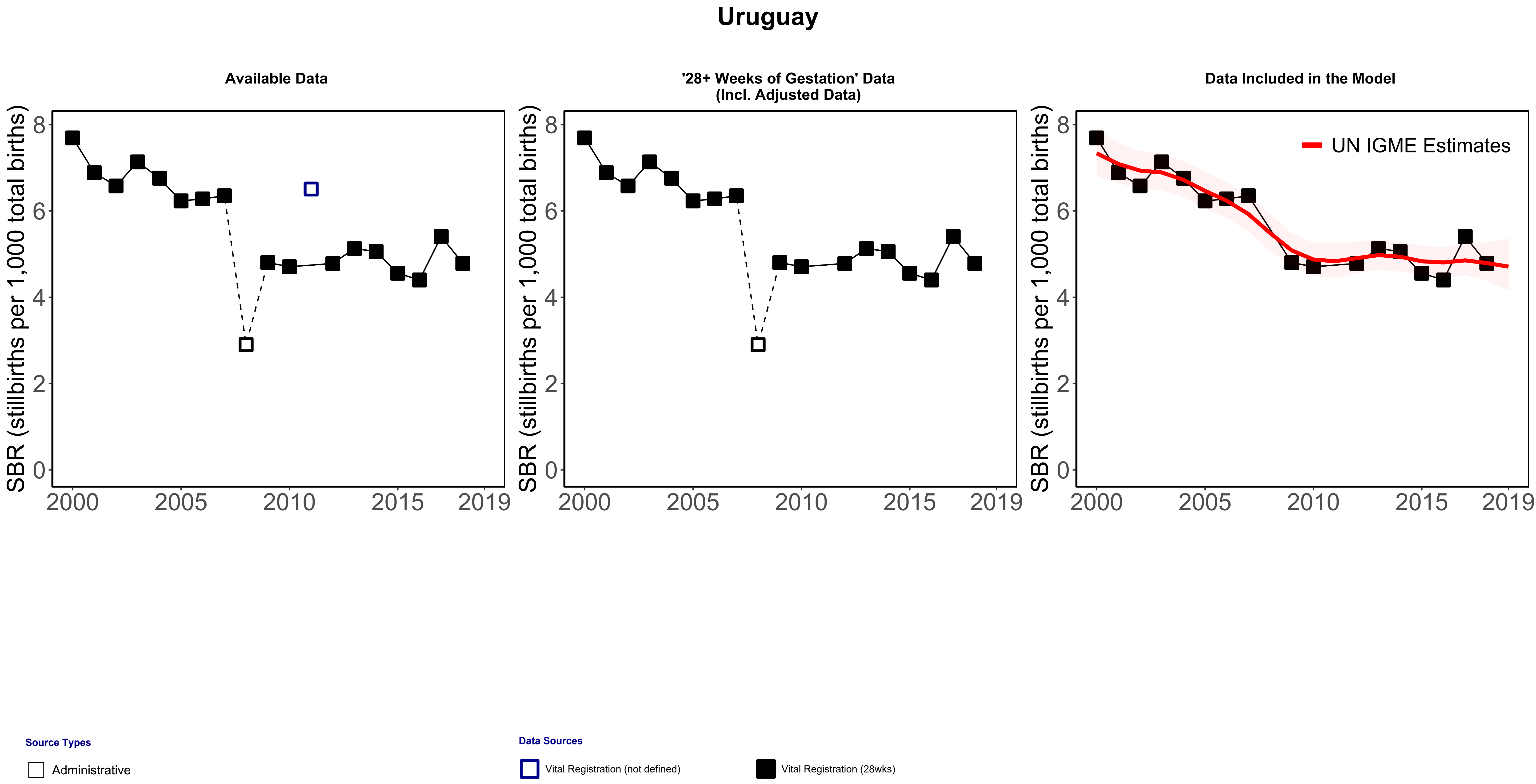
Uruguay
Available Data
'28+ Weeks of Gestation' Data
(Incl. Adjusted Data)
Data Included in the Model
SBR (stillbirths per 1,000 total births)
UN IGME Estimates
0
2
4
6
8
2000
2005
2010
2015
2019
Source Types
Administrative
Data Sources
Vital Registration (not defined)
Vital Registration (28wks)

United States of America

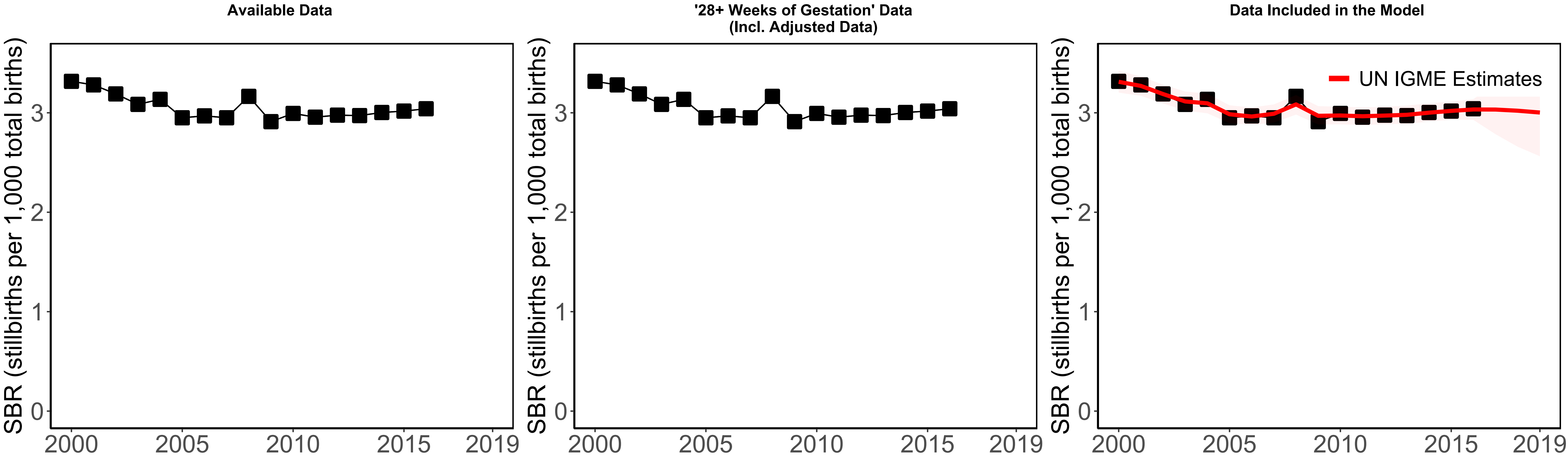

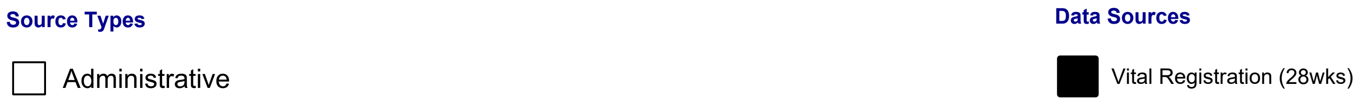

Uzbekistan

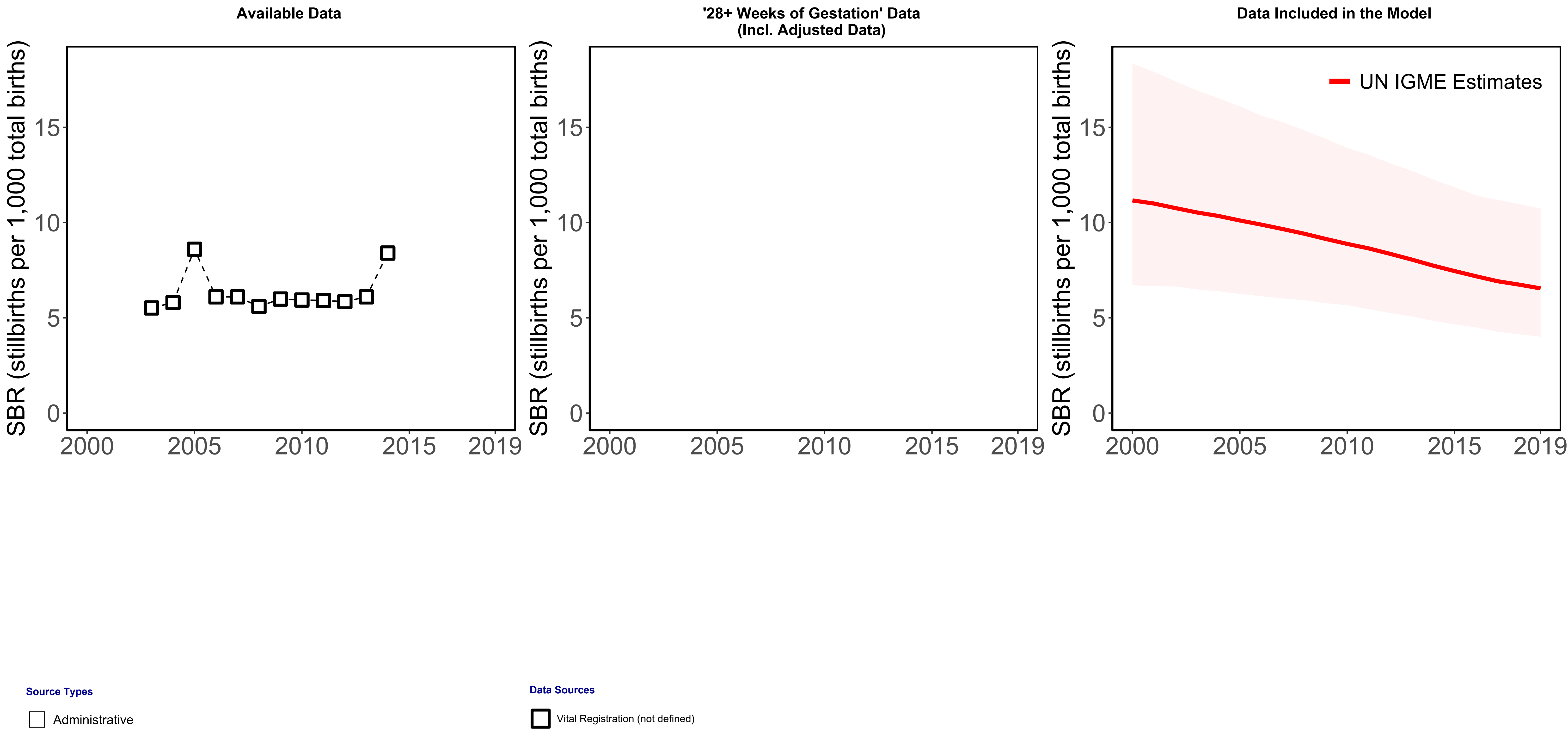
Available Data
'28+ Weeks of Gestation' Data
(Incl. Adjusted Data)
Data Included in the Model
SBR (stillbirths per 1,000 total births)
UN IGME Estimates
0
5
10
15
2000
2005
2010
2015
2019
Source Types
Administrative
Data Sources
Vital Registration (not defined)

Saint Vincent and the Grenadines

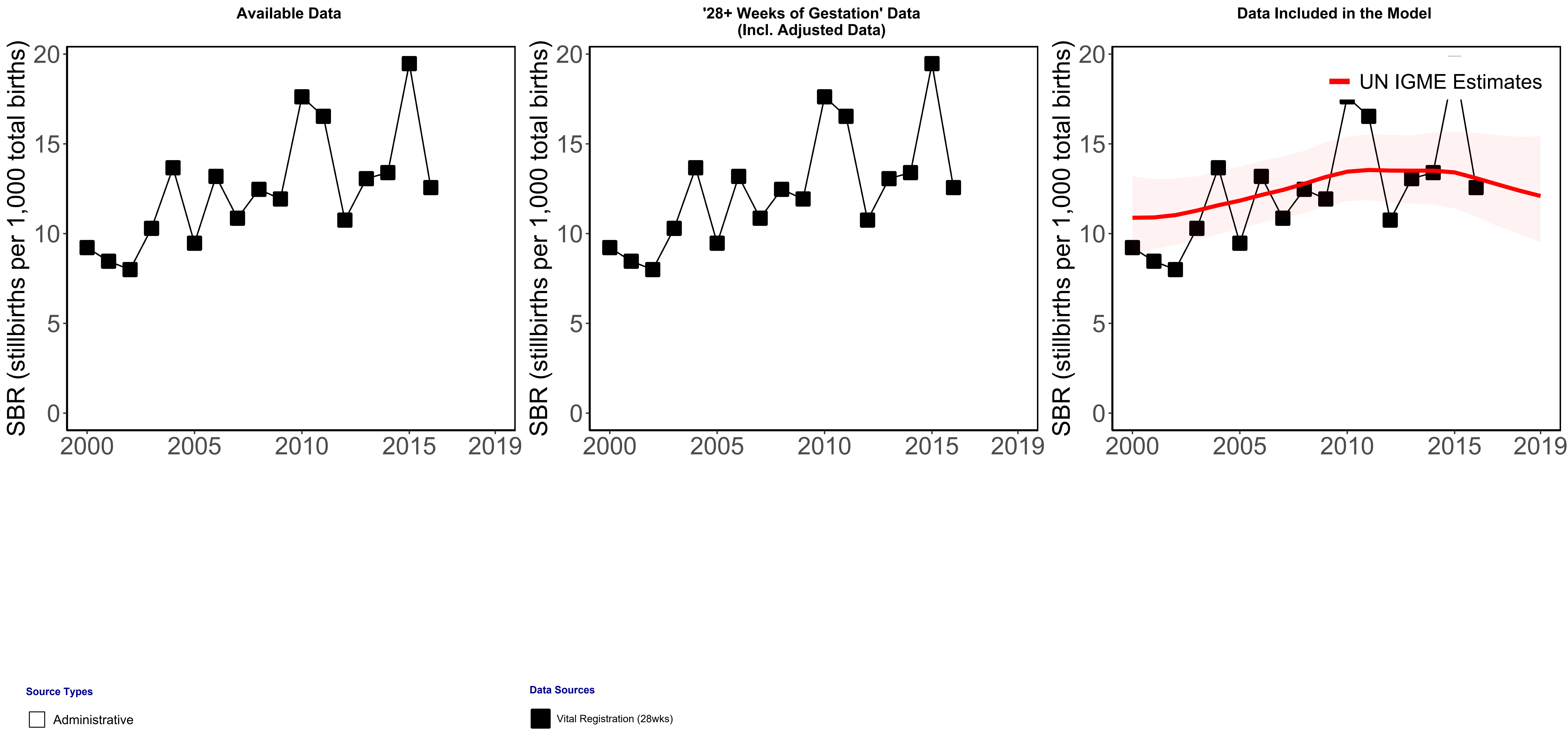

Venezuela (Bolivarian Republic of)

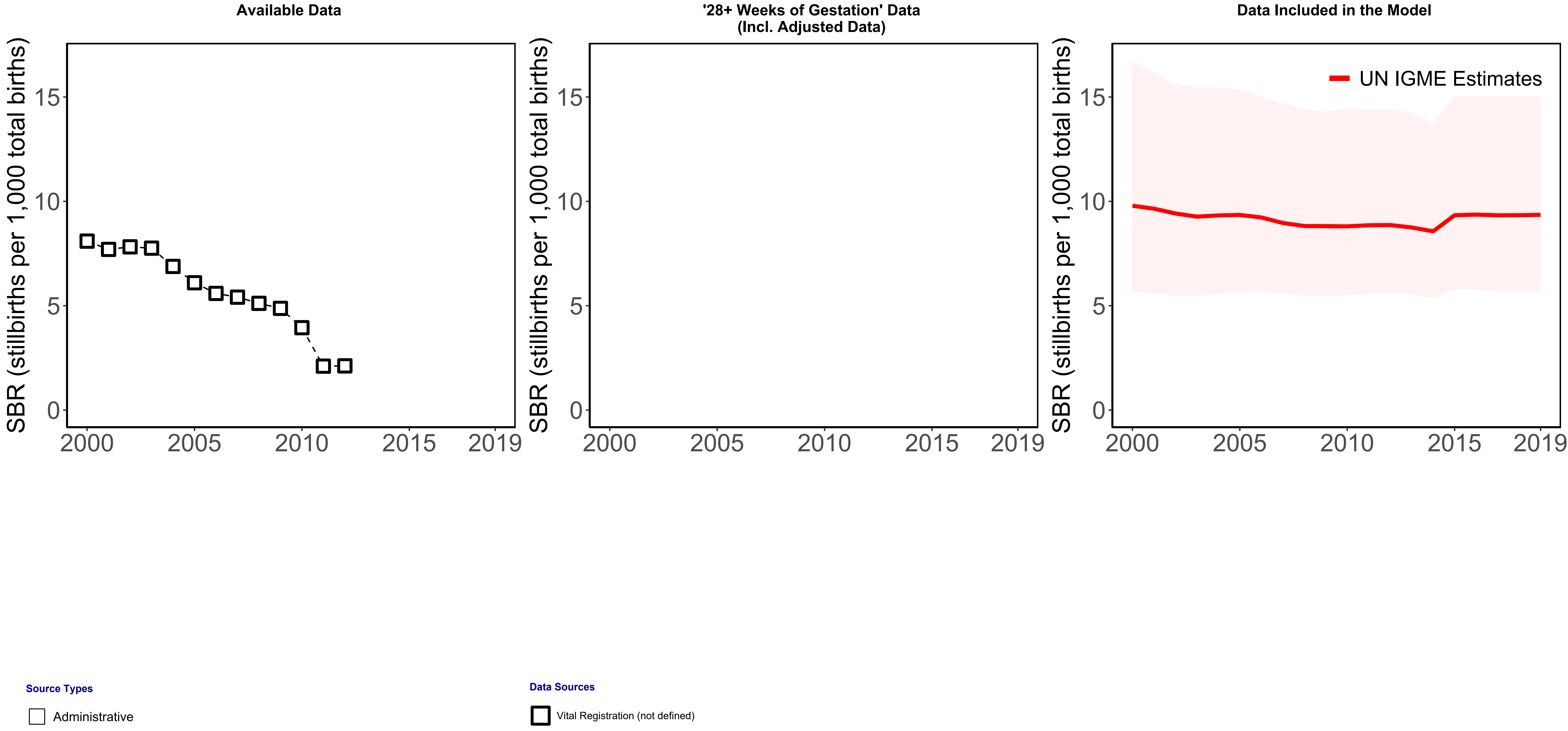

Vietnam

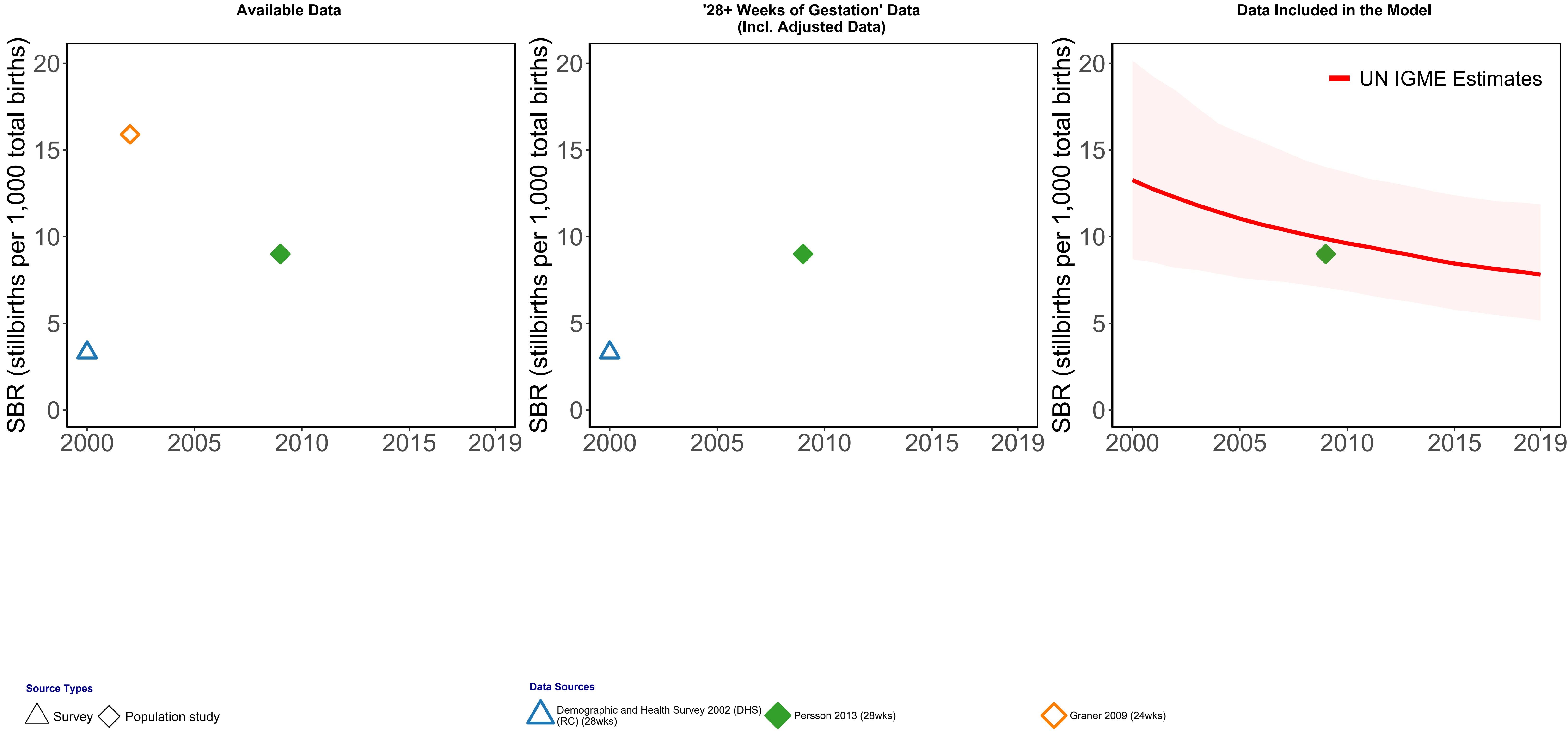

Available Data
'28+ Weeks of Gestation' Data
(Incl. Adjusted Data)
Data Included in the Model
SBR (stillbirths per 1,000 total births)
UN IGME Estimates
0
5
10
15
20
2000
2005
2010
2015
2019
Source Types
Survey
Population study
Data Sources
Demographic and Health Survey 2002 (DHS) (RC) (28wks)
Persson 2013 (28wks)
Graner 2009 (24wks)

Vanuatu

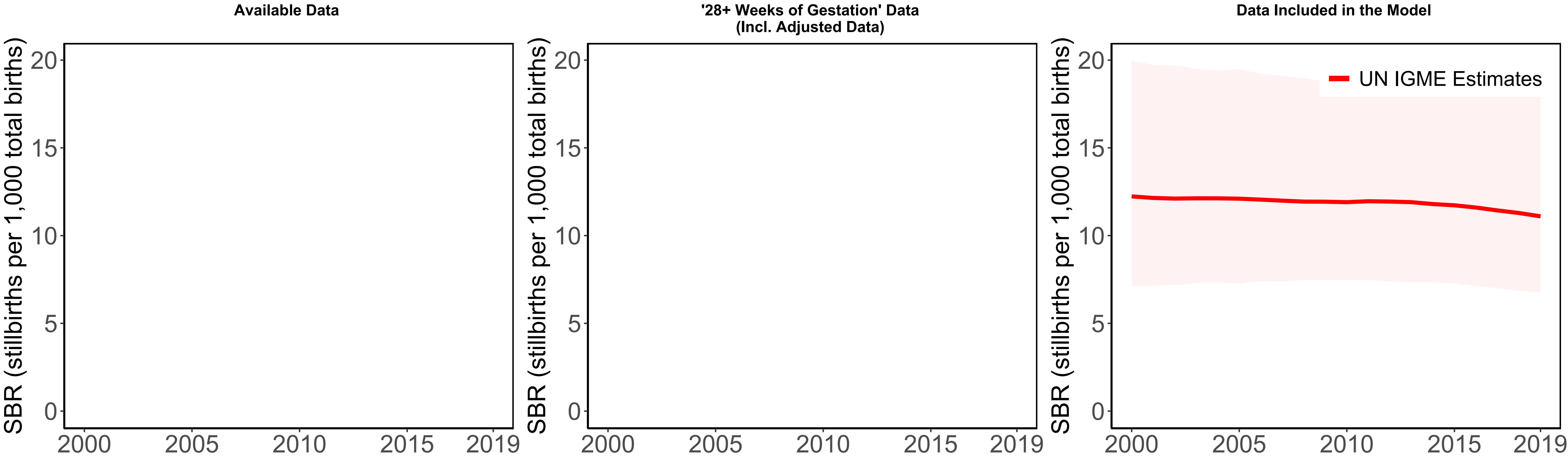

Available Data
'28+ Weeks of Gestation' Data
(Incl. Adjusted Data)
Data Included in the Model
SBR (stillbirths per 1,000 total births)
0
5
10
15
20
2000
2005
2010
2015
2019
UN IGME Estimates

Samoa

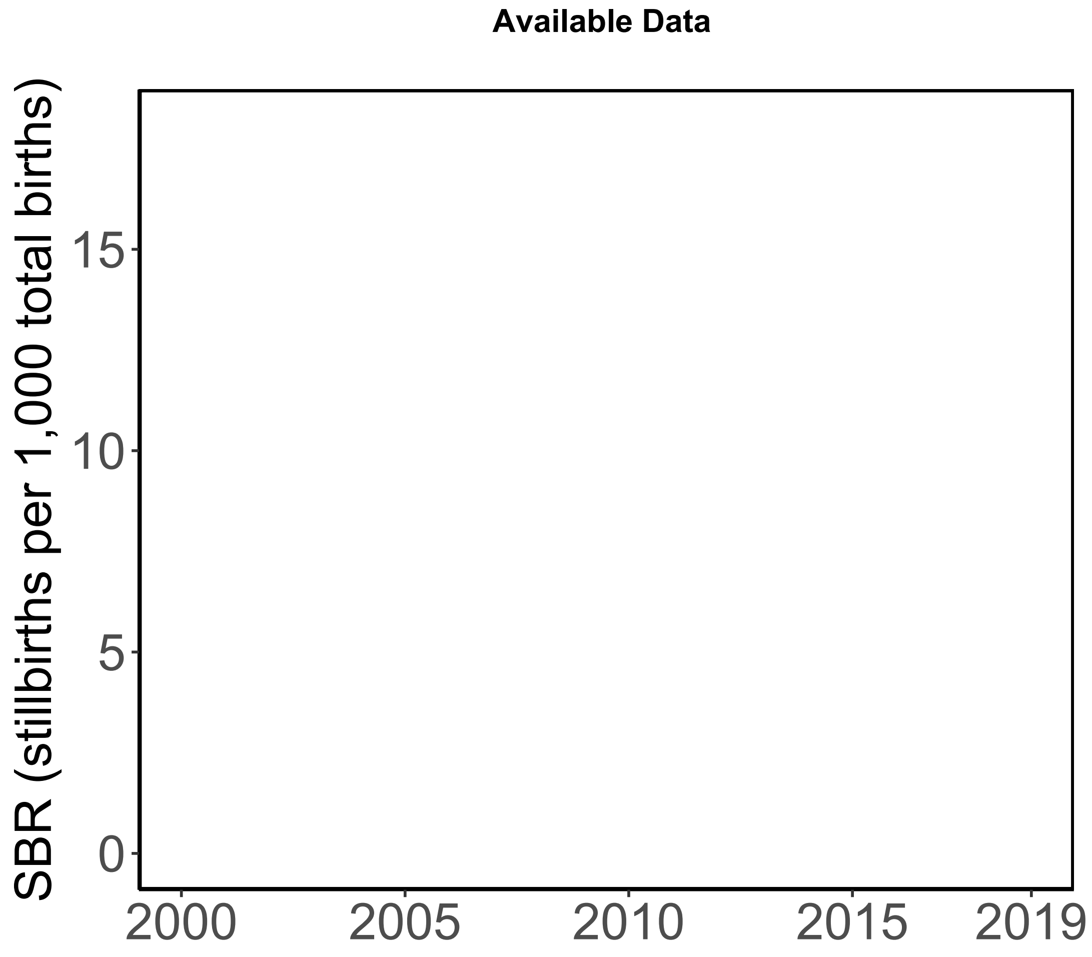
Available Data
SBR (stillbirths per 1,000 total births)
0
5
10
15
2000
2005
2010
2015
2019

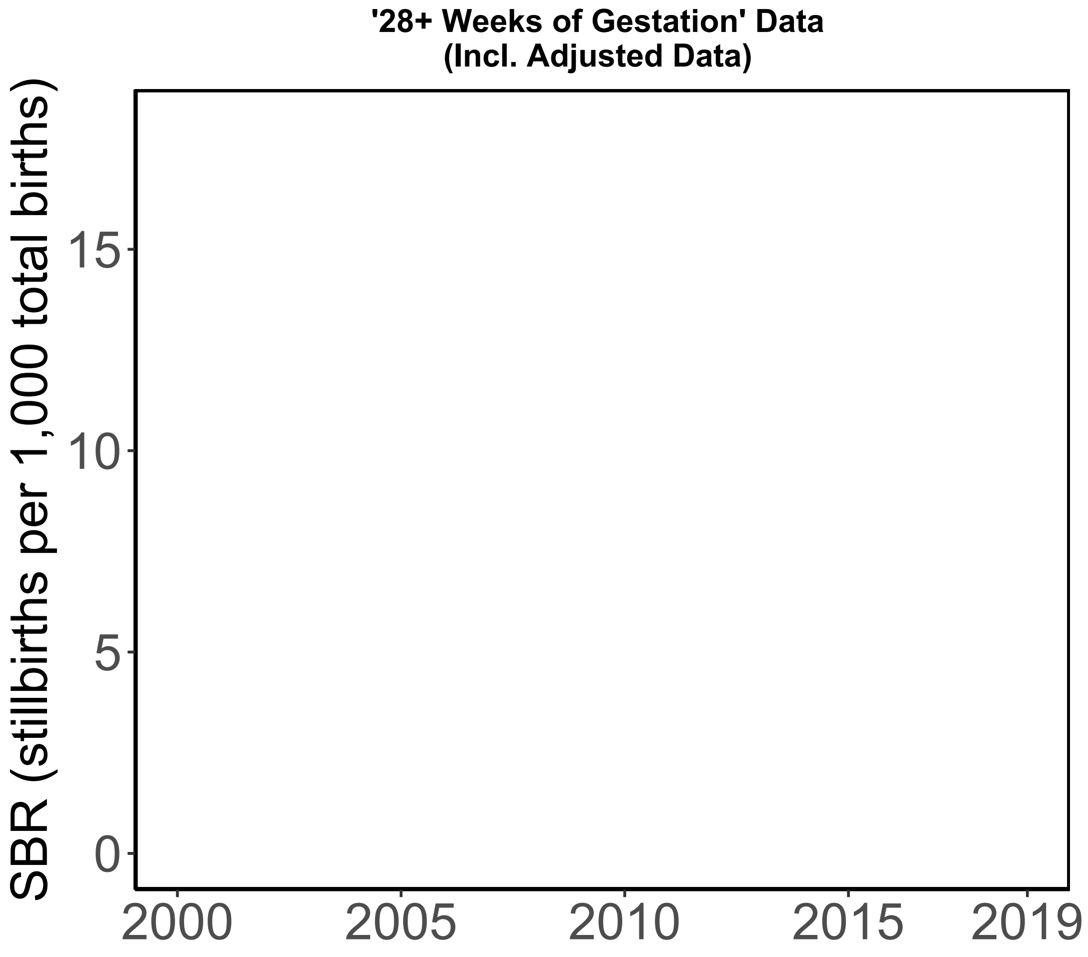
'28+ Weeks of Gestation' Data
(Incl. Adjusted Data)
SBR (stillbirths per 1,000 total births)
0
5
10
15
2000
2005
2010
2015
2019

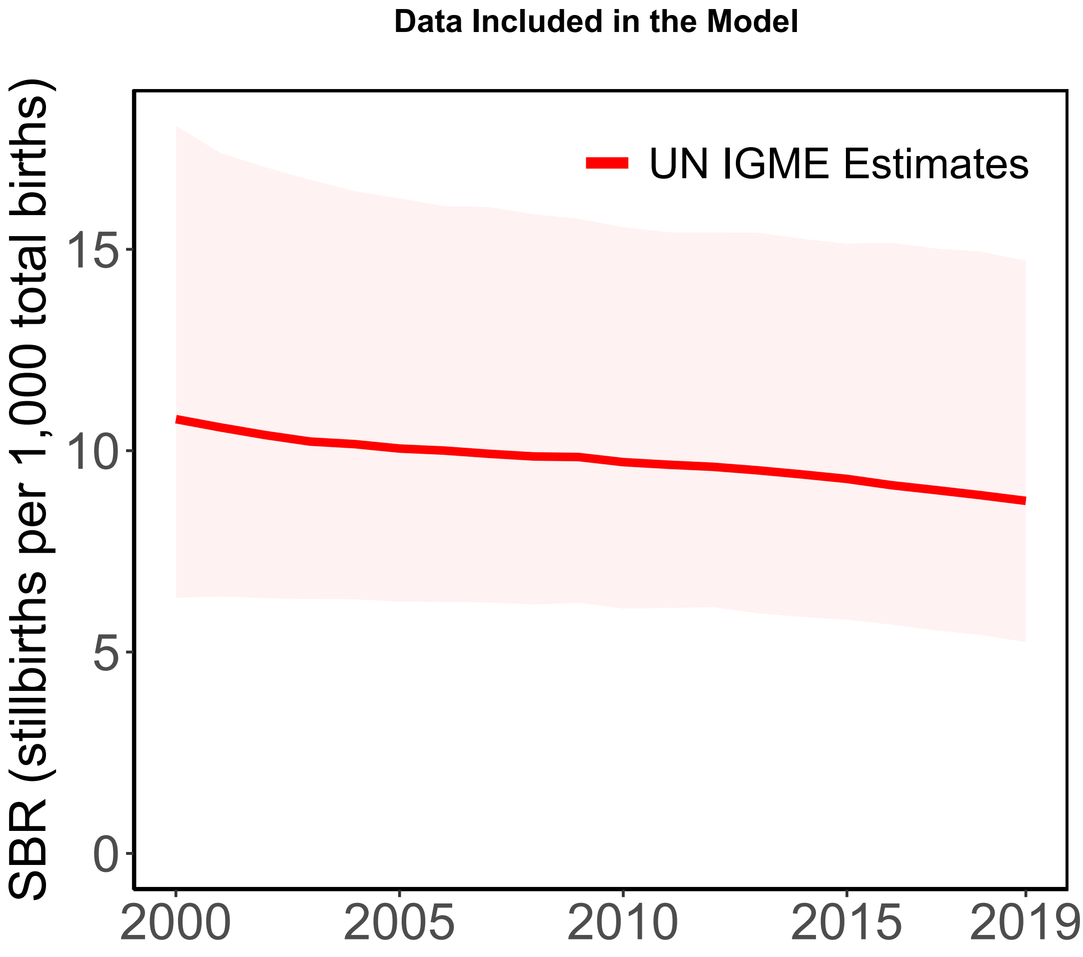
Data Included in the Model
UN IGME Estimates
SBR (stillbirths per 1,000 total births)
0
5
10
15
2000
2005
2010
2015
2019

Yemen

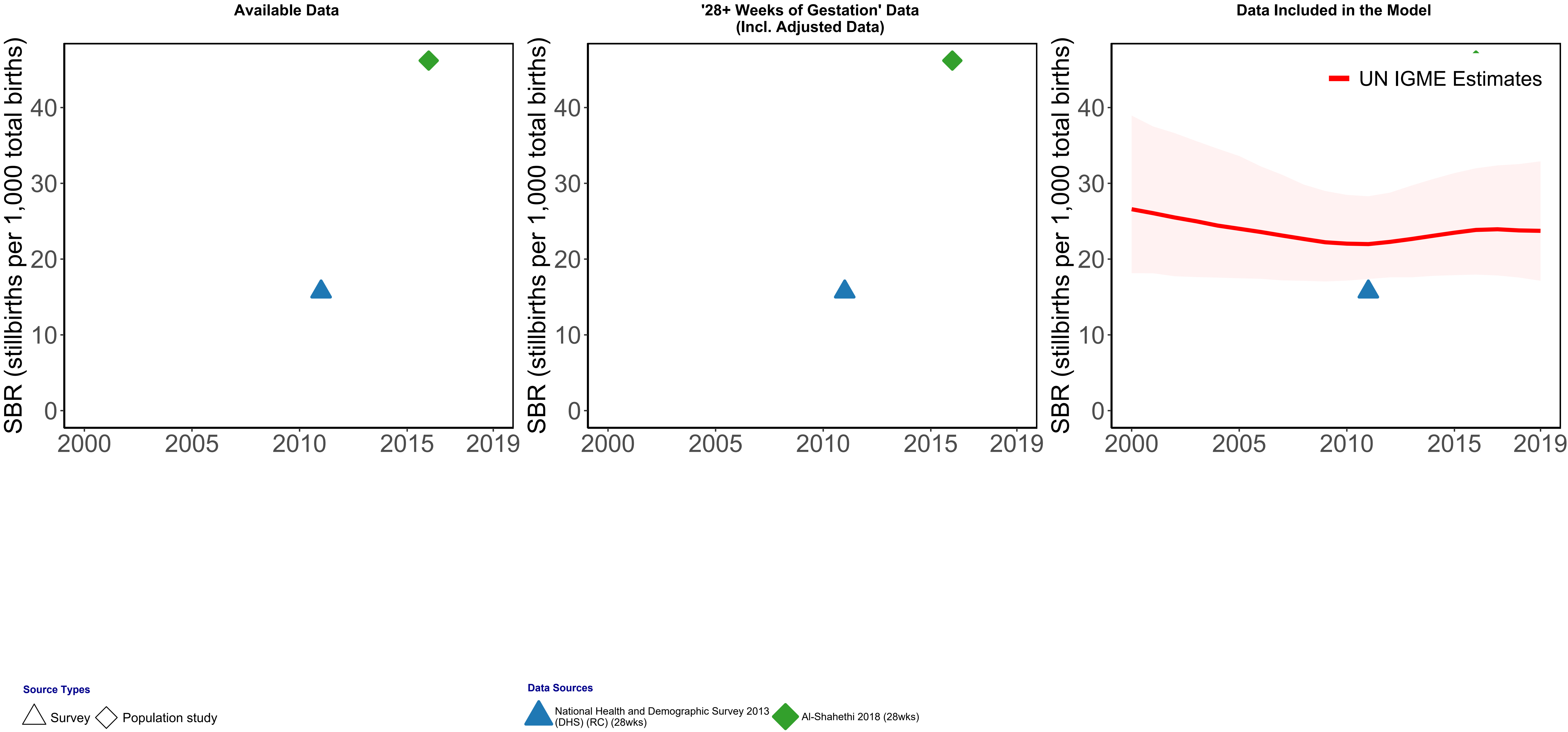

Available Data
'28+ Weeks of Gestation' Data
(Incl. Adjusted Data)
Data Included in the Model
UN IGME Estimates
SBR (stillbirths per 1,000 total births)
0
10
20
30
40
2000
2005
2010
2015
2019
Source Types
Survey
Population study
Data Sources
National Health and Demographic Survey 2013 (DHS) (RC) (28wks)
Al-Shahethi 2018 (28wks)

South Africa

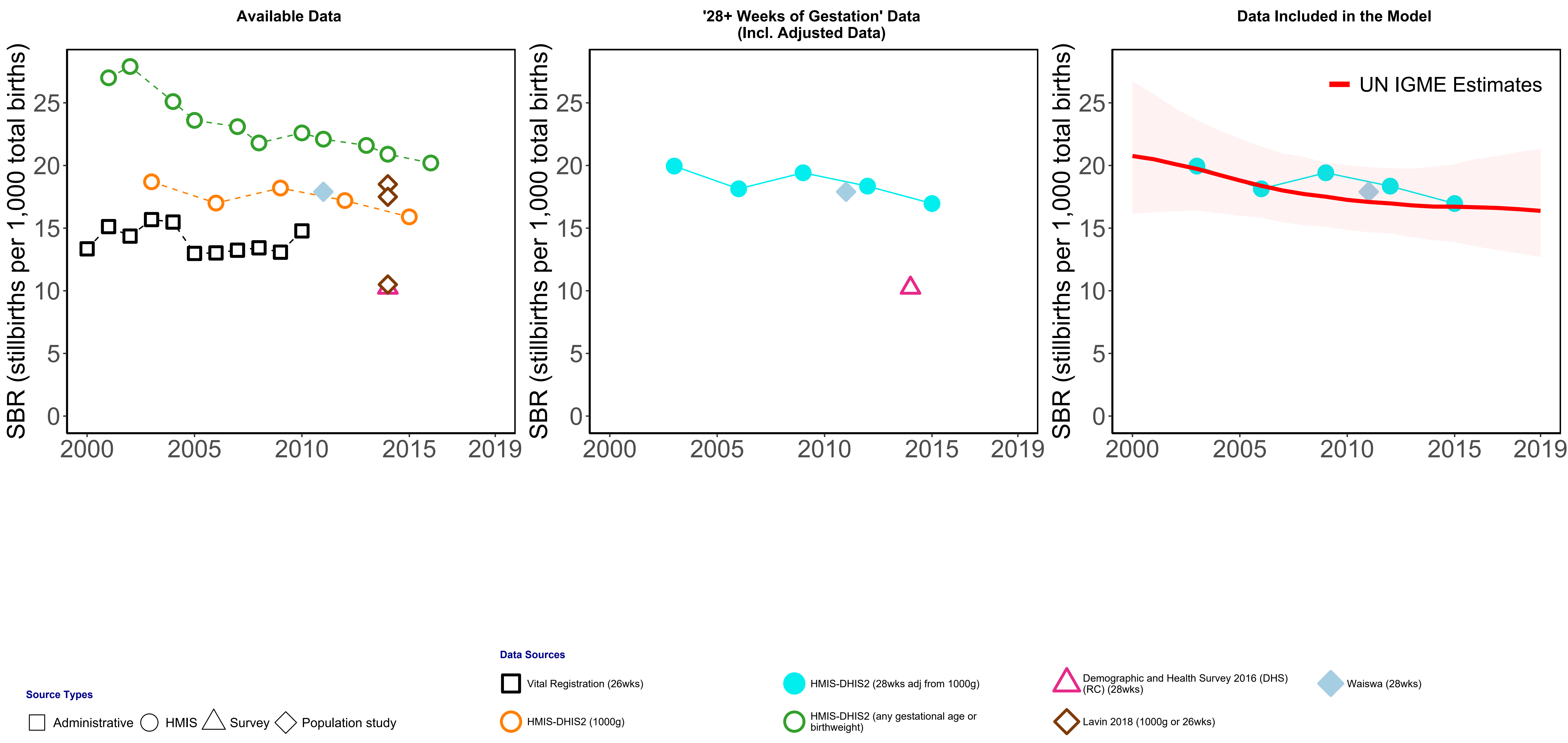

Zambia

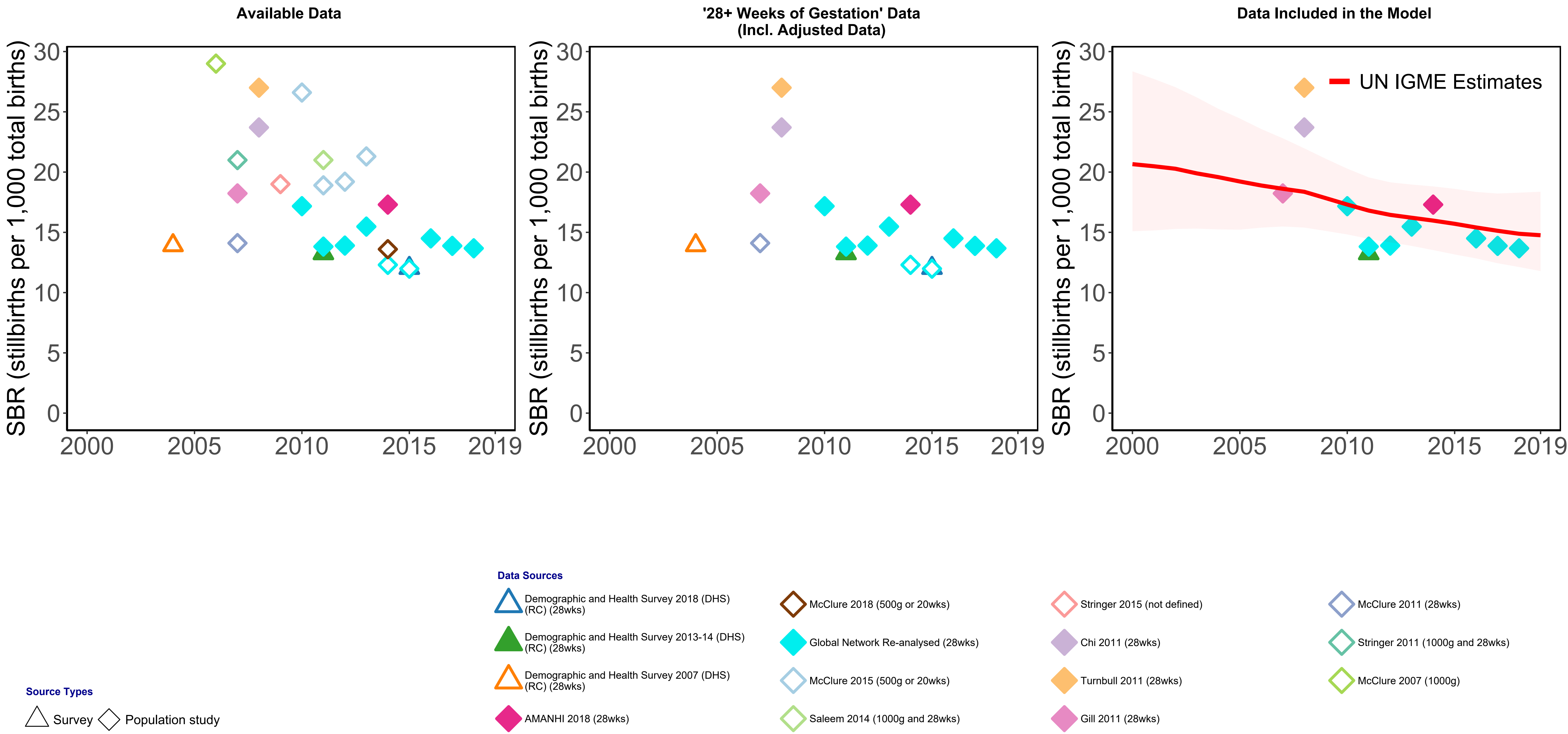
Available Data
'28+ Weeks of Gestation' Data
(Incl. Adjusted Data)
Data Included in the Model
SBR (stillbirths per 1,000 total births)
0
5
10
15
20
25
30
2000
2005
2010
2015
2019
UN IGME Estimates
Data Sources
Demographic and Health Survey 2018 (DHS) (RC) (28wks)
Demographic and Health Survey 2013-14 (DHS) (RC) (28wks)
Demographic and Health Survey 2007 (DHS) (RC) (28wks)
AMANHI 2018 (28wks)
McClure 2018 (500g or 20wks)
Global Network Re-analysed (28wks)
McClure 2015 (500g or 20wks)
Saleem 2014 (1000g and 28wks)
Stringer 2015 (not defined)
Chi 2011 (28wks)
Turnbull 2011 (28wks)
Gill 2011 (28wks)
McClure 2011 (28wks)
Stringer 2011 (1000g and 28wks)
McClure 2007 (1000g)
Source Types
Survey
Population study

Zimbabwe

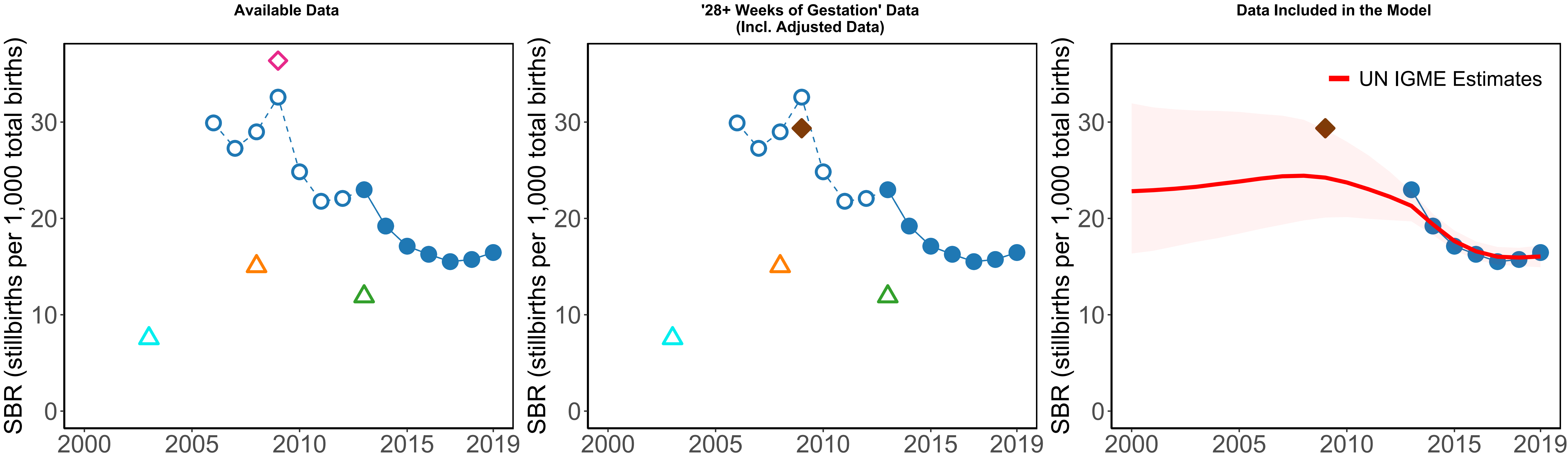

Source Types

HMIS Survey Population study

Data Sources

HMIS-DHIS2 (28wks)

Demographic and Health Survey 2015 (DHS) (RC) (28wks)

Demographic and Health Survey 2010-11 (DHS) (RC) (28wks)

Demographic and Health Survey 2005-06 (DHS) (RC) (28wks)

Tachiweyika 2011 (22wks)

Tachiweyika 2011 (28wks adj from 22wks)
